# Detailed Density Functional Theory Study of the Cationic Zirconocene Compound [Cp(C_5_H_4_CMe_2_C_6_H_4_F)ZrMe]^+^

**DOI:** 10.1021/acsomega.2c04053

**Published:** 2022-09-22

**Authors:** Jörg Saßmannshausen

**Affiliations:** Imperial College London, South Kensington Campus, London SW7 2AZ, U.K.

## Abstract

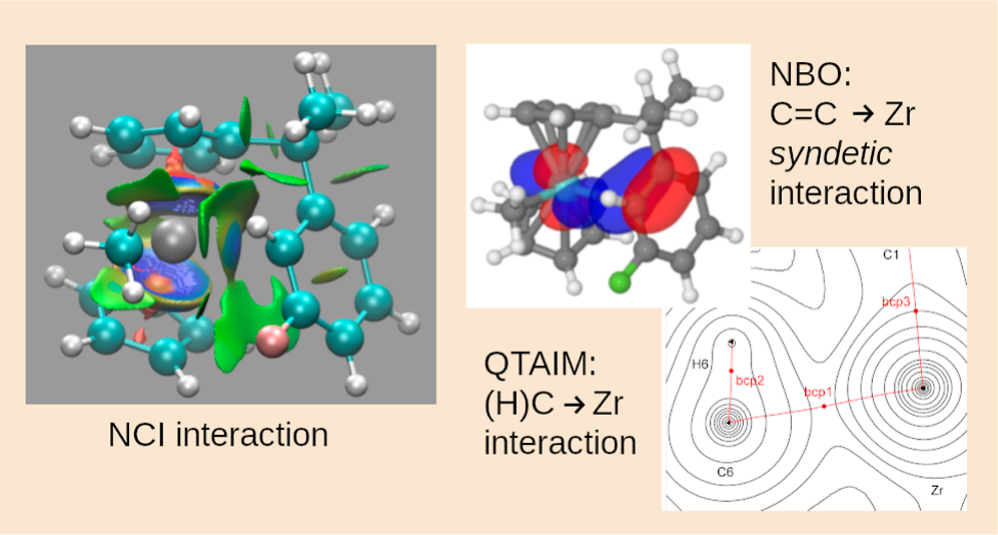

Detailed density functional theory studies at the B3LYP and PBE-D3 levels of theory were performed on the cationic compound [Cp(C_5_H_4_CMe_2_C_6_H_4_F)ZrMe]^+^, with the F atom occupying either the ortho, meta, or para positions of the arene ring. In all cases, the arene ring coordinates with the cationic zirconium metal. The nature of this coordination is such that for the meta- or para-substituted arene ring, it is predominantly the ortho carbon atom of the C–H bond which interacts with the metal, as evident from noncovalent interaction studies. This is further corroborated by the natural bond orbital and quantum theory of atoms in molecular studies. In the case of the F atom being in the ortho position, we obtained clear-cut evidence for a Zr–F coordination.

## Introduction

Transition-metal compounds are often used as catalysts for organic reactions such as C–C bond formation and C–H activation. In particular, the latter is of current interest as it enables the functionalization of hydrocarbons.

In this respect, the discovery around 30 years ago that hydrogen can be in close proximity to a transition metal but is not bound to it was significant, so much so that the term *agostic* was coined by Malcolm Green et al.^1−4^ In recent years, the concept of a hydrogen atom being close to a transition metal but not yet bonded in the classical sense has been expanded, and terms such as *anagostic*(5,6) or *pregostic*(7) have found their way into textbooks. Indeed, quite recently this concept has been expanded to a *reverse* agostic bond, where hydrogens bonded to a transition metal can interact with group 14 atoms such as silicon.^8^

It should be noted that these entities are not of purely academic interest. They play a vital role in the understanding of chemical reactions as they can be considered as “frozen” transition states in C–H activation. Indeed, in a recent study regarding the *reversed* agostic bond interaction between a Ru–H and a Si ligand, evidence of activation of the Ru–H bond by means of X-ray radiation was obtained.^9^ Clearly, without such a *reversed* agostic bond interaction, the Ru–H bond would simply split and the whole molecule just disintegrates.

**Scheme 1:**
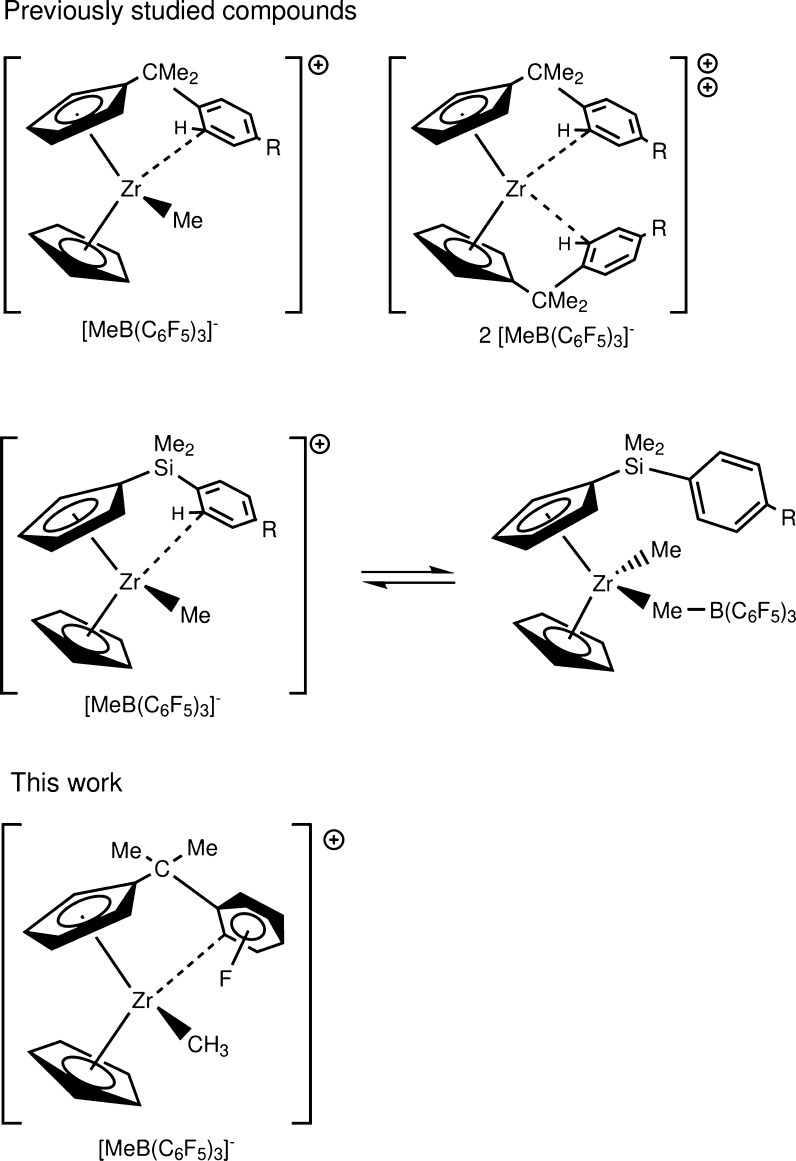
Some Previously Studied Compounds (Top) and the F-Substituted Ones in This Work (Bottom)

A clear understanding of these processes enables us to tailor-make catalysts which either suppress, for example, C–H activation, which might be desirable in olefin polymerization, or enhance it so that functionalization of alkanes can occur. It is thus not surprising that research groups were looking into examples of late transition metals such as Rh^7,10−12^ and Ru^7,12,13^ and early ones such as Zr or Ti.^14^ In particular, for olefin polymerization with group 4 metals, the process of β-H elimination is now well understood, thanks to a number of both experimental and computational contributions.^15−25^ To this end, we have been interested for some time in the coordination of an arene ring to cationic group 4 metals such as titanium and zirconium.^14,26−31^ These compounds could be seen as a surrogate for solvent coordination to cationic group 4 metals, for example. Furthermore, they are also ideal model compounds to probe the cationic group 4 metal–H–C interaction. In these compounds, the arene is usually tethered to the cyclopentadienyl (Cp) ligand via a single atom such as C or Si or a longer carbon chain.^29,30,32−37^ Depending on the design of the ligand, we can achieve exclusive coordination of the arene with a carbon tether or a more fluxional process with a silicon one where exchange with the anion is still possible.^26,29,33^ A very long carbon chain makes this coordination less likely although.^37^ This motif can be taken further: *dicationic* zirconocene compounds can be prepared at −60 °C and are excellent initiators for carbocationic isobutene polymerization, for example.^38^ Our studies have shown very early that the coordination is more via the ortho carbon atom than via the ortho C–H bond, that is, these compounds are strictly speaking not agostic.^28^ In fact, we noticed some time ago in some cases an interaction of the C–C double bond of the arene with the cationic zirconium metal.^14,30^ This type of coordination mode is now called *syndetic*.^12^ Previous experimental studies included a CH_3_ group in the para position of the arene ring, which merely served as a NMR marker group (cf. Scheme 1). However, given the size of the CH_3_ group, we reasoned that it would not be feasible to place it in any other position than para. The F atom is quite remarkable in the respect that it is smaller than H (atomic radii: F: 42 pm; H: 53 pm)^39^ and thus is often used in medicinal chemistry as a replacement for H to enhance the activity of a drug. As it is the most electronegative element, it does have a large −I effect, but this effect is often masked by a strong +M effect. Thus, the presence of a F atom directs an incoming substituent predominantly in the ortho and para positions. Assuming that the positive Zr acts as an incoming group and by replacing H with F in the ortho, meta, or para positions of the arene ring, respectively, we have a very good handle for changing the electron density within the arene ring and probing the interaction with the cationic zirconium metal (cf. Scheme 1). To expand our studies, we are not only using the well-established B3LYP functional but also the recently reported PBE-D3, which includes the dispersion correction from Grimme et al.^28^

## Results

### Choice of Computational Method

We utilize two different density functional theory (DFT) functionals for this work, namely, the previously used B3LYP functional,^40,41^ which will give a reference point to previously published work, and the more recently developed DFT-D method, namely, the PBE-D3 functional. The PBE-D3 functional consists of the Perdew–Burke–Ernzerhof exchange–correlation functional^42^ with Grimme’s empirical correction D3 been added.^43−49^ The reasoning behind it is as follows: it is well known that DFT is not good at capturing long-range interactions, such as, for example, dispersion forces. This shortcoming was addressed in the development of the DFT-D approach which tries to capture these forces by means of an empirical correction. The PBE functional was chosen as it is less computationally expensive for plane-wave methods which are typically being used to model for example molecular dynamic simulations. This allows us to use more realistic model compounds and more often real structures, rather than a “cut-down” version of the real molecule. Hence, we achieve a number of things:

- have reference points between a typical DFT functional (B3LYP) and a typical plane-wave one (PBE), which, by the inclusion of the dispersion, will achieve similar accuracy^48,49^
- calculate larger, more realistic systems with better accuracy but less computational cost, important, for example, for molecular dynamics calculations.

A triple zeta basis set was employed for these calculations consisting of Pople’s 6-311G(d,p) basis set for all elements^50^ but for zirconium where the Stuttgart–Dresden effective core potential was used. This basis set combination is an extension of the previously used ecp1 basis set^28^ and thus will be abbreviated as ecp11 in the remaining manuscript. For the natural bond orbital (NBO), quantum theory of atoms in molecules (QTAIM), and NCI calculation the Stuttgart–Dresden basis set was replaced with the all-electron DZVP^51^ basis set for Zr only. Again, this combination is an extension of the previously used basis set combination. All basis sets were downloaded from the Basis Set Exchange Website.^52−54^

In order to obtain an insight into the coordination mode of the arene to the cationic zirconium metal, five different methods were employed:

- Natural bond orbital (NBO)^55^
- Natural resonance theory (NRT)^56^
- Quantum theory of atoms in molecules (QTAIM)^57^
- Noncovalent interactions (NCIs)^58^
- Chemical shift calculations (NMR)^28^

All five methods should be considered as different “views” of the same interaction and should not be taken as competing against each other. From NBO and QTAIM, the charges of the relevant atoms were obtained. These charges are summarized in Table 4. Some of the results obtained by QTAIM indicate a H···H interaction between the aromatic rings. We are aware of the ongoing discussion about this kind of interaction, so we are not considering them until this topic has been settled.^59^

As part of the new NBO7 program, we were also looking into the NRT,^60^ in particular, that of the coordinated arene ring. The motivation behind this is to get a deeper insight into the effect the F substituent has on the coordination of the cationic Zr to the arene ring.

Finally, for the B3LYP structures, the chemical shifts were computed using the same well-established methodology as our original publication.^28^

In this way, we are able not only to compute a change in the electronic properties of an atom, that is, its shielding, but we are also bridging back our theoretical results to the experimental world as these shifts can be observed by means of NMR spectroscopy. As this method is most reliable with hybrid functionals such as B3LYP, we did not repeat it with the PBE-D obtained structures. It also should be noted that the chemical shift is highly sensitive to the electronic environment of the observed nucleus. Thus, it is also possible to calculate the electron cloud of a bond using an observable method.^61,62^ We thus should be able to get a very detailed and thorough picture of the nature of the coordination modes of these interactions. The results thus obtained are not only of interest for olefin polymerization and oligomerization processes but should also be translatable to organic chemistry, in particular, C–H activation processes, that is, substitution reactions.

### Investigated Compounds

**Scheme 2:**
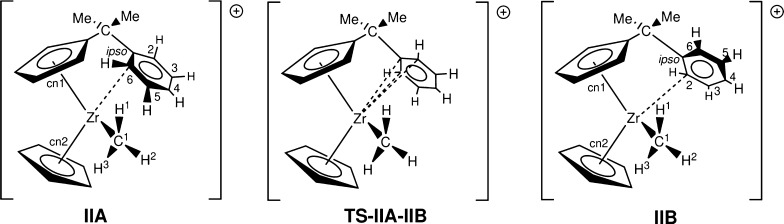
Previously Reported Cationic Zirconocene Compounds

We systematically modified the cationic zirconocene compound in such a way that the F is placed in the ortho, meta, or para position of the arene ring. Furthermore, we explored the two different coordination modes A and B of the arene ring as well. For comparison, we included the already published unsubstituted compound too; however, we have recalculated the structure at both the B3LYP and PBE-D3 level of theory (Scheme 2). We located the transition state of all three isomers, that is, the A → B transition state. In this way, we obtained a complete matrix of all possible structures, which allows us to obtain a comprehensive insight into the nature of these compounds.

The remainder of the paper is split into two parts. We first report structural metrics such as bond distances and angles of all investigated compounds, starting with a comparison of the published X-ray structures of CpC_5_H_4_CMe_2_-*p*-C_6_H_4_CH_3_ZrMe_2_ (**I**) and the previously reported cationic compound [CpC_5_H_4_CMe_2_–C_6_H_5_ZrMe]^+^ (**II**) to compare the two utilized functionals and the results obtained in this study with the previously published ones. We then move on to the computed electronic properties such as QTAIM, NBO, NCI, and chemical shifts. In this way, we should be able to focus more on the similarities and differences, which will enable us to gain a deeper understanding of the interaction. Structures which were computed with the B3LYP functional have no suffix, whereas those computed with the PBE-D3 functional have the -pbe-d suffix. The Supporting Information contains the Cartesian coordinates of all investigated compounds, electron density, Laplacians, and Virial field function plots from the QTAIM analysis, the NBO pictures of relevant interactions, and pictures of the NCI.

### Structural and Energy Investigations

#### Benchmarking against CpC_5_H_4_CMe_2_-*p*-C_6_H_4_CH_3_ZrMe_2_ (**I**)

In order to benchmark the accuracy of the employed methods, that is, how well the structural metrics such as bond distances and angles are reproduced, we computed the structure of the well-known substituted zirconocene compound CpC_5_H_4_CMe_2_-*p*-C_6_H_4_CH_3_ZrMe_2_ (**I**) which has a methyl group in the para position of the tethered arene ring. The relevant metrics of these calculations are summarized in Table 1.

**Table 1:**
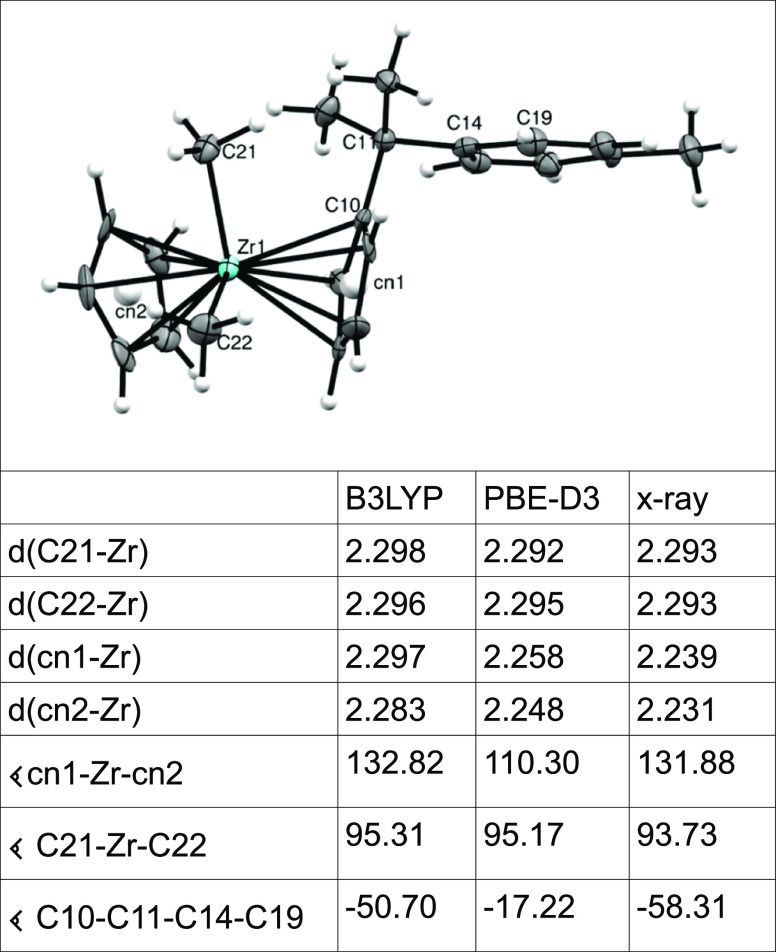
Comparison between the Calculated and Observed Metrics of **I**^a^

a Distances are in Å and angles in degree.

However, the Zr–Me bond distances of the calculated structures are very similar and in very good agreement with the observed structure; for the Cp(center)–Zr distance, the PBE-D3 functional gives results which are in better agreement with the observed structure. However, PBE-D3 seems to over-correct the dispersion forces as it computes a more acute cn1–Zr–cn2 angle (110.30° compared to 131.88° for the X-ray structure); in other words, it opens up the Cp-Zr-Cp wedge and thus exposes the metal more. This over-correction is also observed by the tilting of the attached arene ring which places the ortho C19 atom more over the Cp centroid compared to the results from the B3LYP calculation or X-ray structure (−17.22° compared to −58.31° for the X-ray structure). Thus, when comparing the computed structure with the observed one, this needs to be taken into consideration. Nevertheless, the PBE-D3 results are slightly better than the B3LYP ones which, for example, have been observed for Rh compounds before.^6^

### Comparison between Previously Published and Newly Computed Results of [CpC_5_H_4_CMe_2_–C_6_H_5_ZrMe]^+^ (**II**)

To gain further information, we compare the previously computed results of the unsubstituted zirconocene compounds **IIA** and **IIB** and the transition-state compound **TS-IIA-IIB** with the ones obtained from the larger ecp11 basis set, again, with both functionals.

As it is evident from Table 2, by increasing the basis set from the mixed double zeta ecp1 to the mixed triple zeta ecp11, one does not change some of the key metrics significantly. Thus, we can have some confidence that the increase of the basis set does not significantly change the current results compared with the ones previously reported. The difference between the B3LYP and PBE-D3 functionals is more marked although. In the case of the PBE-D3 functional, the bond distances are generally speaking shorter compared to those of the B3LYP functional. For example, the cn1–Zr distance in **IIA** is 2.227 Å compared to 2.205 Å for PBE-D3. This does have an effect on the observed angles as well (cf. cn1–Zr–cn2: 134.19° (B3LYP); 134.95° (PBE-D3) for **IIA**). These differences could be attributed to the inclusion of the dispersion forces in PBE-D3, which are missing in B3LYP.

**Table 2:**
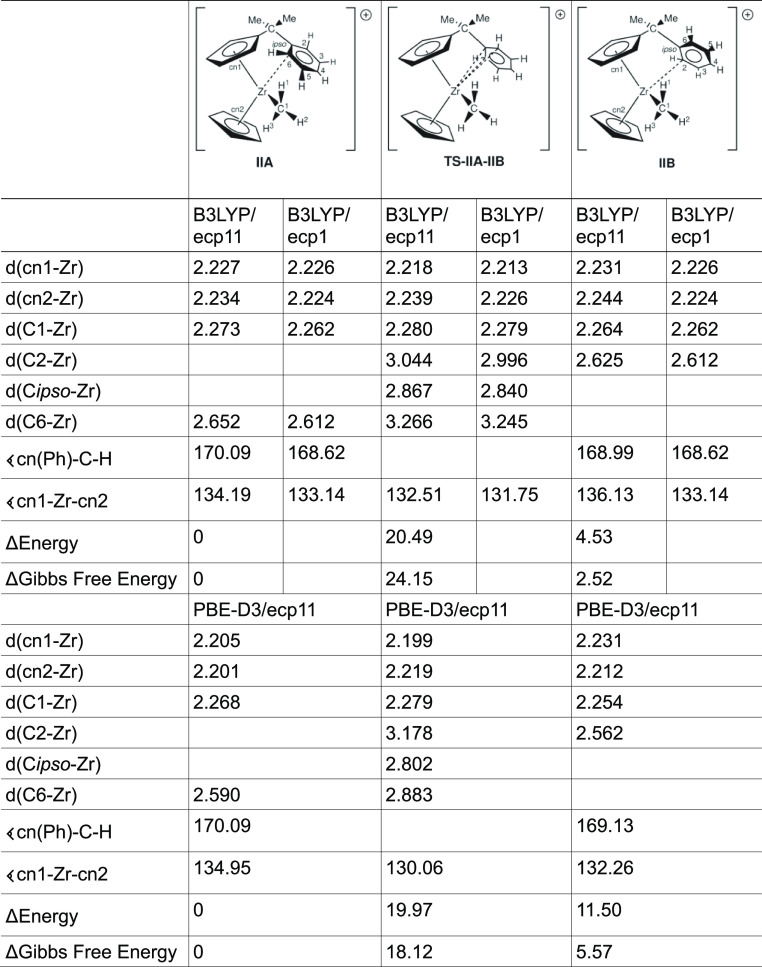
Comparison between the Computed Metrics at the B3LYP/ecp11 and PBE-D3/ecp11 Levels of Theory^a^

a The previously computed structures at the B3LYP/ecp1 level of theory were added as a reference point. Distances are in Å and angles in degrees.

Similar observations can be made of the computed energies. For the electronic energy of the activation barrier **TS-IIA-IIB**, we obtain a barrier of 20.49 kJ/mol (B3LYP, PBE-D3: 10.07 kJ/mol) with rotamer **B** being only 4.53 kJ/mol (B3LYP) higher than **A**. This value is significantly different from the PBE-D3 results (11.50 kJ/mol). For the Gibbs free energy, we found an activation barrier of 24.15 kJ/mol (B3LYP) which is higher than the 18.12 kJ/mol obtained for PBE-D3. The rotamer **B** is only 2.52 kJ/mol (B3LYP) higher in energy than **A**, compared with 5.57 kJ/mol for PBE-D3. Again, this could be attributed due to the inclusion of the dispersion forces (vide supra).

By and large, the results for the structural metrics are very similar and probably within the expected error range of the used methods. Given that the PBE-D3 functional is less computationally expensive compared with B3LYP, we believe that it is valid to use this functional for larger, similar systems instead of B3LYP. For the rest of this study, we will report the results of both functionals side by side so that a comparison can be made and this can be used as a validation for further studies.

### Structural Comparison between the Various Newly Computed Compounds [CpC_5_H_4_CMe_2_–C_6_H_4_FZrMe]^+^ (**1–5**)

Having established the validity of the employed functionals and basis sets and having established a reference point between this and previous work, we move on to report and compare the same structural parameters for the investigated compounds [CpC_5_H_4_CMe_2_–C_6_H_4_FZrMe]^+^ (**1–5**). We have systematically substituted both ortho and meta positions of the coordinated arene ring as well as the para position. This leads to 5 substitutions with each substituted isomer having 2 rotamers of the arene ring resulting in 10 ground-state compounds and 5 transition states, all of which were calculated (cf. Table 3). Starting with **1A** which has the F atom positioned on the ortho carbon C6 pointing toward the open side of the zirconocene, we then replaced the adjacent meta carbon C5 (**2A**), followed by the para carbon C4 (**5A**). As previous calculations and observed NMR data suggest that the arene ring is less likely to detach and reattach to the cationic zirconium metal, this set of data would only cover half of the possible set of isomers. Thus, we continued our replacement pattern and replaced the meta carbon C3 (**4A**) and finally the ortho carbon C2 (**3A**). This leads to a complete set of data which will give us a more finely grained look into the various bonding modes. Graphical representations of all computed molecules including their coordinates are provided in the Supporting Information.

**Table 3:**
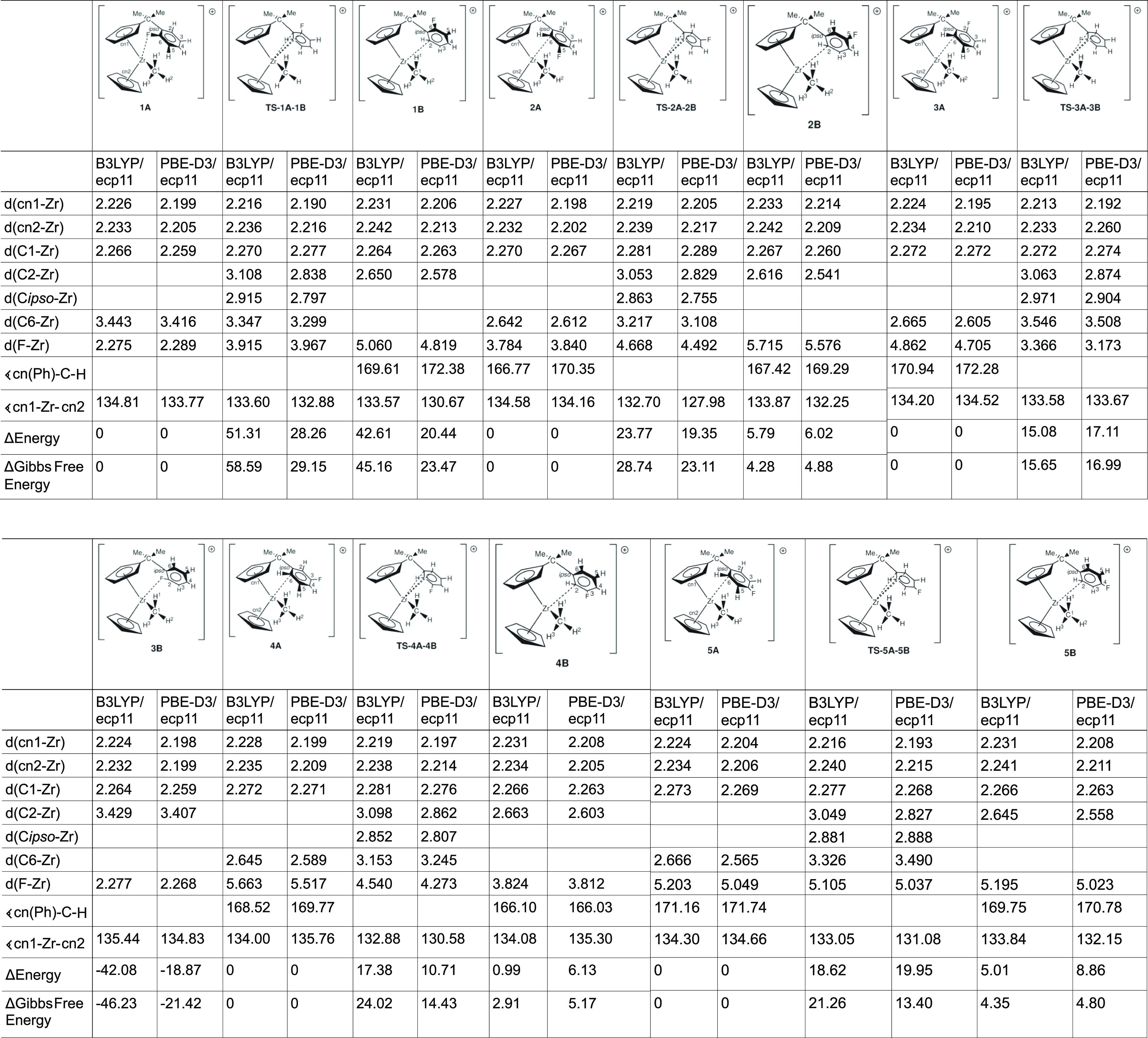
Comparison between the Computed Metrics at the B3LYP/ecp11 and PBE-D3/ecp11 Levels of Theory^a^

a Distances are in Å and angles in degrees.

For **1A**, we noticed that the ortho F atom is coordinated strongly to the Zr atom so much so that the arene ring has moved significantly compared to rotamer **1B**. Similar observations are made by the second ortho-substituted compound, but here it is rotamer **3B** with the F atom coordinated to the metal. This kind of coordination is unique to the ortho-substituted series of compounds and is not found in any of the other ones. Thus, when we report the structural metrics in Table 3, we added one line for the Zr–F distances. Furthermore, we report the electronic energies and the Gibbs free energies relative to the rotamer B to A as well in this table and for comparison in Table 2.

Inspection of Table 3 shows very clearly that the Zr–C1 distance, which is the methyl group attached to Zr, does not change much within the series of calculations and seems also indifferent to the used functional. In most cases, the difference between the two functionals is around ±0.03 Å, which is well within the computational error, and a mean value of around 2.27 Å. This is not unexpected as this bond is a rather rigid σ bond. The “softer”, that is, more flexible dative, bonds such as the bonds between the Cp rings and the zirconium as well as the coordinate arene ring have greater flexibility and thus show a wider variety in distances and angles. We note that the distance between the center of the unsubstituted Cp ring (cn2) and Zr is slightly larger than the center of the substituted Cp ring (cn1) and Zr. This is probably the result of the coordination of the arene ring, which results in the formation of an ansa metallocene-type compound.^63^ Also, the distances of the PBE-D3 functional are generally shorter compared with the B3LYP ones. A similar observation can be made from the distances between Zr and the carbons of the coordinated arene ring, that is, C2, Cipso, and C6. The bond distances obtained from PBE-D3 are generally speaking shorter compared with the ones obtained from B3LYP. This is in line with the earlier observations made with compound **II**. Similar to what has been reported before, the arene H atom of the coordinated C is bent away from the arene plane by around 8–10° with the value being slightly larger for the PBE-D3 calculations.^28^ Again, we can conclude that the observed differences are probably due to the inclusion of the dispersion forces in the PBE-D3 calculations. This leads to subtle but noticeable differences in the computed distances and angles.

### Energetic Comparison between the Various Newly Computed Compounds [CpC_5_H_4_CMe_2_–C_6_H_4_FZrMe]^+^ (**1–5**)

For the electronic energies, we observe a more striking difference between the two functionals. For the sake of this argument and to compare the results directly, we use rotamer **A** as the reference point as this is the global minimum on the potential energy surface of the unsubstituted compound **II**. However, the general trend of which compound is the more “stable” one is the same for both functionals, and the absolute numbers are remarkably different. For example, the transition-state structure **TS-1A-1B** is 51.31 kJ/mol higher in energy compared to **1A**, but for PBE-D3, we only obtain 28.26 kJ/mol. A similar drastic difference can be observed for **1B**: 42.61 kJ/mol (B3LYP) compared with 20.44 (PBE-D3). A similar observation can be made for **3B**: −42.08 versus −18.87 kJ/mol (B3LYP vs PBE-D3). All these cases have the coordination of the F atom to the cationic Zr metal in common. Chemical intuition would tell us that the Zr–F interaction should be rather strong and thus should have a shorter Zr–F distance. This is, however, not what we observe: the Zr–F distance for **1A** is 3.443 Å for B3LYP (3.416 Å for PBE-D3) and 2.277 Å (2.268 Å) for **3B**. The single bond covalent radius of F is 0.71 Å and 1.48 Å for Zr with the sum of the two being 2.19 Å.^64^ Thus, PBE-D3 computes a structure where the Zr–F distance is closer toward a Zr–F bond compared to the slightly longer distance obtained with B3LYP. However, we cannot rule out that the observed energy difference originates from the difference between a pure CGA functional (PBE) and a hybrid CGA (B3LYP) which incorporate a portion of exact exchange from Hartree–Fock theory.^65^

For the computed electronic and Gibbs free energies, we find marked differences between the different isomers and between the F-substituted and unsubstituted parent compound **II**. For example, in the transition state **TS-5A-5B**, where the F is positioned in the para position, the activation barrier is lowered to 18.62 kJ/mol (PBE-D3: 19.95 kJ/mol) with a Gibbs free energy of 21.26 kJ/mol (PBE-D3: 13.40 kJ/mol). The stability of the rotamer **5B** is slightly higher at 5.01 kJ/mol (B3LYP) but lower than that at 8.86 kJ/mol for PBE-D3. The Gibbs free energy results are quite similar: 4.35 kJ/mol (B3LYP, higher compared with **IIB**) and 4.80 kJ/mol (PBE-D3, lower compared with **IIB**). This lowering of the transition barrier can be attributed to the electron-withdrawing (−I) effect of F, which lowers the electron density on the ortho carbon and thus renders the Zr–C(H) interaction weaker (cf. Table 4). This hypothesis will be discussed later.

**Table 4:**
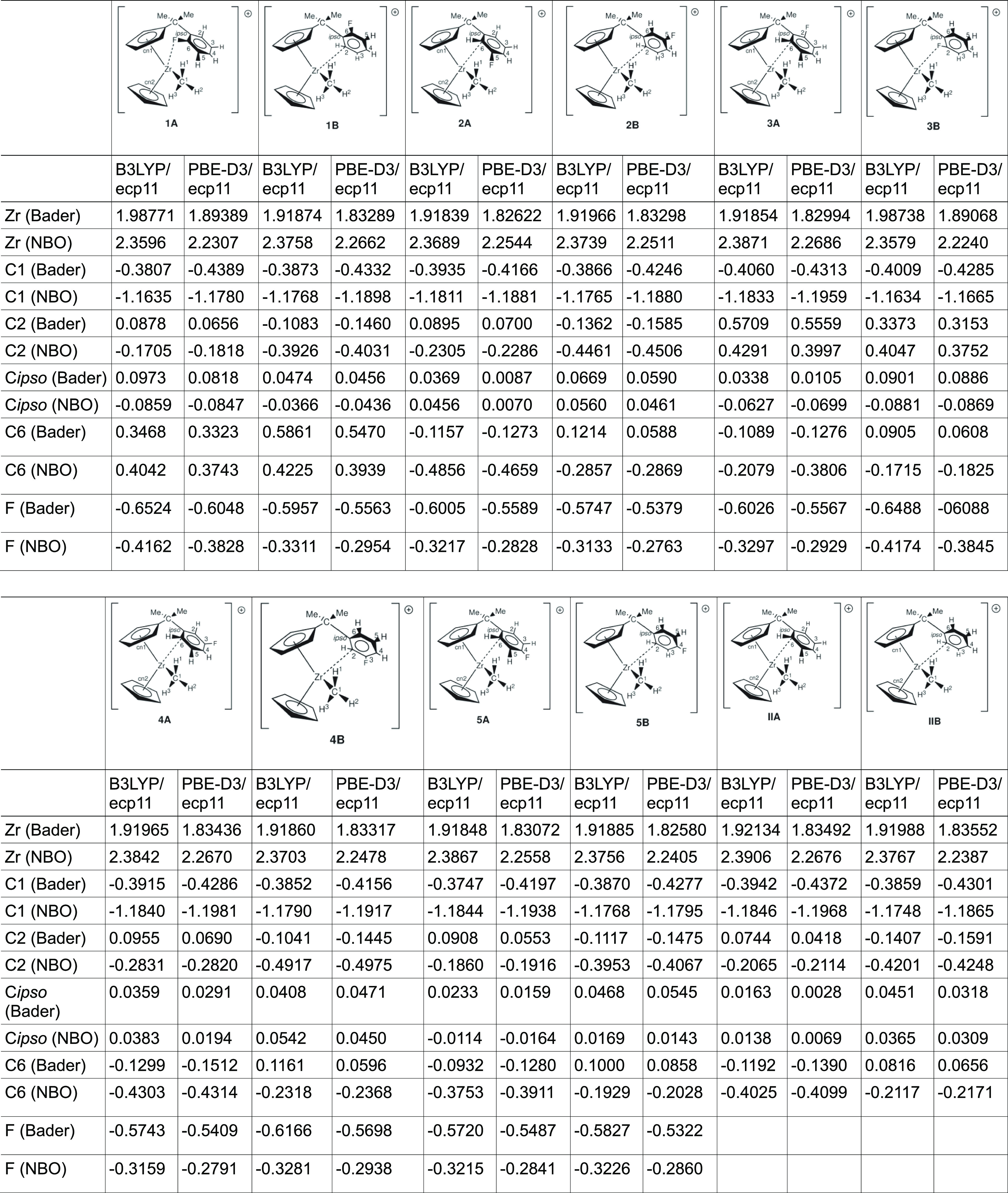
Selected Bader and NBO Charges of All Reported Molecules

For the remaining four isomers, things are a bit more complicated as here the position of F plays a crucial role. For example, for the two ortho-substituted compounds **1** and **3**, the electronic activation energy of **TS-1A-1B** is significantly higher with 51.31 kJ/mol (PBE-D3: 28.26 kJ/mol) with rotamer **1B** being 42.61 kJ/mol (PBE-D3: 20.44 kJ/mol) significantly higher in energy than **1A**. For isomer **3**, we find a similar trend: the Zr–F rotamer **3B** is −42.08 kJ/mol (PBE-D3: −18.87 kJ/mol) **lower** in energy than the noncoordinated rotamer **3A**. This result is what would be expected: a Zr–F interaction is stronger than a Zr–C(H) one, and this is clearly visible by the close Zr–F distances in **1A** and **3B**.

The meta-substituted isomers are a little bit in-between the para and ortho ones. Here, the F atom is sufficiently far enough from the Zr to prevent coordination but still close enough to have an electronic influence (+M effect). Thus, the transition barrier **TS-2A-2B** is only 23.77 kJ/mol (PBE-D3: 19.35 kJ/mol) and for **TS4A-4B** 17.38 kJ/mol (PBE-D3: 10.71 kJ/mol), which is somewhat closer to the ones observed for **TS-5A-5B** and **TS-IIA-IIB**. A more significant difference can be observed for **2B**/**4B**: whereas the energy of **2B** is 5.79 kJ/mol (PBE-D3: 6.02 kJ/mol), for **4B**, we obtain 0.99 kJ/mol for B3LYP but 6.13 kJ/mol for PBE-D3. This example quite nicely demonstrates the influence the inclusion of the dispersion forces into the functional has: including the dispersion force the Zr–F interaction of **4B** that is well captured in the PBE-D3 functional but completely ignored in B3LYP.

### Electronic Comparison between the Various Newly Computed Compounds [CpC_5_H_4_CMe_2_–C_6_H_4_FZrMe]^+^ (**1–5**)

In order to get a deeper understanding of the observed interactions of the arene ring with the cationic Zr metal, we take a closer look at the electronic properties of each calculated molecule. We first look into the charges of some selected atoms and move on to describe the resulting interaction. In this way, we hope to build up a detailed and thorough picture of the electronic nature of these molecules.

### Atomic Charges

As there are several ways of describing the charge of an atom in a molecule and all of them have their own issues, we focus here on QTAIM and NBO charges. We only report the charges of the atoms which we have described in Tables 2 and 3 with the results summarized in Table 4. Note that we only report the charges of the ground-state structures and not those of the transition-state ones.

For the unsubstituted **IIA**, we obtain a QTAIM charge of 0.0744 (NBO: −0.2065) for the noncoordinated carbon C2. Upon coordination with the Zr metal in **IIB**, this carbon becomes more negative, and consequently, the charge changes to −0.1407 (NBO: −0.4201). A similar observation can be made for the second ortho carbon C6: in **IIA**, the charge is −0.1192 (NBO: −0.4026), which changes to 0.0816 (NBO: −0.2117) in **IIB**. Thus, we notice that upon coordination with the cationic Zr, the charge of the coordinated ortho carbon becomes more negative. A similar effect can be observed for the para-substituted compound **5**: the charge of C2 changes from 0.0908 (NBO: −0.1860) in **5A** to −0.1117 (NBO: −0.3953) in **5B**. Similarly, the charge of C6 changes from −0.0932 (NBO: −0.3753) in **5A** to 0.1000 (NBO: −0.1929) in **5B**.

However, whereas the change for C2 for the different rotamers in **II** is 0.2151 (NBO: 0.2136), for **5**, we found a lower value of only 0.2025 (NBO: 0.2093).

Looking at the third carbon which is close to the Zr metal, the ipso carbon of the arene ring, we observe the following: in **II**, the charge changes from 0.0163 (NBO: 0.0138) in **IIA** to 0.0451 (NBO: 0.0365) in **IIB**, that is, a change of −0.0288 (NBO: −0.0227).

As this atom is the furthest away in the para position relative to the substituted carbon C4, we would not expect much of an influence of the F atom here. Indeed, the charges of the ipso C change from 0.0233 (NBO: −0.0114) in **5A** to 0.0468 (NBO: 0.0169) in **5B**, which is a change of −0.0235 (NBO: −0.0283), again lower for the para-substituted compound.

In order to place these numbers into some context, we move on to the meta-substituted compound **4**. For C2 in **4A**, we found a charge of 0.0955 (NBO: −0.2831) and −0.1041 (NBO: −0.4917) in **4B**, that is, a significant change of 0.1996 (NBO: 0.2086). This clearly indicates the influence of the F atom. In **4A**, the F atom is in the para position relative to the coordinated carbon C6. However, in **4B** it is in the ortho position relative to the coordinated C2 and thus much closer to the coordinated carbon atom. This close proximity renders the coordinated C2 *less* negative compared to a meta position or the unsubstituted compound (−0.1041 in **4B** vs −0.1407 in **IIB** or −0.1117 in **5B**) which can be attributed to a +M effect.

To see whether this is a mere steric effect, we compare this with **2** where F is in the meta position as well but points toward the more open side of the metallocene wedge. Again, only looking at C2, we found a charge of 0.0895 (NBO: −0.2305) for **2A** and −0.1362 (NBO: −0.4461) for **2B**. The value of **2B** is much closer to the value observed in **IIB** compared with **4B**. It is tempting to speculate that the shorter Zr–F distance in **4B** (3.824 Å) compared with 5.576 Å in **2B** could lead to some distortion of the electron distribution in the arene ring, resulting in the observed numbers. Thus, we could see this as some sterically induced influence.

The situation is a bit more difficult for the ortho-substituted compounds **1A** and **3B** as the F atom coordinates with the Zr atom when in close proximity. Thus, simply reporting the charges of C2, Cipso, and C6 would not make any sense. To overcome this obstacle, we used the computed structures of **5** and replaced the H atom on C2 and C6 with F, respectively, and adjusted the C–F bond length accordingly. The structures thus obtained, **6A** and **6B**, were subject to a single-point QTAIM and NBO analysis at both B3LYP and PBE-D3 levels of theory. In this way, we hope to get some more detailed insight into the influence of the F atom on the coordination of the arene ring. The results of this investigation are recorded in Table S1.

For structure **6A**, which would be somewhat equivalent to **1A**, we obtain a charge for C2 of 0.0647 (PBE-D3: 0.0720) with a NBO charge of −0.1864 (PBE-D3: −0.1945).

For **6B**, the **3B** equivalent, we obtain for C2 0.3544 (PBE-D3: 0.3519) with a NBO charge of 0.2484 (PBE-D3: 0.2306).

The overall electronic picture we obtain could be summarized as follows: in the case of the unsubstituted arene ring, the positively charged Zr metal induces an electrical field which disturbs the π electron cloud of the arene ring in such a way that there is a charge accumulation at the carbon atom which is in close proximity to the Zr, that is, either C2 or C6. Thus, upon coordination with the Zr atom, these carbon atoms become more electron-rich and thus more negative. The remaining carbon electron density will be depleted, and thus, they become more positive. The introduction of a F atom in the ring changes this significantly. The strong −I effect of F pulls the π electron cloud toward the **C**–F carbon which will be more negative as a consequence. However, due to the +M effect of F, the ortho and para positions relative to the F atom will experience a more profound effect. On top of this effect is the already discussed effect of the cationic Zr which introduces an external electric field which is the strongest at the carbon which is in close proximity to the Zr. There is fortunately one way of looking into this, which is ^13^C NMR, where the observed chemical shifts strongly depend on the electron cloud around the observed nucleus. Thus, if we were to synthesize these compounds, we would have a handle for investigating the above conclusion and could find some support for it. We report the calculated chemical shifts below, once we convinced ourselves further which orbitals are involved in the proposed interactions and also about the nature of the interaction.

### QTAIM Bond Paths and NBO Orbitals

Having established the charges of the individual atoms of interest, we now moving on to the kind of interaction they show if any. Again, we utilize QTAIM and NBO theories here, with the former giving us an indication of whether or not a “bond”, as defined within the QTAIM framework, exists and the latter giving us an indication of which orbitals might be involved in such bonding. The electron density, ρ(**r**), the Laplacian, ∇ρ(**r**), and the Virial field function, VIR, at the bond critical point of the bond path connecting Zr–C2 and Zr–C6 as bond critical point 1 (bcp1), C2/C6 and H/F as bcp2, and Zr–C1 as bcp3, respectively, are reported in Table 5 for both the B3LYP and PBE-D3 calculations. We only report the values of those atoms which interact with Zr rather than reporting all possible values.

**Table 5:**
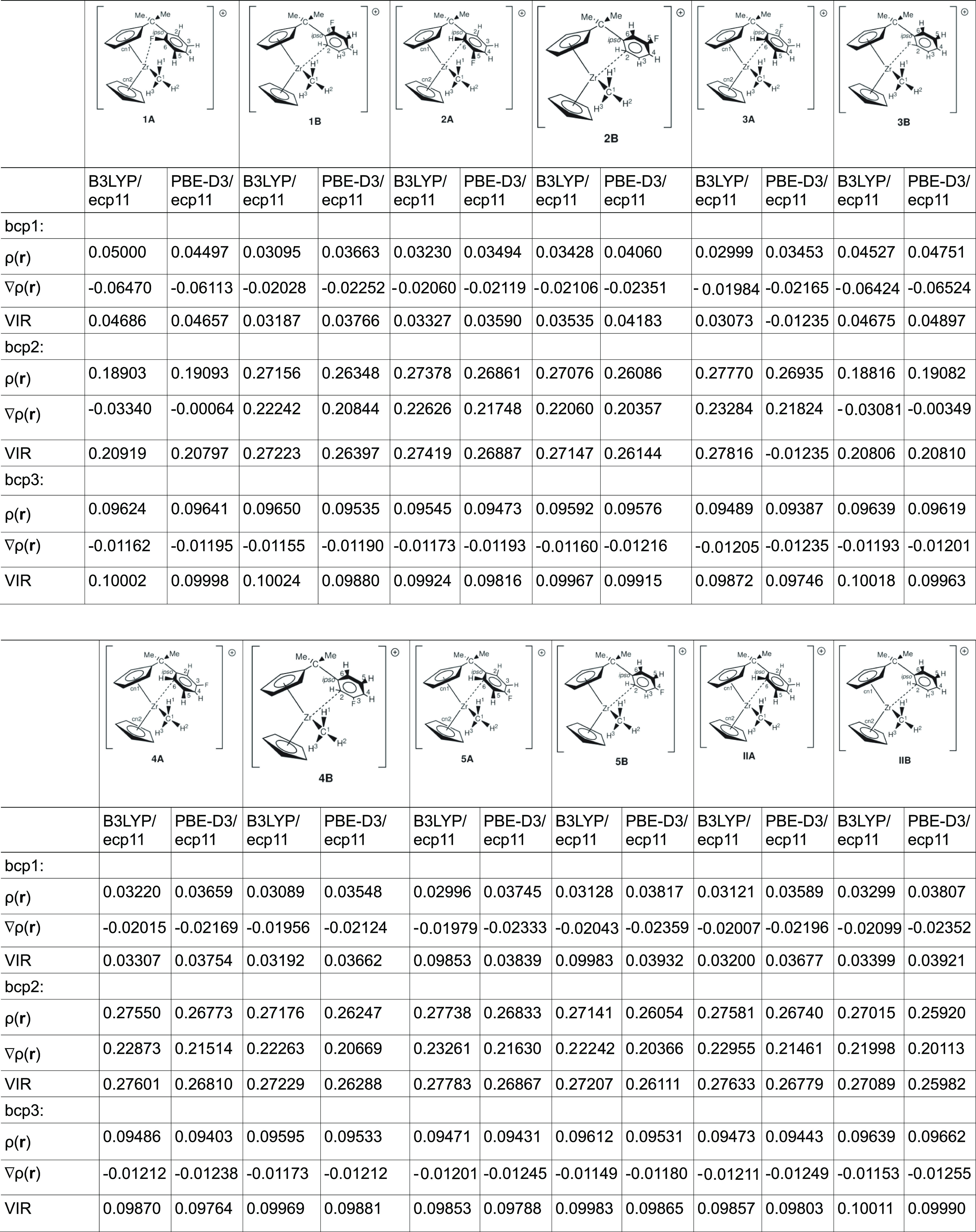
QTAIM Analysis of Relevant Zr–C (Zr–F) and C–H (C–F) Bond Parameters for Both the B3LYP and PBE-D3 Functionals

Inspection of Table 5 reveals the following trends:

- for bcp3, which is the Zr–Me bond, we observe only small changes in the electron density ρ(**r**), which ranges between 0.09471 and 0.09650 au with an average of 0.09570 au and a range of 0.00179 au (B3LYP). In general, the values for rotamer “A” are slightly smaller than those for rotamer “B”, which probably can be attributed to the different electron donation properties of C2 compared with C6.
- for bcp1, which is the Zr–C2 (Zr–C6) interaction, we observe values ranging between 0.02996 and 0.03663 au with an average of 0.02012 au and a range of 0.00667 au (B3LYP). Again, we observe a difference between the values for rotamer “A” and “B”, but this time, it seems to be less straightforward.
- for bpc1, for the Zr–F interaction, we observe a value of 0.0500 and 0.04527 au, which is significantly higher than for the Zr–C2 and Zr–C6 interactions, respectively. This is somewhat expected, given that Zr–F bonds are expected to be stronger; there is no reason why this argument does not hold in this particular case here.
- for bcp2, for the C2–H and C6–H bond, respectively, the changes lie between 0.26348 and 0.27770 au with an average value of 0.27373 au and a range of 0.01422 au (B3LYP). This is the largest change we observe. Again, we observe a difference between the two rotamers, and again, there is no straightforward trend.
- for bpc2, for the C–F bond, we observe a value of 0.18903 and 0.18816 au (B3LYP).

The values for the PBE-D3 calculations follow a similar trend, so we are not reporting them here again.

A closer inspection of the values of the bcp3 and comparison with the unsubstituted compounds **IIA** and **IIB** reveals the following:

- in case of the F atom being in the meta position of the coordinated carbon C2 (**1B**) (C6: **3A**), both values for the A and B rotamer are virtually identical in the case of the B3LYP functional. For the PBE-D3 functional, the substituted compounds appear to have a slightly lower electron density at the bond critical point. This appears to be more prominent in the B rotamer than in the A one
- for the ortho position, the A rotamer, which is the more stable one, has a slightly higher electron density at the bond critical point compared with the unsubstituted compound (**2A**: 0.09545 vs 0.09473 for **IIA**). The same can be observed for the results obtained with the PBE-D3 functional
- for the ortho position, the B rotamer, which is the less stable one, has a slightly less electron density at the bond critical point (**4B**: 0.09595 vs 0.09636 for **IIB**)
- for the para position, a similar trend can be observed.

In general, the effects on the bcp3, that is, the Zr–Me bond, seem to be quite subtle. Placing the F atom in the ortho or para position relative to the coordinated C atom and only considering rotamer A leads to a slight “strengthening” of the Zr–Me bond. However, as rotamer A will interconvert into rotamer B in practice, this effect will be canceled out. This is of some significance as the Me group could be seen as a “model” for the growing polymer chain. This means that if there were large changes in the electron density of this particular bond critical point, we could assume that the coordinated arene ring would promote or stall β-H elimination. This is not the case here as the electron density of this particular bond critical point does not fluctuate much depending on the coordination mode of the arene ring.

Rather than reporting all the relevant NBO orbitals here, we only report one example here and report the NBO orbitals of relevant interactions in the Supporting Information, which also contains the plots of the electron density, Laplacian, and Virial field function. Nonetheless, some relevant parameters such as the Wiberg bond index (WBI) and the natural binding index (NBI) for the Zr–C1, C2–H, and C6–H bonds and the Zr–C2/Zr–C6 or the Zr–F interactions are given in Table 6. These parameters are easier to compare with each other than the second-order perturbation energy which depends on the involved orbitals. In general, there are two types of significant energy, that is, over 2 kcal/mol, interactions observed:

- the weaker interaction of the C–H bond with an empty Zr orbital.
- the more dominant interaction of the C–C double bond with an empty Zr orbital.
- the less dominant interaction of the other C–C double bond with an empty Zr orbital.

**Table 6:**
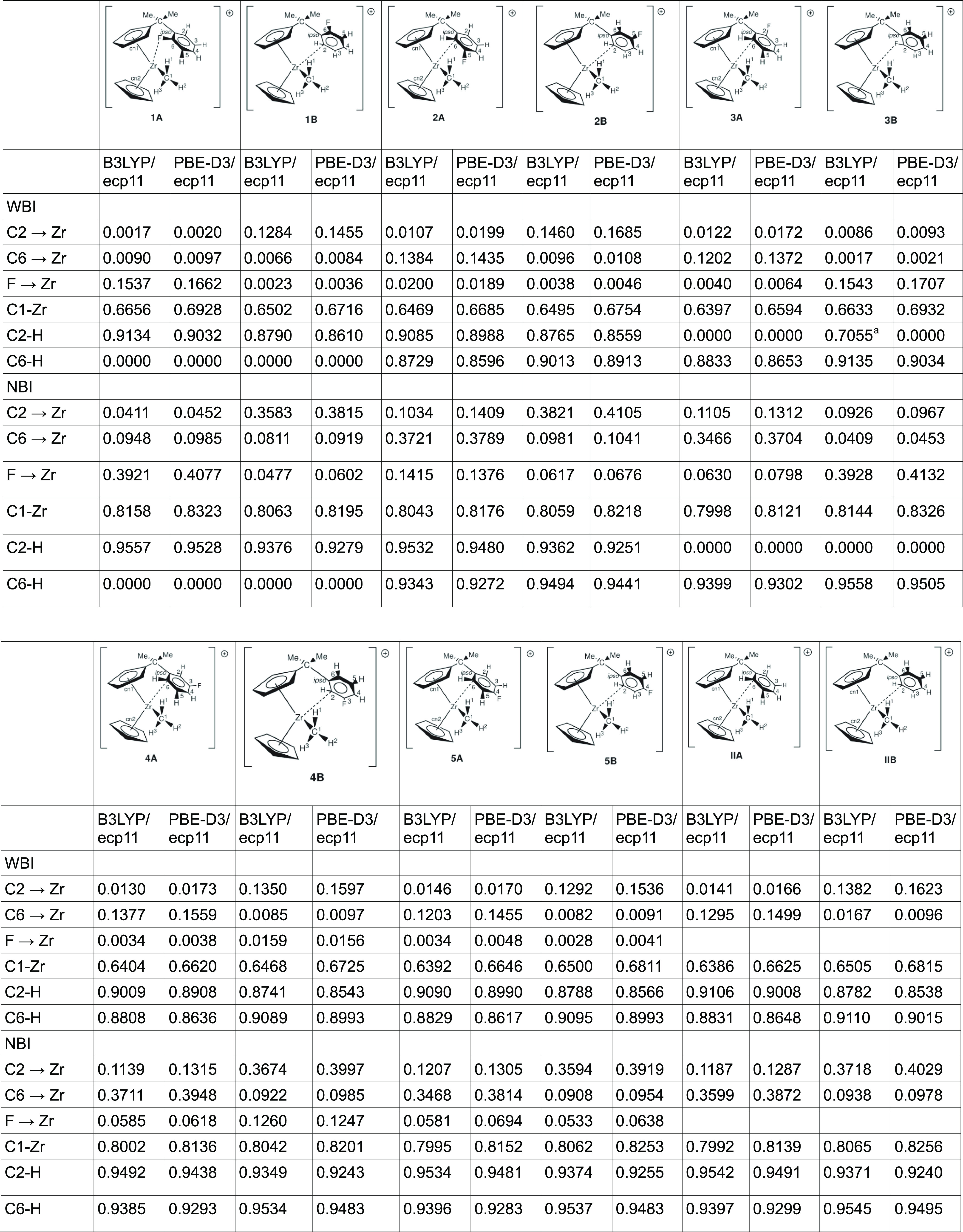
Selected WBIs

a C6–F or C2–F, respectively.

As we have previously noted,^30^ the observed interaction of the arene ring with the cationic Zr metal is not an *agostic* one but rather an interaction of the electron cloud of the ring with the metal. The Zr–H bond only contributes to this interaction but is not the dominant part of it. This observation is basically unaltered by the substitution of a H with F on the arene ring (cf. the Supporting Information for more details).

A close observation of the WBI for the Zr–Me bond reveals a similar trend as we observed for the Bader analysis above. The WBI also confirms the observation that the C–H bond participates in the interaction of the arene ring with the Zr metal as the WBI is lowered upon coordination of C2 (C6) to the metal. For example for the unsubstituted compound **IIA** the WBI of the uncoordinated C2–H is 0.9106 and for the coordinated C6–H 0.88310 with the B3LYP functional. The rotamer **IIB** shows similar values but the opposite trend: here, C2–H (coordinated) has a value of 0.8782 and C6–H (not coordinated) has 0.9110. Thus, the WBI for the C–H bond of the coordinated C is around 0.88, which is lower than the value of the noncoordinated C of around 0.91. This again indicates the lowering of the bond order due to the participation of the C–H bond in the arene–Zr interaction.

### Noncovalent Interactions

**Figure 1:**
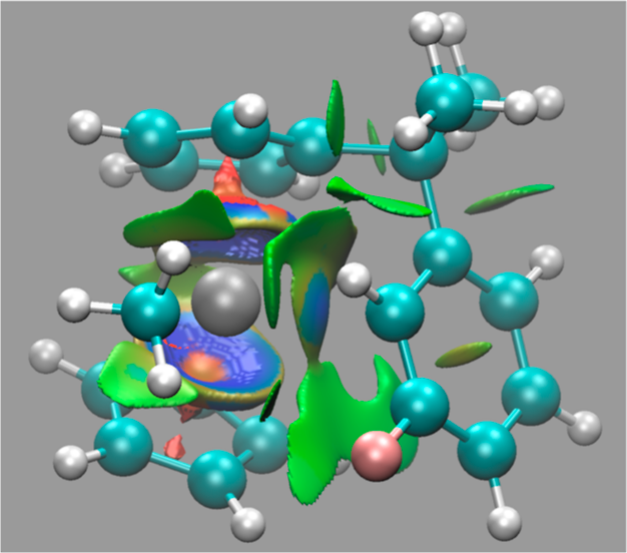
NCI plot of 2A.

Further evidence of the above observations was found with the noncovalent interaction (NCI) analysis. We have computed this for all compounds and all used functionals, and all pictures have been summarized in the Supporting Information. For demonstration purposes, we have included one picture of **2A** which has the F atom adjacent to the coordinated C6 atom (Figure 1). The color coding is as follows: a strong attractive interaction is denoted with a blue color, whereas a repulsive one is denoted with a red color. Thus, the attractive interaction of the Cp rings with the Zr is colored in blue. Weaker attractive interactions are colored in green. Bearing this in mind, one clearly can see the interaction of the C6 atom with the Zr metal (gray color in the picture) as it has a blue surface. We also note the green surface of the C–*H*. This interaction is **not** with the Zr metal but rather with the C–*H* of the Zr–*Me* group.^59^ More interesting is the interaction of the F atom and the adjacent C with the C–*H* of the Cp ring. We also can just about observe the C–C bond interaction with the Zr metal. In summary, the NCI analysis confirms the interactions between the arene ring and the cationic Zr metal.

### Chemical Shift Calculations (NMR)

The calculation of the chemical shifts, using the gauge-including atomic orbitals (GIAO)-DFT method allows us to compare experimental results with computationally obtained ones. As the chemical shift of a nucleus strongly depends on its surrounding electron cloud, we again are able to look from an experimental and theoretical point of view. Thus, we can use different tools to look into the electronic properties of an atom in a molecule. As before,^28^ we are more interested in the accurate calculation of the properties of the arene ring and thus choose benzene as a reference point for these calculations. Furthermore, we have calculated para-^*t*^butyl-fluoro benzene as a surrogate compound for the arene ring in the zirconocenes. The results are summarized in Table 7.

**Table 7:**
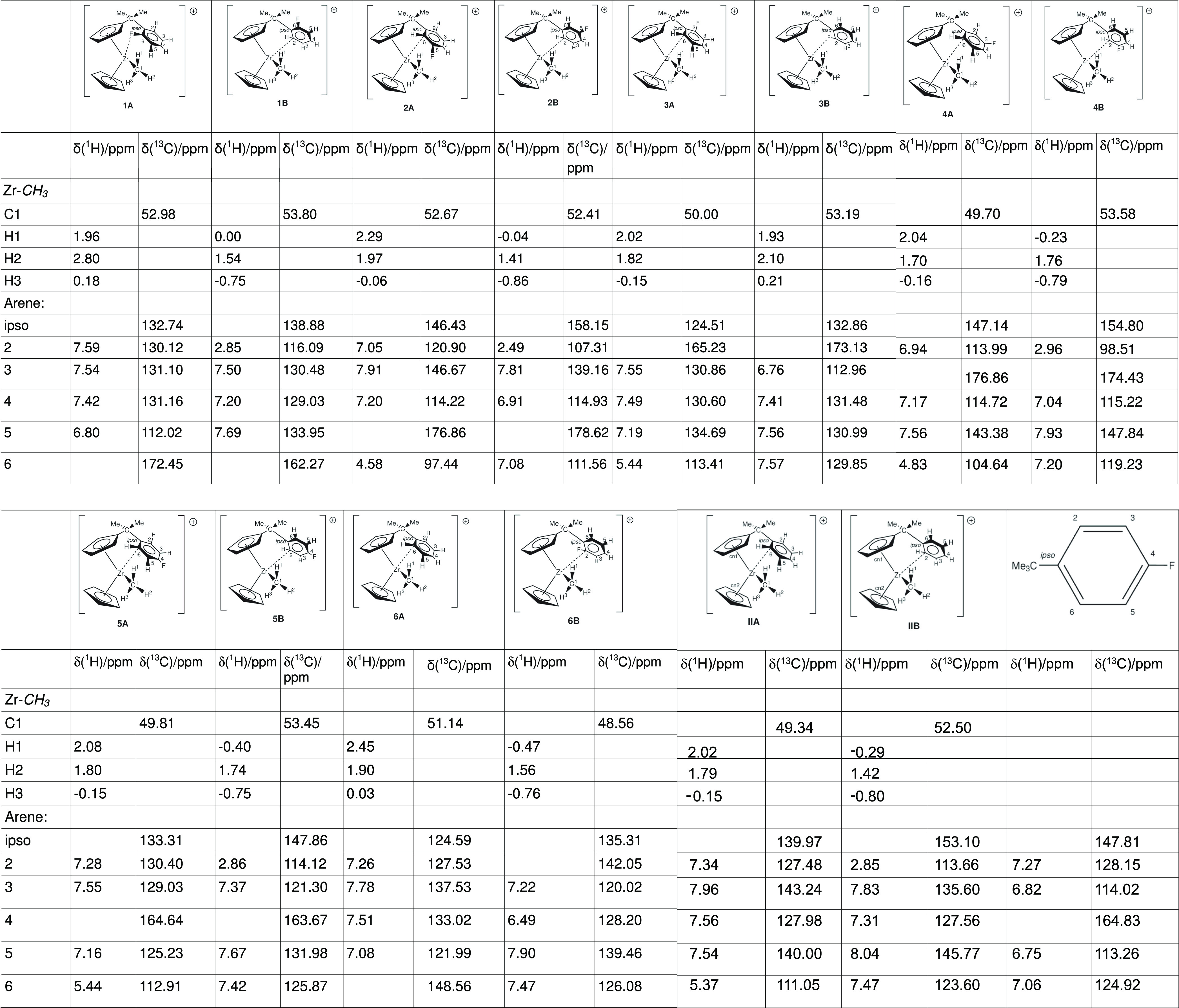
Calculation Chemical Shifts at the B3LYP/Basis II Level of Theory

In general, some observations can be made. For the chemical shift of the Zr–*C*H_3_, for the compounds where C6 is coordinated and the F group is on the opposite side of the ring, that is **4A**, **5A**, and **IIA**, the value is around 49 ppm with only very small differences in the region of a 1/10 of a ppm. Given the uncertainty of the calculations, we could conclude that they are virtually identical. For the remaining four compounds **1A**, **2A**, **3A**, and **6A**, where F is either at or close to the coordinated carbon, the values have a larger spread between 50.00 ppm (**3A**) and 52.98 ppm (**1A**). This warrants some explanation. For **1A**, the F atom is actually coordinated to the Zr, and the −I effect of the F and the positively charged Zr deplete the electron cloud of C1 significantly, and thus, we observed the expected low-field shift of the C atom. For **3A**, the F atom is in the meta position and thus has little effect on the shielding of C1 when compared to the chemical shift of C6 (111.56 ppm) to the unsubstituted **IIA** (111.05 ppm). Consequently, the chemical shifts of C1 in **3A** (50.00 ppm) are very similar to the ones in **IIA** (49.34 ppm). Similar observations can be made for the coordinated C6: 113.41 ppm (**3A**) versus 111.05 ppm (**IIA**). For **2A** (52.67 ppm), the F is in the ortho position of C6; thus, the −I effect is dominating here, leading to a high field shift of C6 (97.44 ppm vs 113.41 ppm for **3A**). From what was said before, we would expect the Zr atom to be less positive and thus C1 to be more shielded compared to **3A**. However, this is not the case. Given that **4A**, **5A**, and **IIA** have nearly identical shifts, although the shifts for C6 vary between 104.64 ppm (**4A**) and 122.91 ppm (**5A**), clearly, there is more to it than the chemical shifts of C6. One possible explanation could be the spatial arrangement of the arene ring and/or an inductive effect of the F atom on C1 in (F–C1 distance 3.82 Å) and thus compensate for the more shielded C6, compared with **3A**. For **6A** (51.14 ppm), the situation seems to be more straightforward. As F is bonded to C6, it renders C6 less shielded, that is, more positive (148.56 ppm), which is somewhat closer to the value for **1A** (172.45 ppm). Thus, we can expect a similar shift for C1, bearing in mind the different orientations of the arene ring for these two compounds.

For the rotamer **B**, we observe a slightly narrower chemical shift between 53.80 ppm (**1B**) and 52.41 ppm (**2B**) with the notable exception of **6B** of 48.56 ppm. Ordering the chemical shifts of the coordinated C2 from most shielded to less, we obtain the following order: **4B** < **2B** < **IIB** < **5B** < **1B** < **6B** < **3B**. However, for the chemical shifts of C1, we obtain a different order: **6B** < **2B** < **IIB** < **5B** < **4B** < **3B** < **1B**. This again demonstrates that a simple answer, like only looking at the chemical shifts of the coordinated carbon atom, is insufficient.

## Discussion

The computed data sets result in a very complex picture. To gain a better understanding, we summarize the relevant parameters of the Zr–CH_3_ moiety in Table 8.

**Table 8:** Summary of Relevant Zr–CH_3_ Parameters with the PBE Calculations in Brackets

	*d*(Zr–C1)	charge (Bader)	charge (NBO)	bcp3 ρ(r)	WBI	NBI	δ (^13^C)/ppm
**1A**	2.266 (2.259)	–0.3807 (−0.4389)	–1.1635 (−1.1780)	0.09624 (0.09641)	0.6656 (0.6928)	0.8158 (0.8323)	52.98
**1B**	2.264 (2.263)	–0.3873 (−0.4332)	–1.1768 (−1.1898)	0.09650 (0.09535)	0.6502 (0.6716)	0.8063 (0.8195)	53.80
**2A**	2.270 (2.267)	–0.3935 (−0.4166)	–1.1811 (−1.1881)	0.09545 (0.09473)	0.6469 (0.6685)	0.8043 (0.8176)	52.67
**2B**	2.267 (2.260)	–0.3866 (−0.4246)	–1.1765 (−1.1880)	0.09592 (0.09576)	0.6495 (0.6754)	0.8059 (0.8218)	52.41
**3A**	2.272 (2.272)	–0.4060 (−0.4313)	–1.1833 (−1.1959)	0.09489 (0.09387)	0.6397 (0.6594)	0.7998 (0.8121)	50.00
**3B**	2.264 (2.259)	–0.4009 (−0.4285)	–1.1634 (−1.1665)	0.09639 (0.09619)	0.6633 (0.6932)	0.8144 (0.8326)	53.19
**4A**	2.272 (2.271)	–0.3915 (−0.4286)	–1.1840 (−1.1981)	0.09486 (0.09403)	0.6404 (0.6620)	0.8002 (0.8136)	49.70
**4B**	2.266 (2.263)	–0.3852 (−0.4156)	–1.1790 (−1.1917)	0.09595 (0.09533)	0.6468 (0.6725)	0.8042 (0.8201)	53.58
**5A**	2.273 (2.269)	–0.3747 (−0.4197)	–1.1844 (−1.1938)	0.09471 (0.09431)	0.6392 (0.6646)	0.7995 (0.8152)	49.81
**5B**	2.266 (2.263)	–0.3870 (−0.4277)	–1.1768 (−1.1795)	0.09612 (0.09531)	0.6500 (0.6811)	0.8062 (0.8253)	53.45
**6A**	2.273 (2.273)	–0.3781 (−0.4086)	–1.16931–1.18742	0.09887 (0.09436)	0.6474 (0.6720)	0.8046 (0.8197)	51.14
**6B**	2.267 (2.266)	–0.3890 (−0.4205)	–1.18690–1.20497	0.09622 (0.09550)	0.6344 (0.6604)	0.7965 (0.8127)	48.56
**IIA**	2.273	–0.3942 (−0.4372)	–1.1846 (−1.1968)	0.09473 (0.09443)	0.6386 (0.6625)	0.7992 (0.8139)	49.34
**IIB**	2.264	–0.3859 (−0.4301)	–1.1748 (−1.1865)	0.09639 (0.09662)	0.6505 (0.6815)	0.8065 (0.8256)	52.50

The first notable observation is that the natural charge varies little, compared to the Bader charges (cf. the Supporting InformationS1). Thus, in the following, we will concentrate on the Bader charges. The next general observation is that the Zr–CH_3_ distance is generally larger for the “A” series of compounds and shorter for the “B” one (cf. Supporting Information S2). This could be simply a steric effect as for the “B” series, the arene ring is “parallel” to the Zr–CH_3_ moiety, whereas for the “A” series, the ortho H of the arene ring is in close proximity to the Zr–CH_3_ moiety. This hypothesis is corroborated by the fact that **1A**, where the arene ring has a similar orientation as in the “B” series, has a Zr–CH_3_ distance which falls within the range of the “B” series of compounds. We note that the WBIs and the NBIs follow the same pattern when plotted against the Zr–CH_3_ distance (cf. S3 and S4). For compounds **2**–**5** and **II**, the “A” series has a lower number compared with the “B” series. This would indicate a “weaker” Zr–CH_3_ bonding for the “A” series and thus should lead to a larger Zr–CH_3_ bond distance and concomitant to a lower number for the bond critical point. This is indeed what is observed. For these compounds, similar observations can be made for the calculated carbon shifts, with the notable exception of **2**, where both **2A** and **2B** have very similar chemical shifts. This could be attributed to the ortho position of the F atom to C6 and thus influences the electron cloud around C1. The exceptions of these observations are compounds **1** and **6**. Here, F is in the ortho position and thus coordinates with the cationic Zr metal in the case of **1A**. This results in a completely different arrangement of the arene ring, and thus, the results cannot be compared directly. For example, whereas for **1A**, both the values of the WBI and the NBI are *higher* compared with the relative values of, for example, the reference compound **IIA**; when compared with their respective rotamer **B**, the bond critical point (cf. Supporting Information S5) and the ^13^C chemical shift (cf. Supporting Information S6) are *lower* compared to **1B**. From the *higher* WBI and the NBI values, we would expect a stronger and thus shorter bond, as we can observe from compounds **2**–**5**. However, this is not the case: the Zr–CH_3_ distance is *larger* compared to what would be expected. The opposite is true for **1B**. Here, the *lower* WBI and the NBI values should lead to a *longer* bond distance compared with **1A** when in fact the bond distance is *shorter*.

For the two hypothetical compounds **6A** and **6B**, the situation is even more puzzling. From the high bond critical point value of **6A**, the highest of all investigated compounds, we would expect the shortest bond distance. However, this is not the case, and it cannot be the case as this calculated structure was only a single-point calculation, that is, no geometry optimization took place. Still, if the observed electronic features are purely based on the geometry, we would expect a significantly lower bond critical point value for **6A**, compared with the value of say **5A** which has a similar bond distance. Graphs S1–S6 of the various discussed relationships between bond distances are available in the Supporting Information.

## Conclusions

We performed a detailed computational study of cationic zirconocene compounds with a substituted arene ring tethered to one of the Cp rings. We used this model compound not only to obtain a detailed insight into the coordination of the arene ring to the cationic Zr metal but also to compare two different computational methods: the use of the well-established functional B3LYP, which we have used in previous studies, and the use of the more modern, that is, recently implemented, DFT-D method. In particular, the latter is of interest as it not only allows us to take care of the dispersion interactions where normal DFT methods are notoriously bad at it, but it also should allow us to compute larger, more realistic systems with reasonable computational costs. The selection of the PBE functional together with Grimme’s DFT-D version 3 method was thus selected bearing this in mind.

Overall, the PBE-D3 results are quite similar to the ones obtained with the B3LYP functional. The metric parameters are comparable. Broadly speaking, the same goes for the rather exhaustive study of electronic parameters (QTAIM, NBO, NCI, and NMR). Our results again confirm that the coordination of the arene ring is not an *agostic* bond but rather the previously observed interaction of the C–C double bond with the cationic Zr metal. The C–H bond only contributes to this coordination. We noticed an influence of the F atom which depends on its position in the ring. The most influence was noticed in either ortho or para to the coordinated carbon atom. However, as we also noticed strong coordination of the F atom to the Zr metal in the case of F being attached to either C2 or C6, these two isomers are probably not realistic models for olefin polymerization as they probably effectively shut down the catalyst.

As similar trends were observed in all investigated cases, this gives us the confidence to use the DFT-D method for our compounds of interest. As the PBE-D3 method is computationally cheap, it also will allow us to get larger, more realistic systems and perform molecular dynamic studies on them. As we are not after exact numbers but more after trends, we can recommend the use of PBE-D3 as an alternative to B3LYP without sacrificing too much accuracy in this particular case. This is in line with previously published results. Furthermore, given the advance of machine learning, with the wealth of information provided (QTAIM, NBO, NCI, and NMR results next to steric parameters), it would be interesting to see if machine learning could cut through the cobwebs of various interactions and deliver us a clearer understanding of which parameters need to be changed to achieve a particular result, like an even more tailor-made catalyst.

As it is known that a F atom in close proximity to the metal center can further enhance the rate of the chain propagation,^66,67^ it is interesting to take that aspect into consideration as well. We are currently working on such a computational investigation, and the results will be published in due time.

Finally, with artificial intelligence (AI) and machine learning (ML) becoming more and more prominent, these kinds of comparative studies which are presented here are becoming more and more important as they serve as a training set for these processes.^68^ In this way, as we provide a very detailed training set which also includes electronic properties, next to the usually used steric ones, we hope that a better-refined training of the AI or ML can be achieved and thus better predictions with less computational expensive methods can be made.

### Computational Details

All calculations were performed on Debian Linux (Jessie). The B3LYP calculations were performed using Gaussian G09, version D01.^69^ The PBE-D3 calculations were performed using GAMESS 2014 R1.^70,71^ In all cases, a mixed basis set consisting out of Pople’s 6-311G(d,p) basis set was used for all elements but Zr, where the Stuttgart–Dresden effective core basis set was used. This mixed basis set is abbreviated as ecp11 and is basically an expansion of the previously used ecp1 basis set.^28^

Magnetic shielding σ has been evaluated for the B3LYP geometries using the implementation of the GIAO-DFT method,^72^ involving the B3LYP functional with a [12s9p5d] all-electron basis for Zr, contracted from the (17s13p9d) set^73^ and including two diffuse p and one diffuse d function^74^ (exponents 0.11323, 0.04108, and 0.0382), and the recommended IGLO-basis II^75^ on C and H. This particular combination of functionals and basis sets has proven quite effective for chemical shift computations for transition-metal complexes.^28^^1^H and ^13^C chemical shifts have been calculated relative to benzene as primary reference (absolute shielding values of σ 24.3 and 45.5, respectively, at the same level) converted into the TMS scale using the experimental δ values of benzene (7.3 and 128.5, respectively).

The QTAIM calculation was performed using the AIM2000 program,^76,77^ and the same program was used to obtain the charges of the atoms. The only exception was the charge for Zr, where a bug in the program did not allow us to compute these charges reliably. Here the multiwfn program was used with an ultrafine grid (“lunatic”) setting for the grid.

All NBO calculations were performed using the NBO version 6.0,^55^ and the so-obtained archive file was used as the input for the NRT calculation performed with NBO version 7.0.^56^ All NCI calculations were performed using the program NCI plot.^58^

Chemical structures were drawn in ChemDraw, and graphical representations of all calculated molecules were done in Jmol^78^ which was also used to create the NBO orbitals. The structural metrics such as bond length and angles were calculated using Mercury which was also used for the representation of the X-ray structure.

## Supporting Information Available

Cartesian coordinates of all calculated structures; relevant QTAIM plots (electron density, Laplacian, and Virial); relevant NBO orbital interactions plots and energies; NCI plots; relevant QTAIM and NBO charges; and correlation graphs (PDF)

## Notes

^†^ Previous address: UCL, Department of Chemistry, 20 Gordon Street, London

## Dedication

Dedicated to the late M. L. H. Green.
