# Supplementary material for: Detailed Density Functional Theory Study of the Cationic Zirconocene Compound [Cp(C5H4CMe2C6H4F)ZrMe]+
Source: ACS Omega. 2022 Sep 22;7(39):35136–52. doi: 10.1021/acsomega.2c04053 (PMC9535714; doi:10.1021/acsomega.2c04053)

Detailed DFT study of the cationic zirconocene compound $[Cp(C_5H_4CMe_2C_6H_4F)ZrMe]^+$

Jörg Saßmannshausen

Electronic Supplementary Information

I. Table of Contents

B3LYP Calculations....................S1
Cartesian Coordinates....................S1
IIA....................S1
IIB....................S3
TS-IIA-IIB....................S4
1A....................S5
1B....................S6
TS-1A-1B....................S7
2A....................S8
2B....................S9
TS-2A-2B....................S10
3A....................S11
3B....................S12
TS-3A-3B....................S13
4A....................S14
4B....................S15
TS-4A-4B....................S16
5A....................S17
5B....................S18
TS-5A-5B....................S19
6A....................S20
6B....................S21
ortho tert-butyl-fluorobenzene....................S22
meta tert-butyl-fluorobenzene....................S23
para tert-butyl-fluorobenzene....................S24
tert-butyl-benzene....................S25
PBE-D3 calculations....................S26
Cartesian Coordinates....................S26
IIA....................S26
IIB....................S28
TS-IIA-IIB....................S29
1A....................S30
1B....................S31
TS-1A-1B....................S32
2A....................S33
2B....................S34
TS-2A-2B....................S35
3A....................S36
3B....................S37
TS-3A-3B....................S38
4A....................S39
4B....................S40
TS-4A-4B....................S41
5A....................S42
5B....................S43
TS-5A-5B....................S44
6A....................S45
6B....................S46
ortho tert-butyl-fluorobenzene....................S47

meta tert-butyl-fluorobenzene....S48
para tert-butyl-fluorobenzene....S49
tert-butyl-benzene....S50
Electronic Properties....S51
QTAIM and NBO charges....S51
tert-butyl-fluorobenzene....S51
tert-butyl-benzene....S52
Compounds 6A and 6B....S53
QTAIM and NBO properties....S54
IIA-B3LYP....S54
Bader:....S54
NBO:....S55
Natural Resonance Theory:....S57
Natural Localised Molecular Orbitals (NLMO):....S58
Non-Covalent Interactions (NCI)....S60
IIB-B3LYP....S61
Bader:....S61
NBO:....S62
Natural Resonance Theory:....S65
Natural Localised Molecular Orbitals (NLMO):....S66
Non-Covalent Interactions (NCI)....S69
1A-B3LYP....S70
Bader:....S70
NBO:....S70
Natural Resonance Theory:....S72
Natural Localised Molecular Orbitals (NLMO):....S73
Non-Covalent Interactions (NCI)....S75
1B-B3LYP....S76
Bader:....S76
NBO:....S76
Natural Resonance Theory:....S79
Natural Localised Molecular Orbitals (NLMO):....S80
Non-Covalent Interactions (NCI)....S83
2A-B3LYP....S84
Bader:....S84
NBO:....S84
Natural Resonance Theory:....S87
Natural Localised Molecular Orbitals (NLMO):....S88
Non-Covalent Interactions (NCI)....S91
2B-B3LYP....S92
Bader:....S92
NBO:....S93
Natural Resonance Theory:....S95
Natural Localised Molecular Orbitals (NLMO):....S96
Non-Covalent Interactions (NCI)....S98
3A-B3LYP....S99
Bader:....S99
NOB:....S99
Natural Resonance Theory:....S102
Natural Localised Molecular Orbitals (NLMO):....S103
Non-Covalent Interactions (NCI)....S106
3B-B3LYP....S107

Bader:....S107
NBO:....S109
Natural Resonance Theory:....S111
Natural Localised Molecular Orbitals (NLMO):....S112
Non-Covalent Interactions (NCI)....S113
4A-B3LYP....S114
Bader:....S114
NBO:....S115
Natural Resonance Theory:....S117
Natural Localised Molecular Orbitals (NLMO):....S118
Non-Covalent Interactions (NCI)....S120
4B-B3LYP....S121
Bader:....S121
NBO:....S121
Natural Resonance Theory:....S124
Natural Localised Molecular Orbitals (NLMO):....S125
Non-Covalent Interactions (NCI)....S128
5A-B3LYP....S129
Bader:....S129
NBO:....S130
Natural Resonance Theory:....S132
Natural Localised Molecular Orbitals (NLMO):....S132
Non-Covalent Interactions (NCI)....S134
5B-B3LYP....S135
Bader:....S135
NBO:....S135
Natural Resonance Theory:....S138
Natural Localised Molecular Orbitals (NLMO):....S139
Non-Covalent Interactions (NCI)....S142
6A-B3LYP....S143
Bader:....S143
NBO:....S144
Natural Resonance Theory:....S146
Natural Localised Molecular Orbitals (NLMO):....S146
Non-Covalent Interactions (NCI)....S149
6B-B3LYP....S150
Bader:....S150
NBO:....S151
Natural Resonance Theory:....S153
Natural Localised Molecular Orbitals (NLMO):....S154
Non-Covalent Interactions (NCI)....S156
IIA-PBE-D3....S158
Bader:....S158
NBO:....S159
Natural Resonance Theory:....S161
Natural Localised Molecular Orbitals (NLMO):....S162
Non-Covalent Interactions (NCI)....S165
IIB-PBE-D3....S166
Bader:....S166
NBO:....S167
Natural Resonance Theory:....S170
Natural Localised Molecular Orbitals (NLMO):....S171

Non-Covalent Interactions (NCI)....S175
1A-PBE-D3....S176
Bader:....S176
NBO:....S177
Natural Resonance Theory:....S179
Natural Localised Molecular Orbitals (NLMO):....S180
Non-Covalent Interactions (NCI)....S182
1B-PBE-D3....S183
Bader:....S183
NBO:....S183
Natural Resonance Theory:....S187
Natural Localised Molecular Orbitals (NLMO):....S187
Non-Covalent Interactions (NCI)....S189
2A-PBE-D3....S190
Bader:....S190
NBO:....S190
Natural Resonance Theory:....S193
Natural Localised Molecular Orbitals (NLMO):....S194
Non-Covalent Interactions (NCI)....S197
2B-PBE-D3....S198
Bader:....S198
NBO:....S198
Natural Resonance Theory:....S202
Natural Localised Molecular Orbitals (NLMO):....S203
Non-Covalent Interactions (NCI)....S206
3A-PBE-D3....S207
Bader:....S207
NBO:....S208
Natural Resonance Theory:....S210
Natural Localised Molecular Orbitals (NLMO):....S211
Non-Covalent Interactions (NCI)....S214
3B-PBE-D3....S215
Bader:....S215
NBO:....S216
Natural Resonance Theory:....S219
Natural Localised Molecular Orbitals (NLMO):....S220
Non-Covalent Interactions (NCI)....S222
4A-PBE-D3....S223
Bader:....S223
NBO:....S224
Natural Resonance Theory:....S227
Natural Localised Molecular Orbitals (NLMO):....S228
Non-Covalent Interactions (NCI)....S231
4B-PBE-D3....S232
Bader:....S232
NBO:....S233
Natural Resonance Theory:....S236
Natural Localised Molecular Orbitals (NLMO):....S238
Non-Covalent Interactions (NCI)....S241
5A-PBE-D3....S242
Bader:....S242
NBO:....S243

Natural Resonance Theory: S246
Natural Localised Molecular Orbitals (NLMO): S247
Non-Covalent Interactions (NCI) S250
5B-PBE-D3 S251
Bader: S251
NBO: S251
Natural Resonance Theory: S255
Natural Localised Molecular Orbitals (NLMO): S256
Non-Covalent Interactions (NCI) S259
6A-PBE-D3 S260
Bader: S260
NBO: S260
Natural Resonance Theory: S263
Natural Localised Molecular Orbitals (NLMO): S264
Non-Covalent Interactions (NCI) S267
6B-PBE-D3 S268
Bader: S268
NBO: S268
Natural Resonance Theory: S272
Natural Localised Molecular Orbitals (NLMO): S274
Non-Covalent Interactions (NCI) S278
Miscellaneous S280
Correlation graphs of selected compounds and properties S280
Graph S1: Bader and NBO charges vs. selected compounds S280
Graph S2: Bader charges and Zr-Me bond distances vs. selected compounds S280
Graph S3: Wiberg Bond Index and Zr-Me bond distances vs. selected compounds S281
Graph S4: Natural Binding Index and Zr-Me bond distances vs. selected compounds S281
Graph S5: Bond Critical Point and Zr-Me bond distances vs. selected compounds S282
Graph S6: Calculated ^{13}C chemical shifts and Zr-Me bond distances vs. selected compounds S282

B3LYP Calculations

Cartesian Coordinates

IIA

IIA: E(RB3LYP) = -823.077330238

C	3.060840	0.078243	-1.125300
C	2.011960	0.641109	-1.905939
C	1.524552	1.777004	-1.223019
C	2.276921	1.928206	-0.025594
C	3.234574	0.889662	0.025945
Zr	0.958802	-0.246891	0.214403
C	1.828846	-0.512578	2.297094
C	-1.215600	0.952515	1.146520
C	-2.016256	0.576035	0.036529
C	-2.432080	1.595695	-0.826789
C	-2.112356	2.930580	-0.572391
C	-1.348825	3.291313	0.537106
C	-0.899487	2.297934	1.398748
C	-2.410035	-0.907648	-0.145881
C	-3.221771	-1.151640	-1.431839
C	-3.270433	-1.358400	1.056394
C	-1.093605	-1.682635	-0.209695
C	-0.413733	-2.309292	0.871863
C	0.831225	-2.796041	0.407545
C	0.942333	-2.466388	-0.964336
C	-0.239666	-1.774310	-1.346089
H	-1.124062	4.332359	0.733718
H	0.727602	2.425398	-1.557012
H	1.554669	-3.341671	0.994245
H	-3.047921	1.362364	-1.684766
H	1.670227	0.279730	-2.864069
H	3.667686	-0.773115	-1.396022
H	-0.785896	-2.422196	1.878395
H	-0.464714	-1.426178	-2.342502
H	3.972903	0.744053	0.798647
H	2.162012	2.718634	0.700855
H	1.751292	-2.746760	-1.621986
H	-1.005270	0.223548	1.929209
H	-0.334213	2.554250	2.287271
H	-2.479895	3.698437	-1.243775
H	-2.700315	-0.821737	-2.332277
H	-2.770174	-1.212596	2.014956
H	-3.521396	-2.417219	0.962181
H	-4.201193	-0.788122	1.082255
H	-4.183333	-0.635815	-1.388008

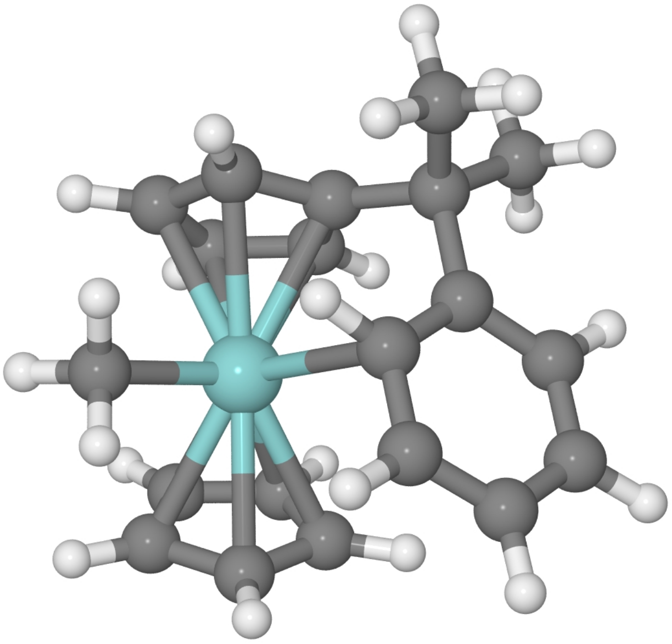

H	-3.422872	-2.219081	-1.536543
H	1.126541	-1.012052	2.972209
H	2.748761	-1.107753	2.308129
H	2.083041	0.464967	2.723521

IIB

IIB: E(RB3LYP) = -823.075607798

C	3.254229	0.913657	0.533212
C	3.408722	-0.322344	-0.146716
C	2.854007	-0.180859	-1.449865
C	2.347646	1.134444	-1.562681
C	2.585713	1.807607	-0.334117
Zr	0.934614	-0.096029	0.198648
C	0.776495	0.776547	2.281743
C	-0.000138	-2.183216	-0.846073
C	-1.044246	-1.682721	-0.011653
C	-0.591687	-1.794255	1.332469
C	0.702804	-2.365291	1.328399
C	1.066633	-2.614065	-0.017530
C	-2.359100	-1.037406	-0.450742
C	-3.515052	-1.590617	0.405507
C	-1.087704	1.119306	-0.952818
C	-2.148286	0.478459	-0.261486
C	-2.924610	1.264975	0.590677
C	-2.680437	2.632350	0.730383
C	-1.654171	3.259110	0.025601
C	-0.864755	2.502167	-0.829927
C	-2.677167	-1.340758	-1.931797
H	1.975738	-3.089128	-0.353564
H	1.293336	-2.599227	2.202319
H	-1.139143	-1.504803	2.215297
H	-3.743007	0.823232	1.142509
H	-3.311115	3.216329	1.390927
H	-1.489048	4.324382	0.126752
H	-0.095062	2.978063	-1.424951
H	-0.584046	0.586592	-1.765968
H	-1.906426	-0.983705	-2.617486
H	-3.615276	-0.859610	-2.215300
H	-2.789497	-2.416987	-2.080203
H	-3.377568	-1.404940	1.471904
H	-3.585361	-2.670513	0.264486
H	-4.468063	-1.152319	0.101088
H	-0.031867	-2.275931	-1.921526
H	3.594490	1.134893	1.532850
H	2.329656	2.832128	-0.107841
H	1.896000	1.562265	-2.446912
H	2.859141	-0.929669	-2.227923
H	3.920333	-1.193727	0.234240
H	-0.272455	0.746712	2.598139
H	1.351938	0.236235	3.041490
H	1.109356	1.818345	2.298239

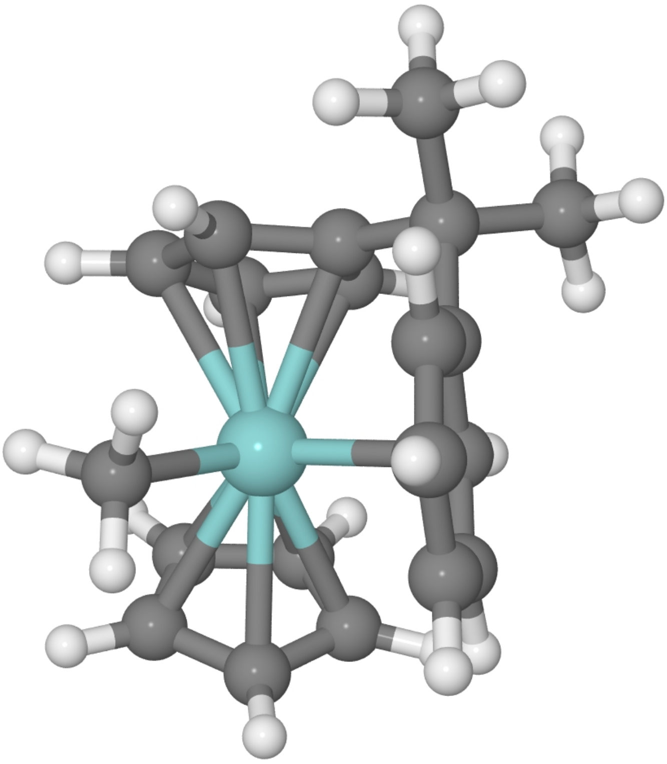

TS-IIA-IIB

TS-IIA-IIB: E(RB3LYP) = -823.069531471

Zr	0.803887	-0.234719	0.200895
C	-0.308299	-1.971757	-1.178346
C	-1.251424	-1.627266	-0.161921
C	-0.718287	-2.084223	1.072292
C	0.531469	-2.701786	0.826687
C	0.779730	-2.644619	-0.564882
H	1.626517	-3.076095	-1.076588
H	1.163920	-3.165792	1.568754
H	-1.178224	-1.985707	2.041972
C	-2.461458	-0.707689	-0.328261
C	-1.878324	0.720861	-0.134305
C	-1.311596	1.434700	-1.213966
C	-0.899838	2.766886	-1.070026
C	-0.981950	3.401024	0.162290
C	-1.452832	2.684036	1.262409
C	-1.889688	1.367169	1.119673
H	-2.296573	0.863558	1.985358
H	-1.507756	3.156663	2.236020
H	-0.683603	4.436733	0.269715
H	-0.533085	3.304554	-1.936652
H	-1.292643	0.995307	-2.203258
C	-3.545721	-1.030483	0.718238
H	-3.936633	-2.033363	0.536730
H	-3.182470	-1.006187	1.745671
H	-4.373850	-0.322995	0.637009
C	-3.101161	-0.873401	-1.721510
H	-3.503817	-1.883395	-1.818886
H	-3.923649	-0.165587	-1.845739
H	-2.403851	-0.722631	-2.545928
H	-0.416219	-1.800502	-2.238783
C	3.244579	-0.477830	-0.349416
C	2.608960	-0.135364	-1.576944
C	2.135907	1.189338	-1.464011
C	2.467167	1.669838	-0.167775
C	3.175313	0.647889	0.508795
H	3.593732	0.714335	1.500219
H	2.254261	2.655181	0.220366
H	1.634782	1.746916	-2.240217
H	2.537166	-0.762656	-2.452735
H	3.748167	-1.408472	-0.132726
C	1.058774	-0.006208	2.455074
H	0.127037	-0.220909	2.985249
H	1.820902	-0.674904	2.868748
H	1.362479	1.018027	2.695342

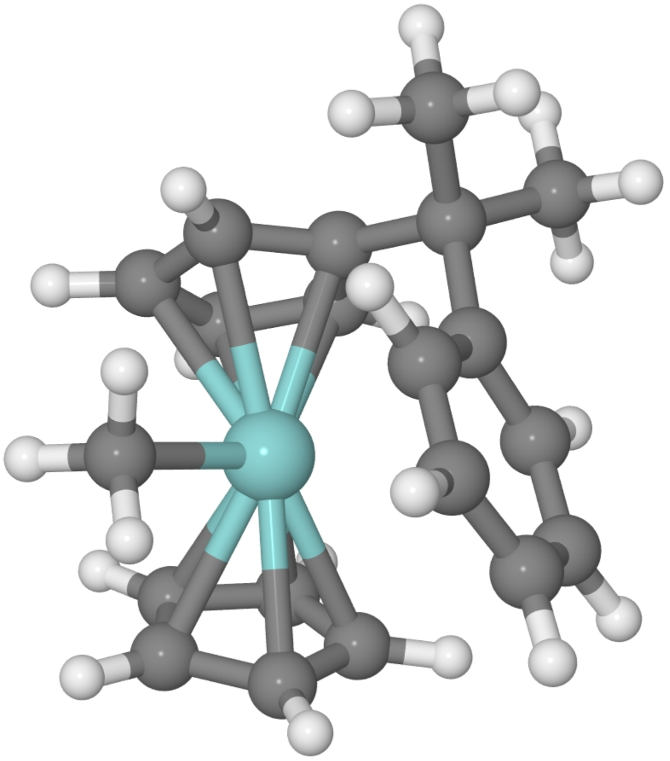

1A

1A: E(RB3LYP) = -922.347845314

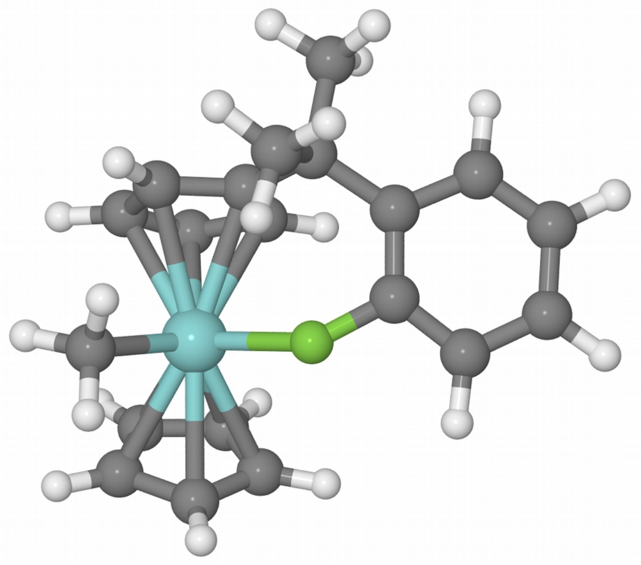

C	-1.853302	1.170703	0.408743
C	-2.569389	0.048328	0.020402
C	-3.843899	0.348967	-0.485408
C	-4.321740	1.655716	-0.567188
C	-3.542367	2.726165	-0.140823
C	-2.265594	2.481836	0.361797
C	-2.054683	-1.389799	0.220572
C	-2.090998	-1.702627	1.737965
F	-0.520605	0.980900	0.921897
Zr	1.346791	-0.075041	0.164831
C	-0.185015	-1.023233	-1.599124
C	-0.654589	-1.568182	-0.364937
C	0.367004	-2.424976	0.125174
C	1.451141	-2.406543	-0.789776
C	1.101191	-1.552398	-1.865193
C	2.459458	1.447118	-1.515140
C	1.993775	2.307597	-0.495241
C	2.716622	2.019040	0.692538
C	3.645520	0.990824	0.401956
C	3.478035	0.621790	-0.957562
C	2.042618	-0.901613	2.156432
C	-2.955792	-2.421239	-0.502774
H	-1.619851	3.276760	0.711622
H	-3.914308	3.741419	-0.194315
H	-5.314074	1.833627	-0.962712
H	-4.481313	-0.458474	-0.817273
H	-1.433549	-1.050217	2.315815
H	-3.106811	-1.569073	2.113759
H	-1.804014	-2.738936	1.927420
H	-2.530609	-3.418425	-0.378297
H	-3.960790	-2.436784	-0.076124
H	-3.028769	-2.219648	-1.573299
H	-0.735200	-0.350384	-2.242369
H	1.699314	-1.354892	-2.741969
H	2.365766	-2.974351	-0.695767
H	0.332332	-3.006921	1.031635
H	2.127975	1.442637	-2.542760
H	4.066883	-0.108386	-1.493320
H	4.359299	0.567396	1.091336
H	2.597538	2.513744	1.645816
H	1.229465	3.063704	-0.606784
H	2.842452	-1.646314	2.075039
H	2.409921	-0.102435	2.807183
H	1.204162	-1.395366	2.660585

1B

1B: E(RB3LYP) = -922.331624259

C	-3.587106	-0.169410	-0.010762
C	-3.098610	-0.102837	1.324512
C	-2.536060	1.180067	1.514702
C	-2.673149	1.908051	0.301859
C	-3.336360	1.081229	-0.633194
Zr	-1.093123	-0.057555	-0.214999
C	-0.777473	0.876939	-2.252409
C	0.780447	-1.755852	0.066164
C	0.421857	-1.789413	-1.310302
C	-0.896351	-2.289818	-1.421893
C	-1.369297	-2.570624	-0.116393
C	-0.343894	-2.231806	0.803504
C	2.102803	-1.217254	0.614314
C	2.275308	-1.541942	2.115937
C	0.952908	1.003973	1.092716
C	2.007315	0.316806	0.432476
C	2.833535	1.122459	-0.353186
C	2.664984	2.500439	-0.465916
C	1.641434	3.145359	0.215875
C	0.782918	2.393526	1.010209
F	3.855230	0.591716	-1.046354
C	3.267100	-1.899658	-0.138142
H	3.361372	3.047492	-1.090201
H	1.529745	4.219349	0.140062
H	0.007438	2.877823	1.589312
H	0.386449	0.463028	1.856254
H	3.210017	-2.974563	0.044760
H	3.232365	-1.737364	-1.213590
H	4.231221	-1.543187	0.225105
H	2.299067	-2.624027	2.260933
H	3.221315	-1.133100	2.475802
H	1.481278	-1.135230	2.745034
H	-0.394834	-2.368567	1.873614
H	-2.323344	-3.009128	0.133886
H	-1.433460	-2.456300	-2.344394
H	1.048863	-1.498224	-2.138136
H	0.280308	0.797286	-2.528079
H	-1.348442	0.397545	-3.055323
H	-1.052011	1.935848	-2.245506
H	-4.119360	-0.999274	-0.451388
H	-3.610964	1.355313	-1.639921
H	-2.357025	2.926154	0.129004
H	-2.113497	1.552226	2.437675
H	-3.182516	-0.877729	2.071808

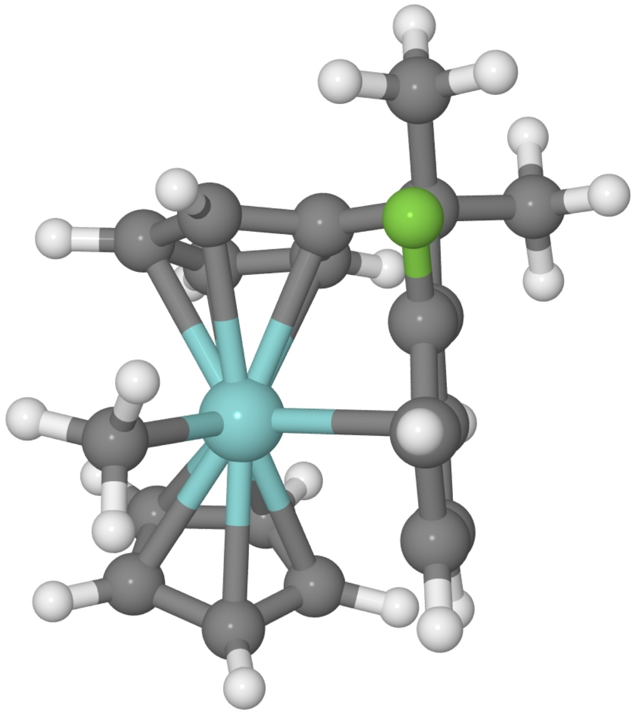

TS-1A-1B

TS-1A-1B: E(RB3LYP) = -922.328314057

Zr	0.947044	-0.149653	0.224881
C	0.211331	-2.210710	-0.939590
C	-0.918854	-1.774158	-0.189421
C	-0.596227	-1.936745	1.187624
C	0.707661	-2.471763	1.286373
C	1.211999	-2.641351	-0.025741
H	2.161883	-3.081026	-0.289708
H	1.219806	-2.723792	2.203044
H	-1.234352	-1.688684	2.020076
C	-2.138111	-1.028028	-0.732452
C	-1.820978	0.471739	-0.443230
C	-1.243901	1.306053	-1.430871
C	-1.055357	2.679496	-1.241070
C	-1.411762	3.272440	-0.037463
C	-1.920048	2.478319	0.987560
C	-2.100268	1.119127	0.780917
F	-2.581923	0.412331	1.823317
H	-2.195124	2.893403	1.949352
H	-1.294998	4.338537	0.112516
H	-0.653629	3.277735	-2.049736
H	-1.029870	0.891404	-2.406309
C	-3.444143	-1.510698	-0.060198
H	-3.643085	-2.535839	-0.378295
H	-3.406876	-1.496522	1.024519
H	-4.284209	-0.889482	-0.380084
C	-2.304961	-1.277540	-2.245568
H	-2.525089	-2.333582	-2.410132
H	-3.141879	-0.694800	-2.635490
H	-1.421711	-1.036524	-2.838331
H	0.287757	-2.258805	-2.014764
C	3.421106	-0.203348	-0.185853
C	2.825161	-0.099843	-1.475086
C	2.213552	1.168847	-1.568049
C	2.425322	1.854390	-0.339952
C	3.193128	1.016264	0.502053
H	3.547142	1.261435	1.490689
H	2.091567	2.854302	-0.103337
H	1.700290	1.559764	-2.433785
H	2.865691	-0.845485	-2.254843
H	4.006161	-1.034905	0.178346
C	0.975438	0.465664	2.409630
H	0.017284	0.239930	2.886812
H	1.750545	-0.033944	3.000465
H	1.157708	1.543108	2.486241

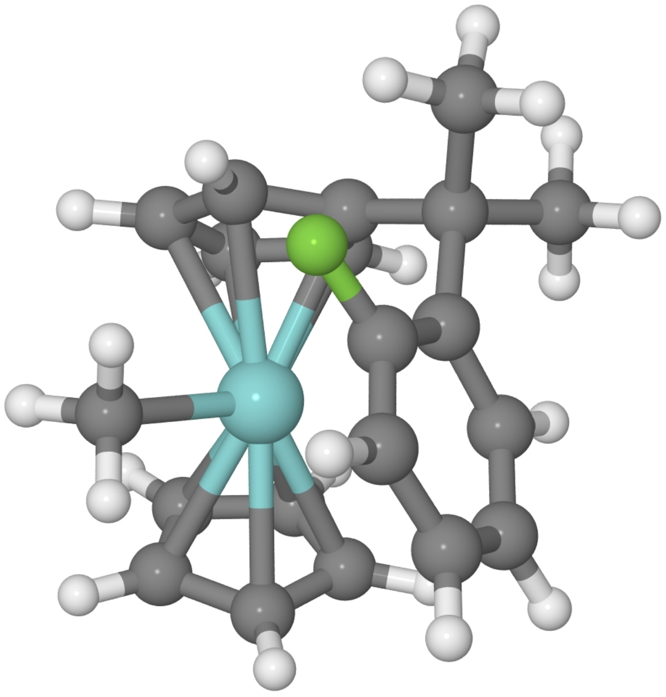

2A

2A: E(RB3LYP) = -922.340131103

C	3.143072	-0.076967	-1.108244
C	2.114113	0.310531	-2.012487
C	1.536240	1.503635	-1.526714
C	2.212719	1.865895	-0.327867
C	3.215284	0.900129	-0.081209
Zr	0.992607	-0.316702	0.168783
C	1.827323	-0.328225	2.279293
C	-1.212107	0.968102	0.853669
C	-2.062164	0.387221	-0.128569
C	-2.565099	1.224502	-1.125108
C	-2.291359	2.595465	-1.118986
C	-1.492288	3.178982	-0.139326
C	-0.973902	2.348178	0.837113
C	-2.384002	-1.118671	-0.034225
C	-3.251202	-1.614999	-1.207266
F	-0.216906	2.868703	1.813973
C	-3.151818	-1.393922	1.278964
C	-1.034185	-1.836985	-0.055442
C	-0.208430	-2.009878	-1.204933
C	1.000850	-2.634781	-0.796640
C	0.937612	-2.835551	0.603387
C	-0.307875	-2.340812	1.058413
H	-2.722368	3.223735	-1.889996
H	-0.962459	0.429952	1.768423
H	-1.290842	4.242095	-0.114644
H	-3.210389	0.827055	-1.895748
H	-2.608657	-1.068802	2.168044
H	-4.107893	-0.866853	1.267492
H	-3.353934	-2.462627	1.377347
H	-3.401492	-2.691776	-1.113973
H	-4.234737	-1.140429	-1.193371
H	-2.794225	-1.427279	-2.180486
H	-0.470285	-1.759059	-2.221807
H	1.801071	-2.951773	-1.448548
H	1.693934	-3.301748	1.216870
H	-0.648254	-2.367133	2.081841
H	1.842151	-0.203385	-2.922215
H	3.800304	-0.926811	-1.220750
H	3.915921	0.907530	0.738603
H	2.017245	2.739675	0.276318
H	0.732900	2.055145	-1.993663
H	2.772581	-0.875457	2.366957
H	2.025822	0.701022	2.599605
H	1.134808	-0.784415	2.993702

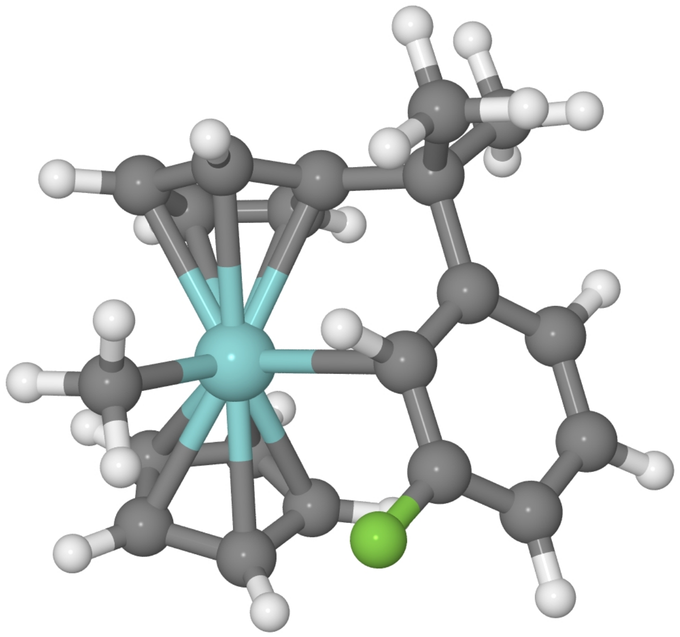

2B

2B: E(RB3LYP) = -922.337926285

C	1.254697	-1.994701	-1.163280
C	1.077906	-0.597387	-1.152761
C	1.989305	0.249003	-0.463675
C	3.011041	-0.353323	0.263066
C	3.133228	-1.740289	0.265457
C	2.276229	-2.581819	-0.435455
Zr	-1.093129	-0.079593	0.211484
C	-0.825272	2.276049	-0.637433
C	0.378661	1.994069	0.074631
C	0.027201	1.848011	1.445780
C	-1.366477	2.050476	1.579307
C	-1.893572	2.323714	0.293248
C	1.775546	1.774387	-0.506369
C	2.812323	2.546514	0.332959
F	4.125945	-2.281171	0.969824
C	-3.557179	-0.488458	0.000273
C	-3.075360	-0.367477	-1.333427
C	-2.253685	-1.487483	-1.598926
C	-2.224356	-2.300618	-0.434163
C	-3.039898	-1.691690	0.546835
C	-0.533955	-1.081581	2.166331
C	1.876184	2.274594	-1.964899
H	3.737746	0.221861	0.819440
H	2.435556	-3.651873	-0.418130
H	0.600522	-2.615996	-1.762053
H	0.404669	-0.158150	-1.893851
H	2.581144	3.612513	0.301443
H	2.819821	2.241588	1.380622
H	3.818223	2.412361	-0.071273
H	1.694658	3.350727	-2.005752
H	2.878948	2.085425	-2.352842
H	1.167431	1.787108	-2.637032
H	-0.908918	2.478473	-1.694852
H	-2.919629	2.569467	0.065272
H	-1.922276	2.031568	2.505595
H	0.703018	1.632860	2.258015
H	0.482520	-0.788346	2.451327
H	-1.186602	-0.811634	3.004260
H	-0.562657	-2.171178	2.070896
H	-4.250301	0.180747	0.488038
H	-3.242023	-2.079684	1.533095
H	-1.699475	-3.237799	-0.322431
H	-1.769982	-1.705079	-2.541013
H	-3.326128	0.417336	-2.031541

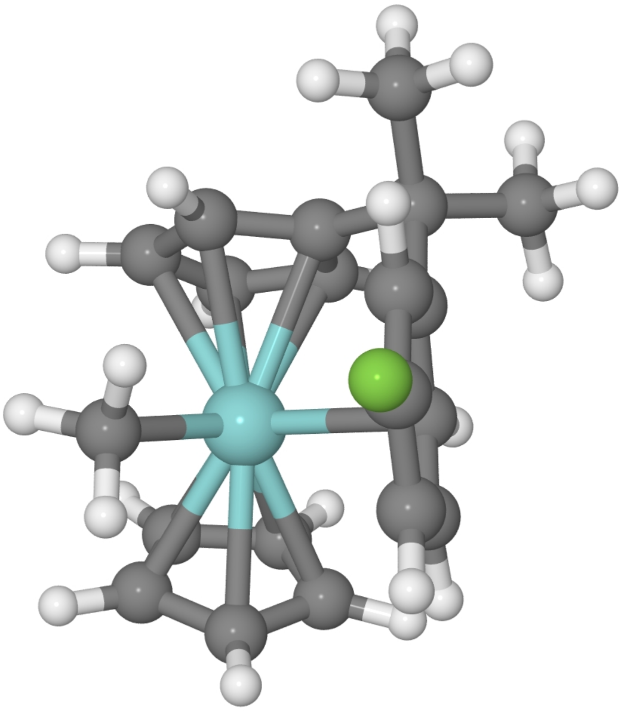

TS-2A-2B

TS-2A-2B: E(RB3LYP) = -922.331083369

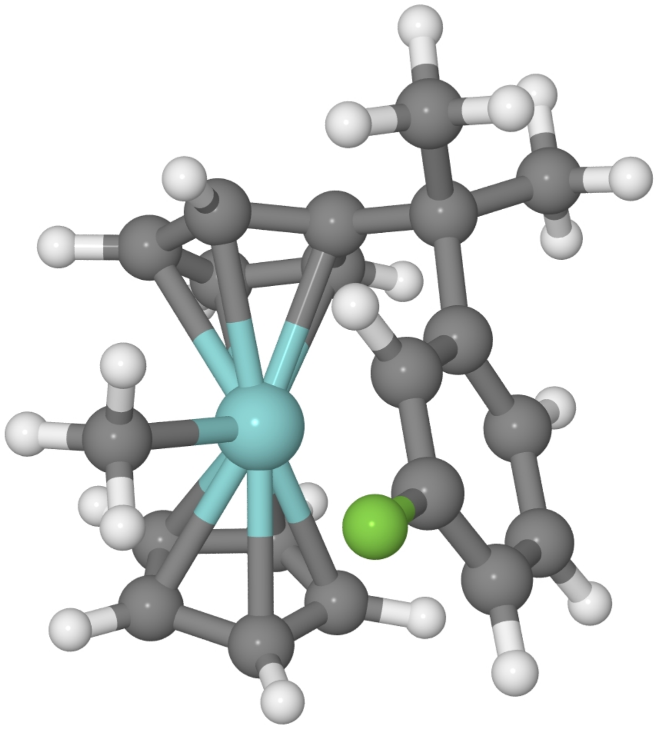

Zr	0.909451	0.100766	-0.201173
C	0.405561	2.295733	0.846441
C	-0.681653	2.035413	-0.043470
C	-0.163578	2.097427	-1.364040
C	1.219302	2.392047	-1.294914
C	1.568576	2.528946	0.069580
H	2.540131	2.803315	0.451516
H	1.880003	2.525389	-2.138639
H	-0.718555	1.952791	-2.276371
C	-2.066095	1.522391	0.353473
C	-1.878209	-0.017564	0.440532
C	-1.423539	-0.641670	1.623202
C	-1.366566	-2.040203	1.727350
C	-1.679025	-2.852875	0.648671
C	-2.030939	-2.230096	-0.543622
C	-2.128985	-0.848723	-0.668667
H	-2.470481	-0.453199	-1.614423
F	-2.298565	-2.981198	-1.615022
H	-1.657386	-3.933333	0.710245
H	-1.086561	-2.490084	2.672460
H	-1.231237	-0.052424	2.509623
C	-3.122118	1.911672	-0.699144
H	-3.222499	2.998259	-0.724877
H	-2.873773	1.586360	-1.709533
H	-4.093797	1.486718	-0.438034
C	-2.517498	2.114439	1.703607
H	-2.646750	3.193449	1.601112
H	-3.475157	1.683633	2.003581
H	-1.807941	1.949449	2.514678
H	0.353311	2.357635	1.922859
C	3.372975	-0.208863	0.177303
C	2.787730	-0.172928	1.475261
C	1.997752	-1.333093	1.625466
C	2.080840	-2.085440	0.422727
C	2.951044	-1.402192	-0.460453
H	3.246677	-1.733956	-1.442822
H	1.600257	-3.033208	0.228516
H	1.449181	-1.609742	2.512754
H	2.951066	0.585371	2.226302
H	4.067559	0.512838	-0.226480
C	0.842636	-0.612976	-2.366586
H	-0.055201	-0.263350	-2.883322
H	1.700281	-0.266783	-2.953132
H	0.850663	-1.707669	-2.400205

3A

3A: E(RB3LYP) = -922.333710412

Zr	1.129404	-0.170357	0.180041
C	-0.740266	-1.849810	-0.147089
C	-2.139978	-1.262997	0.047911
C	-1.918287	0.256397	0.279319
C	-2.478684	1.308239	-0.456271
C	-2.302832	2.648748	-0.120654
C	-1.519377	3.006327	0.969333
C	-0.907500	2.005632	1.718329
C	-1.104095	0.662523	1.372188
H	-0.759109	-0.096734	2.071654
H	-0.314626	2.250615	2.591097
H	-1.399798	4.049539	1.233514
H	-2.804805	3.393638	-0.726804
F	-3.252633	1.063649	-1.526641
C	-2.832946	-1.858603	1.295666
H	-2.285033	-1.681212	2.222366
H	-3.826708	-1.421568	1.411709
H	-2.948947	-2.937594	1.173045
C	-3.010618	-1.617093	-1.176705
H	-3.056507	-2.704531	-1.262027
H	-4.026798	-1.242465	-1.056422
H	-2.617979	-1.219162	-2.110921
C	0.104312	-2.419655	0.846540
C	1.358043	-2.717971	0.261811
C	1.308530	-2.325789	-1.097255
C	0.018111	-1.785497	-1.351090
H	-0.336633	-1.434722	-2.307999
H	2.087616	-2.472534	-1.829905
H	2.194411	-3.181645	0.762722
H	-0.162784	-2.619711	1.872778
C	1.301732	1.978311	-1.176789
C	2.146274	2.164123	-0.047661
C	3.220992	1.251432	-0.148140
C	3.031389	0.481590	-1.325549
C	1.850194	0.946366	-1.969996
H	1.460000	0.593544	-2.912751
H	3.706627	-0.271835	-1.703279
H	4.044870	1.161315	0.542209
H	2.011650	2.898256	0.732684
H	0.406490	2.540854	-1.397797
C	2.204423	-0.389506	2.169569
H	3.191748	-0.855850	2.079264
H	2.363565	0.598602	2.616957
H	1.633786	-1.001999	2.875217

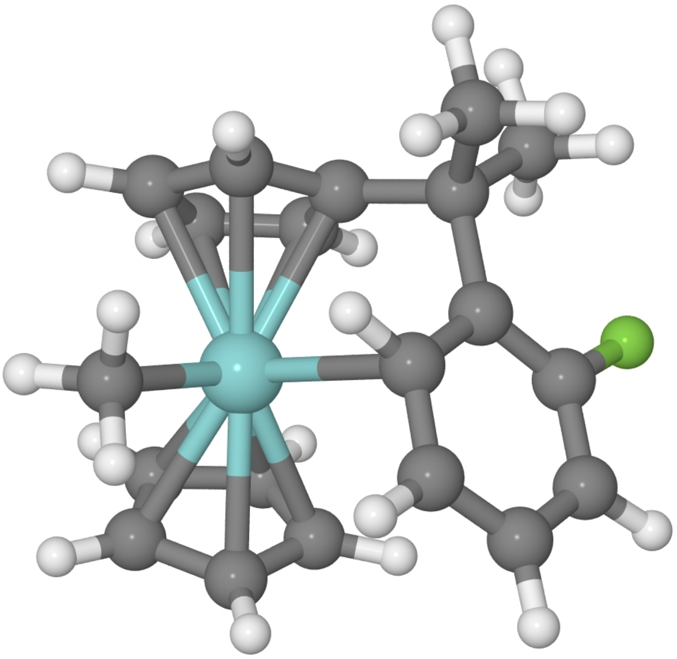

3B

3B: E(RB3LYP) = -922.349725736

C	1.931102	-1.156613	-0.288546
C	2.574053	0.070986	-0.230649
C	3.930221	-0.025196	0.116324
C	4.546428	-1.249947	0.368293
C	3.828455	-2.438282	0.282588
C	2.476337	-2.396459	-0.054543
C	1.878051	1.398679	-0.582554
C	1.614346	1.421719	-2.108832
F	0.528842	-1.172182	-0.629570
Zr	-1.301839	-0.154505	0.263478
C	-1.465796	-1.382516	2.158053
C	-2.715296	-1.907966	-0.930630
C	-3.573932	-1.267027	-0.005905
C	-3.711217	0.087988	-0.404448
C	-2.959002	0.273320	-1.599423
C	-2.345024	-0.958193	-1.920933
C	0.405895	1.207016	1.589051
C	0.590132	1.565781	0.222430
C	-0.604321	2.205809	-0.203466
C	-1.508886	2.250846	0.897429
C	-0.873215	1.643132	2.007756
C	2.765753	2.618695	-0.233912
H	-1.715698	-1.149647	-2.778726
H	-2.899918	1.183704	-2.176457
H	-4.336990	0.827855	0.072411
H	-4.046787	-1.728769	0.846672
H	-2.417700	-2.946520	-0.904189
H	-1.287381	1.529443	2.998076
H	-2.478063	2.726946	0.898929
H	-0.787667	2.628656	-1.179099
H	1.871402	-3.290323	-0.133322
H	4.304824	-3.391067	0.475393
H	5.596482	-1.270769	0.632395
H	4.519105	0.878358	0.189410
H	3.678802	2.627009	-0.832928
H	2.217802	3.535972	-0.455686
H	3.035669	2.637668	0.823600
H	2.557259	1.304786	-2.645678
H	0.947989	0.618512	-2.429974
H	1.178924	2.375820	-2.413048
H	1.129746	0.698423	2.209295
H	-0.541871	-1.311863	2.741781
H	-2.284966	-1.091271	2.824342
H	-1.631462	-2.433726	1.895306

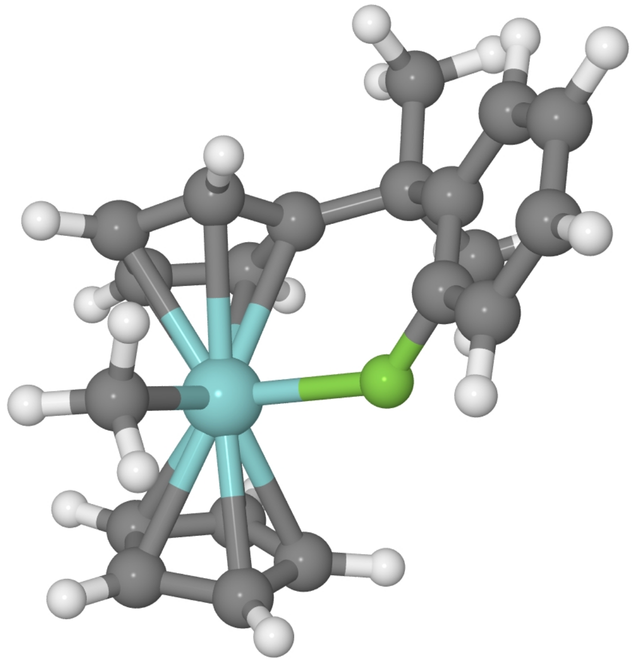

TS-3A-3B

TS-3A-3B: E(RB3LYP) = -922.327970651

Zr	-0.010337	-0.067662	0.041595
C	-0.038733	0.166800	2.520842
C	1.334181	0.017517	2.150459
C	1.490593	-1.309920	1.664183
C	0.237032	-1.964351	1.722051
C	-0.704151	-1.056508	2.267269
H	-1.740051	-1.269849	2.483709
H	0.046297	-2.989632	1.440568
H	2.404213	-1.760014	1.313517
C	2.389860	1.124846	2.092265
C	2.178140	1.825277	0.716322
C	1.135735	2.742015	0.458225
C	1.027114	3.480266	-0.719197
C	1.953567	3.286123	-1.732372
C	2.968453	2.345668	-1.555550
C	3.071932	1.638410	-0.363726
H	3.898375	0.952680	-0.246046
H	3.699434	2.179305	-2.337113
H	1.888117	3.865348	-2.645031
H	0.232659	4.211661	-0.800555
F	0.201629	2.998983	1.413676
C	3.800165	0.510909	2.197521
H	3.904299	0.030669	3.171850
H	4.010270	-0.243744	1.439285
H	4.562805	1.288772	2.122813
C	2.248764	2.117405	3.269230
H	2.447298	1.591364	4.205238
H	2.986332	2.917192	3.169314
H	1.266126	2.575074	3.340622
H	-0.492043	1.050952	2.941233
C	-2.328018	-0.757171	-0.601935
C	-2.461479	0.534615	-0.015994
C	-1.842355	1.466076	-0.877447
C	-1.320099	0.758916	-1.994274
C	-1.641339	-0.610277	-1.834451
H	-1.409006	-1.402207	-2.528775
H	-0.801345	1.192858	-2.836863
H	-1.798387	2.532950	-0.719753
H	-2.974396	0.766878	0.905197
H	-2.736511	-1.677208	-0.210596
C	1.209116	-1.369358	-1.365069
H	2.255371	-1.415504	-1.047829
H	0.852165	-2.403898	-1.420307
H	1.177215	-0.963572	-2.381566

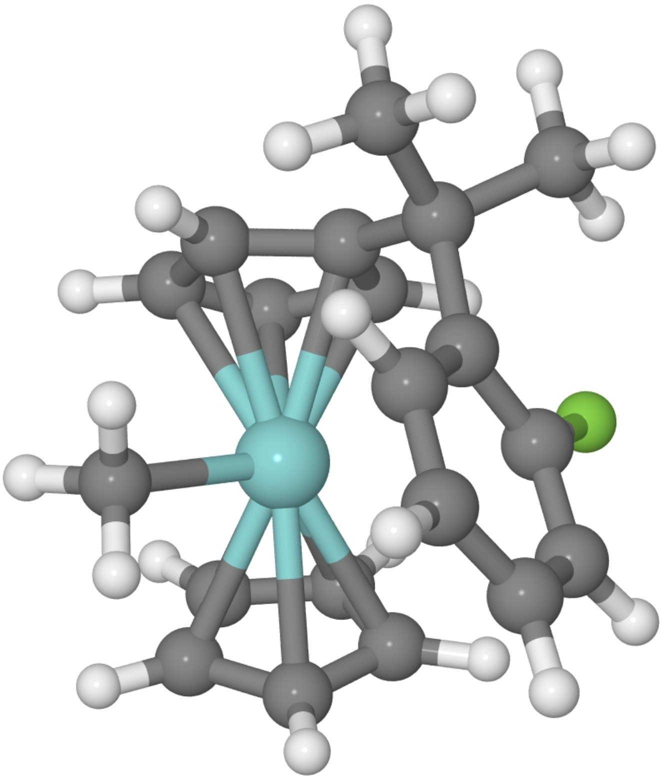

4A

4A: E(RB3LYP) = -922.339140116

C	1.230407	-0.180515	1.304275
C	1.911692	0.447407	0.225841
C	2.799001	-0.328567	-0.519364
C	3.030874	-1.652899	-0.158794
C	2.404977	-2.269370	0.919335
C	1.503098	-1.516020	1.654524
C	1.652108	1.940169	-0.069316
C	2.102146	2.785416	1.143957
F	3.900224	-2.358213	-0.884104
Zr	-1.165056	-0.063687	0.187810
C	-0.535409	1.659380	-1.494103
C	0.146411	2.056812	-0.307208
C	-0.847142	2.441863	0.634572
C	-2.121086	2.305732	0.033028
C	-1.931374	1.824038	-1.284126
C	-1.616042	-1.502693	-1.842238
C	-0.754205	-2.266140	-1.024580
C	-1.458374	-2.603756	0.164287
C	-2.757898	-2.054627	0.076372
C	-2.853514	-1.354458	-1.154240
C	-2.228353	0.027205	2.193509
C	2.415594	2.440619	-1.310315
H	0.677212	0.430370	2.016939
H	1.023536	-1.946750	2.525539
H	2.643102	-3.295426	1.168082
H	3.354293	0.076416	-1.354006
H	1.608414	2.495314	2.072670
H	3.177732	2.675266	1.295541
H	1.890339	3.841333	0.962664
H	2.153757	3.482655	-1.500663
H	3.494722	2.394233	-1.148455
H	2.180669	1.869954	-2.210675
H	-0.075900	1.338347	-2.416664
H	-2.704625	1.667193	-2.020941
H	-3.066316	2.549308	0.494344
H	-0.671062	2.801720	1.636252
H	-1.387027	-1.122154	-2.826282
H	-3.735458	-0.858660	-1.532207
H	-3.541977	-2.156774	0.810000
H	-1.082423	-3.208877	0.975807
H	0.256312	-2.560212	-1.268827
H	-3.310112	0.174487	2.100084
H	-2.087237	-0.917172	2.732499
H	-1.855176	0.843251	2.820317

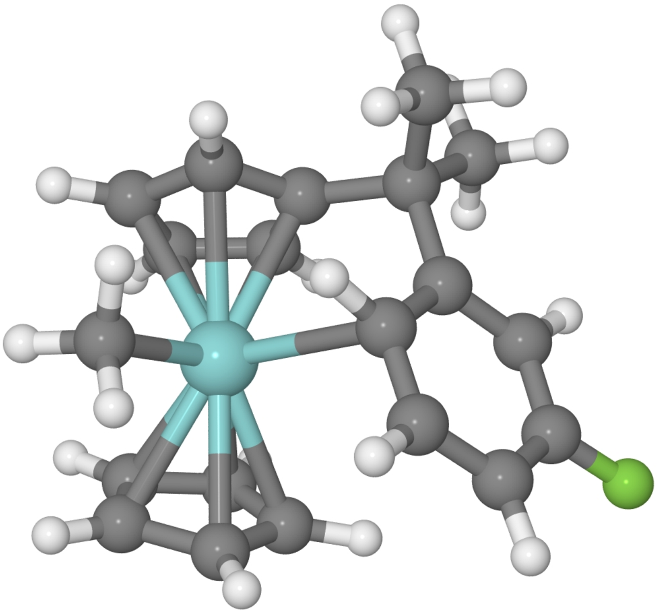

4B

4B: E(RB3LYP) = -922.338761772

C	3.234089	0.803759	0.678550
C	3.432613	-0.356563	-0.114637
C	2.875225	-0.110096	-1.401686
C	2.321358	1.190049	-1.391283
C	2.531101	1.750295	-0.102582
Zr	0.956486	-0.238506	0.239474
C	0.761052	0.427511	2.396964
C	-1.129131	1.073760	-0.770809
C	-1.006737	2.439628	-0.478517
C	-1.791725	3.068366	0.468973
C	-2.764564	2.303173	1.105917
C	-2.949493	0.950621	0.805309
C	-2.158917	0.313674	-0.148705
F	-0.101213	3.158554	-1.161765
C	-2.315302	-1.171097	-0.534153
C	-2.596900	-1.292631	-2.048394
C	0.068956	-2.225586	-1.023007
C	-0.989168	-1.829148	-0.151901
C	-0.545573	-2.068074	1.179327
C	0.757508	-2.615501	1.129459
C	1.137141	-2.719057	-0.231275
C	-3.468124	-1.864182	0.218670
H	-3.404325	2.773427	1.843603
H	-3.737001	0.408918	1.309428
H	-1.659800	4.122852	0.673041
H	-0.637710	0.707311	-1.673581
H	-3.504002	-2.916535	-0.067948
H	-3.350832	-1.822255	1.302476
H	-4.429086	-1.414757	-0.041397
H	-2.678911	-2.344360	-2.330561
H	-3.541235	-0.801916	-2.291819
H	-1.821933	-0.838146	-2.668655
H	0.049619	-2.207826	-2.102650
H	2.057737	-3.141433	-0.604487
H	1.346410	-2.925090	1.980653
H	-1.106363	-1.880716	2.081171
H	-0.291692	0.354613	2.694489
H	1.331004	-0.182610	3.106708
H	1.084263	1.464066	2.527992
H	3.974180	-1.242087	0.183965
H	3.567278	0.941027	1.695505
H	2.231312	2.738874	0.211506
H	1.845916	1.688504	-2.223311
H	2.906208	-0.781997	-2.246518

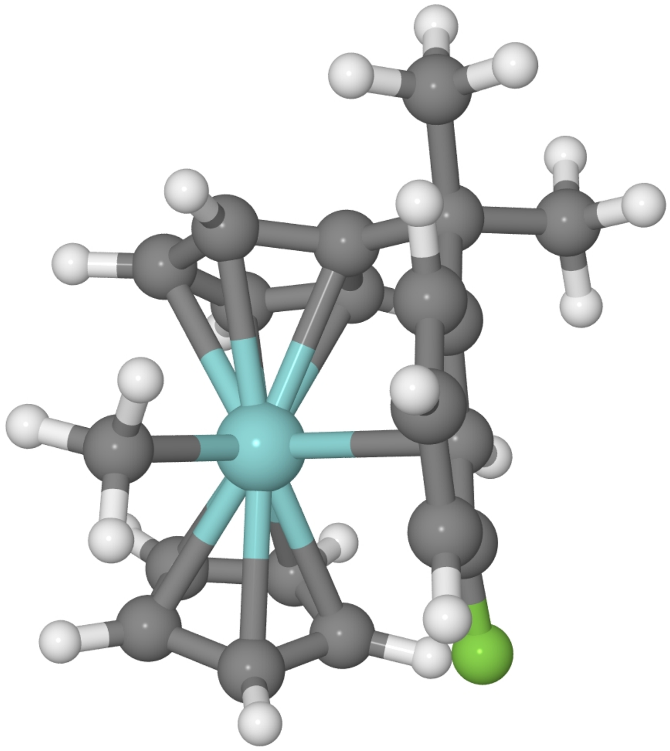

TS-4A-4B

TS-4A-4B: E(RB3LYP) = -922.332524089

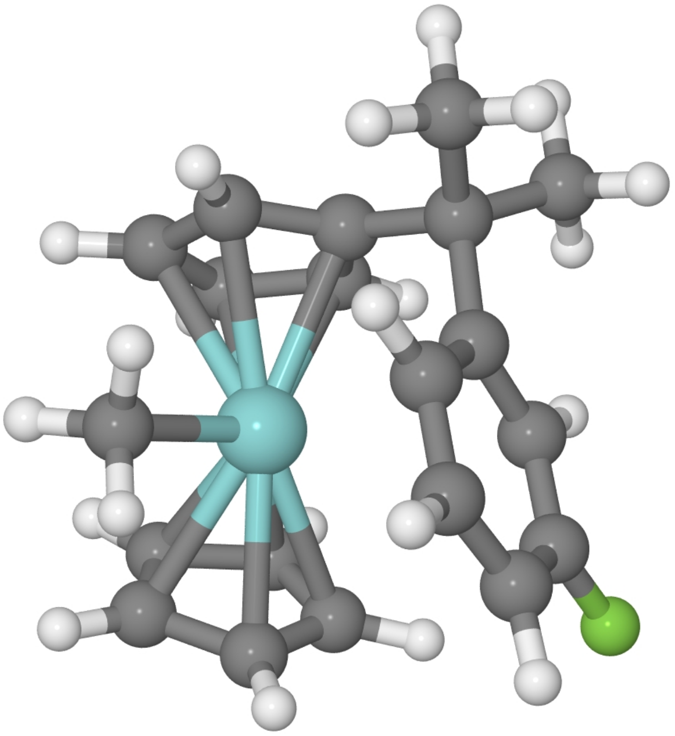

Zr	0.854397	-0.366204	0.200387
C	-0.202990	-1.822629	-1.508745
C	-1.099563	-1.819158	-0.395622
C	-0.450818	-2.505747	0.664206
C	0.824673	-2.927688	0.217127
C	0.972286	-2.519101	-1.129451
H	1.813950	-2.734957	-1.769872
H	1.540412	-3.495133	0.793208
H	-0.851762	-2.686917	1.648055
C	-2.385263	-0.998836	-0.283867
C	-1.888295	0.414436	0.131339
C	-1.498271	1.357307	-0.843744
C	-1.156614	2.653809	-0.463188
C	-1.121305	3.063072	0.858376
C	-1.414036	2.110976	1.830583
C	-1.777805	0.806011	1.481479
H	-2.046280	0.119547	2.271344
H	-1.379736	2.387486	2.877509
H	-0.865359	4.084933	1.107205
F	-0.826561	3.522366	-1.430966
H	-1.562357	1.144769	-1.902232
C	-3.336464	-1.598071	0.769275
H	-3.663609	-2.586143	0.440547
H	-2.879916	-1.717966	1.751721
H	-4.220839	-0.967736	0.883874
C	-3.141017	-0.962329	-1.627343
H	-3.478976	-1.969042	-1.879895
H	-4.019127	-0.317283	-1.551045
H	-2.535328	-0.606981	-2.461533
H	-0.394684	-1.415056	-2.489783
C	3.247699	-0.238186	-0.543426
C	2.490342	0.378515	-1.580353
C	1.949757	1.578139	-1.067361
C	2.356673	1.701865	0.288470
C	3.178662	0.592440	0.602830
H	3.672965	0.415130	1.544531
H	2.121901	2.521497	0.951792
H	1.355796	2.291023	-1.619548
H	2.386314	0.017739	-2.592745
H	3.826137	-1.145830	-0.632624
C	1.218401	-0.649913	2.434435
H	0.335406	-1.056422	2.935333
H	2.046031	-1.334769	2.647141
H	1.465925	0.309489	2.900088

5A

5A: E(RB3LYP) = -922.336453540

C	-2.332828	-2.385282	-0.012780
C	-2.714182	-1.550208	-1.095288
C	-1.584607	-1.390240	-1.946918
C	-0.506489	-2.100271	-1.374463
C	-0.967372	-2.712260	-0.175607
Zr	-1.061685	-0.186311	0.214481
C	-1.818616	-0.560944	2.324959
C	-1.010634	2.247688	1.004949
C	-0.156636	2.157216	-0.129996
C	-0.981097	1.826927	-1.244083
C	-2.327019	1.733627	-0.794490
C	-2.341703	1.995075	0.596118
C	1.371596	2.220219	-0.140742
C	1.856395	2.908544	-1.430569
C	1.457885	-0.034391	1.072833
C	1.854825	0.754417	-0.035664
C	2.710029	0.154394	-0.970613
C	3.174983	-1.148343	-0.802012
C	2.773882	-1.884483	0.305435
C	1.917591	-1.347501	1.252314
F	3.216321	-3.137728	0.458813
C	1.909123	3.025805	1.063631
H	3.857253	-1.595183	-1.515351
H	0.921527	0.427457	1.900212
H	1.649001	-1.922470	2.129871
H	3.054197	0.714134	-1.829494
H	1.618029	2.599197	2.024900
H	3.000249	3.055094	1.035056
H	1.538941	4.052611	1.023320
H	1.441028	3.916239	-1.482141
H	2.944776	2.998250	-1.437840
H	1.553443	2.377804	-2.335141
H	-0.655962	1.714213	-2.266949
H	-3.190964	1.558142	-1.417795
H	-3.213573	2.020003	1.232235
H	-0.709546	2.492099	2.011933
H	-1.564719	-0.846752	-2.879578
H	-3.706901	-1.166093	-1.278049
H	-2.975391	-2.719300	0.786507
H	-0.386572	-3.348762	0.475599
H	0.488929	-2.181667	-1.786613
H	-2.911904	-0.589994	2.391615
H	-1.457547	-1.533243	2.680723
H	-1.484560	0.210310	3.026338

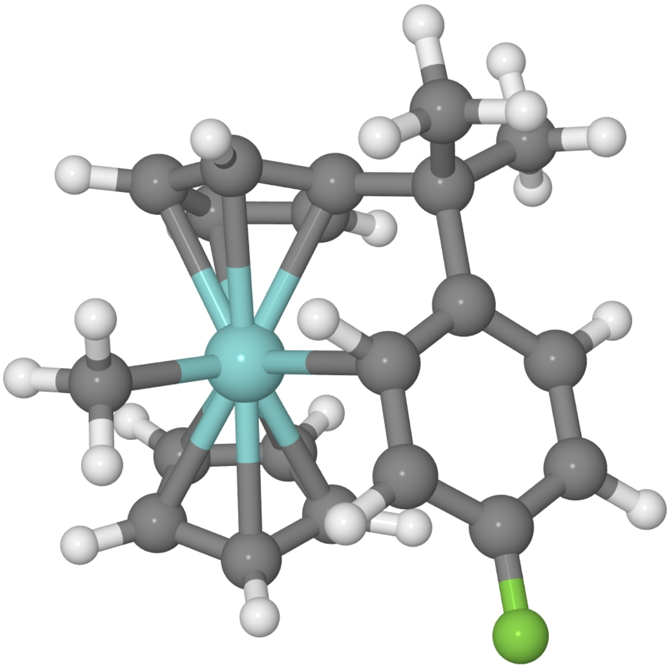

5B

5B: E(RB3LYP) = -922.334546662

C	2.088644	2.290648	-0.343814
C	2.972521	1.624391	0.535416
C	3.472276	0.476432	-0.133374
C	2.911096	0.449215	-1.441098
C	2.057681	1.569652	-1.567754
Zr	1.034310	0.005664	0.194955
C	0.588770	0.833641	2.256493
C	1.867228	-2.378976	0.014842
C	0.725468	-2.277180	-0.819376
C	-0.420033	-2.075504	0.008022
C	0.041211	-2.032619	1.353302
C	1.444106	-2.218414	1.356704
C	-1.861708	-1.832473	-0.442394
C	-2.074762	-2.244786	-1.915732
C	-1.254719	0.583483	-0.997654
C	-1.410564	1.975617	-0.895658
C	-2.372416	2.472081	-0.033593
C	-3.193531	1.620780	0.693714
C	-3.047432	0.241019	0.564806
C	-2.085222	-0.314664	-0.283573
F	-2.516230	3.796613	0.092938
C	-2.820430	-2.669110	0.427452
H	-3.945972	2.044600	1.347909
H	-3.708339	-0.400028	1.131446
H	-0.823895	2.659701	-1.494730
H	-0.619688	0.200122	-1.801652
H	-2.587888	-3.728387	0.306021
H	-2.741772	-2.433927	1.490028
H	-3.857406	-2.518895	0.118705
H	-1.881597	-3.312600	-2.039280
H	-3.107999	-2.050679	-2.210165
H	-1.429669	-1.703356	-2.610543
H	0.725470	-2.392266	-1.893145
H	2.874202	-2.585759	-0.314296
H	2.073987	-2.263039	2.233317
H	-0.568291	-1.894889	2.232112
H	-0.428983	0.536015	2.535599
H	1.252166	0.456538	3.042675
H	0.644822	1.925533	2.278183
H	4.205279	-0.213875	0.256977
H	3.229764	1.939259	1.534802
H	1.557530	3.205962	-0.128079
H	1.513383	1.849414	-2.459067
H	3.129461	-0.274929	-2.211861

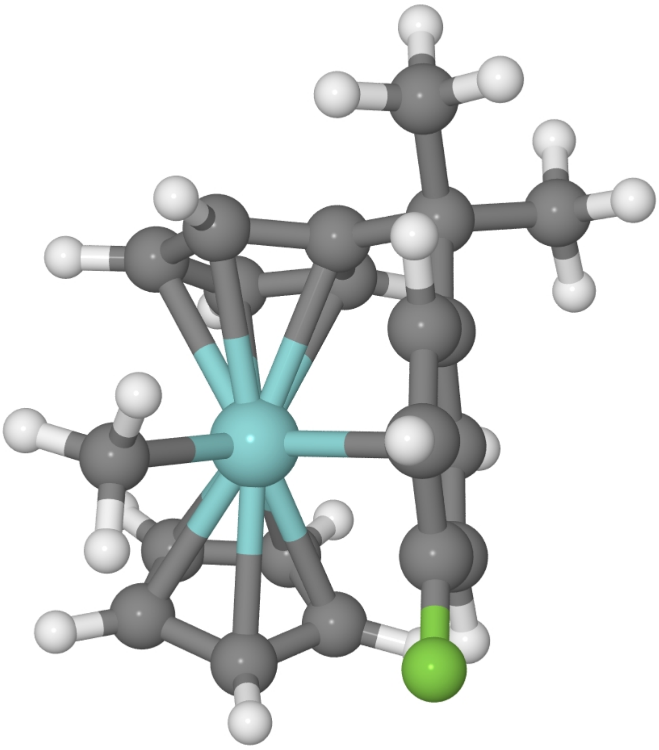

TS-5A-5B

TS-5A-5B: E(RB3LYP) = -922.329365877

Zr	-0.943368	-0.056704	0.202251
C	-0.884348	2.005460	-1.173882
C	0.092987	2.197032	-0.148621
C	-0.610000	2.309617	1.079735
C	-1.997233	2.189112	0.821917
C	-2.168371	2.016163	-0.571918
H	-3.112112	1.945614	-1.091148
H	-2.785095	2.257728	1.557414
H	-0.173693	2.464918	2.052606
C	1.606546	2.039430	-0.305910
C	1.858539	0.510672	-0.158746
C	1.714283	-0.363644	-1.259953
C	2.050170	-1.718907	-1.173057
C	2.489441	-2.225778	0.037277
C	2.583903	-1.419552	1.164816
C	2.270177	-0.067282	1.061328
H	2.391742	0.550038	1.940080
H	2.920624	-1.846147	2.101598
F	2.803617	-3.522490	0.130936
H	1.989362	-2.369200	-2.036863
H	1.431868	0.026913	-2.229179
C	2.354598	2.849359	0.770830
H	2.160949	3.913145	0.621835
H	2.053836	2.606240	1.790341
H	3.431512	2.686316	0.689670
C	2.079000	2.554778	-1.680920
H	1.897860	3.629164	-1.749185
H	3.150571	2.381095	-1.801313
H	1.568027	2.086947	-2.522865
H	-0.695221	1.917278	-2.233285
C	-3.155303	-1.138988	-0.291223
C	-2.469617	-1.091139	-1.538563
C	-1.365528	-1.966006	-1.459171
C	-1.356487	-2.553590	-0.164457
C	-2.478132	-2.061653	0.545491
H	-2.770858	-2.343242	1.544322
H	-0.647974	-3.284222	0.198630
H	-0.668691	-2.173311	-2.256693
H	-2.763934	-0.518146	-2.405069
H	-4.067678	-0.614612	-0.047596
C	-0.944926	-0.393708	2.454475
H	-0.231761	0.265167	2.957659
H	-1.922763	-0.209711	2.912537
H	-0.672623	-1.429676	2.681369

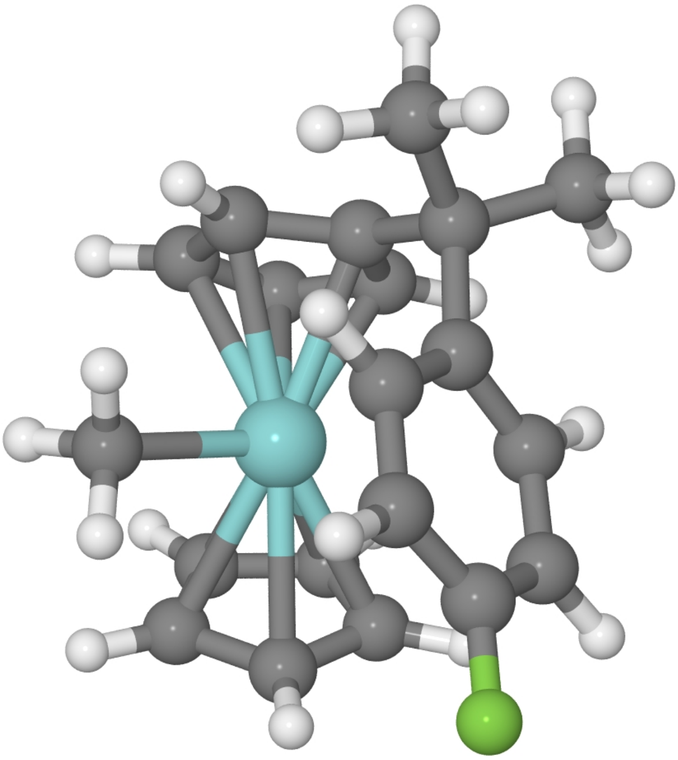

6A

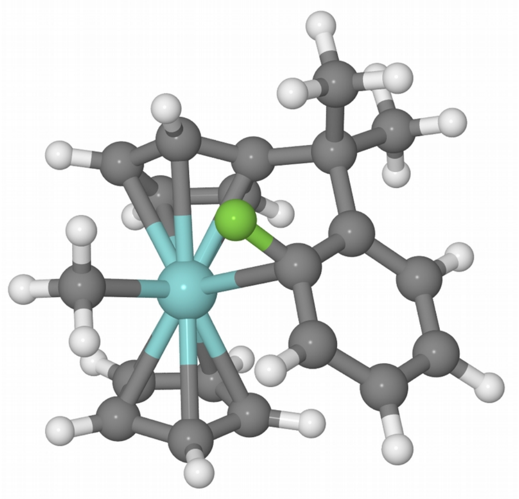

6A: E(RB3LYP) = -4415.91700676

Zr	0.000000	0.000000	0.000000	0.890746
C	0.000000	0.000000	2.550083	-0.103031
C	1.351538	0.000000	2.116554	-0.179293
C	1.613248	1.252019	1.491447	-0.126975
C	0.419480	2.006032	1.515289	-0.161923
C	-0.580125	1.230351	2.165912	-0.161565
H	-1.594285	1.542576	2.367229	0.132794
H	0.293562	3.006015	1.126021	0.139023
H	2.563602	1.577709	1.095995	0.139245
H	2.073068	-0.783242	2.296456	0.143872
H	-0.492974	-0.794871	3.087179	0.153917
C	-1.543952	-1.587828	0.512276	-0.576513
H	-1.125865	-2.429005	1.076358	0.154159
H	-2.330497	-1.150963	1.139014	0.148042
H	-2.010236	-2.013912	-0.381811	0.156294
C	0.685441	-0.011885	-2.441296	-0.102859
C	0.152359	-1.308281	-2.194763	-0.106338
C	0.993696	-1.981006	-1.276997	-0.086357
C	2.051534	-1.105601	-0.934937	-0.129691
C	1.861353	0.111051	-1.646227	-0.140743
H	2.527659	0.959590	-1.619946	0.128994
H	2.890611	-1.340591	-0.297414	0.140551
H	0.859939	-2.991204	-0.920581	0.146775
H	-0.732655	-1.730339	-2.645347	0.138653
C	0.040292	1.110118	-3.256446	-0.201959
C	1.130838	1.925228	-3.976708	-0.251143
H	1.701128	1.266928	-4.634013	0.147806
H	0.686358	2.707316	-4.595941	0.137478
H	1.834007	2.396552	-3.287417	0.115899
C	-0.760719	1.960906	-2.242706	-0.020133
C	-1.773977	1.348873	-1.463431	0.003881
C	-2.574199	2.083978	-0.576264	-0.023568
C	-2.350516	3.445443	-0.455669	-0.076371
C	-1.357943	4.079703	-1.191695	-0.071662
C	-0.567965	3.338567	-2.068271	-0.058765
H	0.183383	3.859327	-2.645957	0.137214
H	-1.220299	5.148921	-1.082899	0.139662
H	-2.958047	4.023603	0.229551	0.138048
H	-3.379647	1.614979	-0.024783	0.163273
F	-2.132337	0.084520	-1.714895	-0.190681
C	-0.921471	0.545622	-4.326388	-0.230138
H	-1.735782	-0.041811	-3.899084	0.130170
H	-1.370111	1.364103	-4.892993	0.133883
H	-0.373975	-0.092340	-5.023699	0.139258

6B

6B: E(RB3LYP) = -4415.91738318

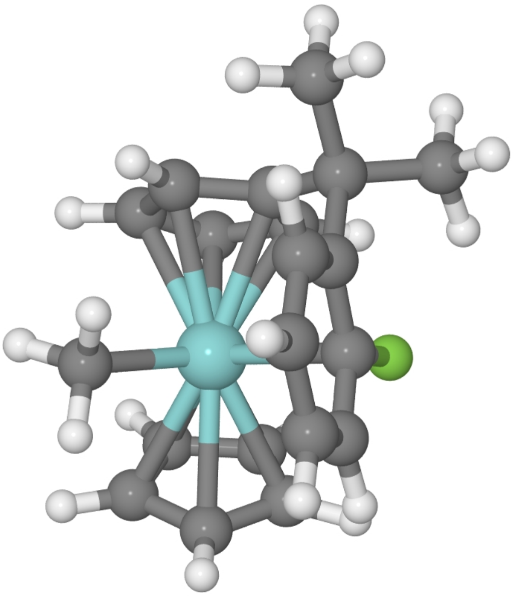

Zr	0.000000	0.000000	0.000000	0.930086
C	0.000000	0.000000	2.548108	-0.107906
C	1.365221	0.000000	2.181563	-0.151860
C	1.648443	-1.233872	1.536452	-0.172063
C	0.455284	-1.991513	1.490747	-0.126298
C	-0.570795	-1.223426	2.109626	-0.175960
H	-1.587677	-1.544111	2.280810	0.146368
H	0.350333	-2.990308	1.093837	0.144214
H	2.615972	-1.560299	1.181065	0.142473
H	2.072222	0.789944	2.387866	0.135633
H	-0.514417	0.787008	3.077335	0.153636
C	-0.510963	2.158464	0.462536	-0.576467
H	-0.425644	2.739563	-0.463395	0.142243
H	-1.531448	2.306871	0.832630	0.158848
H	0.170105	2.589284	1.201712	0.146734
C	-1.830096	-1.497904	-0.905390	-0.108248
C	-0.749700	-1.568046	-1.820662	-0.133985
C	-0.612373	-0.295912	-2.453315	-0.111070
C	-1.606681	0.555285	-1.895480	-0.102223
C	-2.352875	-0.182433	-0.945947	-0.118339
H	-3.194537	0.186744	-0.378051	0.143083
H	-1.780289	1.587614	-2.154180	0.135238
C	0.460552	0.133500	-3.455713	-0.215697
C	1.179441	-1.084642	-4.076558	-0.236168
H	0.465722	-1.703664	-4.624481	0.137616
H	1.944266	-0.746643	-4.778517	0.135554
H	1.672660	-1.715923	-3.334753	0.126421
C	1.462629	0.976032	-2.640295	0.022739
C	2.120321	0.385615	-1.533423	-0.038648
C	3.044198	1.100891	-0.753962	-0.030035
C	3.273191	2.431949	-1.055022	-0.056810
C	2.634148	3.047159	-2.123226	-0.058843
C	1.739105	2.320438	-2.905836	-0.067245
H	1.265236	2.818744	-3.740078	0.133737
H	2.848903	4.086927	-2.338920	0.137607
H	3.939515	2.976035	-0.474137	0.133183
H	3.599036	0.629780	0.046818	0.149640
F	2.065314	-0.941458	-1.371880	-0.198494
C	-0.194531	0.931432	-4.600226	-0.246080
H	-0.921367	0.298832	-5.112492	0.144714
H	-0.720707	1.820452	-4.249017	0.118971
H	0.551749	1.242299	-5.334825	0.136593
H	-0.172485	-2.454692	-2.037240	0.136455
H	-2.212407	-2.316502	-0.314778	0.140787

***ortho tert*-butyl-fluorobenzene**

ortho tert-butyl-fluorobenzene: E(RB3LYP) = -488.864905009

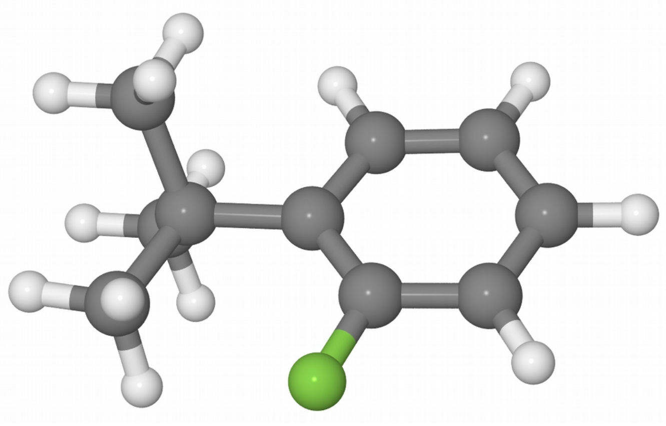

C	1.507474	-0.173016	0.000002
C	-0.037342	-0.174087	-0.000036
C	-0.712345	-1.408998	-0.000025
C	-2.097064	-1.517749	-0.000011
C	-2.882849	-0.367509	-0.000008
C	-2.260045	0.871906	0.000001
C	-0.871550	0.946987	0.000000
F	-0.357670	2.205213	0.000010
H	-2.822659	1.797431	0.000011
H	-3.964488	-0.432627	-0.000005
H	-2.559544	-2.497876	-0.000004
H	-0.126639	-2.320245	-0.000013
C	2.150392	1.229138	0.000372
H	1.873975	1.807462	0.882728
H	3.238013	1.112005	-0.000229
H	1.873091	1.808309	-0.881164
C	2.006912	-0.915099	1.262652
H	1.670030	-0.407461	2.170379
H	1.650779	-1.946715	1.303808
H	3.100703	-0.939090	1.273917
C	2.007040	-0.914455	-1.262978
H	3.100836	-0.938164	-1.274286
H	1.651189	-1.946148	-1.304569
H	1.670006	-0.406517	-2.170480

meta tert-butyl-fluorobenzene

meta tert-butyl-fluorobenzene E(RB3LYP) = -488.869396409

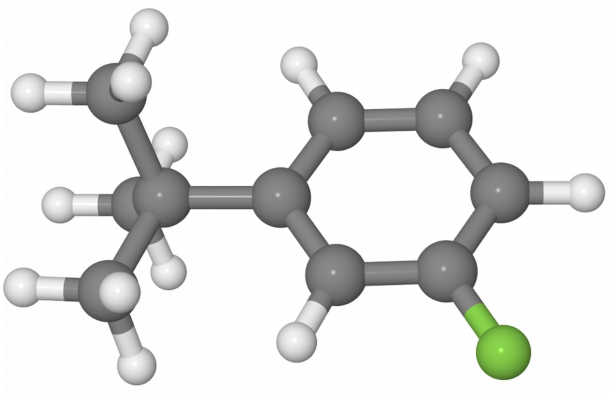

C	-1.749064	-0.162119	0.000000
C	-0.243516	0.155330	-0.000015
C	0.193137	1.490300	-0.000011
C	1.546238	1.809768	-0.000004
C	2.515195	0.807386	-0.000003
C	2.077208	-0.504048	0.000000
C	0.731721	-0.846033	0.000000
H	0.477989	-1.896931	0.000003
F	2.992330	-1.502735	0.000006
H	3.575370	1.026219	0.000000
H	1.854029	2.849325	0.000000
H	-0.532463	2.294675	-0.000003
C	-2.027698	-1.676166	0.000453
H	-1.615749	-2.165733	0.887082
H	-3.106827	-1.850225	0.000134
H	-1.615106	-2.166377	-0.885523
C	-2.400831	0.449458	1.262145
H	-1.961137	0.029202	2.170676
H	-2.273910	1.533613	1.300284
H	-3.474275	0.237816	1.273878
C	-2.400720	0.448681	-1.262582
H	-3.474130	0.236867	-1.274374
H	-2.273967	1.532833	-1.301292
H	-1.960818	0.027998	-2.170815

para tert-butyl-fluorobenzene

para tert-butyl-fluorobenzene E(RB3LYP) = -488.868543817

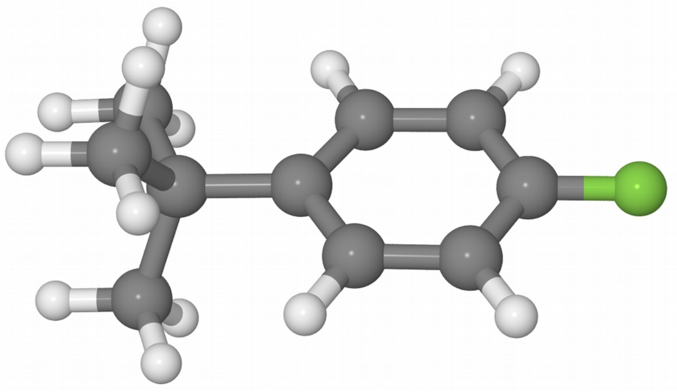

C	1.875650	0.003367	0.000000
C	0.336379	0.029903	0.000025
C	-0.384898	-1.174557	0.000029
C	-1.774948	-1.200272	0.000013
C	-2.463671	0.003119	0.000000
C	-1.798266	1.214975	-0.000005
C	-0.402862	1.217592	0.000002
H	0.102020	2.174135	0.000010
H	-2.364906	2.138017	-0.000011
F	-3.817048	-0.011613	-0.000006
H	-2.325621	-2.132946	0.000013
H	0.142927	-2.121005	0.000029
C	2.484176	1.417765	-0.000575
H	2.191265	1.987956	0.885269
H	3.574908	1.345696	0.000017
H	2.192182	1.986858	-0.887422
C	2.378919	-0.734938	1.262032
H	2.040543	-0.229287	2.170424
H	2.019342	-1.765731	1.300364
H	3.472803	-0.763296	1.275479
C	2.378808	-0.735926	-1.261497
H	3.472687	-0.764543	-1.274883
H	2.018981	-1.766661	-1.299116
H	2.040578	-0.230836	-2.170256

tert-butyl-benzene

tert-butyl-benzene: E(RB3LYP) = -389.604190352

C	1.461022	0.005490	0.000000
C	-0.078587	0.028710	-0.000006
C	-0.796437	-1.177655	-0.000005
C	-2.186951	-1.197933	-0.000002
C	-2.906717	-0.004339	0.000000
C	-2.214931	1.201253	0.000002
C	-0.819420	1.215505	0.000000
H	-0.313823	2.172184	0.000000
H	-2.758183	2.140138	0.000003
H	-3.990838	-0.016753	0.000001
H	-2.709700	-2.148364	-0.000002
H	-0.263937	-2.122093	-0.000004
C	2.069243	1.420056	0.000125
H	1.776039	1.989420	0.886341
H	3.160166	1.348386	0.000005
H	1.775852	1.989653	-0.885881
C	1.964964	-0.733104	1.261717
H	1.625770	-0.228002	2.170177
H	1.605849	-1.763957	1.299317
H	3.059019	-0.760009	1.275261
C	1.964988	-0.732889	-1.261833
H	3.059045	-0.759747	-1.275385
H	1.605923	-1.763753	-1.299592
H	1.625771	-0.227661	-2.170215

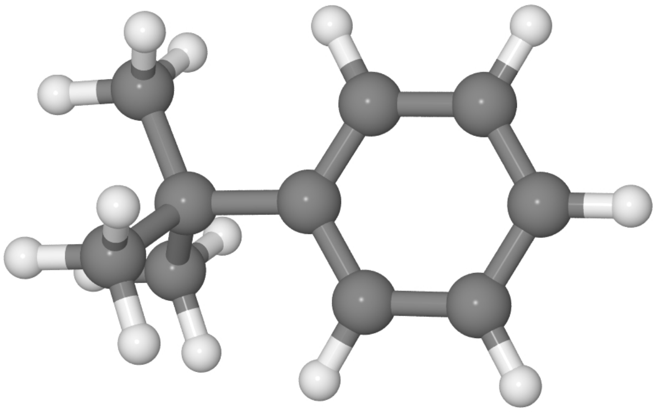

PBE-D3 calculations

Cartesian Coordinates

IIA

IIA-PBE-D3: E= -822.1020039776

C	-2.918575	0.148642	1.169530
C	-1.813009	0.670568	1.912683
C	-1.313481	1.798802	1.208356
C	-2.114769	1.985833	0.042935
C	-3.110303	0.974990	0.024234
Zr	-0.895410	-0.217654	-0.233487
C	-1.826146	-0.392782	-2.294322
C	1.272028	0.877322	-1.135045
C	2.038980	0.513030	0.011594
C	2.423740	1.542635	0.886720
C	2.111955	2.880327	0.604511
C	1.384166	3.229334	-0.540900
C	0.960167	2.224093	-1.413623
C	2.425202	-0.969697	0.207877
C	3.130375	-1.224971	1.553068
C	3.367928	-1.411560	-0.933177
C	1.099429	-1.728574	0.165657
C	0.434845	-2.279762	-0.977248
C	-0.848252	-2.737753	-0.574767
C	-0.998063	-2.453711	0.811379
C	0.204687	-1.839013	1.271122
H	1.161602	4.276144	-0.754999
H	-0.456868	2.406805	1.494642
H	-1.580580	-3.225063	-1.214598
H	3.014983	1.310338	1.773029
H	-1.436849	0.284979	2.857930
H	-3.541877	-0.695395	1.458528
H	0.841652	-2.355945	-1.983690
H	0.406073	-1.534905	2.295289
H	-3.887172	0.854180	-0.726658
H	-1.989758	2.772413	-0.698912
H	-1.847500	-2.734284	1.430800
H	1.099665	0.118269	-1.912576
H	0.418376	2.471070	-2.329536
H	2.455562	3.662777	1.285214
H	2.543671	-0.876832	2.415431
H	2.939423	-1.237799	-1.930581
H	3.594809	-2.483840	-0.837954
H	4.313048	-0.852081	-0.876022
H	4.110774	-0.726438	1.583860
H	3.301868	-2.303539	1.675745
H	-1.147475	-0.915242	-2.989608

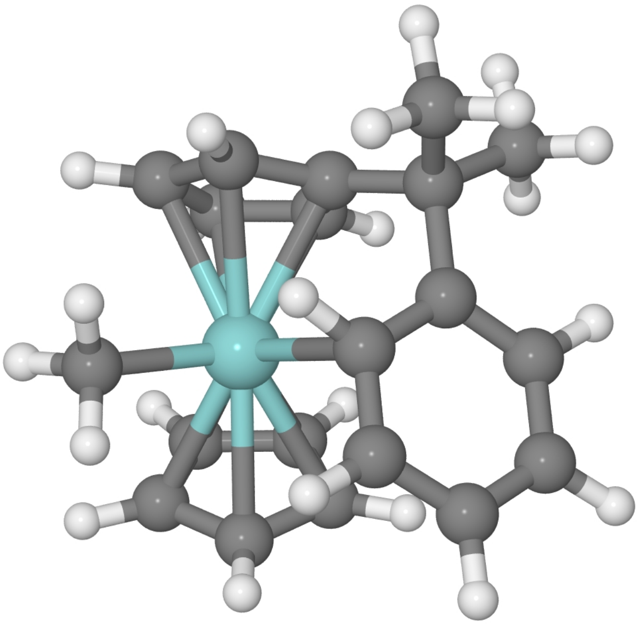

H	-2.782775	-0.942210	-2.319262
H	-2.034595	0.618074	-2.688140

IIB

IIB-PBE-D3: E= -822.0976287468

C	3.150589	-0.908837	-0.651254
C	3.330493	0.277796	0.118006
C	2.787058	0.050129	1.420854
C	2.274555	-1.275716	1.448252
C	2.490357	-1.863317	0.168172
Zr	0.875360	0.073337	-0.202495
C	0.587733	-0.731078	-2.288852
C	-0.038035	2.113886	0.976567
C	-1.059684	1.709018	0.056558
C	-0.534096	1.876858	-1.261394
C	0.788005	2.390003	-1.151953
C	1.091957	2.544185	0.230537
C	-2.409914	1.091817	0.418530
C	-3.504884	1.658491	-0.503845
C	-1.127499	-1.051751	0.930847
C	-2.211000	-0.424981	0.251139
C	-3.016171	-1.234140	-0.560121
C	-2.768086	-2.609279	-0.678328
C	-1.713746	-3.219626	0.011046
C	-0.899605	-2.441079	0.835197
C	-2.790546	1.400178	1.884125
H	2.010881	2.956112	0.641703
H	1.441077	2.647267	-1.984617
H	-1.050569	1.665129	-2.194092
H	-3.858219	-0.798366	-1.098589
H	-3.419229	-3.214425	-1.313324
H	-1.544147	-4.293905	-0.076032
H	-0.107714	-2.908130	1.422492
H	-0.599950	-0.482281	1.722243
H	-2.065112	0.997387	2.606311
H	-3.768277	0.955250	2.119207
H	-2.860411	2.487525	2.037647
H	-3.323173	1.429373	-1.563875
H	-3.541593	2.751902	-0.397720
H	-4.493952	1.262791	-0.229153
H	-0.119866	2.126126	2.061796
H	3.471292	-1.061748	-1.678926
H	2.208601	-2.871301	-0.131404
H	1.817959	-1.759513	2.310938
H	2.794807	0.751071	2.252967
H	3.837692	1.181838	-0.213674
H	-0.515255	-0.772955	-2.397023
H	0.964473	-0.129409	-3.131627
H	0.984137	-1.752896	-2.396251

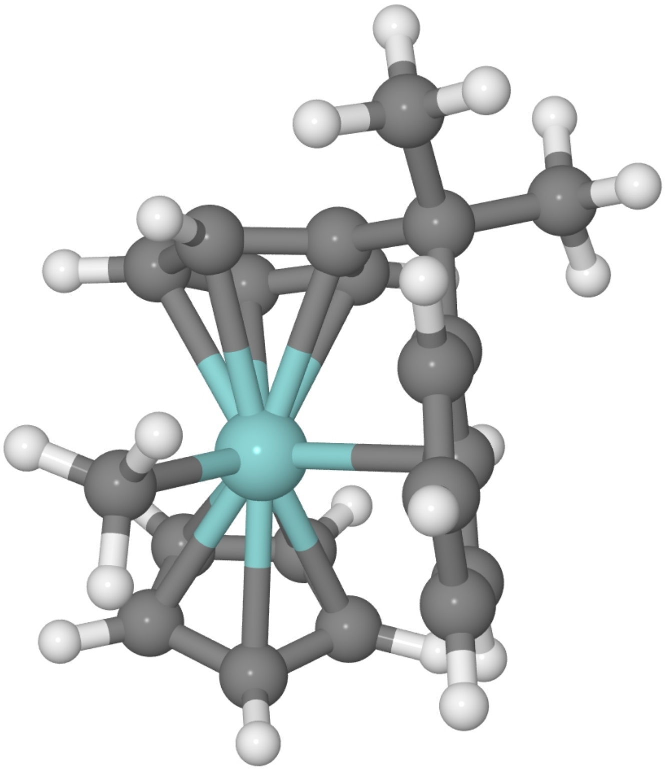

TS-IIA-IIB

TS-IIA-II-PBE-D3: E= -822.0944046621

C	-3.045878	0.781461	-0.509140
C	-3.164492	-0.400679	0.273564
C	-2.550790	-0.149732	1.540298
C	-2.046992	1.175470	1.529871
C	-2.335418	1.746650	0.254904
Zr	-0.712005	-0.153477	-0.178691
C	-0.992640	0.093002	-2.427173
C	0.257063	-1.949593	1.166591
C	1.258612	-1.654316	0.178054
C	0.734150	-2.050015	-1.085294
C	-0.562369	-2.596094	-0.887347
C	-0.846830	-2.562305	0.502765
C	2.504745	-0.793623	0.391308
C	3.068769	-0.950549	1.815959
C	1.958039	0.633915	0.138626
C	1.325765	1.374296	1.172574
C	0.872304	2.691422	0.949966
C	0.963544	3.269673	-0.316260
C	1.491071	2.510706	-1.370556
C	1.985315	1.218719	-1.149886
C	3.618191	-1.174086	-0.603093
H	-1.738866	-2.960393	0.981039
H	-1.207360	-3.001414	-1.664172
H	1.225933	-1.946717	-2.048828
H	2.432012	0.681605	-1.986706
H	1.545372	2.935557	-2.375264
H	0.619747	4.291245	-0.486336
H	0.450729	3.257441	1.783355
H	1.289192	0.971052	2.186633
H	3.990058	-2.180777	-0.365192
H	3.285373	-1.191684	-1.649545
H	4.458662	-0.468384	-0.526163
H	3.423239	-1.981445	1.957118
H	3.920888	-0.270635	1.964102
H	2.335034	-0.748063	2.607894
H	0.347511	-1.804023	2.241291
H	-3.443694	0.923601	-1.510306
H	-2.080817	2.753937	-0.069836
H	-1.549506	1.668034	2.362668
H	-2.508210	-0.838865	2.381216
H	-3.670685	-1.316659	-0.024644
H	-0.064870	-0.167980	-2.961038
H	-1.797911	-0.521914	-2.860442
H	-1.233906	1.148902	-2.639376

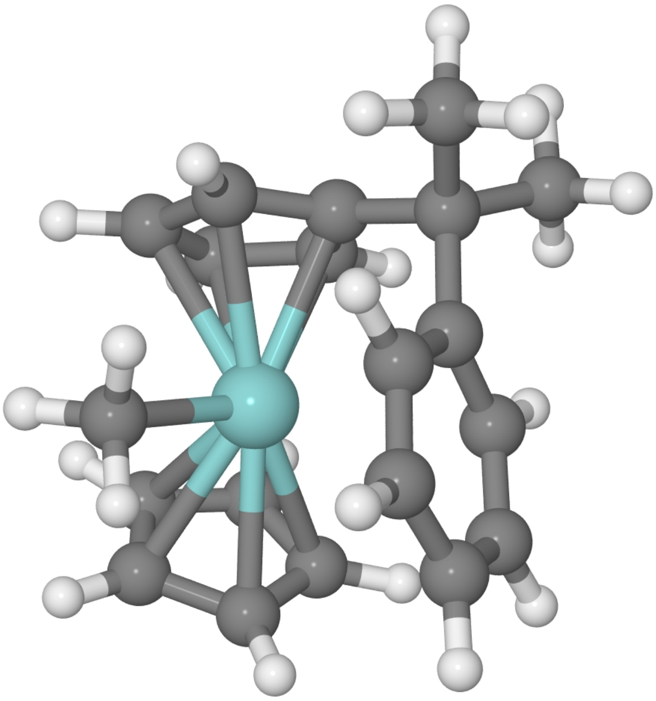

1A

1A-PBE-D3: E= -921.2826502301

C	-3.380820	-0.631938	-0.921501
C	-2.325437	-1.367510	-1.549773
C	-1.776035	-2.251885	-0.587466
C	-2.489771	-2.072312	0.634909
C	-3.493570	-1.086659	0.425104
Zr	-1.284831	0.105452	0.198211
C	-2.163375	0.971580	2.090947
F	0.578370	-0.953091	1.003440
C	1.888427	-1.141654	0.409591
C	2.597459	-0.010331	0.012837
C	3.843549	-0.304742	-0.574959
C	4.306805	-1.617447	-0.719357
C	3.539570	-2.697633	-0.275068
C	2.287245	-2.459048	0.305782
C	2.089548	1.417892	0.264298
C	2.980150	2.474487	-0.433301
C	2.107718	1.683841	1.789130
C	0.693533	1.594412	-0.320290
C	0.221371	1.031706	-1.551538
C	-1.075419	1.556361	-1.815589
C	-1.422048	2.425281	-0.742976
C	-0.329958	2.459422	0.168235
H	1.650778	-3.265241	0.672637
H	3.901819	-3.721873	-0.374897
H	5.281026	-1.794121	-1.178231
H	4.467803	0.519426	-0.920232
H	1.437727	1.007191	2.340091
H	3.125547	1.538936	2.178815
H	1.815247	2.721468	2.007676
H	2.555162	3.473571	-0.262706
H	4.000067	2.470301	-0.020572
H	3.031386	2.311482	-1.519969
H	0.773097	0.340366	-2.188539
H	-1.683012	1.347447	-2.693502
H	-2.350010	2.986373	-0.642765
H	-0.295537	3.046766	1.081620
H	-2.011893	-1.277738	-2.587722
H	-4.019284	0.106079	-1.404111
H	-4.221624	-0.746151	1.156873
H	-2.309053	-2.609075	1.565660
H	-0.951662	-2.944435	-0.754368
H	-3.059890	1.600515	1.953071
H	-2.448112	0.155990	2.777663
H	-1.410113	1.604069	2.591986

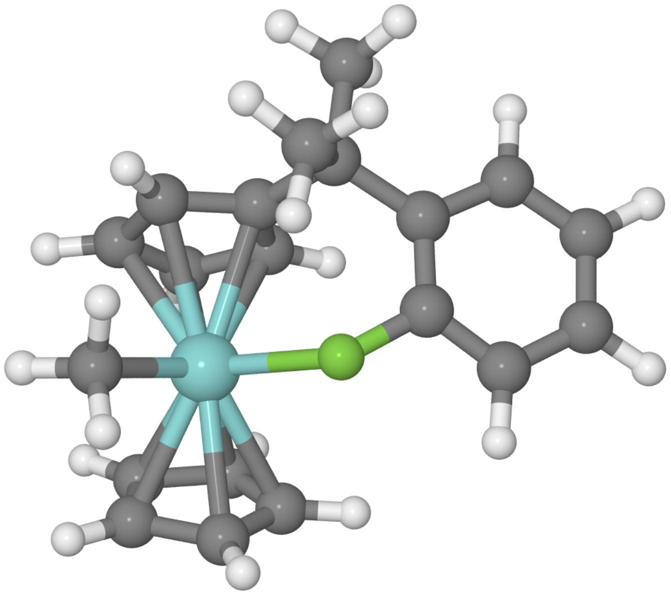

1B

1B-PBD-D3: E= -921.2748693815

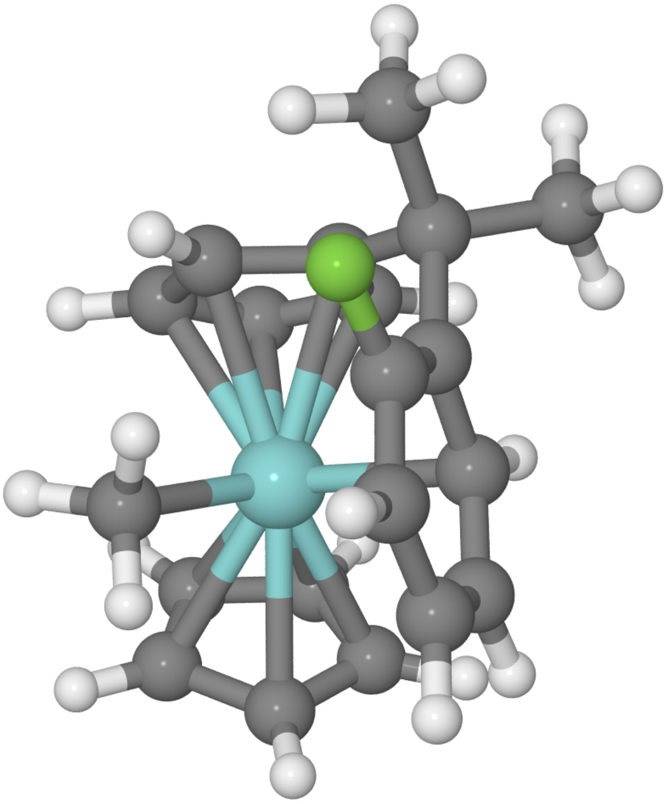

C	0.789411	-2.313459	-1.130099
C	0.990817	-0.921395	-1.215395
C	1.998036	-0.237171	-0.470747
C	2.736938	-1.045475	0.408797
C	2.542202	-2.426845	0.516848
C	1.574297	-3.070089	-0.254205
Zr	-0.989802	0.021243	0.138820
C	-0.382810	2.171155	-0.957162
C	0.755934	1.811207	-0.158331
C	0.349822	1.883440	1.206903
C	-1.012133	2.288281	1.251730
C	-1.458142	2.492391	-0.083497
C	2.110924	1.296444	-0.644858
C	3.235678	1.972932	0.167103
F	3.678077	-0.508048	1.212652
C	-3.488436	0.047376	0.303015
C	-3.189325	0.031485	-1.093539
C	-2.581157	-1.217363	-1.388872
C	-2.513365	-1.977587	-0.184177
C	-3.079792	-1.198542	0.861829
C	-0.434264	-0.839064	2.157338
C	2.348568	1.628005	-2.135349
H	3.170571	-2.978252	1.219114
H	1.439709	-4.149676	-0.174865
H	0.044382	-2.792523	-1.766487
H	0.465874	-0.355203	-2.004363
H	3.189696	3.056378	-0.014832
H	3.148473	1.804551	1.246450
H	4.221957	1.611385	-0.154079
H	2.368825	2.719089	-2.274012
H	3.319038	1.223568	-2.456813
H	1.579825	1.218593	-2.807203
H	-0.409582	2.247120	-2.043312
H	-2.441748	2.843770	-0.386035
H	-1.597687	2.451589	2.155040
H	0.972140	1.675900	2.072922
H	0.636906	-0.655896	2.347968
H	-0.985532	-0.424722	3.018100
H	-0.604999	-1.929003	2.148904
H	-3.983713	0.854069	0.840587
H	-3.197510	-1.504011	1.898187
H	-2.115788	-2.985398	-0.079053
H	-2.255151	-1.547383	-2.375122
H	-3.402071	0.824564	-1.807147

TS-1A-1B

TS-1A-1B: E= -921.2718947674

C	3.041505	-1.079328	-0.649095
C	3.332812	0.103680	0.089217
C	2.813699	-0.056057	1.412526
C	2.193615	-1.329675	1.479264
C	2.311829	-1.953604	0.201445
Zr	0.863991	0.112110	-0.161770
C	0.728501	-0.502928	-2.349586
C	0.291548	2.185294	1.013429
C	-0.874731	1.830120	0.257037
C	-0.541678	1.979256	-1.123374
C	0.801778	2.427906	-1.218304
C	1.318090	2.567977	0.099251
C	-2.121156	1.117856	0.786733
C	-2.280040	1.324295	2.306654
C	-1.820314	-0.374471	0.457224
C	-1.121818	-1.192376	1.391128
C	-0.915464	-2.569938	1.184472
C	-1.367963	-3.178161	0.014665
C	-1.982618	-2.393088	-0.966161
C	-2.175213	-1.028869	-0.749613
F	-2.738254	-0.332336	-1.763762
C	-3.406791	1.649838	0.118077
H	2.304780	2.936693	0.369363
H	1.335519	2.642037	-2.142191
H	-1.197426	1.763170	-1.962748
H	-2.323393	-2.816481	-1.912749
H	-1.237009	-4.249328	-0.146610
H	-0.414129	-3.157010	1.955726
H	-0.845053	-0.763360	2.356349
H	-3.591314	2.673102	0.475649
H	-3.350944	1.680358	-0.974252
H	-4.270476	1.029431	0.400788
H	-2.495831	2.383782	2.504910
H	-3.121202	0.726008	2.685495
H	-1.386077	1.064043	2.890318
H	0.373837	2.214106	2.098036
H	3.323716	-1.274981	-1.680019
H	1.931994	-2.936575	-0.071435
H	1.726330	-1.758089	2.363865
H	2.907976	0.656949	2.229001
H	3.909504	0.951988	-0.274510
H	-0.254755	-0.240436	-2.772126
H	1.486660	-0.041688	-3.004450
H	0.860056	-1.596534	-2.419073

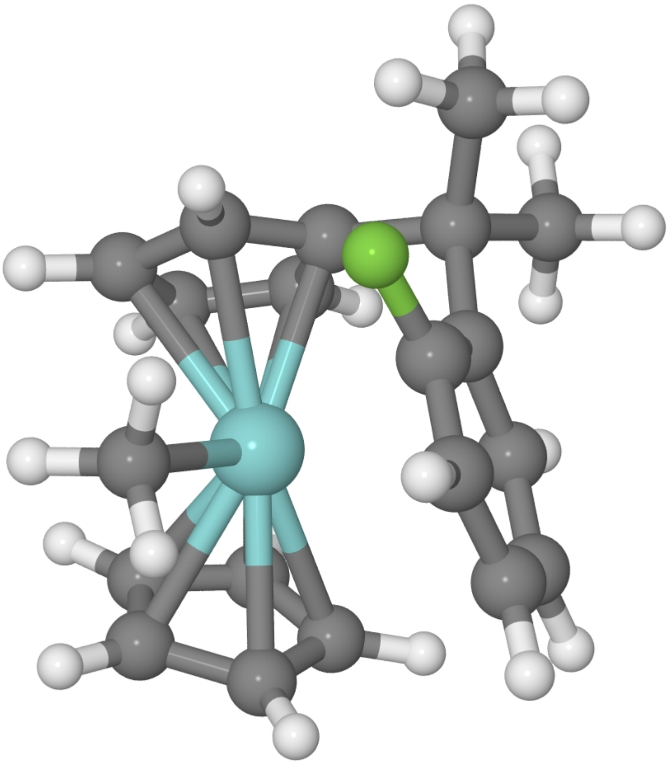

2A

2A-PBE-D3: E= -921.2830002556

C	-3.022320	0.122730	1.149819
C	-1.947780	0.402457	2.049271
C	-1.286656	1.573211	1.591688
C	-1.944589	2.015321	0.405243
C	-3.020967	1.124876	0.138527
Zr	-0.901887	-0.209411	-0.144311
C	-1.680309	0.035901	-2.259307
C	1.393885	0.781976	-0.899820
C	2.035610	0.133720	0.198571
C	2.441598	0.935288	1.277964
C	2.290579	2.330925	1.239884
C	1.697017	2.974590	0.150855
C	1.256764	2.181291	-0.906162
C	2.262559	-1.395356	0.129281
C	2.947822	-1.943748	1.394726
F	0.694345	2.759924	-1.982172
C	3.140580	-1.736048	-1.093187
C	0.857564	-1.985825	-0.004176
C	-0.048593	-2.140362	1.092473
C	-1.309724	-2.552549	0.569760
C	-1.193190	-2.646429	-0.844054
C	0.139428	-2.299266	-1.199292
H	2.654097	2.930249	2.077612
H	1.183038	0.241555	-1.830969
H	1.588313	4.058869	0.103232
H	2.932641	0.481001	2.138142
H	2.707782	-1.384024	-2.040861
H	4.131912	-1.272528	-0.987421
H	3.271737	-2.825756	-1.164823
H	3.014021	-3.038678	1.327112
H	3.970756	-1.549070	1.484651
H	2.403038	-1.698763	2.317589
H	0.186304	-2.003493	2.145342
H	-2.190122	-2.807988	1.155597
H	-1.976905	-2.947655	-1.535771
H	0.534248	-2.278105	-2.212661
H	-1.700856	-0.163578	2.945362
H	-3.741804	-0.688626	1.240484
H	-3.727361	1.204965	-0.683712
H	-1.681937	2.892464	-0.184474
H	-0.426734	2.046445	2.062993
H	-2.686028	-0.371894	-2.453682
H	-1.724503	1.119450	-2.471441
H	-1.001331	-0.437954	-2.988226

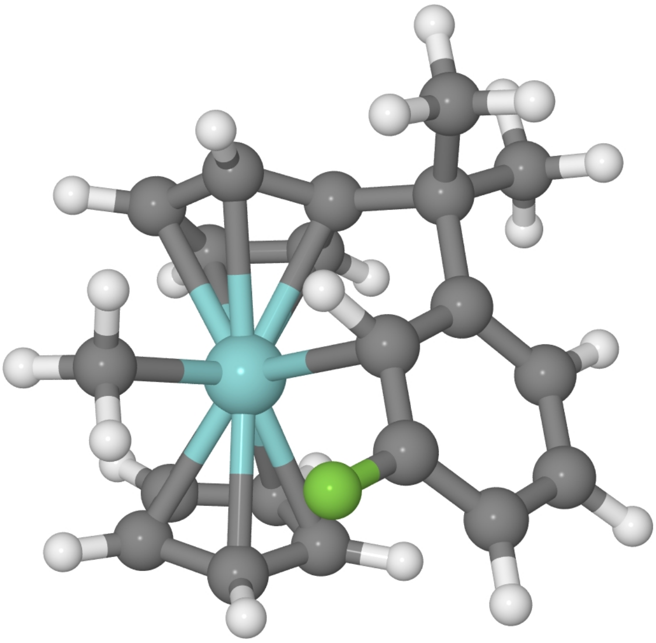

2B

2B-PBE-D3; E= -921.2807106580

C	2.817573	-1.823285	-0.657272
C	3.462060	-0.652508	-0.163253
C	3.093760	-0.489325	1.208798
C	2.232258	-1.563523	1.560330
C	2.050946	-2.377438	0.404768
Zr	1.025046	-0.123971	-0.153076
C	0.245744	-1.093256	-2.040573
C	-1.104331	-0.462773	1.191955
C	-1.304754	-1.862134	1.160524
C	-2.321809	-2.410838	0.383723
C	-3.152496	-1.529172	-0.313683
C	-3.013442	-0.137143	-0.257643
C	-1.988093	0.423418	0.505563
C	-1.699453	1.933099	0.556371
C	-1.754544	2.438327	2.014413
F	-4.131553	-2.033812	-1.074524
C	0.935530	2.241127	0.689564
C	-0.291180	2.047274	-0.028061
C	0.044932	1.853584	-1.402389
C	1.457009	1.937542	-1.536232
C	2.005455	2.186810	-0.246086
C	-2.686353	2.761691	-0.286418
H	-3.719497	0.475776	-0.817918
H	-2.495932	-3.485351	0.323156
H	-0.666496	-2.514239	1.758956
H	-0.409340	-0.042692	1.942195
H	-2.393995	3.820472	-0.253465
H	-2.702958	2.454614	-1.342045
H	-3.707685	2.680182	0.114748
H	-1.486895	3.504569	2.050952
H	-2.772432	2.325100	2.414092
H	-1.073391	1.893647	2.684579
H	1.036682	2.436156	1.755670
H	3.056964	2.346132	-0.017612
H	2.015055	1.851474	-2.467054
H	-0.653895	1.678943	-2.216441
H	-0.793027	-0.741872	-2.178156
H	0.789661	-0.858998	-2.970763
H	0.229436	-2.190161	-1.932853
H	4.146808	-0.016479	-0.721119
H	2.907063	-2.229416	-1.661909
H	1.448913	-3.282422	0.345044
H	1.802192	-1.738527	2.546376
H	3.449419	0.292271	1.877573

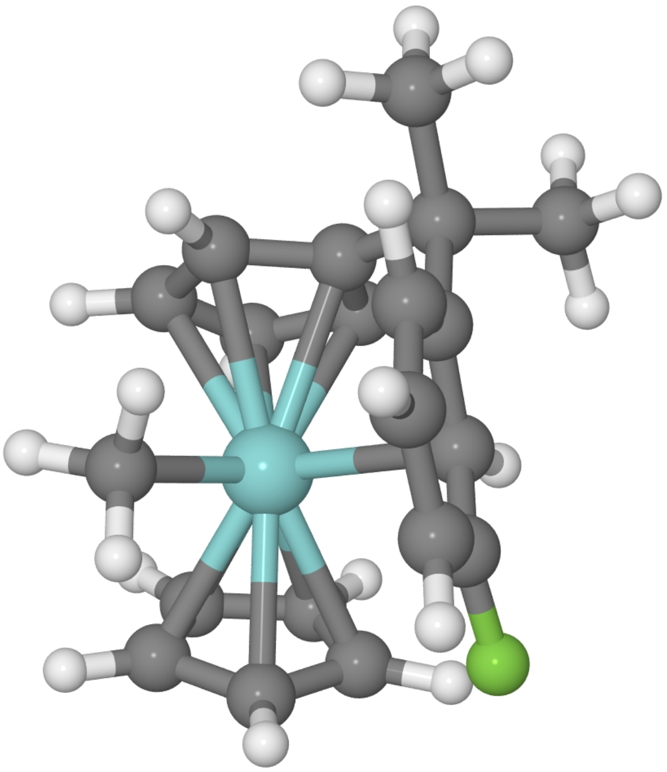

TS-2A-2B

TS-2A-2B: E= -921.2756358525

C	2.594600	-1.778787	-0.534800
C	3.241578	-0.628904	-0.002684
C	2.797355	-0.450374	1.343549
C	1.864941	-1.479134	1.630703
C	1.724197	-2.292886	0.466541
Zr	0.788313	-0.039718	-0.138002
C	0.582885	-0.733111	-2.310205
C	0.721100	2.211621	0.865663
C	-0.408102	2.147299	-0.022412
C	0.100377	2.082185	-1.350885
C	1.520117	2.110520	-1.292742
C	1.901514	2.223377	0.070504
C	-1.859567	1.873097	0.376085
C	-2.218414	2.527879	1.723312
C	-1.874325	0.327385	0.464945
C	-1.404021	-0.351321	1.622983
C	-1.473407	-1.758695	1.715533
C	-1.910133	-2.533834	0.643749
C	-2.254517	-1.862503	-0.533580
C	-2.248503	-0.466357	-0.641145
F	-2.617528	-2.572146	-1.610229
C	-2.836792	2.401587	-0.689108
H	2.918985	2.317312	0.442098
H	2.193255	2.092875	-2.147442
H	-0.484612	2.015405	-2.263328
H	-2.594603	-0.028331	-1.576591
H	-1.975872	-3.621193	0.694385
H	-1.180276	-2.245468	2.647893
H	-1.115940	0.214437	2.510026
H	-2.789097	3.499417	-0.713242
H	-2.612088	2.047178	-1.704210
H	-3.867873	2.106165	-0.445283
H	-2.172292	3.621455	1.620660
H	-3.241214	2.250860	2.017724
H	-1.542762	2.249535	2.543863
H	0.687562	2.294959	1.950116
H	2.747397	-2.192975	-1.527407
H	1.089296	-3.172041	0.372174
H	1.366529	-1.631559	2.585797
H	3.132904	0.319174	2.035444
H	3.975055	-0.015943	-0.522774
H	-0.288993	-0.268158	-2.797768
H	1.459759	-0.490488	-2.932572
H	0.450488	-1.828288	-2.346345

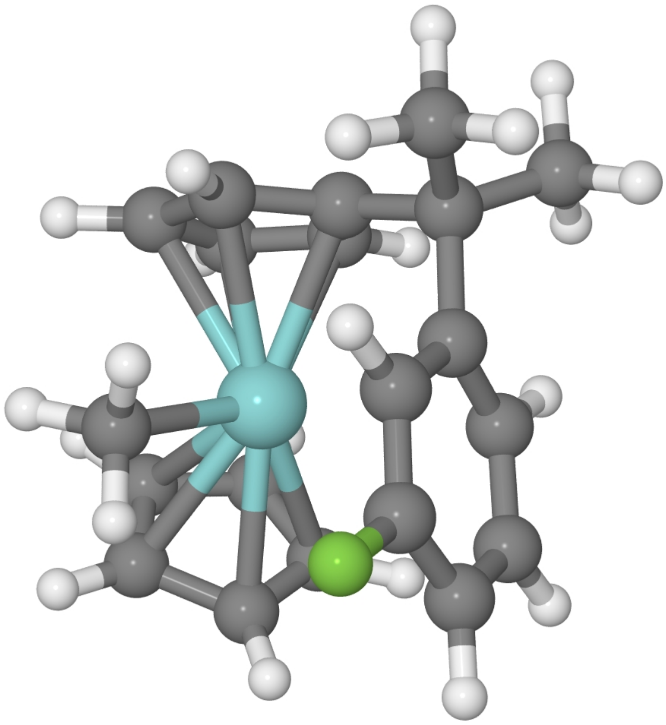

3A

3A-PBE-D3: E=	-921.2762184089		
C	-1.750325	1.051359	1.905582
C	-1.140389	2.050649	1.103869
C	-1.957279	2.261271	-0.046511
C	-3.067534	1.379969	0.037077
C	-2.942597	0.626759	1.241418
Zr	-1.056470	-0.131758	-0.182265
C	-2.126626	-0.358462	-2.173074
C	0.690240	-1.907687	0.137050
C	-0.231168	-2.447894	-0.811090
C	-1.489320	-2.636319	-0.175049
C	-1.362221	-2.197524	1.171604
C	-0.024466	-1.741371	1.367043
C	2.107637	-1.396211	-0.120220
C	3.022176	-1.803479	1.053254
C	1.915590	0.131458	-0.312788
C	2.458214	1.163874	0.478154
C	2.306387	2.520399	0.165428
C	1.544070	2.910899	-0.935771
C	0.929422	1.930665	-1.723728
C	1.126682	0.572234	-1.417519
F	3.191247	0.876816	1.572248
C	2.707723	-1.998759	-1.411075
H	0.779284	-0.183668	-2.134901
H	0.334964	2.199480	-2.598322
H	1.432564	3.969848	-1.174387
H	2.804448	3.251578	0.805780
H	2.121760	-1.778923	-2.314725
H	3.722010	-1.605070	-1.568738
H	2.774916	-3.091964	-1.309524
H	3.046199	-2.901597	1.106439
H	4.048683	-1.446422	0.892757
H	2.679386	-1.421136	2.021553
H	0.387259	-1.366146	2.301705
H	-2.133880	-2.250173	1.936925
H	-2.381179	-3.052935	-0.637872
H	-0.008403	-2.698164	-1.846177
H	-1.388026	0.690366	2.866157
H	-3.659768	-0.103628	1.611270
H	-3.884822	1.308941	-0.676349
H	-1.774367	2.980104	-0.842683
H	-0.215696	2.576587	1.334293
H	-3.128555	-0.815133	-2.097059
H	-2.265616	0.630673	-2.643201
H	-1.546847	-0.999274	-2.859356

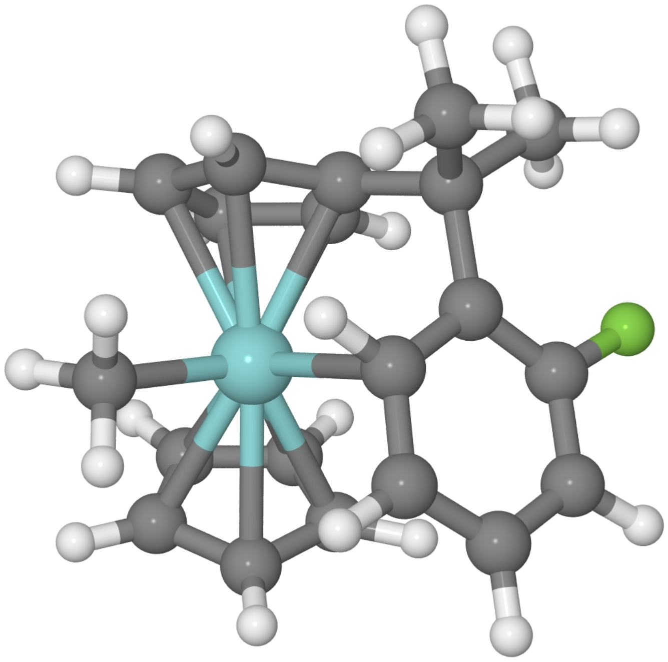

3B

3B-PBD-D3: E= -921.2833997688

C	0.782164	1.619175	-2.012978
C	-0.491237	1.205074	-1.535425
C	-0.613827	1.576013	-0.159675
C	0.609646	2.208355	0.211858
C	1.472896	2.232438	-0.930132
Zr	1.226866	-0.147119	-0.254288
C	2.254254	-1.081235	1.857193
C	2.721773	-1.905491	0.794270
C	3.548000	-1.115634	-0.052155
C	3.572863	0.207962	0.474448
C	2.770868	0.228695	1.661478
C	-1.846147	1.383744	0.723955
C	-2.710218	2.656017	0.554885
C	-2.607529	0.121555	0.286096
C	-2.001278	-1.129843	0.219048
C	-2.577813	-2.328954	-0.145860
C	-3.937128	-2.293784	-0.484716
C	-4.624701	-1.078390	-0.438162
C	-3.971789	0.102144	-0.062854
F	-0.597798	-1.210600	0.572932
C	-1.472381	1.249834	2.223412
C	1.463567	-1.293206	-2.186610
H	1.603711	-1.397502	2.672092
H	2.620826	1.083022	2.317746
H	4.149810	1.042037	0.080461
H	4.075575	-1.462435	-0.937286
H	2.497785	-2.963043	0.660354
H	1.158315	1.486118	-3.024370
H	2.456938	2.694297	-0.974412
H	0.841253	2.637236	1.184113
H	-1.991561	-3.248494	-0.161019
H	-4.443547	-3.214116	-0.779233
H	-5.683464	-1.041777	-0.700113
H	-4.536812	1.034528	-0.044631
H	-3.599543	2.614837	1.201354
H	-2.121821	3.536440	0.849043
H	-3.029666	2.795454	-0.488008
H	-2.388487	1.125455	2.817981
H	-0.821800	0.386890	2.431422
H	-0.968374	2.160008	2.580473
H	-1.250716	0.688228	-2.121316
H	0.509449	-1.239649	-2.741082
H	2.252643	-0.958372	-2.879767
H	1.677059	-2.349059	-1.943909

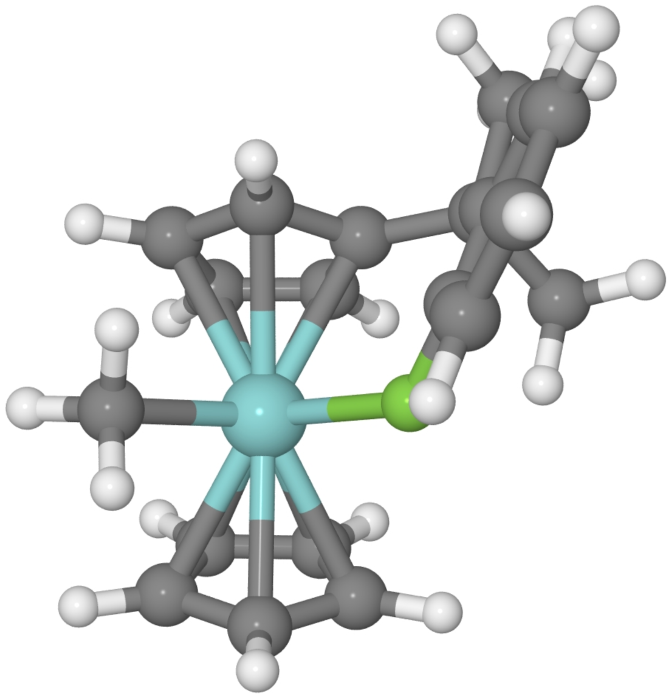

TS-3A-3B

TS-3A-3B: E= -921.2697057537

C	3.040113	-1.068318	-0.647499
C	3.313335	-0.007762	0.260988
C	2.650776	-0.299285	1.494918
C	1.972410	-1.535949	1.343181
C	2.195034	-2.003136	0.014439
Zr	0.864997	0.144565	-0.188384
C	0.985749	-0.085799	-2.447494
C	-0.022968	1.941995	1.220721
C	-0.964206	1.829688	0.140726
C	-0.267528	2.229865	-1.038871
C	1.054238	2.621179	-0.693541
C	1.200501	2.465120	0.711162
C	-2.332353	1.146681	0.160817
C	-3.068582	1.338457	1.504558
C	-1.997391	-0.344095	-0.126819
C	-1.443499	-1.222336	0.841326
C	-1.236032	-2.592175	0.625047
C	-1.541224	-3.137823	-0.620968
C	-2.031115	-2.300882	-1.632111
C	-2.246571	-0.943639	-1.387826
F	-1.150528	-0.746215	2.094113
C	-3.231099	1.744883	-0.939428
H	2.081487	2.710643	1.300105
H	1.805565	2.999974	-1.383998
H	-0.677671	2.267359	-2.043892
H	-2.658007	-0.326652	-2.185684
H	-2.269448	-2.710907	-2.615020
H	-1.399770	-4.205218	-0.798103
H	-0.870104	-3.203832	1.451659
H	-3.431946	2.798535	-0.700021
H	-2.781775	1.717992	-1.941436
H	-4.193956	1.214590	-0.981493
H	-3.310444	2.403756	1.630021
H	-4.013366	0.774859	1.492805
H	-2.491393	1.014196	2.375713
H	-0.220995	1.718032	2.267488
H	3.414144	-1.153055	-1.664052
H	1.798674	-2.920973	-0.416943
H	1.381597	-2.026249	2.114941
H	2.691743	0.299212	2.402820
H	3.946939	0.855016	0.062553
H	0.063544	0.282460	-2.925053
H	1.821205	0.468607	-2.908505
H	1.114279	-1.148485	-2.718065

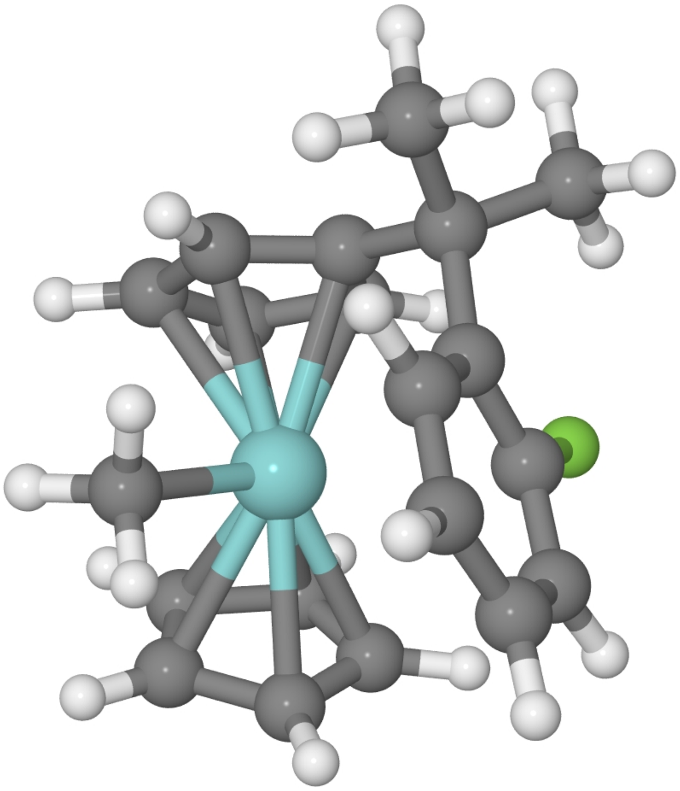

4A

4A-PBE-D3: E= -921.2822966005

C	-2.635866	-1.628453	-1.048283
C	-1.433729	-1.558528	-1.819236
C	-0.400876	-2.183242	-1.072692
C	-0.968360	-2.655322	0.150774
C	-2.349733	-2.327886	0.158785
Zr	-1.105489	-0.107661	0.180220
C	-2.207473	-0.160721	2.164807
C	1.198809	0.033902	1.352453
C	1.818451	0.661377	0.228852
C	2.728066	-0.094549	-0.522561
C	3.052749	-1.393971	-0.118530
C	2.489990	-2.005455	1.005276
C	1.554614	-1.277963	1.737952
C	1.475276	2.128732	-0.105563
C	2.190155	2.635171	-1.369592
F	3.943053	-2.080682	-0.848320
C	1.876291	3.028665	1.084561
C	-0.036851	2.127195	-0.324122
C	-0.714636	1.663352	-1.495984
C	-2.119788	1.668301	-1.234206
C	-2.315286	2.116717	0.101126
C	-1.037302	2.384305	0.662198
H	0.605483	0.633975	2.056248
H	1.106332	-1.709005	2.635351
H	2.792553	-3.015223	1.285534
H	3.236140	0.318239	-1.393994
H	1.419415	2.712815	2.033102
H	2.967382	3.005804	1.219265
H	1.573255	4.067789	0.888268
H	1.866097	3.662940	-1.585309
H	3.280552	2.651515	-1.223615
H	1.968698	2.022723	-2.255706
H	-0.251456	1.395168	-2.443372
H	-2.902212	1.427876	-1.951593
H	-3.270237	2.243351	0.606193
H	-0.864009	2.746365	1.673411
H	-1.334660	-1.121146	-2.810618
H	-3.613763	-1.263214	-1.357960
H	-3.062666	-2.564711	0.943994
H	-0.441411	-3.205357	0.927307
H	0.638106	-2.292139	-1.379026
H	-3.307101	-0.153553	2.067937
H	-1.948337	-1.087025	2.708157
H	-1.944010	0.704620	2.795865

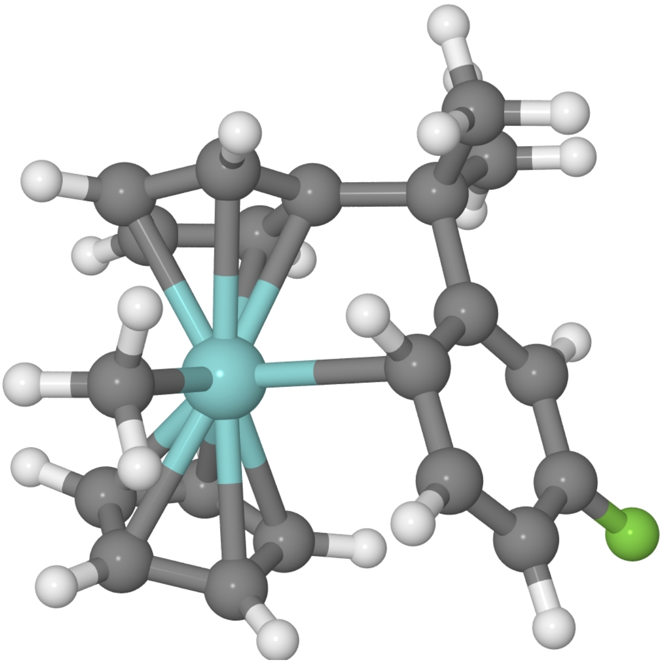

4B

4B-PBE-D3: E= -921.2799643698

C	3.101699	-0.834354	-0.775624
C	3.359230	0.274842	0.081456
C	2.812231	-0.017681	1.369729
C	2.208490	-1.302234	1.297866
C	2.380127	-1.803286	-0.026345
Zr	0.895777	0.248556	-0.217187
C	0.600172	-0.387069	-2.369374
C	-1.168174	-0.997803	0.765065
C	-1.058538	-2.373217	0.473174
C	-1.822908	-2.988801	-0.508805
C	-2.771096	-2.205426	-1.173769
C	-2.954344	-0.848995	-0.864159
C	-2.184253	-0.223314	0.121900
F	-0.196757	-3.119244	1.195586
C	-2.350879	1.256768	0.515923
C	-2.658182	1.366619	2.025983
C	0.050304	2.206426	1.081717
C	-1.007120	1.890863	0.169366
C	-0.514951	2.143266	-1.149499
C	0.816808	2.625725	-1.049619
C	1.166589	2.669529	0.330295
C	-3.476759	1.962990	-0.261984
H	-3.398126	-2.667632	-1.939314
H	-3.728848	-0.288547	-1.386659
H	-1.696985	-4.052530	-0.712884
H	-0.687598	-0.642672	1.691493
H	-3.518479	3.018336	0.042550
H	-3.322502	1.937789	-1.350067
H	-4.453454	1.509126	-0.037313
H	-2.725242	2.424448	2.320031
H	-3.621857	0.885953	2.247011
H	-1.895749	0.886241	2.656205
H	0.001449	2.155282	2.168201
H	2.104382	3.037413	0.741346
H	1.450871	2.926701	-1.882115
H	-1.062250	1.995324	-2.076963
H	-0.466750	-0.252875	-2.623933
H	1.178241	0.183177	-3.116949
H	0.857379	-1.450929	-2.498562
H	3.921768	1.167957	-0.185377
H	3.408770	-0.927714	-1.814524
H	2.031032	-2.765278	-0.397222
H	1.702509	-1.824894	2.107066
H	2.870397	0.619908	2.249929

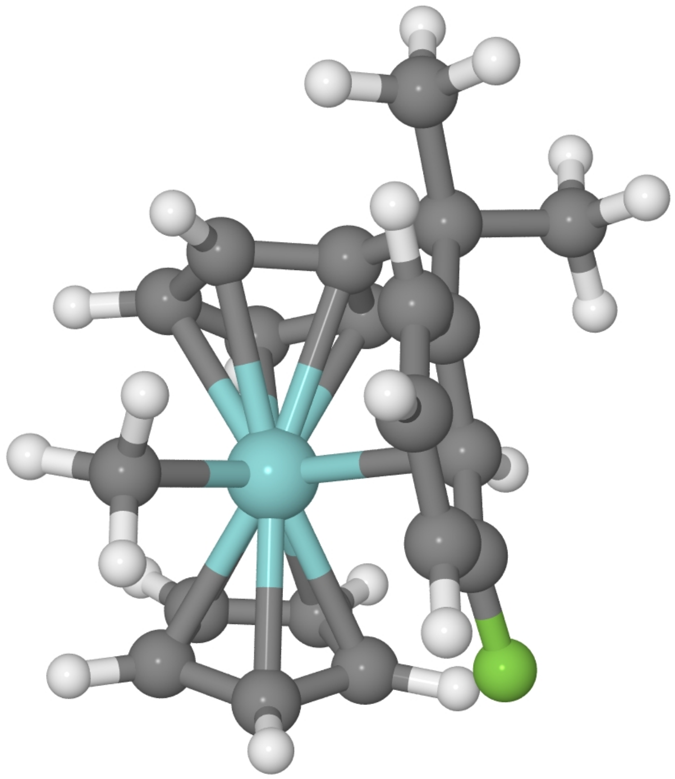

TS-4A-4B

TS-4A-4B: E= -921.2782210607

C	-2.925647	1.170223	-0.485009
C	-3.138824	0.287905	0.611305
C	-2.271092	0.688078	1.675863
C	-1.533299	1.816905	1.236875
C	-1.917939	2.100401	-0.106253
Zr	-0.829286	-0.193600	-0.182044
C	-1.277580	-0.284839	-2.411439
C	-0.187527	-1.913308	1.473531
C	0.771668	-2.024651	0.413239
C	0.065482	-2.476903	-0.740952
C	-1.297409	-2.677774	-0.397241
C	-1.453311	-2.340499	0.973043
C	2.190317	-1.457325	0.381118
C	2.826976	-1.440744	1.783651
C	1.972693	-0.023169	-0.165492
C	1.598643	1.025693	0.716306
C	1.508353	2.342550	0.234845
C	1.670522	2.664884	-1.106645
C	1.933873	1.613624	-1.993290
C	2.077463	0.293707	-1.539899
F	1.228784	3.315739	1.123610
C	3.096675	-2.290302	-0.545609
H	-2.368483	-2.427109	1.554058
H	-2.076056	-3.044999	-1.062551
H	0.489983	-2.658679	-1.724740
H	2.322746	-0.485182	-2.260742
H	2.047856	1.827237	-3.057940
H	1.590700	3.701192	-1.436884
H	1.535509	0.874890	1.795224
H	3.236370	-3.290969	-0.113028
H	2.683683	-2.427503	-1.553822
H	4.083973	-1.815128	-0.644199
H	2.975039	-2.473344	2.130207
H	3.807892	-0.944365	1.749971
H	2.214027	-0.928827	2.538545
H	0.014817	-1.605898	2.497430
H	-3.452768	1.146537	-1.434979
H	-1.537350	2.912292	-0.724563
H	-0.814239	2.380211	1.827135
H	-2.222377	0.239419	2.666179
H	-3.866341	-0.520232	0.647989
H	-0.481155	-0.836203	-2.938468
H	-2.231264	-0.767917	-2.681162
H	-1.313552	0.744208	-2.809876

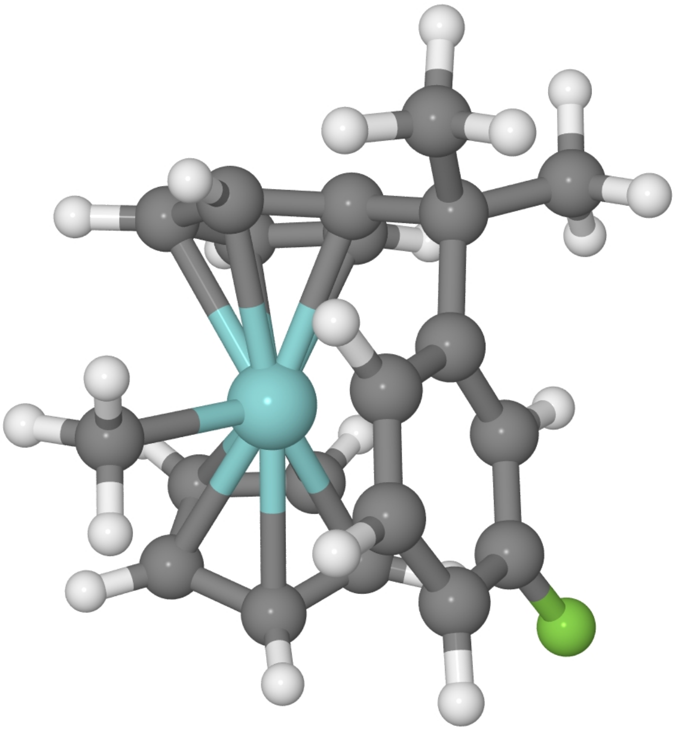

5A

5A-PBE-D3: E=	-921.2799669506		
C	1.453639	1.975731	-1.077080
C	0.693024	2.118137	0.126522
C	1.484517	1.576050	1.185338
C	2.724053	1.121510	0.636056
C	2.701711	1.374071	-0.762790
Zr	0.938750	-0.375297	-0.229398
C	-1.359164	0.414076	-1.050048
C	-1.577667	1.228474	0.095898
C	-2.562401	0.797888	1.007438
C	-3.297869	-0.373592	0.788476
C	-3.042779	-1.151270	-0.343206
C	-2.080601	-0.773166	-1.278383
C	-0.774880	2.543084	0.240172
C	-1.171830	3.505782	-0.901739
F	-3.720720	-2.296590	-0.524638
C	2.121526	-1.987790	1.222500
C	0.976778	-1.573929	1.971446
C	-0.168941	-2.056965	1.287706
C	0.261473	-2.805204	0.150128
C	1.678964	-2.771990	0.114648
C	1.596180	-1.013990	-2.304594
C	-1.030782	3.235895	1.587844
H	-4.073639	-0.693156	1.487330
H	-0.717066	0.787084	-1.861232
H	-1.923585	-1.367654	-2.180093
H	-2.786917	1.396474	1.890932
H	-1.017040	3.068417	-1.898417
H	-2.236118	3.769630	-0.816774
H	-0.579178	4.430433	-0.837133
H	-0.383992	4.119856	1.678306
H	-2.073976	3.578051	1.660050
H	-0.826466	2.580041	2.447317
H	1.215824	1.550897	2.238860
H	3.562800	0.722508	1.202358
H	3.500406	1.160420	-1.469364
H	1.148646	2.295251	-2.071818
H	0.983413	-0.996025	2.892777
H	3.160846	-1.817241	1.497497
H	2.315798	-3.255430	-0.621807
H	-0.386636	-3.319149	-0.557668
H	-1.202440	-1.907528	1.593880
H	2.636462	-1.374674	-2.368709
H	0.953194	-1.855023	-2.621983
H	1.498614	-0.193625	-3.035460

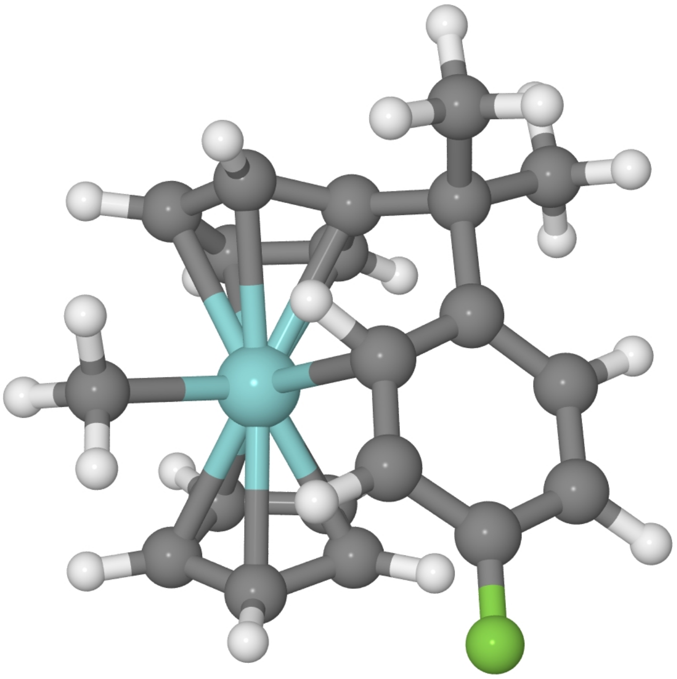

5B

5B-PBE-D3: E= -921.2765955307

C	1.914204	-2.281307	0.202667
C	0.826874	-2.308131	-0.717487
C	-0.385031	-2.109903	0.024009
C	-0.021420	-1.941665	1.394291
C	1.390247	-2.045653	1.504519
Zr	0.952046	0.033008	0.135230
C	-1.286252	0.467962	-1.024340
C	-1.444971	1.859110	-0.857334
C	-2.414224	2.308590	0.038058
C	-3.245482	1.413849	0.712989
C	-3.097764	0.036560	0.510334
C	-2.116669	-0.471815	-0.352896
F	-2.554055	3.628822	0.244414
C	-1.806992	-1.971560	-0.520420
C	-2.759104	-2.873703	0.288300
C	2.798156	1.716889	0.560511
C	3.397213	0.533096	0.041256
C	2.964594	0.369419	-1.311887
C	2.097639	1.451463	-1.621139
C	1.985376	2.276604	-0.463344
C	0.398920	0.959147	2.124841
C	-1.887668	-2.383111	-2.005037
H	-4.007591	1.805488	1.389624
H	-3.763407	-0.644352	1.041315
H	-0.854655	2.577104	-1.427554
H	-0.627108	0.110588	-1.839018
H	-2.446138	-3.921852	0.181229
H	-2.756967	-2.637088	1.361831
H	-3.790528	-2.788053	-0.085632
H	-1.613533	-3.442579	-2.116377
H	-2.913285	-2.253387	-2.379424
H	-1.222035	-1.792918	-2.652548
H	0.907419	-2.489683	-1.787690
H	2.959638	-2.449004	-0.046657
H	1.965153	-1.974725	2.426013
H	-0.704823	-1.760376	2.220060
H	-0.639424	0.640491	2.335868
H	1.005218	0.650076	2.992416
H	0.423344	2.059878	2.080777
H	4.098773	-0.109325	0.570188
H	2.948755	2.126181	1.556106
H	1.405456	3.194136	-0.380926
H	1.624106	1.630593	-2.586615
H	3.276725	-0.417653	-1.995663

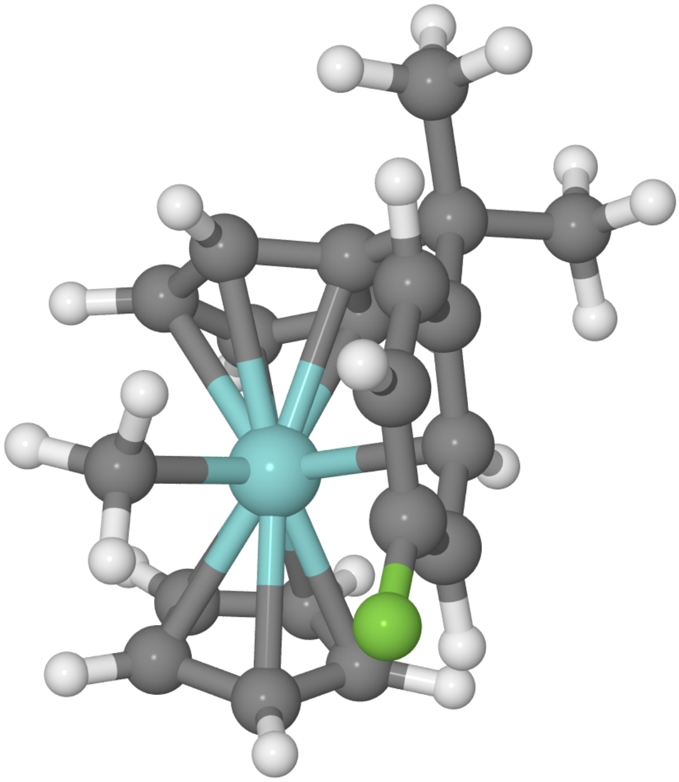

TS-5A-5B

TS-5A-5B: E= -921.2723725855

C	1.711041	-2.685009	-0.534229
C	2.612459	-2.008908	0.335297
C	1.936639	-1.769418	1.572827
C	0.625527	-2.298578	1.461639
C	0.474708	-2.843440	0.153005
Zr	0.828461	-0.348672	-0.179114
C	0.632783	-0.703462	-2.410921
C	1.518169	1.610683	1.167069
C	0.648721	2.124930	0.147613
C	1.312760	1.944258	-1.101512
C	2.575825	1.337409	-0.862413
C	2.711012	1.149559	0.539729
C	-0.808639	2.543293	0.324434
C	-1.065015	3.149825	1.718673
C	-1.581283	1.210205	0.143497
C	-1.586236	0.228153	1.172280
C	-2.356653	-0.945368	1.065279
C	-3.087256	-1.168167	-0.096798
C	-3.055209	-0.260058	-1.157652
C	-2.310845	0.915777	-1.031249
F	-3.820074	-2.288808	-0.207986
C	-1.196366	3.596580	-0.729262
H	3.586311	0.752446	1.049032
H	3.317734	1.092943	-1.620210
H	0.927202	2.221109	-2.078585
H	-2.330576	1.630556	-1.853542
H	-3.630563	-0.471444	-2.060790
H	-2.410176	-1.667680	1.881214
H	-1.076047	0.426218	2.118995
H	-0.608773	4.510264	-0.561901
H	-1.006986	3.272000	-1.761925
H	-2.262226	3.854936	-0.642333
H	-0.476321	4.071340	1.836936
H	-2.129393	3.405311	1.825725
H	-0.798293	2.480845	2.548736
H	1.334132	1.615274	2.239784
H	1.931480	-3.030626	-1.540692
H	-0.419975	-3.321921	-0.243382
H	-0.123657	-2.300209	2.249870
H	2.364177	-1.300174	2.456565
H	3.648192	-1.761580	0.111080
H	0.102196	0.153261	-2.860303
H	1.569950	-0.832206	-2.976277
H	0.027002	-1.612235	-2.571916

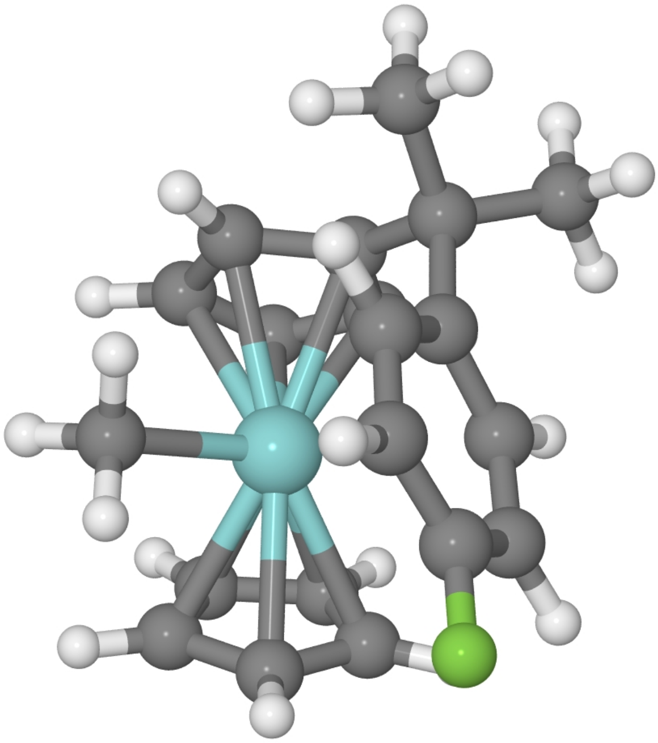

6A

6A-PBE-D3: E= -4414.3991296780

Zr	0.944940	0.227873	-0.173975
C	3.194921	-0.971815	-0.138540
C	3.137115	-0.125106	0.999152
C	2.141732	-0.637197	1.878798
C	1.570804	-1.776600	1.270533
C	2.218639	-1.981502	0.020524
H	2.026251	-2.790598	-0.668594
H	0.792382	-2.396646	1.691191
H	1.889325	-0.241280	2.850996
H	3.786185	0.715588	1.195056
H	3.872073	-0.868105	-0.971621
C	1.668623	0.432855	-2.319126
H	2.607208	0.991566	-2.405525
H	1.855831	-0.560779	-2.743329
H	0.940177	0.948438	-2.953081
C	-1.039569	1.711194	0.365814
C	-0.425444	2.305313	-0.772373
C	0.858710	2.773708	-0.405967
C	1.060365	2.465171	0.960232
C	-0.105311	1.805006	1.437437
H	-0.265464	1.478795	2.453611
H	1.919554	2.738960	1.553982
H	1.549894	3.293309	-1.052266
H	-0.864810	2.410121	-1.752363
C	-2.367882	0.953874	0.406154
C	-3.071270	1.205479	1.753156
H	-3.247783	2.275242	1.875989
H	-4.040942	0.704084	1.784981
H	-2.484916	0.865271	2.608820
C	-2.010214	-0.536377	0.195555
C	-1.318379	-0.929937	-0.977056
C	-1.025380	-2.274278	-1.250364
C	-1.416248	-3.236622	-0.334242
C	-2.083890	-2.889767	0.833306
C	-2.367564	-1.550797	1.094878
H	-2.907518	-1.308787	2.000040
H	-2.387562	-3.670248	1.520796
H	-1.191619	-4.277660	-0.530887
H	-0.542494	-2.568162	-2.174100
F	-1.114637	-0.042259	-1.957241
C	-3.314508	1.419434	-0.723257
H	-2.895382	1.267787	-1.719301
H	-4.253473	0.864250	-0.674835
H	-3.540027	2.482061	-0.609436

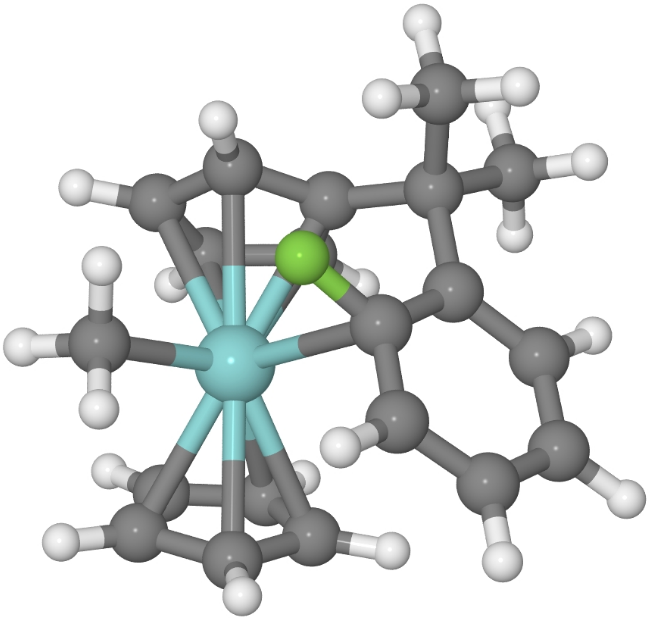

6B

6B-PBE-D3: E= -4414.3995230413

Zr	-0.914615	-0.108976	-0.270645
C	-3.207834	0.937346	-0.643750
C	-2.507593	1.865925	0.159734
C	-2.271254	1.264016	1.424910
C	-2.812359	-0.042239	1.399637
C	-3.385570	-0.249996	0.113530
H	-3.924931	-1.129234	-0.206190
H	-2.824475	-0.742191	2.221867
H	-1.800340	1.734453	2.276905
H	-2.231884	2.868543	-0.132095
H	-3.555099	1.105802	-1.651280
C	-0.732896	0.658238	-2.394874
H	0.321865	0.609610	-2.690533
H	-1.292164	0.071061	-3.131641
H	-1.066347	1.695925	-2.483503
C	-1.107445	-2.608183	0.089187
C	-0.014572	-2.161940	0.874294
C	1.026182	-1.734914	-0.004291
C	0.544891	-1.904846	-1.332389
C	-0.763845	-2.440084	-1.274296
H	-1.377276	-2.704340	-2.123450
H	1.082911	-1.679039	-2.239204
C	2.365919	-1.103274	0.378464
C	2.703977	-1.343710	1.866424
H	2.792222	-2.414222	2.063959
H	3.658549	-0.873782	2.111180
H	1.954745	-0.936214	2.548077
C	2.191439	0.406601	0.116242
C	1.172222	1.112580	0.801153
C	0.965460	2.488033	0.605947
C	1.752767	3.150459	-0.319326
C	2.750704	2.486367	-1.020055
C	2.964216	1.127210	-0.799135
H	3.756509	0.636606	-1.347221
H	3.357916	3.040183	-1.725989
H	1.596428	4.162981	-0.486065
H	0.234529	3.041258	1.181029
F	0.559035	0.532199	1.839133
C	3.490537	-1.728320	-0.470236
H	3.538499	-2.800729	-0.273954
H	3.335106	-1.595886	-1.542088
H	4.460194	-1.298786	-0.208562
H	0.034352	-2.197669	1.952511
H	-2.022049	-3.039950	0.466232

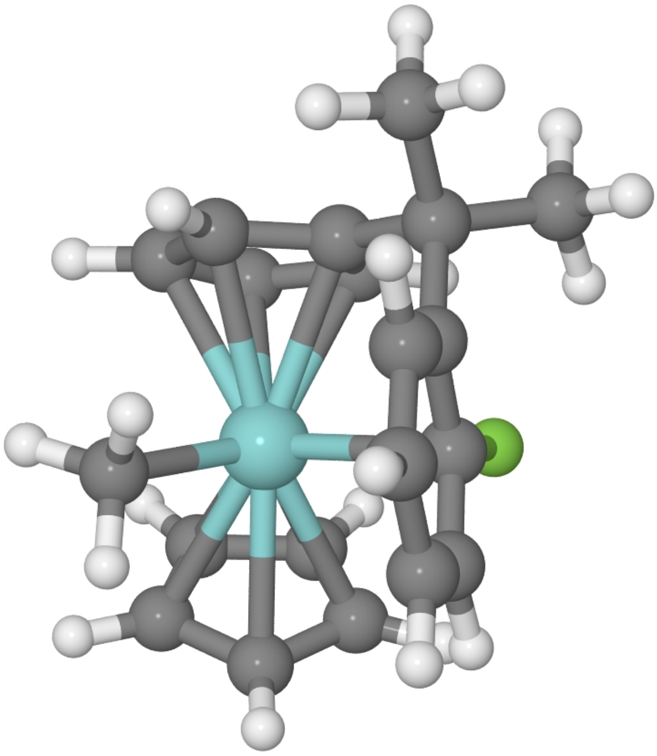

***ortho tert*-butyl-fluorobenzene**

ortho tert-butyl-fluorobenzene E= -488.2567861278

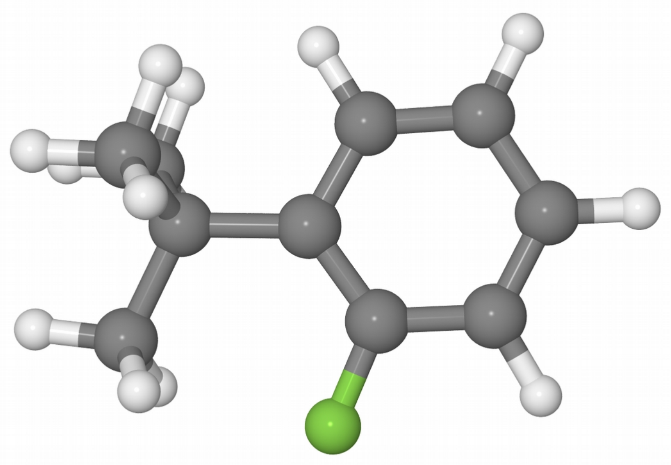

C	1.573522	0.182190	-0.000241
C	0.029205	0.190874	-0.000044
C	-0.639794	1.435017	0.000341
C	-2.029599	1.554595	0.000536
C	-2.826257	0.404237	0.000345
C	-2.209480	-0.845596	-0.000040
C	-0.814928	-0.932028	-0.000225
F	-0.306703	-2.199119	-0.000587
H	-2.782771	-1.774413	-0.000204
H	-3.915828	0.476031	0.000494
H	-2.488925	2.545346	0.000831
H	-0.039181	2.347536	0.000494
C	2.207863	-1.223820	-0.000536
H	1.921671	-1.802885	-0.888954
H	3.304348	-1.114473	-0.000641
H	1.921867	-1.803165	0.887763
C	2.071554	0.923631	-1.264294
H	1.736819	0.405768	-2.176450
H	1.703822	1.959550	-1.309743
H	3.173125	0.956152	-1.272964
C	2.071885	0.923246	1.263910
H	3.173461	0.955698	1.272335
H	1.704238	1.959177	1.309738
H	1.737335	0.405137	2.175994

meta tert-butyl-fluorobenzene

meta tert-butyl-fluorobenzene E= -488.2609082955

C	1.862666	-0.163226	-0.000249
C	0.358115	0.158180	-0.000013
C	-0.072469	1.500402	0.000230
C	-1.430086	1.827943	0.000430
C	-2.407920	0.825922	0.000401
C	-1.975006	-0.495481	0.000172
C	-0.624276	-0.844505	-0.000032
H	-0.371100	-1.904807	-0.000210
F	-2.898783	-1.494345	0.000140
H	-3.475253	1.051362	0.000552
H	-1.734994	2.876892	0.000614
H	0.664601	2.306297	0.000254
C	2.137101	-1.678163	-0.000566
H	1.718533	-2.167752	-0.893863
H	3.223572	-1.856668	-0.000768
H	1.718820	-2.168095	0.892678
C	2.512095	0.448931	-1.263451
H	2.068371	0.022387	-2.176414
H	2.379062	1.540483	-1.302318
H	3.594273	0.240418	-1.275582
C	2.512389	0.448468	1.263024
H	3.594552	0.239855	1.274888
H	2.379450	1.540018	1.302250
H	2.068792	0.021675	2.175932

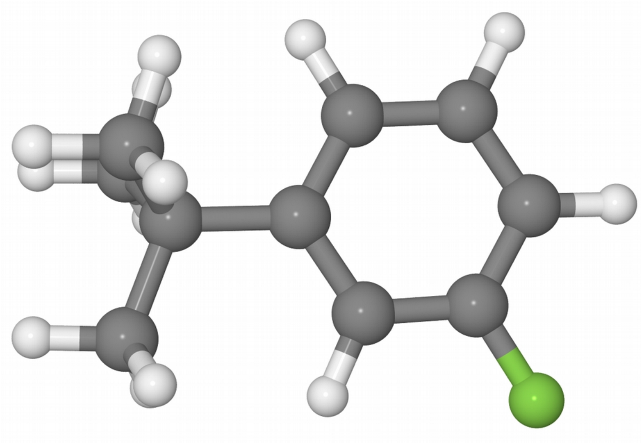

***para tert*-butyl-fluorobenzene**

para tert-butyl-fluorobenzene E= -488.2600867862

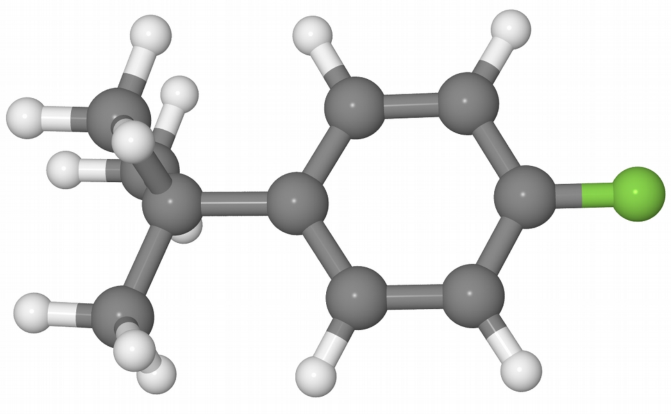

C	2.002415	0.000024	-0.000247
C	0.463303	-0.029361	-0.000034
C	-0.261221	1.179545	0.000371
C	-1.657510	1.204349	0.000571
C	-2.347836	-0.006462	0.000365
C	-1.676789	-1.224041	-0.000030
C	-0.275775	-1.224197	-0.000228
H	0.236246	-2.187273	-0.000534
H	-2.247714	-2.154211	-0.000177
F	-3.707156	0.004740	0.000560
H	-2.214554	2.142861	0.000886
H	0.271507	2.133583	0.000538
C	2.612324	-1.413442	-0.000548
H	2.316811	-1.985534	-0.894085
H	3.711159	-1.341432	-0.000649
H	2.316989	-1.985823	0.892862
C	2.497710	0.742567	-1.263265
H	2.163874	0.225288	-2.176221
H	2.118164	1.774743	-1.303704
H	3.599018	0.787546	-1.275630
C	2.498061	0.742184	1.262858
H	3.599372	0.787154	1.274926
H	2.118536	1.774349	1.303709
H	2.164484	0.224618	2.175746

tert-butyl-benzene

tert-butyl-benzene E= -389.0774005409

C	1.533116	-0.001928	-0.000290
C	-0.006434	-0.029347	-0.000041
C	-0.728171	1.180994	0.000278
C	-2.124976	1.198927	0.000517
C	-2.845207	-0.001629	0.000446
C	-2.148198	-1.211733	0.000136
C	-0.746606	-1.223400	-0.000104
H	-0.233223	-2.186249	-0.000340
H	-2.693942	-2.158801	0.000079
H	-3.937757	0.008568	0.000635
H	-2.653254	2.155767	0.000763
H	-0.191041	2.133124	0.000339
C	2.144070	-1.414886	-0.000592
H	1.848334	-1.987198	-0.893843
H	3.242994	-1.342084	-0.000709
H	1.848537	-1.987482	0.892543
C	2.027997	0.740980	-1.263311
H	1.694138	0.223419	-2.176143
H	1.647616	1.772724	-1.303583
H	3.129399	0.785990	-1.275691
C	2.028398	0.740619	1.262789
H	3.129804	0.785617	1.274837
H	1.648035	1.772354	1.303467
H	1.694824	0.222796	2.175576

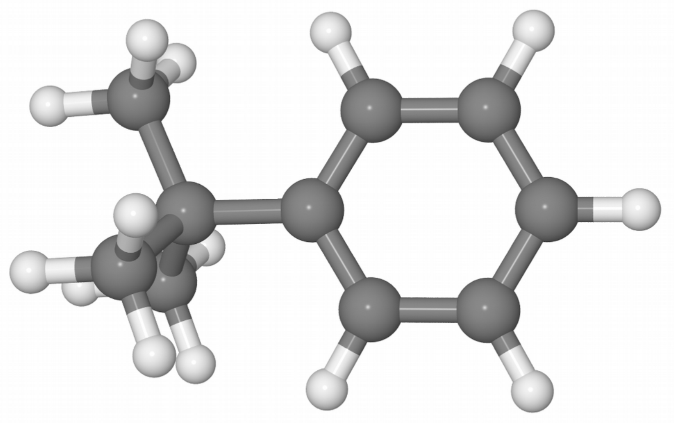

Electronic Properties

QTAIM and NBO charges

tert-butyl-fluorobenzene

Table S1: QTAIM and NBO charges of *ortho*, *meta* and *para tert*-butyl-fluorobenzene

	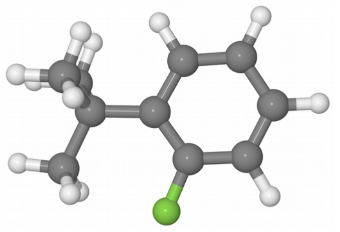		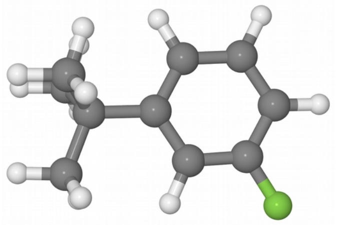		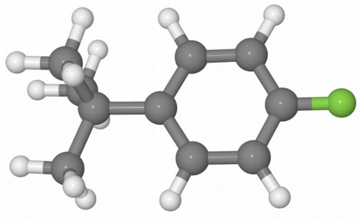	
	B3LYP/ 6-311G(d,p)	PBE-D3/ 6-311G(d,p)	B3LYP/ 6-311G(d,p)	PBE-D3/ 6-311G(d,p)	B3LYP/ 6-311G(d,p)	PBE-D3/ 6-311G(d,p)
C*ipso* (Bader)	0.0045	0.0010	0.0121	-0.0039	-0.0057	-0.0046
C*ipso* (NBO)	-0.0904	-0.08815	-0.0006	-0.0076	-0.0391	-0.0421
C2 (Bader)	-0.0067	-0.0152	-0.0082	-0.0213	0.0105	-0.0131
C2 (NBO)	-0.1840	-0.1977	-0.2255	-0.2352	-0.1859	-0.1996
C3 (Bader)	0.0229	0.0035	0.0300	-0.0021	0.0383	0.0031
C3 (NBO)	-0.2104	-0.2214	-0.1729	-0.1875	-0.2643	-0.2717
C4 (Bader)	0.0216	-0.0064	0.0344	0.0075	0.4912	0.4676
C4 (NBO)	-0.1887	-0.2015	-0.2811	-0.2871	0.4249	0.3902
C5 (Bader)	0.0386	0.0068	0.4907	0.4712	0.0355	-0.0011
C5 (NBO)	-0.2601	-0.2683	0.4419	0.4058	-0.2643	-0.2713
C6 (Bader)	0.4702	0.4453	0.0144	-0.0175	0.0015	-0.0149
C6 (NBO)	0.4235	0.3899	-0.2870	-0.2930	-0.1926	-0.2062
F (Bader)	-0.6283	-0.5908	-0.6156	-0.5856	-0.6250	-0.5766
F (NBO)	-0.3589	-0.3265	-0.3538	-0.3205	-0.3525	-0.3193

tert-butyl-benzene

Table S2: QTAIM and NBO charges of *tert*-butyl-benzene

	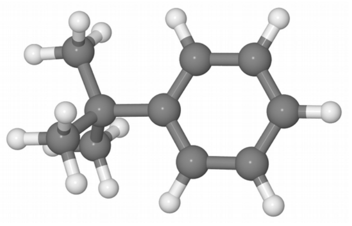	
	B3LYP/<br>6-311G(d,p)	PBE-D3/<br>6-311G(d,p)
C*ipso* (Bader)	-0.0226	-0.0216
C*ipso* (NBO)	-0.0208	-0.0248
C2 (Bader)	-0.0029	-0.0319
C2 (NBO)	-0.2055	-0.2164
C3 (Bader)	0.0078	-0.0114
C3 (NBO)	-0.1909	-0.2029
C4 (Bader)	0.0054	-0.0195
C4 (NBO)	-0.2064	-0.2029
C5 (Bader)	0.0167	-0.0192
C5 (NBO)	-0.1888	-0.2006
C6 (Bader)	-0.0064	-0.0346
C6 (NBO)	-0.2118	-0.2226

Compounds 6A and 6B

Table S3: QTAIM and NBO6 charges of **6**

	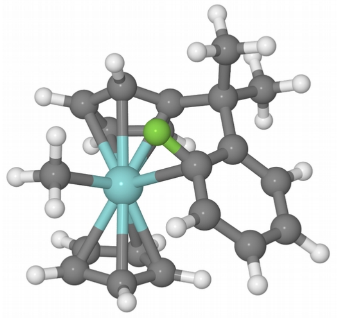 6a		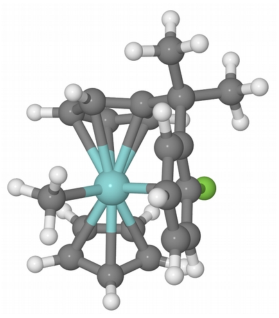 6b	
	B3LYP/ ecp11	PBE-D3/ ecp11	B3LYP/ ecp11	PBE-D3/ ecp11
Zr (Bader)	1.9136	1.8302	1.9285	1.8452
Zr (NBO)	2.3824	2.2575	2.3917	2.2683
C1 (Bader)	-0.3781	-0.4086	-0.3890	-0.4205
C1 (NBO)	-1.1693	-1.1874	-1.1869	-1.2050
C2 (Bader)	0.0647	0.0720	0.3544	0.3519
C2 (NBO)	-0.1864	-0.1945	0.2484	0.2306
C*ipso* (Bader)	0.0216	0.0174	0.0452	0.0468
C*ipso* (NBO)	-0.0632	-0.0645	-0.0383	-0.0415
C6 (Bader)	0.3867	0.3849	0.0764	0.0924
C6 (NBO)	0.2669	0.2473	-0.1919	-0.1995
F (Bader)	-0.6353	-0.6008	-0.6412	-0.6096
F (NBO)	-0.3261	-0.2890	-0.3363	-0.2993

QTAIM and NBO properties

IIA-B3LYP

Bader:

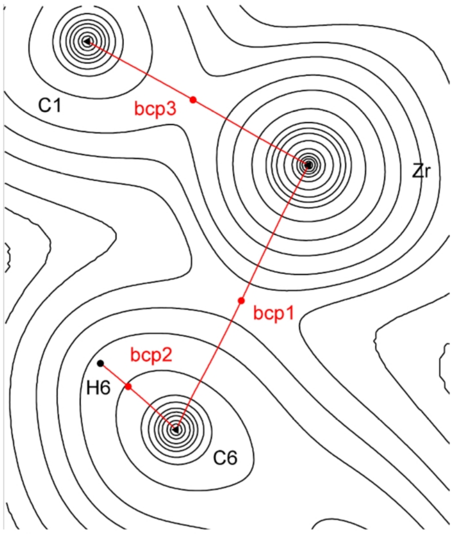

Electrondensity

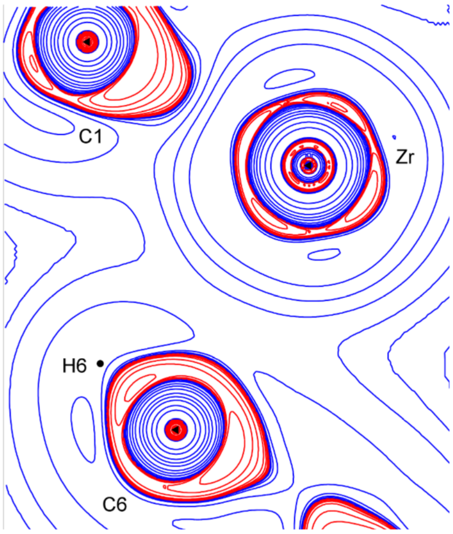

Laplacian

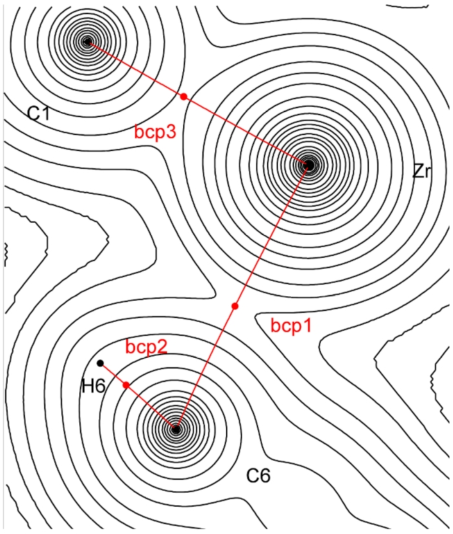

Virial

	$\varrho(\mathbf{r})$	$\nabla^2\varrho(\mathbf{r})$
bcp1	0.03121	-0.02007
bcp2	0.27581	0.22955
bcp3	0.09473	-0.01211

NBO:

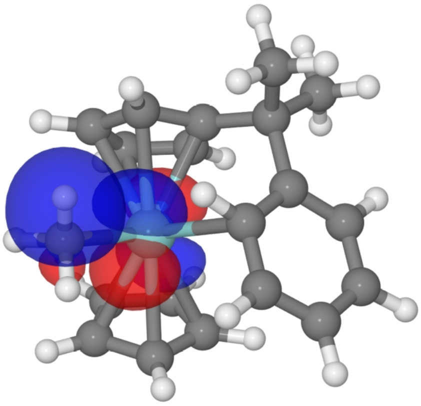

1

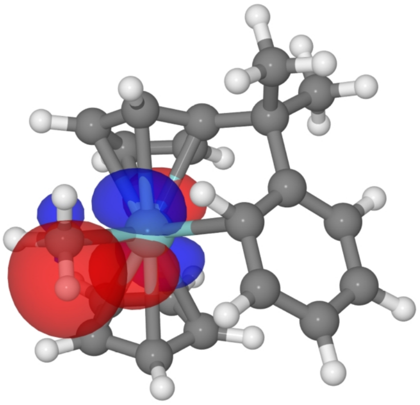

2

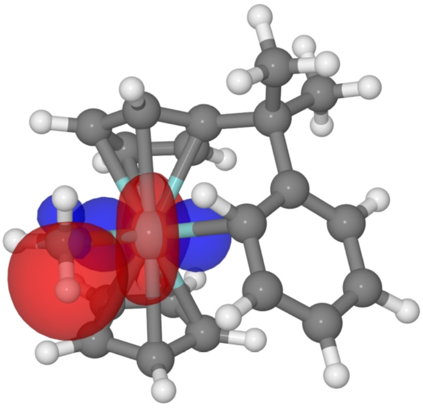

3

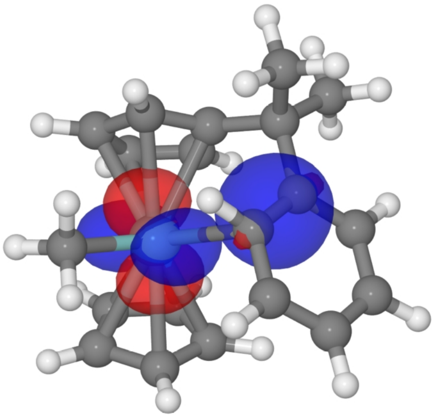

4

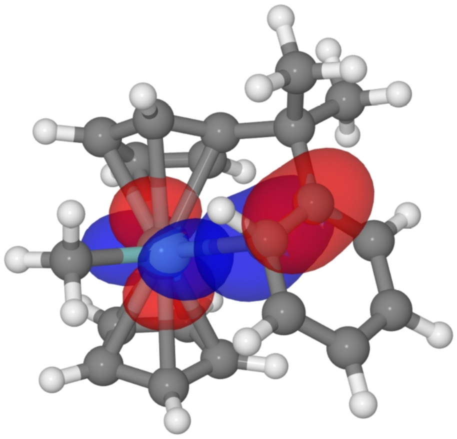

5

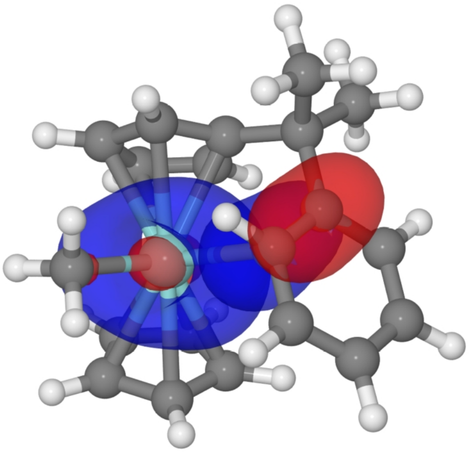

6

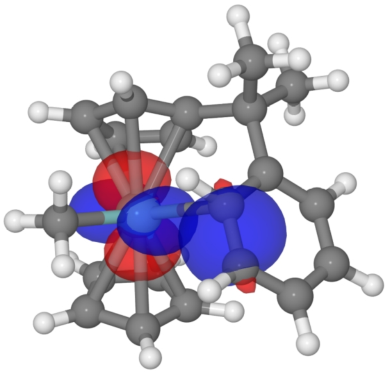

7

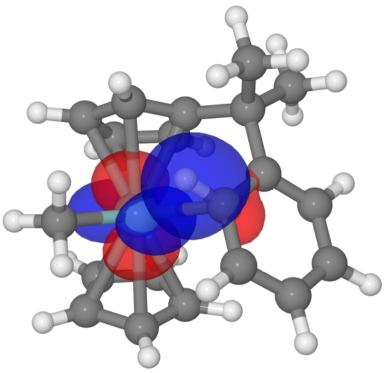

8

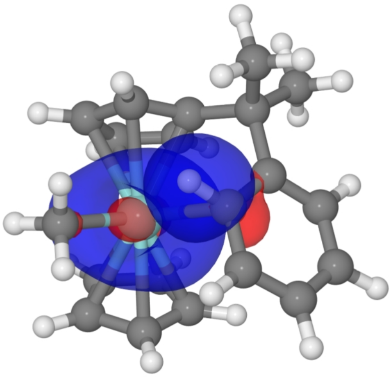

9

	Orbitals	E(2P)
1	σ_{CH} = 0.776$(sp^{2.95})_{C7}$ – 0.631$(s)_{H42}$ → LV_{Zr} = $p^{2.01}d^{99.99}$	2.07
2	σ_{CH} = 0.776$(sp^{2.97})_{C7}$ – 0.631$(s)_{H44}$ → LV_{Zr} = $p^{2.01}d^{99.99}$	2.01
3	σ_{CH} = 0.776$(sp^{2.97})_{C7}$ – 0.631$(s)_{H44}$ → LV_{Zr} = $sp^{0.04}d^{15.40}$	2.15
4	σ_{CC} = 0.710$(sp^{1.74})_{C8}$ – 0.704$(sp^{2.03})_{C9}$ → LV_{Zr} = $sp^{0.04}d^{46.05}$	2.42
5	π_{CC} = 0.760$(sp^{99.99})_{C8}$ – 0.650$(sp^{99.99}d^{0.68})_{C9}$ → LV_{Zr} = $sp^{0.04}d^{46.05}$	7.75
6	π_{CC} = 0.760$(sp^{99.99})_{C8}$ – 0.650$(sp^{99.99}d^{0.68})_{C9}$ → LV_{Zr} = $sd^{0.31}$	8.21
7	σ_{CC} = 0.715$(sp^{1.80})_{C8}$ – 0.699$(sp^{1.78})_{C13}$ → LV_{Zr} = $sp^{0.04}d^{46.05}$	2.85
8	σ_{CH} = 0.788$(sp^{2.64})_{C8}$ – 0.616$(s)_{H33}$ → LV_{Zr} = $sp^{0.04}d^{46.05}$	4.92
9	σ_{CH} = 0.788$(sp^{2.64})_{C8}$ – 0.616$(s)_{H33}$ → LV_{Zr} = $sd^{0.31}$	2.85

Natural Resonance Theory:

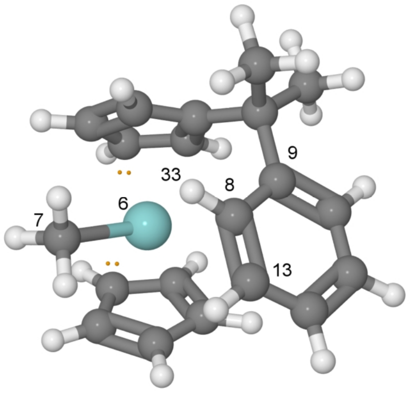

1

Wgt=15.76%;
rhoNL=5.13401;
D(0)=0.09806

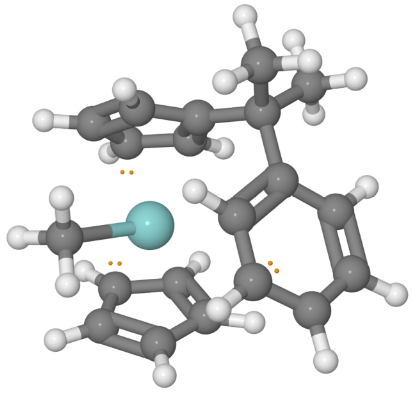

2

Wgt=10.78%;
rhoNL=5.83298;
D(0)=0.10452

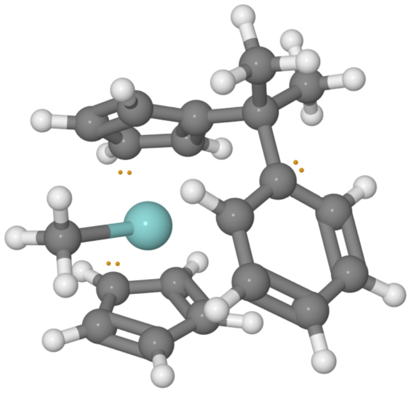

3

Wgt=10.02%;
rhoNL=5.84995;
D(0)=0.10467

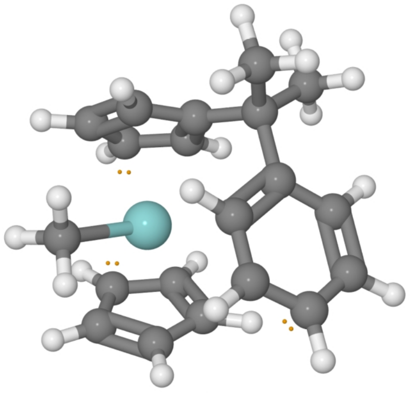

4

Wgt=7.62%;
rhoNL=5.79497;
D(0)=0.10418

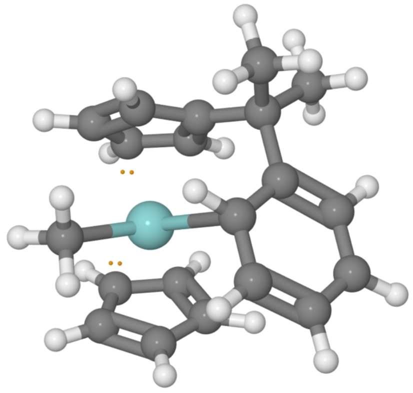

5

Wgt=7.59%;
rhoNL=5.56547;
D(0)=0.1021

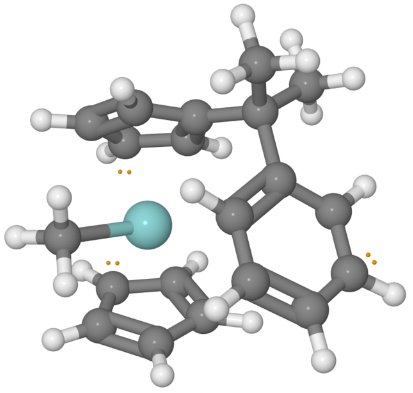

6

Wgt=7.12%;
rhoNL=5.83150;
D(0)=0.10451

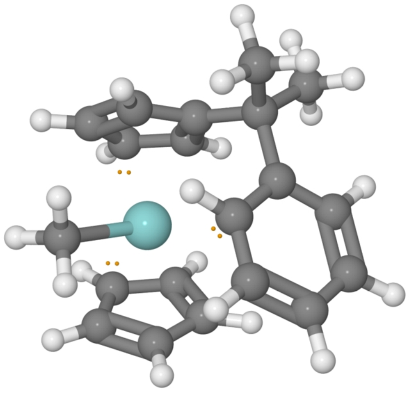

7

Wgt=6.89%;
rhoNL=5.66471;
D(0)=0.10300

Natural Localised Molecular Orbitals (NLMO):

Only contributions over 1% are reported.

NLMO / Occupancy / Percent from Parent NBO / Atomic Hybrid Contributions

Resonance structure **1**:
C-H interaction:

```
  60. (2.00000)  97.7498% BD ( 1) C  8- H 33
                  0.993% Zr  6 s( 24.46%)p 0.04(  0.94%)d 3.05( 74.60%)
                 60.816%  C  8 s( 25.32%)p 2.94( 74.57%)d 0.00(  0.11%)
                 36.990%  H 33 s( 99.95%)p 0.00(  0.05%)
```

C-C interaction:

```
 59. (2.00000)  83.8045% BD ( 2) C  8- C 13
                  2.695% Zr  6 s( 28.03%)p 0.03(  0.97%)d 2.53( 71.00%)
                 49.067%  C  8 s(  0.61%)p99.99( 99.37%)d 0.04(  0.02%)
                  5.099%  C  9 s(  0.14%)p99.99( 99.49%)d 2.54(  0.37%)
                  1.770%  C 10 s(  0.03%)p99.99( 99.82%)d 4.52(  0.15%)
                  1.188%  C 11 s(  0.07%)p99.99( 99.63%)d 4.05(  0.30%)
                  4.859%  C 12 s(  0.01%)p 1.00( 99.66%)d 0.00(  0.33%)
                 34.757%  C 13 s(  0.01%)p99.99( 99.94%)d 3.11(  0.05%)
```

Zr-Me interaction:

```
  53. (2.00000)  97.7841% BD ( 1)Zr  6- C  7
                 19.506% Zr  6 s( 13.33%)p 0.01(  0.08%)d 6.49( 86.59%)
                 78.287%  C  7 s( 25.96%)p 2.85( 74.03%)d 0.00(  0.02%)
```

Resonance structure **2**, **3**:
NLMO algorithm failed to converge

Resonance structure **4**:
C-H interaction:

```
  61. (2.00000)  97.7460% BD ( 1) C  8- H 33
                  0.997% Zr  6 s( 24.27%)p 0.04(  0.93%)d 3.08( 74.80%)
                 60.814%  C  8 s( 25.31%)p 2.95( 74.58%)d 0.00(  0.11%)
                 36.989%  H 33 s( 99.95%)p 0.00(  0.05%)
```

C-C interaction:

```
 59. (2.00000)  83.0879% BD ( 2) C  8- C  9
                  3.268% Zr  6 s( 25.57%)p 0.04(  0.96%)d 2.87( 73.47%)
                 48.166%  C  8 s(  0.66%)p99.99( 99.31%)d 0.05(  0.03%)
                 34.940%  C  9 s(  0.07%)p99.99( 99.87%)d 0.73(  0.05%)
                  4.669%  C 10 s(  0.00%)p 1.00( 99.66%)d 0.00(  0.34%)
                  1.200%  C 11 s(  0.09%)p99.99( 99.60%)d 3.26(  0.31%)
                  1.642%  C 12 s(  0.11%)p99.99( 99.71%)d 1.55(  0.18%)
                  5.092%  C 13 s(  0.01%)p99.99( 99.66%)d24.44(  0.33%)
```

Zr-Me interaction:
54. (2.00000) 97.7841% BD (1)Zr 6- C 7
19.504% Zr 6 s(13.34%)p 0.01(0.08%)d 6.49(86.59%)
78.288% C 7 s(25.96%)p 2.85(74.03%)d 0.00(0.02%)

Resonance structure **5**:
C-H interaction:
60. (2.00000) 97.7822% BD (1) C 8- H 33
1.030% Zr 6 s(25.30%)p 0.04(0.89%)d 2.92(73.81%)
60.868% C 8 s(25.39%)p 2.93(74.50%)d 0.00(0.11%)
36.952% H 33 s(99.95%)p 0.00(0.05%)

Zr-C interaction:
54. (2.00000) 58.8263% BD (1)Zr 6- C 8
4.047% Zr 6 s(26.06%)p 0.03(0.89%)d 2.80(73.05%)
54.932% C 8 s(0.52%)p99.99(99.46%)d 0.03(0.02%)
13.750% C 9 s(0.08%)p99.99(99.76%)d 1.96(0.16%)
1.338% C 10 s(0.08%)p99.99(99.20%)d 8.90(0.72%)
10.375% C 11 s(0.05%)p99.99(99.95%)d 0.06(0.00%)
1.339% C 12 s(0.21%)p99.99(99.06%)d 3.52(0.73%)
13.537% C 13 s(0.01%)p 1.00(99.85%)d 0.00(0.14%)

Zr-Me interaction:
53. (2.00000) 97.7840% BD (1)Zr 6- C 7
19.504% Zr 6 s(13.33%)p 0.01(0.08%)d 6.50(86.59%)
78.288% C 7 s(25.96%)p 2.85(74.03%)d 0.00(0.02%)

Resonance structure **6**:
NLMO algorithm failed to converge

Resonance structure **7**:
C-H interaction:
60. (2.00000) 97.7768% BD (1) C 8- H 33
1.050% Zr 6 s(25.00%)p 0.04(0.88%)d 2.97(74.12%)
60.890% C 8 s(25.40%)p 2.93(74.50%)d 0.00(0.11%)
36.925% H 33 s(99.95%)p 0.00(0.05%)

Zr-Me interaction:
54. (2.00000) 97.7843% BD (1)Zr 6- C 7
19.504% Zr 6 s(13.33%)p 0.01(0.08%)d 6.49(86.59%)
78.288% C 7 s(25.96%)p 2.85(74.03%)d 0.00(0.02%)

Non-Covalent Interactions (NCI)

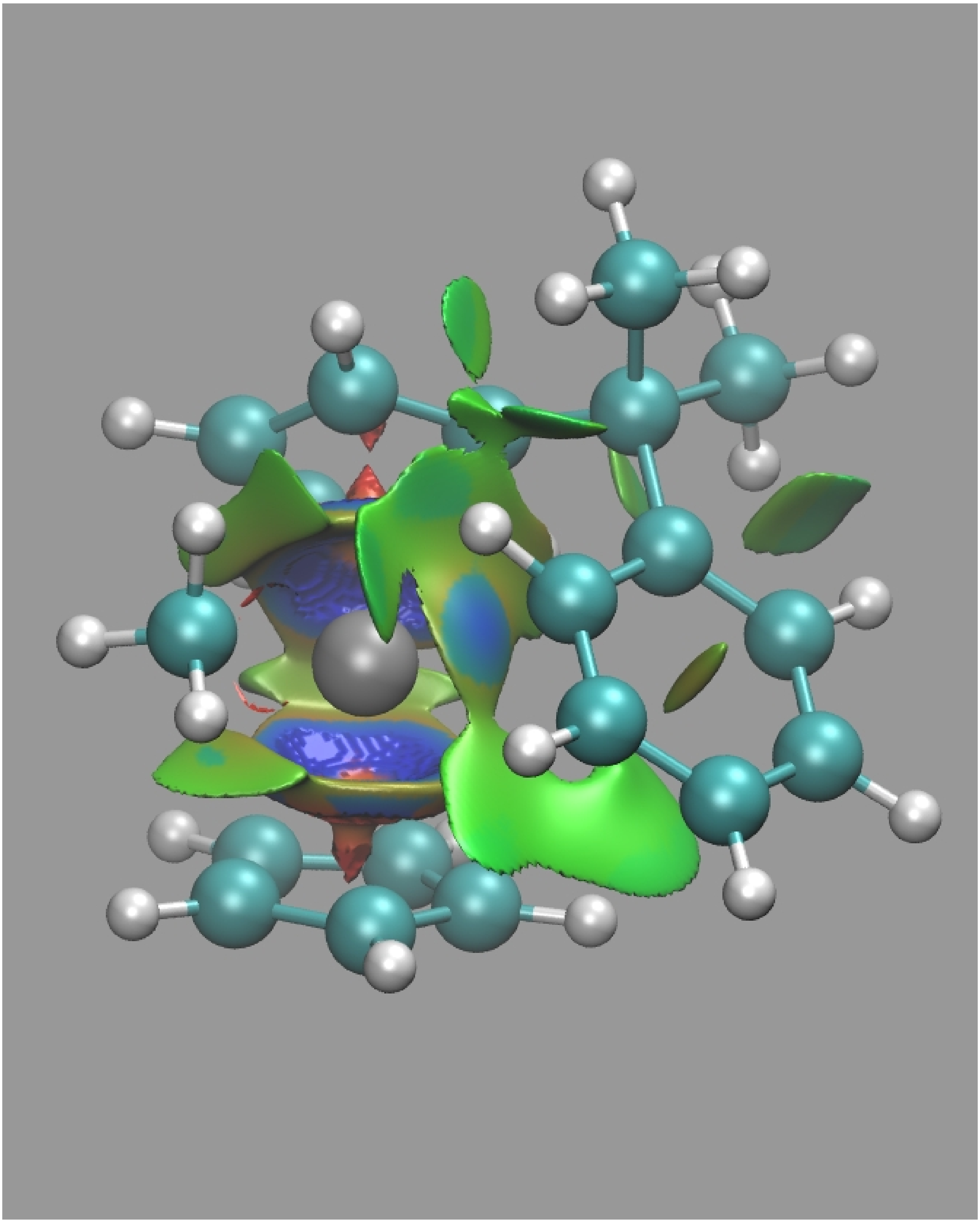

IIB-B3LYP

Bader:

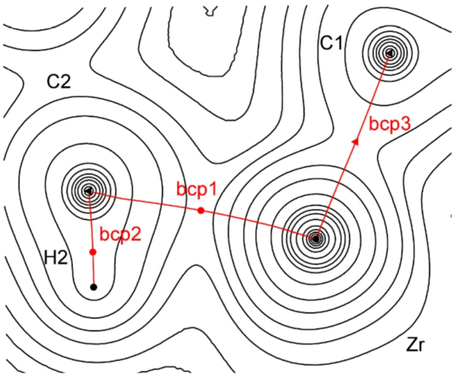

Electrondensity

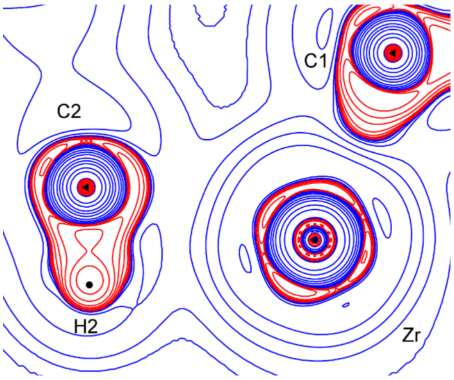

Laplacian

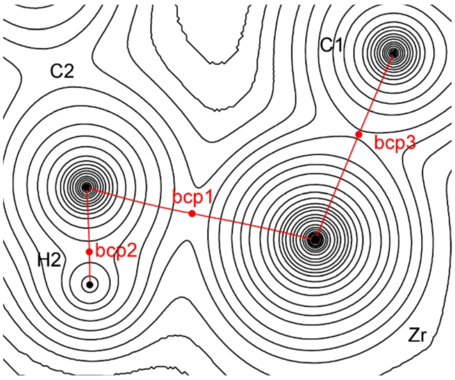

Virial

	$\varrho(\mathbf{r})$	$\nabla^2\varrho(\mathbf{r})$
bcp1	0.03299	-0.02099
bcp2	0.27015	0.21998
bcp3	0.09639	-0.01153

NBO:

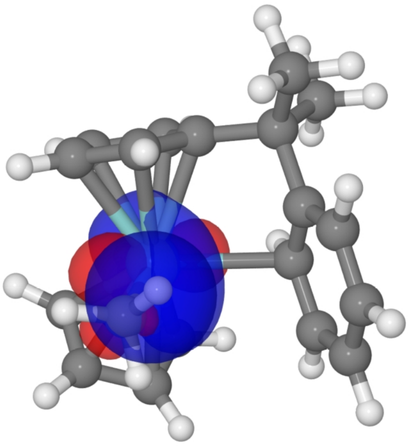

1

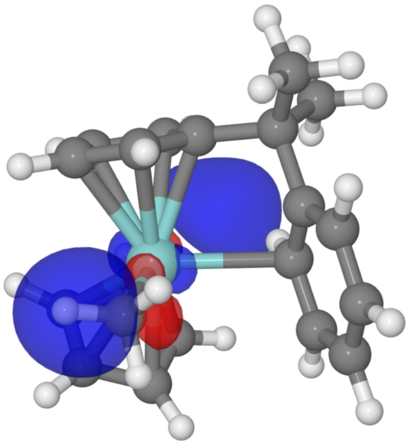

2

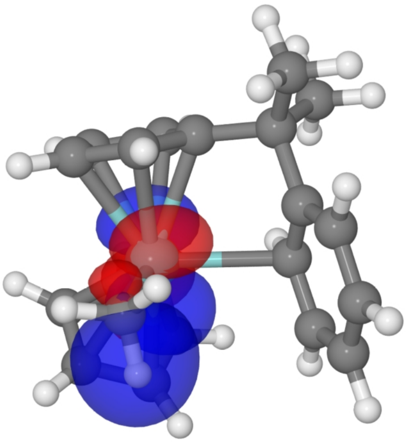

3

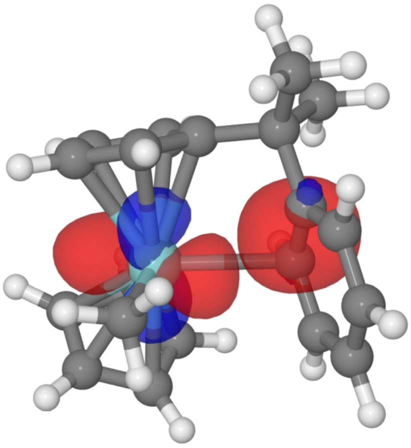

4

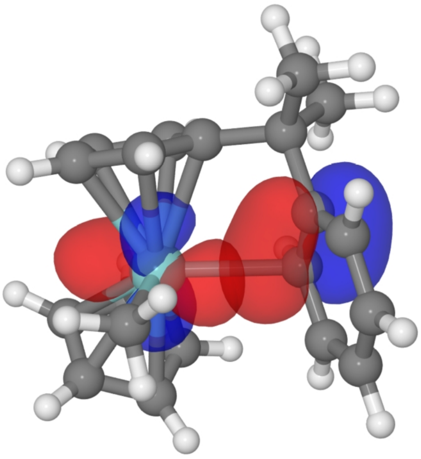

5

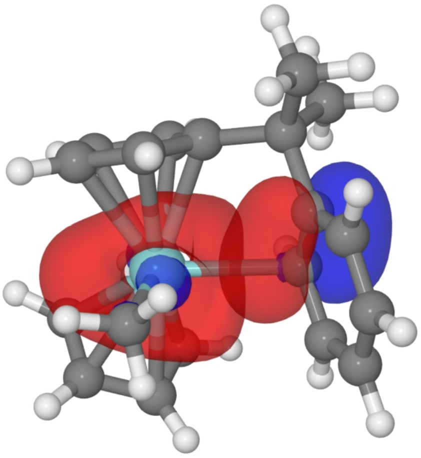

6

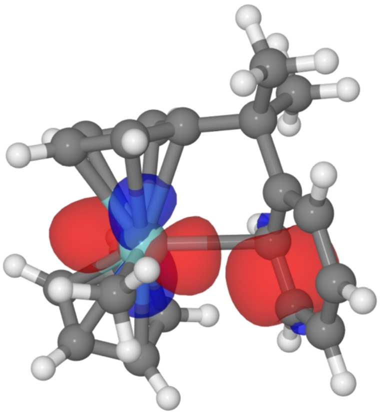

7

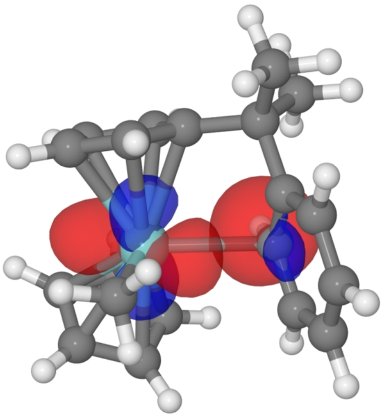

8

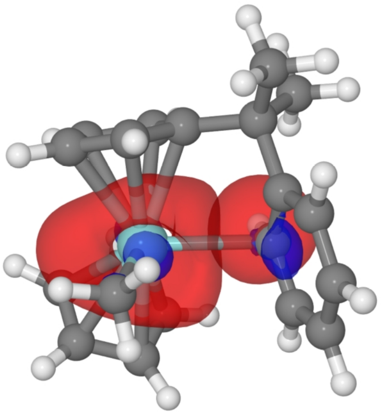

9

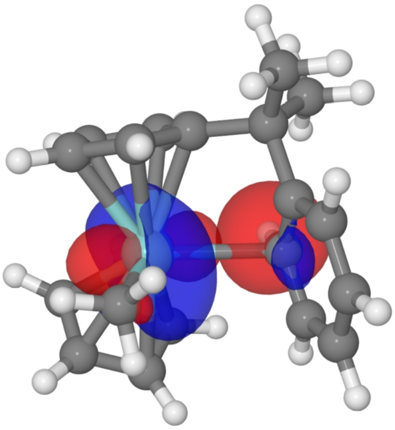

10

	Orbitals	E(2P)
1	σ_{CH} = 0.776$(sp^{2.97})_{C7}$ – 0.630$(s)_{H42}$ → LV_{Zr} = $sp^{0.03}d^{9.24}$	2.87
2	σ_{CH} = 0.781$(sp^{3.12})_{C7}$ – 0.624$(s)_{H43}$ → RY_{Zr} = $sp^{2.45}d^{48.22}$	2.12
3	σ_{CH} = 0.777$(sp^{2.93})_{C7}$ – 0.630$(s)_{H44}$ → LV_{Zr} = $p^{0.74}d^{99.99}$	2.41
4	σ_{CC} = 0.711$(sp^{1.72})_{C15}$ – 0.703$(sp^{2.04})_{C16}$ → LV_{Zr} = $sp^{0.06}d^{50.70}$	2.49
5	π_{CC} = 0.773$(sp^{99.99})_{C15}$ – 0.635$(sp^{99.99}d^{0.83})_{C16}$ → LV_{Zr} = $sp^{0.06}d^{50.70}$	9.10
6	π_{CC} = 0.773$(sp^{99.99})_{C15}$ – 0.635$(sp^{99.99}d^{0.83})_{C16}$ → LV_{Zr} = $sd^{0.36}$	7.62
7	σ_{CC} = 0.715$(sp^{1.79})_{C15}$ – 0.699$(sp^{1.78})_{C20}$ → LV_{Zr} = $sp^{0.06}d^{50.70}$	2.87
8	σ_{CH} = 0.784$(sp^{2.71})C_{15}$ – 0.620$(s)_{H29}$ → LV_{Zr} = $sp^{0.06}d^{50.70}$	2.72
9	σ_{CH} = 0.784$(sp^{2.71})C_{15}$ – 0.620$(s)_{H29}$ → LV_{Zr} = $sd^{0.36}$	5.83
10	σ_{CH} = 0.784$(sp^{2.71})C_{15}$ – 0.620$(s)_{H29}$ → LV_{Zr} = $sp^{0.03}d^{9.24}$	2.99

Natural Resonance Theory:

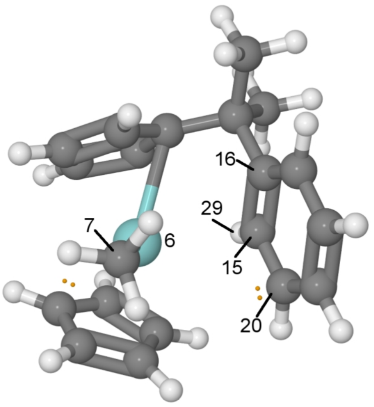

1

Wgt=15.92%;
rhoNL=5.69288;
D(0)=0.10326

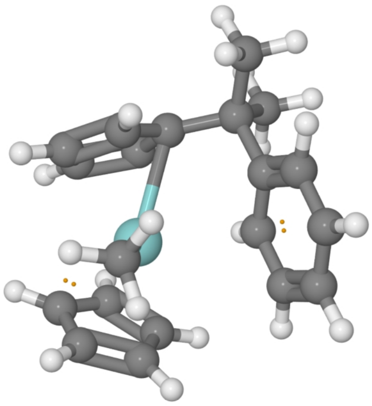

2

Wgt=14.54%;
rhoNL=5.47160;
D(0)=0.10123

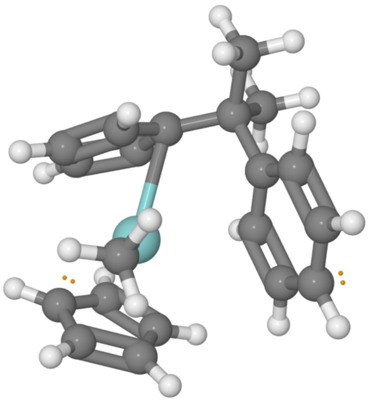

3

Wgt=13.37%;
rhoNL=5.62498;
D(0)=0.10264

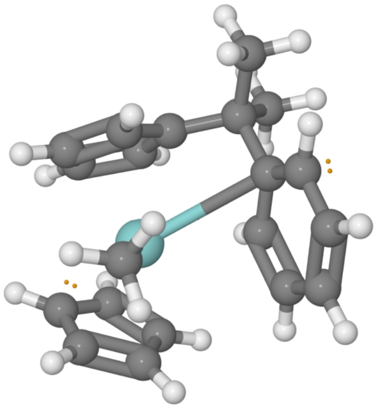

4

Wgt=12.95%;
rhoNL=5.91034;
D(0)=0.10521

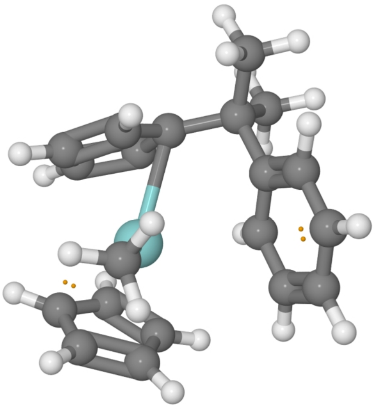

5

Wgt=12.53%;
rhoNL=5.73513;
D(0)=0.10364

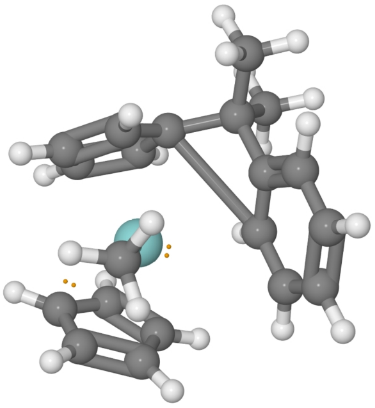

6

Wgt=9.54%;
rhoNL=6.41223;
D(0)=0.10959

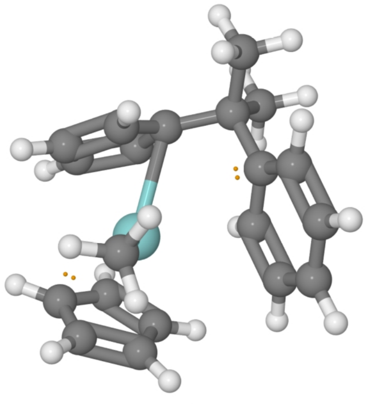

7

Wgt=9.50%;
rhoNL=5.66729;
D(0)=0.1030

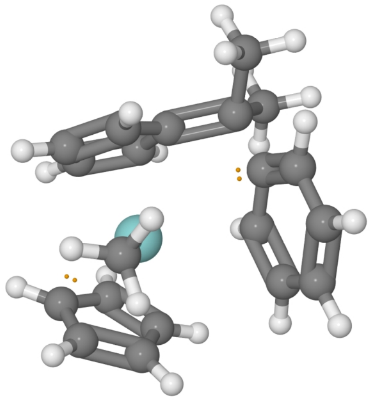

8

Wgt=6.59%;
rhoNL=6.03165;
D(0)=0.10629

Natural Localised Molecular Orbitals (NLMO):

Only contributions over 1% are reported.

NLMO / Occupancy / Percent from Parent NBO / Atomic Hybrid Contributions

Resonance structure **1**:
C-H interaction:
79. (2.00000) 97.2919% BD (1) C 15- H 29
1.499% Zr 6 s(22.63%)p 0.03(0.58%)d 3.39(76.79%)
59.964% C 15 s(24.97%)p 3.00(74.92%)d 0.00(0.11%)
37.380% H 29 s(99.95%)p 0.00(0.05%)

C-C interaction:
77. (2.00000) 83.2789% BD (2) C 15- C 16
2.906% Zr 6 s(27.79%)p 0.03(0.93%)d 2.56(71.28%)
49.635% C 15 s(0.76%)p99.99(99.22%)d 0.03(0.02%)
33.663% C 16 s(0.09%)p99.99(99.86%)d 0.52(0.05%)
5.031% C 17 s(0.00%)p 1.00(99.68%)d 0.00(0.32%)
1.043% C 18 s(0.08%)p99.99(99.56%)d 4.38(0.36%)
1.797% C 19 s(0.10%)p99.99(99.76%)d 1.33(0.14%)
4.905% C 20 s(0.01%)p 1.00(99.65%)d 0.00(0.35%)

Zr-Me interaction:
53. (2.00000) 97.8283% BD (1)Zr 6- C 7
20.128% Zr 6 s(12.77%)p 0.01(0.08%)d 6.82(87.15%)
77.709% C 7 s(25.67%)p 2.90(74.32%)d 0.00(0.02%)

Resonance structure **2**:
C-H interaction:
78. (2.00000) 97.3244% BD (1) C 15- H 29
1.551% Zr 6 s(23.00%)p 0.02(0.56%)d 3.32(76.43%)
60.032% C 15 s(25.07%)p 2.98(74.82%)d 0.00(0.11%)
37.326% H 29 s(99.95%)p 0.00(0.05%)

Zr-Me interaction:
53. (2.00000) 97.8288% BD (1)Zr 6- C 7
20.127% Zr 6 s(12.78%)p 0.01(0.08%)d 6.82(87.14%)
77.711% C 7 s(25.67%)p 2.90(74.32%)d 0.00(0.02%)

Resonance structure **3**:
C-H interaction:
79. (2.00000) 97.2891% BD (1) C 15- H 29
1.488% Zr 6 s(22.51%)p 0.03(0.59%)d 3.42(76.91%)
59.951% C 15 s(24.95%)p 3.00(74.94%)d 0.00(0.11%)
37.392% H 29 s(99.95%)p 0.00(0.05%)

C-C interaction:
78. (2.00000) 84.5054% BD (2) C 15- C 20
2.940% Zr 6 s(29.93%)p 0.03(0.97%)d 2.31(69.11%)

51.331% C 15 s(0.78%)p99.99(99.20%)d 0.02(0.02%)
5.471% C 16 s(0.13%)p99.99(99.56%)d 2.53(0.32%)
1.390% C 17 s(0.10%)p99.99(99.68%)d 2.10(0.22%)
1.232% C 18 s(0.08%)p99.99(99.69%)d 3.09(0.24%)
3.858% C 19 s(0.01%)p99.99(99.61%)d29.98(0.38%)
33.220% C 20 s(0.01%)p99.99(99.94%)d 4.11(0.05%)

Zr-Me interaction:
53. (2.00000) 97.8283% BD (1)Zr 6- C 7
20.129% Zr 6 s(12.77%)p 0.01(0.08%)d 6.83(87.16%)
77.709% C 7 s(25.67%)p 2.90(74.32%)d 0.00(0.02%)

Resonance structure **4**:
C-H interaction:
79. (2.00000) 97.2879% BD (1) C 15- H 29
1.479% Zr 6 s(21.88%)p 0.03(0.59%)d 3.54(77.53%)
59.961% C 15 s(24.98%)p 3.00(74.91%)d 0.00(0.11%)
37.380% H 29 s(99.95%)p 0.00(0.05%)

C-C interaction:
78. (2.00000) 83.5147% BD (2) C 15- C 20
3.183% Zr 6 s(27.85%)p 0.03(0.94%)d 2.56(71.21%)
53.992% C 15 s(0.78%)p99.99(99.21%)d 0.02(0.01%)
7.592% C 16 s(0.11%)p99.99(99.65%)d 2.18(0.24%)
1.637% C 18 s(0.08%)p99.99(99.81%)d 1.54(0.12%)
2.335% C 19 s(0.02%)p99.99(99.41%)d25.37(0.57%)
29.801% C 20 s(0.01%)p99.99(99.94%)d 5.15(0.05%)

Zr-C interaction:
54. (2.00000)   4.1992% BD (1)Zr 6- C 16
6.688% Zr 6 s(0.84%)p 0.30(0.25%)d99.99(98.92%)
13.396% C 8 s(0.41%)p99.99(99.34%)d 0.63(0.26%)
42.877% C 9 s(0.40%)p99.99(99.51%)d 0.21(0.09%)
13.947% C 10 s(0.40%)p99.99(99.36%)d 0.61(0.24%)
1.455% C 11 s(0.82%)p99.99(98.43%)d 0.91(0.74%)
2.106% C 12 s(1.00%)p98.16(98.59%)d 0.40(0.40%)
4.138% C 16 s(1.39%)p70.91(98.29%)d 0.24(0.33%)
10.178% C 17 s(0.08%)p99.99(99.88%)d 0.50(0.04%)
2.020% C 18 s(0.16%)p99.99(99.56%)d 1.78(0.28%)
1.025% C 20 s(1.42%)p69.35(98.46%)d 0.08(0.12%)

Zr-Me interaction:
53. (2.00000) 97.8265% BD (1)Zr 6- C 7
20.161% Zr 6 s(12.81%)p 0.01(0.08%)d 6.80(87.12%)
77.675% C 7 s(25.67%)p 2.89(74.31%)d 0.00(0.02%)

Resonance structure **5**, **6**:
NLMO algorithm failed to converge

Resonance structure **7**:
C-H interaction:
79. (2.00000) 97.2881% BD (1) C 15- H 29
1.494% Zr 6 s(22.46%)p 0.03(0.58%)d 3.43(76.96%)
59.956% C 15 s(24.95%)p 3.00(74.94%)d 0.00(0.11%)
37.386% H 29 s(99.95%)p 0.00(0.05%)

C-C interaction:
78. (2.00000) 84.6061% BD (2) C 15- C 20
2.874% Zr 6 s(30.00%)p 0.03(0.96%)d 2.30(69.03%)
50.334% C 15 s(0.78%)p99.99(99.20%)d 0.02(0.02%)
4.866% C 16 s(0.13%)p99.99(99.52%)d 2.71(0.35%)
1.540% C 17 s(0.09%)p99.99(99.73%)d 1.86(0.18%)
1.076% C 18 s(0.08%)p99.99(99.61%)d 3.71(0.31%)
4.477% C 19 s(0.01%)p99.99(99.65%)d30.43(0.34%)
34.295% C 20 s(0.01%)p99.99(99.95%)d 3.89(0.04%)

Zr-Me interaction:
53. (2.00000) 97.8281% BD (1)Zr 6- C 7
20.128% Zr 6 s(12.77%)p 0.01(0.08%)d 6.83(87.16%)
77.710% C 7 s(25.67%)p 2.90(74.32%)d 0.00(0.02%)

Resonance structure **8**:
C-H interaction:
78. (2.00000) 97.2926% BD (1) C 15- H 29
1.479% Zr 6 s(22.25%)p 0.03(0.59%)d 3.47(77.16%)
59.950% C 15 s(24.97%)p 3.00(74.92%)d 0.00(0.11%)
37.398% H 29 s(99.95%)p 0.00(0.05%)

C-C interaction:
77. (2.00000) 84.5133% BD (2) C 15- C 20
2.826% Zr 6 s(28.68%)p 0.03(0.99%)d 2.45(70.32%)
50.374% C 15 s(0.76%)p99.99(99.22%)d 0.02(0.02%)
4.886% C 16 s(0.13%)p99.99(99.52%)d 2.77(0.35%)
1.606% C 17 s(0.11%)p99.99(99.73%)d 1.60(0.17%)
1.137% C 18 s(0.09%)p99.99(99.62%)d 3.29(0.29%)
4.413% C 19 s(0.01%)p99.99(99.65%)d33.13(0.34%)
34.160% C 20 s(0.01%)p99.99(99.95%)d 4.01(0.04%)

Zr-Me interaction:
53. (2.00000) 97.8301% BD (1)Zr 6- C 7
20.137% Zr 6 s(12.81%)p 0.01(0.08%)d 6.80(87.11%)
77.702% C 7 s(25.67%)p 2.90(74.32%)d 0.00(0.02%)

Non-Covalent Interactions (NCI)

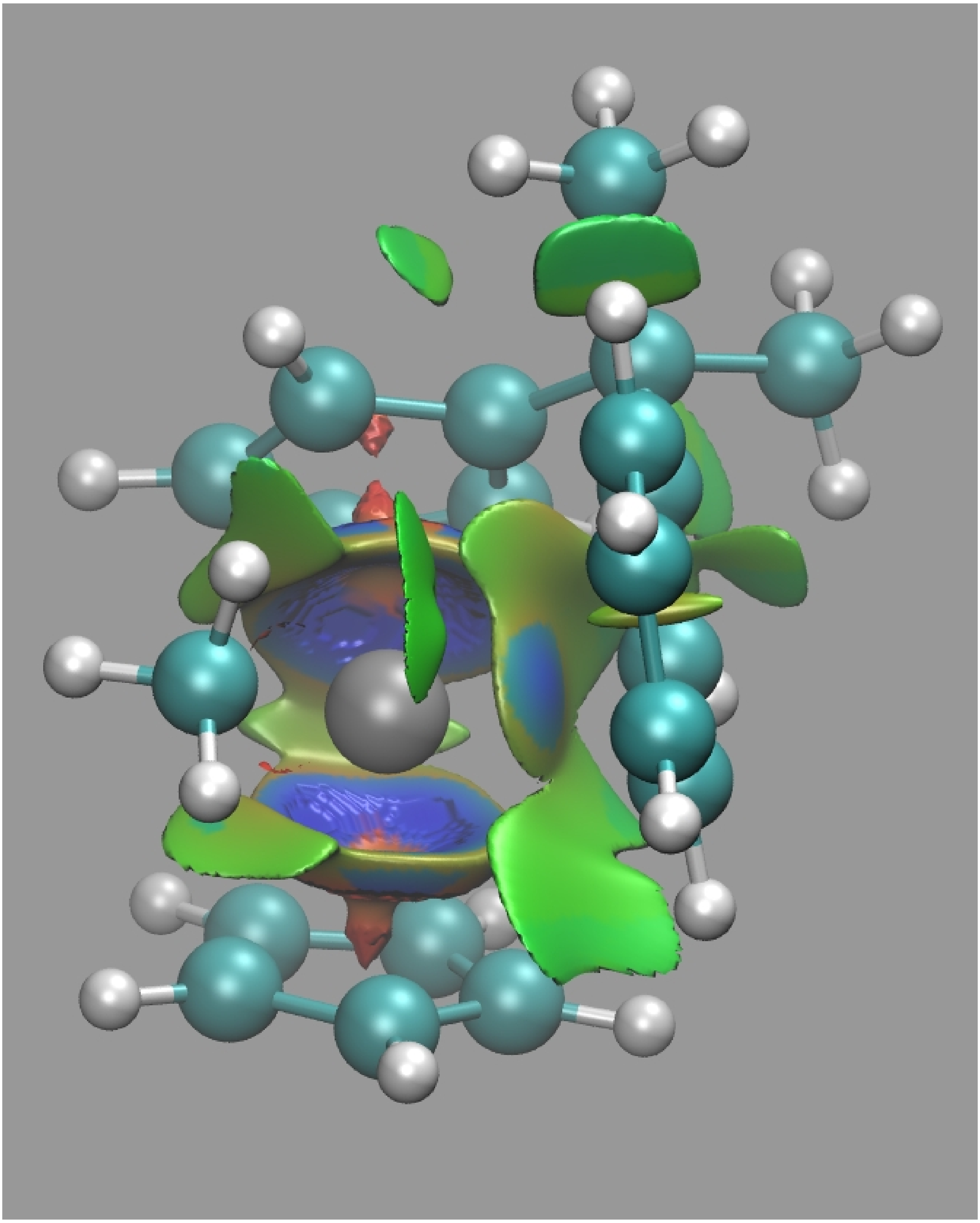

1A-B3LYP

Bader:

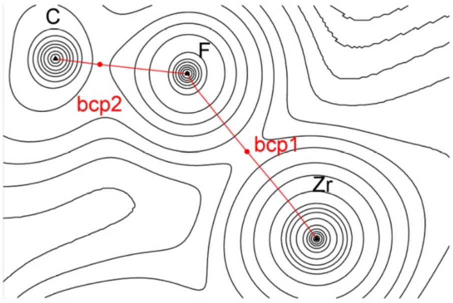

Electrondensity

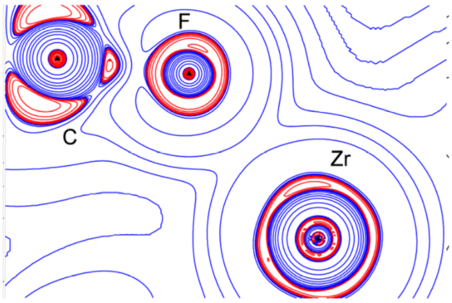

Laplacian

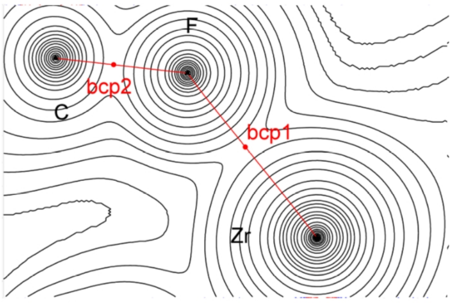

Virial

	$\varrho(\mathbf{r})$	$\nabla^2\varrho(\mathbf{r})$
bcp1	0.04540	-0.06470
bcp2	0.18903	-0.03340
bcp3	0.09624	-0.01162

NBO:

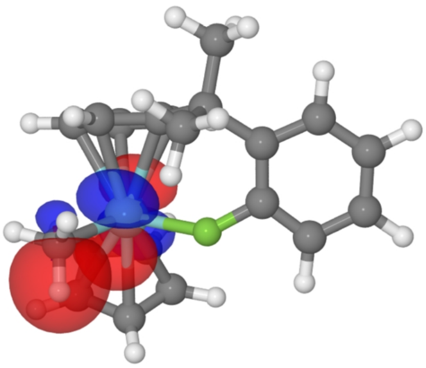
1

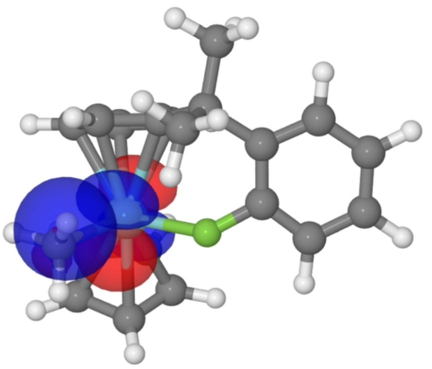
2

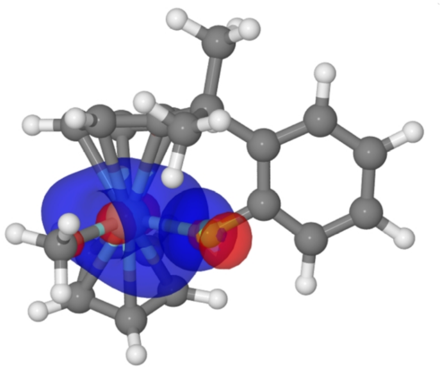
3

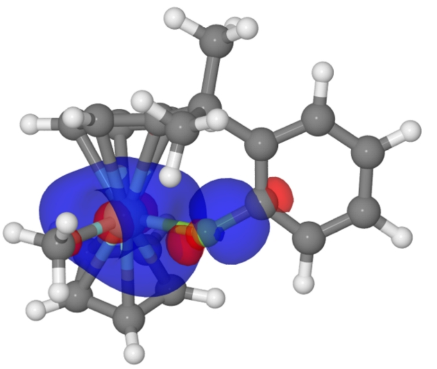
4

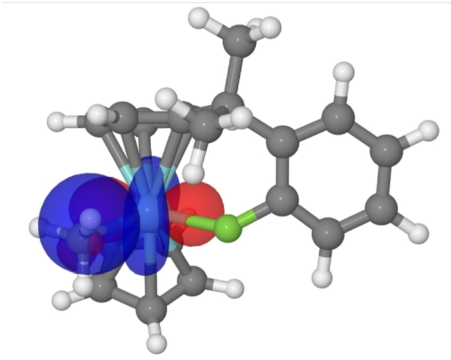

5

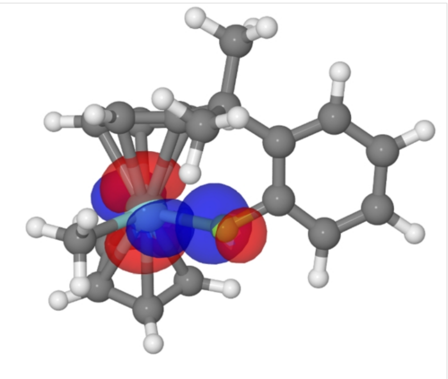

6

	Orbitals	E(2P)
1	σ_{CH} = 0.777$(sp^{2.91})_{C21}$ – 0.629$(s)_{H43}$ → LV_{Zr} = $pd^{99.99}$	2.27
2	σ_{CH} = 0.776$(sp^{2.95})_{C21}$ – 0.630$(s)_{H44}$ → LV_{Zr} = $pd^{99.99}$	2.06
3	LP_F = $sp^{2.36}$ → LV_{Zr} = $sp^{0.20}$	20.97
4	σ_{CF} = 0.476$(sp^{5.16})_{C1}$ – 0.880$(sp^{2.33})_F$ → LV_{Zr} = $sp^{0.20}$	2.12
5	σ_{CH} = 0.776$(sp^{2.95})_{C21}$ – 0.630$(s)_{H44}$ → LV_{Zr} = $sp^{0.18}d^{72.08}$	2.02
6	LP_F = $sp^{2.36}$ → LV_{Zr} = $sp^{0.97}d^{99.99}$	5.04

Natural Resonance Theory:

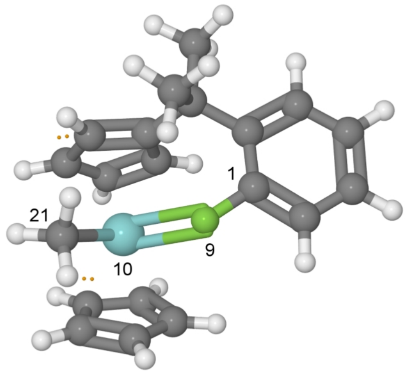

1

Wgt=29.14%;
rhoNL=5.11602;
D(0)=0.09681

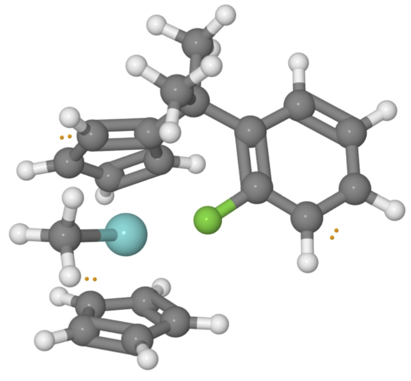

2

Wgt=9.54%;
rhoNL=5.83063;
D(0)=0.10335

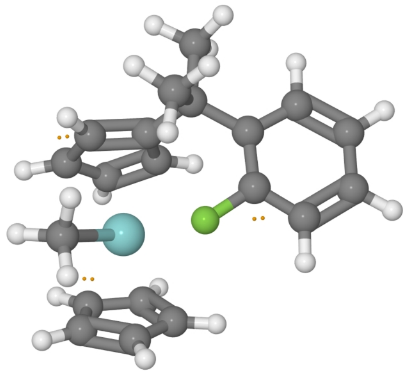

3

Wgt=8.79%;
rhoNL=5.80480;
D(0)=0.10312

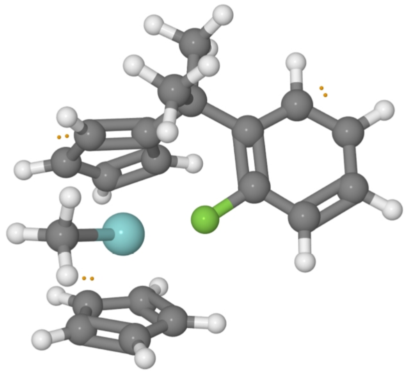

4

Wgt=6.53%;
rhoNL=5.85701;
D(0)=0.10358

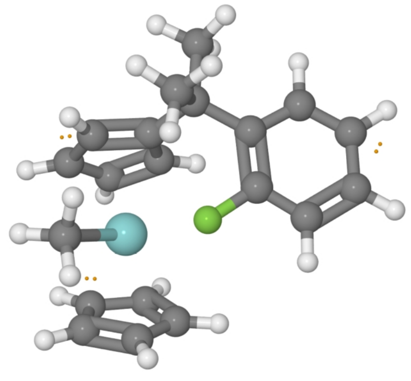

5

Wgt=5.56%;
rhoNL=5.86676;
D(0)=0.10367

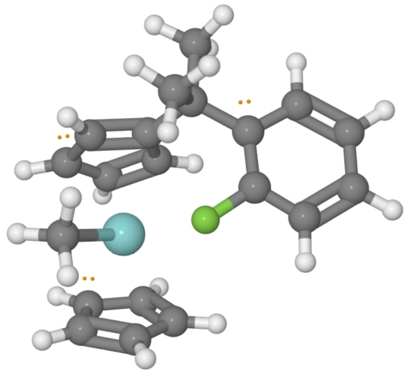

6

Wgt=5.52%;
rhoNL=5.86989;
D(0)=0.10369

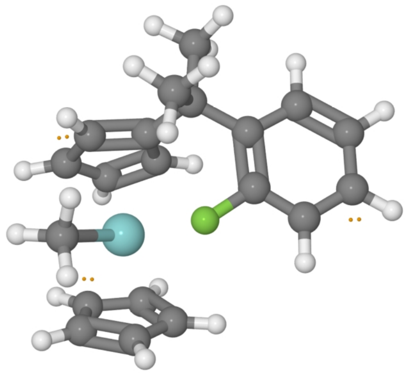

7

Wgt=5.17%;
rhoNL=5.87330;
D(0)=0.10372

Natural Localised Molecular Orbitals (NLMO):

Only contributions over 1% are reported.

NLMO / Occupancy / Percent from Parent NBO / Atomic Hybrid Contributions

Resonance structure **1**:
Zr-F interaction:

```
 64. (2.00000)  99.2386% BD ( 1) F  9-Zr 10
                 96.655%  F  9 s( 47.21%)p 1.12( 52.79%)d 0.00(  0.00%)
                  2.716% Zr 10 s( 38.50%)p 0.01(  0.22%)d 1.59( 61.28%)

 65. (2.00000)  97.9641% BD ( 2) F  9-Zr 10
                 96.969%  F  9 s(  0.15%)p99.99( 99.84%)d 0.04(  0.01%)
                  1.142% Zr 10 s( 11.28%)p 0.10(  1.18%)d 7.76( 87.54%)
```

C-F interaction:

```
 46. (2.00000)  99.6980% BD ( 1) C  1- F  9
                 22.242%  C  1 s( 17.38%)p 4.73( 82.25%)d 0.02(  0.37%)
                 77.468%  F  9 s( 38.55%)p 1.59( 61.42%)d 0.00(  0.02%)
```

Zr-Me interaction:

```
 66. (2.00000)  97.8941% BD ( 1)Zr 10- C 21
                 21.238% Zr 10 s( 13.64%)p 0.01(  0.07%)d 6.33( 86.30%)
                 76.694%  C 21 s( 25.40%)p 2.94( 74.58%)d 0.00(  0.02%)
```

Resonance structur **2**:
C-F interaction:

```
 49. (2.00000)  99.6416% BD ( 1) C  1- F  9
                 22.507%  C  1 s( 17.91%)p 4.56( 81.72%)d 0.02(  0.38%)
                 77.149%  F  9 s( 30.06%)p 2.33( 69.92%)d 0.00(  0.02%)
```

Zr-Me interaction:

```
 66. (2.00000)  97.9214% BD ( 1)Zr 10- C 21
                 21.232% Zr 10 s( 13.59%)p 0.01(  0.07%)d 6.35( 86.34%)
                 76.699%  C 21 s( 25.39%)p 2.94( 74.60%)d 0.00(  0.02%)
```

Resonance structur **3**:
C-F interaction:

```
 48. (2.00000)  99.6438% BD ( 1) C  1- F  9
                 22.511%  C  1 s( 17.90%)p 4.57( 81.73%)d 0.02(  0.38%)
                 77.146%  F  9 s( 30.06%)p 2.33( 69.92%)d 0.00(  0.02%)
```

Zr-Me interaction:

```
 66. (2.00000)  97.9214% BD ( 1)Zr 10- C 21
                 21.232% Zr 10 s( 13.59%)p 0.01(  0.07%)d 6.35( 86.34%)
                 76.699%  C 21 s( 25.39%)p 2.94( 74.60%)d 0.00(  0.02%)
```

Resonance structur **4**:
C-F interaction:
49. (2.00000) 99.6416% BD (1) C 1- F 9
22.507% C 1 s(17.91%)p 4.56(81.72%)d 0.02(0.38%)
77.149% F 9 s(30.06%)p 2.33(69.92%)d 0.00(0.02%)

Zr-Me interaction:
66. (2.00000) 97.9214% BD (1)Zr 10- C 21
21.232% Zr 10 s(13.59%)p 0.01(0.07%)d 6.35(86.34%)
76.699% C 21 s(25.39%)p 2.94(74.60%)d 0.00(0.02%)

Resonance structur **5**:
NLMO algorithm failed to converge

Resonance structur **6**:
C-F interaction:
48. (2.00000) 99.6426% BD (1) C 1- F 9
22.508% C 1 s(17.90%)p 4.56(81.72%)d 0.02(0.38%)
77.148% F 9 s(30.06%)p 2.33(69.92%)d 0.00(0.02%)

Zr-Me interaction:
21.231% Zr 10 s(13.59%)p 0.01(0.07%)d 6.35(86.34%)
76.699% C 21 s(25.39%)p 2.94(74.60%)d 0.00(0.02%)

Resonance structur **7**:
NLMO algorithm failed to converge

Non-Covalent Interactions (NCI)

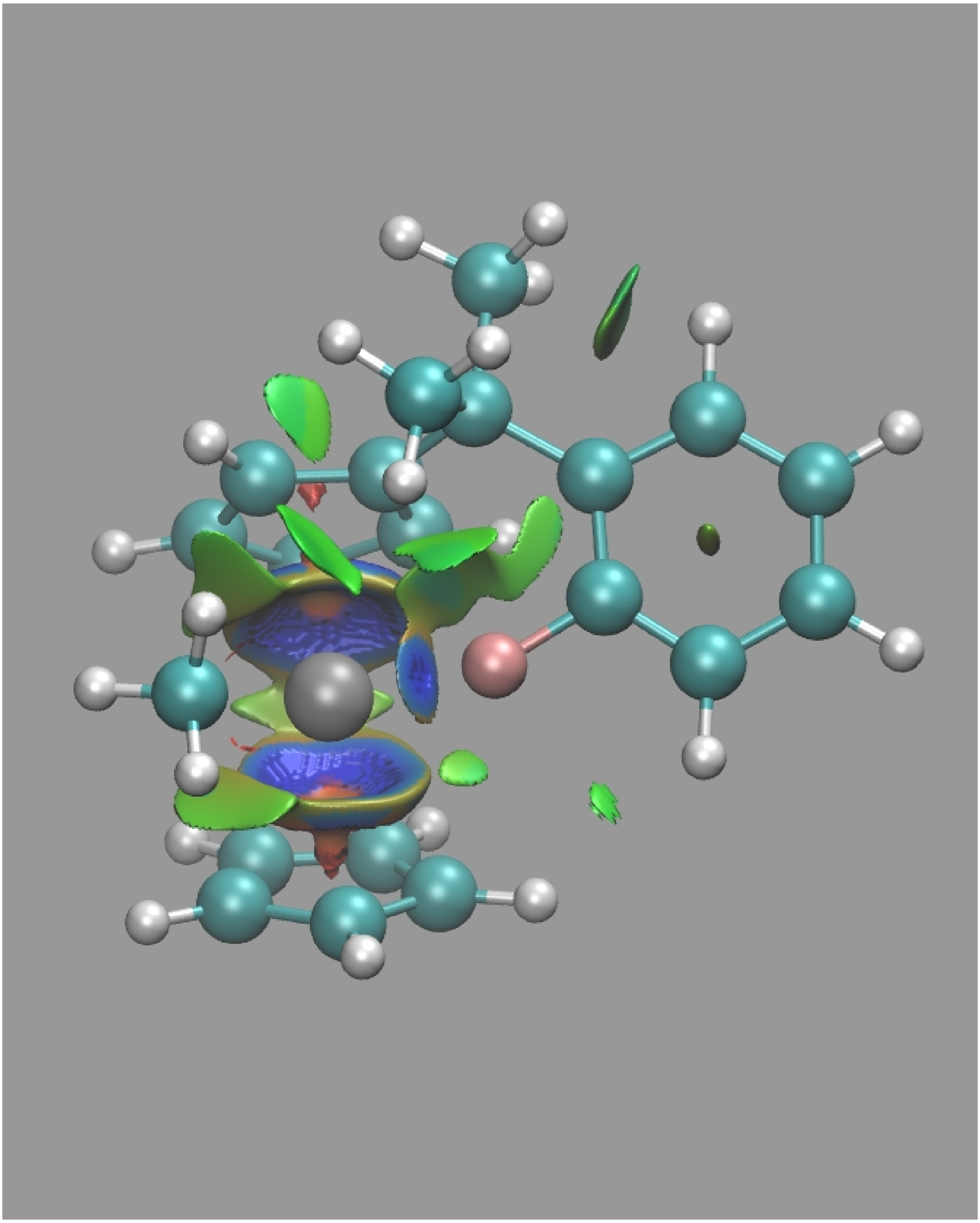

1B-B3LYP

Bader:

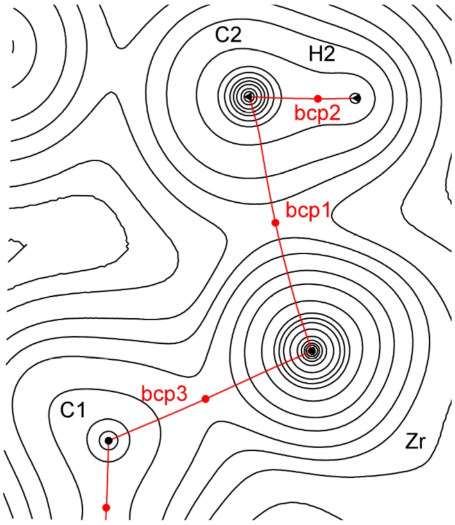

Electrondensity

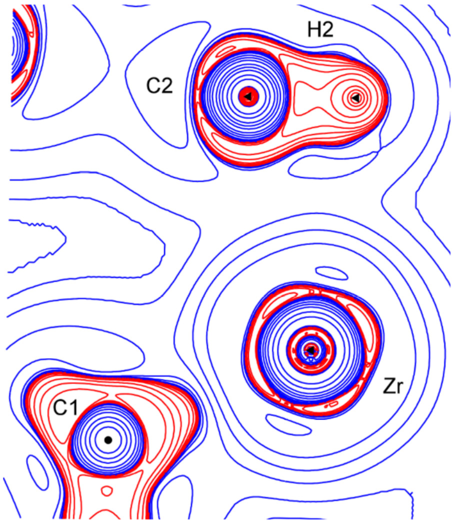

Laplacian

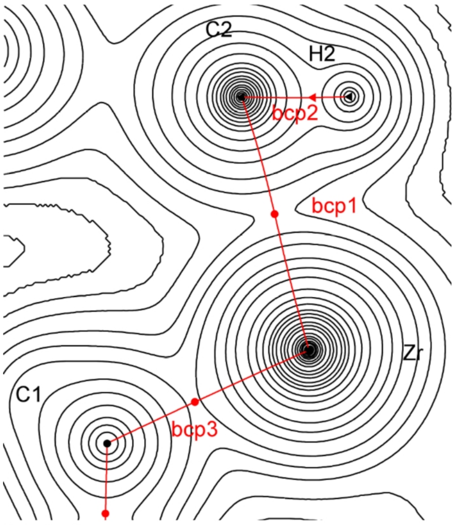

Virial

	ϱ(**r**)	∇^2ϱ(**r**)
bcp1	0.03095	-0.02028
bcp2	0.27156	0.22242
bcp3	0.09650	-0.01155

NBO:

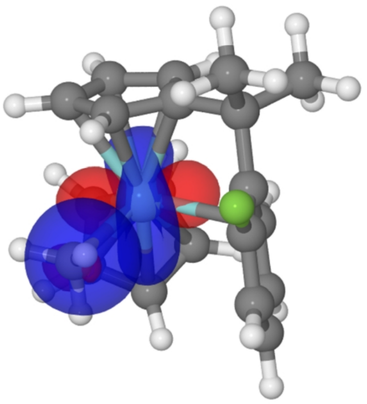

1

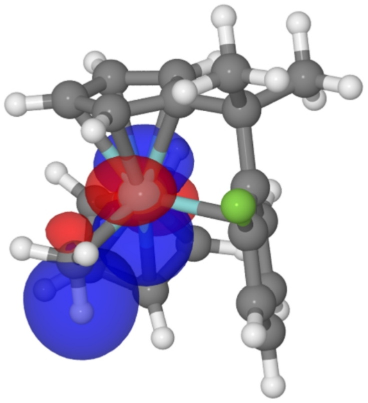

2

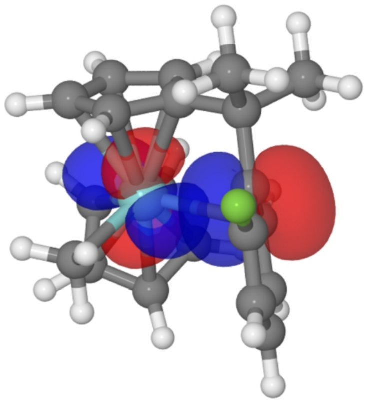

3

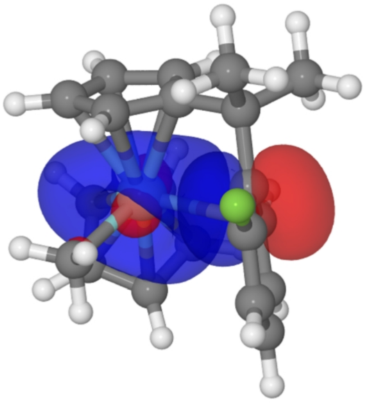

4

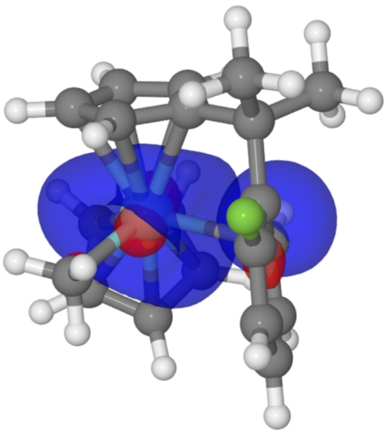

5

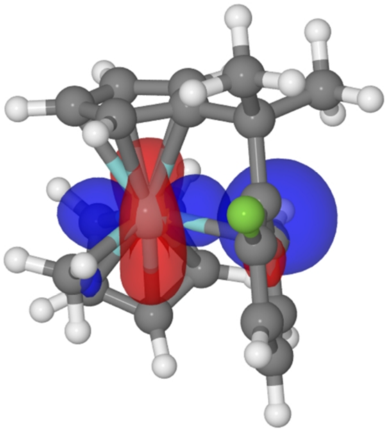

6

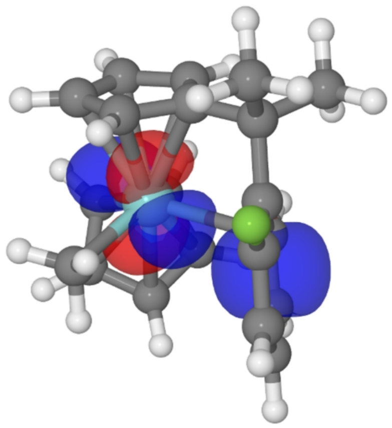

7

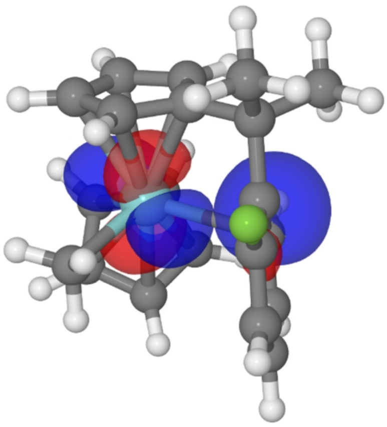

8

	Orbitals	E(2P)
1	$\sigma_{CH} = 0.776(sp^{2.96})_{C7} – 0.630(s)_{H37} \rightarrow$ $LV_{Zr} = sd^{12.04}$	2.72
2	$\sigma_{CH} = 0.775(sp^{2.93}) – 0.630(s)_{H39} \rightarrow$ $LV_{Zr} = sp^{0.35}d^{99.99}$	2.46
3	$\pi_{CC} = 0.759(sp^{99.99})_{C15} - 0.651(sp^{99.99})_{C16} \rightarrow$ $LV_{Zr} = sd^{66.87}$	7.81
4	$\pi_{CC} = 0.759(sp^{99.99})_{C15} - 0.651(sp^{99.99})_{C16} \rightarrow$ $LV_{Zr} = sd^{0.31}$	7.08
5	$\sigma_{CH} = 0.785(sp^{2.71})_{C15} – 0.620(s)_{H26} \rightarrow$ $LV_{Zr} = sd^{0.31}$	5.33
6	$\sigma_{CH} = 0.785(sp^{2.71})_{C15} – 0.620(s)_{H26} \rightarrow$ $LV_{Zr} = sd^{12.04}$	3.22
7	$\sigma_{CC} = 0.715(sp^{1.75})_{C15} – 0.699(sp^{1.78})_{C20} \rightarrow$ $LV_{Zr} = sd^{66.87}$	2.54
8	$\sigma_{CH} = 0.785(sp^{2.71})_{C15} – 0.620(s)_{H26} \rightarrow$ $LV_{Zr} = sd^{66.87}$	2.30

Natural Resonance Theory:

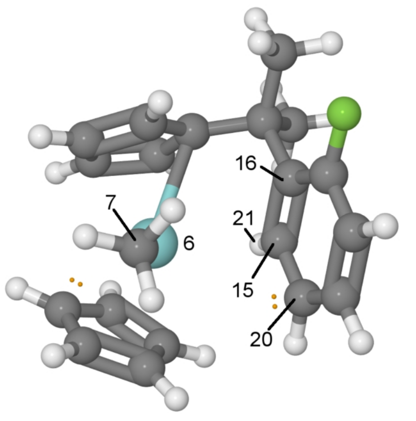

1

Wgt=16.10%;
rhoNL=5.75159;
D(0)=0.10264

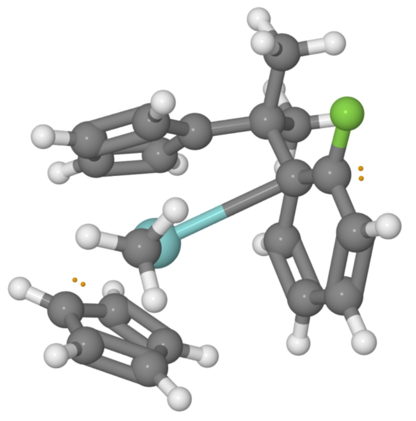

2

Wgt=14.55%;
rhoNL=5.95000;
D(0)=0.10440

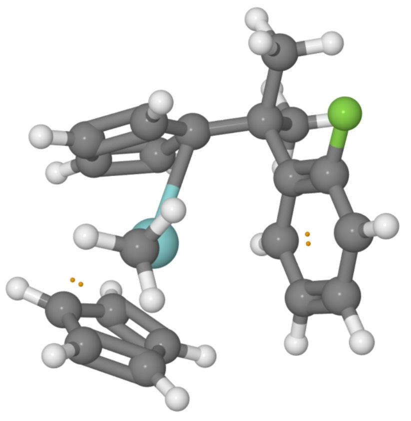

3

Wgt=14.11%;
rhoNL=5.61260;
D(0)=0.10140

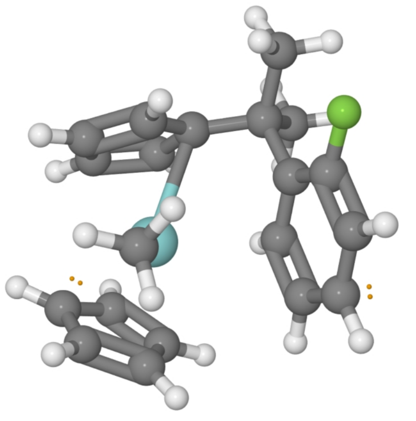

4

Wgt=13.17%;
rhoNL=5.74460;
D(0)=0.10258

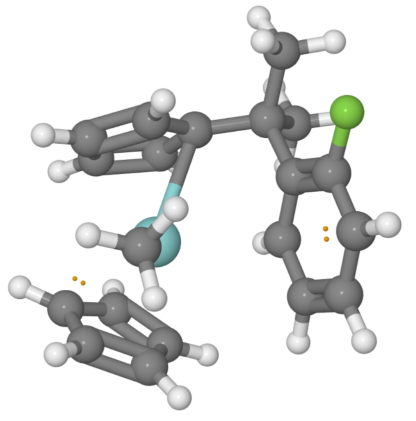

5

Wgt=12.09%;
rhoNL=5.82075;
D(0)=0.10326

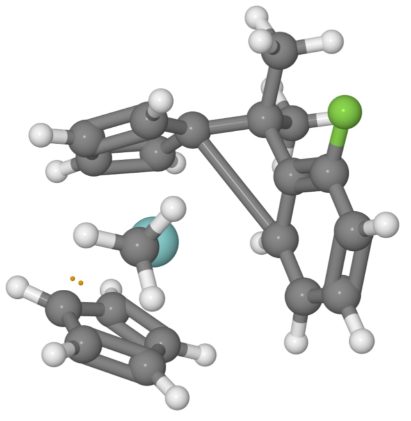

6

Wgt=8.83%;
rhoNL=6.51046;
D(0)=0.10921

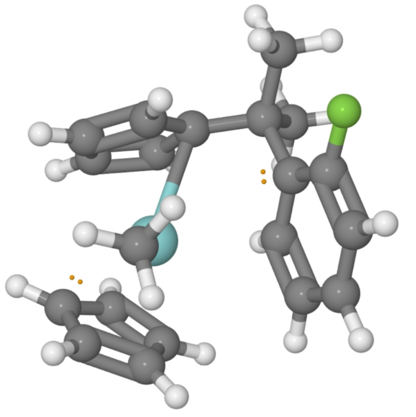

7

Wgt=8.68%;
rhoNL=5.74029;
D(0)=0.10254

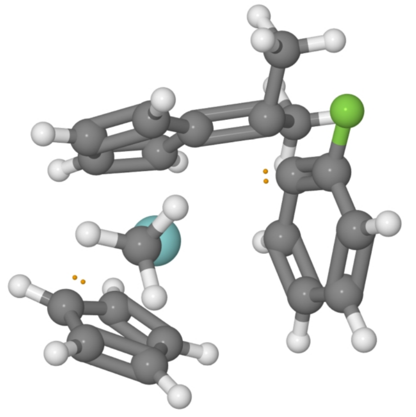

8

Wgt=6.18%;
rhoNL=6.10263;
D(0)=0.10573

Natural Localised Molecular Orbitals (NLMO):

Only contributions over 1% are reported.

NLMO / Occupancy / Percent from Parent NBO / Atomic Hybrid Contributions

Resonance structure **1**:
C-H interaction:

```
 83. (2.00000)  97.3550% BD ( 1) C 15- H 26
                 1.421% Zr  6 s( 22.60%)p 0.03(  0.57%)d 3.40( 76.82%)
                60.099%  C 15 s( 25.08%)p 2.98( 74.82%)d 0.00(  0.11%)
                37.305%  H 26 s( 99.95%)p 0.00(  0.05%)
```

Zr-Me interaction:

```
  57. (2.00000)  97.8329% BD ( 1)Zr  6- C  7
                20.111% Zr  6 s( 12.64%)p 0.01(  0.08%)d 6.90( 87.28%)
                77.731%  C  7 s( 25.66%)p 2.90( 74.32%)d 0.00(  0.02%)
```

Resonance structure **2**:
C-H interaction:

```
 83. (2.00000)  97.3537% BD ( 1) C 15- H 26
                 1.387% Zr  6 s( 22.16%)p 0.03(  0.60%)d 3.49( 77.24%)
                60.078%  C 15 s( 25.02%)p 2.99( 74.87%)d 0.00(  0.11%)
                37.324%  H 26 s( 99.95%)p 0.00(  0.05%)
```

C-C interaction:

```
 82. (2.00000)  82.2409% BD ( 2) C 15- C 20
                 3.856% Zr  6 s( 18.14%)p 0.03(  0.63%)d 4.48( 81.23%)
                 1.028%  C  8 s(  0.29%)p99.99( 99.36%)d 1.21(  0.35%)
                47.866%  C 15 s(  0.73%)p99.99( 99.25%)d 0.03(  0.02%)
                 2.832%  C 16 s(  0.10%)p99.99( 99.33%)d 5.64(  0.57%)
                 2.468%  C 17 s(  0.07%)p99.99( 99.81%)d 1.73(  0.12%)
                 2.301%  C 18 s(  0.08%)p99.99( 99.77%)d 2.03(  0.15%)
                 3.282%  C 19 s(  0.03%)p99.99( 99.50%)d13.60(  0.46%)
                34.404%  C 20 s(  0.01%)p 1.00( 99.95%)d 0.00(  0.05%)
```

Zr-Me interaction:

```
 57. (2.00000)  97.8266% BD ( 1)Zr  6- C  7
                20.148% Zr  6 s( 12.70%)p 0.01(  0.07%)d 6.87( 87.22%)
                77.689%  C  7 s( 25.70%)p 2.89( 74.28%)d 0.00(  0.02%)
```

Resonance structure **3**:
C-H interaction:

```
  82. (2.00000)  97.3849% BD ( 1) C 15- H 26
                 1.463% Zr  6 s( 22.99%)p 0.02(  0.56%)d 3.33( 76.46%)
                60.154%  C 15 s( 25.16%)p 2.97( 74.73%)d 0.00(  0.11%)
                37.262%  H 26 s( 99.95%)p 0.00(  0.05%)
```

Zr-Me interaction:
57. (2.00000) 97.8333% BD (1)Zr 6- C 7
20.109% Zr 6 s(12.65%)p 0.01(0.08%)d 6.90(87.28%)
77.733% C 7 s(25.66%)p 2.90(74.32%)d 0.00(0.02%)

Resonance structure **4**:
C-H interaction:
83. (2.00000) 97.3534% BD (1) C 15- H 26
1.407% Zr 6 s(22.46%)p 0.03(0.58%)d 3.43(76.96%)
60.085% C 15 s(25.01%)p 2.99(74.88%)d 0.00(0.11%)
37.319% H 26 s(99.95%)p 0.00(0.05%)

C-C Interaction:
82. (2.00000) 85.0133% BD (2) C 15- C 20
2.754% Zr 6 s(31.00%)p 0.03(0.96%)d 2.20(68.04%)
50.073% C 15 s(0.70%)p99.99(99.28%)d 0.03(0.02%)
4.945% C 16 s(0.10%)p99.99(99.56%)d 3.25(0.34%)
1.216% C 17 s(0.18%)p99.99(99.52%)d 1.63(0.30%)
1.132% C 18 s(0.06%)p99.99(99.62%)d 5.12(0.31%)
4.382% C 19 s(0.01%)p 1.00(99.64%)d 0.00(0.35%)
34.975% C 20 s(0.01%)p99.99(99.94%)d 3.40(0.05%)

Zr-Me interaction:
57. (2.00000) 97.8332% BD (1)Zr 6- C 7
20.110% Zr 6 s(12.64%)p 0.01(0.08%)d 6.90(87.28%)
77.732% C 7 s(25.66%)p 2.90(74.32%)d 0.00(0.02%)

Resonance structure **5**:
Zr-C interaction:
82. (2.00000) 97.3834% BD (1) C 15- H 26
1.468% Zr 6 s(23.08%)p 0.02(0.56%)d 3.31(76.37%)
60.161% C 15 s(25.17%)p 2.97(74.73%)d 0.00(0.11%)
37.255% H 26 s(99.95%)p 0.00(0.05%)

Zr-Me interaction:
57. (2.00000) 97.8328% BD (1)Zr 6- C 7
20.109% Zr 6 s(12.64%)p 0.01(0.08%)d 6.90(87.28%)
77.733% C 7 s(25.66%)p 2.90(74.32%)d 0.00(0.02%)

Resonance structure **6**:
NLMO algorithm failed to converge

Resonance structure **7**:
C-H interaction:
83. (2.00000) 97.3518% BD (1) C 15- H 26
1.412% Zr 6 s(22.43%)p 0.03(0.58%)d 3.43(77.00%)
60.089% C 15 s(25.01%)p 2.99(74.88%)d 0.00(0.11%)
37.314% H 26 s(99.95%)p 0.00(0.05%)

C-C interaction:
82. (2.00000) 85.0773% BD (2) C 15- C 20
2.721% Zr 6 s(31.03%)p 0.03(0.96%)d 2.19(68.01%)
49.339% C 15 s(0.70%)p99.99(99.28%)d 0.03(0.02%)
4.359% C 16 s(0.11%)p99.99(99.51%)d 3.49(0.38%)
1.451% C 17 s(0.16%)p99.99(99.61%)d 1.40(0.22%)
1.147% C 18 s(0.06%)p99.99(99.60%)d 5.58(0.34%)
4.713% C 19 s(0.01%)p 1.00(99.66%)d 0.00(0.34%)
35.759% C 20 s(0.01%)p99.99(99.94%)d 3.27(0.04%)

Zr-Me interaction:
57. (2.00000) 97.8328% BD (1)Zr 6- C 7
20.109% Zr 6 s(12.64%)p 0.01(0.08%)d 6.90(87.28%)
77.733% C 7 s(25.66%)p 2.90(74.32%)d 0.00(0.02%)

Resonance structure **8**:
C-H interaction:
82. (2.00000) 97.3555% BD (1) C 15- H 26
1.398% Zr 6 s(22.40%)p 0.03(0.59%)d 3.44(77.00%)
60.069% C 15 s(25.08%)p 2.98(74.81%)d 0.00(0.11%)
37.338% H 26 s(99.95%)p 0.00(0.05%)

C-C interaction:
81. (2.00000) 84.9801% BD (2) C 15- C 20
2.671% Zr 6 s(29.73%)p 0.03(1.00%)d 2.33(69.27%)
49.522% C 15 s(0.68%)p99.99(99.30%)d 0.03(0.02%)
4.500% C 16 s(0.11%)p99.99(99.52%)d 3.47(0.37%)
1.458% C 17 s(0.17%)p99.99(99.60%)d 1.33(0.23%)
1.196% C 18 s(0.07%)p99.99(99.62%)d 4.83(0.32%)
4.596% C 19 s(0.01%)p 1.00(99.65%)d 0.00(0.34%)
35.480% C 20 s(0.01%)p99.99(99.94%)d 3.30(0.04%)

Zr-Me interaction:
57. (2.00000) 97.8327% BD (1)Zr 6- C 7
20.125% Zr 6 s(12.70%)p 0.01(0.07%)d 6.87(87.22%)
77.718% C 7 s(25.69%)p 2.89(74.29%)d 0.00(0.02%)

Non-Covalent Interactions (NCI)

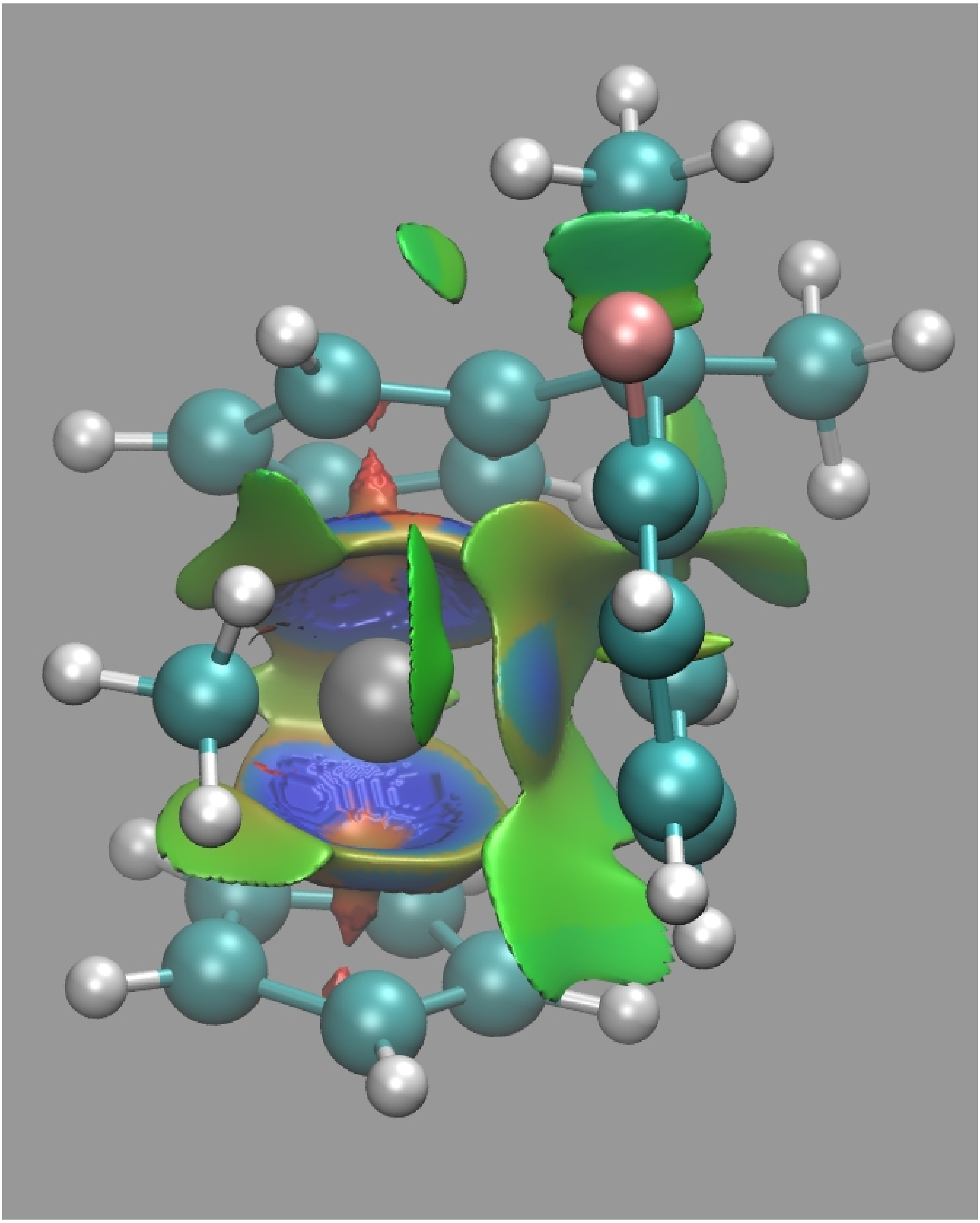

2A-B3LYP

Bader:

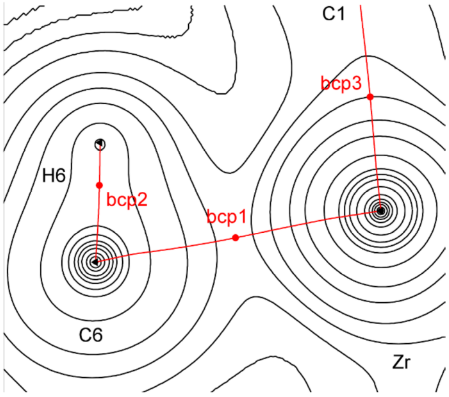

Electrondensity

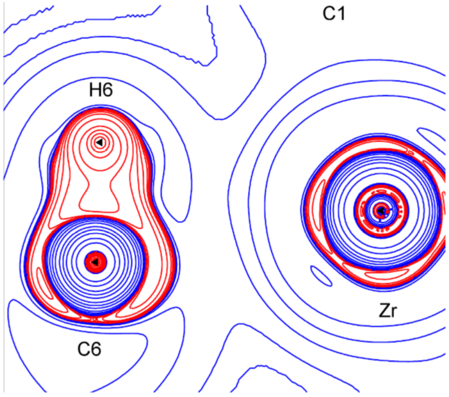

Laplacian

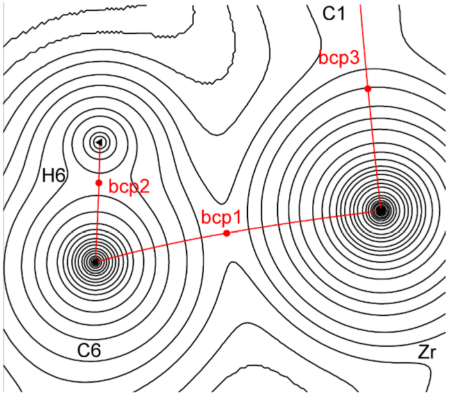

Virial

	$\varrho(\mathbf{r})$	$\nabla^2\varrho(\mathbf{r})$
bcp1	0.03230	-0.02060
bcp2	0.27378	0.22626
bcp3	0.09545	-0.01173

NBO:

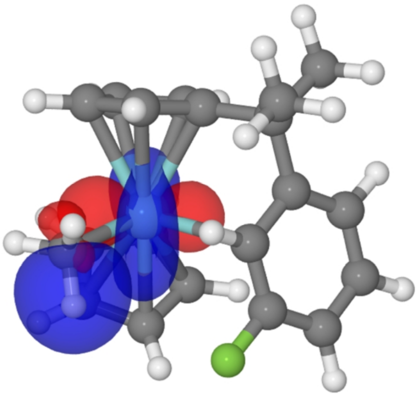

1

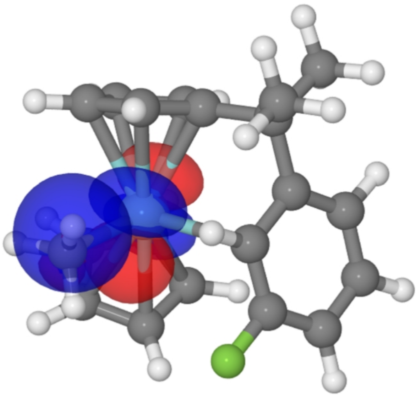

2

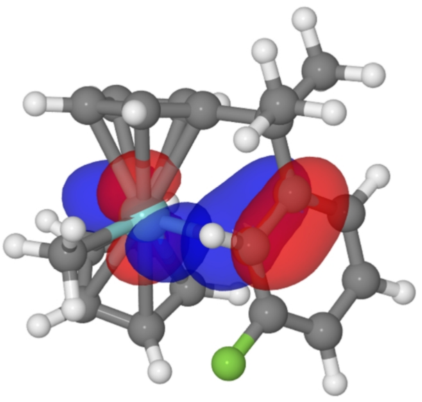

3

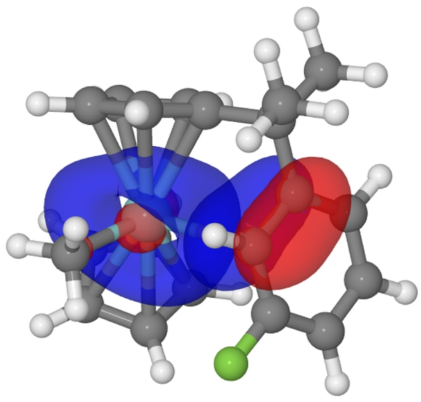

4

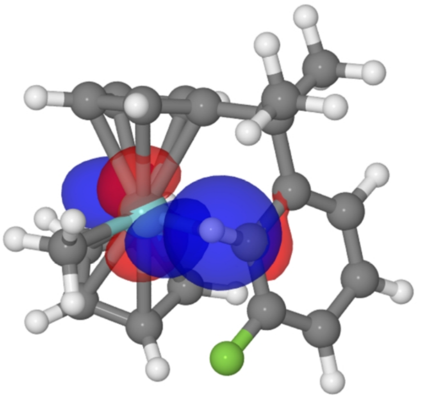

5

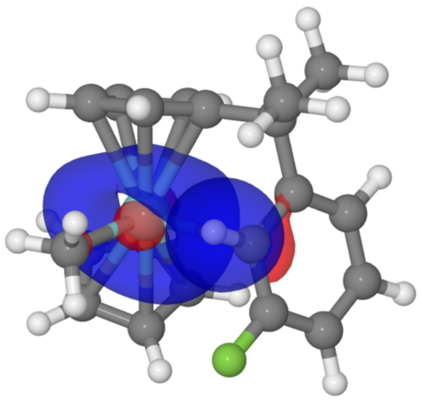

6

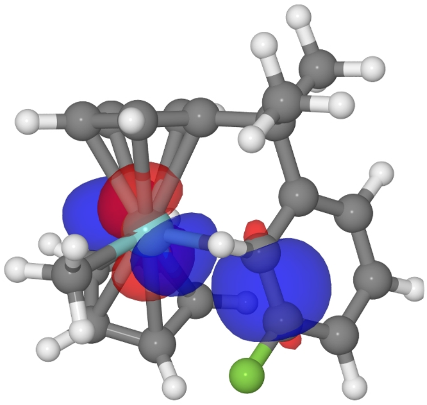

7

	Orbitals	E(2P)
1	σ_{CH} = 0.778$(sp^{2.95})_{C7}$ – 0.628$(s)_{H43}$ → LV_{Zr} = $sd^{13.05}$	2.41
2	σ_{CH} = 0.776$(sp^{2.95})_{C7}$ – 0.631$(s)_{H44}$ → LV_{Zr} = $p^{1.35}d^{99.99}$	2.15
3	π_{CC} = 0.780$(sp^{99.99})_{C8}$ – 0.626$(sp^{99.99}d^{0.89})_{C9}$ → LV_{Zr} = $sd^{43.66}$	7.99
4	π_{CC} = 0.780$(sp^{99.99})_{C8}$ – 0.626$(sp^{99.99}d^{0.89})_{C9}$ → LV_{Zr} = $sd^{0.32}$	8.61
5	σ_{CH} = 0.793$(sp^{2.56})_{C8}$ – 0.609$(s)_{H24}$ → LV_{Zr} = $sd^{43.66}$	5.52
6	σ_{CH} = 0.793$(sp^{2.56})_{C8}$ – 0.609$(s)_{H24}$ → LV_{Zr} = $sd^{0.32}$	2.97
7	σ_{CC} = 0.716$(sp^{1.92})_{C8}$ – 0.698$(sp^{1.58})_{C13}$ → LV_{Zr} = $sd^{43.66}$	2.68

Natural Resonance Theory:

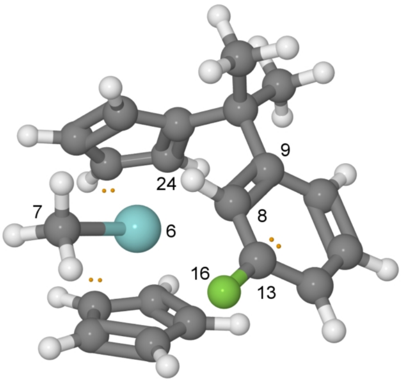

1

Wgt=11.04%;
rhoNL=5.97607;
D(0)=0.10463

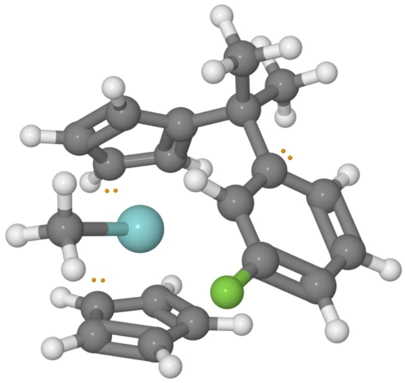

2

Wgt=10.26%;
rhoNL=6.02666;
D(0)=0.10507

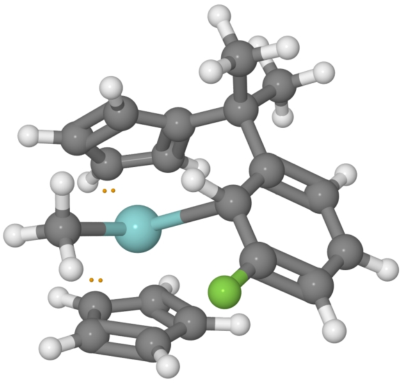

3

Wgt=9.88%;
rhoNL=5.64835;
D(0)=0.10172

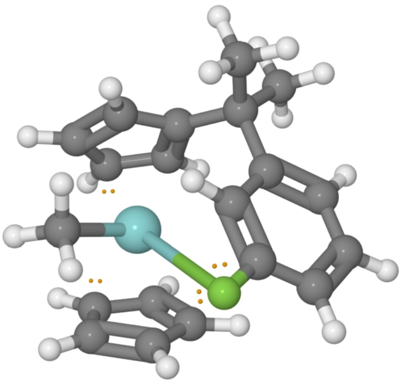

4

Wgt=8.82%;
rhoNL=5.28198;
D(0)=0.09837

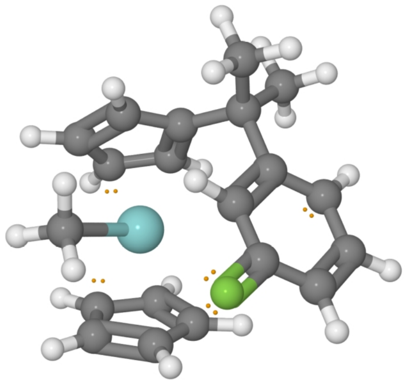

5

Wgt=8.52%;
rhoNL=5.82961;
D(0)=0.10334

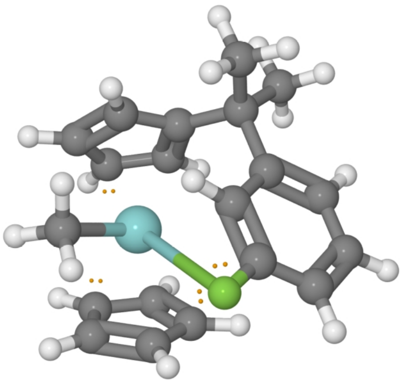

6

Wgt=6.47%;
rhoNL=5.23948;
D(0)=0.09797

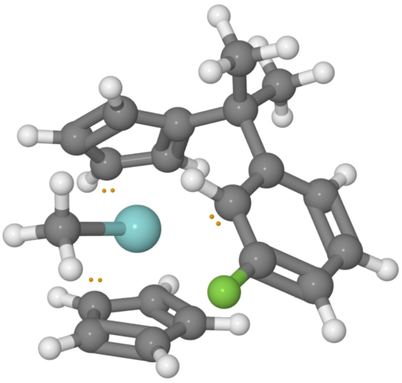

7

Wgt=5.22%;
rhoNL=5.76928;
D(0)=0.10280

Natural Localised Molecular Orbitals (NLMO):

Only contributions over 1% are reported.

NLMO / Occupancy / Percent from Parent NBO / Atomic Hybrid Contributions

Resonance structure **1**:
NLMO algorithm failed to converge

Resonance structure **2**:
NLMO algorithm failed to converge

Resonance structure **3**:
Zr-C interaction:

```
  58. (2.00000)  61.1103% BD ( 1)Zr  6- C  8
                   4.098% Zr  6 s( 24.91%)p 0.03(  0.72%)d 2.98( 74.36%)
                  57.159%  C  8 s(  0.81%)p99.99( 99.17%)d 0.02(  0.02%)
                  12.945%  C  9 s(  0.09%)p99.99( 99.75%)d 1.88(  0.16%)
                   1.266%  C 10 s(  0.12%)p99.99( 99.20%)d 5.85(  0.68%)
                   9.446%  C 11 s(  0.06%)p99.99( 99.94%)d 0.04(  0.00%)
                   1.261%  C 12 s(  0.22%)p99.99( 99.12%)d 3.09(  0.67%)
                  13.144%  C 13 s(  0.02%)p99.99( 99.79%)d12.99(  0.20%)
```

C-H interaction:

```
 64. (2.00000)  97.5789% BD ( 1) C  8- H 24
                   1.110% Zr  6 s( 26.97%)p 0.04(  0.95%)d 2.67( 72.08%)
                  61.533%  C  8 s( 26.04%)p 2.84( 73.86%)d 0.00(  0.10%)
                  36.089%  H 24 s( 99.96%)p 0.00(  0.04%)
```

Zr-Me interaction:

```
  57. (2.00000)  97.7230% BD ( 1)Zr  6- C  7
                  19.940% Zr  6 s( 13.11%)p 0.01(  0.08%)d 6.62( 86.80%)
                  77.793%  C  7 s( 25.83%)p 2.87( 74.15%)d 0.00(  0.02%)
```

Resonance structure **4**:
C-H interaction:

```
  64. (2.00000)  97.5487% BD ( 1) C  8- H 24
                   1.058% Zr  6 s( 26.54%)p 0.04(  0.99%)d 2.73( 72.47%)
                  61.472%  C  8 s( 25.98%)p 2.85( 73.92%)d 0.00(  0.10%)
                  36.140%  H 24 s( 99.96%)p 0.00(  0.04%)
```

C-C interaction:

```
  63. (2.00000)  83.9670% BD ( 2) C  8- C 13
                   2.887% Zr  6 s( 25.42%)p 0.03(  0.73%)d 2.90( 73.85%)
                  51.633%  C  8 s(  0.92%)p99.99( 99.05%)d 0.03(  0.03%)
                   5.327%  C  9 s(  0.16%)p99.99( 99.50%)d 2.19(  0.35%)
                   1.739%  C 10 s(  0.06%)p99.99( 99.79%)d 2.70(  0.15%)
                   1.158%  C 11 s(  0.11%)p99.99( 99.60%)d 2.62(  0.29%)
                   4.332%  C 12 s(  0.00%)p 1.00( 99.58%)d 0.00(  0.41%)
                  32.358%  C 13 s(  0.02%)p99.99( 99.93%)d 3.19(  0.06%)
```

Zr-Me interaction:
56. (2.00000) 97.7095% BD (1)Zr 6- C 7
19.940% Zr 6 s(13.07%)p 0.01(0.08%)d 6.65(86.85%)
77.787% C 7 s(25.81%)p 2.87(74.17%)d 0.00(0.02%)

Resonance structure **5**:
C-H interaction:
64. (2.00000) 97.5452% BD (1) C 8- H 24
1.066% Zr 6 s(25.90%)p 0.04(0.99%)d 2.82(73.12%)
61.490% C 8 s(25.75%)p 2.88(74.16%)d 0.00(0.10%)
36.124% H 24 s(99.96%)p 0.00(0.04%)

C-C interaction:
62. (2.00000) 83.3962% BD (2) C 8- C 9
3.367% Zr 6 s(24.46%)p 0.03(0.81%)d 3.06(74.74%)
52.078% C 8 s(0.98%)p99.99(98.99%)d 0.03(0.03%)
31.362% C 9 s(0.08%)p99.99(99.86%)d 0.72(0.06%)
3.457% C 10 s(0.00%)p 1.00(99.58%)d 0.00(0.42%)
1.204% C 11 s(0.12%)p99.99(99.63%)d 2.02(0.25%)
1.236% C 12 s(0.17%)p99.99(99.59%)d 1.38(0.24%)
5.539% C 13 s(0.04%)p99.99(99.47%)d12.20(0.49%)

Zr-Me interaction:
57. (2.00000) 97.7231% BD (1)Zr 6- C 7
19.941% Zr 6 s(13.11%)p 0.01(0.08%)d 6.62(86.81%)
77.791% C 7 s(25.84%)p 2.87(74.15%)d 0.00(0.02%)

Resonance structure **6**:
C-H interaction:
64. (2.00000) 97.5487% BD (1) C 8- H 24
1.058% Zr 6 s(26.54%)p 0.04(0.99%)d 2.73(72.47%)
61.472% C 8 s(25.98%)p 2.85(73.92%)d 0.00(0.10%)
36.140% H 24 s(99.96%)p 0.00(0.04%)

C-C interaction:
63. (2.00000) 83.9670% BD (2) C 8- C 13
2.887% Zr 6 s(25.42%)p 0.03(0.73%)d 2.90(73.85%)
51.633% C 8 s(0.92%)p99.99(99.05%)d 0.03(0.03%)
5.327% C 9 s(0.16%)p99.99(99.50%)d 2.19(0.35%)
1.739% C 10 s(0.06%)p99.99(99.79%)d 2.70(0.15%)
1.158% C 11 s(0.11%)p99.99(99.60%)d 2.62(0.29%)
4.332% C 12 s(0.00%)p 1.00(99.58%)d 0.00(0.41%)
32.358% C 13 s(0.02%)p99.99(99.93%)d 3.19(0.06%)

Zr-Me interaction:
56. (2.00000) 97.7095% BD (1)Zr 6- C 7
19.940% Zr 6 s(13.07%)p 0.01(0.08%)d 6.65(86.85%)
77.787% C 7 s(25.81%)p 2.87(74.17%)d 0.00(0.02%)

Resonance structure **7**:

C-H interaction:

```
 64. (2.00000)  97.5724% BD ( 1) C  8- H 24
                  1.133% Zr  6 s( 26.51%)p 0.04(  0.94%)d 2.74( 72.55%)
                 61.556%  C  8 s( 26.12%)p 2.83( 73.78%)d 0.00(  0.10%)
                 36.058%  H 24 s( 99.96%)p 0.00(  0.04%)
```

Zr-Me interaction:

```
 58. (2.00000)  97.7233% BD ( 1)Zr  6- C  7
                 19.940% Zr  6 s( 13.12%)p 0.01(  0.08%)d 6.62( 86.80%)
                 77.792%  C  7 s( 25.84%)p 2.87( 74.15%)d 0.00(  0.02%)
```

Non-Covalent Interactions (NCI)

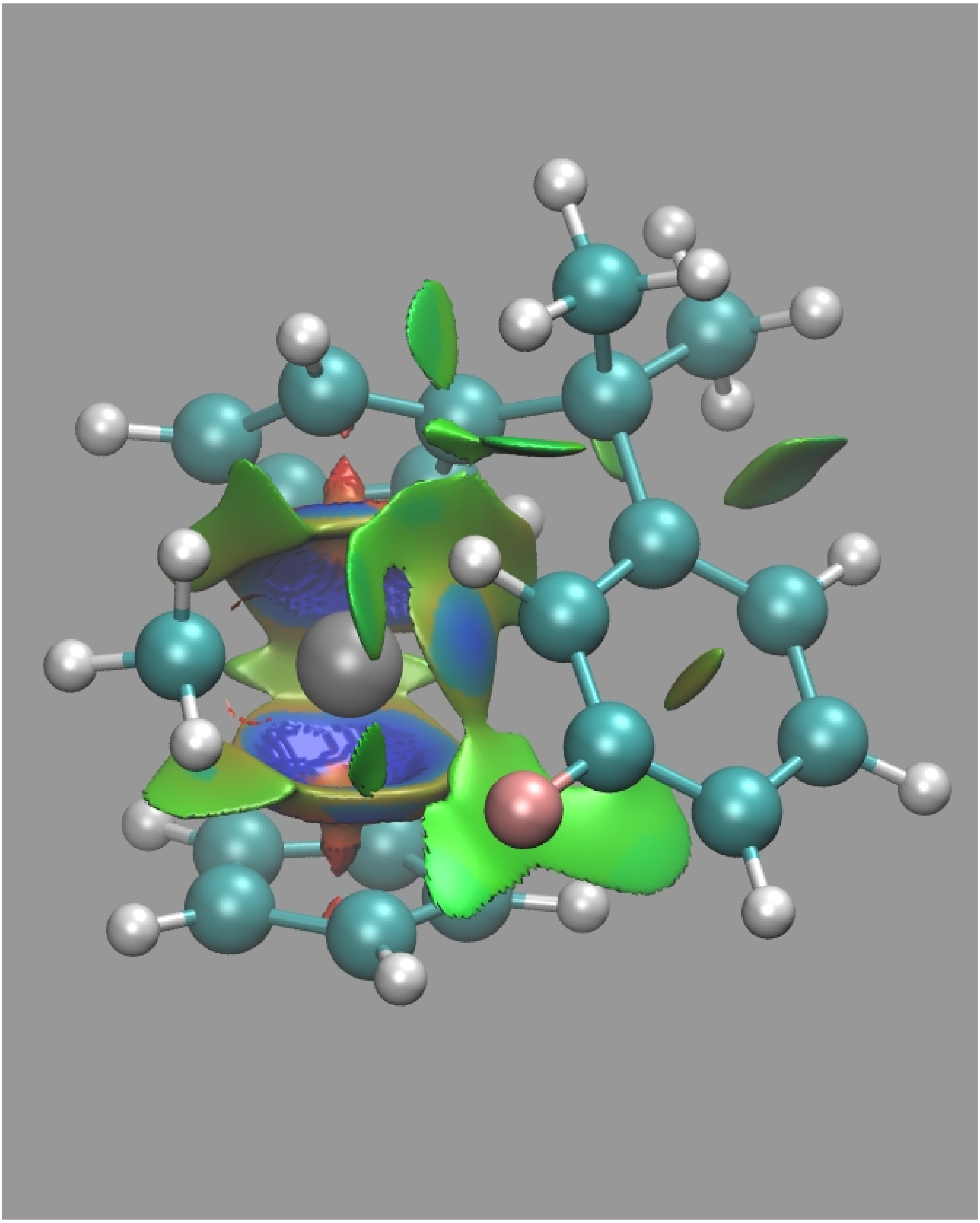

2B-B3LYP

Bader:

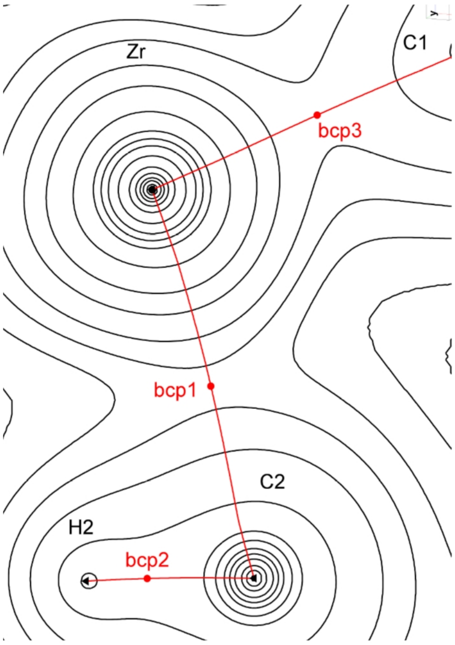

Electrondensity

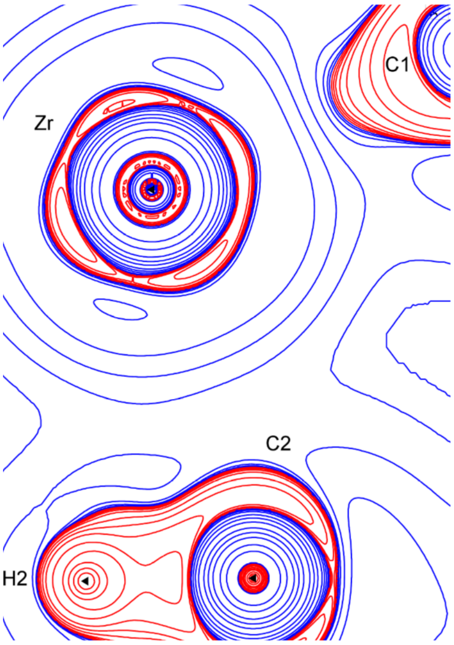

Laplacian

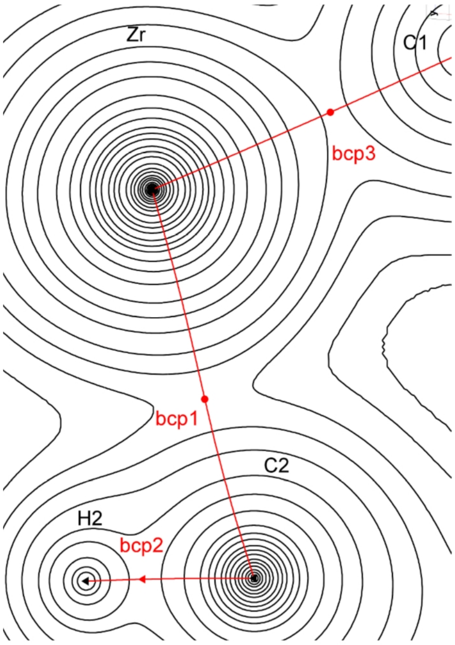

Virial

	ϱ(**r**)	∇^2ϱ(**r**)
bcp1	0.03428	-0.02106
bcp2	0.27076	0.22060
bcp3	0.09592	-0.01160

NBO:

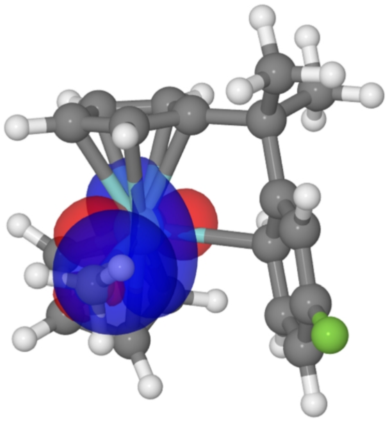

1

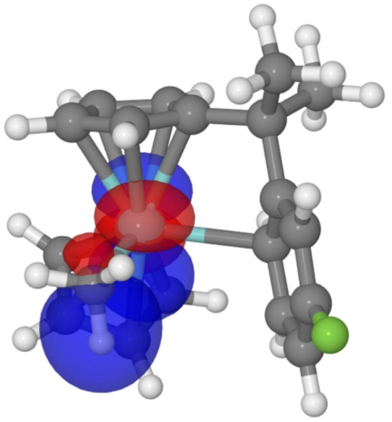

2

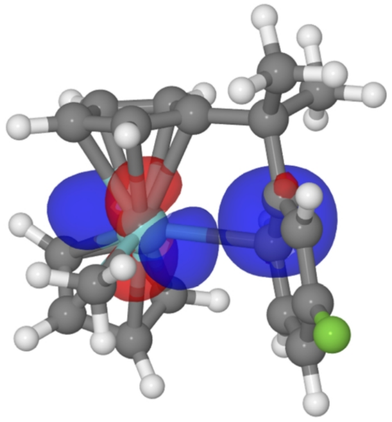

3

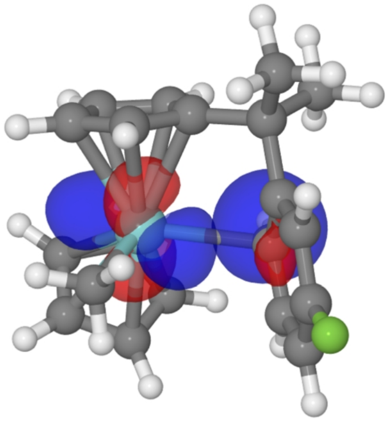

4

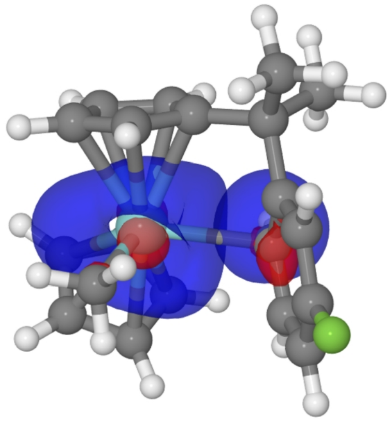

5

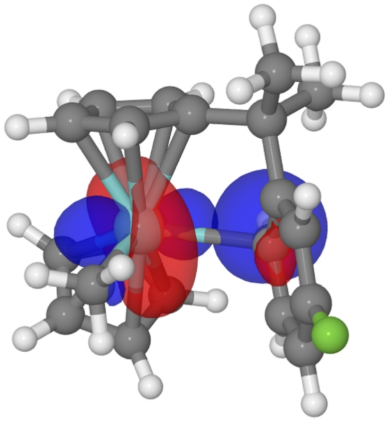

6

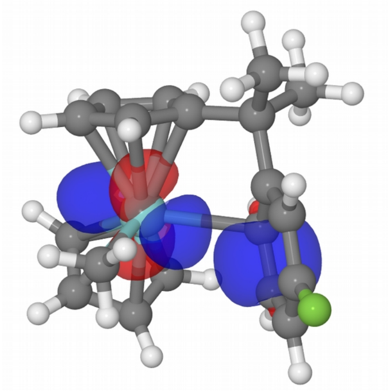

7

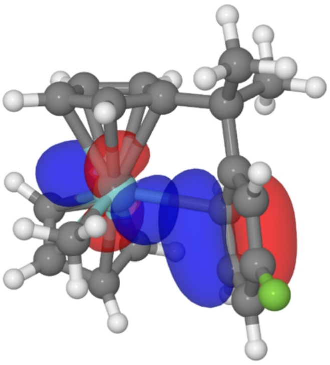

8

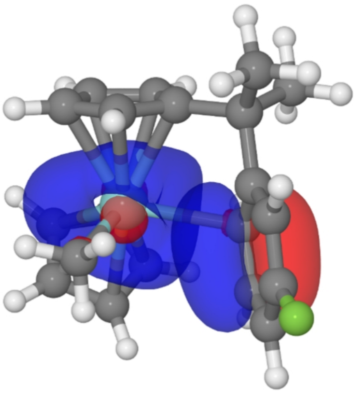

9

	Orbitals	E(2P)
1	σ_{CH} = 0.776$(sp^{2.96})_{C21}$ – 0.631$(s)_{H37}$ → LV_{Zr} = $sd^{10.90}$	2.60
2	σ_{CH} = 0.777$(sp^{2.93})_{C21}$ – 0.630$(s)_{H39}$ → LV_{Zr} = $p^{0.70}d^{99.99}$	2.39
3	σ_{CC} = 0.710$(sp^{1.74})_{C2}$ – 0.704$(sp^{2.02})_{C3}$ → LV_{Zr} = $sd^{43.39}$	2.59
4	σ_{CH} = 0.786$(sp^{2.66})_{C2}$ – 0.618$(s)_{H26}$ → LV_{Zr} = $sd^{43.39}$	2.91
5	σ_{CH} = 0.786$(sp^{2.66})_{C2}$ – 0.618$(s)_{H26}$ → LV_{Zr} = $sd^{0.35}$	5.52
6	σ_{CH} = 0.786$(sp^{2.66})_{C2}$ – 0.618$(s)_{H26}$ → LV_{Zr} = $sd^{10.90}$	3.10
7	σ_{CC} = 0.700$(sp^{1.76})_{C1}$ – 0.714$(sp^{1.81})_{C2}$ → LV_{Zr} = $sd^{43.39}$	3.03
8	π_{CC} = 0.626$(p^{99.99}d^{2.69})_{C1}$ – 0.780$(sp^{99.99})_{C2}$ → LV_{Zr} = $sd^{43.39}$	9.02
9	π_{CC} = 0.626$(p^{99.99}d^{2.69})_{C1}$ – 0.780$(sp^{99.99})_{C2}$ → LV_{Zr} = $sd^{0.35}$	7.39

Natural Resonance Theory:

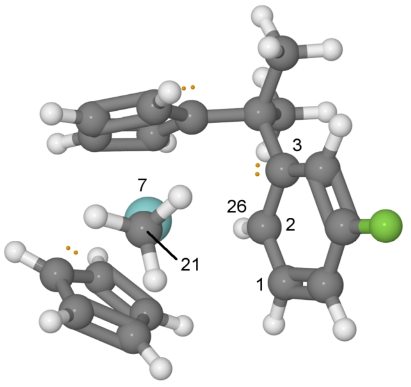<br>**1**	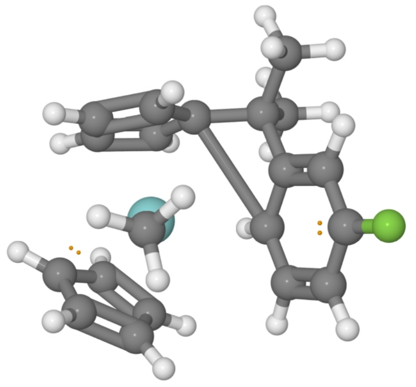<br>**2**	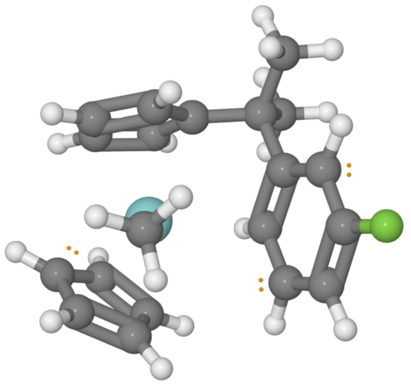<br>**3**
Wgt=13.37%;<br>rhoNL=6.02051;<br>D(0)=0.10502	Wgt=11.08%;<br>rhoNL=5.84664;<br>D(0)=0.10349	Wgt=9.74%;<br>rhoNL=6.07860;<br>D(0)=0.10552
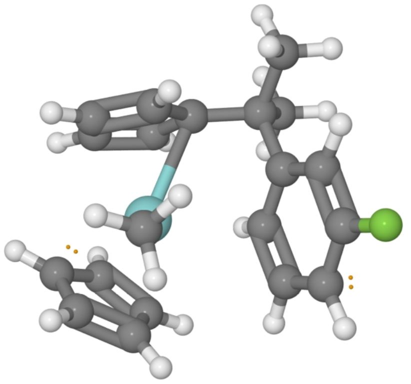<br>**4**	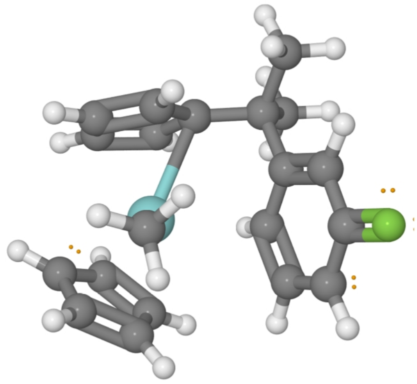<br>**5**	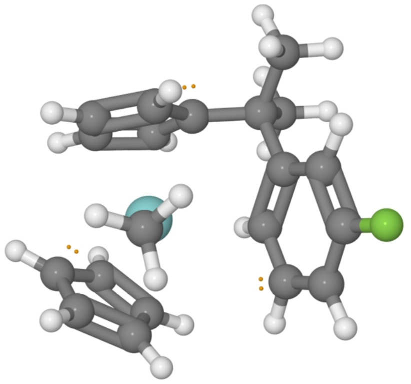<br>**6**
Wgt=6.86%;<br>rhoNL=5.73091;<br>D(0)=0.10246	Wgt=5.19%;<br>rhoNL=5.57042;<br>D(0)=0.10102	Wgt=5.18%;<br>rhoNL=6.01107;<br>D(0)=0.10493

Natural Localised Molecular Orbitals (NLMO):

Only contributions over 1% are reported.

NLMO / Occupancy / Percent from Parent NBO / Atomic Hybrid Contributions

Resonance structure **1**:
NLMO algorithm failed to converge

Resonance structure **2**:
C-H interaction:

```
  51. (2.00000)  97.3020% BD ( 1) C  2- H 26
                    60.312%  C  2 s( 25.43%)p 2.93( 74.46%)d 0.00(  0.11%)
                     1.551% Zr  7 s( 21.58%)p 0.02(  0.54%)d 3.61( 77.88%)
                    37.027%  H 26 s( 99.95%)p 0.00(  0.05%)
```

Zr-Me interaction:

```
 60. (2.00000)  97.7487% BD ( 1)Zr  7- C 21
                    20.186% Zr  7 s( 13.08%)p 0.01(  0.08%)d 6.64( 86.85%)
                    77.584%  C 21 s( 25.73%)p 2.89( 74.25%)d 0.00(  0.02%)
```

Resonance structure **3**:
C-H interaction:

```
  51. (2.00000)  97.2741% BD ( 1) C  2- H 26
                    60.229%  C  2 s( 25.29%)p 2.95( 74.60%)d 0.00(  0.11%)
                     1.482% Zr  7 s( 21.31%)p 0.03(  0.56%)d 3.67( 78.12%)
                    37.103%  H 26 s( 99.95%)p 0.00(  0.05%)
```

C-C interaction:

```
 50. (2.00000)  83.1314% BD ( 2) C  2- C  3
                     6.022%  C  1 s(  0.01%)p99.99( 99.70%)d25.29(  0.29%)
                    51.878%  C  2 s(  0.94%)p99.99( 99.04%)d 0.02(  0.02%)
                    31.280%  C  3 s(  0.12%)p99.99( 99.82%)d 0.43(  0.05%)
                     3.747%  C  4 s(  0.00%)p 1.00( 99.61%)d 0.00(  0.39%)
                     1.033%  C  5 s(  0.10%)p99.99( 99.55%)d 3.56(  0.35%)
                     1.491%  C  6 s(  0.10%)p99.99( 99.68%)d 2.20(  0.22%)
                     2.877% Zr  7 s( 29.17%)p 0.03(  1.02%)d 2.39( 69.81%)
```

Zr-Me interaction:

```
  60. (2.00000)  97.7522% BD ( 1)Zr  7- C 21
                    20.164% Zr  7 s( 13.20%)p 0.01(  0.08%)d 6.57( 86.72%)
                    77.607%  C 21 s( 25.73%)p 2.89( 74.25%)d 0.00(  0.02%)
```

Resonance structure **4**:
C-H interaction:

```
  50. (2.00000)  97.2819% BD ( 1) C  2- H 26
                    60.198%  C  2 s( 25.24%)p 2.96( 74.66%)d 0.00(  0.11%)
                     1.462% Zr  7 s( 22.21%)p 0.03(  0.58%)d 3.48( 77.22%)
                    37.142%  H 26 s( 99.95%)p 0.00(  0.05%)
```

C-C interaction:

```
 46. (2.00000)  84.5646% BD ( 2) C  1- C  2
                 32.310%  C  1 s(  0.01%)p99.99( 99.94%)d 4.03(  0.05%)
                 52.288%  C  2 s(  0.99%)p99.99( 99.00%)d 0.02(  0.02%)
                  5.642%  C  3 s(  0.14%)p99.99( 99.55%)d 2.34(  0.32%)
                  1.386%  C  4 s(  0.12%)p99.99( 99.64%)d 1.96(  0.24%)
                  1.056%  C  5 s(  0.14%)p99.99( 99.53%)d 2.47(  0.34%)
                  3.670%  C  6 s(  0.01%)p99.99( 99.61%)d32.74(  0.38%)
                  3.060% Zr  7 s( 29.65%)p 0.03(  0.90%)d 2.34( 69.46%)
```

Zr-Me interaction:

```
  60. (2.00000)  97.8498% BD ( 1)Zr  7- C 21
                 20.104% Zr  7 s( 13.09%)p 0.01(  0.08%)d 6.64( 86.84%)
                 77.755%  C 21 s( 25.72%)p 2.89( 74.27%)d 0.00(  0.02%)
```

Resonance structure **5**:

C-H interaction:

```
  49. (2.00000)  97.2809% BD ( 1) C  2- H 26
                 60.199%  C  2 s( 25.25%)p 2.96( 74.64%)d 0.00(  0.11%)
                  1.463% Zr  7 s( 22.16%)p 0.03(  0.58%)d 3.49( 77.26%)
                 37.141%  H 26 s( 99.95%)p 0.00(  0.05%)
```

C-C interaction:

```
  45. (2.00000)  84.6904% BD ( 2) C  1- C  2
                 32.764%  C  1 s(  0.01%)p99.99( 99.94%)d 3.91(  0.05%)
                 51.951%  C  2 s(  0.98%)p99.99( 99.01%)d 0.02(  0.02%)
                  5.022%  C  3 s(  0.18%)p99.99( 99.46%)d 2.05(  0.36%)
                  1.742%  C  4 s(  0.10%)p99.99( 99.73%)d 1.79(  0.17%)
                  1.013%  C  5 s(  0.13%)p99.99( 99.53%)d 2.62(  0.34%)
                  3.775%  C  6 s(  0.01%)p99.99( 99.62%)d29.99(  0.37%)
                  3.062% Zr  7 s( 29.62%)p 0.03(  0.91%)d 2.35( 69.48%)
```

Zr-Me interaction:

```
  60. (2.00000)  97.8498% BD ( 1)Zr  7- C 21
                 20.102% Zr  7 s( 13.09%)p 0.01(  0.08%)d 6.63( 86.83%)
                 77.757%  C 21 s( 25.72%)p 2.89( 74.27%)d 0.00(  0.02%)
```

Resonance structure **6**:

NLMO algorithm failed to converge

Non-Covalent Interactions (NCI)

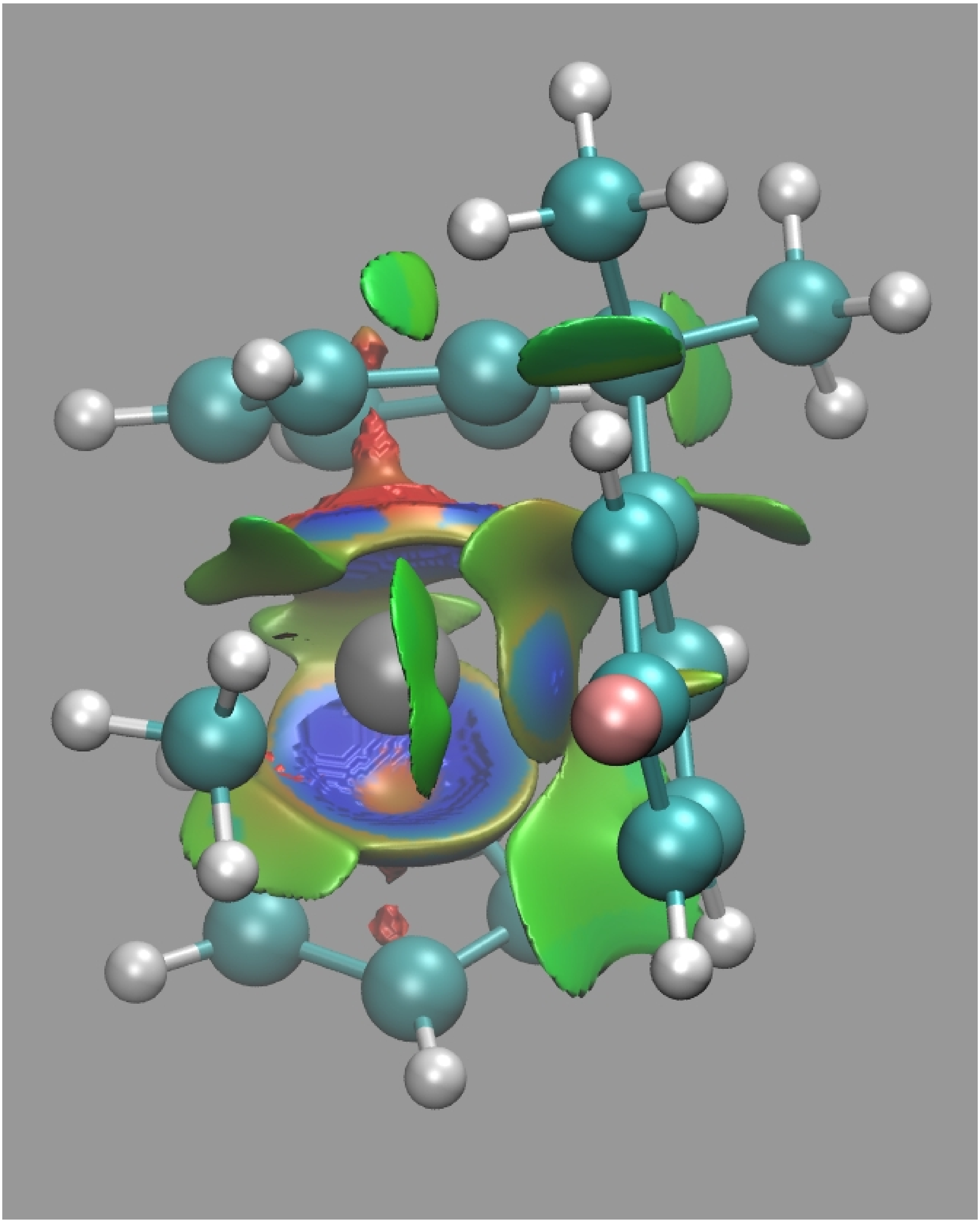

3A-B3LYP

Bader:

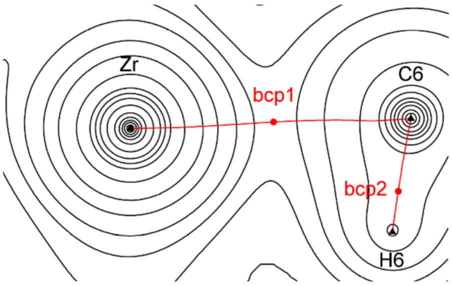

Electrondensity

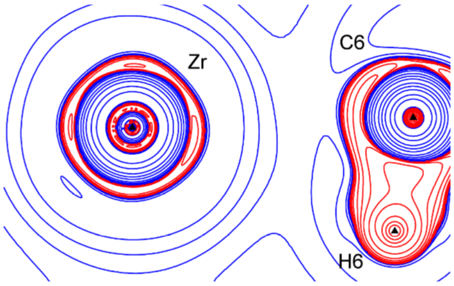

Laplacian

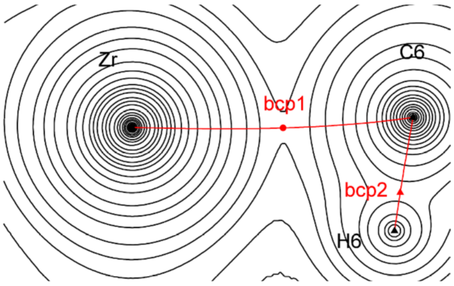

Virial

	$\varrho(\mathbf{r})$	$\nabla^2\varrho(\mathbf{r})$
bcp1	0.02999	-0.01984
bcp2	0.27770	0.23284
bcp3	0.09489	-0.01205

NOB:

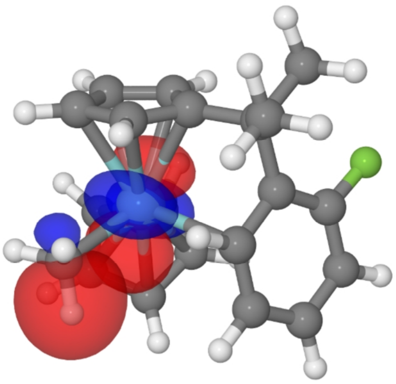

1

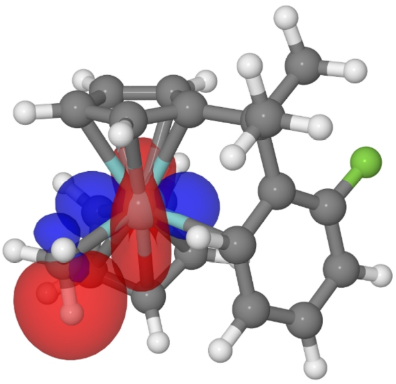

2

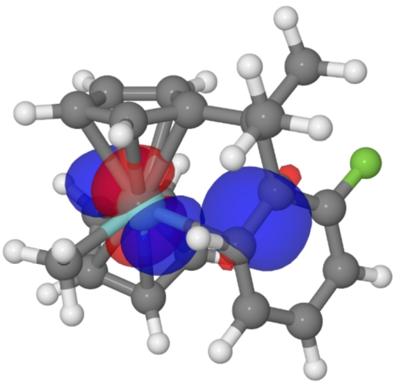

3

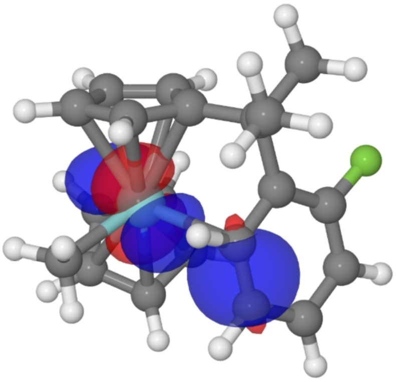

4

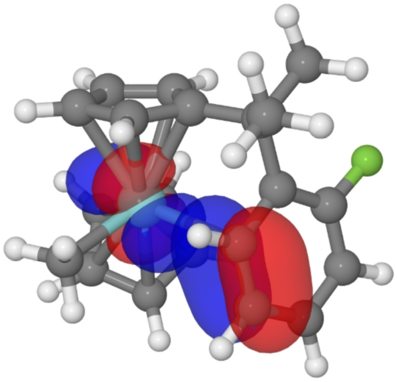

5

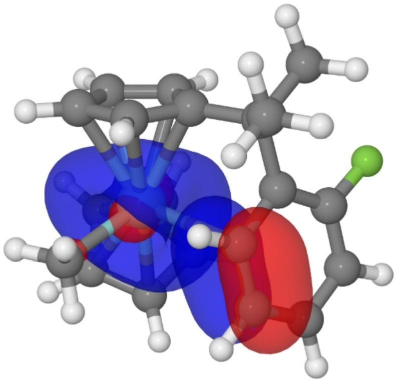

6

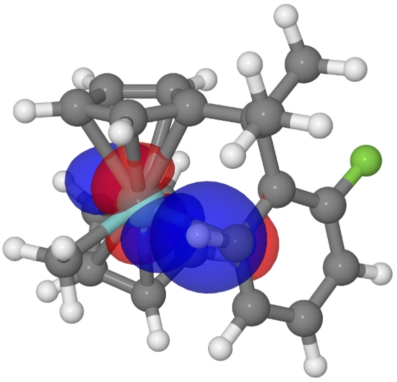

7

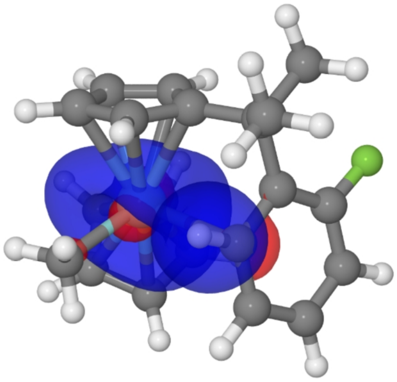

8

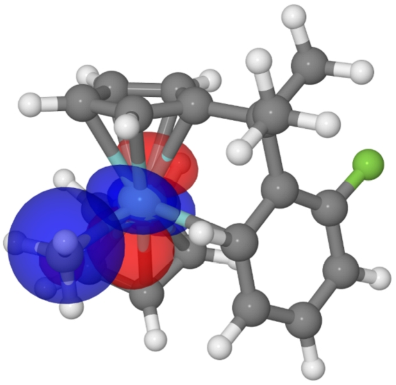

9

	Orbitals	E(2P)
1	σ_{CH} = 0.7761$(sp^{2.97})_{C41}$ – 0.631$(s)_{H43}$ → LV_{Zr} = $p^{2.56}d^{99.9}$	2.06
2	σ_{CH} = 0.7761$(sp^{2.97})_{C41}$ – 0.631$(s)_{H43}$ → LV_{Zr} = $sd^{18.08}$	2.16
3	σ_{CC} = 0.708$(sp^{2.02})_{C4}$ – 0.706$(sp^{1.77})_{C9}$ → LV_{Zr} = $sd^{55.08}$	2.33
4	σ_{CC} = 0.699$(sp^{1.78})_{C8}$ – 0.715$(sp^{1.76})_{C9}$ → LV_{Zr} = $sd^{55.08}$	2.89
5	π_{CC} = 0.659$(p^{99.99}d^{2.51})_{C8}$ – 0.752$(sp^{99.99})_{C9}$ → LV_{Zr} = $sd^{55.08}$	7.26
6	π_{CC} = 0.659$(p^{99.99}d^{2.51})_{C8}$ – 0.752$(sp^{99.99})_{C9}$ LV_{Zr} = $sd^{0.28}$	6.11
7	σ_{CH} = 0.789$(sp^{2.64})_{C9}$ – 0.615$(s)_{H10}$ → LV_{Zr} = $sd^{55.08}$	4.47
8	σ_{CH} = 0.789$(sp^{2.64})_{C9}$ – 0.615$(s)_{H10}$ → LV_{Zr} = $sd^{0.28}$	2.87
9	σ_{CH} = 0.776$(sp^{2.95})_{C41}$ – 0.631$(s)_{H44}$ → LV_{Zr} = $p^{2.56}d^{99.9}$	2.06

Natural Resonance Theory:

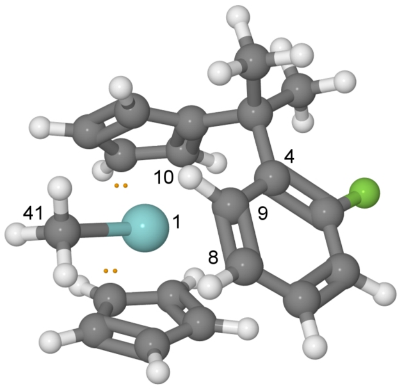

1

Wgt=14.02%;
rhoNL=5.21888;
D(0)=0.09778

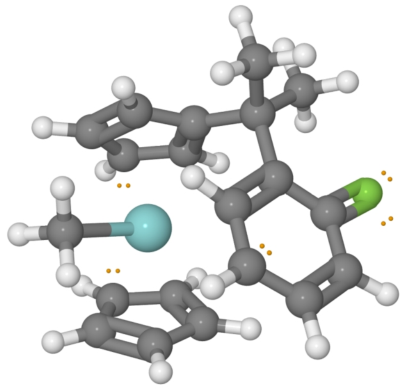

2

Wgt=12.45%;
rhoNL=5.82720;
D(0)=0.10332

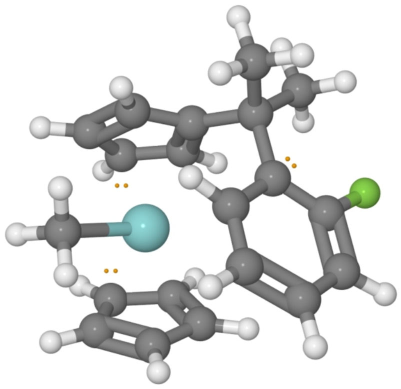

3

Wgt=9.16%;
rhoNL=5.95020;
D(0)=0.10440

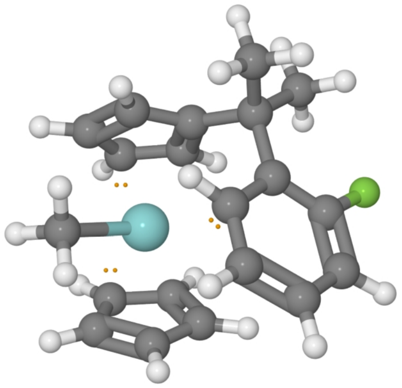

4

Wgt=8.30%;
rhoNL=5.82179;
D(0)=0.10327

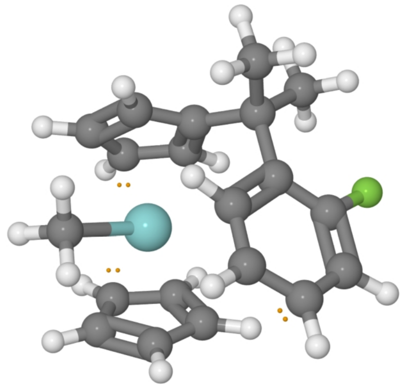

5

Wgt=7.20%;
rhoNL=5.92939;
D(0)=0.10422

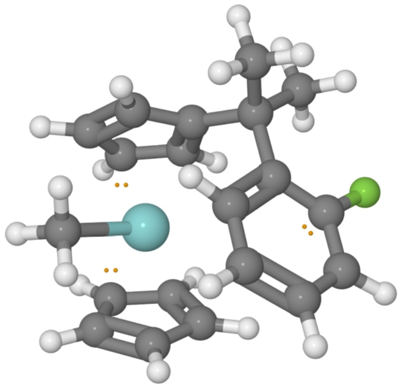

6

Wgt=5.78%;
rhoNL=5.89884;
D(0)=0.10395

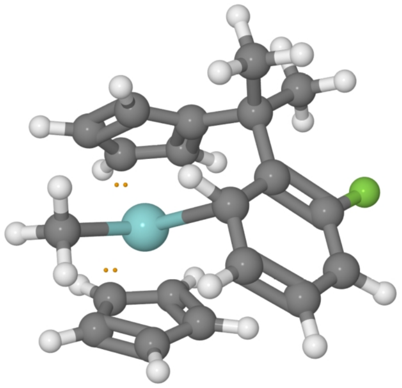

7

Wgt=5.59%;
rhoNL=5.72220;
D(0)=0.10238

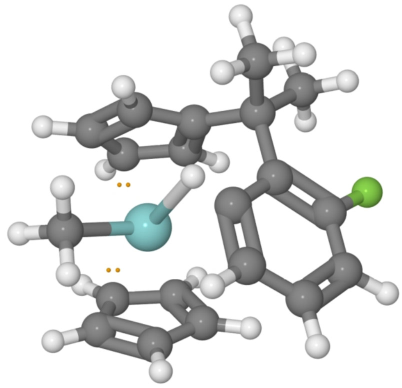

8

Wgt=3.05%;
rhoNL=6.40319;
D(0)=0.10830

Natural Localised Molecular Orbitals (NLMO):

Only contributions over 1% are reported.

NLMO / Occupancy / Percent from Parent NBO / Atomic Hybrid Contributions

Resonance structure **1**:
C-H interaction:

```
  66. (2.00000)  97.7991% BD ( 1) C  9- H 10
                  0.908% Zr  1 s( 24.71%)p 0.04(  0.95%)d 3.01( 74.33%)
                 60.955%  C  9 s( 25.46%)p 2.92( 74.44%)d 0.00(  0.10%)
                 36.891%  H 10 s( 99.95%)p 0.00(  0.05%)
```

C-C interaction:

```
 64. (2.00000)  84.2931% BD ( 2) C  8- C  9
                  2.516% Zr  1 s( 29.02%)p 0.03(  1.00%)d 2.41( 69.98%)
                  4.660%  C  4 s(  0.13%)p99.99( 99.48%)d 3.12(  0.39%)
                  1.619%  C  5 s(  0.08%)p99.99( 99.72%)d 2.62(  0.20%)
                  1.239%  C  6 s(  0.06%)p99.99( 99.61%)d 5.58(  0.33%)
                  5.121%  C  7 s(  0.00%)p 1.00( 99.67%)d 0.00(  0.33%)
                 36.160%  C  8 s(  0.02%)p99.99( 99.94%)d 3.01(  0.05%)
                 48.155%  C  9 s(  0.53%)p99.99( 99.45%)d 0.05(  0.03%)
```

Zr-Me interaction:

```
 45. (2.00000)  97.7589% BD ( 1)Zr  1- C 41
                 19.576% Zr  1 s( 13.12%)p 0.01(  0.08%)d 6.61( 86.80%)
                 78.191%  C 41 s( 25.92%)p 2.86( 74.06%)d 0.00(  0.02%)
```

Resonance structure **2**:
C-H interaction:

```
  66. (2.00000)  97.7953% BD ( 1) C  9- H 10
                  0.906% Zr  1 s( 24.61%)p 0.04(  0.95%)d 3.02( 74.44%)
                 60.951%  C  9 s( 25.45%)p 2.93( 74.45%)d 0.00(  0.10%)
                 36.893%  H 10 s( 99.95%)p 0.00(  0.05%)
```

C-C interaction:

```
  55. (2.00000)  83.4359% BD ( 2) C  4- C  9
                  3.022% Zr  1 s( 25.89%)p 0.04(  0.98%)d 2.82( 73.13%)
                 37.635%  C  4 s(  0.06%)p99.99( 99.88%)d 1.01(  0.06%)
                  5.227%  C  5 s(  0.02%)p99.99( 99.50%)d20.91(  0.48%)
                  1.602%  C  7 s(  0.08%)p99.99( 99.75%)d 2.08(  0.17%)
                  4.358%  C  8 s(  0.01%)p99.99( 99.62%)d26.11(  0.37%)
                 45.820%  C  9 s(  0.54%)p99.99( 99.42%)d 0.07(  0.04%)
```

Zr-Me interaction:

```
  45. (2.00000)  97.7595% BD ( 1)Zr  1- C 41
                 19.574% Zr  1 s( 13.13%)p 0.01(  0.08%)d 6.61( 86.79%)
                 78.195%  C 41 s( 25.92%)p 2.86( 74.06%)d 0.00(  0.02%)
```

Resonance structure **3**:
NLMO algorithm failed to converge

Resonance structure **4**:
C-H interaction:
```
  66. (2.00000)  97.8213% BD ( 1) C  9- H 10
                   0.955% Zr  1 s( 25.20%)p 0.04(  0.90%)d 2.93( 73.90%)
                  61.018%  C  9 s( 25.51%)p 2.92( 74.38%)d 0.00(  0.10%)
                  36.837%  H 10 s( 99.95%)p 0.00(  0.05%)
```

Zr-Me interaction:
```
  46. (2.00000)  97.7593% BD ( 1)Zr  1- C 41
                  19.575% Zr  1 s( 13.13%)p 0.01(  0.08%)d 6.61( 86.80%)
                  78.193%  C 41 s( 25.92%)p 2.86( 74.06%)d 0.00(  0.02%)
```

Resonance structure **5**:
C-H interaction:
```
 66. (2.00000)  97.7991% BD ( 1) C  9- H 10
                   0.900% Zr  1 s( 24.60%)p 0.04(  0.96%)d 3.03( 74.44%)
                  60.947%  C  9 s( 25.44%)p 2.93( 74.45%)d 0.00(  0.10%)
                  36.900%  H 10 s( 99.95%)p 0.00(  0.05%)
```

C-C interaction:
```
  56. (2.00000)  83.0916% BD ( 2) C  4- C  9
                   3.049% Zr  1 s( 26.18%)p 0.04(  1.00%)d 2.78( 72.82%)
                  36.446%  C  4 s(  0.06%)p99.99( 99.88%)d 1.00(  0.06%)
                   5.169%  C  5 s(  0.00%)p 1.00( 99.59%)d 0.00(  0.40%)
                   1.199%  C  6 s(  0.09%)p99.99( 99.62%)d 3.29(  0.29%)
                   1.640%  C  7 s(  0.12%)p99.99( 99.70%)d 1.58(  0.18%)
                   4.857%  C  8 s(  0.01%)p99.99( 99.65%)d24.35(  0.34%)
                  46.666%  C  9 s(  0.55%)p99.99( 99.41%)d 0.07(  0.04%)
```
Zr-Me interaction:
```
  46. (2.00000)  97.7591% BD ( 1)Zr  1- C 41
                  19.575% Zr  1 s( 13.12%)p 0.01(  0.08%)d 6.61( 86.80%)
                  78.193%  C 41 s( 25.92%)p 2.86( 74.06%)d 0.00(  0.02%)
```

Resonance structure **6**:
C-H interaction:
```
 66. (2.00000)  97.7991% BD ( 1) C  9- H 10
                  0.900% Zr  1 s( 24.61%)p 0.04(  0.96%)d 3.03( 74.44%)
                 60.946%  C  9 s( 25.45%)p 2.93( 74.45%)d 0.00(  0.10%)
                 36.900%  H 10 s( 99.95%)p 0.00(  0.05%)
```

C-C interaction:
```
 56. (2.00000)  83.0915% BD ( 2) C  4- C  9
                  3.049% Zr  1 s( 26.19%)p 0.04(  1.00%)d 2.78( 72.81%)
                 36.447%  C  4 s(  0.06%)p99.99( 99.88%)d 1.00(  0.06%)
                  5.169%  C  5 s(  0.00%)p 1.00( 99.59%)d 0.00(  0.40%)
                  1.199%  C  6 s(  0.09%)p99.99( 99.61%)d 3.15(  0.29%)
                  1.639%  C  7 s(  0.12%)p99.99( 99.70%)d 1.59(  0.18%)
                  4.859%  C  8 s(  0.01%)p99.99( 99.65%)d24.66(  0.34%)
                 46.665%  C  9 s(  0.54%)p99.99( 99.42%)d 0.07(  0.04%)
```

Zr-Me interaction:
```
 46. (2.00000)  97.7591% BD ( 1)Zr  1- C 41
                 19.575% Zr  1 s( 13.13%)p 0.01(  0.08%)d 6.61( 86.80%)
                 78.192%  C 41 s( 25.92%)p 2.86( 74.06%)d 0.00(  0.02%)
```

Resonance structure **7**:
C-H interaction:
```
 66. (2.00000)  97.8266% BD ( 1) C  9- H 10
                  0.941% Zr  1 s( 25.35%)p 0.04(  0.91%)d 2.91( 73.74%)
                 61.000%  C  9 s( 25.47%)p 2.92( 74.42%)d 0.00(  0.10%)
                 36.860%  H 10 s( 99.95%)p 0.00(  0.05%)
```

Zr-Me interaction:
```
 46. (2.00000)  97.7592% BD ( 1)Zr  1- C 41
                 19.574% Zr  1 s( 13.12%)p 0.01(  0.08%)d 6.61( 86.80%)
                 78.194%  C 41 s( 25.92%)p 2.86( 74.07%)d 0.00(  0.02%)
```

Resonance structure **8**:
NLMO algorithm failed to converge

Non-Covalent Interactions (NCI)

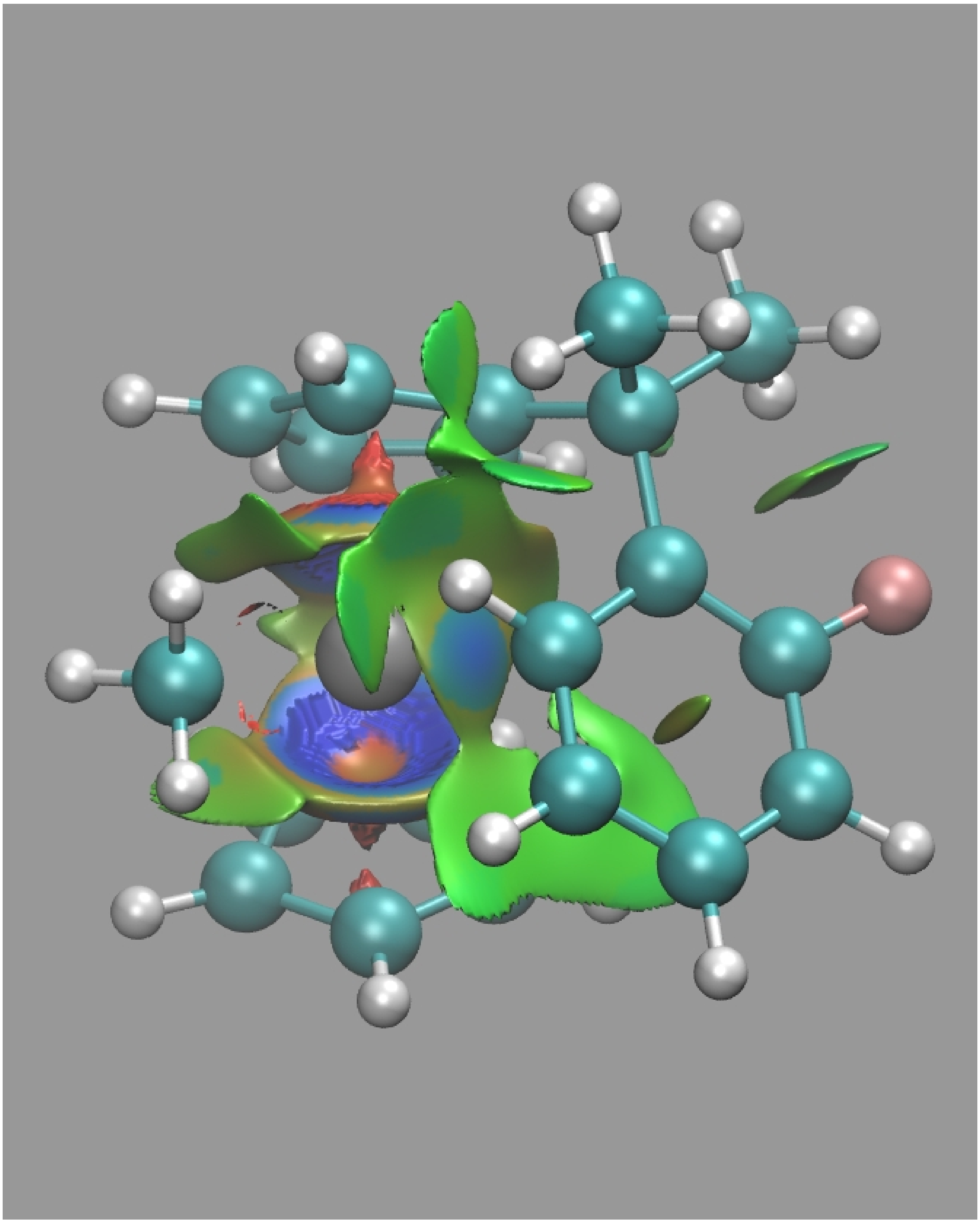

3B-B3LYP

Bader:

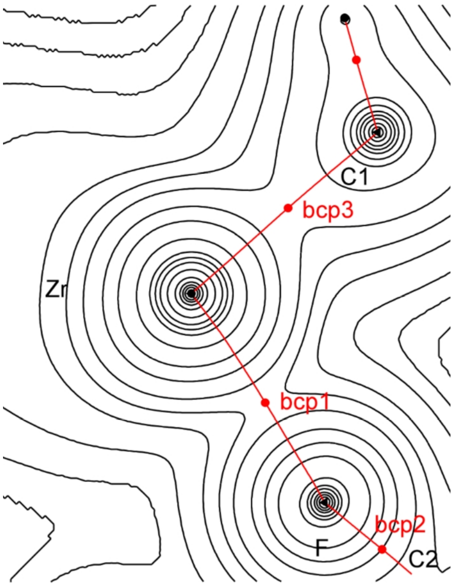

Electrondensity-1

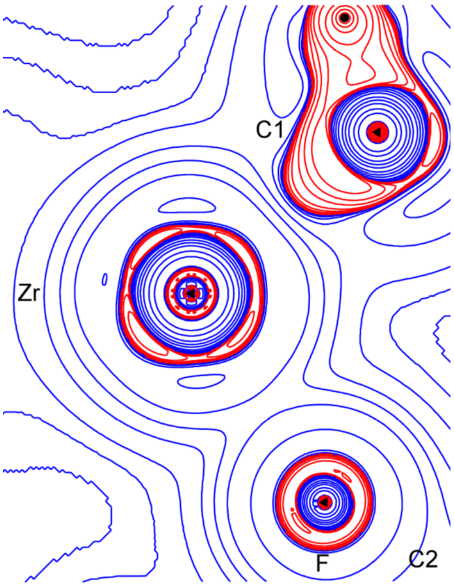

Laplacian-1

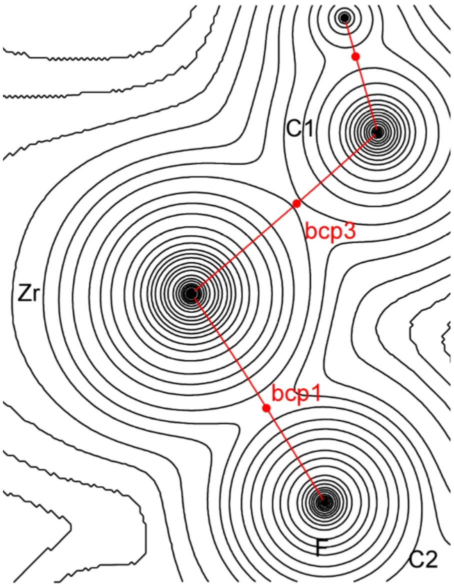

Virial-1

	$\varrho(\mathbf{r})$	$\nabla^2\varrho(\mathbf{r})$
bcp1	0.04527	-0.06424
bcp2	0.18816	-0.03081
bcp3	0.09639	-0.01193

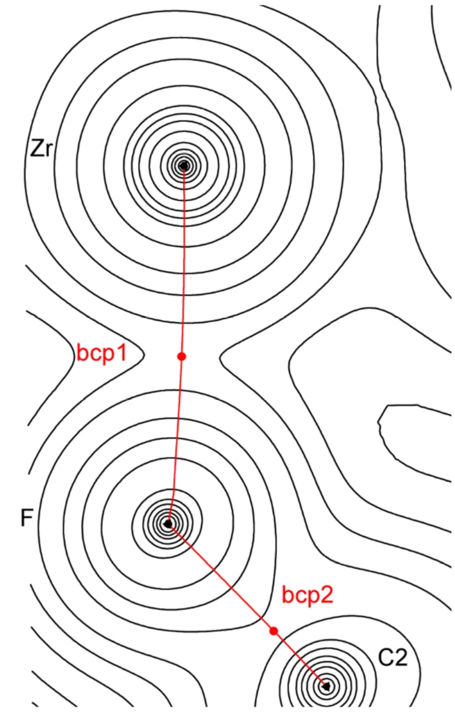

Electrondensity-2

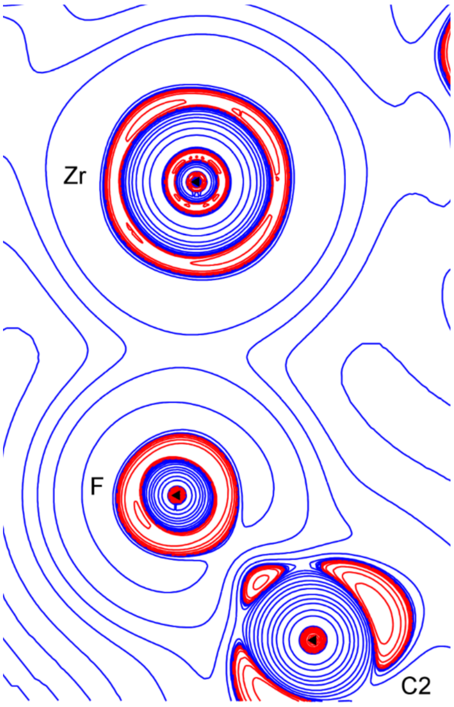

Laplacian-2

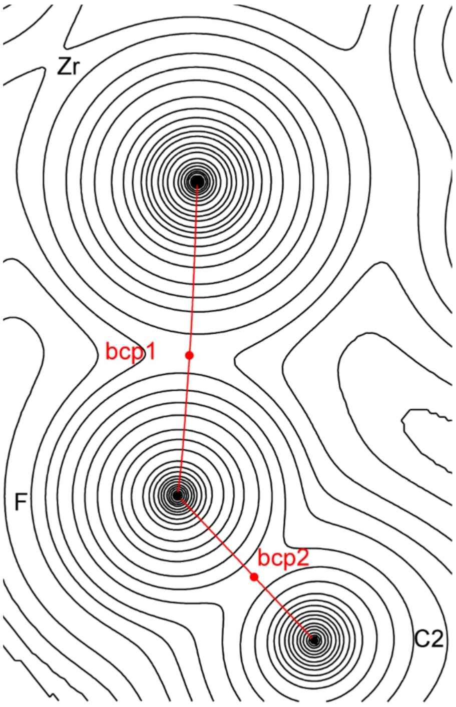

Virial-2

	ϱ(**r**)	∇²ϱ(**r**)
bcp1		
bcp2		
bcp3		

Check!!

NBO:

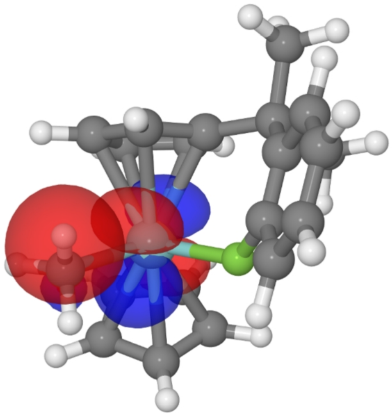

1

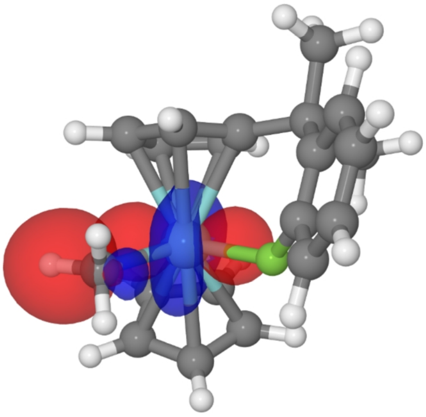

2

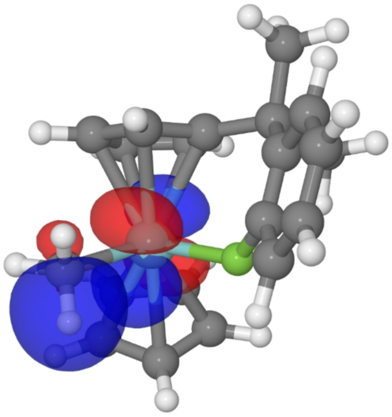

3

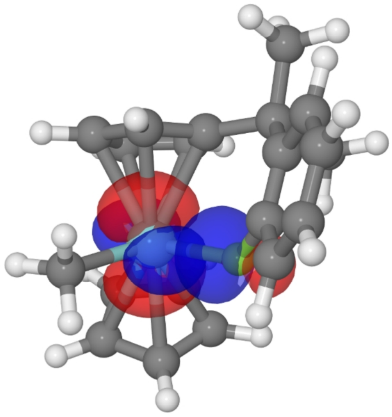

4

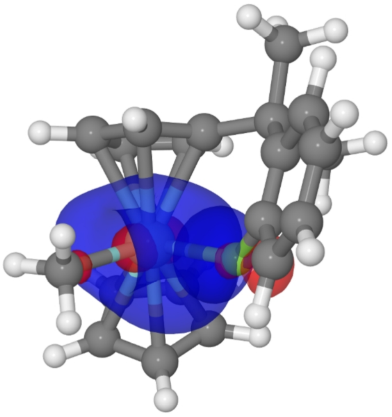

5

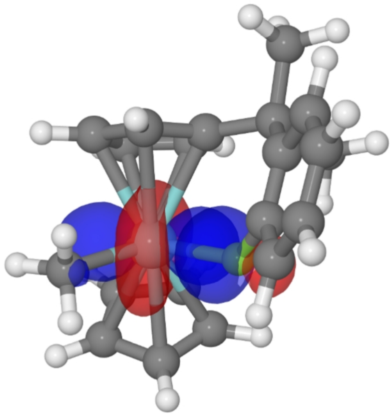

6

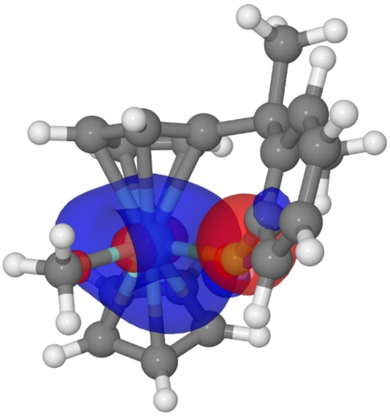

7

	Orbitals	E(2P)
1	σ_{CH} = 0.777($sp^{2.92}$)$_{C11}$ – 0.630(s)$_{H42}$ → LV_{Zr} = $p^1d^{99.99}$	2.19
2	σ_{CH} = 0.782($sp^{3.11}$)$_{C11}$ – 0.623(s)$_{H43}$ → LV_{Zr} = $sp^{0.18}d^{88.72}$	1.94
3	σ_{CH} = 0.777($sp^{2.95}$)$_{C11}$ – 0.630(s)$_{H44}$ → LV_{Zr} = $p^1d^{99.99}$	2.16
4	LP_F = $sp^{1.97}$ → LV_{Zr} = $sp^{1.18}d^{99.99}$	7.20
5	LP_F = $sp^{1.97}$ → LV_{Zr} = $sd^{0.19}$	21.81
6	LP_F = $sp^{1.97}$ → LV_{Zr} = $sp^{0.18}d^{88.72}$	3.85
7	σ_{CF} = 0.475($sp^{5.19}$)$_{C1}$ – 0.880($sp^{2.35}$)$_F$ → LV_{Zr} = $sd^{0.19}$	2.22

Natural Resonance Theory:

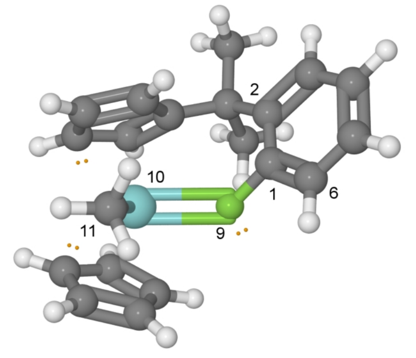

1

Wgt=26.09%;
rhoNL=5.09649;
D(0)=0.09662

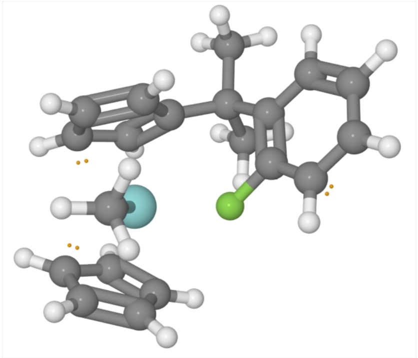

2

Wgt=9.57%;
rhoNL=5.81722;
D(0)=0.10323

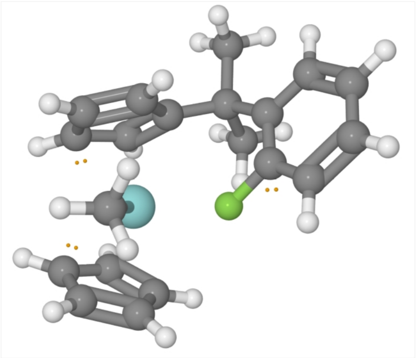

3

Wgt=8.68%;
rhoNL=5.79063;
D(0)=0.10299

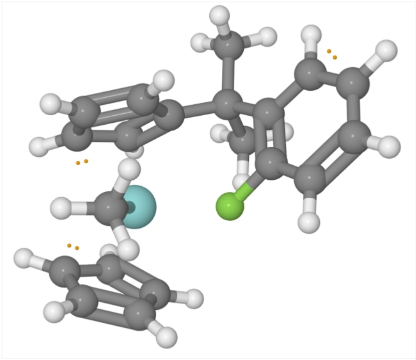

4

Wgt=6.52%;
rhoNL=5.84105;
D(0)=0.10344

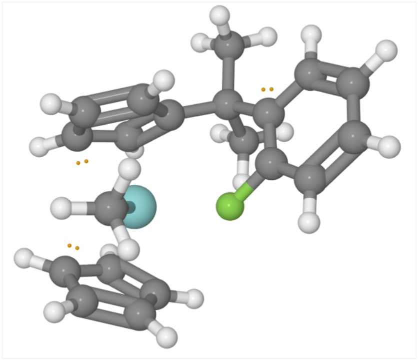

5

Wgt=5.49%;
rhoNL=5.85343;
D(0)=0.10355

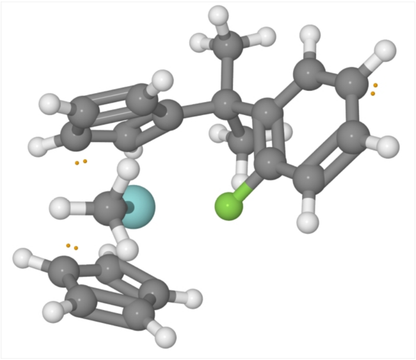

6

Wgt=5.48%;
rhoNL=5.85192;
D(0)=0.10354

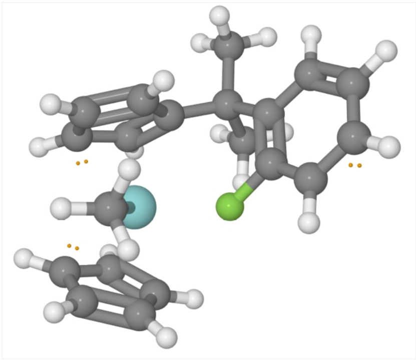

7

Wgt=5.17%;
rhoNL=5.85577;
D(0)=0.10357

Natural Localised Molecular Orbitals (NLMO):

Only contributions over 1% are reported.

NLMO / Occupancy / Percent from Parent NBO / Atomic Hybrid Contributions

Resonance structure **1**:
C-H interaction:
C-C interaction:
Zr-Me interaction:

Resonance structure **1**:
Zr-F interaction:

```
  64. (2.00000)  99.1079% BD ( 1) F  9-Zr 10
                 96.708%  F  9 s( 41.37%)p 1.42( 58.62%)d 0.00(  0.00%)
                  2.647% Zr 10 s( 36.25%)p 0.01(  0.35%)d 1.75( 63.40%)

 65. (2.00000)  98.2661% BD ( 2) F  9-Zr 10
                 97.210%  F  9 s(  0.55%)p99.99( 99.44%)d 0.01(  0.01%)
                  1.130% Zr 10 s( 15.57%)p 0.04(  0.67%)d 5.38( 83.76%)
```

Zr-Me interaction:

```
  66. (2.00000)  97.5942% BD ( 1)Zr 10- C 11
                 21.154% Zr 10 s( 13.63%)p 0.01(  0.08%)d 6.33( 86.29%)
                 76.640%  C 11 s( 25.51%)p 2.92( 74.48%)d 0.00(  0.02%)
```

Resonance structure **2**:
Zr-Me interaction:

```
 66. (2.00000)  97.7818% BD ( 1)Zr 10- C 11
                 21.159% Zr 10 s( 13.51%)p 0.01(  0.08%)d 6.40( 86.41%)
                 76.633%  C 11 s( 25.44%)p 2.93( 74.55%)d 0.00(  0.02%)
```

Resonance structure **3**:
Zr-Me interaction:

```
  66. (2.00000)  97.7818% BD ( 1)Zr 10- C 11
                 21.159% Zr 10 s( 13.51%)p 0.01(  0.08%)d 6.40( 86.41%)
                 76.633%  C 11 s( 25.44%)p 2.93( 74.55%)d 0.00(  0.02%)
```

Resonance structure **4**:
Zr-Me interaction:

```
  66. (2.00000)  97.7818% BD ( 1)Zr 10- C 11
                 21.159% Zr 10 s( 13.51%)p 0.01(  0.08%)d 6.40( 86.41%)
                 76.633%  C 11 s( 25.44%)p 2.93( 74.55%)d 0.00(  0.02%)
```

Resonance structure **5**, **6**, **7**:
NLMO algorithm failed to converge

Non-Covalent Interactions (NCI)

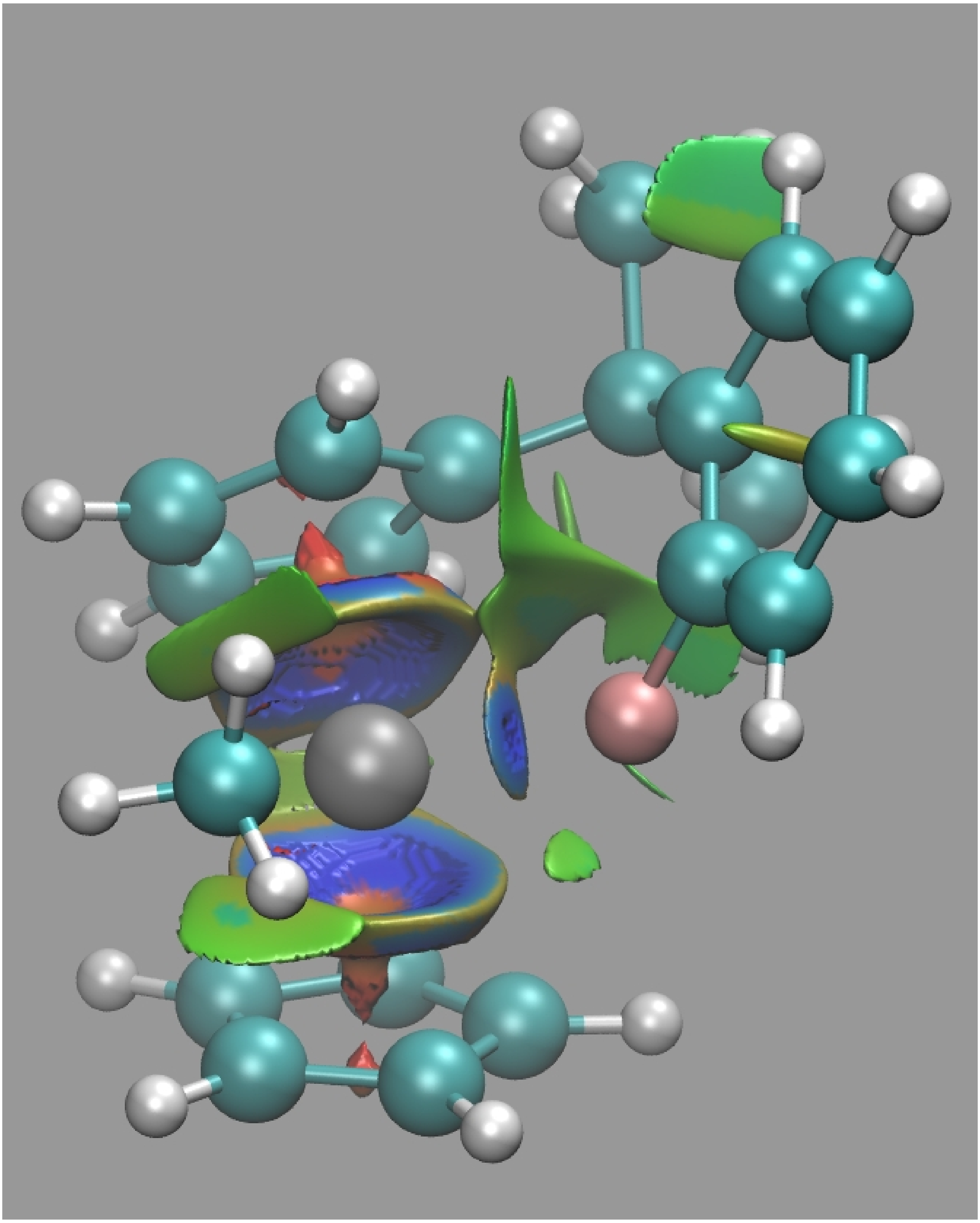

4A-B3LYP

Bader:

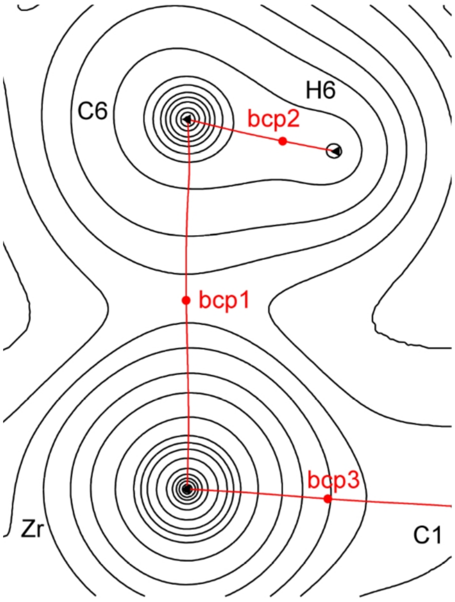

Electrondensity

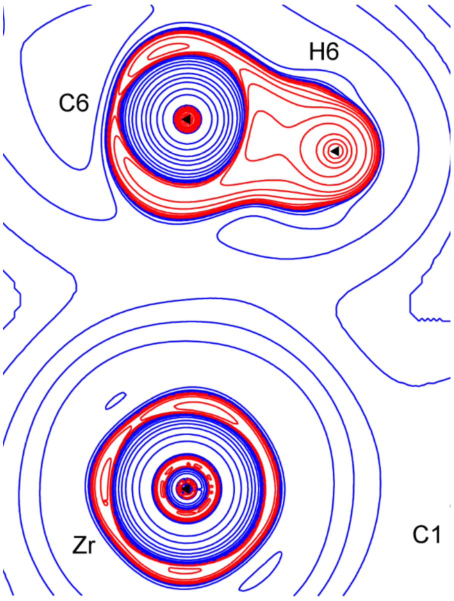

Laplacian

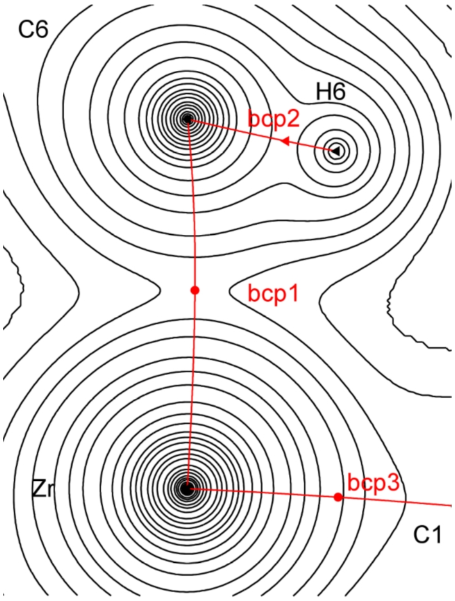

Virial

	ϱ(**r**)	∇^2ϱ(**r**)
bcp1	0.03220	-0.02015
bcp2	0.27550	0.22873
bcp3	0.09486	-0.01212

NBO:

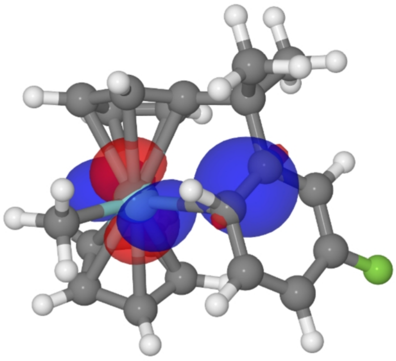

1

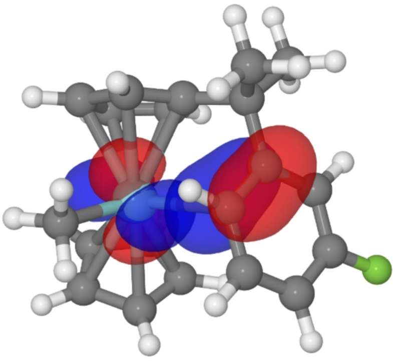

2

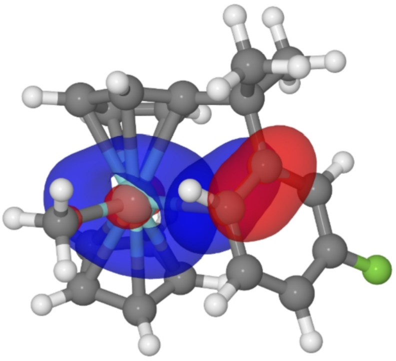

3

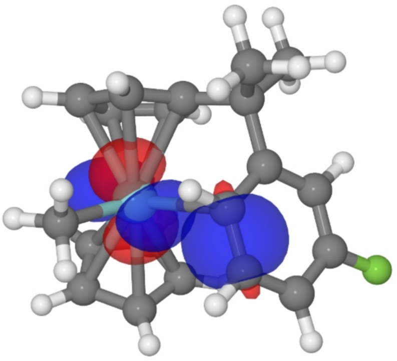

4

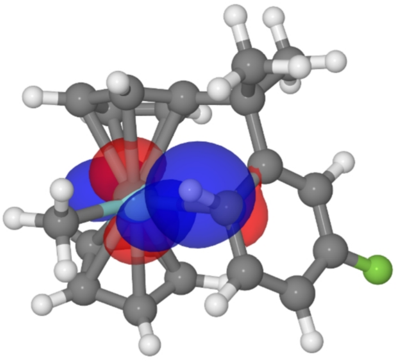

5

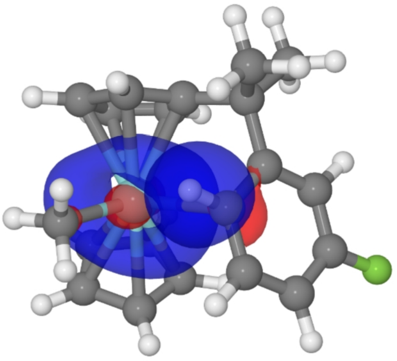

6

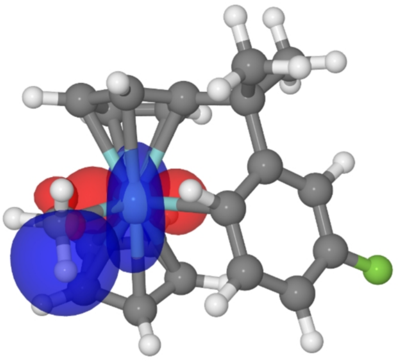

7

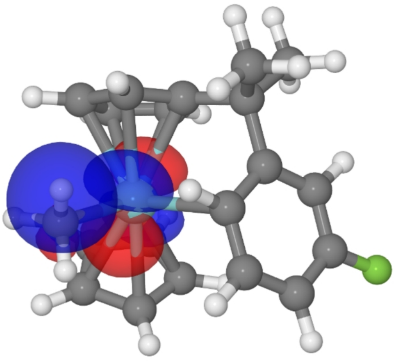

8

	Orbitals	E(2P)
1	σ_{CC} = 0.709$(sp^{1.75})_{C1}$ – 0.705$(sp^{2.01})_{C2}$ → LV_{Zr} = $sd^{42.45}$	2.41
2	π_{CC} = 0.773$(sp^{99.99})_{C1}$ – 0.634$(p^{99.99}d^{0.62})_{C2}$ → LV_{Zr} = $sd^{42.45}$	8.11
3	π_{CC} = 0.773$(sp^{99.99})_{C1}$ – 0.634$(p^{99.99}d^{0.62})_{C2}$ → LV_{Zr} = $sd^{0.32}$	8.32
4	σ_{CC} = 0.714$(sp^{1.81})_{C1}$ – 0.700$(sp^{1.76})_{C6}$ → LV_{Zr} = $sd^{42.45}$	2.80
5	σ_{CH} = 0.789$(sp^{2.61})_{C1}$ - 0.614$(s)_{H23}$ → LV_{Zr} = $sd^{42.45}$	4.97
6	σ_{CH} = 0.789$(sp^{2.61})_{C1}$ - 0.614$(s)_{H23}$ → LV_{Zr} = $sd^{0.32}$	2.80
7	σ_{CH} = 0.776$(sp^{2.98})_{C21}$ - 0.630$(s)_{H43}$ → LV_{Zr} = $sd^{14.33}$	2.26
8	σ_{CH} = 0.776$(sp^{2.95})_{C21}$ 0.631$(s)_{H44}$ → LV_{Zr} = $p^{0.80}d^{99.99}$	2.08

Natural Resonance Theory:

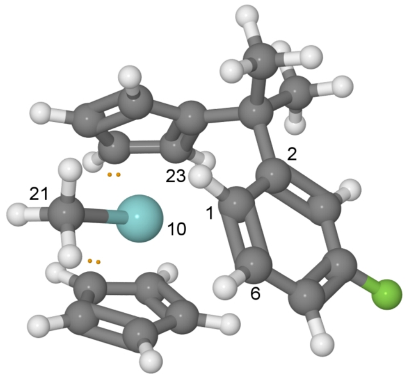

1

Wgt=16.13%;
rhoNL=5.25926;
D(0)=0.09815

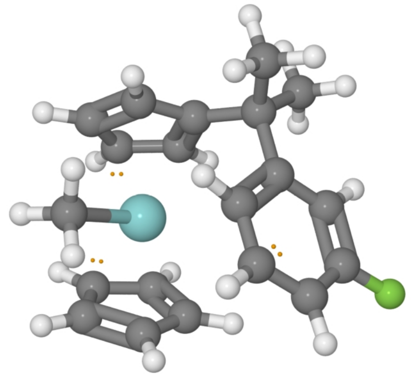

2

Wgt=11.04%;
rhoNL=5.99844;
D(0)=0.10482

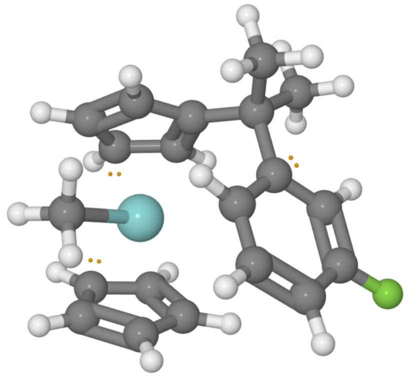

3

Wgt=8.20%;
rhoNL=5.99128;
D(0)=0.10476

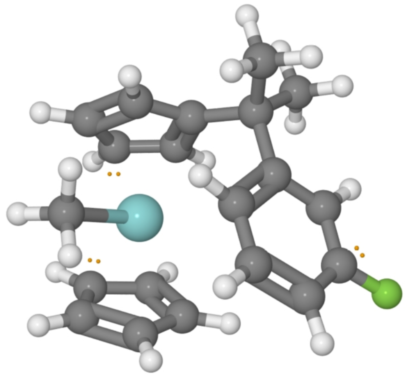

4

Wgt=7.99%;
rhoNL=5.94311;
D(0)=0.10434

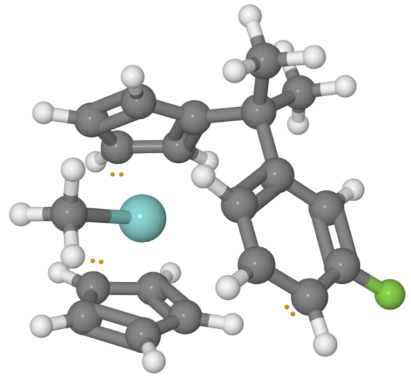

5

Wgt=6.29%;
rhoNL=5.90728;
D(0)=0.10402

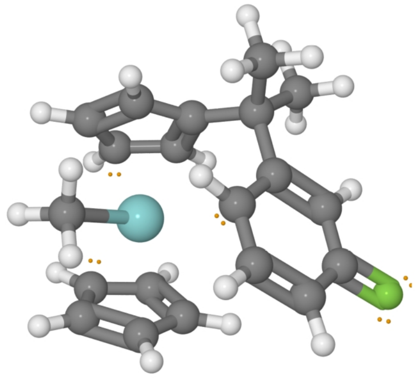

6

Wgt=6.12%;
rhoNL=5.61422;
D(0)=0.10141

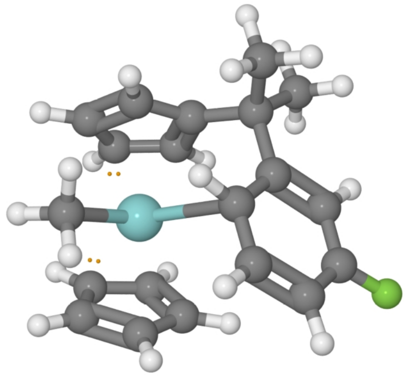

7

Wgt=5.04%;
rhoNL=5.61913;
D(0)=0.10146

Natural Localised Molecular Orbitals (NLMO):

Only contributions over 1% are reported.

NLMO / Occupancy / Percent from Parent NBO / Atomic Hybrid Contributions

Resonance structure **1**:
C-H interaction:

```
  48. (2.00000)  97.6958% BD ( 1) C  1- H 23
                 60.939%  C  1 s( 25.55%)p 2.91( 74.35%)d 0.00(  0.11%)
                  1.003% Zr 10 s( 24.62%)p 0.04(  0.98%)d 3.02( 74.40%)
                 36.815%  H 23 s( 99.96%)p 0.00(  0.04%)
```

C-C interaction:

```
 47. (2.00000)  83.8682% BD ( 2) C  1- C  6
                 50.383%  C  1 s(  0.80%)p99.99( 99.18%)d 0.03(  0.02%)
                  5.369%  C  2 s(  0.16%)p99.99( 99.48%)d 2.21(  0.36%)
                  1.805%  C  3 s(  0.04%)p99.99( 99.79%)d 4.07(  0.17%)
                  1.119%  C  4 s(  0.14%)p99.99( 99.49%)d 2.75(  0.38%)
                  4.371%  C  5 s(  0.00%)p 1.00( 99.65%)d 0.00(  0.35%)
                 33.504%  C  6 s(  0.01%)p99.99( 99.94%)d 3.21(  0.05%)
                  2.854% Zr 10 s( 27.82%)p 0.03(  0.89%)d 2.56( 71.29%)
```

Zr-Me interaction:

```
  66. (2.00000)  97.7841% BD ( 1)Zr 10- C 21
                 19.593% Zr 10 s( 13.42%)p 0.01(  0.08%)d 6.44( 86.50%)
                 78.199%  C 21 s( 25.93%)p 2.86( 74.05%)d 0.00(  0.02%)
```

Resonance structure **2**, **3**, **4**:
NLMO algorithm failed to converge

Resonance structure **5**:
C-H interaction:

```
 49. (2.00000)  97.6897% BD ( 1) C  1- H 23
                 60.951%  C  1 s( 25.55%)p 2.91( 74.35%)d 0.00(  0.10%)
                  1.016% Zr 10 s( 24.56%)p 0.04(  0.96%)d 3.03( 74.48%)
                 36.801%  H 23 s( 99.96%)p 0.00(  0.04%)
```

C-C interaction:

```
 47. (2.00000)  83.0654% BD ( 2) C  1- C  2
                 49.695%  C  1 s(  0.84%)p99.99( 99.13%)d 0.04(  0.03%)
                 33.388%  C  2 s(  0.09%)p99.99( 99.86%)d 0.65(  0.06%)
                  4.303%  C  3 s(  0.00%)p 1.00( 99.65%)d 0.00(  0.35%)
                  1.114%  C  4 s(  0.15%)p99.99( 99.45%)d 2.69(  0.40%)
                  1.731%  C  5 s(  0.12%)p99.99( 99.69%)d 1.67(  0.19%)
                  5.417%  C  6 s(  0.01%)p99.99( 99.66%)d22.50(  0.32%)
                  3.336% Zr 10 s( 25.56%)p 0.03(  0.88%)d 2.88( 73.56%)
```

Zr-Me interaction:

```
 66. (2.00000)  97.7843% BD ( 1)Zr 10- C 21
                19.594% Zr 10 s( 13.42%)p 0.01(  0.08%)d 6.44( 86.50%)
                78.199%  C 21 s( 25.93%)p 2.86( 74.05%)d 0.00(  0.02%)
```

Resonance structure **6**:

C-H interaction:

```
 47. (2.00000)  97.7294% BD ( 1) C  1- H 23
                61.024%  C  1 s( 25.67%)p 2.89( 74.23%)d 0.00(  0.10%)
                 1.071% Zr 10 s( 25.12%)p 0.04(  0.91%)d 2.95( 73.98%)
                36.745%  H 23 s( 99.96%)p 0.00(  0.04%)
```

Zr-Me interaction:

```
 66. (2.00000)  97.7844% BD ( 1)Zr 10- C 21
                19.593% Zr 10 s( 13.42%)p 0.01(  0.08%)d 6.44( 86.50%)
                78.200%  C 21 s( 25.93%)p 2.86( 74.05%)d 0.00(  0.02%)
```

Resonance structure **7**:

Zr-C interaction:

```
 47. (2.00000)  60.4597% BD ( 1) C  1-Zr 10
                56.381%  C  1 s(  0.69%)p99.99( 99.30%)d 0.02(  0.01%)
                13.201%  C  2 s(  0.09%)p99.99( 99.74%)d 1.78(  0.17%)
                 1.410%  C  3 s(  0.09%)p99.99( 99.21%)d 8.00(  0.70%)
                 9.429%  C  4 s(  0.06%)p99.99( 99.93%)d 0.10(  0.01%)
                 1.487%  C  5 s(  0.16%)p99.99( 99.15%)d 4.17(  0.68%)
                13.146%  C  6 s(  0.01%)p 1.00( 99.84%)d 0.00(  0.15%)
                 4.240% Zr 10 s( 25.87%)p 0.03(  0.81%)d 2.83( 73.32%)
```

C-H interaction:

```
 48. (2.00000)  97.7298% BD ( 1) C  1- H 23
                61.014%  C  1 s( 25.65%)p 2.89( 74.24%)d 0.00(  0.10%)
                 1.054% Zr 10 s( 25.65%)p 0.04(  0.92%)d 2.86( 73.43%)
                36.756%  H 23 s( 99.96%)p 0.00(  0.04%)
```

Zr-Me interaction:

```
 66. (2.00000)  97.7840% BD ( 1)Zr 10- C 21
                19.595% Zr 10 s( 13.42%)p 0.01(  0.08%)d 6.44( 86.50%)
                78.197%  C 21 s( 25.93%)p 2.86( 74.06%)d 0.00(  0.02%)
```

Non-Covalent Interactions (NCI)

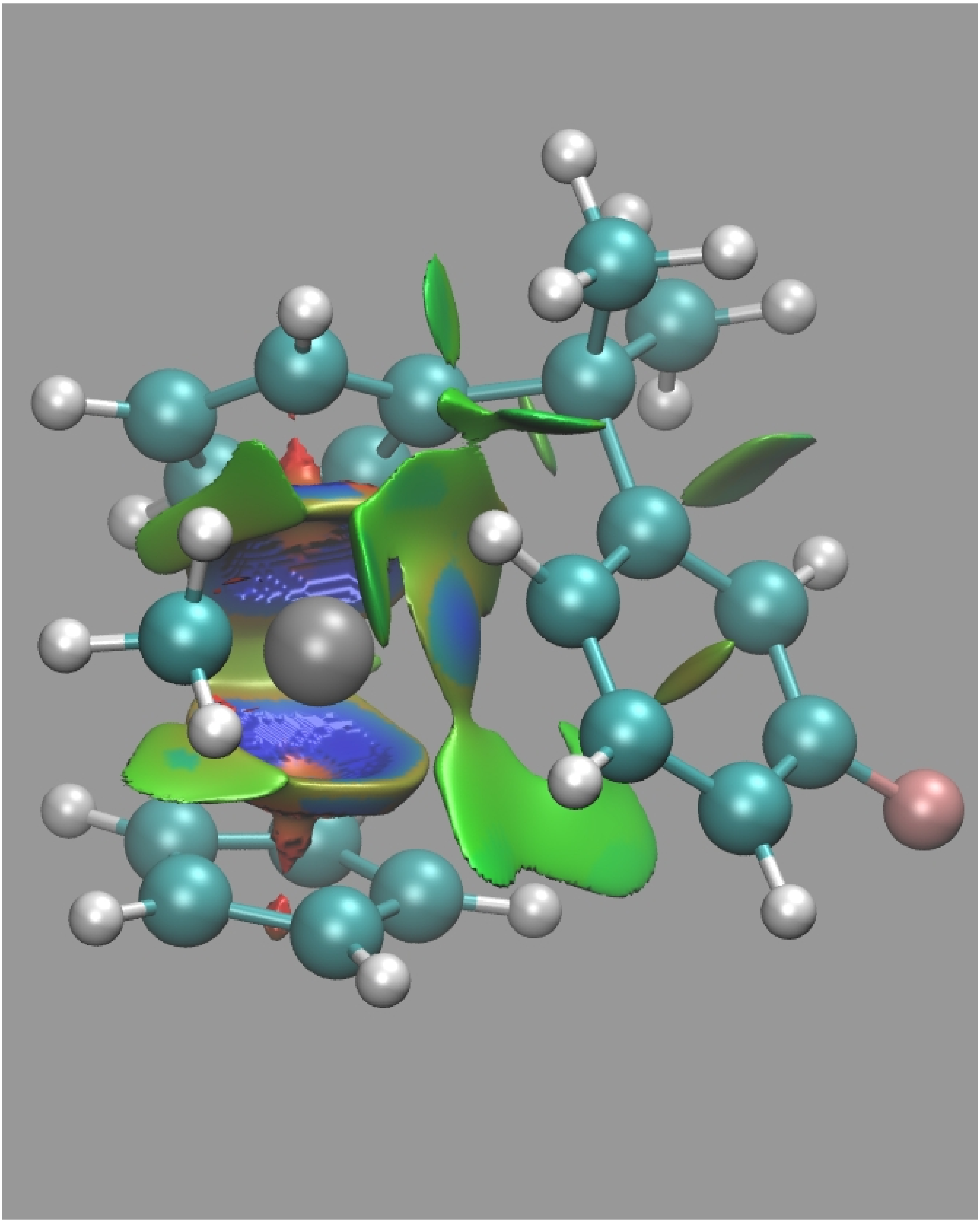

4B-B3LYP

Bader:

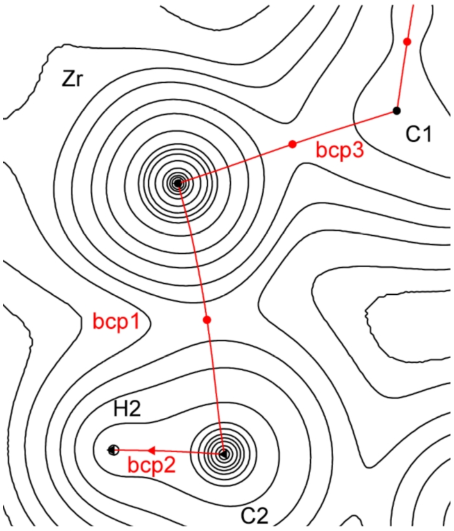

Electrondensity

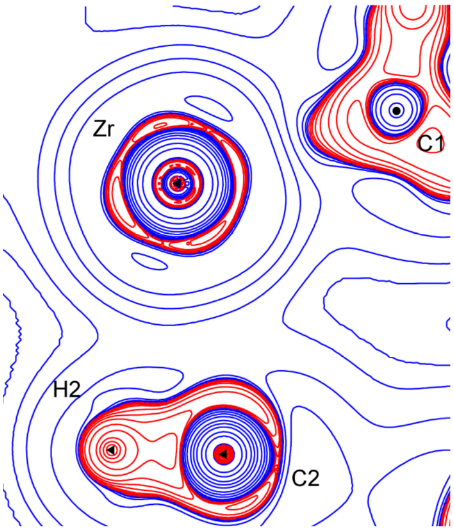

Laplacian

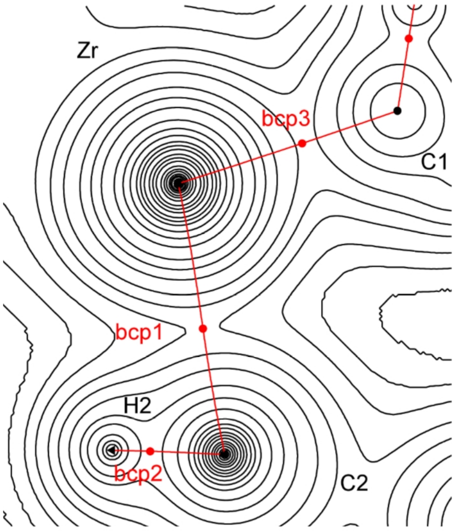

Virial

	ϱ(**r**)	∇^2ϱ(**r**)
bcp1	0.03089	-0.01956
bcp2	0.27176	0.22263
bcp3	0.09595	-0.01173

NBO:

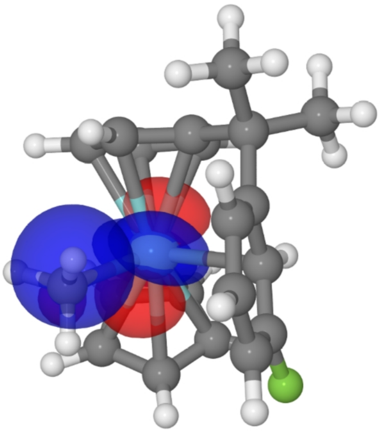

1

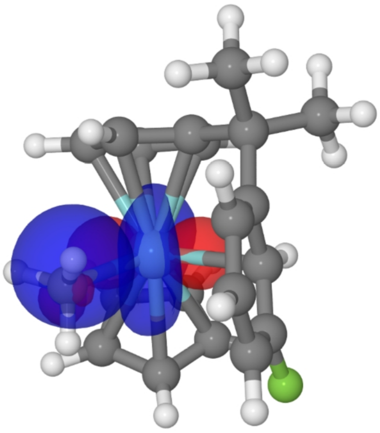

2

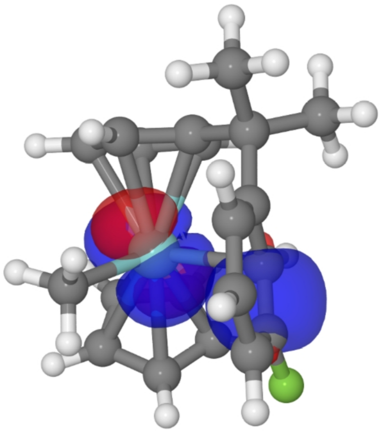

3

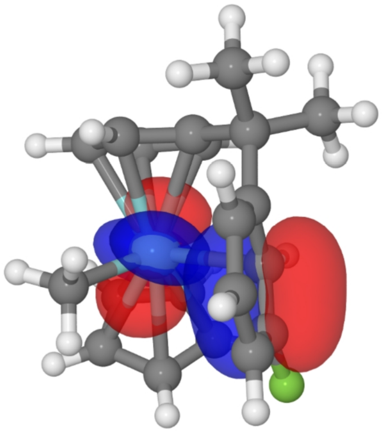

4

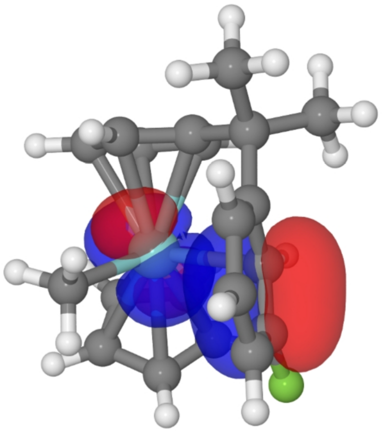

5

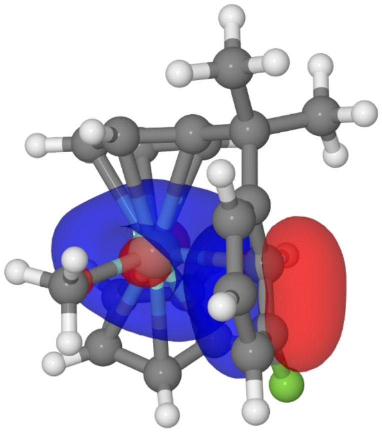

6

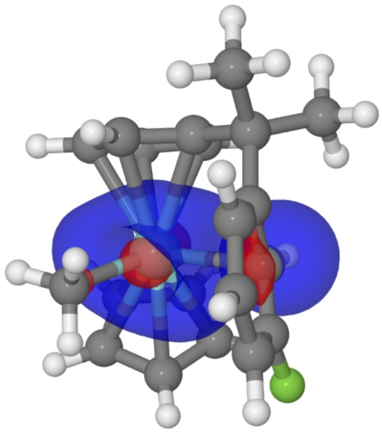

7

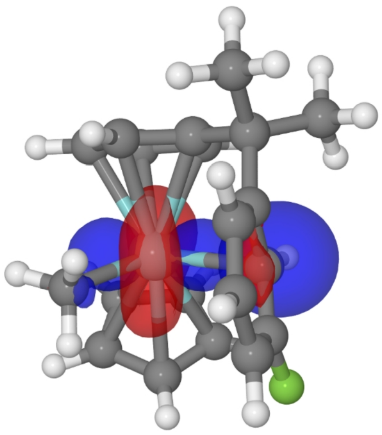

8

	Orbitals	E(2P)
1	$\sigma_{CH} = 0.776(sp^{2.97})_{C7} – 0.630(s)_{H37} \rightarrow$ $LV_{Zr} = sp^{0.14}d^{99.99}$	2.04
2	$\sigma_{CH} = 0.776(sp^{2.97})_{C7} – 0.630(s)_{H37} \rightarrow$ $LV_{Zr} = sp^{0.05}d^{12.96}$	2.80
3	$\sigma_{CC} = 0.716(sp^{1.92})_{C8} – 0.698(sp^{1.58})_{C9} \rightarrow$ $LV_{Zr} = sp^{0.06}d^{67.46}$	2.02
4	$\pi_{CC} = 0.781(sp^{99.99})_{C8} – 0.625(p^{99.99}d^{2.57})_{C9} \rightarrow$ $LV_{Zr} = sp^{0.14}d^{99.99}$	2.34
5	$\pi_{CC} = 0.781(sp^{99.99})_{C8} – 0.625(p^{99.99}d^{2.57})_{C9} \rightarrow$ $LV_{Zr} = sp^{0.06}d^{67.46}$	5.56
6	$\pi_{CC} = 0.781(sp^{99.99})_{C8} – 0.625(p^{99.99}d^{2.57})_{C9} \rightarrow$ $LV_{Zr} = sd^{0.31}$	6.51
7	$\sigma_{CH} = 0.790(sp^{2.59})_{C8} – 0.613(H)_{26} \rightarrow$ $LV_{Zr} = sd^{0.31}$	4.76
8	$\sigma_{CH} = 0.790(sp^{2.59})_{C8} – 0.613(H)_{26} \rightarrow$ $LV_{Zr} = sp^{0.05}d^{12.96}$	2.98

Natural Resonance Theory:

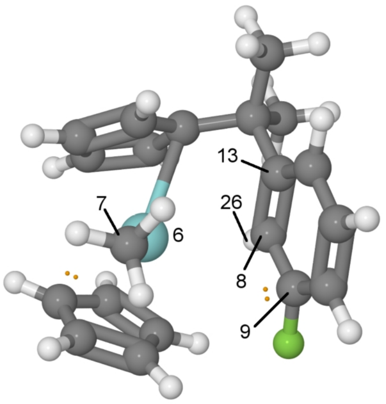

1

Wgt=16.68%;
rhoNL=5.78276;
D(0)=0.1029

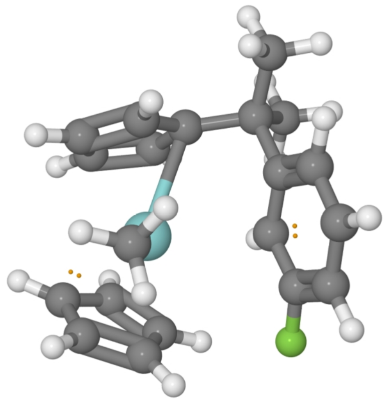

2

Wgt=13.67%;
rhoNL=5.54658;
D(0)=0.10080

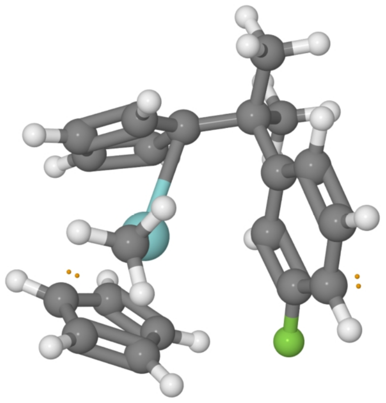

3

Wgt=13.08%;
rhoNL=5.71284;
D(0)=0.10230

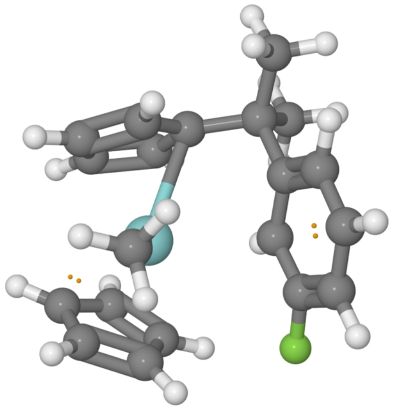

4

Wgt=12.80%;
rhoNL=5.85694;
D(0)=0.10358

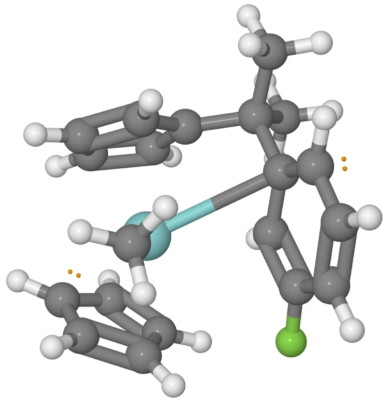

5

Wgt=12.21%;
rhoNL=6.00278;
D(0)=0.10486

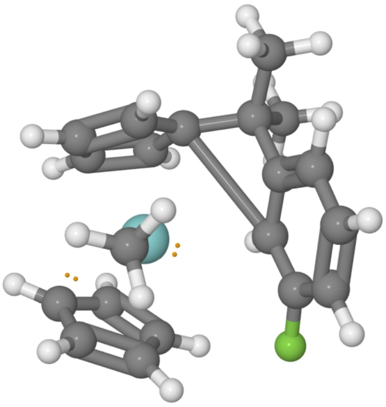

6

Wgt=10.78%;
rhoNL=6.46170;
D(0)=0.10880

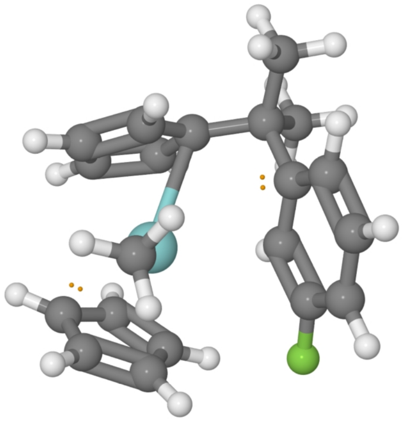

7

Wgt=9.37%;
rhoNL=5.80228;
D(0)=0.10310

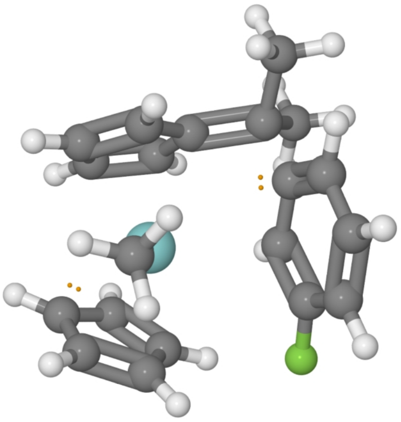

8

Wgt=6.36%;
rhoNL=6.11529;
D(0)=0.10584

Natural Localised Molecular Orbitals (NLMO):

Only contributions over 1% are reported.

NLMO / Occupancy / Percent from Parent NBO / Atomic Hybrid Contributions

Resonance structure **1**:
C-H interaction:
```
 65. (2.00000)  97.3918% BD ( 1) C  8- H 26
                  1.298% Zr  6 s( 24.28%)p 0.03(  0.67%)d 3.09( 75.05%)
                 60.925%  C  8 s( 25.79%)p 2.87( 74.11%)d 0.00(  0.10%)
                 36.525%  H 26 s( 99.96%)p 0.00(  0.04%)
```

C-C interaction:
```
 64. (2.00000)  83.6081% BD ( 2) C  8- C 13
                  2.924% Zr  6 s( 29.00%)p 0.03(  0.88%)d 2.42( 70.12%)
                 51.648%  C  8 s(  1.00%)p99.23( 98.98%)d 0.02(  0.02%)
                  5.496%  C  9 s(  0.01%)p 1.00( 99.55%)d 0.00(  0.44%)
                  1.574%  C 10 s(  0.11%)p99.99( 99.75%)d 1.37(  0.15%)
                  1.051%  C 11 s(  0.12%)p99.99( 99.55%)d 2.93(  0.34%)
                  4.347%  C 12 s(  0.00%)p 1.00( 99.65%)d 0.00(  0.35%)
                 31.978%  C 13 s(  0.12%)p99.99( 99.83%)d 0.43(  0.05%)
```

Zr-Me interaction:
```
 57. (2.00000)  97.8535% BD ( 1)Zr  6- C  7
                 20.002% Zr  6 s( 12.83%)p 0.01(  0.08%)d 6.79( 87.09%)
                 77.861%  C  7 s( 25.71%)p 2.89( 74.28%)d 0.00(  0.02%)
```

Resonance structure **2**:
C-H interaction:
```
 64. (2.00000)  97.4225% BD ( 1) C  8- H 26
                  1.353% Zr  6 s( 24.71%)p 0.03(  0.64%)d 3.02( 74.65%)
                 60.992%  C  8 s( 25.93%)p 2.85( 73.97%)d 0.00(  0.10%)
                 36.469%  H 26 s( 99.96%)p 0.00(  0.04%)
```

Zr-Me interaction:
```
 57. (2.00000)  97.8537% BD ( 1)Zr  6- C  7
                 20.002% Zr  6 s( 12.83%)p 0.01(  0.08%)d 6.79( 87.09%)
                 77.861%  C  7 s( 25.71%)p 2.89( 74.28%)d 0.00(  0.02%)
```

Resonance structure **3**:
C-H interaction:
```
 65. (2.00000)  97.3895% BD ( 1) C  8- H 26
                  1.290% Zr  6 s( 24.24%)p 0.03(  0.67%)d 3.10( 75.09%)
                 60.915%  C  8 s( 25.77%)p 2.88( 74.13%)d 0.00(  0.10%)
                 36.534%  H 26 s( 99.96%)p 0.00(  0.04%)
```

C-C interaction:
```
  63. (2.00000)  84.6246% BD ( 2) C  8- C  9
                  2.807% Zr  6 s( 29.91%)p 0.03(  0.81%)d 2.32( 69.28%)
                 52.600%  C  8 s(  1.04%)p94.88( 98.93%)d 0.02(  0.02%)
                 32.056%  C  9 s(  0.01%)p99.99( 99.93%)d 3.97(  0.06%)
                  3.918%  C 10 s(  0.01%)p 1.00( 99.56%)d 0.00(  0.43%)
                  1.053%  C 11 s(  0.12%)p99.99( 99.60%)d 2.46(  0.28%)
                  1.353%  C 12 s(  0.11%)p99.99( 99.67%)d 1.93(  0.22%)
                  5.619%  C 13 s(  0.16%)p99.99( 99.53%)d 1.99(  0.31%)
```
Zr-Me interaction:
```
  57. (2.00000)  97.8538% BD ( 1)Zr  6- C  7
                 20.001% Zr  6 s( 12.84%)p 0.01(  0.08%)d 6.78( 87.09%)
                 77.863%  C  7 s( 25.71%)p 2.89( 74.28%)d 0.00(  0.02%)
```

Resonance structure **4**, **5**, **6**:
NLMO algorithm failed to converge

Resonance structure **7**:
C-H interaction:
```
  65. (2.00000)  97.3874% BD ( 1) C  8- H 26
                  1.294% Zr  6 s( 24.18%)p 0.03(  0.67%)d 3.11( 75.15%)
                 60.919%  C  8 s( 25.77%)p 2.88( 74.13%)d 0.00(  0.10%)
                 36.528%  H 26 s( 99.96%)p 0.00(  0.04%)
```

C-C interaction:
```
  63. (2.00000)  84.4884% BD ( 2) C  8- C  9
                  2.708% Zr  6 s( 29.94%)p 0.03(  0.79%)d 2.31( 69.26%)
                 51.567%  C  8 s(  1.04%)p95.32( 98.94%)d 0.03(  0.03%)
                 32.939%  C  9 s(  0.01%)p99.99( 99.93%)d 4.00(  0.06%)
                  5.202%  C 10 s(  0.00%)p 1.00( 99.65%)d 0.00(  0.34%)
                  1.095%  C 12 s(  0.13%)p99.99( 99.62%)d 2.00(  0.25%)
                  5.424%  C 13 s(  0.16%)p99.99( 99.53%)d 2.00(  0.31%)
```

Zr-Me interaction:
```
  57. (2.00000)  97.8535% BD ( 1)Zr  6- C  7
                 20.000% Zr  6 s( 12.83%)p 0.01(  0.08%)d 6.79( 87.09%)
                 77.863%  C  7 s( 25.71%)p 2.89( 74.28%)d 0.00(  0.02%)
```

Resonance structure **8**:
C-H interaction:
```
  64. (2.00000)  97.3913% BD ( 1) C  8- H 26
                  1.289% Zr  6 s( 23.87%)p 0.03(  0.68%)d 3.16( 75.45%)
                 60.911%  C  8 s( 25.80%)p 2.87( 74.10%)d 0.00(  0.10%)
                 36.539%  H 26 s( 99.96%)p 0.00(  0.04%)
```

C-C interaction:

```
  62. (2.00000)  84.5485% BD ( 2) C  8- C  9
                  2.797% Zr  6 s( 29.02%)p 0.03(  0.81%)d 2.42( 70.17%)
                 52.301%  C  8 s(  1.03%)p95.64( 98.94%)d 0.02(  0.02%)
                 32.273%  C  9 s(  0.01%)p99.99( 99.93%)d 4.49(  0.06%)
                  4.151%  C 10 s(  0.00%)p 1.00( 99.58%)d 0.00(  0.42%)
                  1.129%  C 11 s(  0.12%)p99.99( 99.59%)d 2.39(  0.29%)
                  1.667%  C 12 s(  0.10%)p99.99( 99.74%)d 1.54(  0.16%)
                  5.139%  C 13 s(  0.15%)p99.99( 99.50%)d 2.26(  0.34%)
```

Zr-Me interaction:

```
  57. (2.00000)  97.8513% BD ( 1)Zr  6- C  7
                 20.028% Zr  6 s( 12.87%)p 0.01(  0.08%)d 6.77( 87.06%)
                 77.833%  C  7 s( 25.71%)p 2.89( 74.28%)d 0.00(  0.02%)
```

Non-Covalent Interactions (NCI)

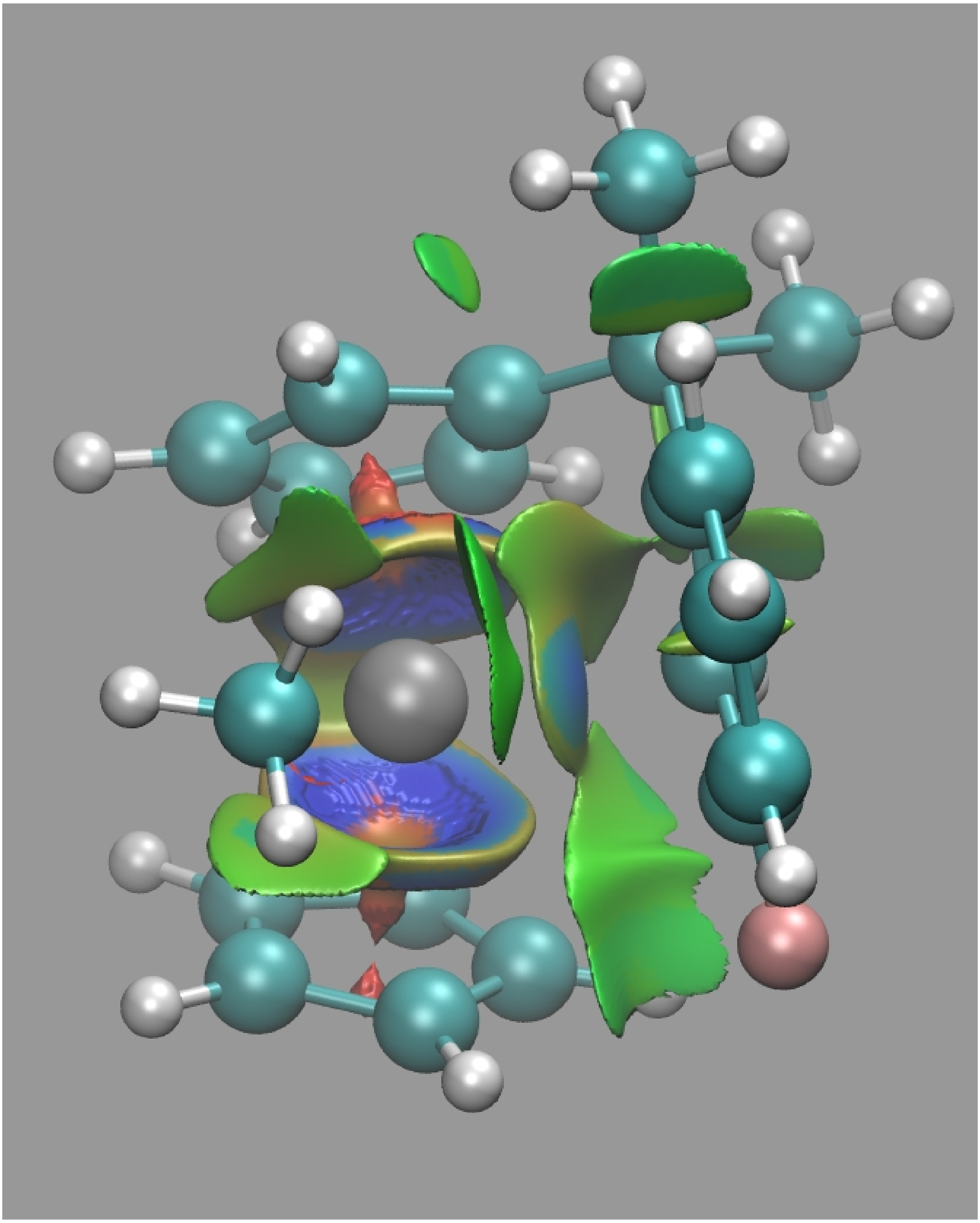

5A-B3LYP

Bader:

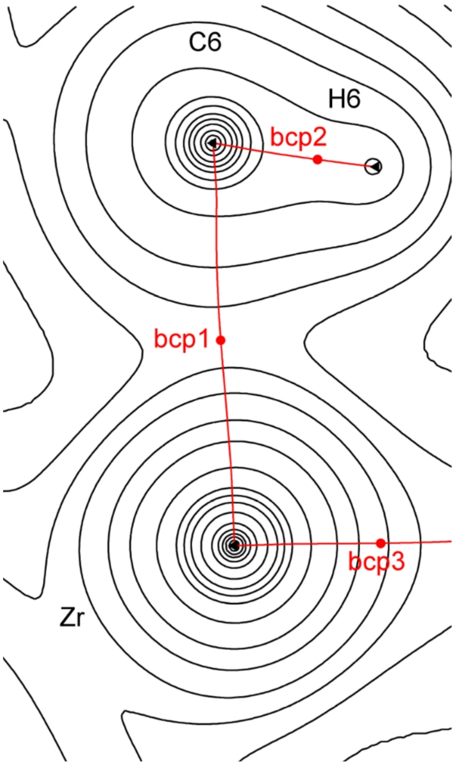

Electrondensity

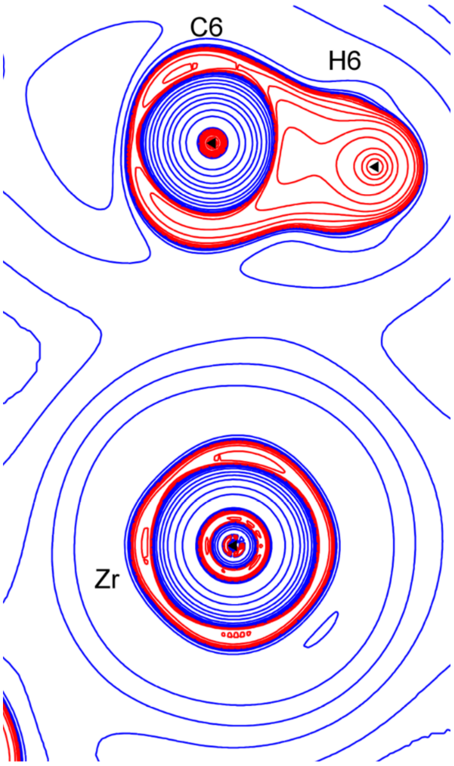

Laplacian

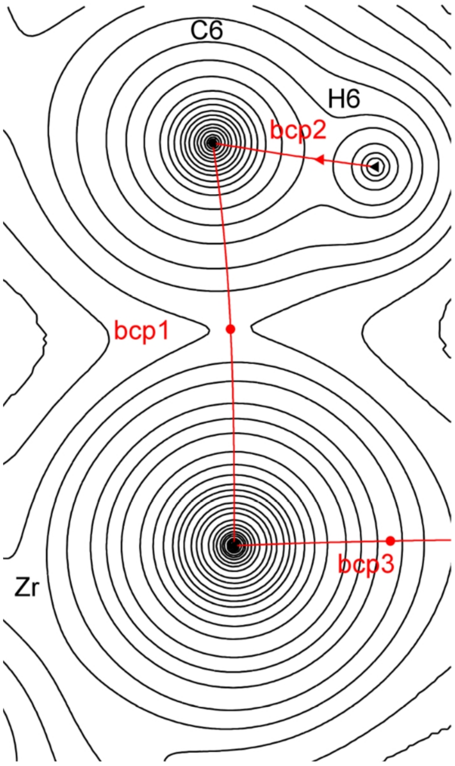

Virial

	ϱ(**r**)	∇^2ϱ(**r**)
bcp1	0.02996	-0.01979
bcp2	0.27738	0.23261
bcp3	0.09471	-0.01201

NBO:

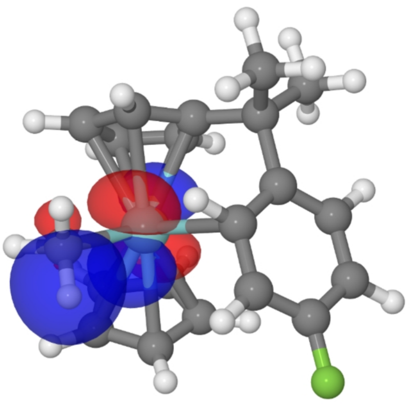

1

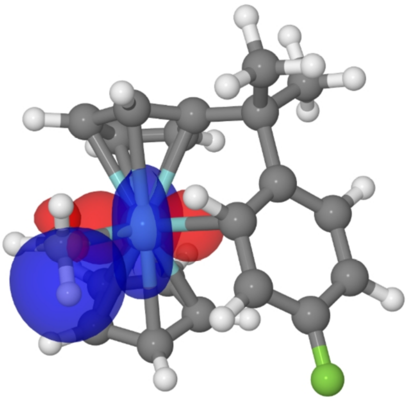

2

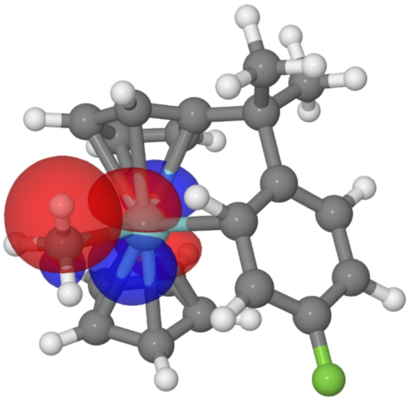

3

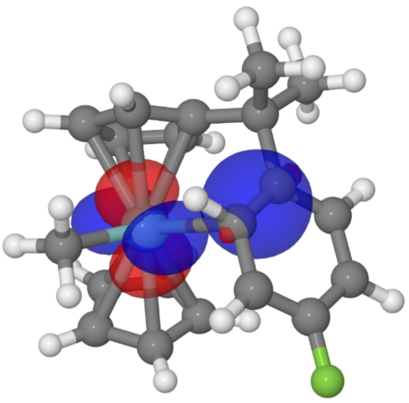

4

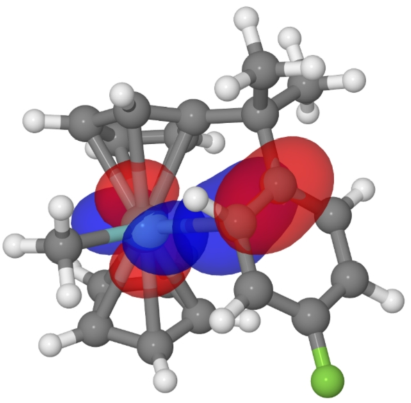

5

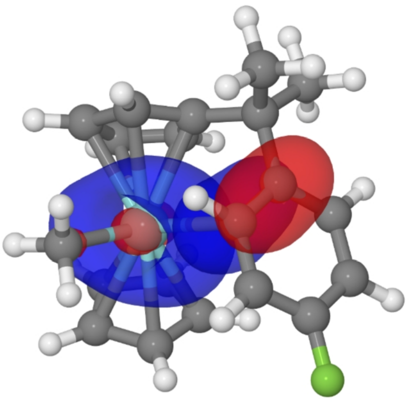

6

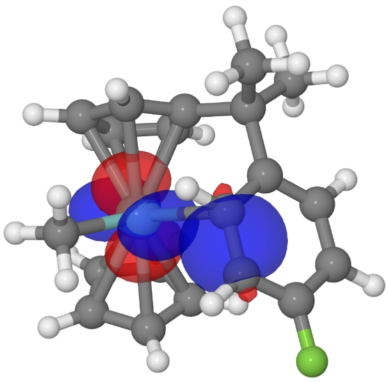

7

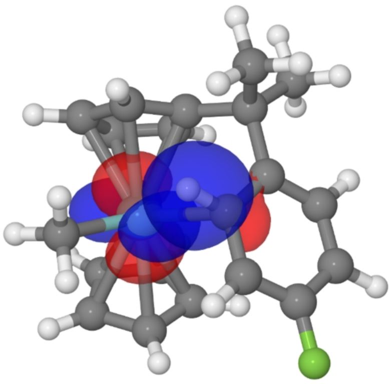

8

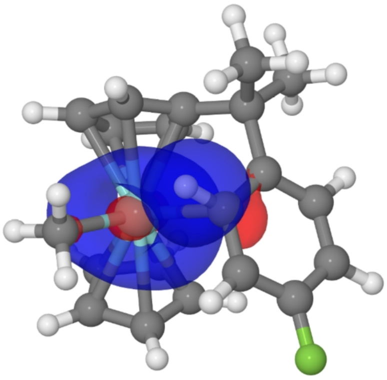

9

	Orbitals	E(2P)
1	$\sigma_{CH} = 0.776(sp^{2.97})_{C7} - 0.630(s)_{H43} \rightarrow$ $LV_{Zr} = p^{1.84}d^{99.99}$	2.01
2	$\sigma_{CH} = 0.776(sp^{2.97})_{C7} - 0.630(s)_{H43} \rightarrow$ $LV_{Zr} = sd^{17.47}$	2.19
3	$\sigma_{CH} = 0.776(sp^{2.95})_{C7} - 0.631(s)_{H44} \rightarrow$ $LV_{Zr} = p^{1.84}d^{99.99}$	2.08
4	$\sigma_{CC} = 0.710(sp^{1.71})_{C15} - 0.704(sp^{2.03})_{C16} \rightarrow$ $LV_{Zr} = sp^{0.05}d^{55.75}$	2.43
5	$\pi_{CC} = 0.747(sp^{99.99})_{C15} - 0.664(sp^{99.99}d^{0.74})_{C16} \rightarrow$ $LV_{Zr} = sp^{0.05}d^{55.75}$	6.64
6	$\pi_{CC} = 0.747(sp^{99.99})_{C15} - 0.664(sp^{99.99}d^{0.74})_{C16} \rightarrow$ $LV_{Zr} = sd^{0.29}$	7.78
7	$\sigma_{CC} = 0.711(sp^{1.83})_{C15} - 0.703(sp^{1.73})_{C20} \rightarrow$ $LV_{Zr} = sp^{0.05}d^{55.75}$	2.86
8	$\sigma_{CH} = 0.790(sp^{2.62})_{C15} - 0.613(s)_{H24} \rightarrow$ $LV_{Zr} = sp^{0.05}d^{55.75}$	4.77
9	$\sigma_{CH} = 0.790(sp^{2.62})_{C15} - 0.613(s)_{H24} \rightarrow$ $LV_{Zr} = sd^{0.29}$	2.69

Natural Resonance Theory:

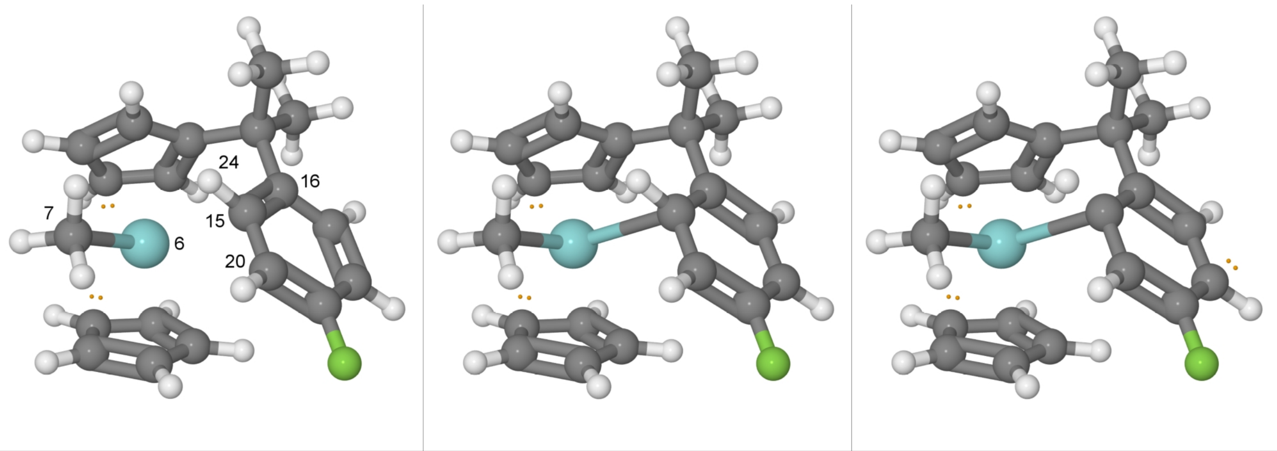

Wgt=39.50%; rhoNL=5.24569; D(0)=0.0980	Wgt=26.11%; rhoNL=5.72624; D(0)=0.10242	Wgt=6.33%; rhoNL=6.62684; D(0)=0.11018

Natural Localised Molecular Orbitals (NLMO):

Only contributions over 1% are reported.

NLMO / Occupancy / Percent from Parent NBO / Atomic Hybrid Contributions

Resonance structure **1**:
C-H interaction:

```
 82. (2.00000)  97.8034% BD ( 1) C 15- H 24
                  0.926% Zr  6 s( 24.21%)p 0.04(  0.96%)d 3.09( 74.83%)
                 61.021%  C 15 s( 25.39%)p 2.93( 74.50%)d 0.00(  0.10%)
                 36.838%  H 24 s( 99.95%)p 0.00(  0.05%)
```

C-C interaction:

```
 80. (2.00000)  83.3587% BD ( 2) C 15- C 16
                  3.122% Zr  6 s( 26.02%)p 0.04(  0.98%)d 2.81( 73.01%)
                 46.917%  C 15 s(  0.55%)p99.99( 99.41%)d 0.07(  0.04%)
                 36.459%  C 16 s(  0.07%)p99.99( 99.88%)d 0.77(  0.05%)
                  4.988%  C 17 s(  0.00%)p 1.00( 99.67%)d 0.00(  0.33%)
                  1.280%  C 18 s(  0.07%)p99.99( 99.60%)d 4.56(  0.33%)
                  1.493%  C 19 s(  0.16%)p99.99( 99.60%)d 1.46(  0.23%)
                  4.736%  C 20 s(  0.01%)p 1.00( 99.66%)d 0.00(  0.34%)
```

Zr-Me interaction:

```
 57. (2.00000)  97.7692% BD ( 1)Zr  6- C  7
                 19.575% Zr  6 s( 13.09%)p 0.01(  0.08%)d 6.63( 86.83%)
                 78.203%  C  7 s( 25.95%)p 2.85( 74.04%)d 0.00(  0.02%)
```

Resonance structure **2**:
C-H interaction:
82. (2.00000) 97.8347% BD (1) C 15- H 24
0.957% Zr 6 s(25.02%)p 0.04(0.93%)d 2.96(74.05%)
61.067% C 15 s(25.44%)p 2.93(74.46%)d 0.00(0.10%)
36.806% H 24 s(99.95%)p 0.00(0.05%)

Zr-C interaction:
58. (2.00000) 57.3848% BD (1)Zr 6- C 15
3.766% Zr 6 s(26.59%)p 0.03(0.91%)d 2.73(72.50%)
53.768% C 15 s(0.44%)p99.99(99.54%)d 0.04(0.02%)
14.319% C 16 s(0.08%)p99.99(99.76%)d 1.90(0.15%)
1.358% C 17 s(0.07%)p99.99(99.17%)d10.06(0.75%)
11.174% C 18 s(0.04%)p99.99(99.95%)d 0.23(0.01%)
1.238% C 19 s(0.27%)p99.99(98.60%)d 4.25(1.13%)
13.729% C 20 s(0.01%)p 1.00(99.87%)d 0.00(0.13%)

57. (2.00000) 97.7694% BD (1)Zr 6- C 7
19.573% Zr 6 s(13.10%)p 0.01(0.08%)d 6.63(86.83%)
78.205% C 7 s(25.94%)p 2.85(74.04%)d 0.00(0.02%)

Resonance structure **3**:

Zr-C interaction:
59. (2.00000) 59.7781% BD (1)Zr 6- C 15
2.608% Zr 6 s(28.23%)p 0.01(0.41%)d 2.53(71.36%)
61.343% C 15 s(25.92%)p 2.85(74.00%)d 0.00(0.08%)
2.543% C 16 s(0.42%)p99.99(99.40%)d 0.42(0.18%)
2.375% C 20 s(0.29%)p99.99(99.50%)d 0.72(0.21%)
30.042% H 24 s(99.95%)p 0.00(0.05%)

Zr-Me interaction:
58. (2.00000) 97.7692% BD (1)Zr 6- C 7
19.573% Zr 6 s(13.09%)p 0.01(0.08%)d 6.63(86.83%)
78.205% C 7 s(25.94%)p 2.85(74.04%)d 0.00(0.02%)

Non-Covalent Interactions (NCI)

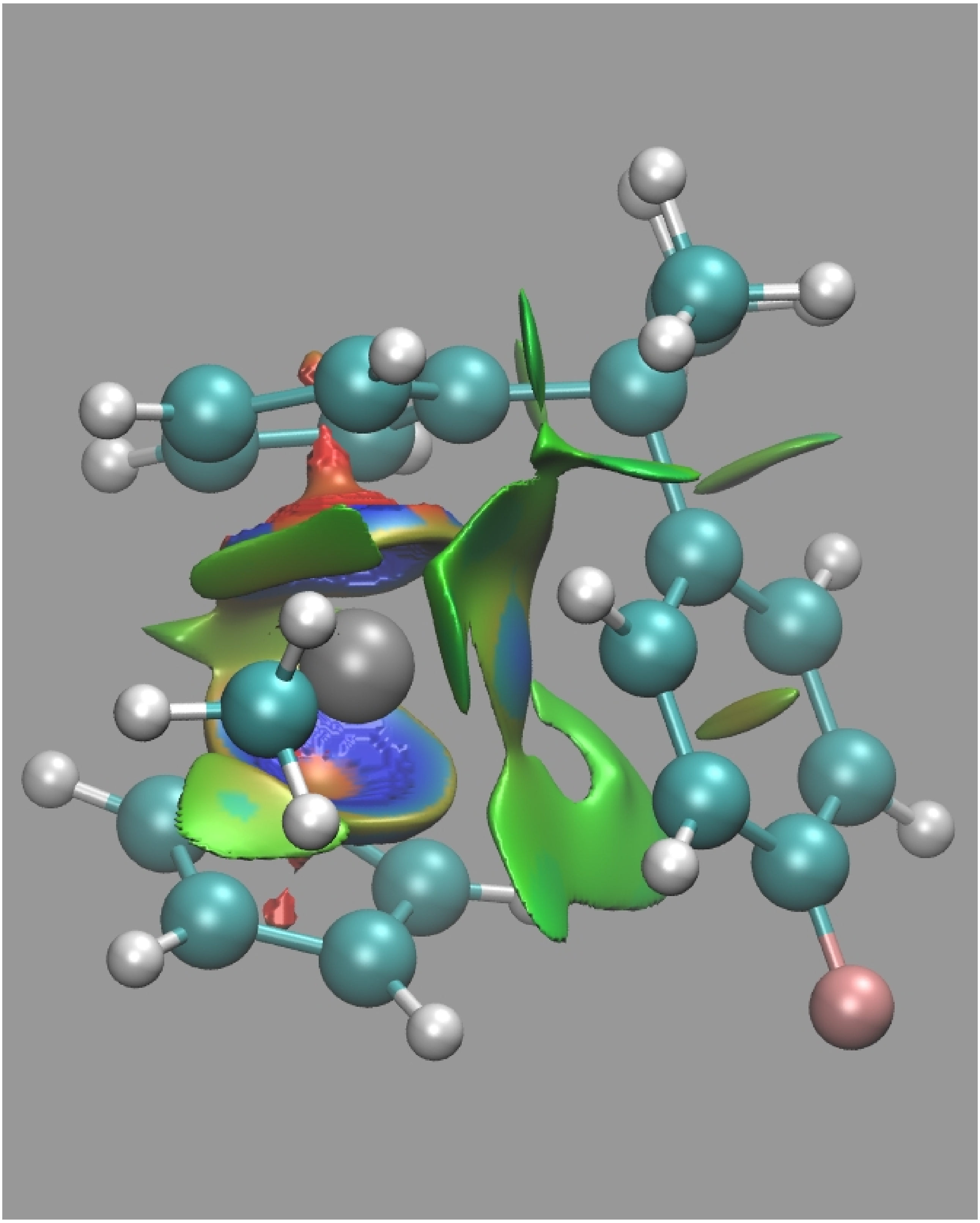

5B-B3LYP

Bader:

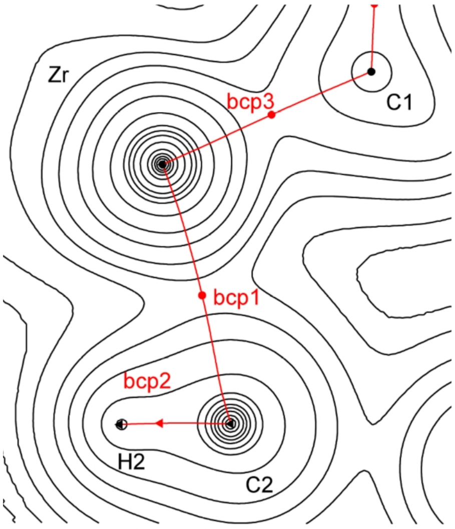

Electrondensity

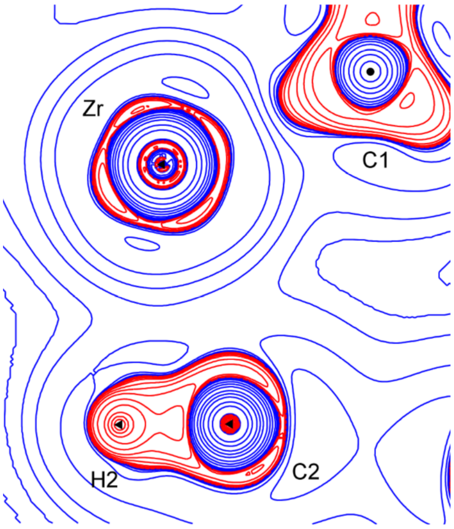

Laplacian

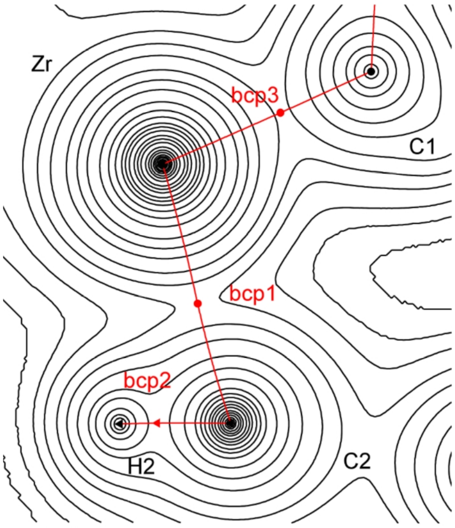

Virial

	$\varrho(\mathbf{r})$	$\nabla^2\varrho(\mathbf{r})$
bcp1	0.03128	-0.02043
bcp2	0.27141	0.22242
bcp3	0.09612	-0.01149

NBO:

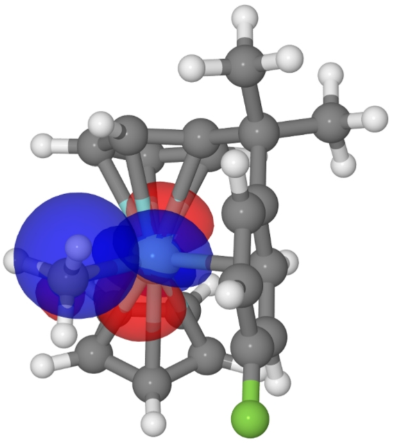

1

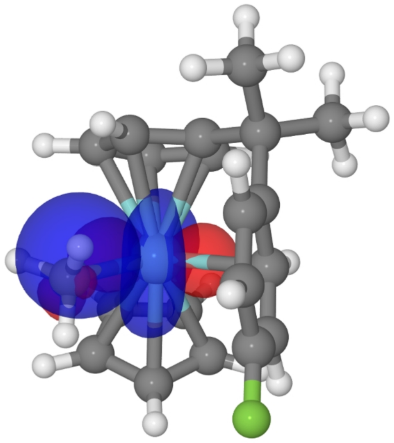

2

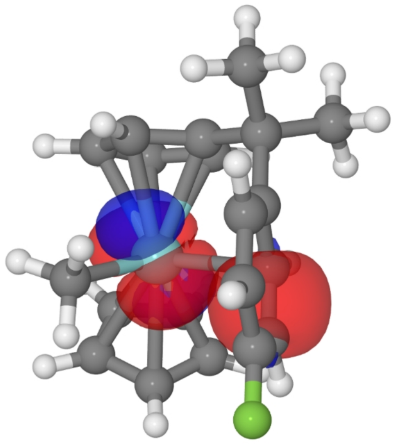

3

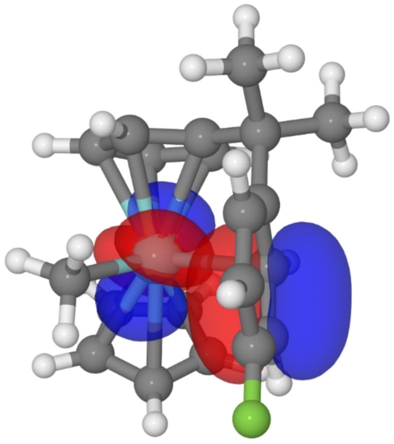

4

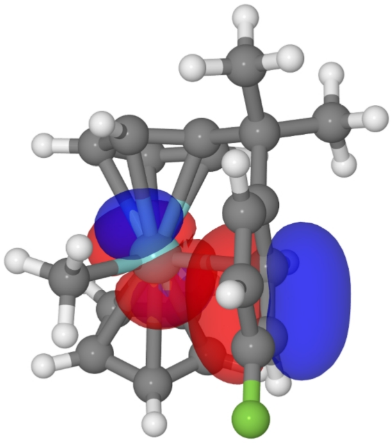

5

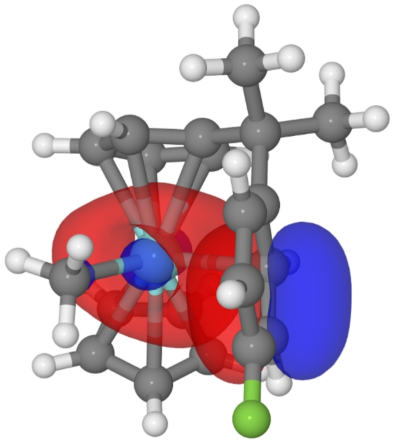

6

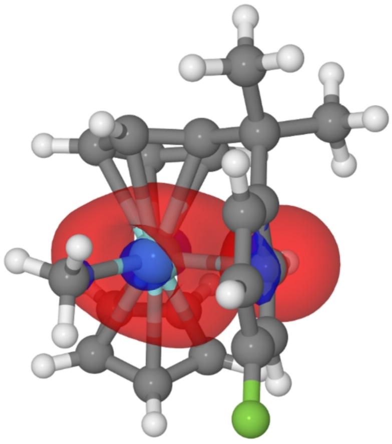

7

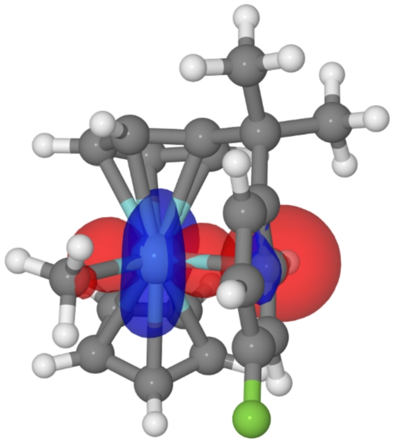

8

	Orbitals	E(2P)
1	$\sigma_{CH} = 0.776(sp^{2.98})_{C7} – 0.631(s)_{H37} \rightarrow$ $LV_{Zr} = sp^{0.14}d^{99.99}$	2.01
2	$\sigma_{CH} = 0.776(sp^{2.98})_{C7} – 0.631(s)_{H37} \rightarrow$ $LV_{Zr} = sp^{0.05}d^{13.57}$	2.92
3	$\sigma_{CC} = 0.712(sp^{1.82})_{C15} – 0.702(sp^{1.73})_{C16} \rightarrow$ $LV_{Zr} = sp^{0.09}d^{84.23}$	2.03
4	$\pi_{CC} = 0.754(sp^{99.99})_{C15} – 0.657(sp^{99.9}d^{4.70})_{C16} \rightarrow$ $LV_{Zr} = sp^{0.14}d^{99.99}$	2.10
5	$\pi_{CC} = 0.754(sp^{99.99})_{C15} – 0.657(sp^{99.9}d^{4.70})_{C16} \rightarrow$ $LV_{Zr} = sp^{0.09}d^{84.23}$	5.38
6	$\pi_{CC} = 0.754(sp^{99.99})_{C15} – 0.657(sp^{99.9}d^{4.70})_{C16} \rightarrow$ $LV_{Zr} = sd^{0.30}$	6.86
7	$\sigma_{CH} = 0.785(sp^{2.69})_{C15} – 0.619(s)_{H26} \rightarrow$ $LV_{Zr} = sd^{0.30}$	5.21
8	$\sigma_{CH} = 0.785(sp^{2.69})_{C15} – 0.619(s)_{H26} \rightarrow$ $LV_{Zr} = sp^{0.05}d^{13.57}$	3.35

Natural Resonance Theory:

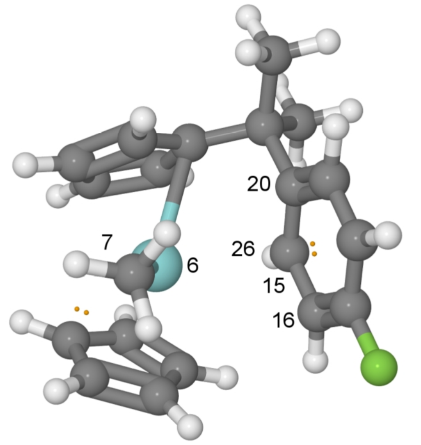

1

Wgt=13.87%;
rhoNL=5.77040;
D(0)=0.10281

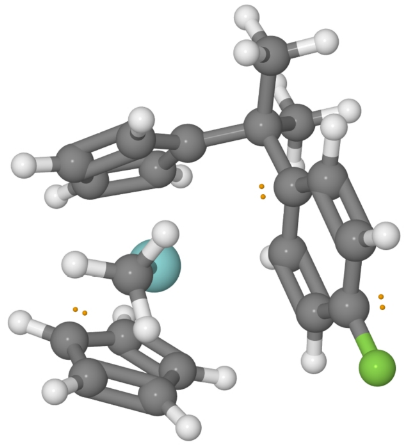

2

Wgt=12.80%;
rhoNL=6.00487;
D(0)=0.10488

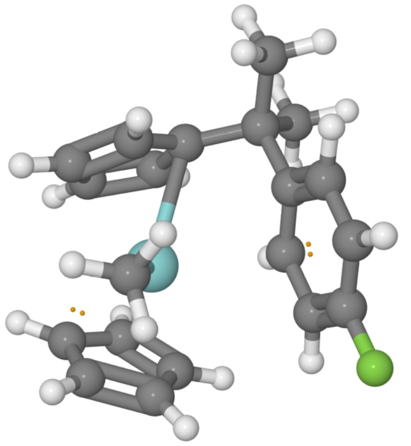

3

Wgt=11.93%;
rhoNL=5.62027;
D(0)=0.10147

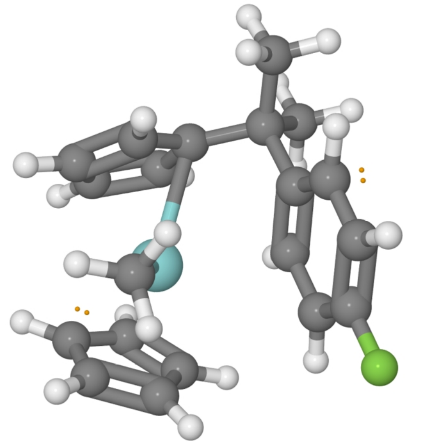

4

Wgt=11.84%;
rhoNL=5.77789;
D(0)=0.10288

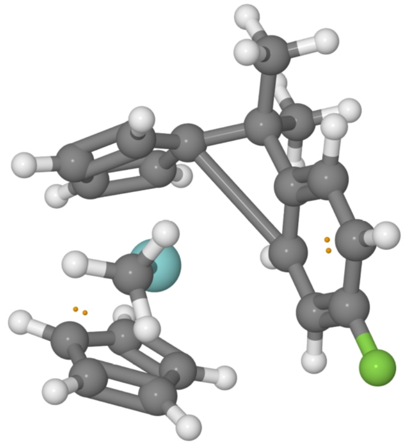

5

Wgt=11.72%;
rhoNL=5.90444;
D(0)=0.10400

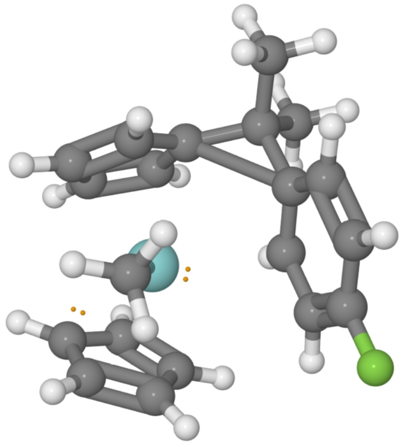

6

Wgt=6.94%;
rhoNL=6.52725;
D(0)=0.10935

Natural Localised Molecular Orbitals (NLMO):

Only contributions over 1% are reported.

NLMO / Occupancy / Percent from Parent NBO / Atomic Hybrid Contributions

Resonance structure **1**:
C-H interaction:
C-C interaction:
Zr-Me interaction:

Resonance structure **1**:
C-H interaction:

```
  83. (2.00000)  97.3689% BD ( 1) C 15- H 26
                    1.427% Zr  6 s( 22.68%)p 0.03(  0.57%)d 3.38( 76.75%)
                   60.167%  C 15 s( 25.07%)p 2.98( 74.82%)d 0.00(  0.11%)
                   37.254%  H 26 s( 99.95%)p 0.00(  0.05%)
```

C-C interaction:

```
 82. (2.00000)  83.5808% BD ( 2) C 15- C 20
                    2.735% Zr  6 s( 28.38%)p 0.03(  0.94%)d 2.49( 70.68%)
                   48.570%  C 15 s(  0.67%)p99.99( 99.31%)d 0.04(  0.03%)
                    4.452%  C 16 s(  0.00%)p 1.00( 99.63%)d 0.00(  0.37%)
                    1.707%  C 17 s(  0.12%)p99.99( 99.71%)d 1.34(  0.17%)
                    1.152%  C 18 s(  0.07%)p99.99( 99.56%)d 5.62(  0.38%)
                    5.340%  C 19 s(  0.00%)p 1.00( 99.68%)d 0.00(  0.32%)
                   35.029%  C 20 s(  0.09%)p99.99( 99.86%)d 0.57(  0.05%)
```

Zr-Me interaction:

```
  57. (2.00000)  97.8283% BD ( 1)Zr  6- C  7
                   20.129% Zr  6 s( 12.71%)p 0.01(  0.08%)d 6.86( 87.22%)
                   77.709%  C  7 s( 25.67%)p 2.89( 74.31%)d 0.00(  0.02%)
```

Resonance structure **2**:
C-H interaction:

```
  83. (2.00000)  97.3674% BD ( 1) C 15- H 26
                    1.405% Zr  6 s( 22.08%)p 0.03(  0.58%)d 3.50( 77.34%)
                   60.156%  C 15 s( 25.06%)p 2.99( 74.83%)d 0.00(  0.11%)
                   37.264%  H 26 s( 99.95%)p 0.00(  0.05%)
```

C-C interaction:

```
  81. (2.00000)  84.6965% BD ( 2) C 15- C 16
                    2.873% Zr  6 s( 27.11%)p 0.03(  0.87%)d 2.66( 72.02%)
                   50.105%  C 15 s(  0.69%)p99.99( 99.29%)d 0.03(  0.02%)
                   34.660%  C 16 s(  0.01%)p 1.00( 99.94%)d 0.00(  0.05%)
                    4.202%  C 17 s(  0.01%)p99.99( 99.52%)d37.64(  0.47%)
                    1.170%  C 18 s(  0.09%)p99.99( 99.69%)d 2.52(  0.22%)
                    1.311%  C 19 s(  0.12%)p99.99( 99.65%)d 1.99(  0.23%)
                    5.215%  C 20 s(  0.10%)p99.99( 99.58%)d 3.16(  0.32%)
```

Zr-Me interaction:
```
  58. (2.00000)  97.8278% BD ( 1)Zr  6- C  7
                 20.157% Zr  6 s( 12.75%)p 0.01(  0.07%)d 6.84( 87.18%)
                 77.681%  C  7 s( 25.68%)p 2.89( 74.30%)d 0.00(  0.02%)
```

Resonance structure **3**:
C-H interaction:
```
  82. (2.00000)  97.3983% BD ( 1) C 15- H 26
                  1.471% Zr  6 s( 23.05%)p 0.02(  0.56%)d 3.31( 76.40%)
                 60.227%  C 15 s( 25.16%)p 2.97( 74.74%)d 0.00(  0.11%)
                 37.206%  H 26 s( 99.95%)p 0.00(  0.05%)
```

Zr-Me interaction:
```
  57. (2.00000)  97.8286% BD ( 1)Zr  6- C  7
                 20.127% Zr  6 s( 12.71%)p 0.01(  0.08%)d 6.86( 87.21%)
                 77.712%  C  7 s( 25.67%)p 2.89( 74.31%)d 0.00(  0.02%)
```

Resonance structure **4**:
C-H interaction:
```
  83. (2.00000)  97.3685% BD ( 1) C 15- H 26
                  1.427% Zr  6 s( 22.68%)p 0.03(  0.57%)d 3.38( 76.75%)
                 60.169%  C 15 s( 25.08%)p 2.98( 74.82%)d 0.00(  0.11%)
                 37.252%  H 26 s( 99.95%)p 0.00(  0.05%)
```

C-C interaction:
```
  82. (2.00000)  83.4838% BD ( 2) C 15- C 20
                  2.780% Zr  6 s( 28.61%)p 0.03(  0.94%)d 2.46( 70.45%)
                 49.987%  C 15 s(  0.66%)p99.99( 99.32%)d 0.04(  0.02%)
                  5.821%  C 16 s(  0.01%)p 1.00( 99.71%)d 0.00(  0.28%)
                  1.083%  C 17 s(  0.18%)p99.99( 99.49%)d 1.86(  0.33%)
                  4.804%  C 19 s(  0.00%)p 1.00( 99.66%)d 0.00(  0.34%)
                 33.543%  C 20 s(  0.09%)p99.99( 99.86%)d 0.59(  0.05%)
```

Zr-Me interaction:
```
  57. (2.00000)  97.8285% BD ( 1)Zr  6- C  7
                  0.058%  C  1 s(  4.00%)p23.27( 93.13%)d 0.72(  2.87%)
                  0.243%  C  2 s(  6.17%)p15.05( 92.78%)d 0.17(  1.05%)
                  0.262%  C  3 s(  6.30%)p14.85( 93.58%)d 0.02(  0.12%)
                  0.207%  C  4 s( 21.58%)p 3.60( 77.69%)d 0.03(  0.73%)
                  0.045%  C  5 s(  9.50%)p 9.27( 88.02%)d 0.26(  2.48%)
                 20.130% Zr  6 s( 12.71%)p 0.01(  0.08%)d 6.86( 87.22%)
                 77.708%  C  7 s( 25.67%)p 2.89( 74.31%)d 0.00(  0.02%)
```

Resonance structure **5**:

C-H interaction:

```
  82. (2.00000)  97.3980% BD ( 1) C 15- H 26
                  1.463% Zr  6 s( 22.51%)p 0.02(  0.56%)d 3.42( 76.94%)
                 60.233%  C 15 s( 25.17%)p 2.97( 74.72%)d 0.00(  0.11%)
                 37.199%  H 26 s( 99.95%)p 0.00(  0.05%)
```

Zr-Me interaction:

```
  57. (2.00000)  97.8239% BD ( 1)Zr  6- C  7
                 20.173% Zr  6 s( 12.72%)p 0.01(  0.07%)d 6.86( 87.21%)
                 77.661%  C  7 s( 25.67%)p 2.90( 74.31%)d 0.00(  0.02%)
```

Resonance structure **6**:

NLMO algorithm failed to converge

Non-Covalent Interactions (NCI)

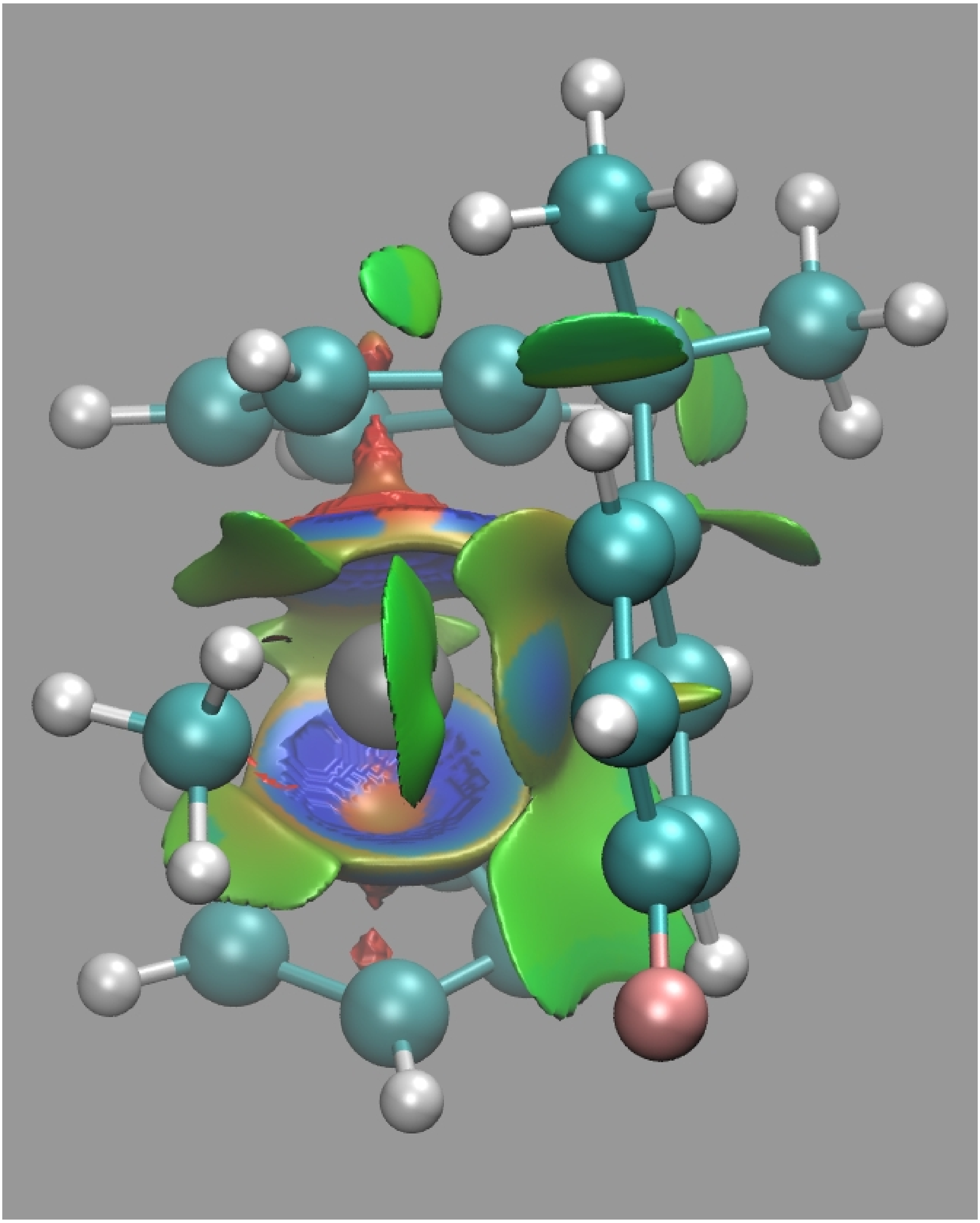

6A-B3LYP

Bader:

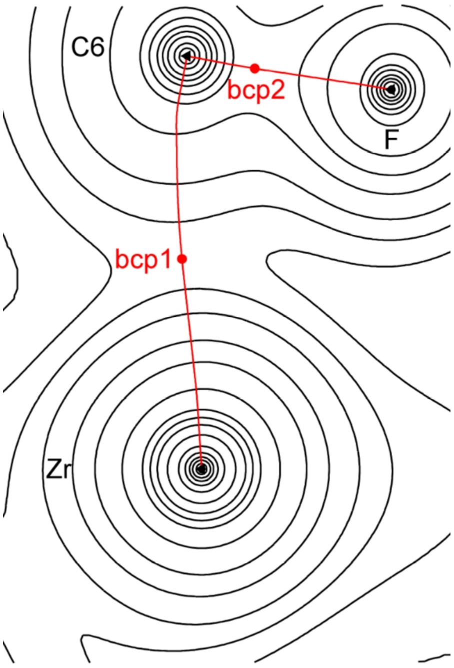

Electrondensity

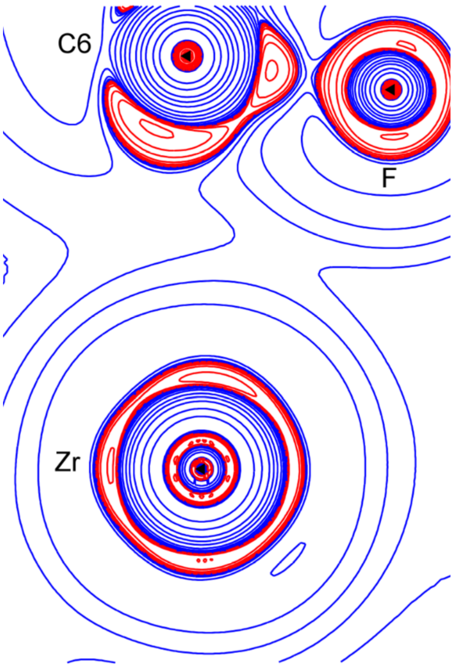

Laplacian

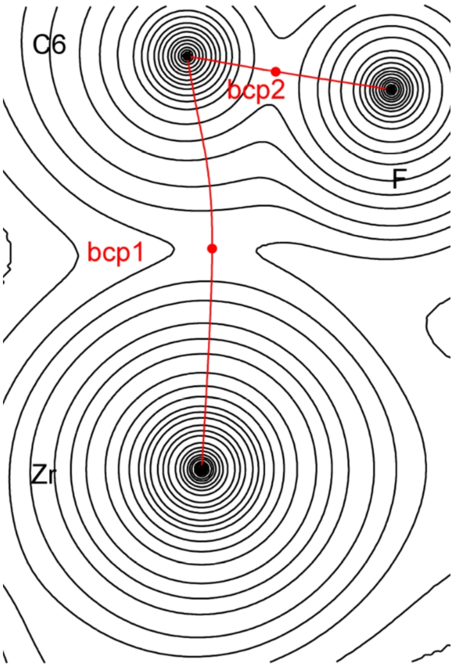

Virial

	ϱ(**r**)	∇^2ϱ(**r**)
bcp1	0.02802	-0.02087
bcp2	0.25684	-0.06651
bcp3	0.09520	-0.01094

NBO:

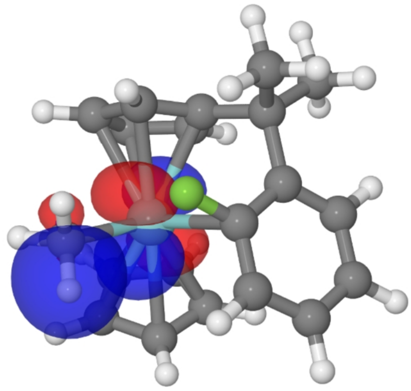

1

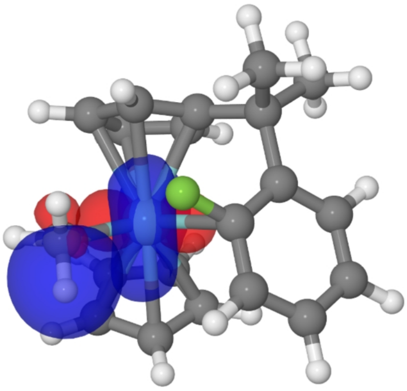

2

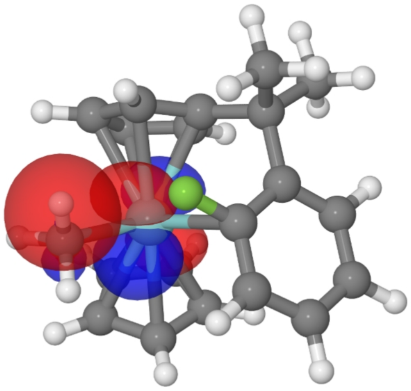

3

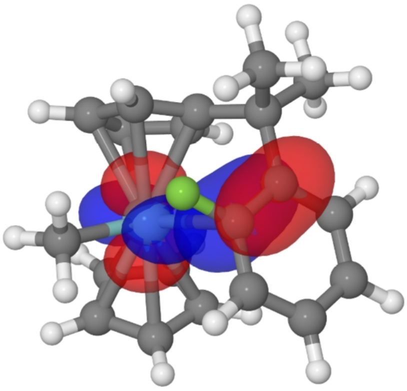

4

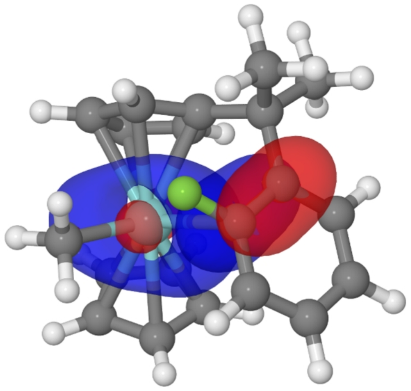

5

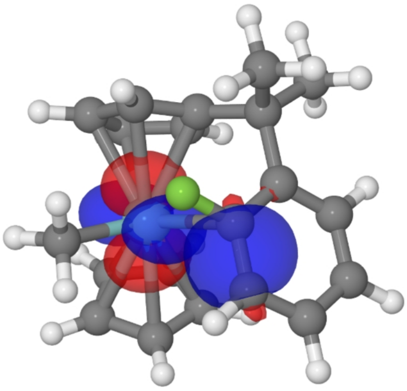

6

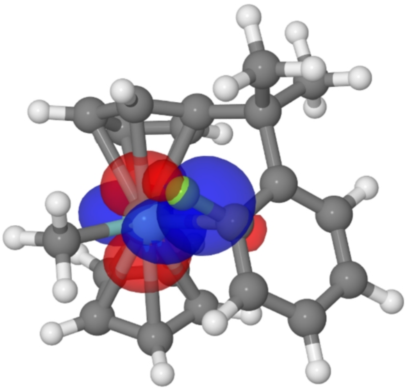

7

	Orbitals	E(2P)
1	$\sigma_{CH} = 0.775(sp^{2.95})_{C12} – 0.632(s)_{H14} \rightarrow$ $LV_{Zr} = sp^{1.17}d^{99.99}$	2.29
2	$\sigma_{CH} = 0.775(sp^{2.95})_{C12} – 0.632(s)_{H14} \rightarrow$ $LV_{Zr} = sd^{4.06}$	2.66
3	$\sigma_{CH} = 0.777(sp^{2.88})_{C12} – 0.629(s)_{H15} \rightarrow$ $LV_{Zr} = sp^{1.17}d^{99.99}$	1.90
4	$\pi_{CC} = 0.671(sp^{99.99}d^{0.78})_{C30} – 0.741(sp^{99.99})_{C31} \rightarrow$ $LV_{Zr} = sp^{0.17}d^{99.99}$	5.03
5	$\pi_{CC} = 0.671(sp^{99.99}d^{0.78})_{C30} – 0.741(sp^{99.99})_{C31} \rightarrow$ $LV_{Zr} = sd^{0.54}$	8.61
6	$\sigma_{CC} = 0.712(sp^{1.60})_{C31} – 0.702(sp^{1.88})_{C32} \rightarrow$ $LV_{Zr} = sp^{0.17}d^{99.99}$	3.17
7	$\sigma_{CF} = 0.520(sp^{3.71})_{C31} – 0.854(sp^{2.09})_{F} \rightarrow$ $LV_{Zr} = sp^{0.17}d^{99.99}$	2.46

Natural Resonance Theory:

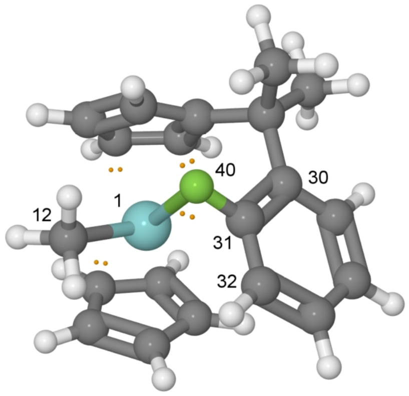

1

Wgt=15.41%;
rhoNL=5.20546;
D(0)=0.09765

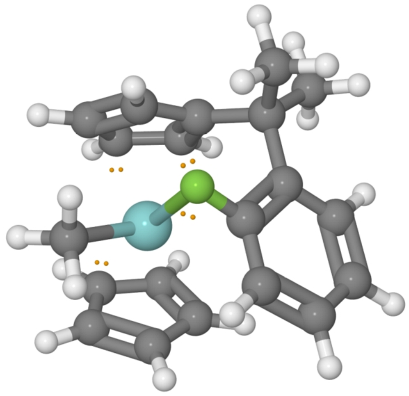

2

Wgt=11.82%;
rhoNL=5.20186;
D(0)=0.09762

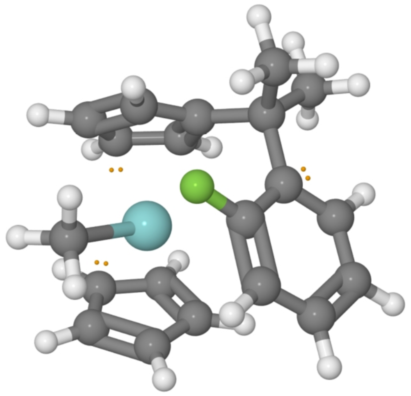

3

Wgt=10.98%;
rhoNL=5.87939;
D(0)=0.10378

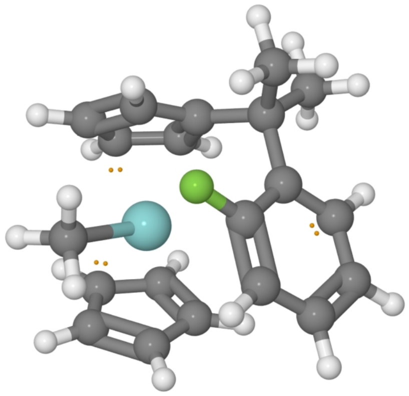

4

Wgt=8.40%;
rhoNL=5.91030;
D(0)=0.10405

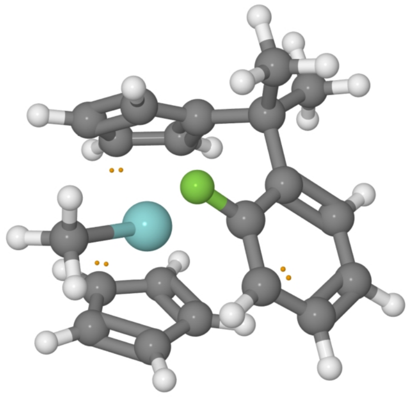

5

Wgt=7.38%;
rhoNL=5.93725;
D(0)=0.10429

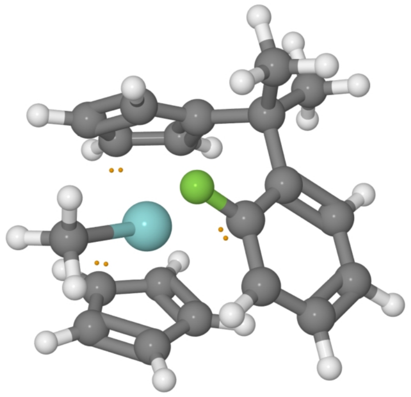

6

Wgt=7.06%;
rhoNL=5.80480;
D(0)=0.10312

Natural Localised Molecular Orbitals (NLMO):

Only contributions over 1% are reported.

NLMO / Occupancy / Percent from Parent NBO / Atomic Hybrid Contributions

Resonance structure **1**:
Zr-F interaction:

```
 45. (2.00000)  99.3303% BD ( 1)Zr  1- F 40
                       0.576% Zr  1 s( 20.87%)p 0.01(  0.21%)d 3.78( 78.92%)
                       0.396%  C 31 s( 25.33%)p 2.91( 73.80%)d 0.03(  0.87%)
                      98.764%  F 40 s( 65.25%)p 0.53( 34.75%)d 0.00(  0.01%)
```

C-C interaction:
80. (2.00000) 83.1369% BD (2) C 30- C 31
3.032% Zr 1 s(21.63%)p 0.05(1.19%)d 3.57(77.18%)
37.775% C 30 s(0.09%)p99.99(99.85%)d 0.80(0.07%)
45.379% C 31 s(0.74%)p99.99(99.22%)d 0.06(0.05%)
4.865% C 32 s(0.04%)p99.99(99.58%)d10.62(0.39%)
1.598% C 33 s(0.14%)p99.99(99.66%)d 1.48(0.20%)
1.311% C 34 s(0.08%)p99.99(99.62%)d 3.49(0.29%)
4.967% C 35 s(0.02%)p99.99(99.66%)d20.98(0.32%)

Zr-Me interaction:
44. (2.00000) 97.6164% BD (1)Zr 1- C 12
20.022% Zr 1 s(12.07%)p 0.01(0.09%)d 7.28(87.85%)
77.606% C 12 s(25.94%)p 2.85(74.04%)d 0.00(0.02%)

Resonance structure **2**:

Zr-F interaction:
45. (2.00000) 99.3303% BD (1)Zr 1- F 40
0.576% Zr 1 s(20.87%)p 0.01(0.21%)d 3.78(78.92%)
0.396% C 31 s(25.33%)p 2.91(73.80%)d 0.03(0.87%)
98.764% F 40 s(65.25%)p 0.53(34.75%)d 0.00(0.01%)

C-C interaction:
80. (2.00000) 83.1369% BD (2) C 30- C 31
3.032% Zr 1 s(21.63%)p 0.05(1.19%)d 3.57(77.18%)
37.775% C 30 s(0.09%)p99.99(99.85%)d 0.80(0.07%)
45.379% C 31 s(0.74%)p99.99(99.22%)d 0.06(0.05%)
4.865% C 32 s(0.04%)p99.99(99.58%)d10.62(0.39%)
1.598% C 33 s(0.14%)p99.99(99.66%)d 1.48(0.20%)
1.311% C 34 s(0.08%)p99.99(99.62%)d 3.49(0.29%)
4.967% C 35 s(0.02%)p99.99(99.66%)d20.98(0.32%)

Zr-Me interaction:
44. (2.00000) 97.6164% BD (1)Zr 1- C 12
20.022% Zr 1 s(12.07%)p 0.01(0.09%)d 7.28(87.85%)
77.606% C 12 s(25.94%)p 2.85(74.04%)d 0.00(0.02%)

Resonance structure **3**:

C-C interaction:
83. (2.00000) 83.1698% BD (2) C 31- C 32
2.166% Zr 1 s(24.17%)p 0.06(1.43%)d 3.08(74.40%)
5.268% C 30 s(0.17%)p99.99(99.42%)d 2.30(0.40%)
45.926% C 31 s(0.70%)p99.99(99.26%)d 0.05(0.03%)
37.264% C 32 s(0.02%)p99.99(99.92%)d 3.04(0.06%)
5.638% C 33 s(0.05%)p99.99(99.64%)d 6.07(0.31%)
1.334% C 34 s(0.08%)p99.99(99.66%)d 3.38(0.26%)
1.896% C 35 s(0.03%)p99.99(99.84%)d 4.57(0.13%)

Zr-Me interaction:
46. (2.00000) 97.6165% BD (1)Zr 1- C 12
20.019% Zr 1 s(12.08%)p 0.01(0.09%)d 7.27(87.83%)
77.609% C 12 s(25.94%)p 2.85(74.04%)d 0.00(0.02%)

Resonance structure **4**, **5**:
NLMO algorithm failed to converge

Resonance structure **6**:

Zr-Me interaction:
46. (2.00000) 97.6164% BD (1)Zr 1- C 12
20.019% Zr 1 s(12.08%)p 0.01(0.09%)d 7.27(87.83%)
77.610% C 12 s(25.94%)p 2.85(74.04%)d 0.00(0.02%)

Non-Covalent Interactions (NCI)

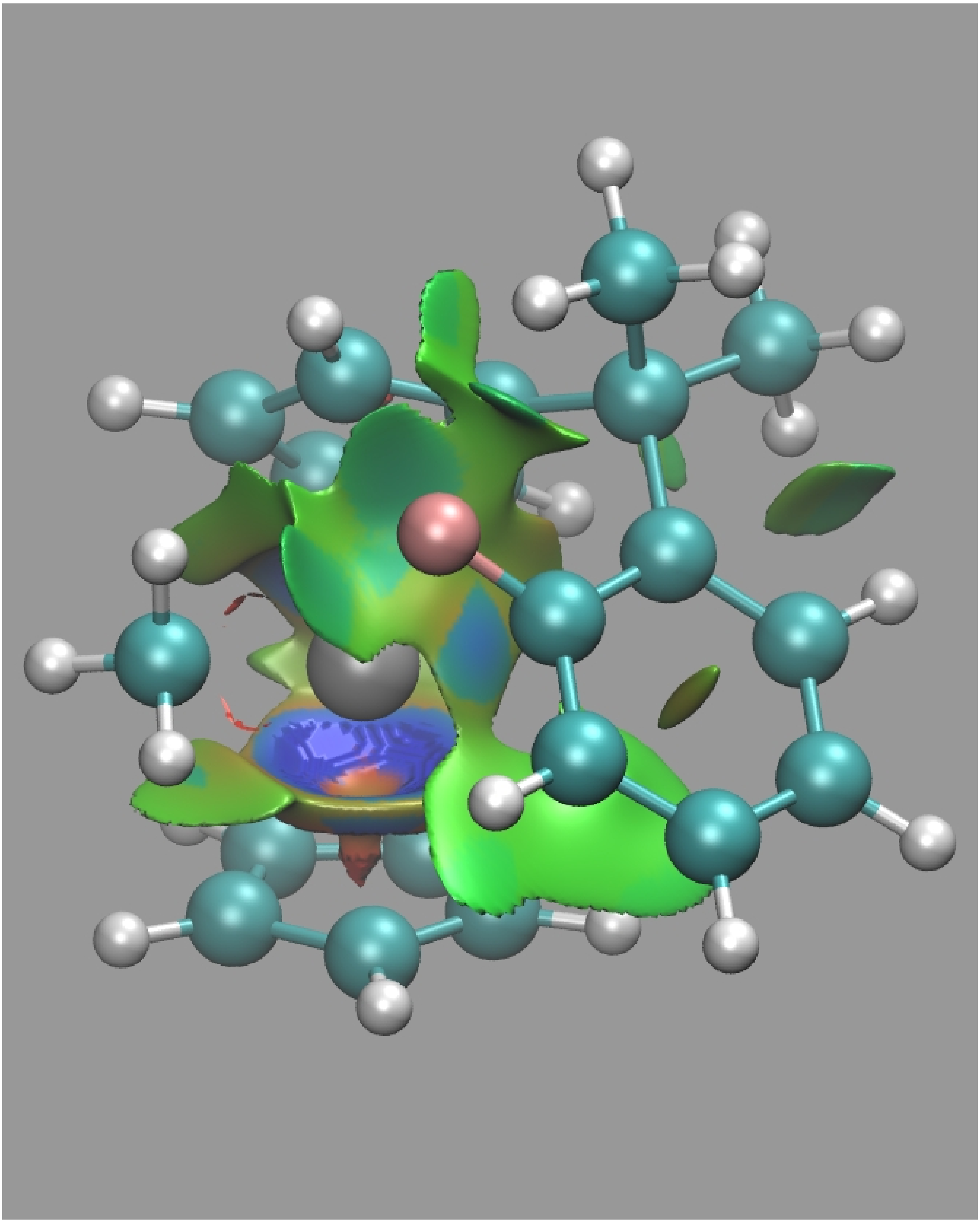

6B-B3LYP

Bader:

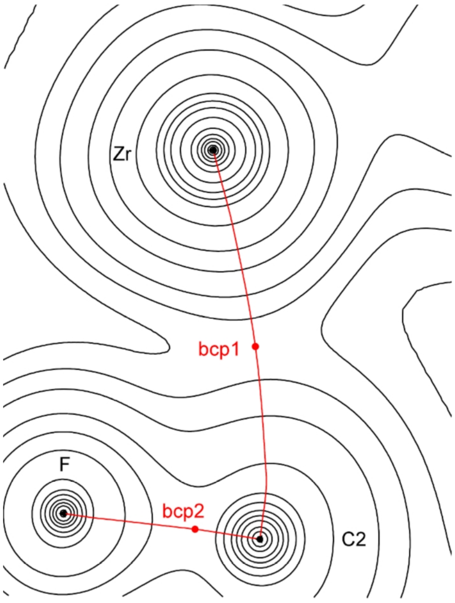

Electrondensity

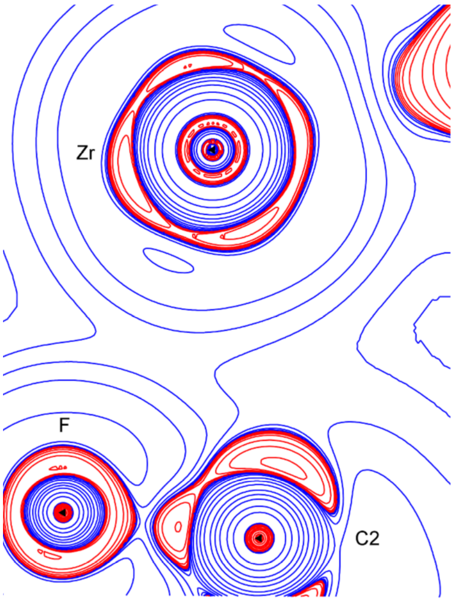

Laplacian

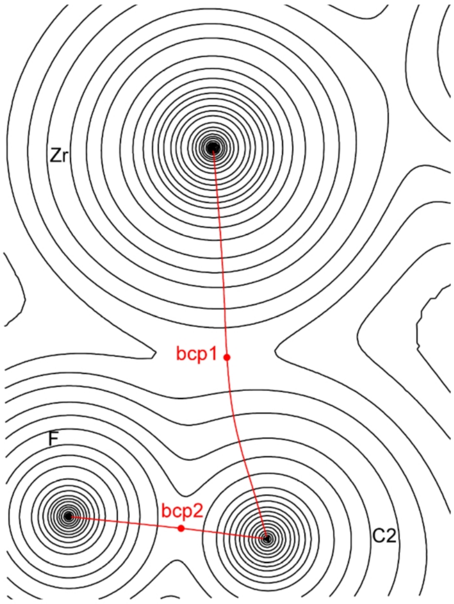

Virial

	ϱ(**r**)	∇^2ϱ(**r**)
bcp1	0.02866	-0.02159
bcp2	0.25328	-0.08674
bcp3	0.09622	-0.01157

NBO:

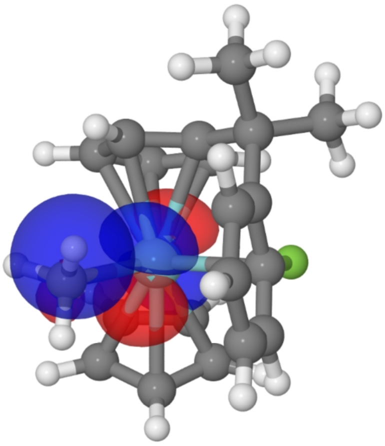

1

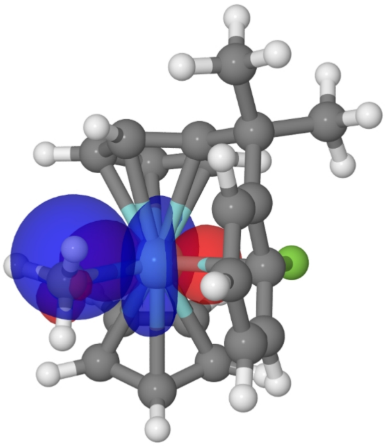

2

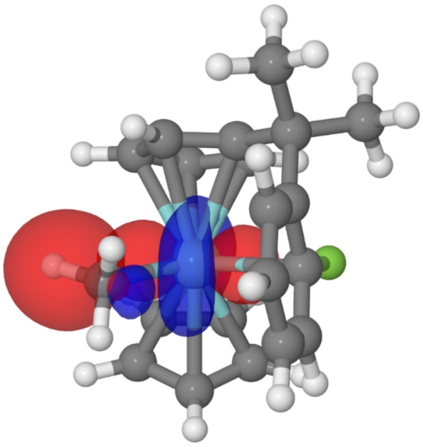

3

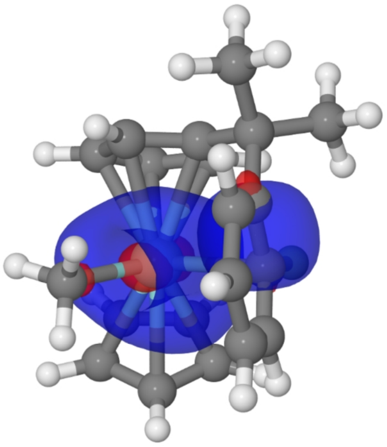

4

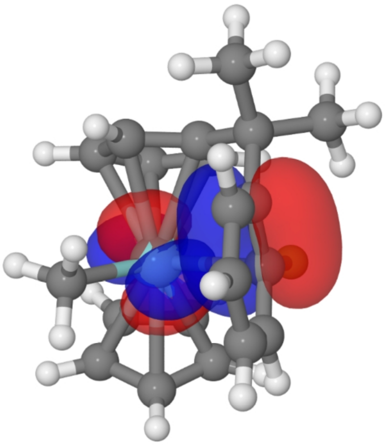

5

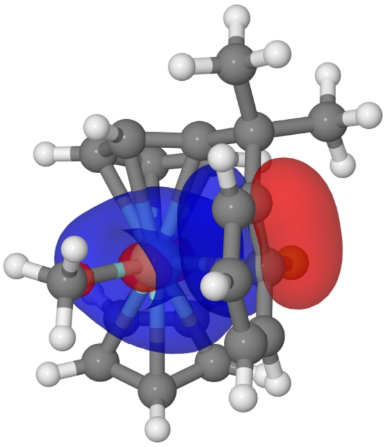

6

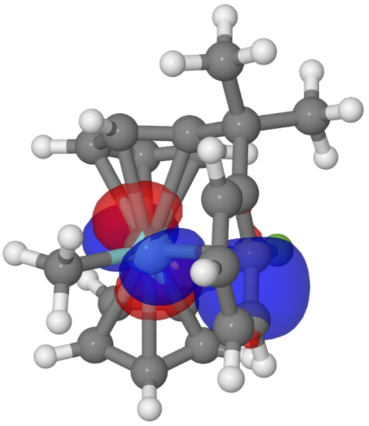

7

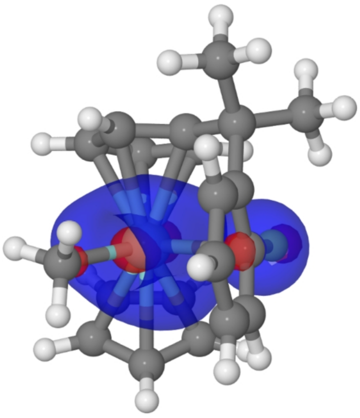

8

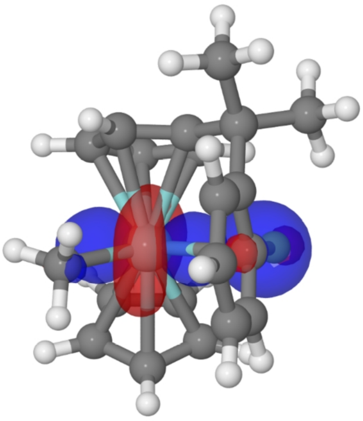

9

	Orbitals	E(2P)
1	σ_{CH} = 0.776$(sp^{3.00})_{C12}$ – 0.631$(s)_{H13}$ → $LV_{Zr} = p^{2.10}d^{99.99}$	2.23
2	σ_{CH} = 0.776$(sp^{3.00})_{C12}$ – 0.631$(s)_{H13}$ → $LV_{Zr} = sp^{0.12}d^{59.00}$	2.13
3	σ_{CH} = 0.781$(sp^{3.08})_{C12}$ – 0.625$(s)_{H14}$ → $LV_{Zr} = sp^{0.12}d^{59.00}$	2.14
4	σ_{CC} = 0.705$(sp^{2.14})_{C28}$ - 0.709$(sp^{1.50})_{C29}$ → $LV_{Zr} = sd^{0.19}$	2.63
5	π_{CC} = 0.657$(sp^{99.99}d^{0.45})_{C28}$ – 0.754$(sp^{99.99})_{C29}$ → $LV_{Zr} = sp^{0.13}d^{99.99}$	5.29
6	π_{CC} = 0.657$(sp^{99.99}d^{0.45})_{C28}$ – 0.754$(sp^{99.99})_{C29}$ → $LV_{Zr} = sd^{0.19}$	5.07
7	σ_{CC} = 0.712$(sp^{1.59})_{C29}$ – 0.702$(sp^{1.88})_{C30}$ → $LV_{Zr} = sp^{0.13}d^{99.99}$	2.69
8	σ_{CF} = 0.515$(sp^{3.82})_{C29}$ – 0.857$(sp^{2})_{F}$ → $LV_{Zr} = sd^{0.19}$	2.12
9	σ_{CF} = 0.515$(sp^{3.82})_{C29}$ – 0.857$(sp^{2})_{F}$ → $LV_{Zr} = sp^{0.12}d^{59.00}$	3.07

Natural Resonance Theory:

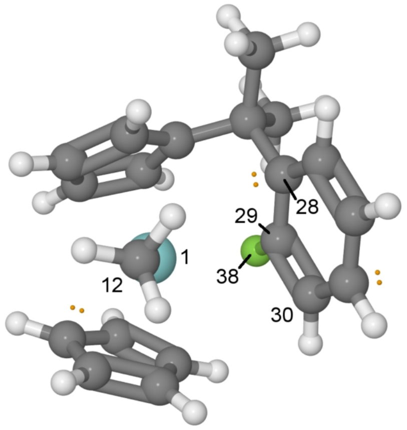

1

Wgt=15.49%;
rhoNL=6.00847;
D(0)=0.10491

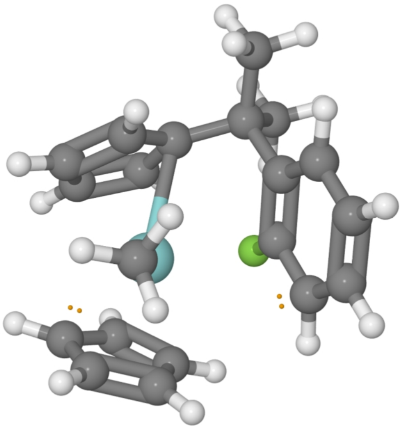

2

Wgt=14.54%;
rhoNL=5.73814;
D(0)=0.10252

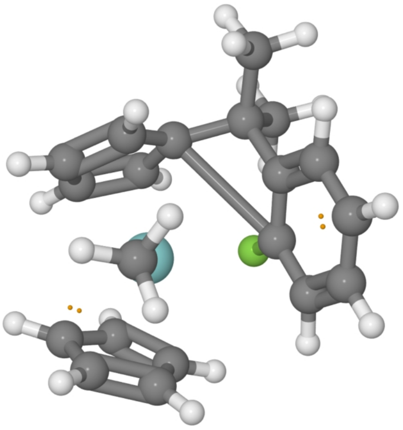

3

Wgt=12.85%;
rhoNL=5.88839;
D(0)=0.10386

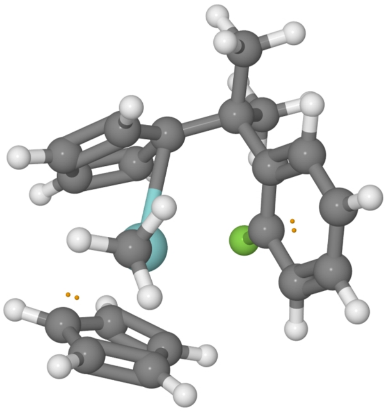

4

Wgt=12.08%;
rhoNL=5.57270;
D(0)=0.10104

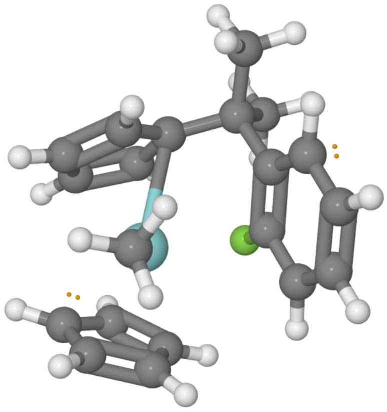

5

Wgt=11.98%;
rhoNL=5.75025;
D(0)=0.10263

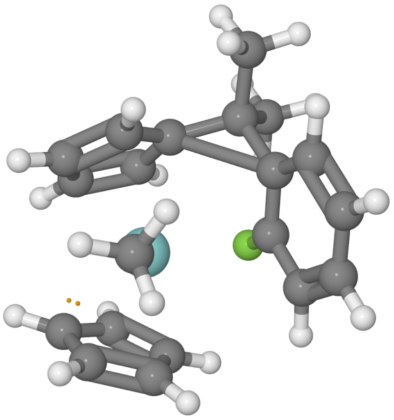

6

Wgt=8.08%;
rhoNL=6.48213;
D(0)=0.10897

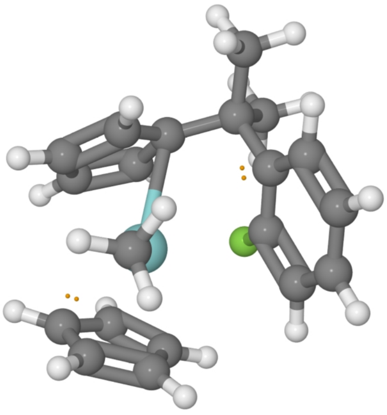

7

Wgt=6.29%;
rhoNL=5.72006;
D(0)=0.10236

Natural Localised Molecular Orbitals (NLMO):

Only contributions over 1% are reported.

NLMO / Occupancy / Percent from Parent NBO / Atomic Hybrid Contributions

Resonance structure **1**:
C-C interaction:

```
 83. (2.00000)  83.3827% BD ( 2) C 29- C 30
                  2.387% Zr  1 s( 20.38%)p 0.04(  0.80%)d 3.87( 78.83%)
                  7.843%  C 28 s(  0.19%)p99.99( 99.55%)d 1.36(  0.26%)
                 50.082%  C 29 s(  0.98%)p99.99( 99.00%)d 0.02(  0.02%)
                 33.440%  C 30 s(  0.02%)p99.99( 99.91%)d 3.48(  0.07%)
                  3.596%  C 31 s(  0.06%)p99.99( 99.53%)d 7.50(  0.41%)
                  1.243%  C 32 s(  0.11%)p99.99( 99.71%)d 1.68(  0.18%)
```

Zr-Me interaction:

```
 46. (2.00000)  97.8363% BD ( 1)Zr  1- C 12
                 19.503% Zr  1 s( 11.84%)p 0.01(  0.08%)d 7.44( 88.08%)
                 78.341%  C 12 s( 25.66%)p 2.90( 74.32%)d 0.00(  0.02%)
```

Resonance structure **2**:
C-C interaction:

```
 81. (2.00000)  83.2061% BD ( 2) C 28- C 29
                  2.268% Zr  1 s( 19.59%)p 0.04(  0.70%)d 4.07( 79.71%)
                 36.587%  C 28 s(  0.14%)p99.99( 99.80%)d 0.46(  0.06%)
                 46.647%  C 29 s(  0.97%)p99.99( 98.99%)d 0.03(  0.03%)
                  5.375%  C 30 s(  0.03%)p99.99( 99.61%)d12.26(  0.36%)
                  1.253%  C 31 s(  0.10%)p99.99( 99.65%)d 2.41(  0.25%)
```

Zr-Me interaction:

```
 45. (2.00000)  97.8390% BD ( 1)Zr  1- C 12
                 19.500% Zr  1 s( 11.86%)p 0.01(  0.08%)d 7.42( 88.06%)
                 78.346%  C 12 s( 25.66%)p 2.90( 74.32%)d 0.00(  0.02%)
```

Resonance structure **3**:
Zr-Me interaction:

```
 45. (2.00000)  97.8348% BD ( 1)Zr  1- C 12
                 19.498% Zr  1 s( 11.81%)p 0.01(  0.08%)d 7.46( 88.11%)
                 78.344%  C 12 s( 25.66%)p 2.90( 74.32%)d 0.00(  0.02%)
```

Resonance structure **4**:
Zr-Me interaction:

```
 45. (2.00000)  97.8398% BD ( 1)Zr  1- C 12
                 19.495% Zr  1 s( 11.88%)p 0.01(  0.08%)d 7.41( 88.04%)
                 78.352%  C 12 s( 25.66%)p 2.90( 74.32%)d 0.00(  0.02%)
```

Resonance structure **5**:
C-C interaction:

```
 81. (2.00000)  83.1839% BD ( 2) C 28- C 29
                  2.336% Zr  1 s( 19.73%)p 0.04(  0.71%)d 4.03( 79.55%)
                 35.200%  C 28 s(  0.14%)p99.99( 99.80%)d 0.47(  0.07%)
```

48.015% C 29 s(0.98%)p99.99(98.99%)d 0.03(0.03%)
6.236% C 30 s(0.03%)p99.99(99.65%)d11.07(0.32%)
1.132% C 31 s(0.11%)p99.99(99.58%)d 2.86(0.31%)
5.006% C 33 s(0.03%)p99.99(99.66%)d11.25(0.31%)

Zr-Me interaction:
45. (2.00000) 97.8389% BD (1)Zr 1- C 12
19.499% Zr 1 s(11.86%)p 0.01(0.08%)d 7.42(88.06%)
78.347% C 12 s(25.66%)p 2.90(74.32%)d 0.00(0.02%)

Resonance structure **6**:
NLMO algorithm failed to converge

Resonance structure **7**:
C-C interaction:
83. (2.00000) 84.0691% BD (2) C 29- C 30
2.213% Zr 1 s(21.80%)p 0.04(0.78%)d 3.55(77.42%)
5.202% C 28 s(0.23%)p99.99(99.42%)d 1.57(0.36%)
46.501% C 29 s(0.97%)p99.99(99.00%)d 0.03(0.03%)
37.599% C 30 s(0.02%)p99.99(99.92%)d 3.00(0.06%)
6.001% C 31 s(0.06%)p99.99(99.67%)d 4.92(0.27%)
1.183% C 33 s(0.08%)p99.99(99.70%)d 2.76(0.22%)

Zr-Me interaction:
45. (2.00000) 97.8387% BD (1)Zr 1- C 12
19.498% Zr 1 s(11.87%)p 0.01(0.08%)d 7.41(88.05%)
78.347% C 12 s(25.66%)p 2.90(74.32%)d 0.00(0.02%)

Non-Covalent Interactions (NCI)

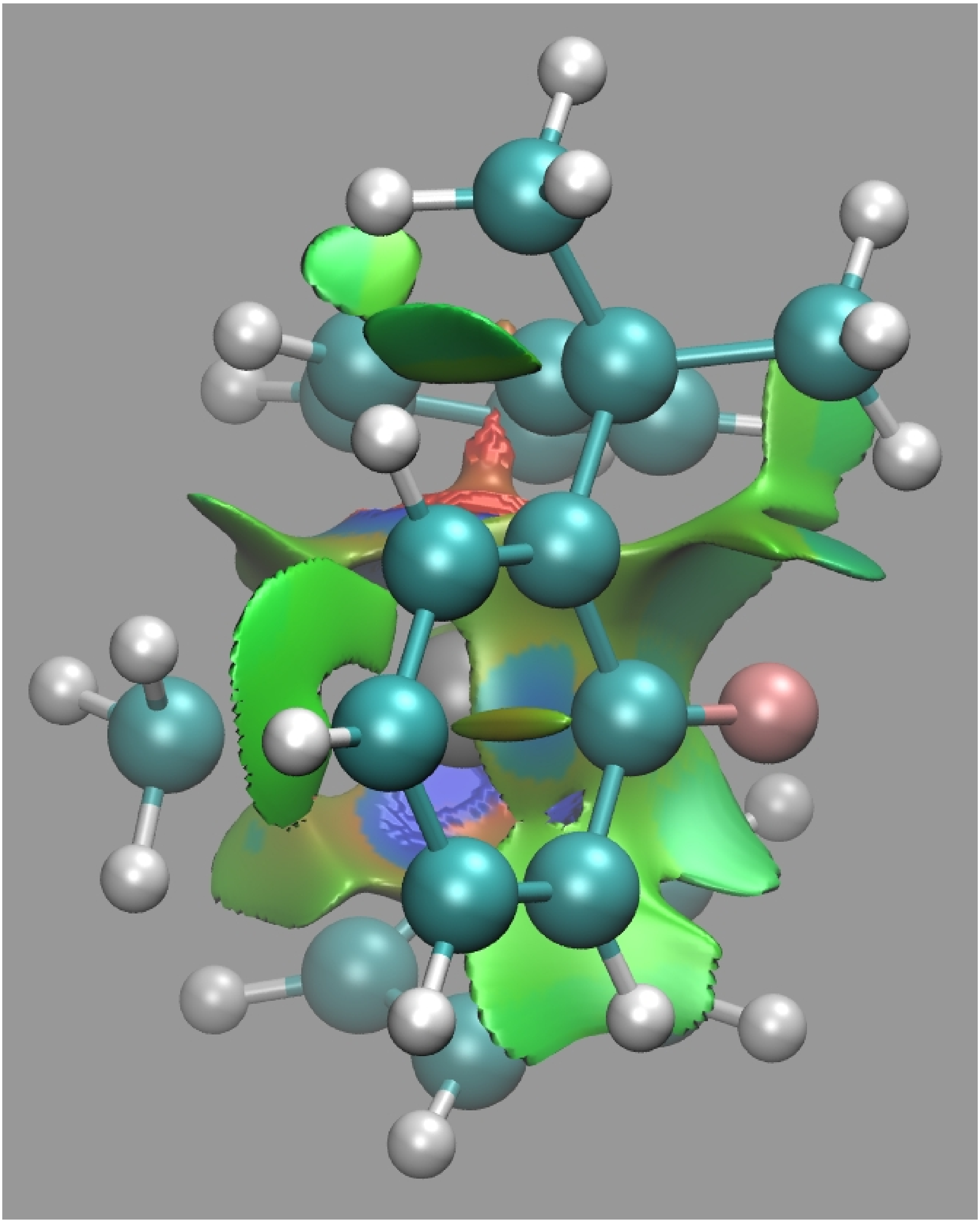

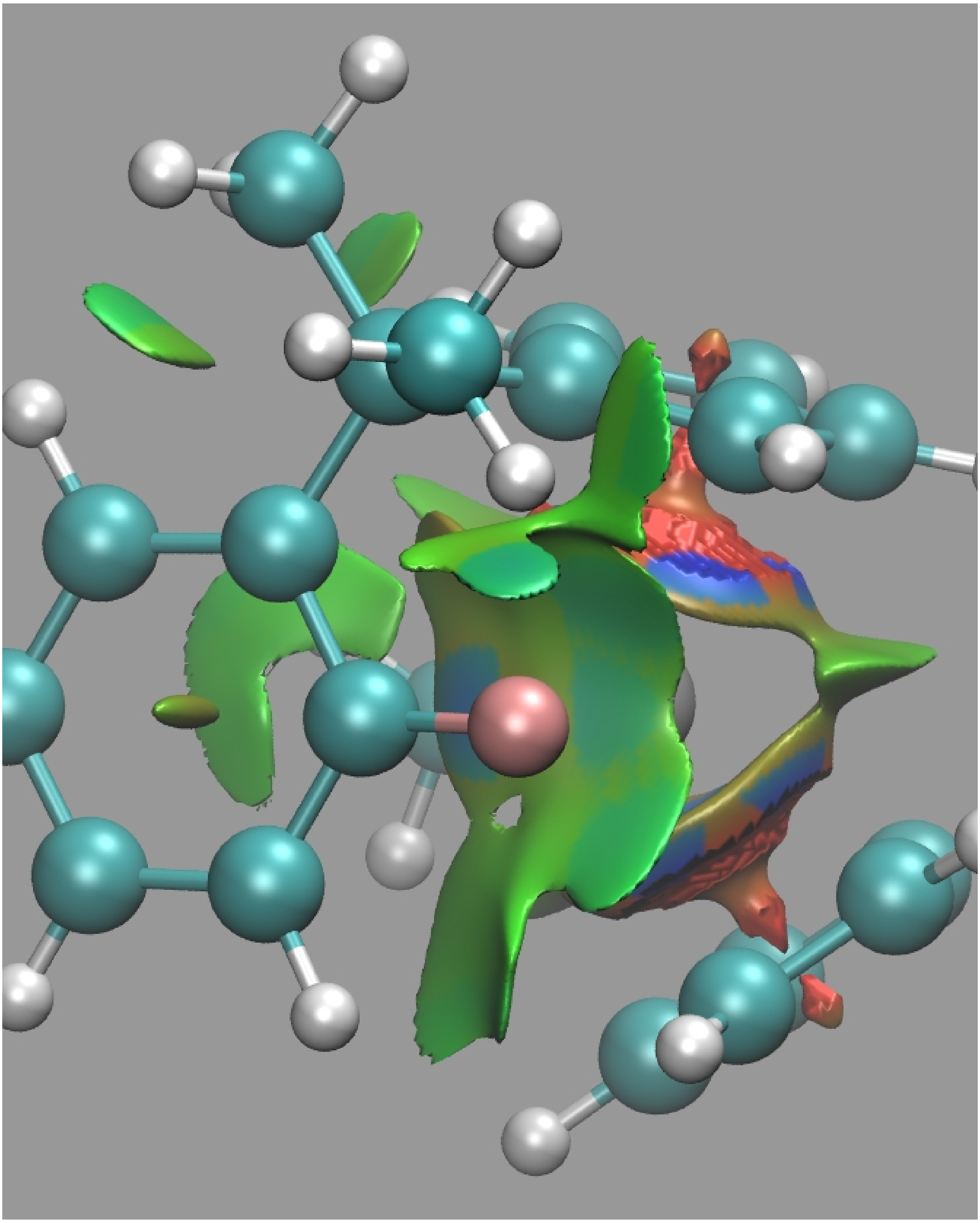

IIA-PBE-D3

Bader:

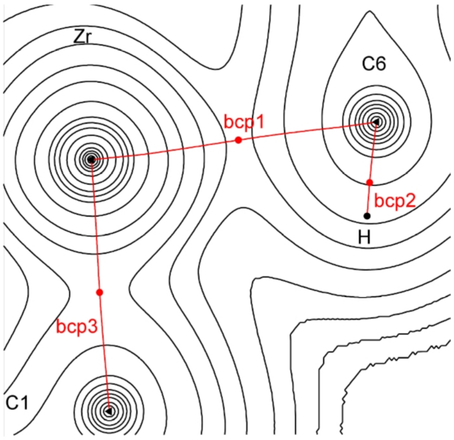

Electrondensity

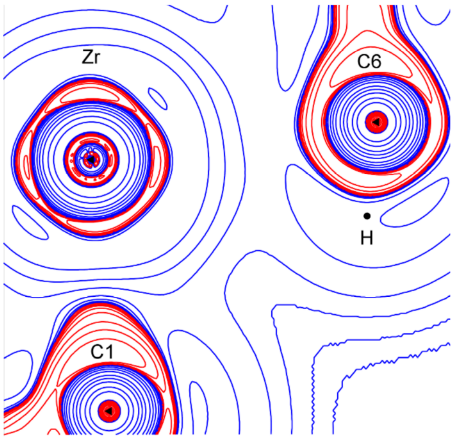

Laplacian

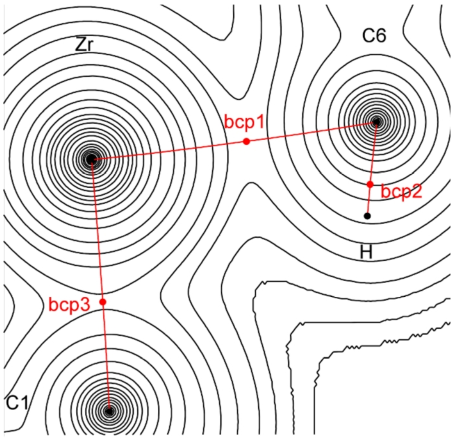

Virial

	ϱ(**r**)	∇²ϱ(**r**)
bcp1	0.03589	-0.02196
bcp2	0.26740	0.21461
bcp3	0.09443	-0.01249

NBO:

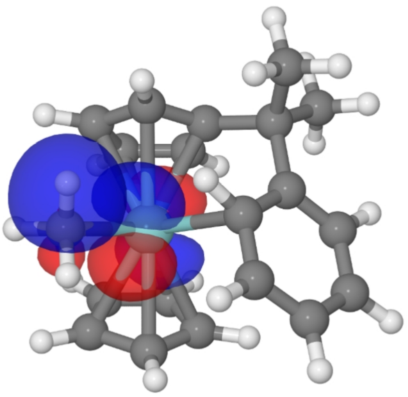

1

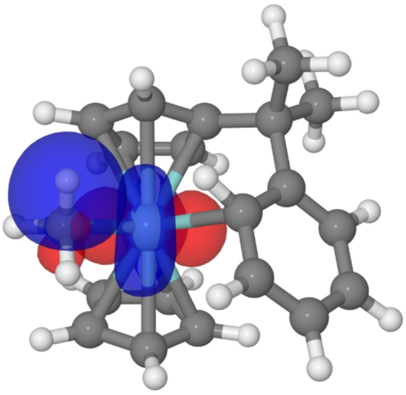

2

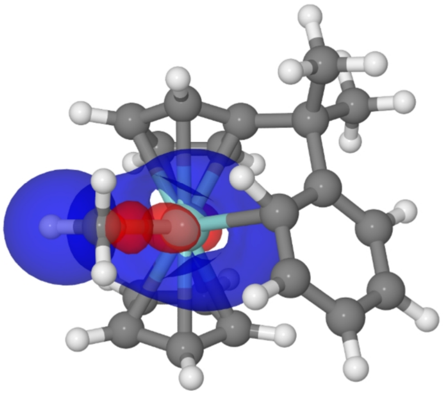

3

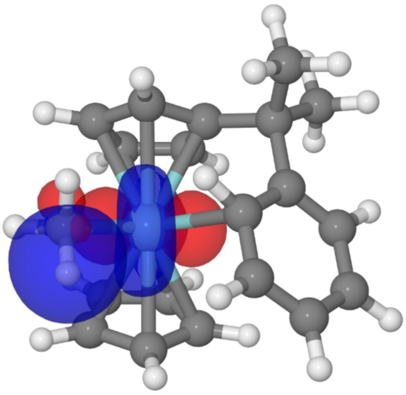

4

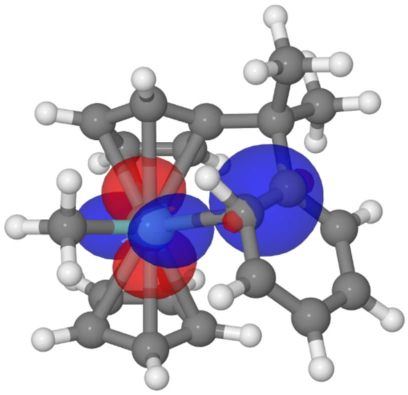

5

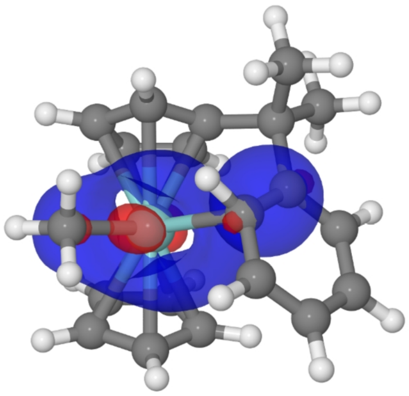

6

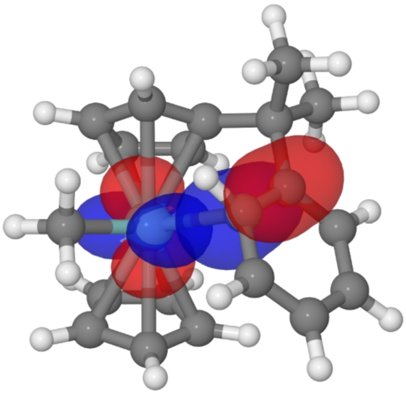

7

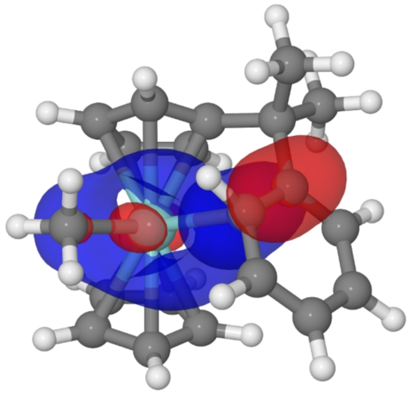

8

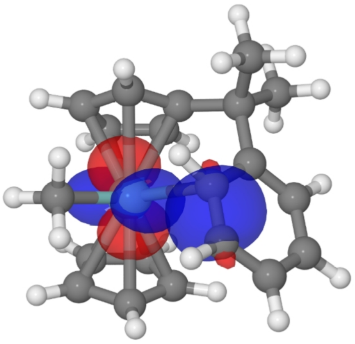

9

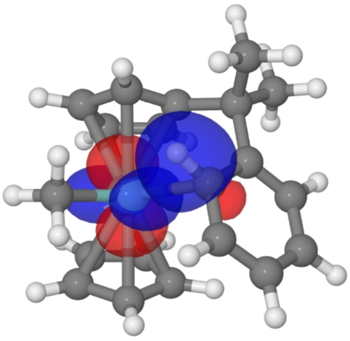

10

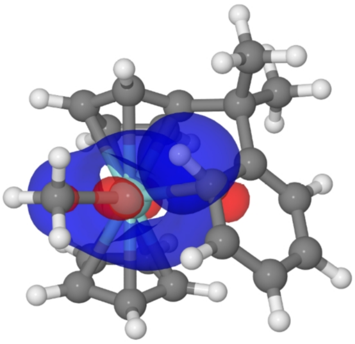

11

	Orbitals	E(2P)
1	σ_{CH} = 0.780$(sp^{3})_{C7}$ 0.625$(s)_{H42}$ → LV_{Zr} = $p^{0.70}d^{99.99}$	2.23
2	σ_{CH} = 0.780$(sp^{3})_{C7}$ 0.625$(s)_{H42}$ → LV_{Zr} = $sd^{12.22}$	2.43
3	σ_{CH} = 0.787$(sp^{3.23})_{C7}$ 0.617$(s)_{H43}$ → LV_{Zr} = $sd^{0.31}$	2.23
4	σ_{CH} = 0.781$(sp^{3.01})_{C7}$ 0.625$(s)_{H44}$ → LV_{Zr} = $sd^{12.22}$	2.95
5	σ_{CC} = 0.710$(sp^{1.79})_{C8}$ 0.704$(sp^{2.05})_{C9}$ → LV_{Zr} = $sd^{45.97}$	2.81
6	σ_{CC} = 0.710$(sp^{1.79})_{C8}$ 0.704$(sp^{2.05})_{C9}$ → LV_{Zr} = $sd^{0.31}$	3.33
7	σ_{CC} = 0.759$(p)_{C9}$ 0.653$(p)_{C9}$ → LV_{Zr} = $sd^{45.97}$	8.13
8	σ_{CC} = 0.759$(p)_{C9}$ 0.653$(p)_{C9}$ → LV_{Zr} = $sd^{0.31}$	5.25
9	σ_{CC} = 0.715$(sp^{1.80})_{C8}$ 0.699$(sp^{1.79})_{C13}$ → LV_{Zr} = $sd^{45.97}$	3.45
10	σ_{CH} = 0.793$(sp^{2.57})_{C8}$ 0.609$(s)_{H33}$ → LV_{Zr} = $sd^{45.97}$	5.49
11	σ_{CH} = 0.793$(sp^{2.57})_{C8}$ 0.609$(s)_{H33}$ → LV_{Zr} = $sd^{0.31}$	4.47

Natural Resonance Theory:

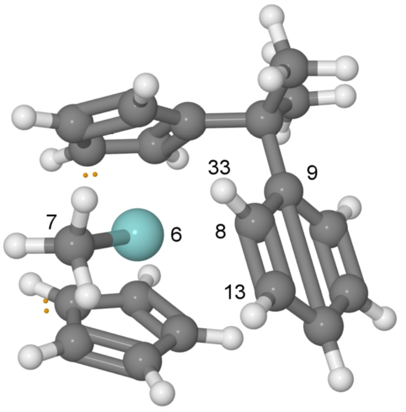

1

Wgt=17.61%;
rhoNL=5.62199;
D(0)=0.10047

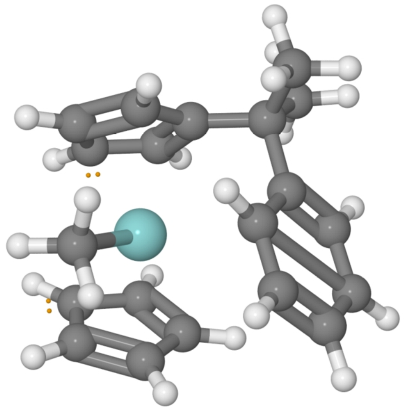

2

Wgt=13.85%;
rhoNL=5.58604;
D(0)=0.10015

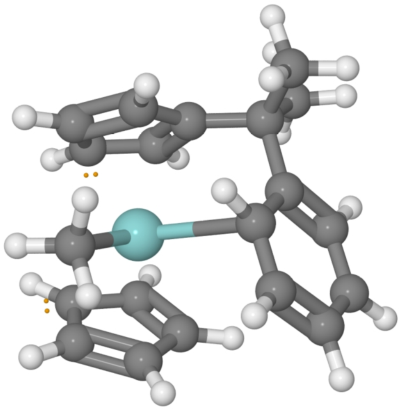

3

Wgt=7.27%;
rhoNL=5.70517; D(0)=0.101

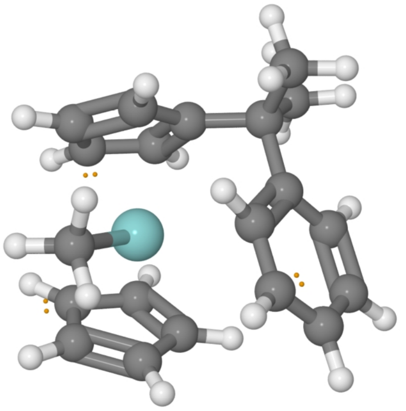

4

Wgt=6.46%;
rhoNL=5.97372;
D(0)=0.10357

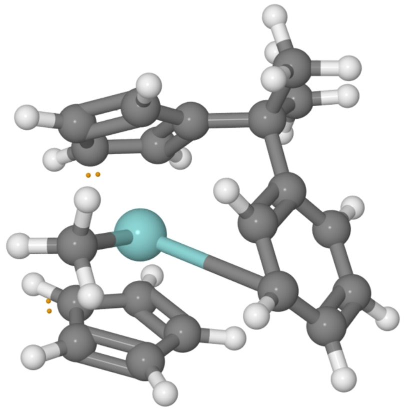

5

Wgt=6.35%;
rhoNL=5.94644;
D(0)=0.10333

Natural Localised Molecular Orbitals (NLMO):

Only contributions over 1% are reported.

NLMO / Occupancy / Percent from Parent NBO / Atomic Hybrid Contributions

Resonance structure **1**:
C-H interaction:
```
 60. (2.00000)  97.2895% BD ( 1) C  8- H 33
                 1.279% Zr  6 s( 29.65%)p 0.02(  0.71%)d 2.35( 69.63%)
                61.334%  C  8 s( 26.37%)p 2.79( 73.55%)d 0.00(  0.08%)
                35.987%  H 33 s( 99.97%)p 0.00(  0.03%)
```

C-C interaction:
```
 58. (2.00000)  98.7560% BD ( 1) C  8- C 13
                50.523%  C  8 s( 32.19%)p 2.11( 67.76%)d 0.00(  0.05%)
                48.355%  C 13 s( 32.78%)p 2.05( 67.17%)d 0.00(  0.04%)
 59. (2.00000)  83.5619% BD ( 2) C  8- C 13
                 2.978% Zr  6 s( 22.63%)p 0.04(  0.82%)d 3.38( 76.55%)
                48.614%  C  8 s(  0.81%)p99.99( 99.17%)d 0.03(  0.02%)
                 4.950%  C  9 s(  0.18%)p99.99( 99.47%)d 1.91(  0.35%)
                 1.723%  C 10 s(  0.04%)p99.99( 99.80%)d 4.30(  0.16%)
                 1.120%  C 11 s(  0.11%)p99.99( 99.54%)d 3.13(  0.35%)
                 4.942%  C 12 s(  0.01%)p99.99( 99.68%)d25.71(  0.31%)
                34.971%  C 13 s(  0.02%)p99.99( 99.94%)d 1.65(  0.04%)
```

Zr-Me interaction:
```
 53. (2.00000)  97.1814% BD ( 1)Zr  6- C  7
                20.161% Zr  6 s( 12.08%)p 0.00(  0.05%)d 7.28( 87.88%)
                77.032%  C  7 s( 26.79%)p 2.73( 73.20%)d 0.00(  0.01%)
```

Resonance structure **2**:
C-H interaction:
```
 60. (2.00000)  97.3021% BD ( 1) C  8- H 33
                 1.321% Zr  6 s( 30.13%)p 0.02(  0.70%)d 2.30( 69.17%)
                61.372%  C  8 s( 26.48%)p 2.77( 73.44%)d 0.00(  0.08%)
                  35.955%  H 33 s( 99.97%)p 0.00(  0.03%)
```

C-C interaction:
```
 58. (2.00000)  67.4330% BD ( 1) C  8- C 11
                 3.448% Zr  6 s( 18.81%)p 0.05(  1.02%)d 4.26( 80.17%)
                43.293%  C  8 s(  0.77%)p99.99( 99.22%)d 0.01(  0.01%)
                 7.250%  C  9 s(  0.14%)p99.99( 99.58%)d 1.95(  0.28%)
                 6.888%  C 10 s(  0.04%)p99.99( 99.79%)d 4.50(  0.17%)
                24.215%  C 11 s(  0.05%)p99.99( 99.94%)d 0.22(  0.01%)
                 6.495%  C 12 s(  0.09%)p99.99( 99.72%)d 2.08(  0.19%)
                 7.452%  C 13 s(  0.02%)p99.99( 99.75%)d11.43(  0.23%)
```

Zr-Me interaction:
```
 53. (2.00000)  97.1819% BD ( 1)Zr  6- C  7
                20.158% Zr  6 s( 12.08%)p 0.00(  0.05%)d 7.27( 87.87%)
                77.035%  C  7 s( 26.79%)p 2.73( 73.20%)d 0.00(  0.01%)
```

Resonance structure **3**:
C-H interaction:
```
 60. (2.00000)  97.3091% BD ( 1) C  8- H 33
                 1.306% Zr  6 s( 30.09%)p 0.02(  0.70%)d 2.30( 69.21%)
                61.362%  C  8 s( 26.44%)p 2.78( 73.47%)d 0.00(  0.08%)
                35.970%  H 33 s( 99.97%)p 0.00(  0.03%)
```

C-C interaction:
```
 58. (2.00000)  98.3244% BD ( 1) C  8- C  9
                49.654%  C  8 s( 32.14%)p 2.11( 67.81%)d 0.00(  0.05%)
                48.822%  C  9 s( 29.40%)p 2.40( 70.56%)d 0.00(  0.04%)
 59. (2.00000)  98.7501% BD ( 1) C  8- C 13
                50.527%  C  8 s( 32.22%)p 2.10( 67.74%)d 0.00(  0.05%)
                48.347%  C 13 s( 32.83%)p 2.04( 67.12%)d 0.00(  0.04%)
```

Zr-C interaction:
```
 54. (2.00000)  58.8705% BD ( 1)Zr  6- C  8
                 4.503% Zr  6 s( 19.74%)p 0.04(  0.74%)d 4.03( 79.52%)
                54.549%  C  8 s(  0.78%)p99.99( 99.21%)d 0.02(  0.01%)
                13.620%  C  9 s(  0.12%)p99.99( 99.73%)d 1.21(  0.15%)
                 1.314%  C 10 s(  0.10%)p99.99( 99.17%)d 7.32(  0.73%)
                10.350%  C 11 s(  0.06%)p99.99( 99.94%)d 0.01(  0.00%)
                 1.336%  C 12 s(  0.30%)p99.99( 98.96%)d 2.51(  0.74%)
                13.502%  C 13 s(  0.02%)p99.99( 99.86%)d 6.58(  0.12%)
```

Zr-Me interaction:
```
 53. (2.00000)  97.1812% BD ( 1)Zr  6- C  7
                20.160% Zr  6 s( 12.08%)p 0.00(  0.05%)d 7.28( 87.88%)
                77.033%  C  7 s( 26.78%)p 2.73( 73.21%)d 0.00(  0.01%)
```

Resonance structure **4**:
NLMO algorithm failed to converge

Resonance structure **5**:
C-H interaction:
```
 61. (2.00000)  97.2981% BD ( 1) C  8- H 33
                 1.254% Zr  6 s( 29.33%)p 0.02(  0.73%)d 2.39( 69.94%)
                61.329%  C  8 s( 26.41%)p 2.78( 73.51%)d 0.00(  0.08%)
                36.005%  H 33 s( 99.97%)p 0.00(  0.03%)
```

```
C-C interaction:
  58. (2.00000)  98.3353% BD ( 1) C  8- C  9
                  49.668%  C  8 s( 32.01%)p 2.12( 67.95%)d 0.00(  0.05%)
                  48.813%  C  9 s( 29.42%)p 2.40( 70.54%)d 0.00(  0.04%)
  59. (2.00000)  82.4382% BD ( 2) C  8- C  9
                   3.707% Zr  6 s( 19.46%)p 0.04(  0.78%)d 4.10( 79.76%)
                  46.330%  C  8 s(  0.83%)p99.99( 99.13%)d 0.04(  0.03%)
                  36.127%  C  9 s(  0.10%)p99.99( 99.86%)d 0.47(  0.05%)
                   5.463%  C 10 s(  0.00%)p 1.00( 99.71%)d 0.00(  0.29%)
                   1.029%  C 11 s(  0.13%)p99.99( 99.44%)d 3.50(  0.44%)
                   1.908%  C 12 s(  0.15%)p99.99( 99.70%)d 1.02(  0.15%)
                   4.271%  C 13 s(  0.03%)p99.99( 99.61%)d10.34(  0.36%)

Zr-C interaction:
  54. (2.00000)  32.5963% BD ( 1)Zr  6- C 13
                   1.208% Zr  6 s( 11.81%)p 0.05(  0.59%)d 7.41( 87.60%)
                  10.153%  C  8 s(  0.91%)p99.99( 98.87%)d 0.24(  0.22%)
                  10.859%  C  9 s(  0.05%)p99.99( 99.80%)d 3.35(  0.15%)
                  31.188%  C 10 s(  0.01%)p99.99( 99.98%)d 1.24(  0.01%)
                   6.453%  C 11 s(  0.03%)p99.99( 99.70%)d 7.47(  0.26%)
                   7.810%  C 12 s(  0.00%)p 1.00( 99.80%)d 0.00(  0.19%)
                  31.774%  C 13 s(  0.02%)p99.99( 99.97%)d 0.46(  0.01%)

Zr-Me interaction:
  53. (2.00000)  97.1817% BD ( 1)Zr  6- C  7
                  20.156% Zr  6 s( 12.10%)p 0.00(  0.05%)d 7.26( 87.86%)
                  77.037%  C  7 s( 26.80%)p 2.73( 73.20%)d 0.00(  0.01%)
```

Non-Covalent Interactions (NCI)

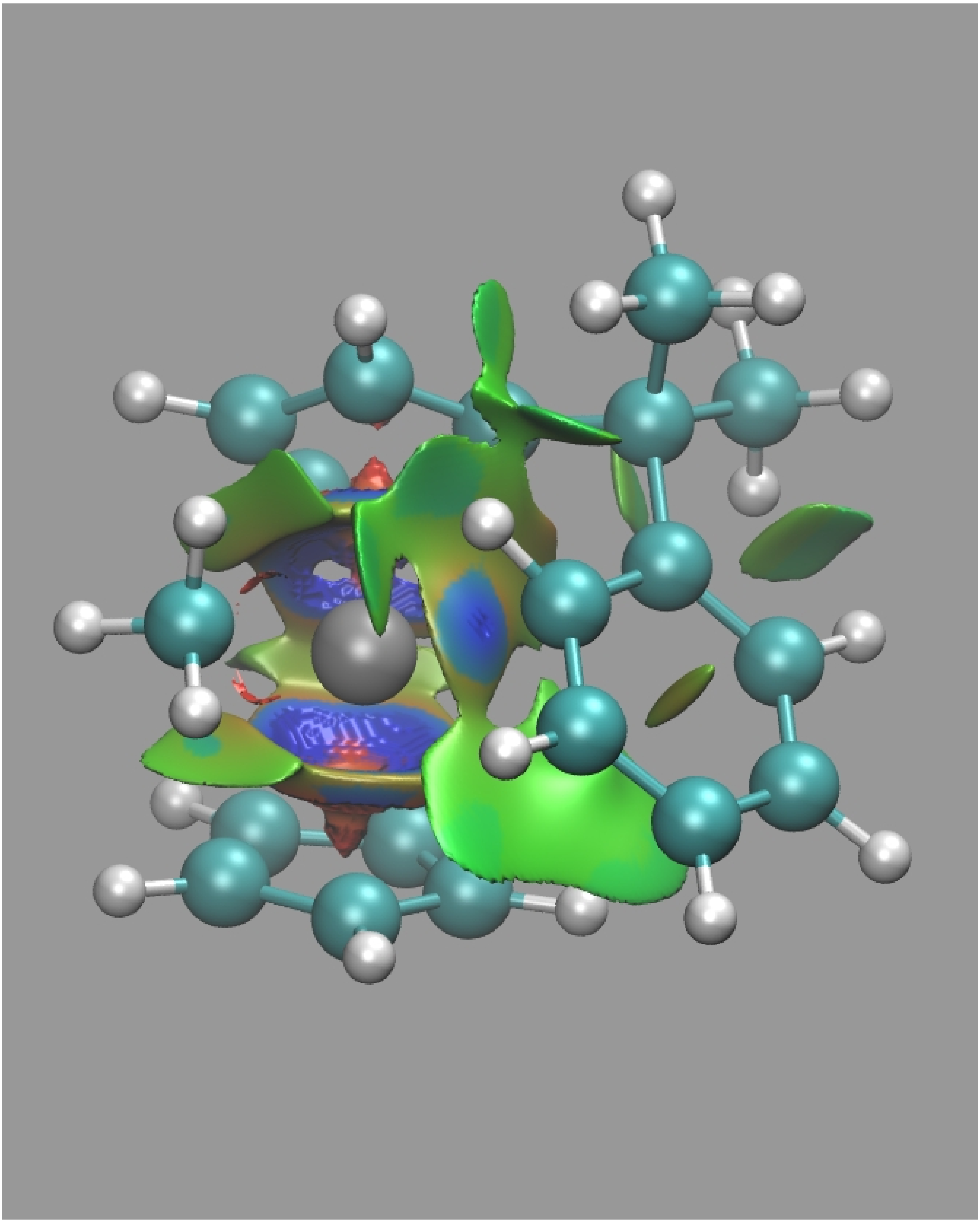

IIB-PBE-D3

Bader:

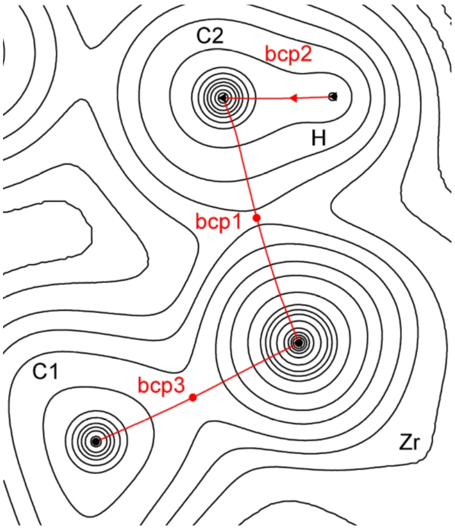

Electrondensity

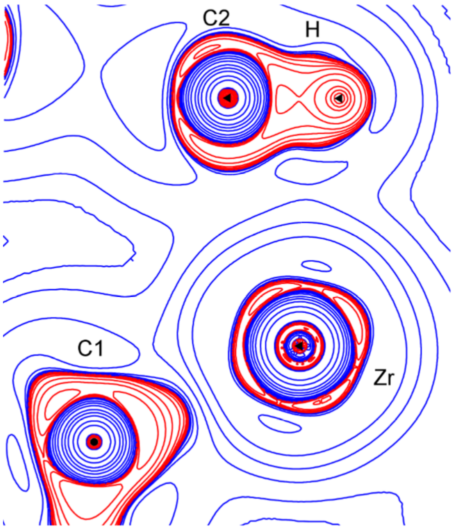

Laplacian

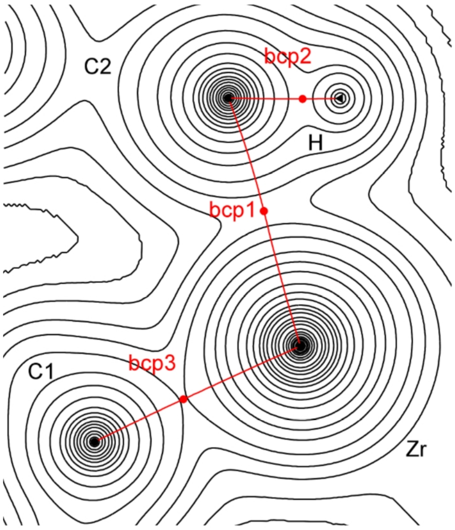

Virial

	$\varrho(\mathbf{r})$	$\nabla^2\varrho(\mathbf{r})$
bcp1	0.03807	-0.02352
bcp2	0.25920	0.20113
bcp3	0.09662	-0.01255

NBO:

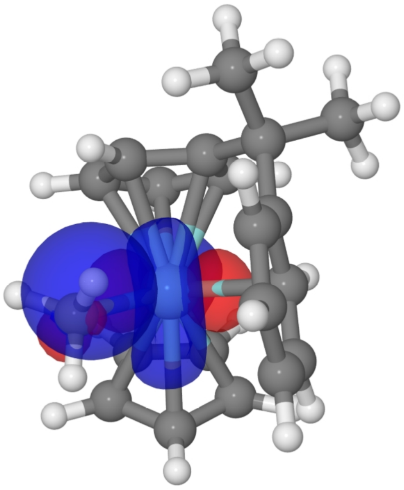

1

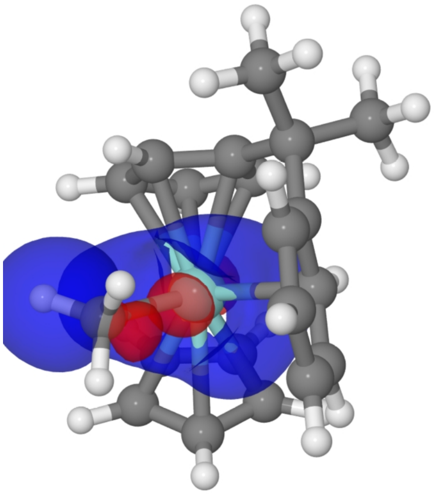

2

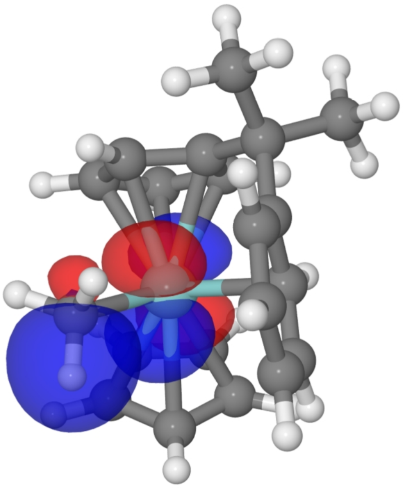

3

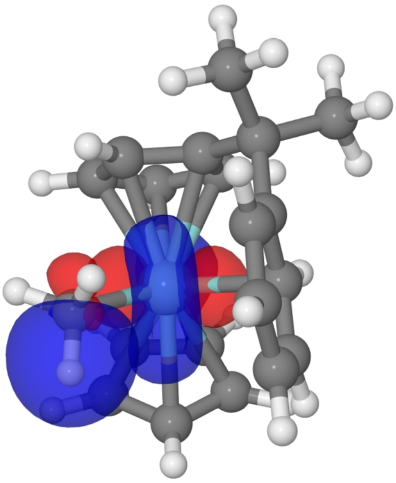

4

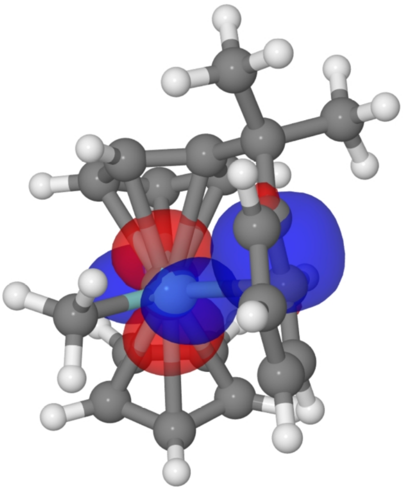

5

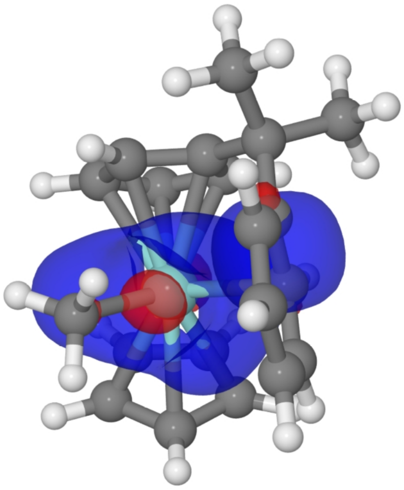

6

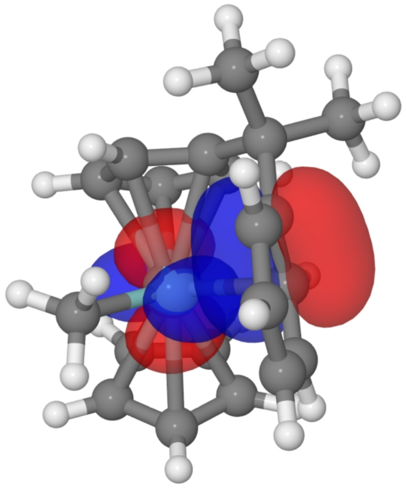

7

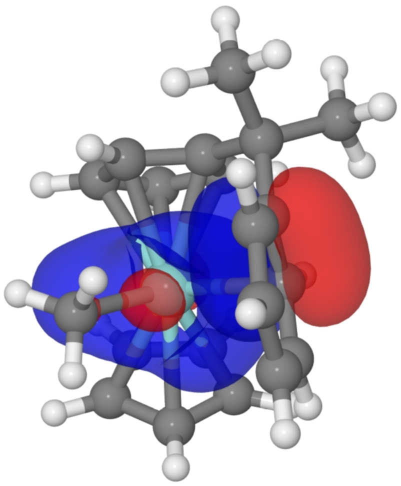

8

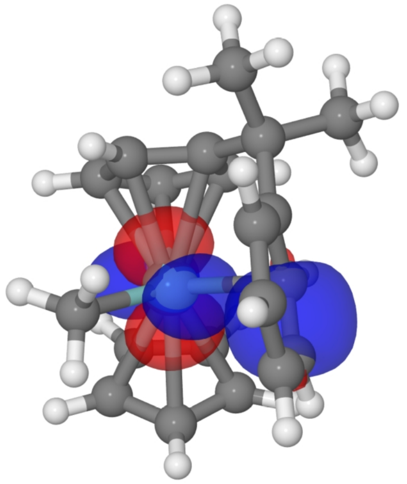

9

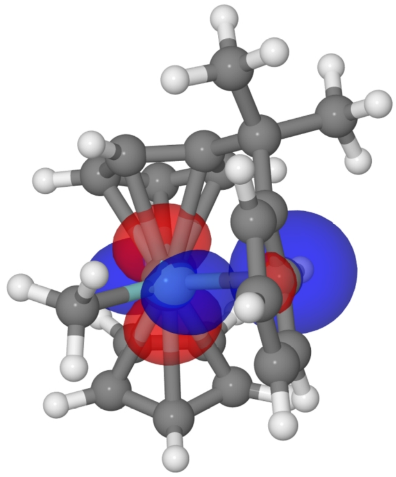

10

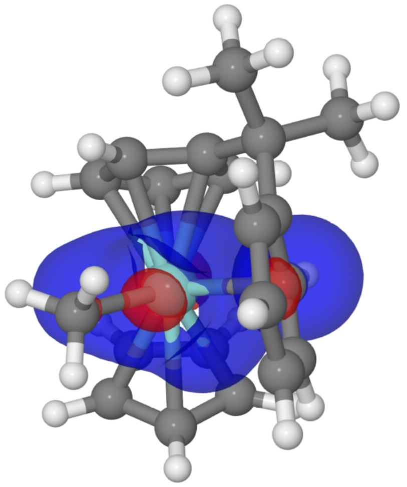

11

	Orbitals	E(2P)
1	σ_{CH} = 0.780$(sp^{3.08})_{C7}$ – 0.625$(s)_{H42}$ → LV_{Zr} = $sd^{5.39}$	5.30
2	σ_{CH} = 0.787$(sp^{3.13})_{C7}$ – 0.617$(s)_{H43}$ → LV_{Zr} = $sd^{0.44}$	2.24
3	σ_{CH} = 0.782$(sp^{2.94})_{C7}$ – 0.624$(s)_{H44}$ → LV_{Zr} = $p^{0.96}d^{99.99}$	2.46
4	σ_{CH} = 0.782$(sp^{2.94})_{C7}$ – 0.624$(s)_{H44}$ → LV_{Zr} = $sd^{5.39}$	2.39
5	σ_{CC} = 0.711$(sp^{1.74})_{C15}$ – 0.702$(sp^{2.06})_{C16}$ → LV_{Zr} = $sd^{71.56}$	2.53
6	σ_{CC} = 0.711$(sp^{1.74})_{C15}$ – 0.702$(sp^{2.06})_{C16}$ → LV_{Zr} = $sd^{0.44}$	3.35
7	π_{CC} = 0.771$(p^{99.99})_{C15}$ – 0.637$(p^{99.99})_{C16}$ → LV_{Zr} = $sd^{71.56}$	8.39
8	π_{CC} = 0.771$(p^{99.99})_{C15}$ – 0.637$(p^{99.99})_{C16}$ → LV_{Zr} = $sd^{0.44}$	5.15
9	σ_{CC} = 0.715$(sp^{1.80})_{C15}$ – 0.698$(sp^{1.79})_{C20}$ → LV_{Zr} = $sd^{71.56}$	3.12
10	σ_{CH} = 0.789$(sp^{2.67})_{C15}$ – 0.614$(s)_{H29}$ → LV_{Zr} = $sd^{71.56}$	2.97
11	σ_{CH} = 0.789$(sp^{2.67})_{C15}$ – 0.614$(s)_{H29}$ → LV_{Zr} = $sd^{0.44}$	9.09

Natural Resonance Theory:

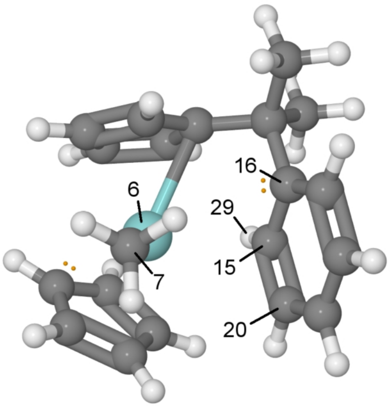

1

Wgt=14.75%;
rhoNL=5.81901;
D(0)=0.10222

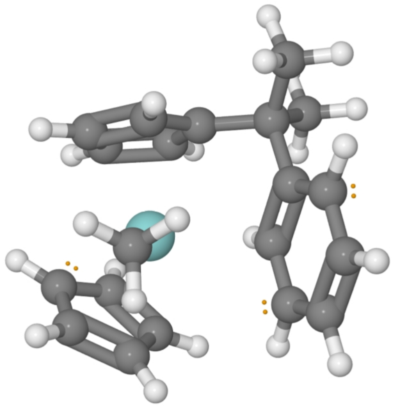

2

Wgt=13.33%;
rhoNL=6.11800;
D(0)=0.10481

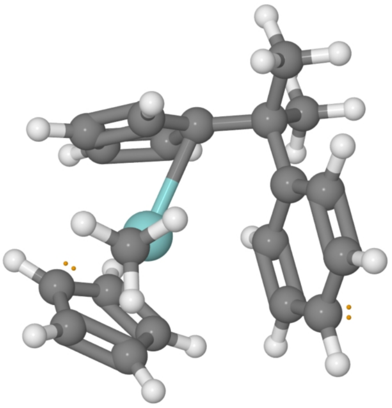

3

Wgt=12.19%;
rhoNL=5.78751;
D(0)=0.10194

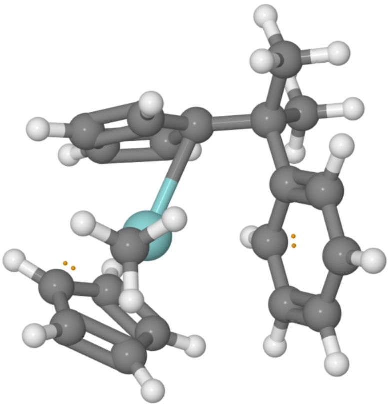

4

Wgt=9.65%;
rhoNL=5.63675;
D(0)=0.10061

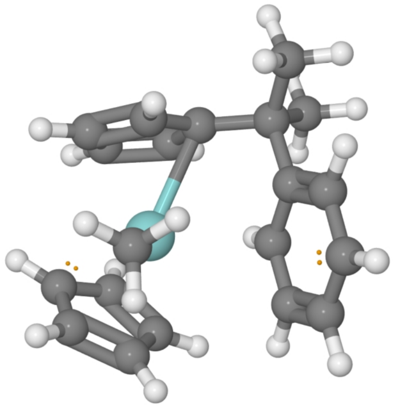

5

Wgt=6.83%;
rhoNL=5.89119;
D(0)=0.10285

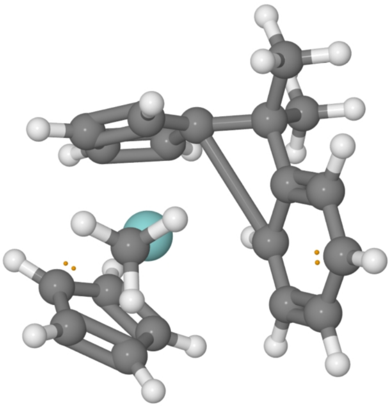

6

Wgt=6.36%;
rhoNL=5.95174;
D(0)=0.10338

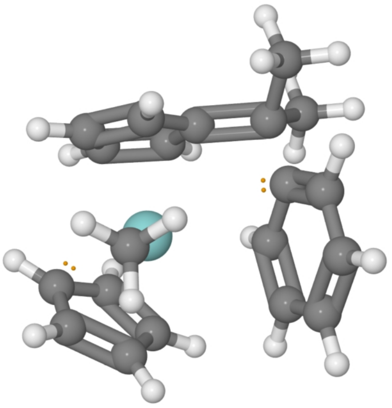

7

Wgt=5.36%;
rhoNL=6.17419;
D(0)=0.10529

Natural Localised Molecular Orbitals (NLMO):

Only contributions over 1% are reported.

NLMO / Occupancy / Percent from Parent NBO / Atomic Hybrid Contributions

Resonance structure **1**:
C-H interaction:

```
 79. (2.00000)  96.4462% BD ( 1) C 15- H 29
                  2.153% Zr  6 s( 24.65%)p 0.02(  0.47%)d 3.04( 74.88%)
                 60.135%  C 15 s( 25.70%)p 2.89( 74.21%)d 0.00(  0.09%)
                 36.344%  H 29 s( 99.97%)p 0.00(  0.03%)
```

C-C interaction:

```
 77. (2.00000)  98.7561% BD ( 1) C 15- C 20
                 50.602%  C 15 s( 32.22%)p 2.10( 67.73%)d 0.00(  0.05%)
                 48.281%  C 20 s( 32.66%)p 2.06( 67.30%)d 0.00(  0.04%)
 78. (2.00000)  84.5648% BD ( 2) C 15- C 20
                  3.095% Zr  6 s( 23.91%)p 0.03(  0.79%)d 3.15( 75.30%)
                 49.972%  C 15 s(  0.91%)p99.99( 99.08%)d 0.02(  0.02%)
                  4.656%  C 16 s(  0.17%)p99.99( 99.50%)d 1.97(  0.33%)
                  1.504%  C 17 s(  0.13%)p99.99( 99.67%)d 1.63(  0.21%)
                  1.050%  C 18 s(  0.09%)p99.99( 99.58%)d 3.67(  0.33%)
                  4.414%  C 19 s(  0.01%)p 1.00( 99.66%)d 0.00(  0.33%)
                 34.614%  C 20 s(  0.02%)p99.99( 99.94%)d 1.82(  0.04%)
```

Zr-Me interaction:

```
 53. (2.00000)  97.4294% BD ( 1)Zr  6- C  7
                 21.130% Zr  6 s( 11.67%)p 0.00(  0.04%)d 7.57( 88.29%)
                 76.314%  C  7 s( 26.35%)p 2.79( 73.64%)d 0.00(  0.01%)
```

Resonance structure **2**:
C-H interaction:

```
 78. (2.00000)  98.7536% BD ( 1) C 15- C 20
                 50.595%  C 15 s( 32.30%)p 2.09( 67.66%)d 0.00(  0.05%)
                 48.283%  C 20 s( 32.70%)p 2.06( 67.26%)d 0.00(  0.04%)
```

C-C interaction:
76. (2.00000) 98.4324% BD (1) C 15- C 16
0.014% C 2 s(17.81%)p 4.61(82.03%)d 0.01(0.15%)
0.213% Zr 6 s(23.49%)p 0.15(3.44%)d 3.11(73.07%)
0.213% C 13 s(0.70%)p99.99(97.45%)d 2.62(1.85%)
0.100% C 14 s(17.26%)p 4.74(81.78%)d 0.06(0.95%)
49.970% C 15 s(32.67%)p 2.06(67.29%)d 0.00(0.04%)
48.613% C 16 s(29.20%)p 2.42(70.76%)d 0.00(0.04%)
77. (2.00000) 82.7638% BD (2) C 15- C 16
2.806% Zr 6 s(24.03%)p 0.04(0.87%)d 3.12(75.09%)
1.033% C 9 s(1.37%)p71.97(98.35%)d 0.21(0.29%)
50.348% C 15 s(0.75%)p99.99(99.24%)d 0.02(0.02%)
32.449% C 16 s(0.13%)p99.99(99.82%)d 0.33(0.04%)
4.010% C 17 s(0.00%)p 1.00(99.62%)d 0.00(0.38%)
1.239% C 18 s(0.06%)p99.99(99.68%)d 4.65(0.27%)
1.484% C 19 s(0.10%)p99.99(99.68%)d 2.11(0.22%)
5.735% C 20 s(0.03%)p99.99(99.70%)d 9.33(0.27%)
Zr-Me interaction:
54. (2.00000) 96.7812% BD (1)Zr 6- C 7
20.956% Zr 6 s(11.37%)p 0.00(0.04%)d 7.79(88.59%)
75.886% C 7 s(26.36%)p 2.79(73.63%)d 0.00(0.01%)

Resonance structure **3**:
C-H interaction:
79. (2.00000) 96.4468% BD (1) C 15- H 29
2.147% Zr 6 s(24.71%)p 0.02(0.47%)d 3.03(74.82%)
60.128% C 15 s(25.70%)p 2.89(74.21%)d 0.00(0.09%)

C-C interaction:
77. (2.00000) 98.7561% BD (1) C 15- C 20
50.603% C 15 s(32.21%)p 2.10(67.74%)d 0.00(0.05%)
48.280% C 20 s(32.66%)p 2.06(67.30%)d 0.00(0.04%)
78. (2.00000) 84.5046% BD (2) C 15- C 20
3.129% Zr 6 s(23.88%)p 0.03(0.79%)d 3.15(75.33%)
50.651% C 15 s(0.90%)p99.99(99.08%)d 0.02(0.02%)
5.205% C 16 s(0.16%)p99.99(99.54%)d 1.86(0.30%)
1.274% C 17 s(0.15%)p99.99(99.59%)d 1.83(0.27%)
1.043% C 18 s(0.09%)p99.99(99.60%)d 3.40(0.31%)
4.099% C 19 s(0.01%)p 1.00(99.64%)d 0.00(0.35%)
33.888% C 20 s(0.02%)p99.99(99.94%)d 1.89(0.04%)

Zr-Me interaction:
53. (2.00000) 97.4297% BD (1)Zr 6- C 7
21.129% Zr 6 s(11.67%)p 0.00(0.04%)d 7.56(88.29%)
76.315% C 7 s(26.35%)p 2.79(73.64%)d 0.00(0.01%)

Resonance structure **4**:
C-H interaction:

```
  78. (2.00000)  96.4692% BD ( 1) C 15- H 29
                   2.196% Zr  6 s( 24.94%)p 0.02(  0.46%)d 2.99( 74.59%)
                  60.176%  C 15 s( 25.82%)p 2.87( 74.09%)d 0.00(  0.09%)
                  36.314%  H 29 s( 99.97%)p 0.00(  0.03%)
```

C-C interaction:

```
  76. (2.00000)  98.4241% BD ( 1) C 15- C 16
                  49.976%  C 15 s( 32.72%)p 2.05( 67.23%)d 0.00(  0.04%)
                  48.603%  C 16 s( 29.19%)p 2.43( 70.77%)d 0.00(  0.04%)
  77. (2.00000)  98.7489% BD ( 1) C 15- C 20
                  50.604%  C 15 s( 32.25%)p 2.10( 67.70%)d 0.00(  0.05%)
                  48.276%  C 20 s( 32.66%)p 2.06( 67.30%)d 0.00(  0.04%)
```

Zr-Me interaction:

```
  53. (2.00000)  97.4296% BD ( 1)Zr  6- C  7
                  21.128% Zr  6 s( 11.67%)p 0.00(  0.04%)d 7.57( 88.29%)
                  76.316%  C  7 s( 26.35%)p 2.79( 73.64%)d 0.00(  0.01%)
```

Resonance structure **5**:
NLMO algorithm failed to converge

Resonance structure **6**:
C-H interaction:

```
  78. (2.00000)  96.4720% BD ( 1) C 15- H 29
                   2.195% Zr  6 s( 24.98%)p 0.02(  0.46%)d 2.99( 74.56%)
                  60.185%  C 15 s( 25.84%)p 2.87( 74.07%)d 0.00(  0.09%)
                  36.307%  H 29 s( 99.97%)p 0.00(  0.03%)
```

C-C interaction:

```
 63. (2.00000)  54.0930% BD ( 1) C  9- C 15
                   2.757% Zr  6 s( 23.86%)p 0.05(  1.24%)d 3.14( 74.90%)
                   4.263%  C  8 s(  0.66%)p99.99( 99.10%)d 0.37(  0.25%)
                  21.184%  C  9 s(  0.42%)p99.99( 99.55%)d 0.08(  0.03%)
                   4.673%  C 10 s(  0.66%)p99.99( 99.09%)d 0.38(  0.25%)
                   1.102%  C 12 s(  0.36%)p99.99( 99.43%)d 0.57(  0.21%)
                  35.254%  C 15 s(  0.61%)p99.99( 99.38%)d 0.02(  0.01%)
                  12.298%  C 16 s(  0.50%)p99.99( 99.38%)d 0.25(  0.12%)
                   1.027%  C 17 s(  0.95%)p99.99( 98.27%)d 0.82(  0.78%)
                   8.219%  C 18 s(  0.00%)p 1.00( 99.99%)d 0.00(  0.00%)
                   1.689%  C 19 s(  0.05%)p99.99( 99.60%)d 6.89(  0.35%)
                   5.968%  C 20 s(  0.24%)p99.99( 99.61%)d 0.65(  0.15%)
```

Zr-Me interaction:

```
  53. (2.00000)  97.4261% BD ( 1)Zr  6- C  7
                  21.133% Zr  6 s( 11.61%)p 0.00(  0.04%)d 7.61( 88.35%)
                  76.309%  C  7 s( 26.34%)p 2.80( 73.65%)d 0.00(  0.01%)
```

Resonance structure **7**:

C-H interaction:

```
  78. (2.00000)  96.4465% BD ( 1) C 15- H 29
                  2.162% Zr  6 s( 24.42%)p 0.02(  0.48%)d 3.08( 75.10%)
                 60.155%  C 15 s( 25.79%)p 2.87( 74.12%)d 0.00(  0.09%)
                 36.348%  H 29 s( 99.97%)p 0.00(  0.03%)
```

C-C interaction:

```
  76. (2.00000)  98.7569% BD ( 1) C 15- C 20
                 50.601%  C 15 s( 32.18%)p 2.11( 67.77%)d 0.00(  0.05%)
                 48.283%  C 20 s( 32.69%)p 2.06( 67.27%)d 0.00(  0.04%)
  77. (2.00000)  84.4688% BD ( 2) C 15- C 20
                  3.024% Zr  6 s( 22.54%)p 0.04(  0.81%)d 3.40( 76.65%)
                 50.006%  C 15 s(  0.79%)p99.99( 99.20%)d 0.02(  0.02%)
                  4.716%  C 16 s(  0.15%)p99.99( 99.51%)d 2.17(  0.33%)
                  1.552%  C 17 s(  0.13%)p99.99( 99.67%)d 1.54(  0.20%)
                  1.104%  C 18 s(  0.10%)p99.99( 99.59%)d 3.23(  0.31%)
                  4.348%  C 19 s(  0.01%)p 1.00( 99.66%)d 0.00(  0.33%)
                 34.469%  C 20 s(  0.02%)p99.99( 99.94%)d 1.92(  0.04%)
```

Zr-Me interaction:

```
  53. (2.00000)  97.4330% BD ( 1)Zr  6- C  7
                 21.113% Zr  6 s( 11.66%)p 0.00(  0.04%)d 7.58( 88.31%)
                 76.335%  C  7 s( 26.35%)p 2.79( 73.64%)d 0.00(  0.01%)
```

Non-Covalent Interactions (NCI)

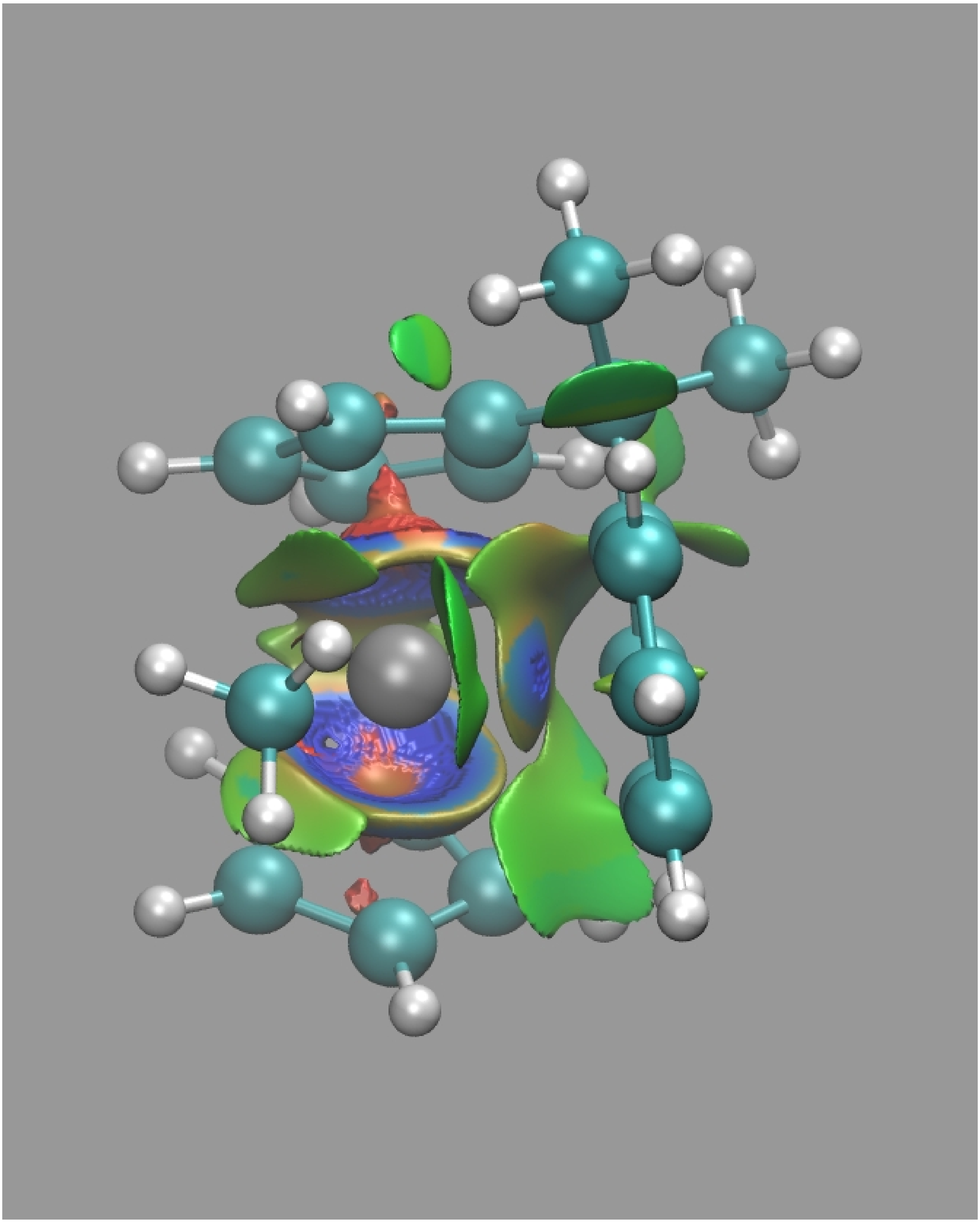

1A-PBE-D3

Bader:

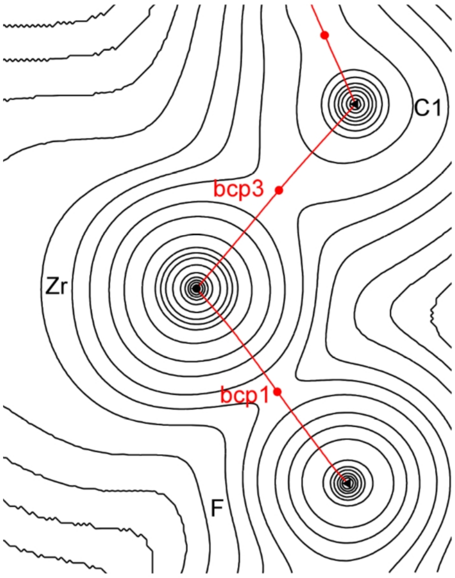

Electrondensity

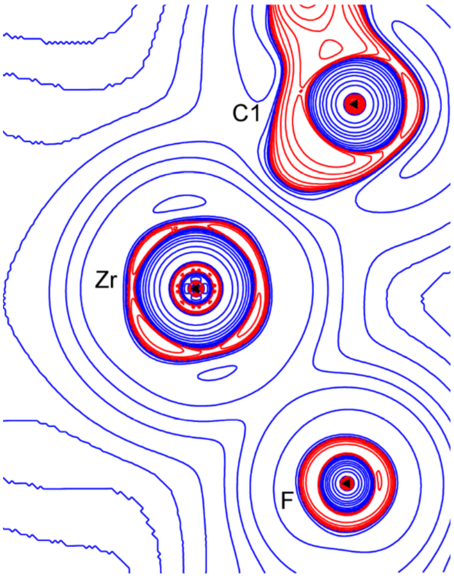

Laplacian

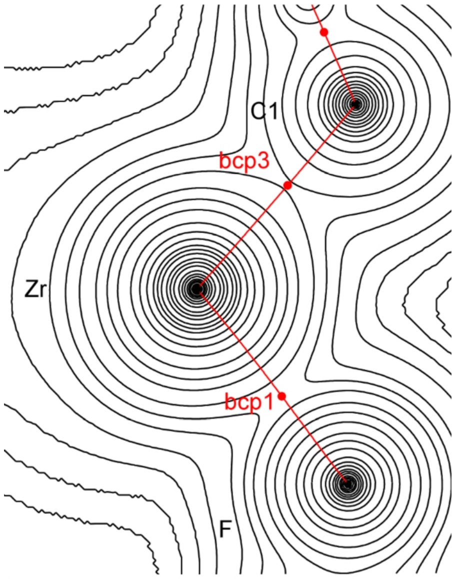

Virial

	ϱ(**r**)	∇^2ϱ(**r**)
bcp1	0.04497	-0.06113
bcp2	0.19093	-0.00064
bcp3	0.09641	-0.01195

NBO:

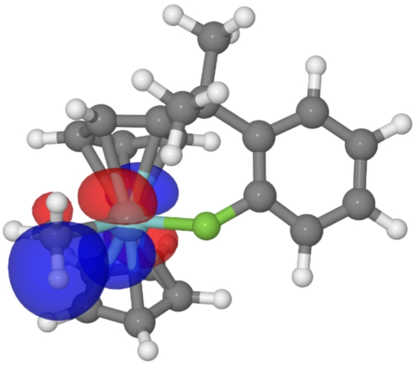

1

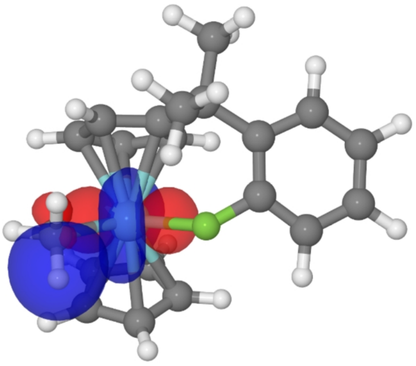

2

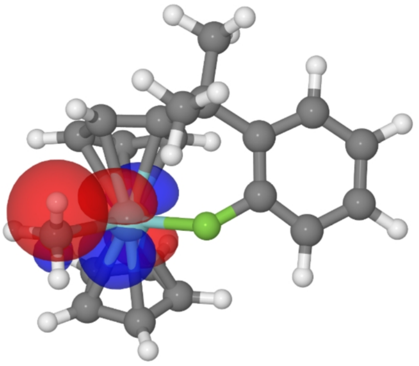

3

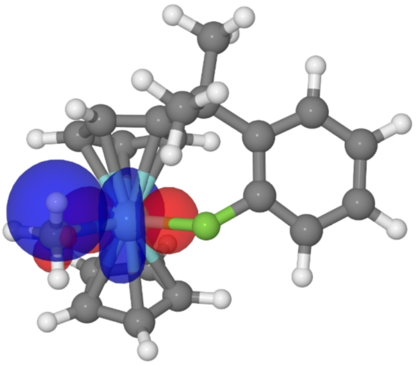

4

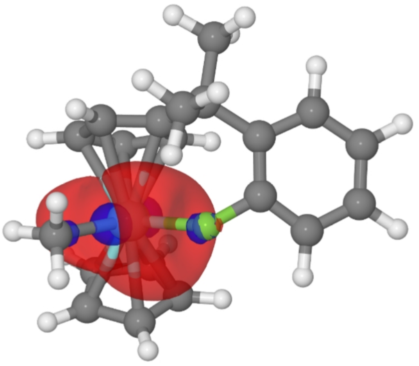

5

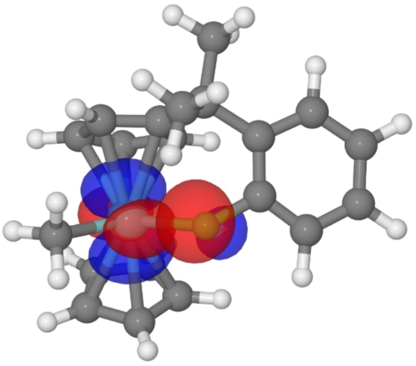

6

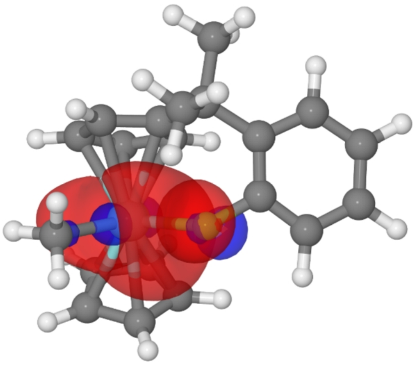

7

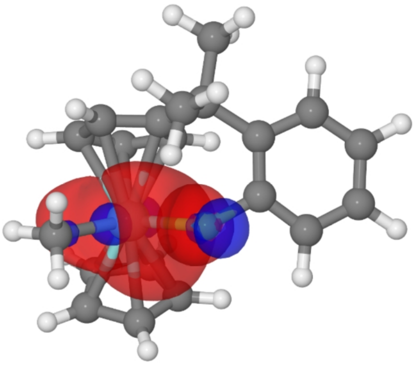

8

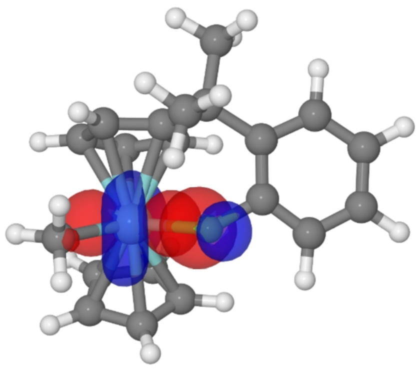

9

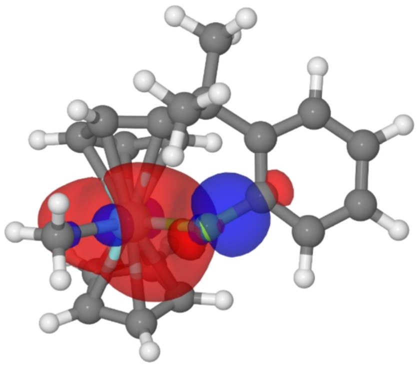

10

	Orbitals	E(2P)
1	σ_{CH} = 0.782$(sp^{2.96})_{C7}$ – 0.624$(s)_{H43}$ → LV_{Zr} = $pd^{99.99}$	2.16
2	σ_{CH} = 0.782$(sp^{2.96})_{C7}$ – 0.624$(s)_{H43}$ → LV_{Zr} = $sd^{28.20}$	2.11
3	σ_{CH} = 0.781$(sp^{2.97})_{C7}$ – 0.625$(s)_{H44}$ → LV_{Zr} = $pd^{99.99}$	2.11
4	σ_{CH} = 0.781$(sp^{2.97})_{C7}$ – 0.625$(s)_{H44}$ → LV_{Zr} = $sd^{28.20}$	2.15
5	CR = $(s)_{F8}$ → LV_{Zr} = $sd^{0.20}$	3.03
6	LP = $(sp^{2.45})_{F8}$ → LV_{Zr} = $p^{0.69}d^{99.99}$	3.80
7	LP = $(sp^{2.45})_{F8}$ → LV_{Zr} = $sd^{0.20}$	10.64
8	LP = $(sp^{3.10})_{F8}$ → LV_{Zr} = $sd^{0.20}$	18.40
9	LP = $(sp^{3.10})_{F8}$ → LV_{Zr} = $sd^{28.20}$	5.32
10	σ_{CF} = 0.875$(sp^{2.65})_{F8}$ – 0.484$(sp^{5.19})_{C9}$ → LV_{Zr} = $sd^{0.20}$	3.20

Natural Resonance Theory:

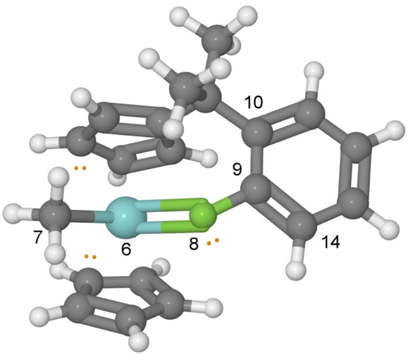

1

Wgt=20.27%;
rhoNL=5.28319;
D(0)=0.09628

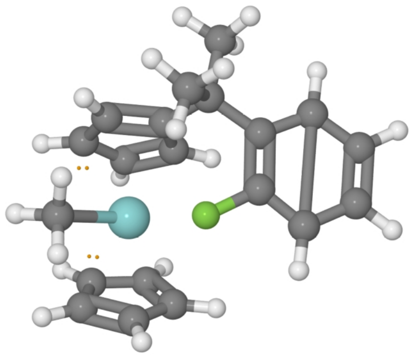

2

Wgt=14.56%;
rhoNL=5.69598;
D(0)=0.0999

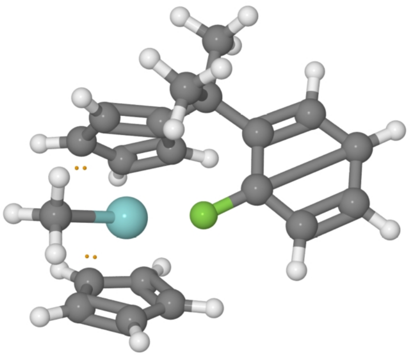

3

Wgt=12.68%;
rhoNL=5.71037;
D(0)=0.10010

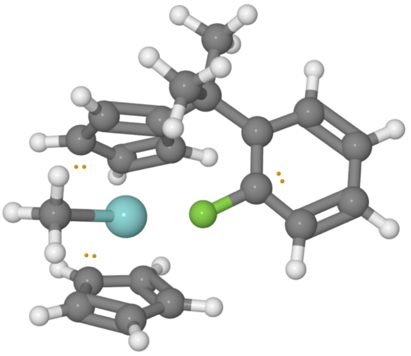

4

Wgt=7.38%;
rhoNL=5.98453;
D(0)=0.10247

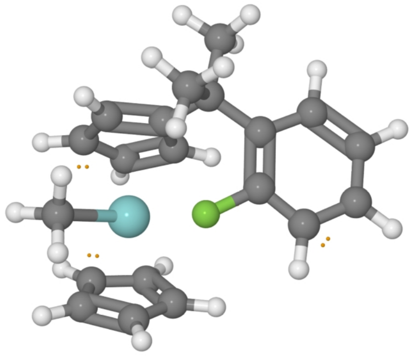

5

Wgt=5.74%;
rhoNL=6.00737;
D(0)=0.10267

Natural Localised Molecular Orbitals (NLMO):

Only contributions over 1% are reported.

NLMO / Occupancy / Percent from Parent NBO / Atomic Hybrid Contributions

```
Resonance structure 1:
C-F interaction:
  61. (2.00000)  99.6043% BD ( 1) F  8- C  9
                  76.626%  F  8 s( 35.25%)p 1.84( 64.73%)d 0.00(  0.02%)
                  23.010%  C  9 s( 18.31%)p 4.44( 81.37%)d 0.02(  0.31%)

Zr-F interaction:
  56. (2.00000)  99.0894% BD ( 1)Zr  6- F  8
                   2.836% Zr  6 s( 36.52%)p 0.01(  0.23%)d 1.73( 63.25%)
                  96.418%  F  8 s( 46.64%)p 1.14( 53.35%)d 0.00(  0.00%)
  57. (2.00000)  97.5527% BD ( 2)Zr  6- F  8
                   1.380% Zr  6 s(  7.34%)p 0.13(  0.99%)d12.48( 91.67%)
                  96.358%  F  8 s(  0.19%)p99.99( 99.80%)d 0.04(  0.01%)
                   1.004%  C  9 s(  3.06%)p30.35( 92.94%)d 1.30(  3.99%)

C-C interaction:
  63. (2.00000)  98.9568% BD ( 1) C  9- C 14
                  49.982%  C  9 s( 39.69%)p 1.52( 60.28%)d 0.00(  0.03%)
                  49.074%  C 14 s( 31.77%)p 2.15( 68.16%)d 0.00(  0.07%)

Zr-Me interaction:
  55. (2.00000)  97.4416% BD ( 1)Zr  6- C  7
                  22.024% Zr  6 s( 12.03%)p 0.00(  0.06%)d 7.31( 87.91%)
                  75.482%  C  7 s( 26.30%)p 2.80( 73.70%)d 0.00(  0.01%)

Resonance structure 2:
C-F interaction:
  61. (2.00000)  99.5468% BD ( 1) F  8- C  9
                  76.262%  F  8 s( 27.47%)p 2.64( 72.51%)d 0.00(  0.02%)
                  23.320%  C  9 s( 18.95%)p 4.26( 80.74%)d 0.02(  0.31%)

C-C interaction:
  62. (2.00000)  98.7522% BD ( 1) C  9- C 10
                  49.272%  C  9 s( 40.16%)p 1.49( 59.82%)d 0.00(  0.03%)
                  49.620%  C 10 s( 28.31%)p 2.53( 71.62%)d 0.00(  0.07%)
  63. (2.00000)  84.2807% BD ( 2) C  9- C 10
                  43.639%  C  9 s(  0.01%)p99.99( 99.96%)d 1.93(  0.03%)
                  40.644%  C 10 s(  0.01%)p99.99( 99.93%)d 5.49(  0.06%)

Zr-Me interaction:
  57. (2.00000)  97.4906% BD ( 1)Zr  6- C  7
                  22.016% Zr  6 s( 11.96%)p 0.01(  0.06%)d 7.36( 87.98%)
                  75.488%  C  7 s( 26.25%)p 2.81( 73.74%)d 0.00(  0.01%)
```

Resonance structure **3**:
C-F interaction:
61. (2.00000) 99.5495% BD (1) F 8- C 9
76.258% F 8 s(27.47%)p 2.64(72.51%)d 0.00(0.02%)
23.324% C 9 s(18.94%)p 4.26(80.75%)d 0.02(0.31%)

C-C interaction:
63. (2.00000) 66.6642% BD (1) C 9- C 12
37.045% C 9 s(0.01%)p99.99(99.98%)d 0.08(0.00%)
8.285% C 10 s(0.02%)p99.99(99.69%)d11.63(0.28%)
8.082% C 11 s(0.00%)p 1.00(99.82%)d 0.00(0.18%)
29.690% C 12 s(0.00%)p 1.00(99.99%)d 0.00(0.01%)
8.059% C 13 s(0.00%)p 1.00(99.82%)d 0.00(0.18%)
8.553% C 14 s(0.01%)p99.99(99.70%)d22.18(0.28%)

Zr-Me interaction:
57. (2.00000) 97.4907% BD (1)Zr 6- C 7
22.016% Zr 6 s(11.96%)p 0.01(0.06%)d 7.36(87.98%)
75.488% C 7 s(26.25%)p 2.81(73.74%)d 0.00(0.01%)

Resonance structure **4**:
C-F interaction:
62. (2.00000) 99.5495% BD (1) F 8- C 9
76.258% F 8 s(27.47%)p 2.64(72.51%)d 0.00(0.02%)
23.324% C 9 s(18.94%)p 4.26(80.75%)d 0.02(0.31%)

Zr-Me interaction:
58. (2.00000) 97.4906% BD (1)Zr 6- C 7
22.016% Zr 6 s(11.96%)p 0.01(0.06%)d 7.36(87.98%)
75.488% C 7 s(26.25%)p 2.81(73.74%)d 0.00(0.01%)

Resonance structure **5**:
C-F interaction:
62. (2.00000) 99.5469% BD (1) F 8- C 9
76.262% F 8 s(27.47%)p 2.64(72.51%)d 0.00(0.02%)
23.320% C 9 s(18.95%)p 4.26(80.74%)d 0.02(0.31%)

C-C interaction:
63. (2.00000) 98.7522% BD (1) C 9- C 10
49.272% C 9 s(40.16%)p 1.49(59.82%)d 0.00(0.03%)
49.620% C 10 s(28.31%)p 2.53(71.62%)d 0.00(0.07%)
64. (2.00000) 84.2866% BD (2) C 9- C 10
43.788% C 9 s(0.01%)p99.99(99.96%)d 1.91(0.03%)
40.501% C 10 s(0.01%)p99.99(99.93%)d 5.36(0.06%)

Zr-Me interaction:
58. (2.00000) 97.4906% BD (1)Zr 6- C 7
22.016% Zr 6 s(11.96%)p 0.01(0.06%)d 7.36(87.98%)
75.488% C 7 s(26.25%)p 2.81(73.74%)d 0.00(0.01%)

Non-Covalent Interactions (NCI)

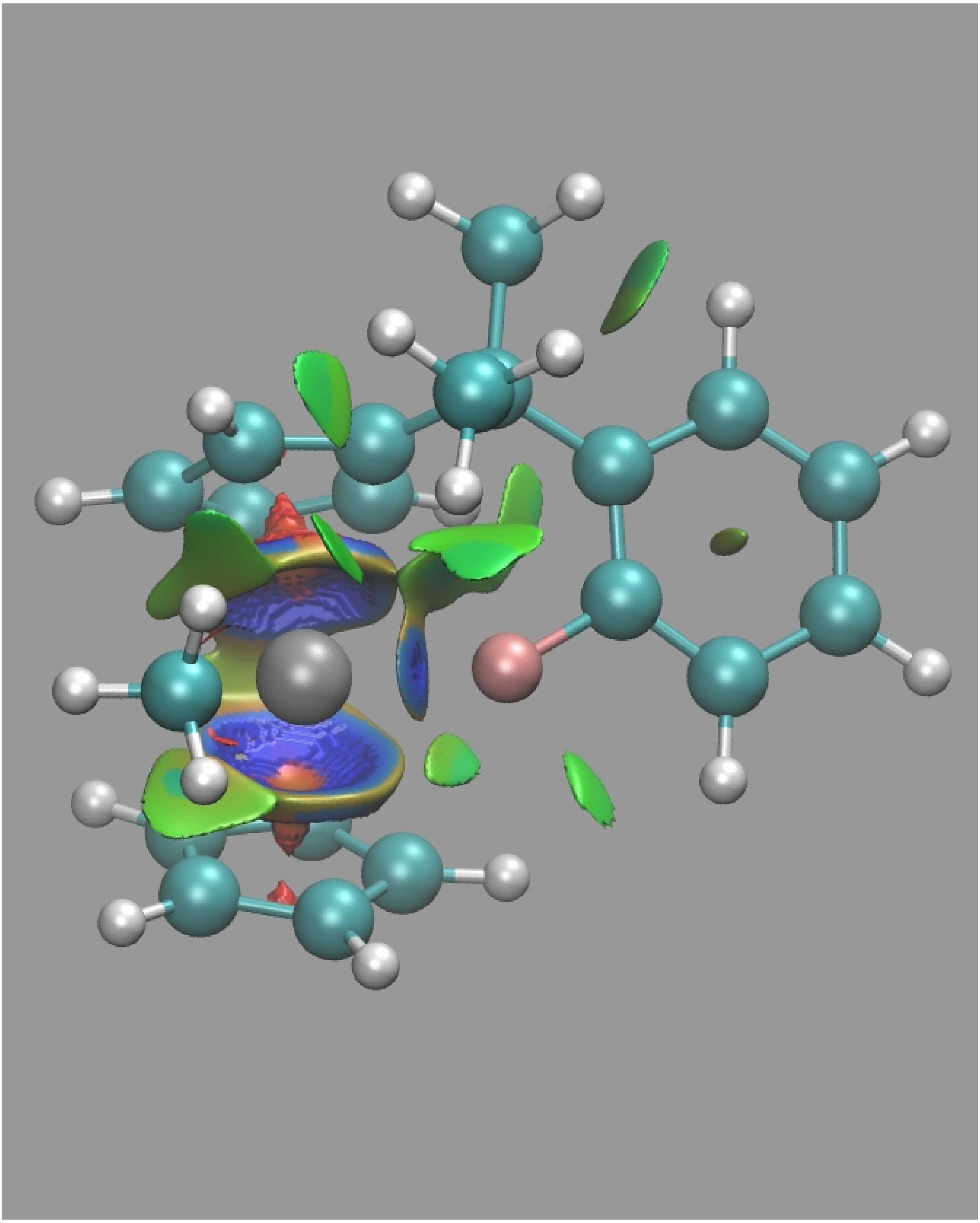

1B-PBE-D3

Bader:

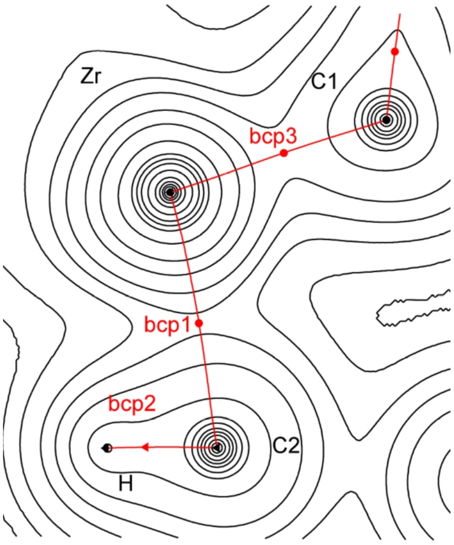

Electrondensity

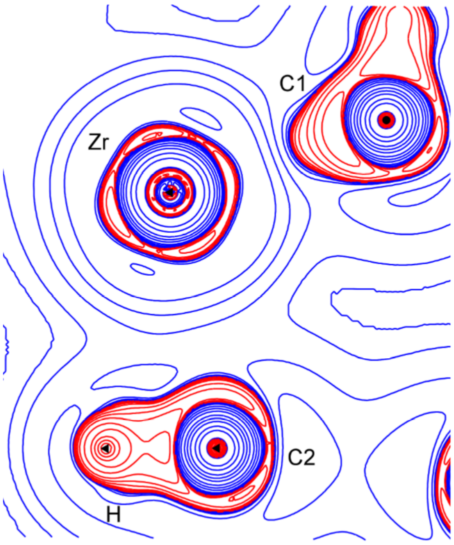

Laplacian

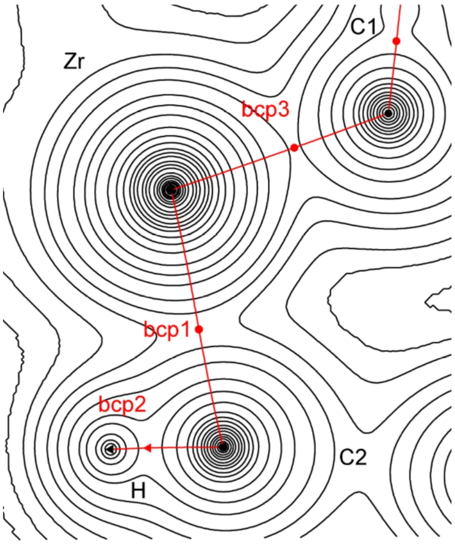

Virial

	$\varrho(\mathbf{r})$	$\nabla^2\varrho(\mathbf{r})$
bcp1	0.03663	-0.02252
bcp2	0.26348	0.20844
bcp3	0.09535	-0.01190

NBO:

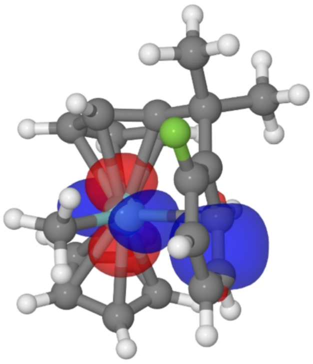

1

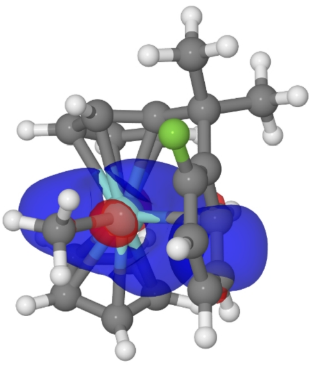

2

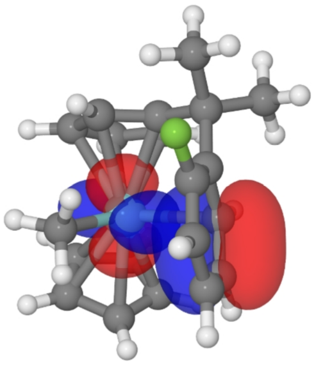

3

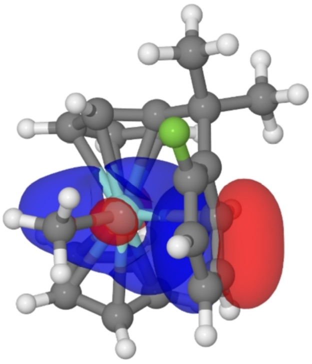

4

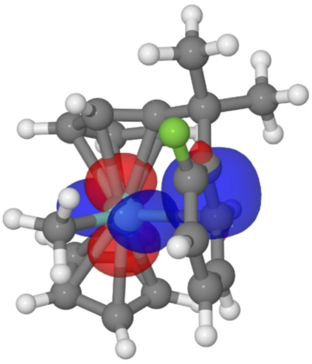

5

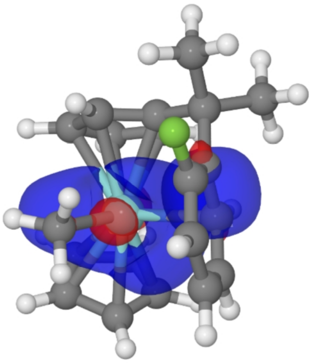

6

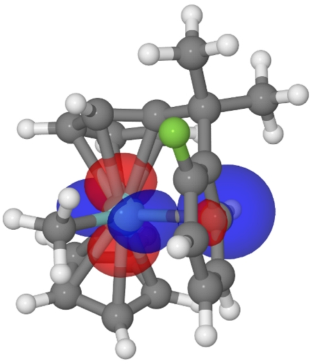

7

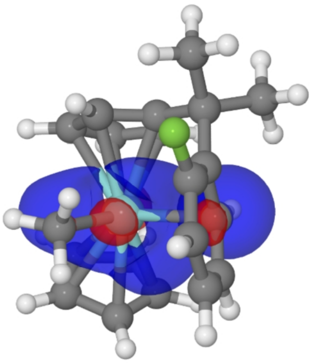

8

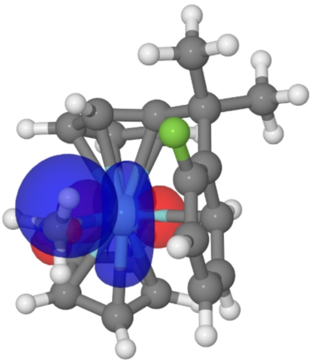

9

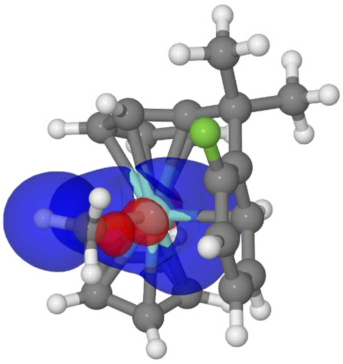

10

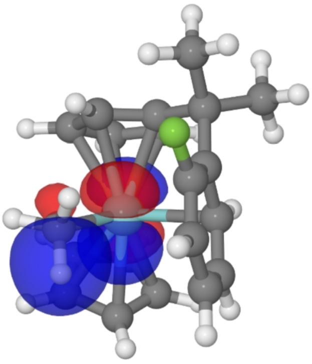

11

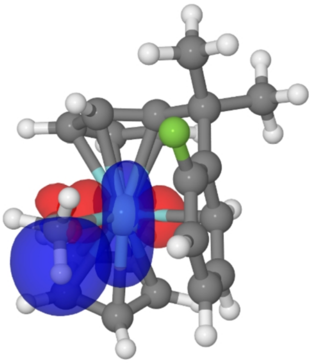

12

	Orbitals	E(2P)
1	σ_{CC} = 0.699$(sp^{1.80})_{C1}$ – 0.716$(sp^{1.76})_{C2}$ → LV_{Zr} = $sd^{99.99}$	3.31
2	σ_{CC} = 0.699$(sp^{1.80})_{C1}$ – 0.716$(sp^{1.76})_{C2}$ → LV_{Zr} = $sp^{0.42}$	2.10
3	σ_{CC} = 0.655$(p)_{C1}$ – 0.756$(p)_{C2}$ → LV_{Zr} = $sd^{99.99}$	7.37
4	π_{CC} = 0.655$(p)_{C1}$ – 0.756$(p)_{C2}$ → LV_{Zr} = $sp^{0.42}$	5.51
5	σ_{CC} = 0.708$(sp^{1.79})_{C2}$ – 0.706$(sp^{2.04})_{C3}$ → LV_{Zr} = $sd^{99.99}$	3.06
6	σ_{CC} = 0.708$(sp^{1.79})_{C2}$ – 0.706$(sp^{2.04})_{C3}$ → LV_{Zr} = $sp^{0.42}$	3.85
7	σ_{CH} = 0.791$(sp^{2.63})_{C2}$ – 0.612$(s)_{H26}$ → LV_{Zr} = $sd^{99.99}$	2.83
8	σ_{CH} = 0.791$(sp^{2.63})_{C2}$ – 0.612$(s)_{H26}$ → LV_{Zr} = $sp^{0.42}$	7.68
9	σ_{CH} = 0.780$(sp^{2.99})_{C21}$ – 0.626$(s)_{H37}$ → LV_{Zr} = $sd^{5.92}$	3.55
10	σ_{CH} = 0.787$(sp^{3.19})_{C21}$ – 0.6171$(s)_{H38}$ → LV_{Zr} = $sp^{0.42}$	2.38
11	σ_{CH} = 0.781$(sp^{2.99})_{C21}$ – 0.625$(s)_{H39}$ → LV_{Zr} =	2.32
12	σ_{CH} = 0.781$(sp^{2.99})_{C21}$ – 0.625$(s)_{H39}$ → LV_{Zr} = $sd^{5.92}$	3.17

Natural Resonance Theory:

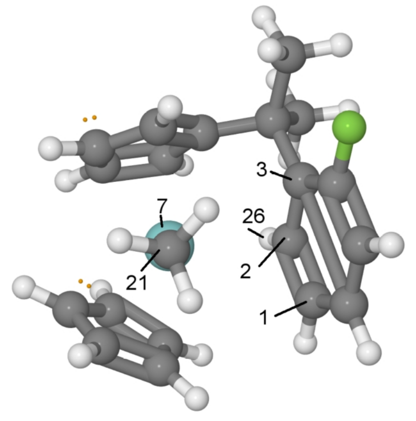	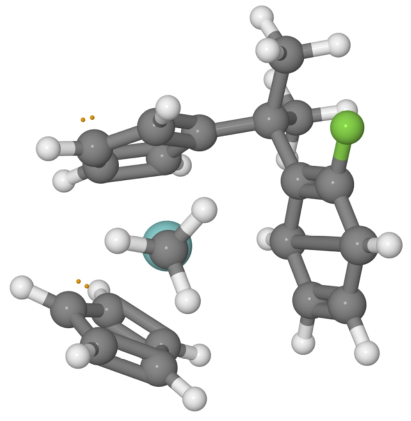	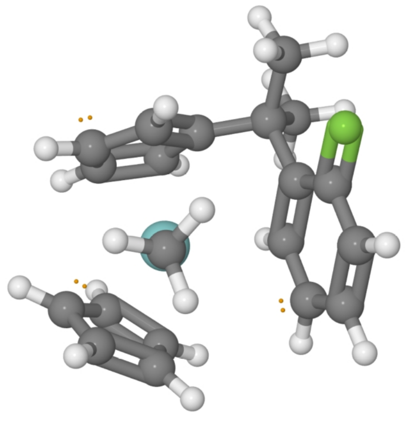
**1**	**2**	**3**
Wgt=19.09%;<br>rhoNL=5.78534;<br>D(0)=0.10075	Wgt=14.78%;<br>rhoNL=5.75336;<br>D(0)=0.10048	Wgt=14.64%;<br>rhoNL=5.99705;<br>D(0)=0.10258

Natural Localised Molecular Orbitals (NLMO):

Only contributions over 1% are reported.

NLMO / Occupancy / Percent from Parent NBO / Atomic Hybrid Contributions

Resonance structure **1**:
C-H interaction:
50. (2.00000) 96.9321% BD (1) C 2- H 26
60.751% C 2 s(26.07%)p 2.83(73.85%)d 0.00(0.08%)
1.664% Zr 7 s(27.50%)p 0.02(0.47%)d 2.62(72.04%)
36.209% H 26 s(99.97%)p 0.00(0.03%)

C-C interaction:
45. (2.00000) 98.7592% BD (1) C 1- C 2
48.265% C 1 s(32.63%)p 2.06(67.33%)d 0.00(0.04%)
50.612% C 2 s(32.84%)p 2.04(67.12%)d 0.00(0.05%)
46. (2.00000) 84.7659% BD (2) C 1- C 2
35.923% C 1 s(0.03%)p99.99(99.93%)d 1.15(0.04%)
48.864% C 2 s(0.79%)p99.99(99.19%)d 0.03(0.02%)

Zr-Me interaction:
60. (2.00000) 97.2864% BD (1)Zr 7- C 21
20.648% Zr 7 s(11.82%)p 0.00(0.03%)d 7.46(88.15%)
76.651% C 21 s(26.51%)p 2.77(73.48%)d 0.00(0.01%)

Resonance structure **2**:

C-H interaction:

```
 51. (2.00000)  96.9454% BD ( 1) C  2- H 26
                60.785%  C  2 s( 26.17%)p 2.82( 73.75%)d 0.00(  0.08%)
                 1.704% Zr  7 s( 27.95%)p 0.02(  0.46%)d 2.56( 71.59%)
                36.180%  H 26 s( 99.97%)p 0.00(  0.03%)
```

C-C interaction:

```
 50. (2.00000)  68.1072% BD ( 1) C  2- C  5
                 7.585%  C  1 s(  0.04%)p99.99( 99.72%)d 5.93(  0.24%)
                43.544%  C  2 s(  0.71%)p99.99( 99.28%)d 0.01(  0.01%)
                 6.742%  C  3 s(  0.15%)p99.99( 99.59%)d 1.71(  0.26%)
                 6.297%  C  4 s(  0.06%)p99.99( 99.67%)d 4.67(  0.27%)
                24.622%  C  5 s(  0.01%)p99.99( 99.97%)d 1.38(  0.02%)
                 7.144%  C  6 s(  0.04%)p99.99( 99.79%)d 3.98(  0.17%)
                 3.218% Zr  7 s( 20.06%)p 0.05(  1.01%)d 3.93( 78.93%)
```

Zr-Me interaction:

```
 60. (2.00000)  97.2862% BD ( 1)Zr  7- C 21
                20.650% Zr  7 s( 11.81%)p 0.00(  0.03%)d 7.47( 88.16%)
                76.650%  C 21 s( 26.51%)p 2.77( 73.48%)d 0.00(  0.01%)
```

Resonance structure **3**:

C-H interaction:

```
 50. (2.00000)  96.9202% BD ( 1) C  2- H 26
                60.784%  C  2 s( 26.13%)p 2.82( 73.79%)d 0.00(  0.08%)
                 1.699% Zr  7 s( 27.77%)p 0.02(  0.46%)d 2.58( 71.77%)
                36.178%  H 26 s( 99.97%)p 0.00(  0.03%)
```

C-C interaction:

```
 48. (2.00000)  98.0816% BD ( 1) C  2- C  3
                49.207%  C  2 s( 32.22%)p 2.10( 67.74%)d 0.00(  0.05%)
                49.026%  C  3 s( 29.22%)p 2.42( 70.74%)d 0.00(  0.04%)
 49. (2.00000)  83.5746% BD ( 2) C  2- C  3
                 4.252%  C  1 s(  0.03%)p99.99( 99.60%)d11.83(  0.37%)
                47.387%  C  2 s(  0.67%)p99.99( 99.30%)d 0.04(  0.03%)
                36.193%  C  3 s(  0.11%)p99.99( 99.84%)d 0.48(  0.05%)
                 5.116%  C  4 s(  0.02%)p99.99( 99.52%)d25.38(  0.46%)
                 1.727%  C  6 s(  0.06%)p99.99( 99.79%)d 2.38(  0.15%)
                 2.851% Zr  7 s( 22.19%)p 0.03(  0.73%)d 3.47( 77.08%)
```

Zr-Me interaction:

```
 60. (2.00000)  97.2862% BD ( 1)Zr  7- C 21
                20.650% Zr  7 s( 11.82%)p 0.00(  0.03%)d 7.46( 88.16%)
                76.649%  C 21 s( 26.51%)p 2.77( 73.49%)d 0.00(  0.01%)
```

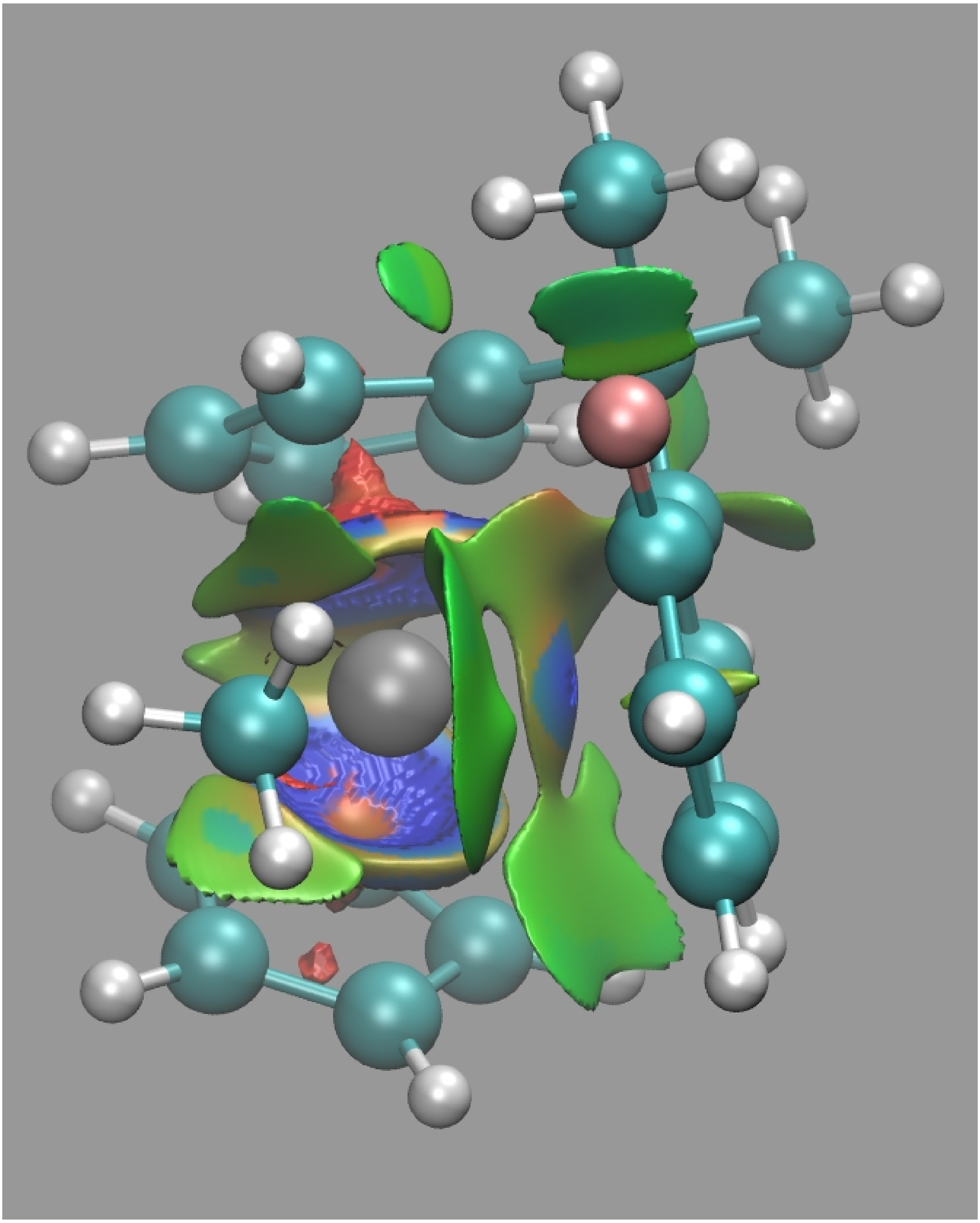

Non-Covalent Interactions (NCI)

2A-PBE-D3

Bader:

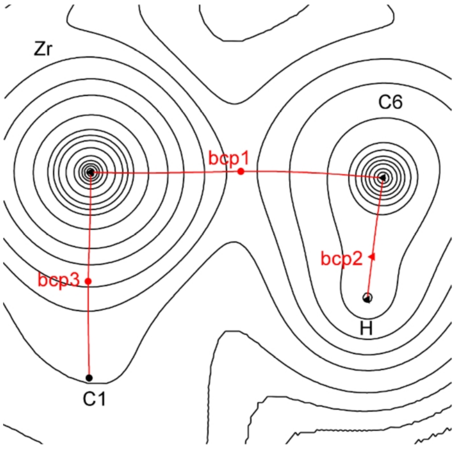

Electrondensity

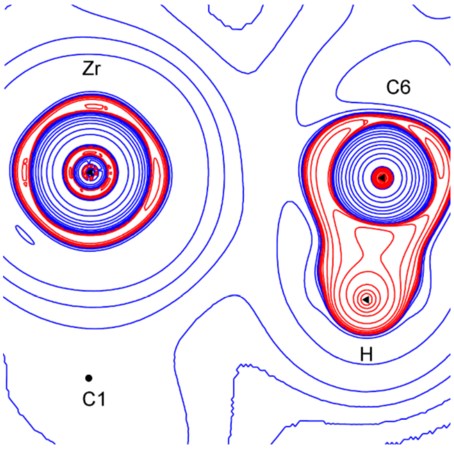

Laplacian

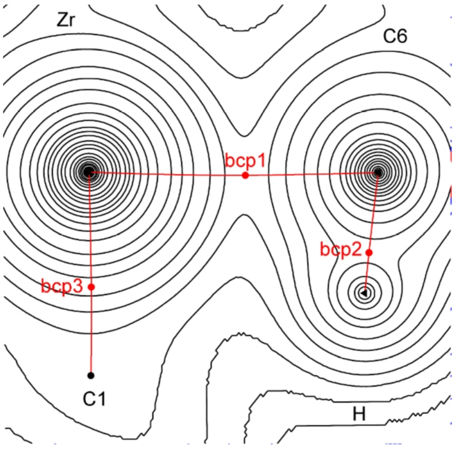

Virial

	$\varrho(\mathbf{r})$	$\nabla^2\varrho(\mathbf{r})$
bcp1	0.03494	-0.02119
bcp2	0.26861	0.21748
bcp3	0.09473	-0.01193

NBO:

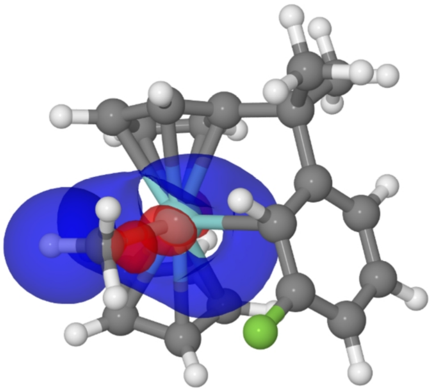

1

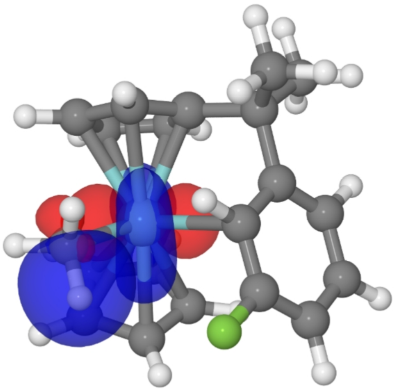

2

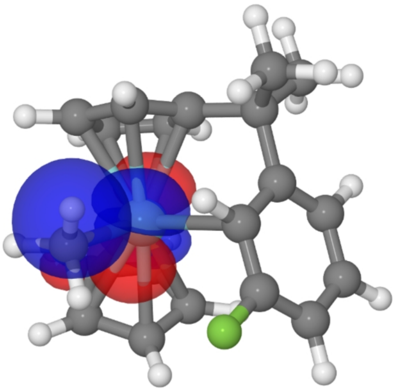

3

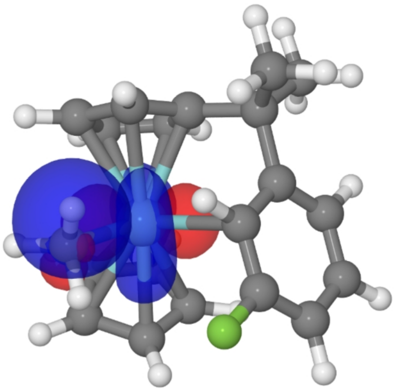

4

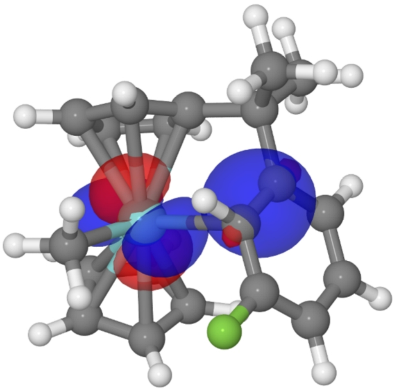

5

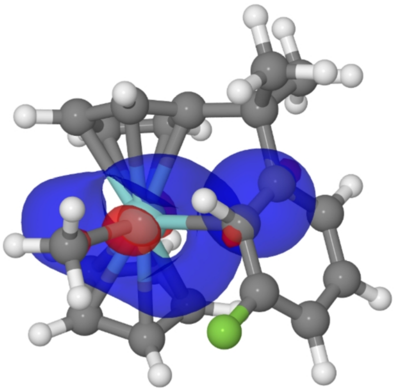

6

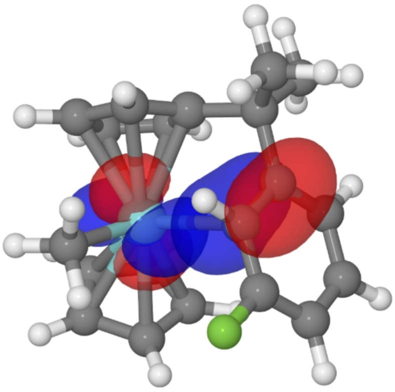

7

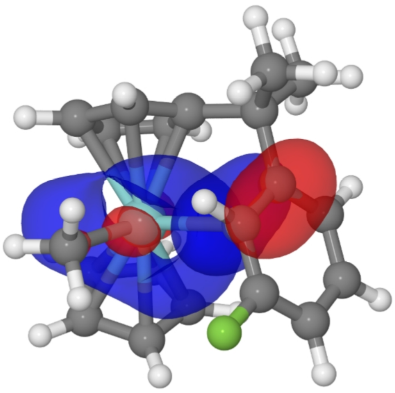

8

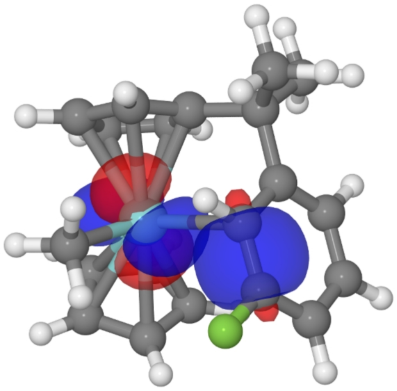

9

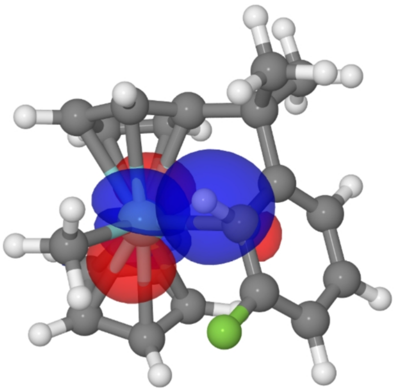

10

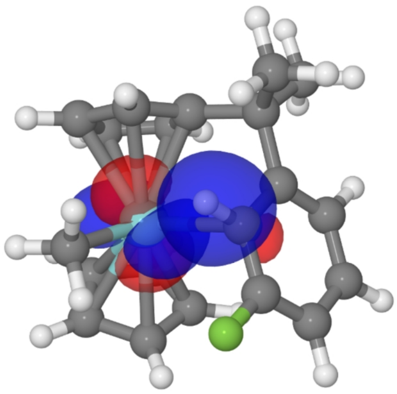

11

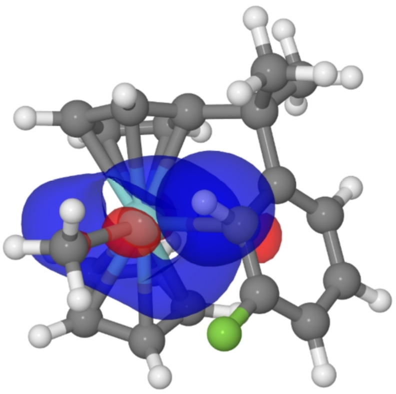

12

	Orbitals	E(2P)
1	σ_{CH} = 0.787$(sp^{3.19})_{C7}$ – 0.617$(s)_{H42}$ → LV_{Zr} = $sd^{0.31}$	2.15
2	σ_{CH} = 0.782$(sp^{3.00})_{C7}$ – 0.623$(s)_{H43}$ → LV_{Zr} = $sd^{11.27}$	3.37
3	σ_{CH} = 0.780$(sp^{2.99})_{C7}$ – 0.626$(s)_{H44}$ → LV_{Zr} = $sd^{99.99}$	2.11
4	σ_{CH} = 0.780$(sp^{2.99})_{C7}$ – 0.626$(s)_{H44}$ → LV_{Zr} = $sd^{11.27}$	2.66
5	σ_{CC} = 0.713$(sp^{1.72})_{C8}$ – 0.701$(sp^{2.10})_{C9}$ → LV_{Zr} = $sd^{64.45}$	3.11
6	σ_{CC} = 0.713$(sp^{1.72})_{C8}$ – 0.701$(sp^{2.10})_{C9}$ → LV_{Zr} = $sd^{0.31}$	3.90
7	π_{CC} = 0.763$(p)_{C8}$ – 0.646$(p)_{C9}$ → LV_{Zr} = $sd^{64.45}$	7.61
8	π_{CC} = 0.763$(p)_{C8}$ – 0.646$(p)_{C9}$ → LV_{Zr} = $sd^{0.31}$	5.56
9	σ_{CC} = 0.714$(sp^{1.96})_{C8}$ – 0.700$(sp^{1.58})_{C13}$ → LV_{Zr} = $sd^{64.45}$	3.27
10	σ_{CH} = 0.799$(sp^{2.45})_{C8}$ – 0.602$(s)_{H24}$ → LV_{Zr} = $sd^{99.99}$	2.40
11	σ_{CH} = 0.799$(sp^{2.45})_{C8}$ – 0.602$(s)_{H24}$ → LV_{Zr} = $sd^{64.45}$	3.88
12	σ_{CH} = 0.799$(sp^{2.45})_{C8}$ – 0.602$(s)_{H24}$ → LV_{Zr} = $sd^{0.31}$	3.80

Natural Resonance Theory:

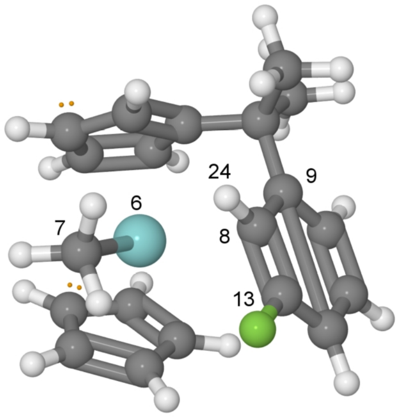

1

Wgt=19.15%;
rhoNL=5.84272;
D(0)=0.10125

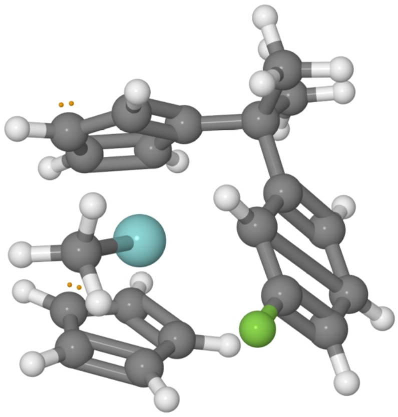

2

Wgt=14.88%;
rhoNL=5.80357;
D(0)=0.1009

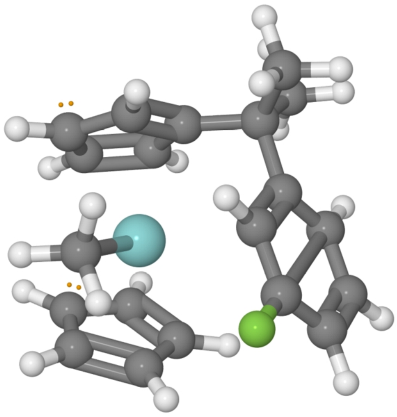

3

Wgt=13.81%;
rhoNL=5.85139;
D(0)=0.10133

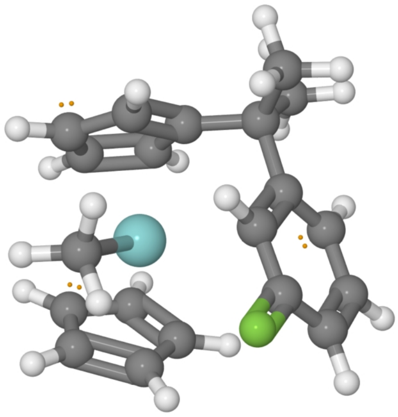

4

Wgt=5.61%;
rhoNL=6.05616;
D(0)=0.10309

Natural Localised Molecular Orbitals (NLMO):

Only contributions over 1% are reported.

NLMO / Occupancy / Percent from Parent NBO / Atomic Hybrid Contributions

Resonance structure **1**:
C-H interaction:

```
 64. (2.00000)  97.3486% BD ( 1) C  8- H 24
                  1.143% Zr  6 s( 31.06%)p 0.03(  0.79%)d 2.19( 68.15%)
                 62.184%  C  8 s( 27.26%)p 2.67( 72.66%)d 0.00(  0.07%)
                 35.199%  H 24 s( 99.98%)p 0.00(  0.02%)
```

C-C interaction:

```
 62. (2.00000)  98.8229% BD ( 1) C  8- C 13
                 50.493%  C  8 s( 30.66%)p 2.26( 69.26%)d 0.00(  0.08%)
                 48.426%  C 13 s( 36.98%)p 1.70( 63.00%)d 0.00(  0.02%)
 63. (2.00000)  83.3897% BD ( 2) C  8- C 13
                  2.731% Zr  6 s( 21.42%)p 0.04(  0.81%)d 3.63( 77.76%)
                 49.866%  C  8 s(  0.81%)p99.99( 99.16%)d 0.04(  0.03%)
                  5.306%  C  9 s(  0.23%)p99.99( 99.41%)d 1.53(  0.36%)
                  1.907%  C 10 s(  0.01%)p 1.00( 99.85%)d 0.00(  0.14%)
                  1.104%  C 11 s(  0.12%)p99.99( 99.52%)d 3.05(  0.36%)
                  4.829%  C 12 s(  0.01%)p 1.00( 99.63%)d 0.00(  0.37%)
                 33.547%  C 13 s(  0.02%)p99.99( 99.93%)d 2.67(  0.05%)
```

Zr-Me interaction:

```
 57. (2.00000)  97.0005% BD ( 1)Zr  6- C  7
                 20.569% Zr  6 s( 11.33%)p 0.00(  0.04%)d 7.82( 88.63%)
                 76.447%  C  7 s( 26.63%)p 2.76( 73.37%)d 0.00(  0.01%)
```

Resonance structure **2**:
C-H interaction:

```
 64. (2.00000)  97.3548% BD ( 1) C  8- H 24
                  1.174% Zr  6 s( 31.35%)p 0.03(  0.79%)d 2.16( 67.86%)
                 62.205%  C  8 s( 27.36%)p 2.65( 72.56%)d 0.00(  0.07%)
                 35.178%  H 24 s( 99.98%)p 0.00(  0.02%)
```

C-C interaction:

```
 62. (2.00000)  67.8982% BD ( 1) C  8- C 11
                  3.131% Zr  6 s( 19.36%)p 0.05(  0.94%)d 4.12( 79.70%)
                 44.665%  C  8 s(  0.79%)p99.99( 99.20%)d 0.02(  0.01%)
                  7.471%  C  9 s(  0.19%)p99.99( 99.53%)d 1.51(  0.29%)
                  6.845%  C 10 s(  0.02%)p99.99( 99.82%)d 7.83(  0.16%)
                 23.319%  C 11 s(  0.04%)p99.99( 99.95%)d 0.21(  0.01%)
                  6.125%  C 12 s(  0.07%)p99.99( 99.76%)d 2.66(  0.18%)
                  7.580%  C 13 s(  0.01%)p99.99( 99.67%)d25.25(  0.32%)
```

Zr-Me interaction:

```
 57. (2.00000)  97.0003% BD ( 1)Zr  6- C  7
                20.568% Zr  6 s( 11.33%)p 0.00(  0.04%)d 7.82( 88.63%)
                76.447%  C  7 s( 26.62%)p 2.76( 73.37%)d 0.00(  0.01%)
```

Resonance structure **3**:

C-H interaction:

```
 64. (2.00000)  97.3488% BD ( 1) C  8- H 24
                 1.145% Zr  6 s( 30.86%)p 0.03(  0.79%)d 2.21( 68.35%)
                62.188%  C  8 s( 27.26%)p 2.67( 72.67%)d 0.00(  0.07%)
                35.196%  H 24 s( 99.98%)p 0.00(  0.02%)
```

C-C interaction:

```
 61. (2.00000)  98.0673% BD ( 1) C  8- C  9
                50.019%  C  8 s( 32.71%)p 2.06( 67.25%)d 0.00(  0.05%)
                48.196%  C  9 s( 29.01%)p 2.45( 70.95%)d 0.00(  0.04%)
 62. (2.00000)  82.8688% BD ( 2) C  8- C  9
                 3.471% Zr  6 s( 22.07%)p 0.04(  0.86%)d 3.49( 77.07%)
                47.865%  C  8 s(  0.84%)p99.99( 99.12%)d 0.05(  0.04%)
                35.013%  C  9 s(  0.13%)p99.99( 99.82%)d 0.40(  0.05%)
                 4.462%  C 10 s(  0.01%)p 1.00( 99.66%)d 0.00(  0.33%)
                 1.131%  C 11 s(  0.11%)p99.99( 99.53%)d 3.16(  0.36%)
                 1.545%  C 12 s(  0.13%)p99.99( 99.69%)d 1.40(  0.18%)
                 5.393%  C 13 s(  0.03%)p99.99( 99.58%)d15.39(  0.39%)
```

Zr-Me interaction:

```
 57. (2.00000)  97.0017% BD ( 1)Zr  6- C  7
                20.563% Zr  6 s( 11.35%)p 0.00(  0.04%)d 7.81( 88.61%)
                76.453%  C  7 s( 26.62%)p 2.76( 73.37%)d 0.00(  0.01%)
```

Resonance structure **4**:

C-H interaction:

```
 64. (2.00000)  97.3496% BD ( 1) C  8- H 24
                 1.148% Zr  6 s( 30.78%)p 0.03(  0.79%)d 2.22( 68.44%)
                62.205%  C  8 s( 27.09%)p 2.69( 72.83%)d 0.00(  0.07%)
                35.185%  H 24 s( 99.98%)p 0.00(  0.02%)
```

C-C interaction:

```
 61. (2.00000)  98.0661% BD ( 1) C  8- C  9
                50.004%  C  8 s( 32.90%)p 2.04( 67.05%)d 0.00(  0.05%)
                48.202%  C  9 s( 29.00%)p 2.45( 70.96%)d 0.00(  0.04%)
 62. (2.00000)  83.1691% BD ( 2) C  8- C  9
                 3.581% Zr  6 s( 21.28%)p 0.04(  0.87%)d 3.66( 77.85%)
                49.757%  C  8 s(  0.84%)p99.99( 99.12%)d 0.05(  0.04%)
                33.446%  C  9 s(  0.13%)p99.99( 99.82%)d 0.43(  0.06%)
                 3.498%  C 10 s(  0.01%)p 1.00( 99.59%)d 0.00(  0.40%)
                 1.198%  C 11 s(  0.12%)p99.99( 99.60%)d 2.23(  0.28%)
                 1.144%  C 12 s(  0.17%)p99.99( 99.53%)d 1.70(  0.29%)
                 5.225%  C 13 s(  0.04%)p99.99( 99.46%)d12.45(  0.50%)
                 1.036%  F 16 s(  0.54%)p99.99( 99.45%)d 0.02(  0.01%)
```

Zr-Me interaction:

```
 57. (2.00000)  97.0013% BD ( 1)Zr  6- C  7
                20.564% Zr  6 s( 11.34%)p 0.00(  0.04%)d 7.82( 88.62%)
                76.452%  C  7 s( 26.62%)p 2.76( 73.37%)d 0.00(  0.01%)
```

Non-Covalent Interactions (NCI)

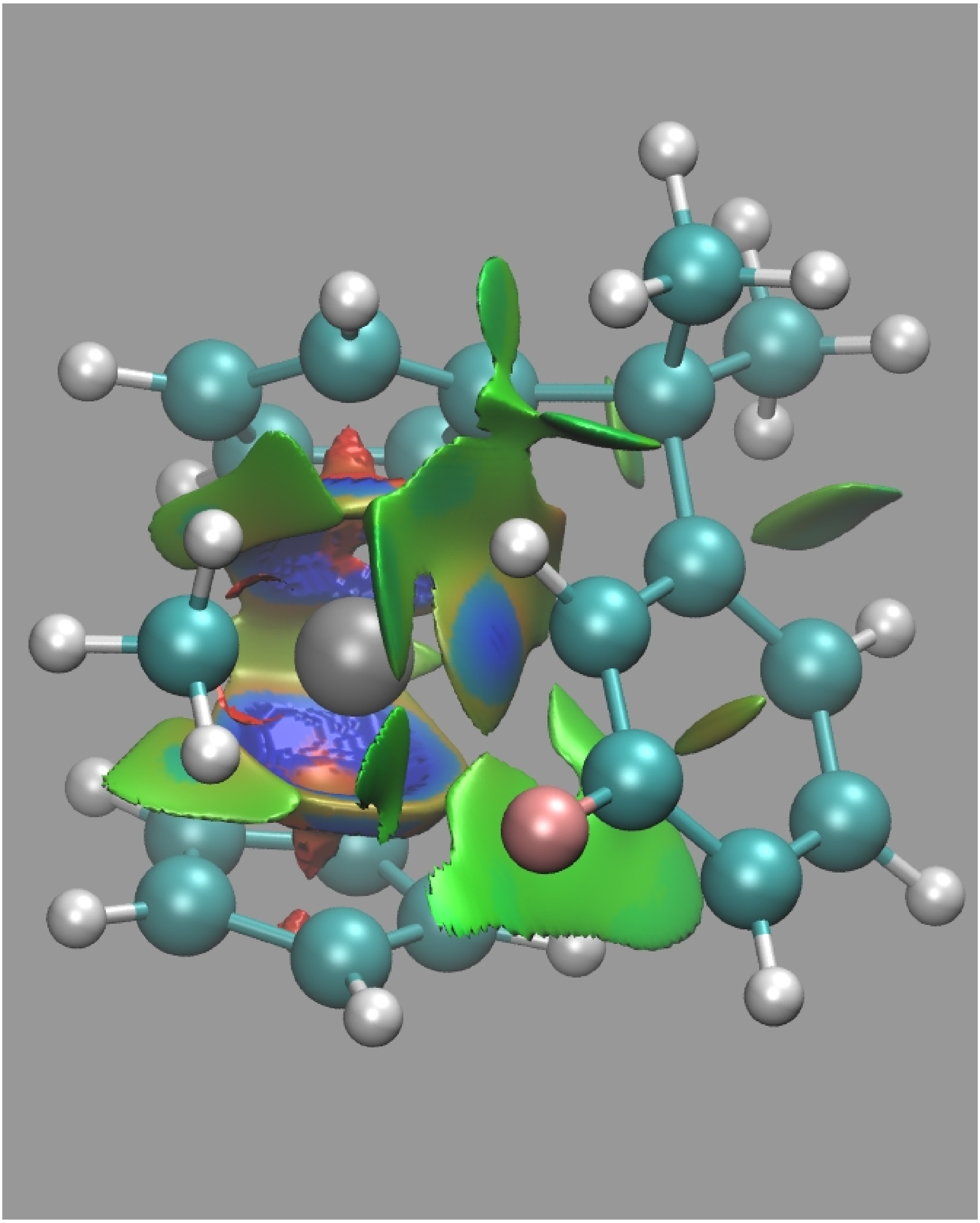

2B-PBE-D3

Bader:

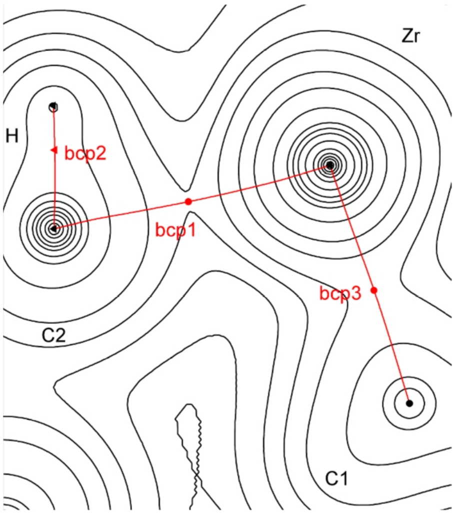

Electrondensity

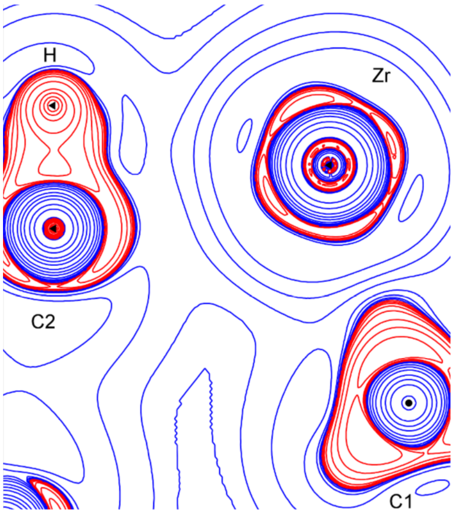

Laplacian

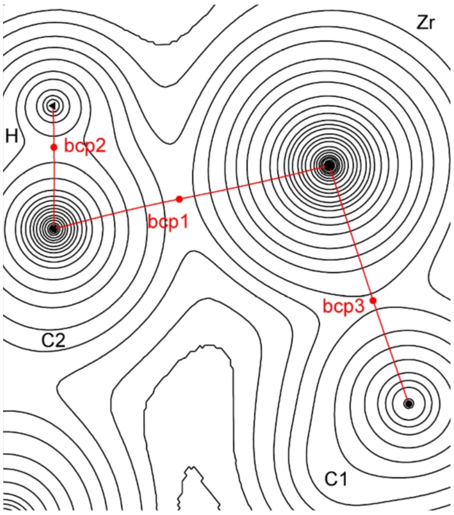

Virial

	ϱ(**r**)	∇^2ϱ(**r**)
bcp1	0.04060	-0.02351
bcp2	0.26086	0.20357
bcp3	0.09576	-0.01216

NBO:

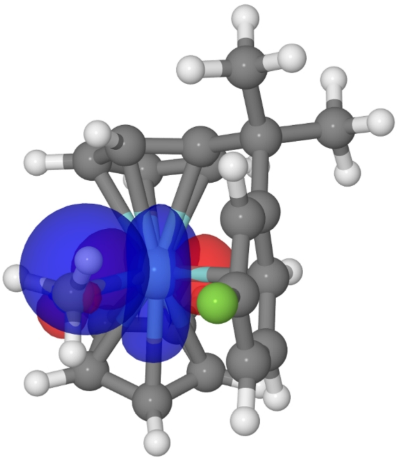

1

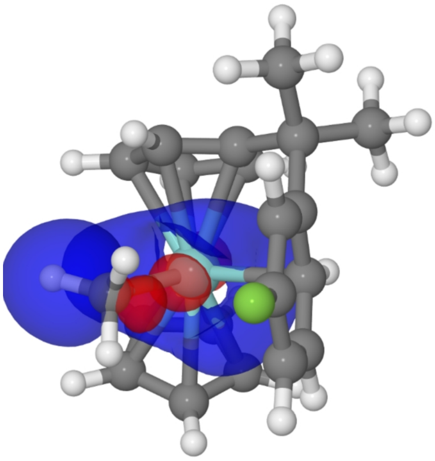

2

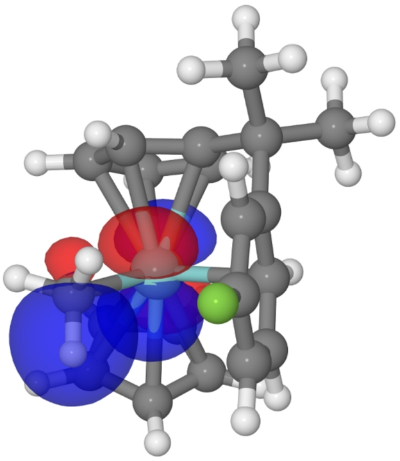

3

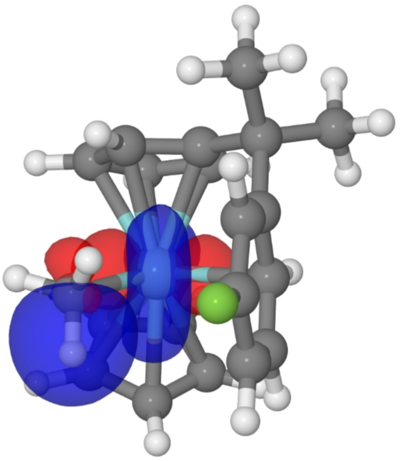

4

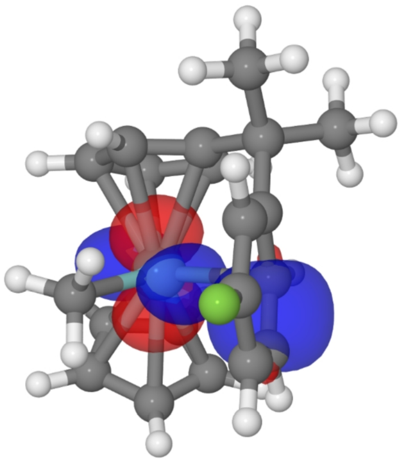

5

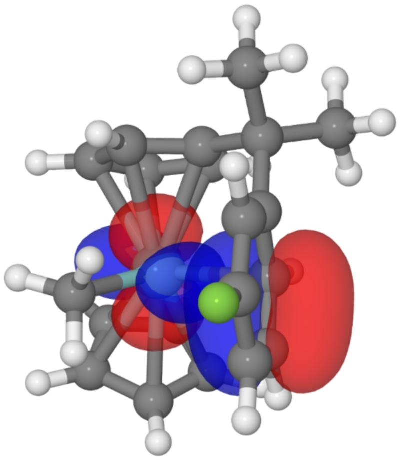

6

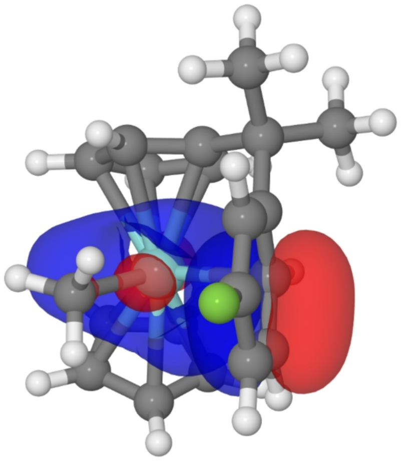

7

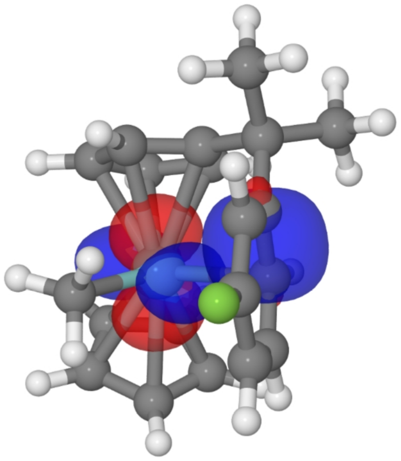

8

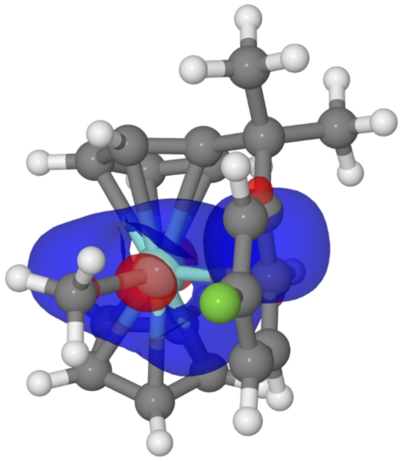

9

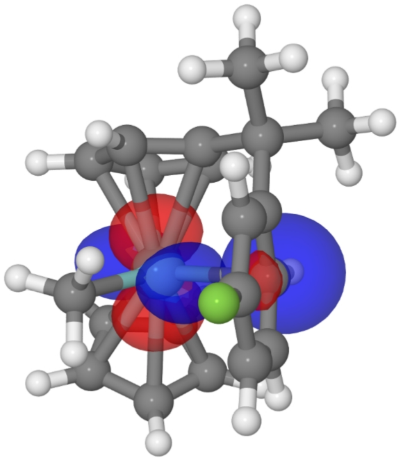

10

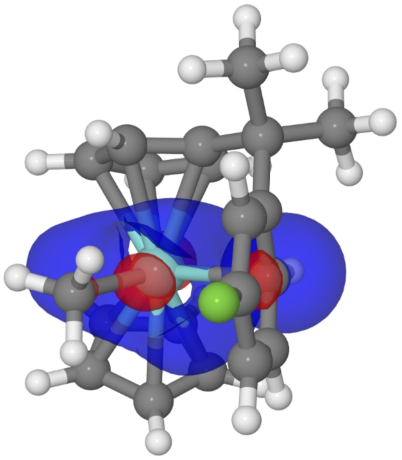

11

	Orbitals	E(2P)
1	$\sigma_{CH} = 0.780(sp^{3.01})_{C7} – 0.626(s)_{H37} \rightarrow$ $LV_{Zr} = sd^{7.93}$	4.00
2	$\sigma_{CH} = 0.787(sp^{3.17})_{C7} – 0.617(s)_{H38} \rightarrow$ $LV_{Zr} = sd^{0.36}$	2.17
3	$\sigma_{CH} = 0.781(sp^{2.89})_{C7} – 0.624(s)_{H39} \rightarrow$ $LV_{Zr} = sp^{0.22}d^{99.99}$	2.49
4	$\sigma_{CH} = 0.781(sp^{2.89})_{C7} – 0.624(s)_{H39} \rightarrow$ $LV_{Zr} = sd^{7.93}$	2.68
5	$\sigma_{CC} = 0.714(sp^{1.83})_{C8} – 0.700(sp^{1.77})_{C9} \rightarrow$ $LV_{Zr} = sd^{68.98}$	3.77
6	$\pi_{CC} = 0.775(p)_{C8} – 0.633(p)_{C9} \rightarrow$ $LV_{Zr} = sd^{0.36}$	9.67
7	$\pi_{CC} = 0.775(p)_{C8} – 0.633(p)_{C9} \rightarrow$ $LV_{Zr} = sd^{0.36}$	5.39
8	$\sigma_{CC} = 0.711(sp^{1.76})_{C8} – 0.703(s)_{C13} \rightarrow$ $LV_{Zr} = sd^{0.36}$	2.98
9	$\sigma_{CC} = 0.711(sp^{1.76})_{C8} – 0.703(s)_{C13} \rightarrow$ $LV_{Zr} = sd^{0.36}$	3.33
10	$\sigma_{CH} = 0.791(sp^{2.61})_{C8} – 0.612(s)_{H26} \rightarrow$ $LV_{Zr} = sd^{0.36}$	3.45
11	$\sigma_{CH} = 0.791(sp^{2.61})_{C8} – 0.612(s)_{H26} \rightarrow$ $LV_{Zr} = sd^{0.36}$	7.99

Natural Resonance Theory:

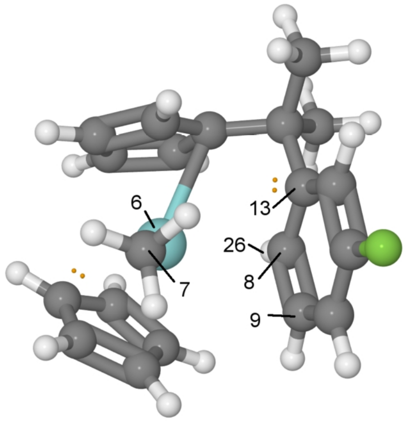

1

Wgt=14.20%;
rhoNL=5.99793;
D(0)=0.10259

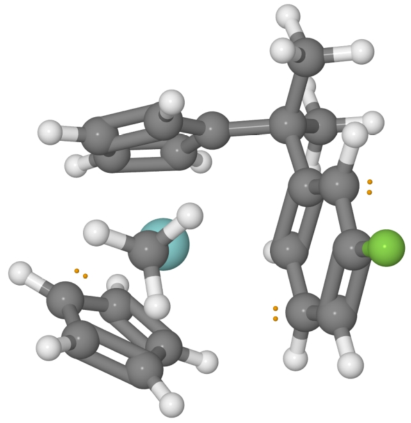

2

Wgt=13.77%;
rhoNL=6.25889;
D(0)=0.10480

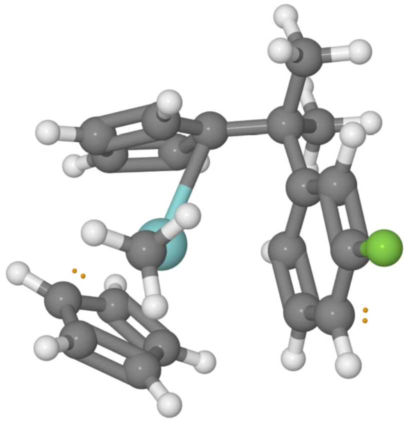

3

Wgt=10.74%;
rhoNL=5.92015;
D(0)=0.10192

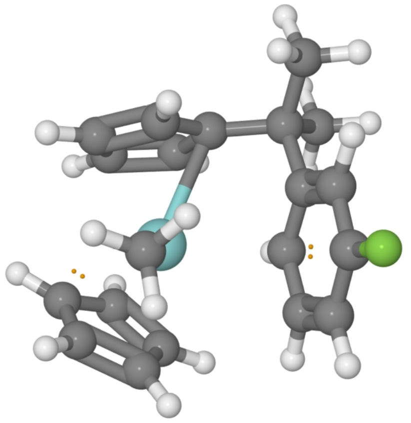

4

Wgt=9.07%;
rhoNL=5.71287;
D(0)=0.10012

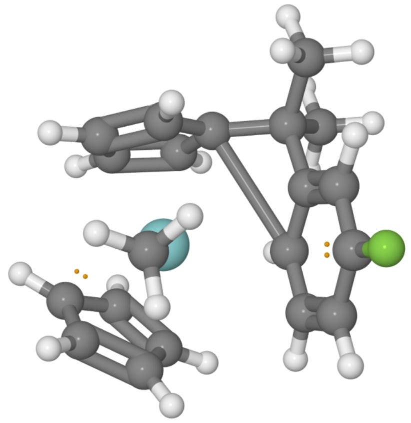

5

Wgt=8.74%;
rhoNL=6.01459;
D(0)=0.10273

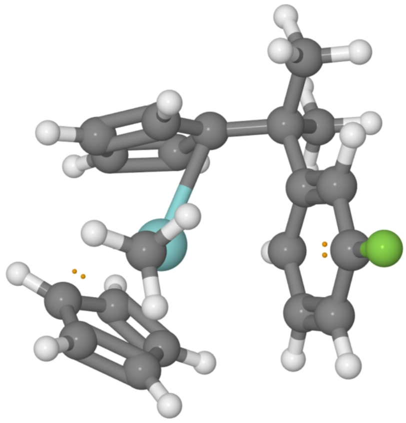

6

Wgt=7.72%;
rhoNL=5.97598;
D(0)=0.10240

Natural Localised Molecular Orbitals (NLMO):

Only contributions over 1% are reported.

NLMO / Occupancy / Percent from Parent NBO / Atomic Hybrid Contributions

Resonance structure **1**:
C-H interaction:

```
  65. (2.00000)  96.6366% BD ( 1) C  8- H 26
                   1.945% Zr  6 s( 26.45%)p 0.02(  0.48%)d 2.76( 73.07%)
                  60.479%  C  8 s( 26.16%)p 2.82( 73.75%)d 0.00(  0.09%)
                  36.194%  H 26 s( 99.97%)p 0.00(  0.03%)
```

C-C interaction:

```
  62. (2.00000)  98.7335% BD ( 1) C  8- C  9
                  50.445%  C  8 s( 31.84%)p 2.14( 68.11%)d 0.00(  0.05%)
                  48.411%  C  9 s( 33.01%)p 2.03( 66.95%)d 0.00(  0.04%)
  63. (2.00000)  84.5630% BD ( 2) C  8- C  9
                   3.131% Zr  6 s( 24.17%)p 0.03(  0.74%)d 3.11( 75.10%)
                  50.065%  C  8 s(  1.11%)p89.47( 98.88%)d 0.02(  0.02%)
                  34.519%  C  9 s(  0.02%)p99.99( 99.94%)d 1.64(  0.04%)
                   4.858%  C 10 s(  0.01%)p 1.00( 99.71%)d 0.00(  0.29%)
                   1.069%  C 12 s(  0.21%)p99.99( 99.47%)d 1.49(  0.32%)
                   5.109%  C 13 s(  0.21%)p99.99( 99.49%)d 1.45(  0.30%)
```

Zr-Me interaction:

```
  57. (2.00000)  97.3454% BD ( 1)Zr  6- C  7
                  20.903% Zr  6 s( 11.92%)p 0.00(  0.04%)d 7.39( 88.04%)
                  76.456%  C  7 s( 26.40%)p 2.79( 73.59%)d 0.00(  0.01%)
```

Resonance structure **2**:
C-H interaction:

```
  65. (2.00000)  96.6219% BD ( 1) C  8- H 26
                   2.018% Zr  6 s( 26.02%)p 0.02(  0.46%)d 2.82( 73.51%)
                  60.554%  C  8 s( 26.30%)p 2.80( 73.61%)d 0.00(  0.09%)
                  36.121%  H 26 s( 99.97%)p 0.00(  0.03%)
```

C-C interaction:

```
 63. (2.00000)  98.4036% BD ( 1) C  8- C 13
                  49.818%  C  8 s( 32.34%)p 2.09( 67.61%)d 0.00(  0.05%)
                  48.736%  C 13 s( 29.40%)p 2.40( 70.56%)d 0.00(  0.04%)
  64. (2.00000)  82.5274% BD ( 2) C  8- C 13
                   2.857% Zr  6 s( 23.27%)p 0.04(  0.85%)d 3.26( 75.88%)
                  50.972%  C  8 s(  0.94%)p99.99( 99.04%)d 0.02(  0.02%)
                   5.946%  C  9 s(  0.04%)p99.99( 99.69%)d 7.40(  0.27%)
                   1.529%  C 10 s(  0.11%)p99.99( 99.66%)d 2.01(  0.23%)
                   1.023%  C 11 s(  0.06%)p99.99( 99.56%)d 5.88(  0.38%)
                   3.789%  C 12 s(  0.01%)p 1.00( 99.62%)d 0.00(  0.37%)
                  31.569%  C 13 s(  0.15%)p99.99( 99.80%)d 0.30(  0.05%)
                   1.444%  C 18 s(  0.98%)p99.99( 98.79%)d 0.23(  0.22%)
```

Zr-Me interaction:
58. (2.00000) 97.2402% BD (1)Zr 6- C 7
20.949% Zr 6 s(12.03%)p 0.00(0.04%)d 7.31(87.94%)
76.316% C 7 s(26.43%)p 2.78(73.57%)d 0.00(0.01%)

Resonance structure **3**:
C-H interaction:
65. (2.00000) 96.6412% BD (1) C 8- H 26
1.939% Zr 6 s(26.55%)p 0.02(0.48%)d 2.75(72.97%)
60.473% C 8 s(26.16%)p 2.82(73.76%)d 0.00(0.09%)
36.203% H 26 s(99.97%)p 0.00(0.03%)

C-C interaction:
62. (2.00000) 98.7335% BD (1) C 8- C 9
50.446% C 8 s(31.84%)p 2.14(68.11%)d 0.00(0.05%)
48.410% C 9 s(33.01%)p 2.03(66.95%)d 0.00(0.04%)
63. (2.00000) 84.6000% BD (2) C 8- C 9
3.275% Zr 6 s(23.88%)p 0.03(0.75%)d 3.16(75.37%)
51.600% C 8 s(1.11%)p89.35(98.88%)d 0.02(0.02%)
33.030% C 9 s(0.02%)p99.99(99.94%)d 1.72(0.04%)
3.594% C 10 s(0.01%)p99.99(99.62%)d35.08(0.37%)
1.027% C 11 s(0.11%)p99.99(99.52%)d 3.22(0.37%)
1.342% C 12 s(0.18%)p99.99(99.55%)d 1.50(0.27%)
5.376% C 13 s(0.21%)p99.99(99.49%)d 1.44(0.30%)

Zr-Me interaction:
57. (2.00000) 97.3456% BD (1)Zr 6- C 7
20.905% Zr 6 s(11.92%)p 0.00(0.04%)d 7.39(88.04%)
76.453% C 7 s(26.40%)p 2.79(73.59%)d 0.00(0.01%)

Resonance structure **4**:
C-H interaction:
64. (2.00000) 96.6522% BD (1) C 8- H 26
2.007% Zr 6 s(27.12%)p 0.02(0.47%)d 2.67(72.42%)
60.530% C 8 s(26.31%)p 2.80(73.61%)d 0.00(0.09%)
36.144% H 26 s(99.97%)p 0.00(0.03%)

C-C interaction:
62. (2.00000) 98.7257% BD (1) C 8- C 9
50.447% C 8 s(31.87%)p 2.14(68.08%)d 0.00(0.05%)
48.406% C 9 s(33.02%)p 2.03(66.94%)d 0.00(0.04%)
63. (2.00000) 98.3931% BD (1) C 8- C 13
49.831% C 8 s(32.42%)p 2.08(67.54%)d 0.00(0.05%)
48.715% C 13 s(29.43%)p 2.40(70.53%)d 0.00(0.04%)

Zr-Me interaction:
57. (2.00000) 97.3456% BD (1)Zr 6- C 7
20.903% Zr 6 s(11.92%)p 0.00(0.04%)d 7.39(88.04%)
76.456% C 7 s(26.40%)p 2.79(73.59%)d 0.00(0.01%)

Resonance structure **5**:
C-H interaction:

```
 64. (2.00000)  96.6651% BD ( 1) C  8- H 26
                 2.001% Zr  6 s( 26.31%)p 0.02(  0.47%)d 2.78( 73.22%)
                60.539%  C  8 s( 26.32%)p 2.80( 73.59%)d 0.00(  0.09%)
                36.147%  H 26 s( 99.97%)p 0.00(  0.03%)
```

C-C interaction:

```
 61. (2.00000)  98.7249% BD ( 1) C  8- C  9
                50.436%  C  8 s( 31.90%)p 2.13( 68.05%)d 0.00(  0.05%)
                48.414%  C  9 s( 33.05%)p 2.02( 66.91%)d 0.00(  0.04%)
 62. (2.00000)  98.3928% BD ( 1) C  8- C 13
                49.825%  C  8 s( 32.37%)p 2.09( 67.58%)d 0.00(  0.05%)
                48.724%  C 13 s( 29.43%)p 2.40( 70.54%)d 0.00(  0.04%)
 63. (2.00000)  55.9391% BD ( 1) C  8- C 18
                 3.312% Zr  6 s( 25.30%)p 0.04(  1.02%)d 2.91( 73.68%)
                56.660%  C  8 s(  0.90%)p99.99( 99.09%)d 0.01(  0.01%)
                12.510%  C  9 s(  0.05%)p99.99( 99.82%)d 2.98(  0.14%)
                 1.386%  C 10 s(  0.14%)p99.99( 99.24%)d 4.32(  0.62%)
                 7.581%  C 11 s(  0.02%)p99.99( 99.98%)d 0.13(  0.00%)
                14.593%  C 13 s(  0.18%)p99.99( 99.70%)d 0.70(  0.12%)
                 2.138%  C 18 s(  0.74%)p99.99( 99.17%)d 0.13(  0.10%)
```

Zr-Me interaction:

```
 57. (2.00000)  97.3437% BD ( 1)Zr  6- C  7
                20.950% Zr  6 s( 11.96%)p 0.00(  0.04%)d 7.36( 88.01%)
                76.407%  C  7 s( 26.40%)p 2.79( 73.59%)d 0.00(  0.01%)
```

Resonance structure **6**:
NLMO algorithm failed to converge

Non-Covalent Interactions (NCI)

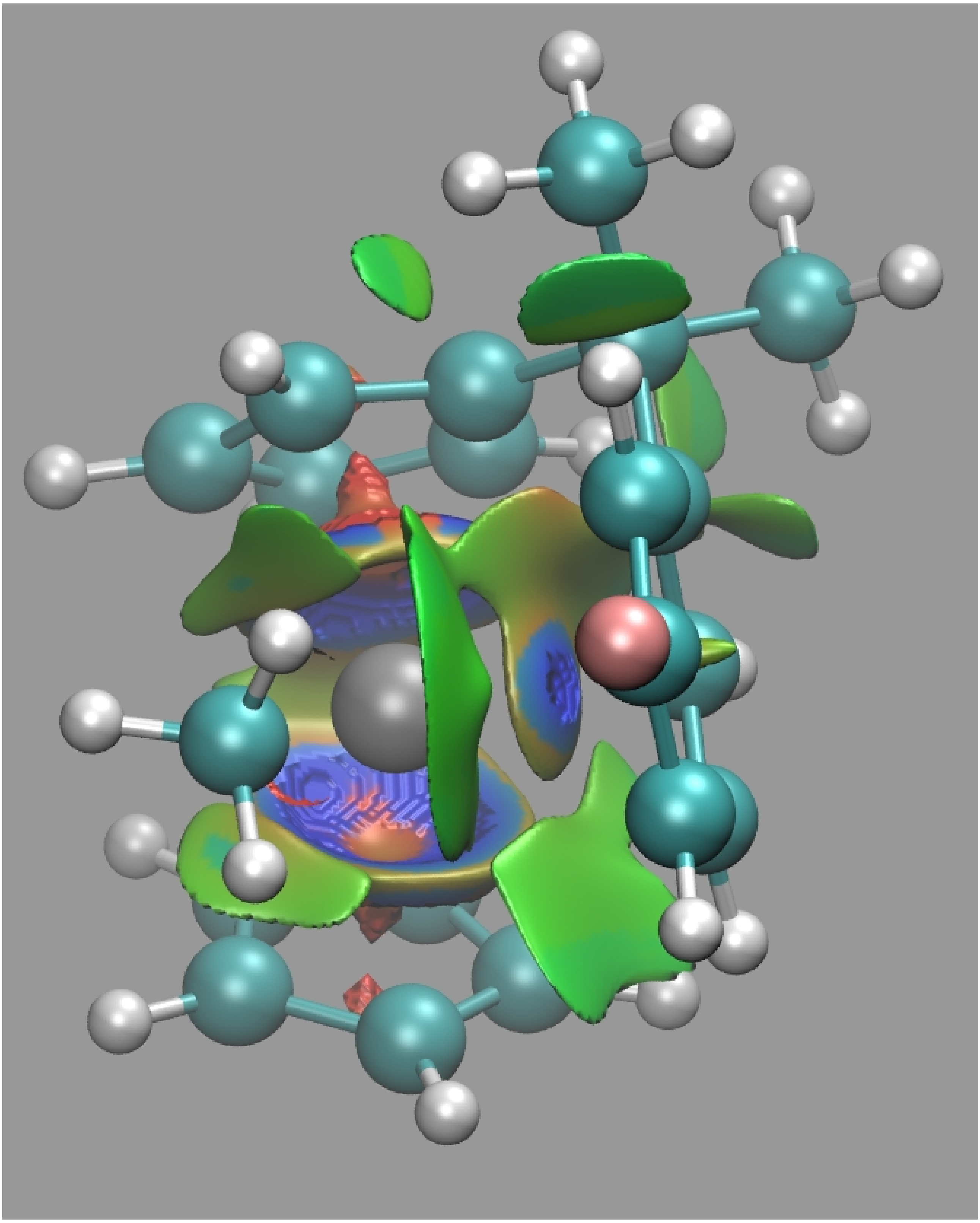

3A-PBE-D3

Bader:

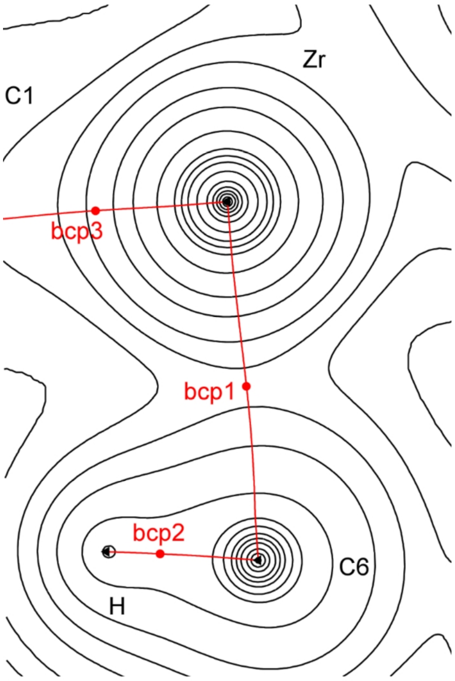

Electrondensity

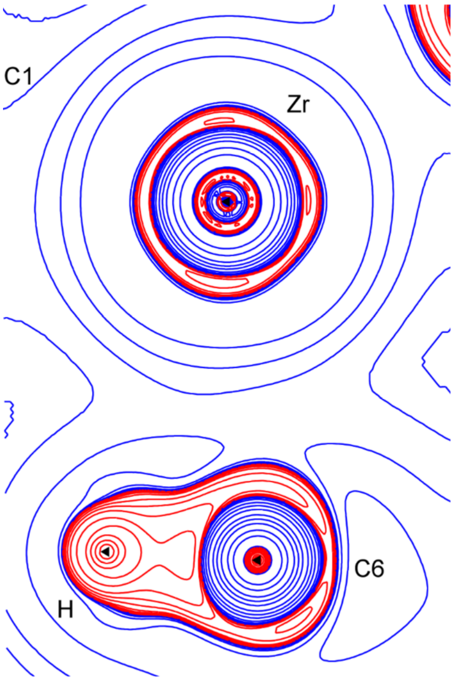

Laplacian

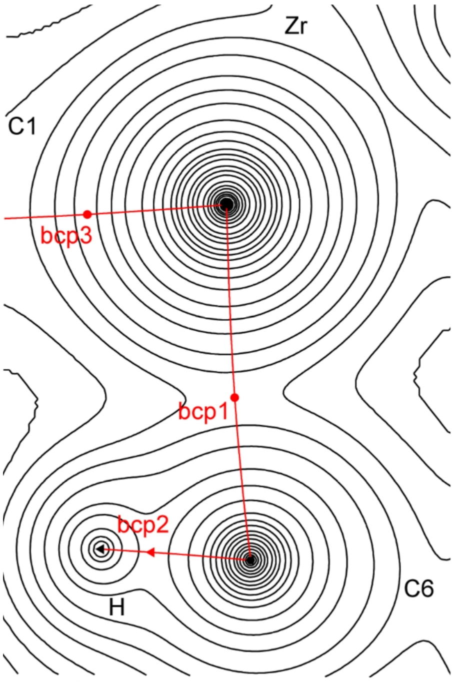

Virial

	$\varrho(\mathbf{r})$	$\nabla^2\varrho(\mathbf{r})$
bcp1	0.03453	-0.02165
bcp2	0.26935	0.21824
bcp3	0.09387	-0.01235

NBO:

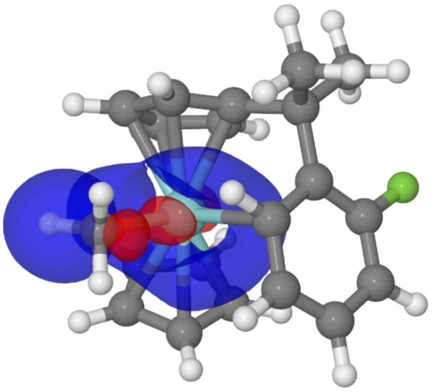

1

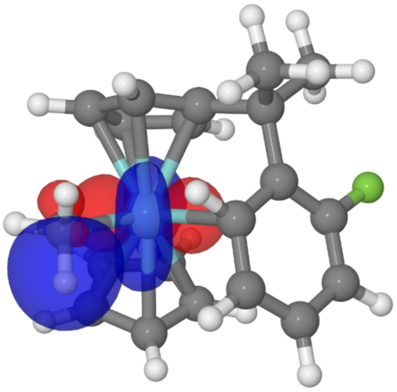

2

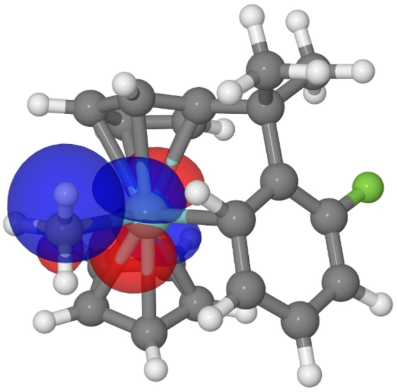

3

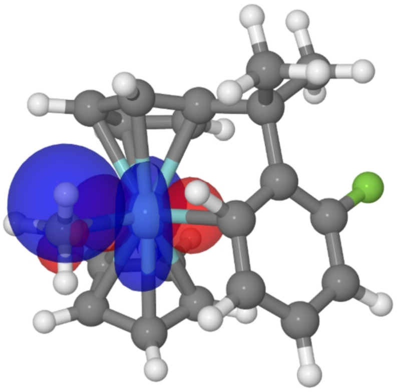

4

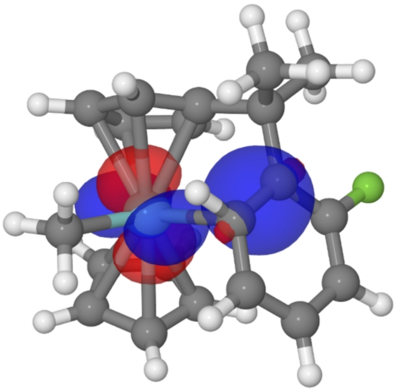

5

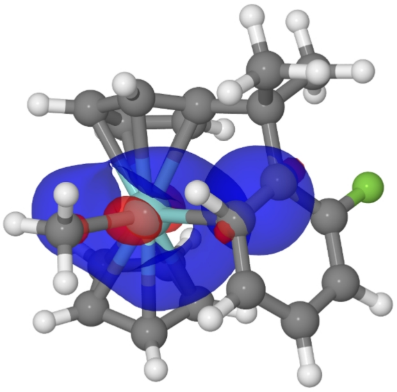

6

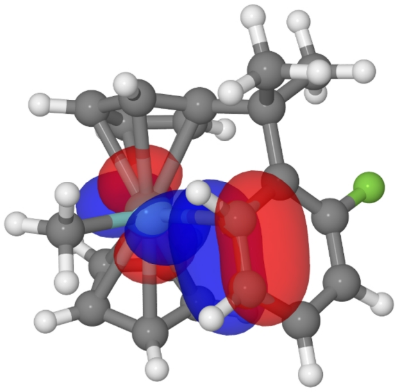

7

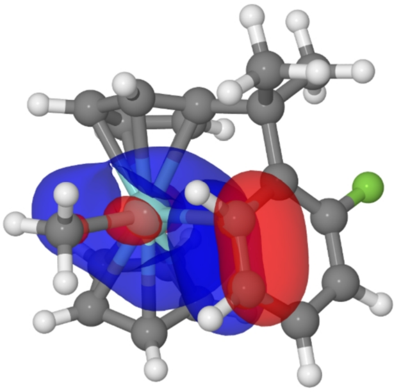

8

	Orbitals	E(2P)
1	σ_{CH} = 0.787$(sp^{3.24})_{C7}$ – 0.6168$(s)_{H42}$ → LV_{Zr} = $sd^{0.27}$	2.11
2	σ_{CH} = 0.781$(sp^{3.00})_{C7}$ – 0.625$(s)_{H43}$ → LV_{Zr} = $sd^{16.27}$	2.59
3	σ_{CH} = 0.780$(sp^{3.01})_{C7}$ – 0.6261$(s)_{H44}$ → LV_{Zr} = $sp^{0.34}d^{99.99}$	2.13
4	σ_{CH} = 0.780$(sp^{3.01})_{C7}$ – 0.6261$(s)_{H44}$ → LV_{Zr} = $sd^{16.27}$	2.51
5	σ_{CC} = 0.708$(sp^{2.03})_{C15}$ – 0.706$(s)_{C20}$ → LV_{Zr} = $sd^{60.36}$	2.99
6	σ_{CC} = 0.708$(sp^{2.03})_{C15}$ – 0.706$(s)_{C20}$ → LV_{Zr} = $sd^{0.27}$	3.55
7	π_{CH} = 0.663$(p)_{C19}$ – 0.749$(p)_{C20}$ → LV_{Zr} = $sd^{60.36}$	7.54
8	π_{CH} = 0.663$(p)_{C19}$ – 0.749$(p)_{C20}$ → LV_{Zr} = $sd^{0.27}$	4.16

Natural Resonance Theory:

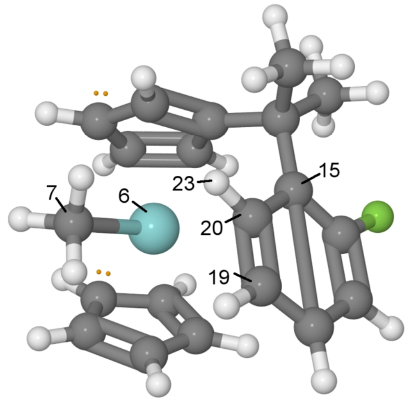

1

Wgt=20.21%;
rhoNL=5.80258;
D(0)=0.10090

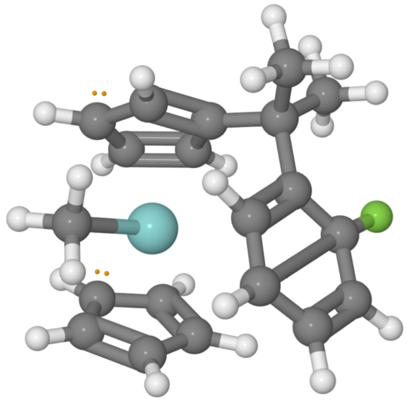

2

Wgt=15.89%;
rhoNL=5.80934;
D(0)=0.10096

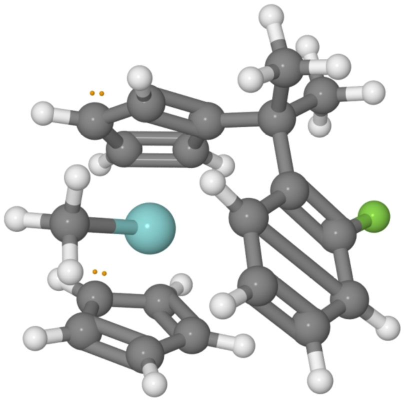

3

Wgt=14.98%;
rhoNL=5.79946;
D(0)=0.10088

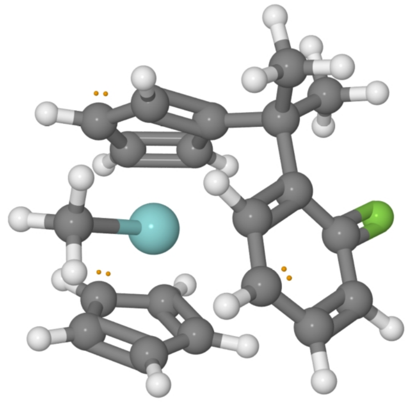

4

Wgt=7.75%;
rhoNL=6.02802;
D(0)=0.10285

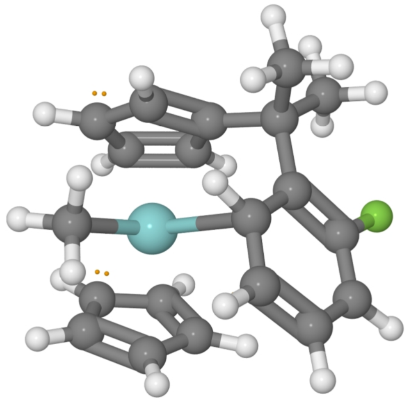

5

Wgt=5.49%;
rhoNL=5.94397;
D(0)=0.10213

Natural Localised Molecular Orbitals (NLMO):

Only contributions over 1% are reported.

NLMO / Occupancy / Percent from Parent NBO / Atomic Hybrid Contributions

Resonance structure **1**:
C-H interaction:

```
 92. (2.00000)  97.4129% BD ( 1) C 20- H 23
                 1.130% Zr  6 s( 30.35%)p 0.03(  0.77%)d 2.27( 68.88%)
                61.596%  C 20 s( 26.45%)p 2.78( 73.47%)d 0.00(  0.08%)
                35.844%  H 23 s( 99.98%)p 0.00(  0.02%)
```

C-C interaction:

```
 89. (2.00000)  98.7594% BD ( 1) C 19- C 20
                48.331%  C 19 s( 32.68%)p 2.06( 67.28%)d 0.00(  0.05%)
                50.545%  C 20 s( 32.79%)p 2.05( 67.17%)d 0.00(  0.05%)
 90. (2.00000)  84.0053% BD ( 2) C 19- C 20
                 2.736% Zr  6 s( 25.04%)p 0.03(  0.80%)d 2.96( 74.15%)
                 4.448%  C 15 s(  0.19%)p99.99( 99.42%)d 2.08(  0.39%)
                 1.682%  C 16 s(  0.05%)p99.99( 99.76%)d 4.39(  0.20%)
                 1.204%  C 17 s(  0.07%)p99.99( 99.56%)d 5.39(  0.37%)
                 5.235%  C 18 s(  0.01%)p99.99( 99.68%)d25.09(  0.31%)
                36.555%  C 19 s(  0.03%)p99.99( 99.93%)d 1.34(  0.04%)
                47.468%  C 20 s(  0.61%)p99.99( 99.36%)d 0.04(  0.03%)
```

Zr-Me interaction:

```
 57. (2.00000)  97.1661% BD ( 1)Zr  6- C  7
                20.143% Zr  6 s( 11.96%)p 0.00(  0.04%)d 7.36( 88.00%)
                77.034%  C  7 s( 26.90%)p 2.72( 73.09%)d 0.00(  0.01%)
```

Resonance structure **2**:
C-H interaction:

```
 92. (2.00000)  97.4086% BD ( 1) C 20- H 23
                 1.123% Zr  6 s( 30.20%)p 0.03(  0.77%)d 2.29( 69.03%)
                61.590%  C 20 s( 26.43%)p 2.78( 73.49%)d 0.00(  0.08%)
                35.849%  H 23 s( 99.98%)p 0.00(  0.02%)
```

C-C interaction:

```
 80. (2.00000)  98.0249% BD ( 1) C 15- C 20
                49.263%  C 15 s( 29.34%)p 2.41( 70.62%)d 0.00(  0.04%)
                48.910%  C 20 s( 31.87%)p 2.14( 68.08%)d 0.00(  0.05%)
 81. (2.00000)  82.6158% BD ( 2) C 15- C 20
                 3.485% Zr  6 s( 21.90%)p 0.04(  0.86%)d 3.53( 77.24%)
                36.382%  C 15 s(  0.11%)p99.99( 99.83%)d 0.50(  0.05%)
                 4.967%  C 16 s(  0.00%)p 1.00( 99.61%)d 0.00(  0.39%)
                 1.171%  C 17 s(  0.10%)p99.99( 99.59%)d 3.10(  0.31%)
                 1.515%  C 18 s(  0.15%)p99.99( 99.62%)d 1.53(  0.23%)
                 5.059%  C 19 s(  0.03%)p99.99( 99.66%)d 8.99(  0.30%)
                46.260%  C 20 s(  0.66%)p99.99( 99.31%)d 0.06(  0.04%)
```

Zr-Me interaction:
57. (2.00000) 97.1671% BD (1)Zr 6- C 7
20.138% Zr 6 s(11.97%)p 0.00(0.04%)d 7.35(87.99%)
77.041% C 7 s(26.90%)p 2.72(73.09%)d 0.00(0.01%)

Resonance structure **3**:
C-H interaction:
92. (2.00000) 97.4140% BD (1) C 20- H 23
1.159% Zr 6 s(30.56%)p 0.02(0.76%)d 2.25(68.68%)
61.611% C 20 s(26.51%)p 2.77(73.41%)d 0.00(0.08%)
35.826% H 23 s(99.98%)p 0.00(0.02%)

C-C interaction:
81. (2.00000) 98.0115% BD (1) C 15- C 20
49.255% C 15 s(29.35%)p 2.41(70.60%)d 0.00(0.04%)
48.914% C 20 s(31.90%)p 2.13(68.05%)d 0.00(0.05%)
85. (2.00000) 67.3327% BD (1) C 17- C 20
3.010% Zr 6 s(21.15%)p 0.05(1.01%)d 3.68(77.84%)
6.801% C 15 s(0.16%)p99.99(99.56%)d 1.74(0.28%)
7.193% C 16 s(0.04%)p99.99(99.72%)d 6.99(0.25%)
26.697% C 17 s(0.03%)p99.99(99.95%)d 0.57(0.02%)
7.167% C 18 s(0.07%)p99.99(99.74%)d 2.60(0.19%)
7.602% C 19 s(0.03%)p99.99(99.74%)d 7.08(0.23%)
40.681% C 20 s(0.61%)p99.99(99.38%)d 0.02(0.01%)
90. (2.00000) 98.7532% BD (1) C 19- C 20
48.327% C 19 s(32.68%)p 2.06(67.27%)d 0.00(0.05%)
50.548% C 20 s(32.81%)p 2.05(67.14%)d 0.00(0.05%)

Zr-Me interaction:
57. (2.00000) 97.1661% BD (1)Zr 6- C 7
20.141% Zr 6 s(11.96%)p 0.00(0.04%)d 7.36(88.00%)
77.036% C 7 s(26.90%)p 2.72(73.10%)d 0.00(0.01%)

Resonance structure **4**:
C-H interaction:
92. (2.00000) 97.4079% BD (1) C 20- H 23
1.125% Zr 6 s(30.09%)p 0.03(0.77%)d 2.30(69.14%)
61.591% C 20 s(26.43%)p 2.78(73.48%)d 0.00(0.08%)
35.847% H 23 s(99.98%)p 0.00(0.02%)

C-C interaction:
```
 80. (2.00000)  98.0225% BD ( 1) C 15- C 20
                 49.262%  C 15 s( 29.35%)p 2.41( 70.61%)d 0.00(  0.04%)
                 48.911%  C 20 s( 31.90%)p 2.13( 68.05%)d 0.00(  0.05%)
  81. (2.00000)  83.0005% BD ( 2) C 15- C 20
                  3.463% Zr  6 s( 21.43%)p 0.04(  0.85%)d 3.63( 77.72%)
                 38.185%  C 15 s(  0.11%)p99.99( 99.83%)d 0.49(  0.05%)
                  5.119%  C 16 s(  0.06%)p99.99( 99.46%)d 7.60(  0.47%)
                  1.592%  C 18 s(  0.16%)p99.99( 99.66%)d 1.16(  0.18%)
                  4.141%  C 19 s(  0.04%)p99.99( 99.60%)d10.29(  0.36%)
                 44.832%  C 20 s(  0.63%)p99.99( 99.33%)d 0.07(  0.04%)
```

Zr-Me interaction:
```
  57. (2.00000)  97.1676% BD ( 1)Zr  6- C  7
                 20.134% Zr  6 s( 11.98%)p 0.00(  0.04%)d 7.34( 87.98%)
                 77.045%  C  7 s( 26.90%)p 2.72( 73.10%)d 0.00(  0.01%)
```

Resonance structure **5**:
C-H interaction:
```
  92. (2.00000)  97.4174% BD ( 1) C 20- H 23
                  1.153% Zr  6 s( 30.34%)p 0.02(  0.75%)d 2.27( 68.92%)
                 61.614%  C 20 s( 26.50%)p 2.77( 73.42%)d 0.00(  0.08%)
                 35.825%  H 23 s( 99.98%)p 0.00(  0.02%)
```

C-C interaction:
```
  82. (2.00000)  98.0118% BD ( 1) C 15- C 20
                 49.258%  C 15 s( 29.38%)p 2.40( 70.58%)d 0.00(  0.04%)
                 48.908%  C 20 s( 31.92%)p 2.13( 68.03%)d 0.00(  0.05%)
  90. (2.00000)  98.7531% BD ( 1) C 19- C 20
                 48.322%  C 19 s( 32.73%)p 2.05( 67.22%)d 0.00(  0.05%)
                 50.552%  C 20 s( 32.77%)p 2.05( 67.18%)d 0.00(  0.05%)
```

Zr-Me interaction:
```
  57. (2.00000)  97.1656% BD ( 1)Zr  6- C  7
                 20.137% Zr  6 s( 11.94%)p 0.00(  0.04%)d 7.37( 88.01%)
                 77.040%  C  7 s( 26.89%)p 2.72( 73.10%)d 0.00(  0.01%)
  58. (2.00000)  57.2981% BD ( 1)Zr  6- C 20
                  4.105% Zr  6 s( 20.47%)p 0.04(  0.78%)d 3.85( 78.75%)
                 13.825%  C 15 s(  0.14%)p99.99( 99.72%)d 1.06(  0.15%)
                  1.230%  C 16 s(  0.12%)p99.99( 98.75%)d 9.73(  1.13%)
                 11.079%  C 17 s(  0.05%)p99.99( 99.95%)d 0.07(  0.00%)
                  1.360%  C 18 s(  0.24%)p99.99( 98.99%)d 3.21(  0.77%)
                 14.245%  C 19 s(  0.03%)p99.99( 99.85%)d 3.90(  0.12%)
                 53.395%  C 20 s(  0.61%)p99.99( 99.37%)d 0.03(  0.02%)
```

Non-Covalent Interactions (NCI)

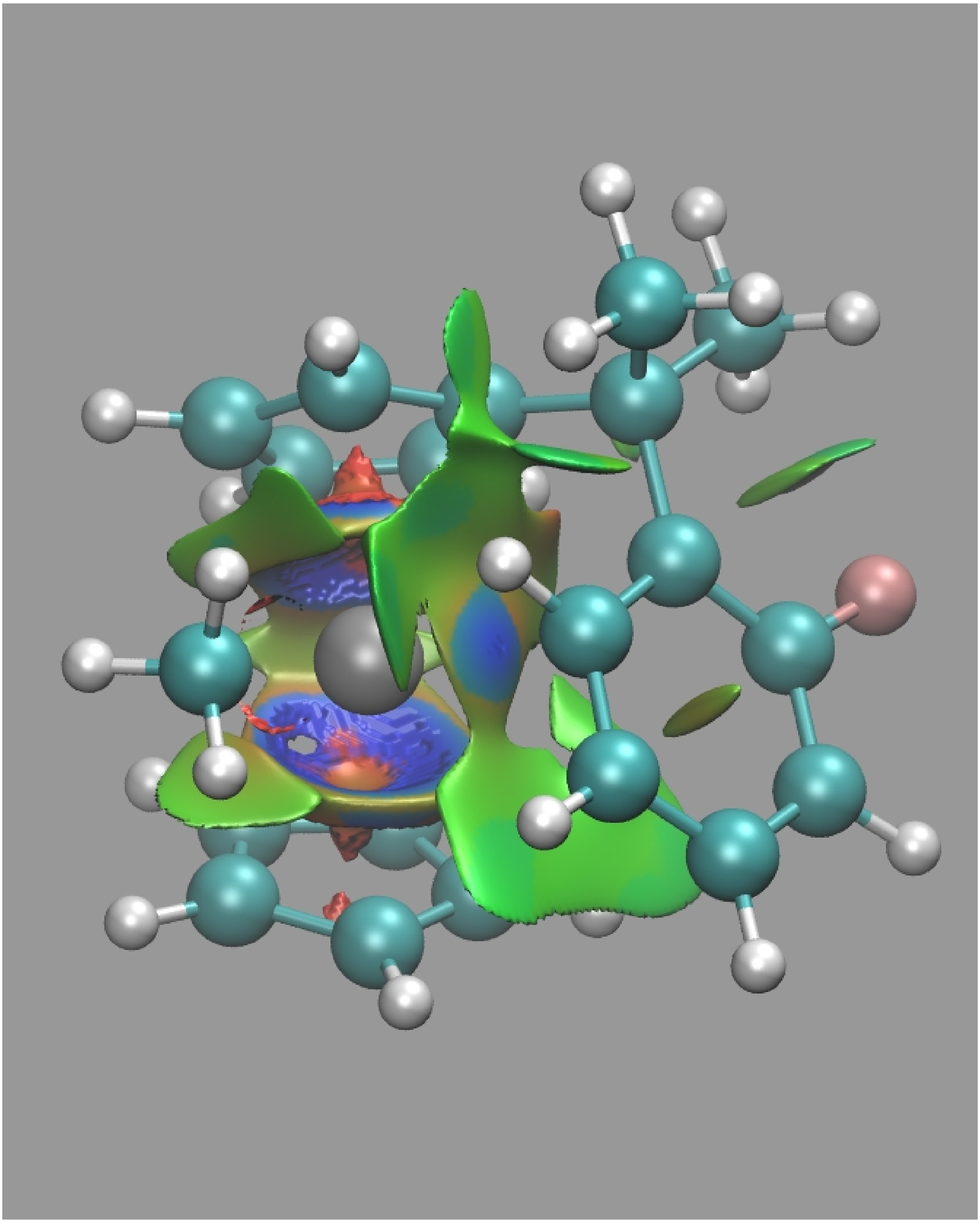

3B-PBE-D3

Bader:

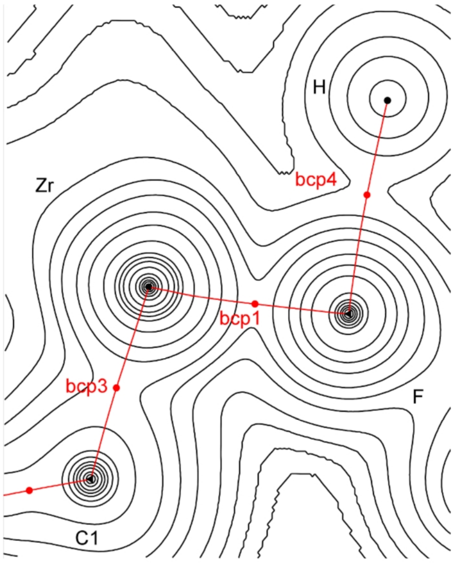

Electrondensity

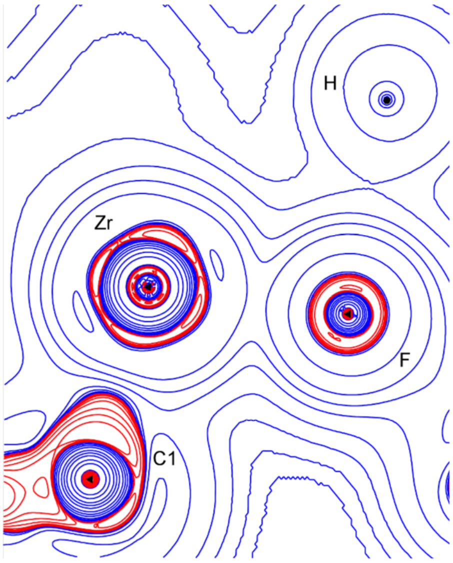

Laplacian

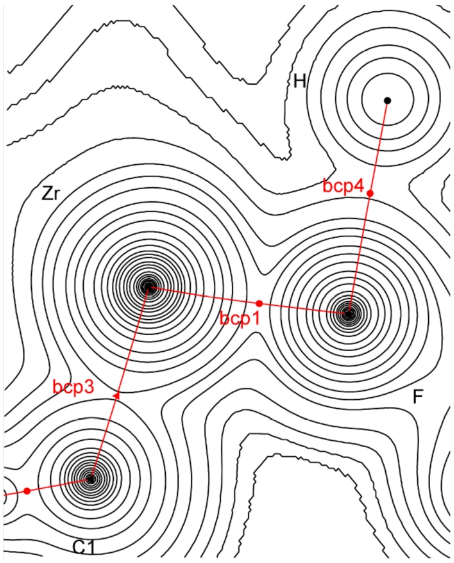

Virial

	$\varrho(\mathbf{r})$	$\nabla^2\varrho(\mathbf{r})$
bcp1	0.04751	-0.06524
bcp2	0.19082	-0.00349
bcp3	0.09619	-0.01201

NBO:

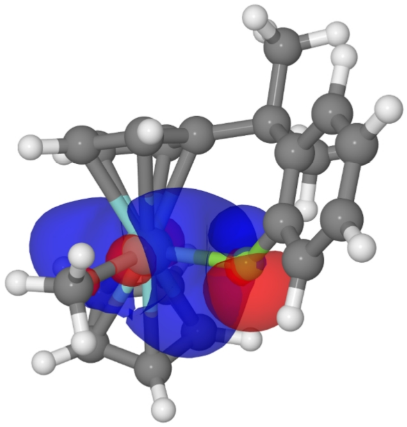

1

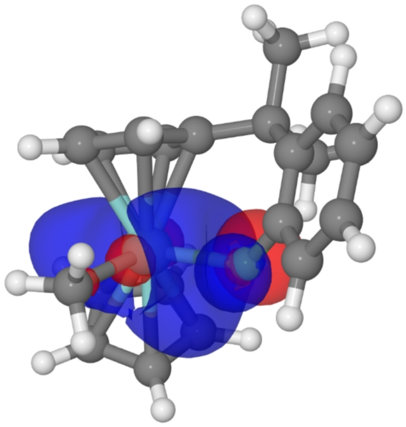

2

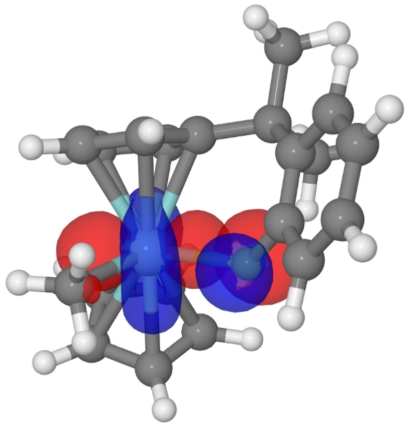

3

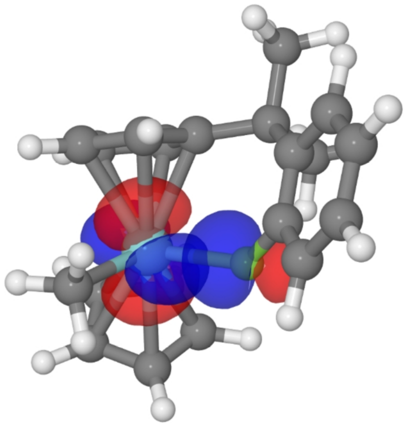

4

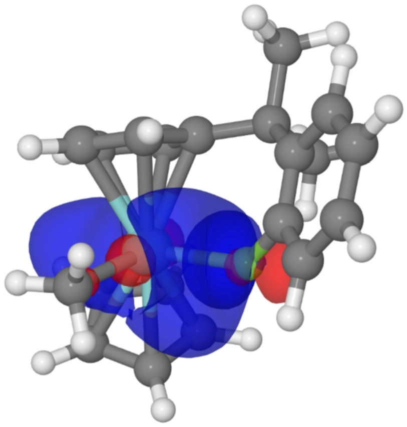

5

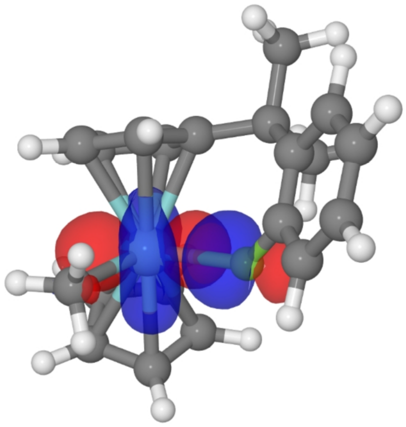

6

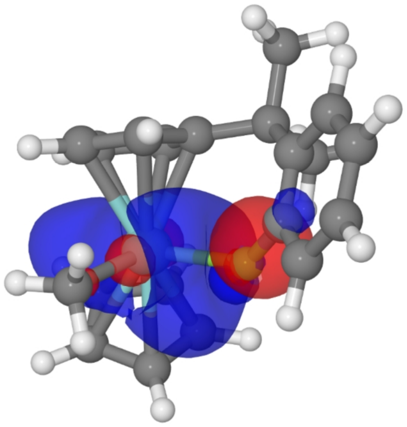

7

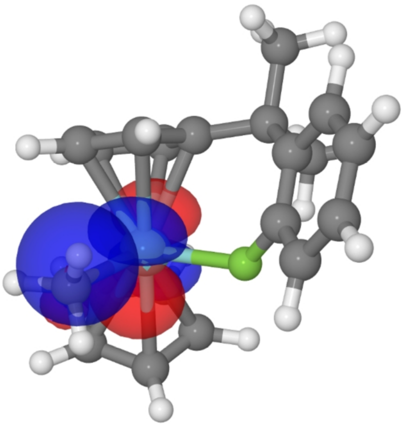

8

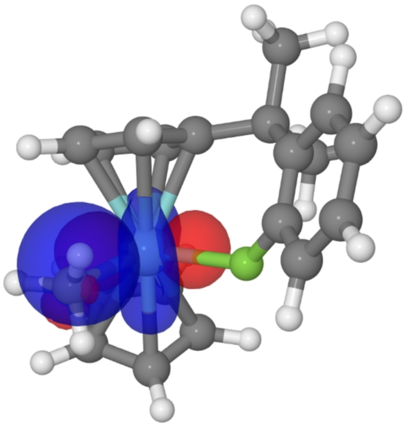

9

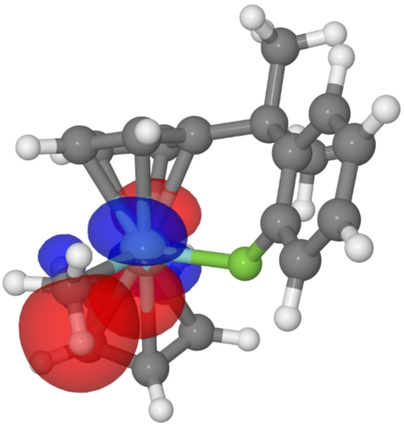

10

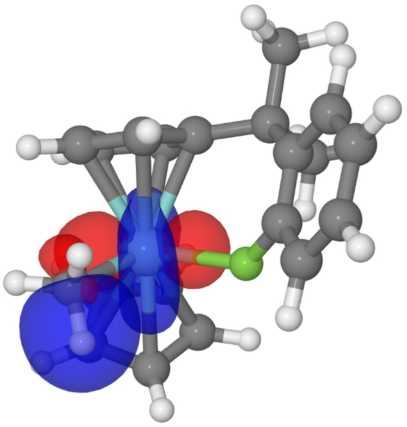

11

	Orbitals	E(2P)
1	$LP_F = sp^{2.54} \rightarrow$<br>$LV_{Zr} = sd^{0.21}$	7.79
2	$LP_F = sp^{6.80} \rightarrow$<br>$LV_{Zr} = sd^{0.21}$	5.26
3	$LP_F = sp^{6.80} \rightarrow$<br>$LV_{Zr} = sd^{24.01}$	4.39
4	$LP_F = sp^{2.20} \rightarrow$<br>$LV_{Zr} = sp^{0.52}d^{99.99}$	6.26
5	$LP_F = sp^{2.20} \rightarrow$<br>$LV_{Zr} = sd^{0.21}$	21.53
6	$LP_F = sp^{2.20} \rightarrow$<br>$LV_{Zr} = sd^{24.01}$	2.26
7	$\sigma_{CF} = 0.483(sp^{5.25})_{C15} - 0.876(sp^{2.61})_F \rightarrow$<br>$LV_{Zr} = sd^{0.21}$	3.49
8	$\sigma_{CH} = 0.781(sp^{2.97})_{C22} - 0.625(s)_{H42} \rightarrow$<br>$LV_{Zr} = p^1d^{99.99}$	2.20
9	$\sigma_{CH} = 0.781(sp^{2.97})_{C22} - 0.625(s)_{H42} \rightarrow$<br>$LV_{Zr} = sd^{24.01}$	2.38
10	$\sigma_{CH} = 0.781(sp^{2.97})_{C22} - 0.625(s)_{H44} \rightarrow$<br>$LV_{Zr} = p^1d^{99.99}$	2.08
11	$\sigma_{CH} = 0.781(sp^{2.97})_{C22} - 0.625(s)_{H44} \rightarrow$<br>$LV_{Zr} = sd^{24.01}$	2.29

Natural Resonance Theory:

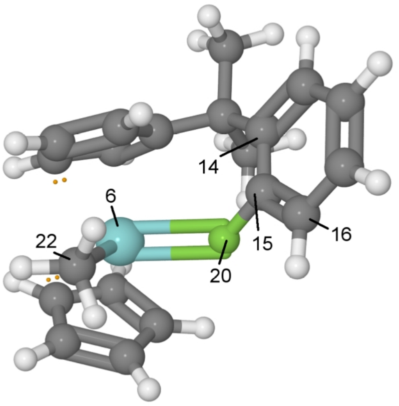

1

Wgt=24.54%;
rhoNL=5.27220;
D(0)=0.09618

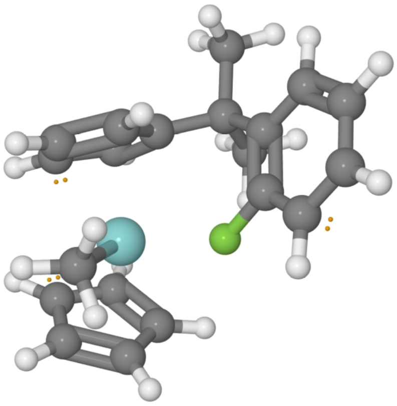

2

Wgt=10.55%;
rhoNL=6.00240;
D(0)=0.1026

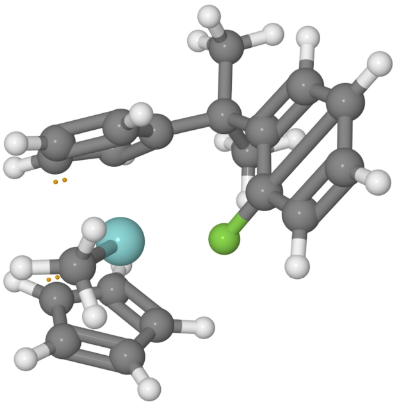

3

Wgt=9.40%;
rhoNL=5.70303;
D(0)=0.10004

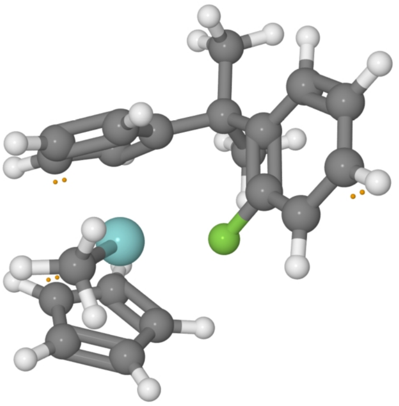

4

Wgt=6.84%;
rhoNL=6.04283;
D(0)=0.10297

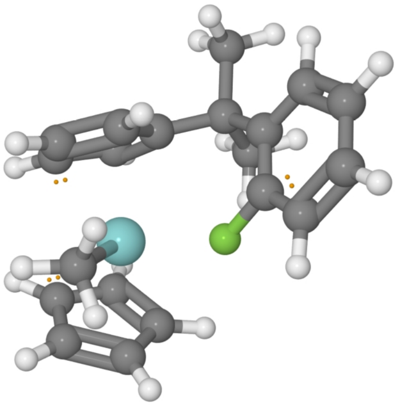

5

Wgt=6.29%;
rhoNL=5.97919;
D(0)=0.10243

Natural Localised Molecular Orbitals (NLMO):

Only contributions over 1% are reported.

NLMO / Occupancy / Percent from Parent NBO / Atomic Hybrid Contributions

Resonance structure **1**:
Zr-F interaction:

```
 55. (2.00000)  99.0100% BD ( 1)Zr  6- F 20
                  2.854% Zr  6 s( 31.40%)p 0.01(  0.36%)d 2.17( 68.24%)
                 96.377%  F 20 s( 39.22%)p 1.55( 60.78%)d 0.00(  0.00%)
 56. (2.00000)  97.9943% BD ( 2)Zr  6- F 20
                  1.345% Zr  6 s( 12.48%)p 0.05(  0.60%)d 6.97( 86.92%)
                 96.737%  F 20 s(  0.81%)p99.99( 99.18%)d 0.01(  0.01%)
```

C-F interaction:

```
  81. (2.00000)  99.5901% BD ( 1) C 15- F 20
                 23.102%  C 15 s( 18.47%)p 4.40( 81.22%)d 0.02(  0.31%)
                 76.521%  F 20 s( 32.25%)p 2.10( 67.73%)d 0.00(  0.02%)
```

C-C interaction:

```
  80. (2.00000)  85.2830% BD ( 2) C 15- C 16
                  5.631%  C 14 s(  0.02%)p99.99( 99.61%)d15.20(  0.37%)
                 44.561%  C 15 s(  0.01%)p99.99( 99.96%)d 2.43(  0.03%)
                 40.723%  C 16 s(  0.00%)p 1.00( 99.93%)d 0.00(  0.06%)
                  5.863%  C 17 s(  0.00%)p 1.00( 99.69%)d 0.00(  0.31%)
                  1.328%  C 18 s(  0.00%)p 1.00( 99.66%)d 0.00(  0.34%)
                  1.726%  C 19 s(  0.00%)p 1.00( 99.76%)d 0.00(  0.23%)
```

Zr-Me interaction:

```
  57. (2.00000)  96.8276% BD ( 1)Zr  6- C 22
                 22.189% Zr  6 s( 11.90%)p 0.00(  0.06%)d 7.40( 88.04%)
                 74.994%  C 22 s( 26.29%)p 2.80( 73.70%)d 0.00(  0.01%)
```

Resonance structure **2**:
C-F interaction:

```
  81. (2.00000)  99.5606% BD ( 1) C 15- F 20
                 23.172%  C 15 s( 18.90%)p 4.27( 80.79%)d 0.02(  0.31%)
                 76.424%  F 20 s( 27.75%)p 2.60( 72.23%)d 0.00(  0.02%)
```

C-C interaction:

```
 78. (2.00000)  84.3709% BD ( 2) C 14- C 15
                 40.402%  C 14 s(  0.01%)p 1.00( 99.93%)d 0.00(  0.06%)
                 43.969%  C 15 s(  0.01%)p99.99( 99.96%)d 2.26(  0.03%)
                  5.984%  C 16 s(  0.01%)p99.99( 99.61%)d35.62(  0.37%)
                  1.759%  C 17 s(  0.00%)p 1.00( 99.76%)d 0.00(  0.23%)
                  1.536%  C 18 s(  0.00%)p 1.00( 99.72%)d 0.00(  0.28%)
                  5.637%  C 19 s(  0.00%)p 1.00( 99.65%)d 0.00(  0.34%)
```

Zr-Me interaction:

```
                    0.013%  H 41 s( 99.41%)p 0.01(  0.59%)
  58. (2.00000)  97.1717% BD ( 1)Zr  6- C 22
                 22.204% Zr  6 s( 11.80%)p 0.00(  0.06%)d 7.47( 88.14%)
                 74.983%  C 22 s( 26.14%)p 2.83( 73.86%)d 0.00(  0.01%)
```

Resonance structure **3**:

C-F interaction:

```
  81. (2.00000)  99.5616% BD ( 1) C 15- F 20
                 23.175%  C 15 s( 18.89%)p 4.28( 80.79%)d 0.02(  0.31%)
                 76.422%  F 20 s( 27.74%)p 2.60( 72.23%)d 0.00(  0.02%)
```

C-C interaction:

```
 80. (2.00000)  66.7090% BD ( 1) C 15- C 18
                  8.304%  C 14 s(  0.02%)p99.99( 99.70%)d14.25(  0.28%)
                 37.105%  C 15 s(  0.01%)p99.99( 99.99%)d 0.08(  0.00%)
                  8.540%  C 16 s(  0.01%)p 1.00( 99.71%)d 0.00(  0.28%)
                  7.976%  C 17 s(  0.00%)p 1.00( 99.82%)d 0.00(  0.18%)
                 29.672%  C 18 s(  0.00%)p 1.00( 99.99%)d 0.00(  0.01%)
                  8.138%  C 19 s(  0.00%)p 1.00( 99.82%)d 0.00(  0.18%)
```

Zr-Me interaction:

```
  57. (2.00000)  97.1717% BD ( 1)Zr  6- C 22
                 22.205% Zr  6 s( 11.80%)p 0.00(  0.06%)d 7.47( 88.14%)
                 74.983%  C 22 s( 26.14%)p 2.83( 73.86%)d 0.00(  0.01%)
```

Resonance structure **4**:

NLMO algorithm failed to converge

Resonance structure **5**:

C-F interaction:

```
  80. (2.00000)  99.5616% BD ( 1) C 15- F 20
                 23.175%  C 15 s( 18.89%)p 4.28( 80.79%)d 0.02(  0.31%)
                 76.422%  F 20 s( 27.74%)p 2.60( 72.24%)d 0.00(  0.02%)
```

Zr-Me interaction:

```
  58. (2.00000)  97.1717% BD ( 1)Zr  6- C 22
                 22.204% Zr  6 s( 11.81%)p 0.00(  0.06%)d 7.47( 88.14%)
                 74.984%  C 22 s( 26.14%)p 2.83( 73.86%)d 0.00(  0.01%)
```

Non-Covalent Interactions (NCI)

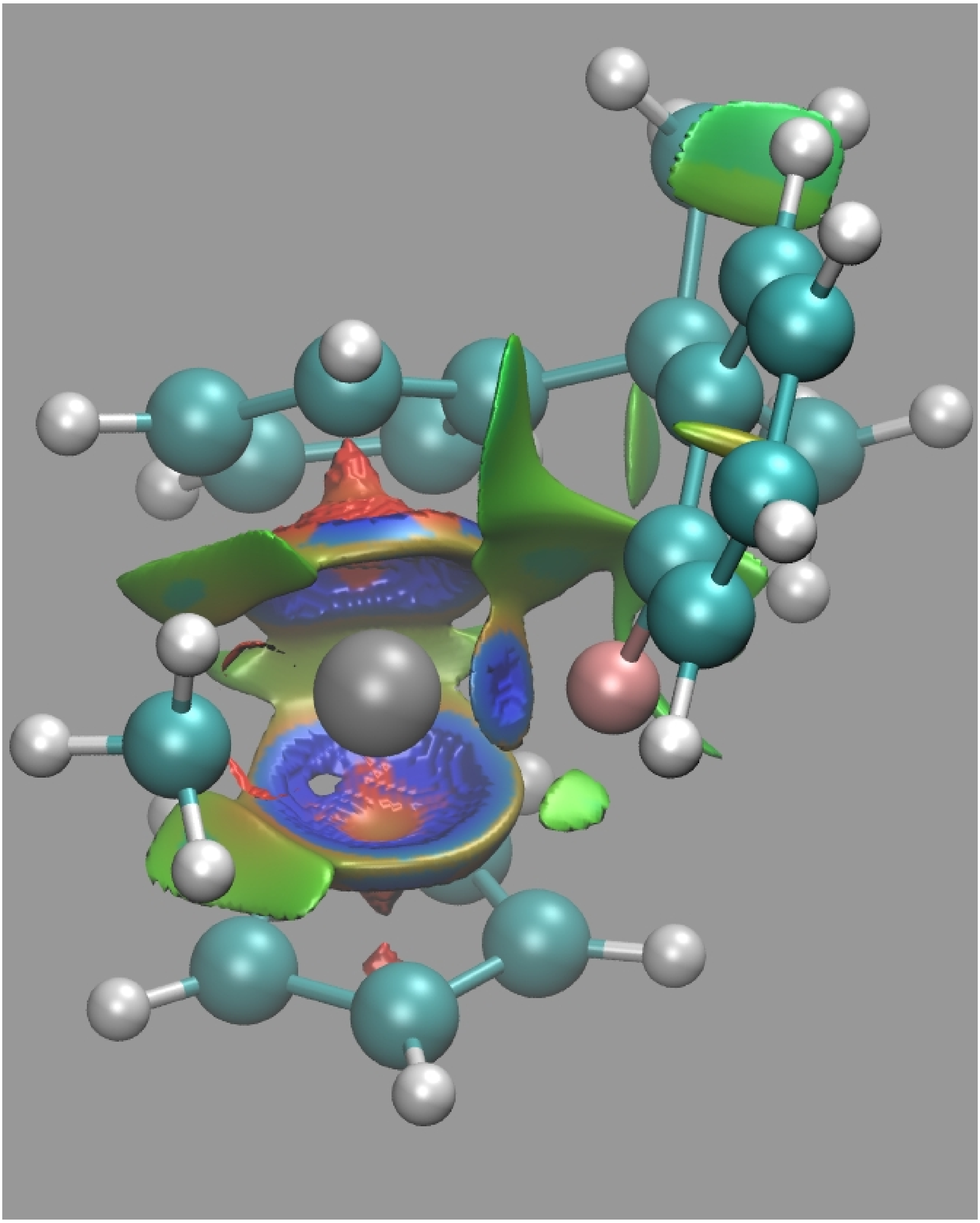

4A-PBE-D3

Bader:

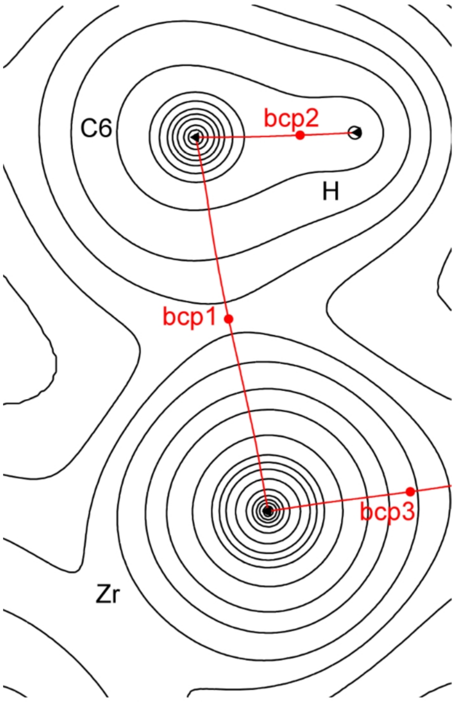

Electrondensity

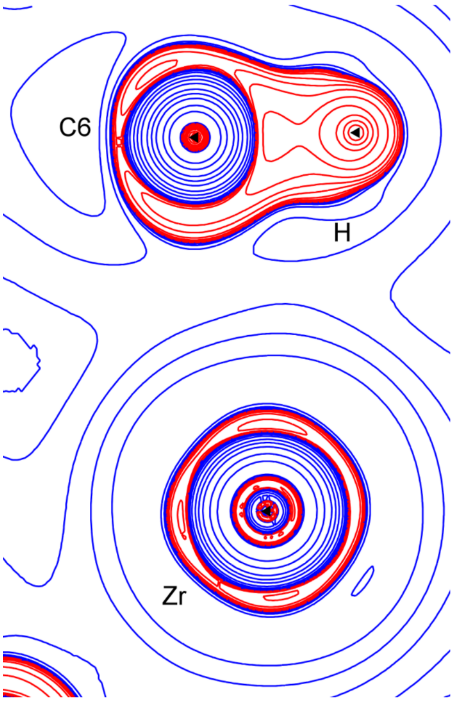

Laplacian

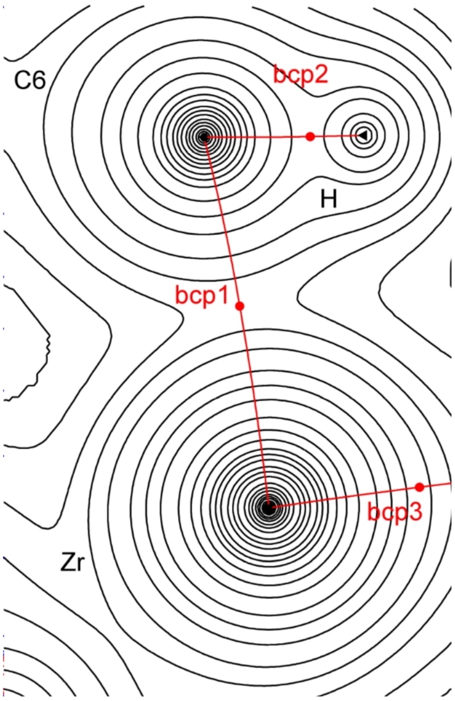

Virial

	$\varrho(\mathbf{r})$	$\nabla^2\varrho(\mathbf{r})$
bcp1	0.03659	-0.02169
bcp2	0.26773	0.21514
bcp3	0.09403	-0.01238

NBO:

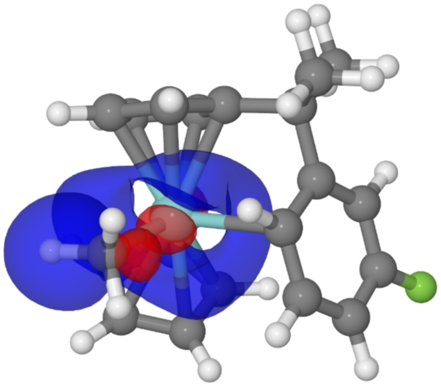

1

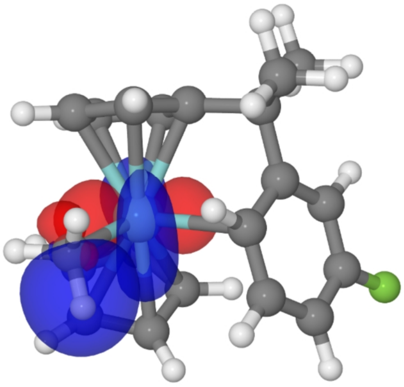

2

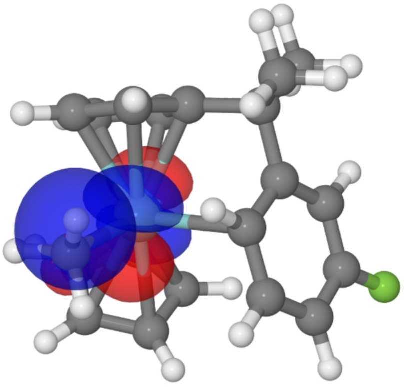

3

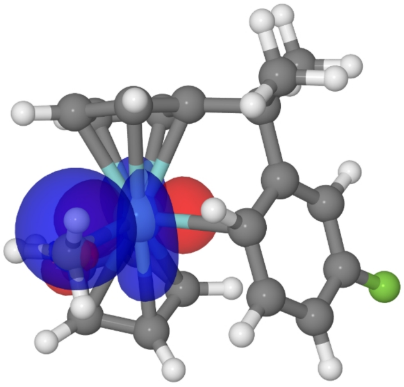

4

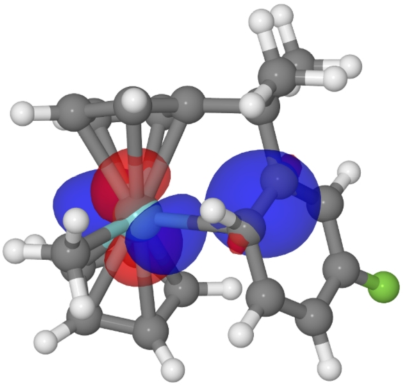

5

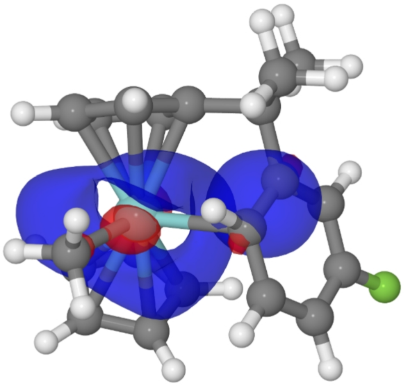

6

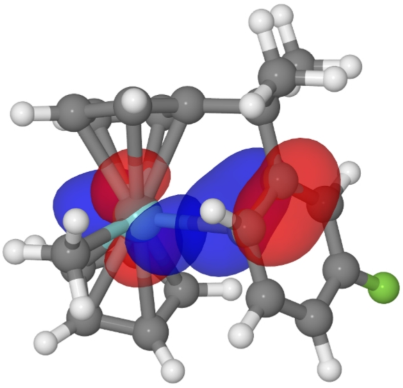

7

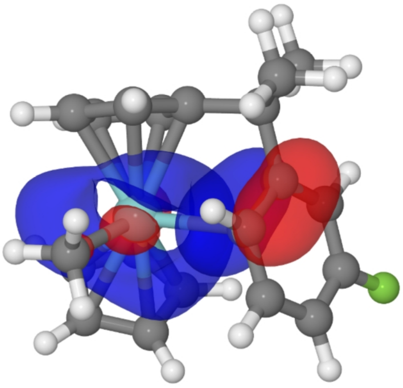

8

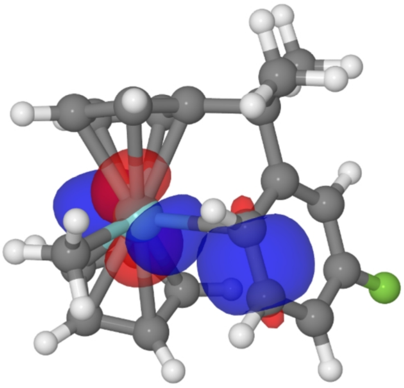

9

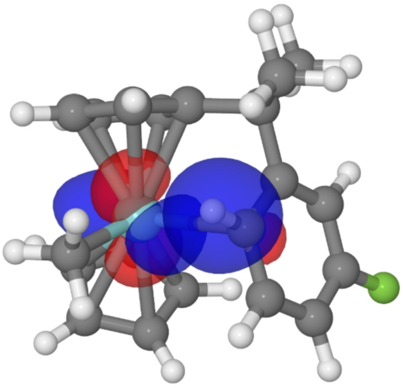

10

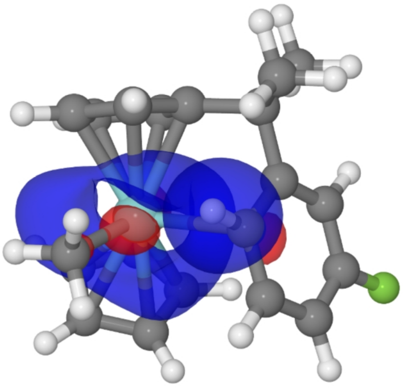

11

	Orbitals	E(2P)
1	$\sigma_{CH} = 0.787(sp^{3.23})_{C7} – 0.617(s)_{H42} \rightarrow$<br>$LV_{Zr} = sd^{0.28}$	2.11
2	$\sigma_{CH} = 0.781(sp^{3.03})_{C7} – 0.625(s)_{H43} \rightarrow$<br>$LV_{Zr} = sp^{0.04}d^{17.05}$	2.75
3	$\sigma_{CH} = 0.780(sp^{3.00})_{C7} – 0.626(s)_{H44} \rightarrow$<br>$LV_{Zr} = sp^{0.03}d^{0.30}$	2.22
4	$\sigma_{CH} = 0.780(sp^{3.00})_{C7} – 0.626(s)_{H44} \rightarrow$<br>$LV_{Zr} = sp^{0.04}d^{17.05}$	2.18
5	$\sigma_{CC} = 0.709(sp^{1.79})_{C8} – 0.706(sp^{2.02})_{C9} \rightarrow$<br>$LV_{Zr} = sp^{0.03}d^{42.86}$	3.03
6	$\sigma_{CC} = 0.709(sp^{1.79})_{C8} – 0.706(sp^{2.02})_{C9} \rightarrow$<br>$LV_{Zr} = sd^{0.28}$	3.08
7	$\pi_{CC} = 0.766(p)_{C8} – 0.643(p)_{C9} \rightarrow$<br>$LV_{Zr} = sp^{0.03}d^{42.86}$	8.82
8	$\pi_{CC} = 0.766(p)_{C8} – 0.643(p)_{C9} \rightarrow$<br>$LV_{Zr} = sd^{0.28}$	5.05
9	$\sigma_{CC} = 0.714(sp^{1.83})_{C8} – 0.700(sp^{1.77})_{C13} \rightarrow$<br>$LV_{Zr} = sp^{0.03}d^{42.86}$	3.43
10	$\sigma_{CH} = 0.795(sp^{2.53})_{C8} – 0.607(s)_{H23} \rightarrow$<br>$LV_{Zr} = sp^{0.03}d^{42.86}$	5.66
11	$\sigma_{CH} = 0.795(sp^{2.53})_{C8} – 0.607(s)_{H23} \rightarrow$<br>$LV_{Zr} = sd^{0.28}$	4.04

Natural Resonance Theory:

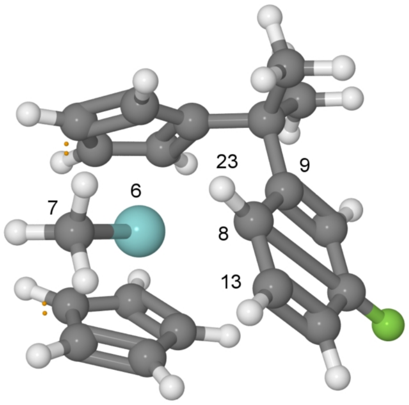

1

Wgt=9.37%;
rhoNL=5.69698;
D(0)=0.09998

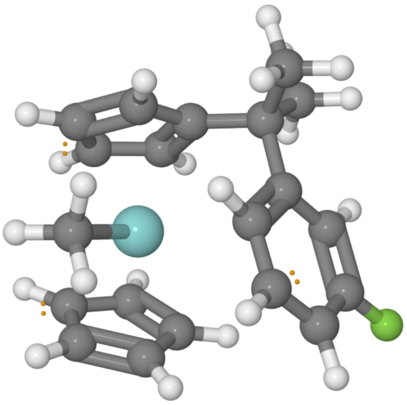

2

Wgt=8.61%;
rhoNL=6.16127;
D(0)=0.10398

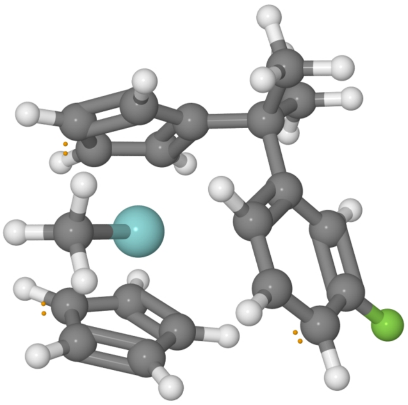

3

Wgt=7.19%;
rhoNL=6.08761;
D(0)=0.10335

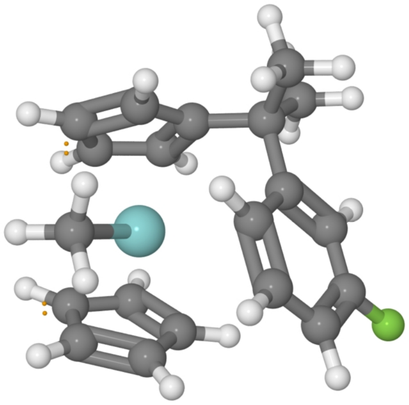

4

Wgt=6.71%;
rhoNL=5.42864;
D(0)=0.09760

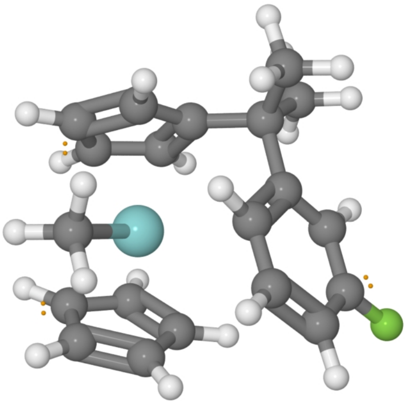

5

Wgt=6.17%;
rhoNL=6.10027;
D(0)=0.10346

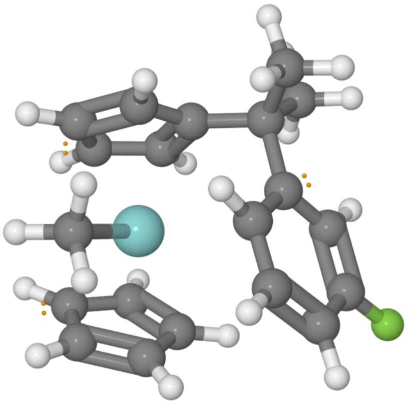

6

Wgt=5.40%;
rhoNL=6.14123;
D(0)=0.10381

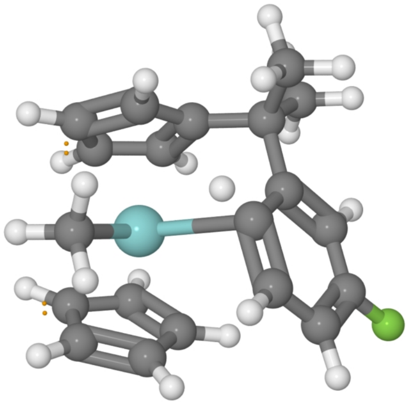

7

Wgt=5.07%;
rhoNL=6.11395;
D(0)=0.10358

Natural Localised Molecular Orbitals (NLMO):

Only contributions over 1% are reported.

NLMO / Occupancy / Percent from Parent NBO / Atomic Hybrid Contributions

Resonance structure **1**:
C-H interaction:

```
  64. (2.00000)  97.3043% BD ( 1) C  8- H 23
                  1.301% Zr  6 s( 29.76%)p 0.02(  0.73%)d 2.34( 69.51%)
                 61.576%  C  8 s( 26.79%)p 2.73( 73.13%)d 0.00(  0.08%)
                 35.754%  H 23 s( 99.98%)p 0.00(  0.02%)
```

C-C interaction:

```
 62. (2.00000)  67.2506% BD ( 1) C  8- C 11
                  3.642% Zr  6 s( 18.22%)p 0.05(  0.90%)d 4.44( 80.89%)
                 45.411%  C  8 s(  0.86%)p99.99( 99.13%)d 0.01(  0.01%)
                  7.238%  C  9 s(  0.17%)p99.99( 99.54%)d 1.72(  0.29%)
                  6.929%  C 10 s(  0.03%)p99.99( 99.78%)d 7.50(  0.19%)
                 21.888%  C 11 s(  0.05%)p99.99( 99.94%)d 0.25(  0.01%)
                  6.459%  C 12 s(  0.08%)p99.99( 99.71%)d 2.85(  0.22%)
                  7.420%  C 13 s(  0.02%)p99.99( 99.74%)d11.68(  0.24%)
```

Zr-Me interaction:

```
  57. (2.00000)  97.2504% BD ( 1)Zr  6- C  7
                 20.173% Zr  6 s( 12.17%)p 0.00(  0.04%)d 7.21( 87.79%)
                 77.088%  C  7 s( 26.92%)p 2.72( 73.08%)d 0.00(  0.01%)
```

Resonance structure **2**:
NLMO algorithm failed to converge

Resonance structure **3**:
C-H interaction:

```
  65. (2.00000)  97.2880% BD ( 1) C  8- H 23
                  1.260% Zr  6 s( 29.18%)p 0.03(  0.74%)d 2.40( 70.08%)
                 61.543%  C  8 s( 26.66%)p 2.75( 73.26%)d 0.00(  0.08%)
                 35.783%  H 23 s( 99.98%)p 0.00(  0.02%)
```

C-C interaction:

```
  62. (2.00000)  98.3410% BD ( 1) C  8- C  9
                 49.486%  C  8 s( 32.10%)p 2.11( 67.85%)d 0.00(  0.05%)
                 48.998%  C  9 s( 29.73%)p 2.36( 70.23%)d 0.00(  0.04%)
  63. (2.00000)  82.6458% BD ( 2) C  8- C  9
                  3.736% Zr  6 s( 19.77%)p 0.04(  0.76%)d 4.02( 79.47%)
                 48.397%  C  8 s(  0.95%)p99.99( 99.02%)d 0.03(  0.03%)
                 34.262%  C  9 s(  0.11%)p99.99( 99.84%)d 0.47(  0.05%)
                  4.354%  C 10 s(  0.00%)p 1.00( 99.67%)d 0.00(  0.33%)
                  1.082%  C 11 s(  0.16%)p99.99( 99.40%)d 2.65(  0.43%)
                  1.702%  C 12 s(  0.15%)p99.99( 99.64%)d 1.36(  0.21%)
                  5.245%  C 13 s(  0.03%)p99.99( 99.66%)d 9.45(  0.30%)
```

Zr-Me interaction:
58. (2.00000) 97.2498% BD (1)Zr 6- C 7
20.172% Zr 6 s(12.17%)p 0.00(0.04%)d 7.21(87.79%)
77.088% C 7 s(26.92%)p 2.72(73.08%)d 0.00(0.01%)

Resonance structure **4**:
C-H interaction:
64. (2.00000) 97.2867% BD (1) C 8- H 23
1.263% Zr 6 s(29.38%)p 0.03(0.74%)d 2.38(69.87%)
61.543% C 8 s(26.70%)p 2.74(73.22%)d 0.00(0.08%)
35.780% H 23 s(99.98%)p 0.00(0.02%)

C-C interaction:
62. (2.00000) 98.7441% BD (1) C 8- C 13
50.374% C 8 s(31.72%)p 2.15(68.23%)d 0.00(0.05%)
48.489% C 13 s(33.19%)p 2.01(66.77%)d 0.00(0.04%)
63. (2.00000) 83.5028% BD (2) C 8- C 13
3.031% Zr 6 s(22.02%)p 0.03(0.74%)d 3.51(77.23%)
49.404% C 8 s(0.86%)p99.99(99.12%)d 0.02(0.02%)
5.198% C 9 s(0.25%)p99.99(99.40%)d 1.41(0.35%)
1.863% C 10 s(0.03%)p99.99(99.80%)d 5.34(0.17%)
1.065% C 11 s(0.16%)p99.99(99.39%)d 2.74(0.45%)
4.577% C 12 s(0.00%)p 1.00(99.67%)d 0.00(0.32%)
34.115% C 13 s(0.02%)p99.99(99.94%)d 1.79(0.04%)

Zr-Me interaction:
57. (2.00000) 97.2498% BD (1)Zr 6- C 7
20.174% Zr 6 s(12.16%)p 0.00(0.04%)d 7.22(87.80%)
77.086% C 7 s(26.92%)p 2.72(73.08%)d 0.00(0.01%)

Resonance structure **5**:
NLMO algorithm failed to converge

Resonance structure **6**:
NLMO algorithm failed to converge

Resonance structure **7**:
C-C interaction:
63. (2.00000) 98.7883% BD (1) C 8- C 13
50.478% C 8 s(30.10%)p 2.32(69.85%)d 0.00(0.05%)
48.405% C 13 s(33.24%)p 2.01(66.72%)d 0.00(0.04%)
64. (2.00000) 83.5745% BD (2) C 8- C 13
3.020% Zr 6 s(22.83%)p 0.03(0.72%)d 3.35(76.45%)
49.372% C 8 s(0.81%)p99.99(99.17%)d 0.03(0.02%)
5.064% C 9 s(0.26%)p99.99(99.38%)d 1.40(0.36%)
1.955% C 10 s(0.03%)p99.99(99.81%)d 5.88(0.16%)
1.107% C 11 s(0.16%)p99.99(99.41%)d 2.80(0.43%)
4.561% C 12 s(0.00%)p 1.00(99.67%)d 0.00(0.32%)
34.214% C 13 s(0.02%)p99.99(99.94%)d 1.80(0.04%)

Zr-C interaction:

```
 58. (2.00000)  62.5565% BD ( 1)Zr  6- C  8
                  1.524% Zr  6 s( 26.84%)p 0.02(  0.61%)d 2.70( 72.55%)
                 61.333%  C  8 s( 29.55%)p 2.38( 70.37%)d 0.00(  0.08%)
                 35.688%  H 23 s( 99.98%)p 0.00(  0.02%)
```

Zr-Me interaction:

```
 57. (2.00000)  97.2496% BD ( 1)Zr  6- C  7
                 20.174% Zr  6 s( 12.16%)p 0.00(  0.04%)d 7.22( 87.79%)
                 77.086%  C  7 s( 26.91%)p 2.72( 73.08%)d 0.00(  0.01%)
```

Non-Covalent Interactions (NCI)

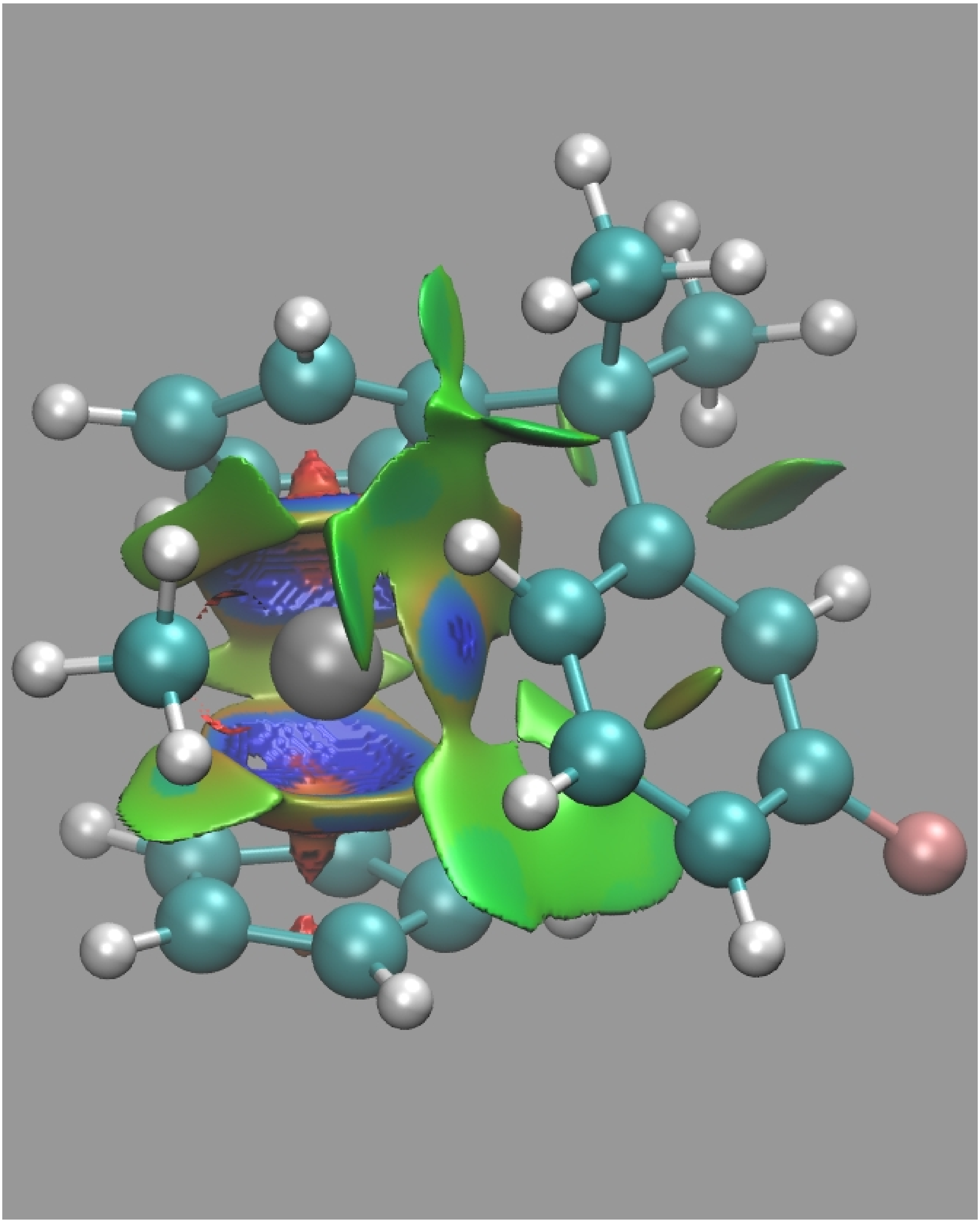

4B-PBE-D3

Bader:

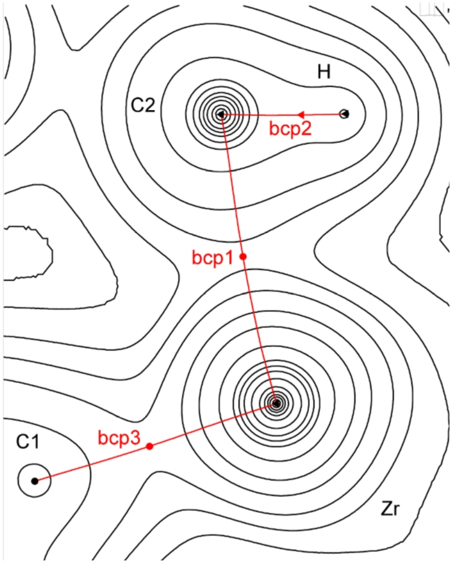

Electrondensity

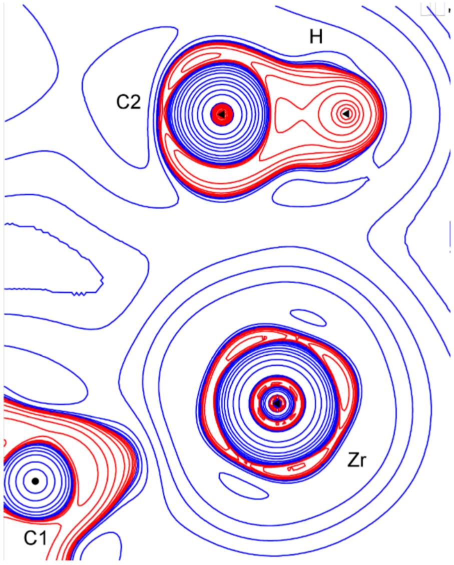

Laplacian

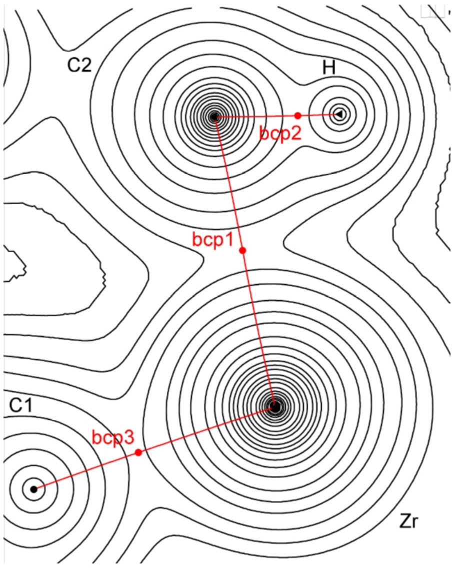

Virial

	$\varrho(\mathbf{r})$	$\nabla^2\varrho(\mathbf{r})$
bcp1	0.03548	-0.02124
bcp2	0.26247	0.20669
bcp3	0.09533	-0.01212

NBO:

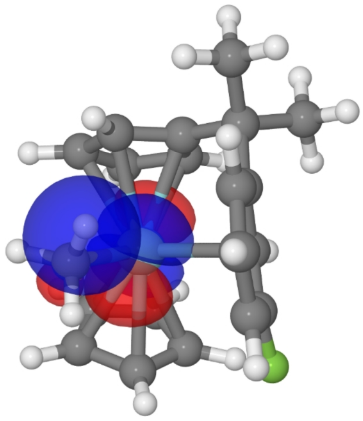

1

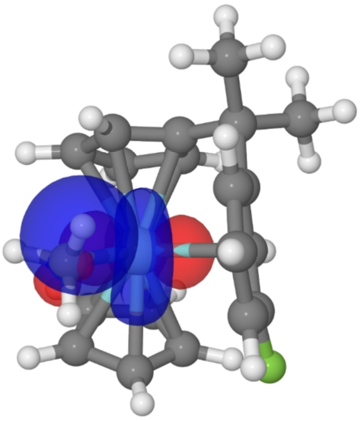

2

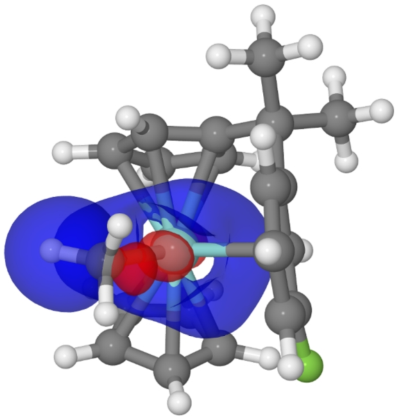

3

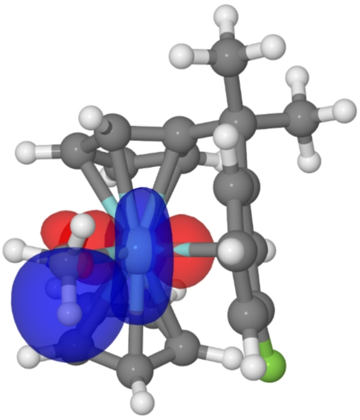

4

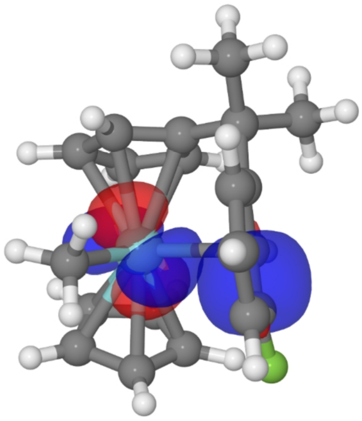

5

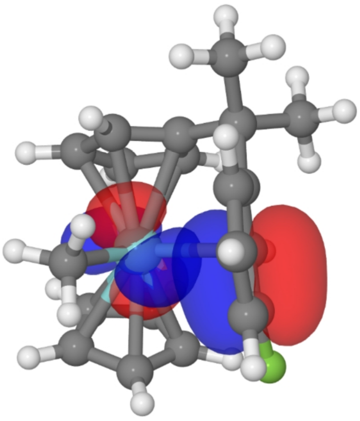

6

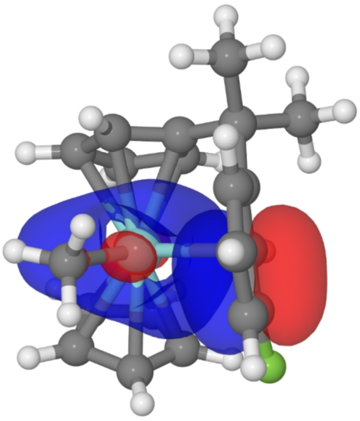

7

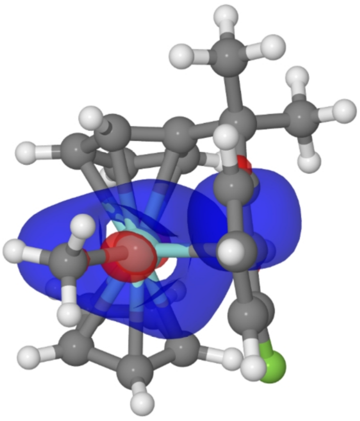

8

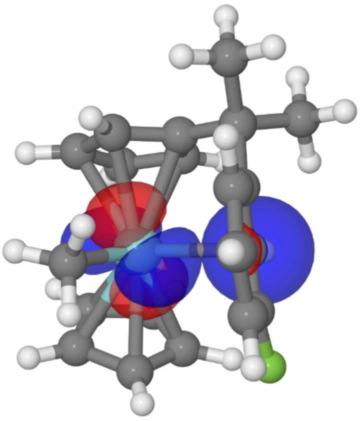

9

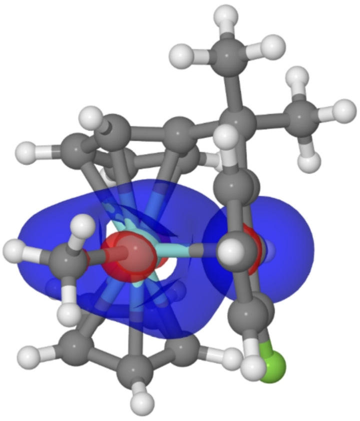

10

	Orbitals	E(2P)
1	σ_{CH} = 0.781(sp$^{3.01)}$C$_7$ – 0.625(s)H$_{37}$ → LV$_{Zr}$ = sp$^{0.30}$d$^{99.99}$	2.19
2	σ_{CH} = 0.781(sp$^{3.01)}$C$_7$ – 0.625(s)H$_{37}$ → LV$_{Zr}$ = sd$^{10.28}$	3.48
3	σ_{CH} = 0.787(sp$^{3.20)}$C$_7$ – 0.617(s)H$_{38}$ → LV$_{Zr}$ = sd$^{0.32}$	2.23
4	σ_{CH} = 0.781(sp$^{2.97)}$C$_7$ – 0.6244(s)H$_{39}$ → LV$_{Zr}$ = sd$^{10.28}$	2.54
5	σ_{CC} = 0.715(sp$^{1.98}$)$_{C8}$ – 0.699(sp$^{1.57}$)$_{C9}$ → LV$_{Zr}$ = sd$^{54.80}$	2.91
6	π_{CC} = 0.779(sp$^{95.16}$)$_{C8}$ – 0.628(p)$_{C9}$ → LV$_{Zr}$ = sd$^{54.80}$	7.39
7	π_{CC} = 0.779(sp$^{95.16}$)$_{C8}$ – 0.628(p)$_{C9}$ → LV$_{Zr}$ = sd$^{0.32}$	4.44
8	σ_{CC} = 0.715(sp$^{1.96}$)$_{C8}$ – 0.699(sp$^{2.10}$)$_{C13}$ → LV$_{Zr}$ = sd$^{0.32}$	3.27
9	σ_{CH} = 0.795(sp$^{2.52}$)$_{C8}$ – 0.606(s)$_{H26}$ → LV$_{Zr}$ = sd$^{54.80}$	2.64
10	σ_{CH} = 0.795(sp$^{2.52}$)$_{C8}$ – 0.606(s)$_{H26}$ → LV$_{Zr}$ = sd$^{0.32}$	6.75

Natural Resonance Theory:

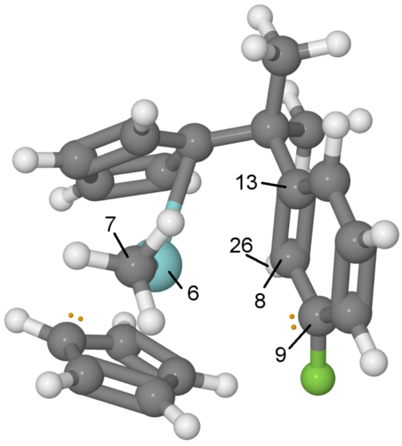

1

Wgt=16.70%;
rhoNL=5.94539;
D(0)=0.10214

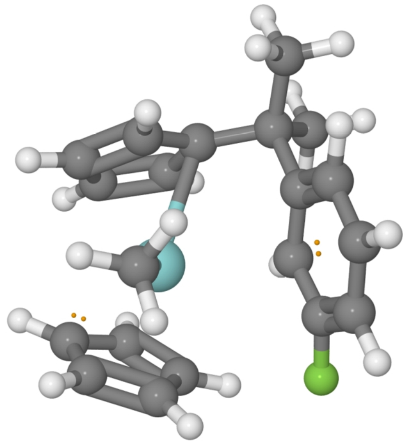

2

Wgt=13.51%;
rhoNL=5.72458;
D(0)=0.10022

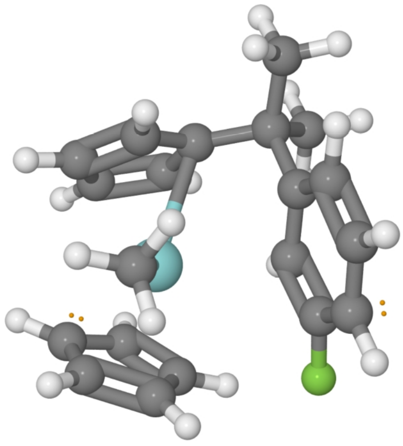

3

Wgt=12.99%;
rhoNL=5.89286;
D(0)=0.10169

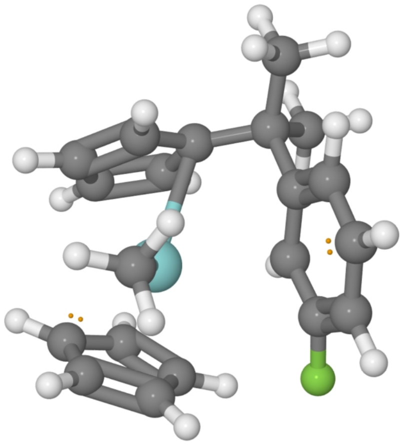

4

Wgt=12.63%;
rhoNL=6.02482;
D(0)=0.10282

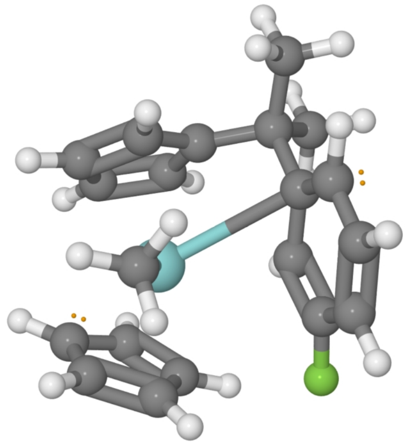

5

Wgt=12.05%;
rhoNL=6.16586;
D(0)=0.10401

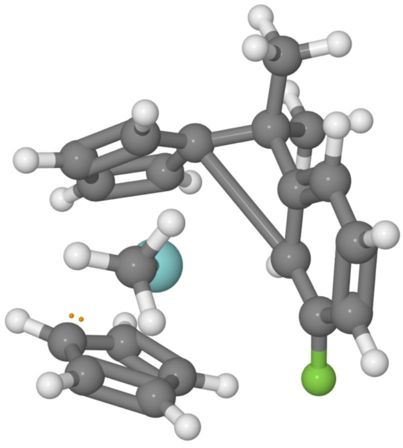

6

Wgt=11.04%;
rhoNL=6.61545;
D(0)=0.10774

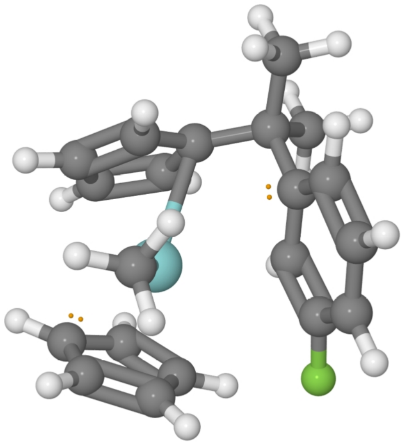 **7**	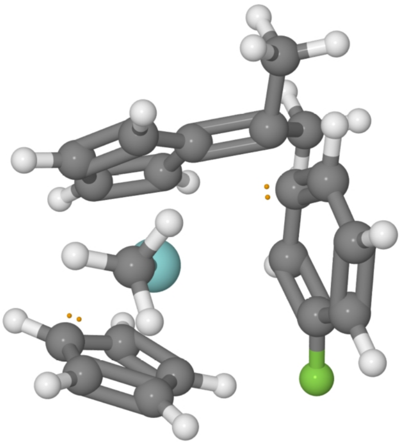 **8**
Wgt=9.43%;<br>rhoNL=5.96571;<br>D(0)=0.10231	Wgt=7.15%;<br>rhoNL=6.27755;<br>D(0)=0.10495

Natural Localised Molecular Orbitals (NLMO):

Only contributions over 1% are reported.

NLMO / Occupancy / Percent from Parent NBO / Atomic Hybrid Contributions

Resonance structure **1**:
C-H interaction:

```
 65. (2.00000)  96.8083% BD ( 1) C  8- H 26
                  1.715% Zr  6 s( 28.80%)p 0.02(  0.51%)d 2.46( 70.70%)
                 61.297%  C  8 s( 26.92%)p 2.71( 73.00%)d 0.00(  0.08%)
                 35.546%  H 26 s( 99.97%)p 0.00(  0.03%)
```

C-C interaction:

```
 63. (2.00000)  98.1445% BD ( 1) C  8- C 13
                 50.275%  C  8 s( 33.00%)p 2.03( 66.96%)d 0.00(  0.04%)
                 48.024%  C 13 s( 28.95%)p 2.45( 71.01%)d 0.00(  0.04%)
 64. (2.00000)  83.3373% BD ( 2) C  8- C 13
                  3.226% Zr  6 s( 23.45%)p 0.03(  0.70%)d 3.23( 75.85%)
                 51.187%  C  8 s(  1.23%)p79.98( 98.75%)d 0.01(  0.02%)
                  5.300%  C  9 s(  0.02%)p99.99( 99.55%)d17.36(  0.42%)
                  1.560%  C 10 s(  0.13%)p99.99( 99.71%)d 1.29(  0.17%)
                  1.007%  C 11 s(  0.13%)p99.99( 99.50%)d 2.92(  0.37%)
                  4.334%  C 12 s(  0.00%)p 1.00( 99.66%)d 0.00(  0.34%)
                 32.167%  C 13 s(  0.13%)p99.99( 99.82%)d 0.34(  0.05%)
```

Zr-Me interaction:

```
 57. (2.00000)  97.3876% BD ( 1)Zr  6- C  7
                 20.752% Zr  6 s( 11.95%)p 0.00(  0.04%)d 7.36( 88.01%)
                 76.648%  C  7 s( 26.55%)p 2.77( 73.44%)d 0.00(  0.01%)
```

Resonance structure **2**:
C-H interaction:

```
 64. (2.00000)  96.8283% BD ( 1) C  8- H 26
                  1.752% Zr  6 s( 28.92%)p 0.02(  0.50%)d 2.44( 70.58%)
                 61.333%  C  8 s( 27.04%)p 2.70( 72.88%)d 0.00(  0.08%)
                 35.518%  H 26 s( 99.97%)p 0.00(  0.03%)
```

C-C interaction:

```
 62. (2.00000)  98.8273% BD ( 1) C  8- C  9
                 50.557%  C  8 s( 30.56%)p 2.27( 69.36%)d 0.00(  0.08%)
                 48.374%  C  9 s( 37.08%)p 1.70( 62.89%)d 0.00(  0.02%)
 63. (2.00000)  98.1377% BD ( 1) C  8- C 13
                 50.279%  C  8 s( 33.05%)p 2.02( 66.90%)d 0.00(  0.04%)
                 48.017%  C 13 s( 28.94%)p 2.45( 71.02%)d 0.00(  0.04%)
```

Zr-Me interaction:

```
 57. (2.00000)  97.3884% BD ( 1)Zr  6- C  7
                 20.750% Zr  6 s( 11.96%)p 0.00(  0.04%)d 7.36( 88.00%)
                 76.650%  C  7 s( 26.55%)p 2.77( 73.44%)d 0.00(  0.01%)
```

Resonance structure **3**:
C-H interaction:
65. (2.00000) 96.8052% BD (1) C 8- H 26
1.698% Zr 6 s(28.74%)p 0.02(0.51%)d 2.46(70.75%)
61.281% C 8 s(26.88%)p 2.72(73.04%)d 0.00(0.08%)
35.562% H 26 s(99.97%)p 0.00(0.03%)

C-C interaction:
62. (2.00000) 98.8339% BD (1) C 8- C 9
50.556% C 8 s(30.53%)p 2.27(69.40%)d 0.00(0.08%)
48.377% C 9 s(37.09%)p 1.70(62.89%)d 0.00(0.02%)
63. (2.00000) 84.3045% BD (2) C 8- C 9
3.110% Zr 6 s(23.77%)p 0.03(0.67%)d 3.18(75.56%)
52.142% C 8 s(1.28%)p76.84(98.69%)d 0.02(0.02%)
32.194% C 9 s(0.02%)p99.99(99.94%)d 2.68(0.04%)
3.948% C 10 s(0.00%)p 1.00(99.59%)d 0.00(0.41%)
1.011% C 11 s(0.12%)p99.99(99.55%)d 2.64(0.32%)
1.355% C 12 s(0.15%)p99.99(99.60%)d 1.59(0.24%)
5.478% C 13 s(0.24%)p99.99(99.46%)d 1.24(0.30%)

Zr-Me interaction:
57. (2.00000) 97.3889% BD (1)Zr 6- C 7
20.748% Zr 6 s(11.96%)p 0.00(0.04%)d 7.36(88.00%)
76.653% C 7 s(26.55%)p 2.77(73.45%)d 0.00(0.01%)

Resonance structure **4**:
NLMO algorithm failed to converge
C-H interaction:

Resonance structure **5**:
NLMO algorithm failed to converge

Resonance structure **6**:
NLMO algorithm failed to converge

Resonance structure **7**:
C-H interaction:
65. (2.00000) 96.8019% BD (1) C 8- H 26
1.706% Zr 6 s(28.70%)p 0.02(0.51%)d 2.47(70.80%)
61.287% C 8 s(26.89%)p 2.72(73.03%)d 0.00(0.08%)
35.554% H 26 s(99.97%)p 0.00(0.03%)

C-C interaction:

```
  62. (2.00000)  98.8339% BD ( 1) C  8- C  9
                 50.556%  C  8 s( 30.52%)p 2.27( 69.40%)d 0.00(  0.08%)
                 48.377%  C  9 s( 37.09%)p 1.70( 62.89%)d 0.00(  0.02%)
  63. (2.00000)  84.1717% BD ( 2) C  8- C  9
                  3.002% Zr  6 s( 23.85%)p 0.03(  0.66%)d 3.17( 75.50%)
                 51.169%  C  8 s(  1.28%)p77.19( 98.70%)d 0.02(  0.02%)
                 33.018%  C  9 s(  0.02%)p99.99( 99.94%)d 2.73(  0.04%)
                  5.229%  C 10 s(  0.00%)p 1.00( 99.67%)d 0.00(  0.33%)
                  1.115%  C 12 s(  0.18%)p99.99( 99.54%)d 1.62(  0.29%)
                  5.269%  C 13 s(  0.24%)p99.99( 99.45%)d 1.25(  0.30%)
```

Zr-Me interaction:

```
  57. (2.00000)  97.3883% BD ( 1)Zr  6- C  7
                 20.749% Zr  6 s( 11.96%)p 0.00(  0.04%)d 7.36( 88.00%)
                 76.651%  C  7 s( 26.55%)p 2.77( 73.45%)d 0.00(  0.01%)
```

Resonance structure **8**:

C-H interaction:

```
  64. (2.00000)  96.8074% BD ( 1) C  8- H 26
                  1.695% Zr  6 s( 28.41%)p 0.02(  0.51%)d 2.50( 71.08%)
                 61.281%  C  8 s( 26.90%)p 2.71( 73.02%)d 0.00(  0.08%)
                 35.565%  H 26 s( 99.97%)p 0.00(  0.03%)
```

C-C interaction:

```
  61. (2.00000)  98.8341% BD ( 1) C  8- C  9
                 50.553%  C  8 s( 30.49%)p 2.28( 69.43%)d 0.00(  0.08%)
                 48.379%  C  9 s( 37.13%)p 1.69( 62.85%)d 0.00(  0.02%)
  62. (2.00000)  84.1365% BD ( 2) C  8- C  9
                  3.179% Zr  6 s( 21.66%)p 0.03(  0.65%)d 3.59( 77.70%)
                 51.862%  C  8 s(  1.26%)p78.06( 98.71%)d 0.02(  0.02%)
                 32.301%  C  9 s(  0.01%)p99.99( 99.94%)d 3.03(  0.04%)
                  4.196%  C 10 s(  0.00%)p 1.00( 99.60%)d 0.00(  0.39%)
                  1.085%  C 11 s(  0.14%)p99.99( 99.54%)d 2.33(  0.32%)
                  1.680%  C 12 s(  0.14%)p99.99( 99.67%)d 1.25(  0.18%)
                  5.016%  C 13 s(  0.27%)p99.99( 99.40%)d 1.24(  0.33%)
```

Zr-Me interaction:

```
  57. (2.00000)  97.3845% BD ( 1)Zr  6- C  7
                 20.784% Zr  6 s( 11.98%)p 0.00(  0.04%)d 7.34( 87.98%)
                 76.614%  C  7 s( 26.55%)p 2.77( 73.44%)d 0.00(  0.01%)
```

Non-Covalent Interactions (NCI)

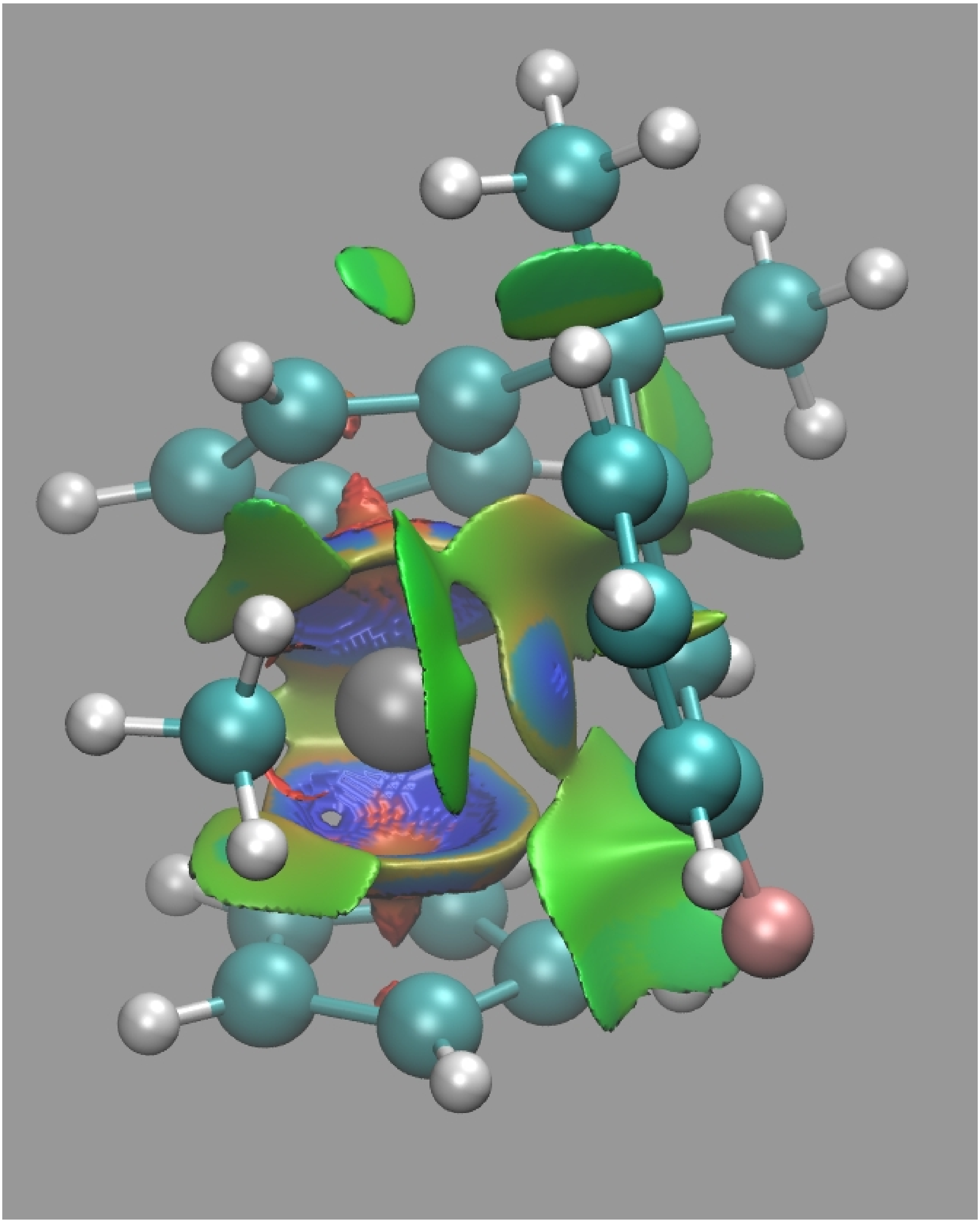

5A-PBE-D3

Bader:

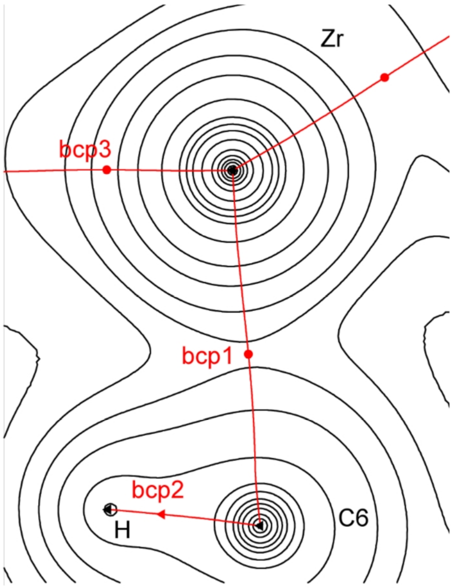

Electrondensity

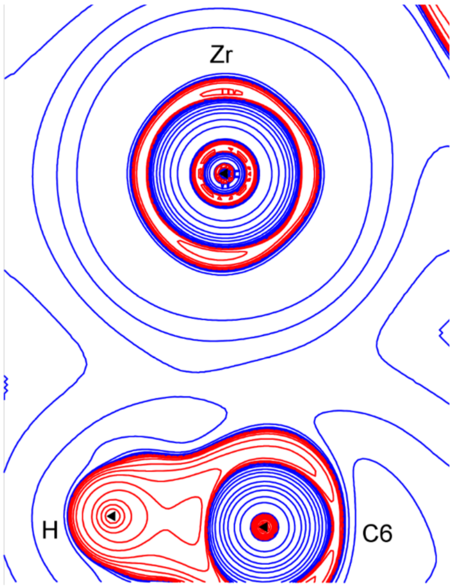

Laplacian

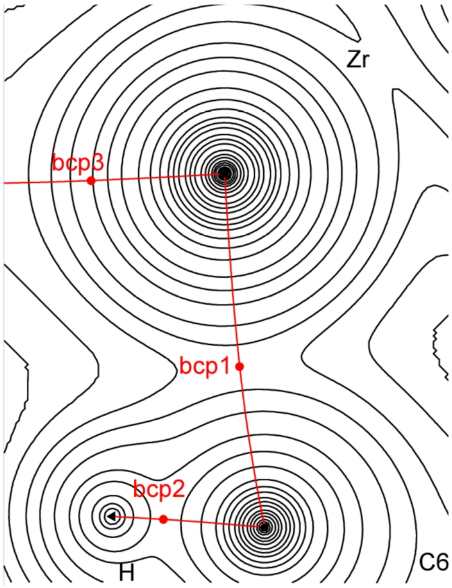

Virial

	ϱ(**r**)	∇^2ϱ(**r**)
bcp1	0.03745	-0.02333
bcp2	0.26833	0.21630
bcp3	0.09431	-0.01245

NBO:

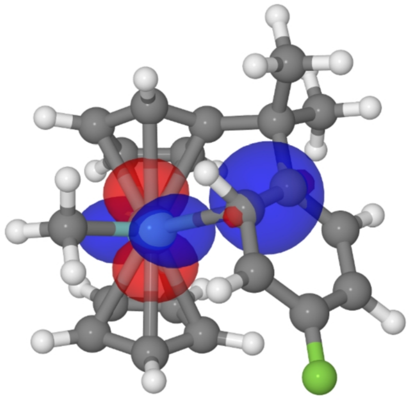

1

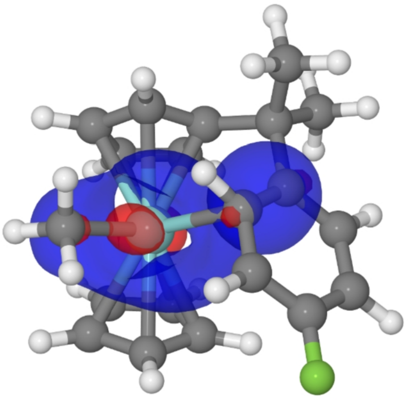

2

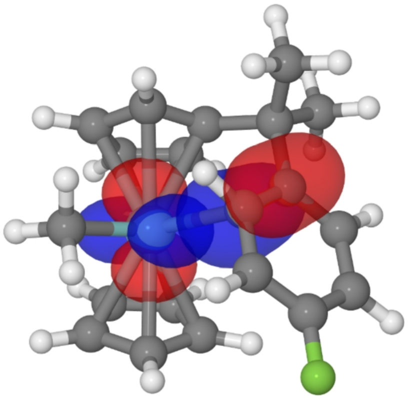

3

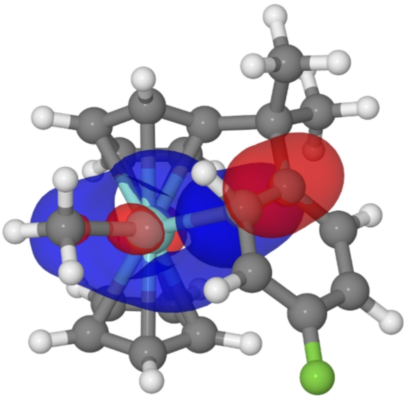

4

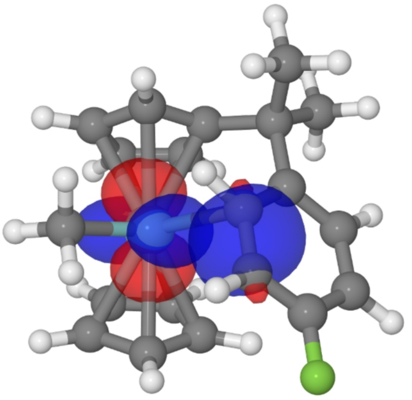

5

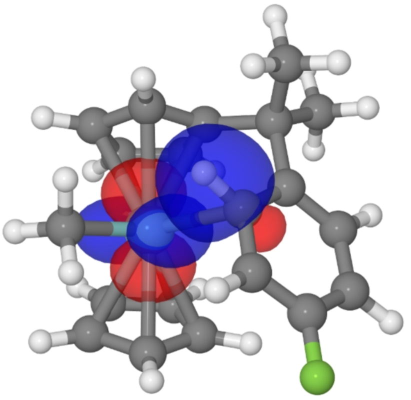

6

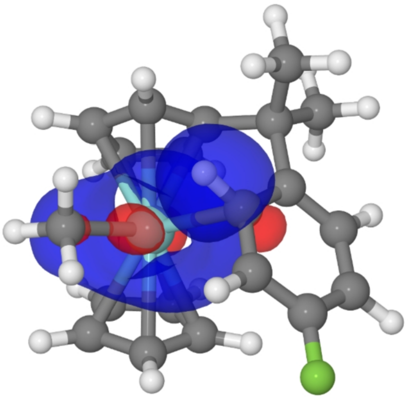

7

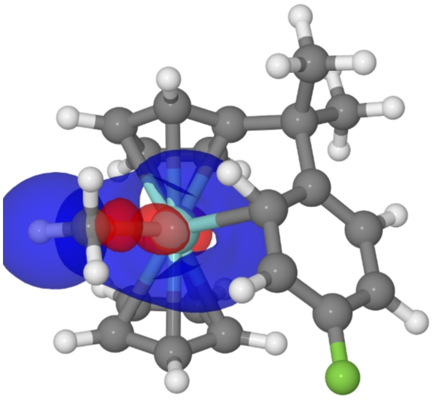

8

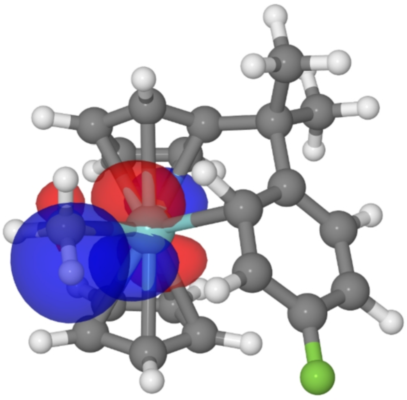

9

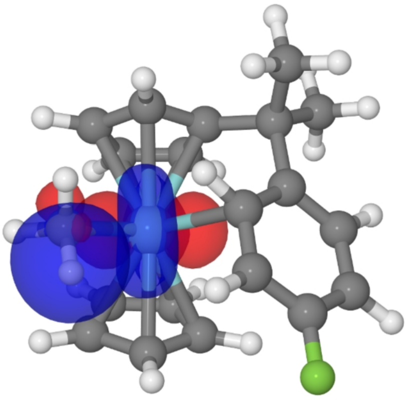

10

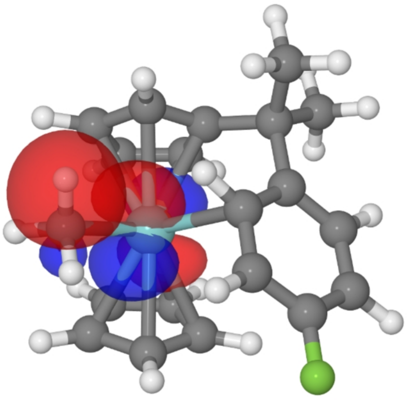

11

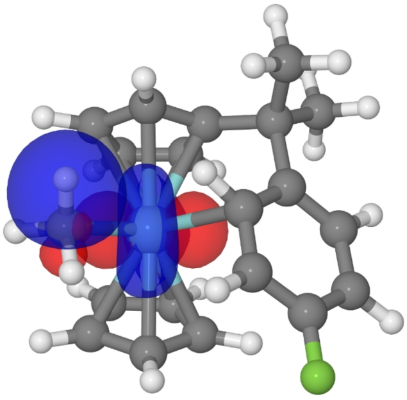

12

	Orbitals	E(2P)
1	$\sigma_{CC} = 0.711(sp^{1.74})_{C7} – 0.703(sp^{2.06})_{C8} \rightarrow LV_{Zr} = sd^{59.09}$	2.74
2	$\sigma_{CC} = 0.711(sp^{1.74})_{C7} – 0.703(sp^{2.06})_{C8} \rightarrow LV_{Zr} = sd^{3.77}$	3.77
3	$\pi_{CC} = 0.748(p)_{C7} – 0.664(p)_{C8} \rightarrow LV_{Zr} = sd^{59.09}$	7.48
4	$\pi_{CC} = 0.748(p)_{C7} – 0.664(p)_{C8} \rightarrow LV_{Zr} = sd^{3.77}$	5.26
5	$\sigma_{CC} = 0.711(sp^{1.84})_{C12} – 0.703(sp^{1.73})_{C13} \rightarrow LV_{Zr} = sd^{59.09}$	3.62
6	$\sigma_{CH} = 0.795(sp^{2.57})_{C7} – 0.607(s)_{H24} \rightarrow LV_{Zr} = sd^{59.09}$	5.28
7	$\sigma_{CH} = 0.795(sp^{2.57})_{C7} – 0.607(s)_{H24} \rightarrow LV_{Zr} = sd^{3.77}$	4.87
8	$\sigma_{CH} = 0.788(sp^{3.20})_{C21} – 0.616(s)_{H42} \rightarrow LV_{Zr} = sd^{3.77}$	2.28
9	$\sigma_{CH} = 0.780(sp^{3.04})_{C21} – 0.625(s)_{H43} \rightarrow LV_{Zr} = p^{0.76}d^{99.99}$	2.04
10	$\sigma_{CH} = 0.780(sp^{3.04})_{C21} – 0.625(s)_{H43} \rightarrow LV_{Zr} = sd^{10.84}$	3.22
11	$\sigma_{CH} = 0.781(sp^{2.99})_{C21} – 0.625(s)_{H44} \rightarrow LV_{Zr} = p^{0.76}d^{99.99}$	2.18
12	$\sigma_{CH} = 0.781(sp^{2.99})_{C21} – 0.625(s)_{H44} \rightarrow LV_{Zr} = sd^{10.84}$	2.40

Natural Resonance Theory:

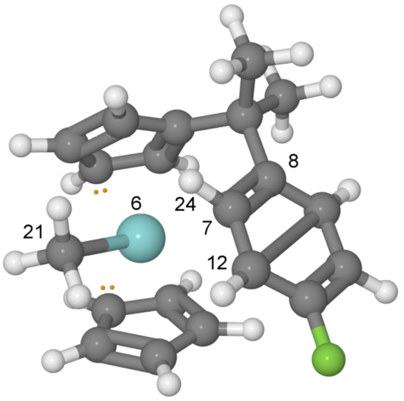

1

Wgt=20.94%;
rhoNL=5.79005;
D(0)=0.10080

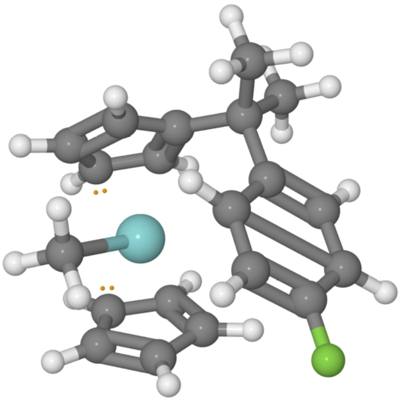

2

Wgt=15.21%;
rhoNL=5.75369;
D(0)=0.10048

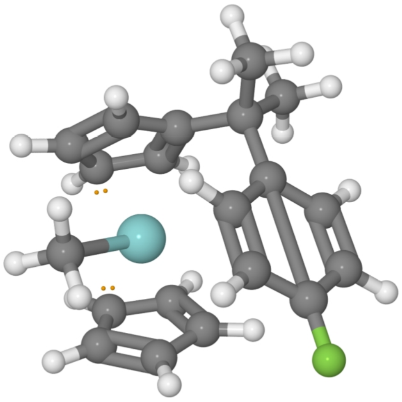

3

Wgt=14.78%;
rhoNL=5.73774;
D(0)=0.10034

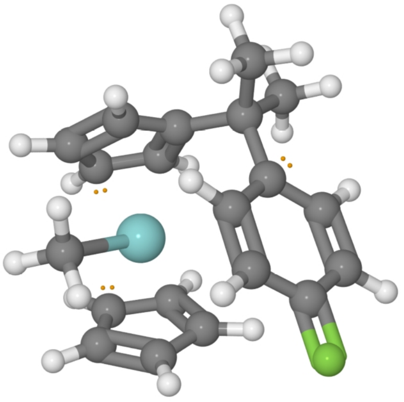

4

Wgt=8.19%;
rhoNL=5.91588;
D(0)=0.10188

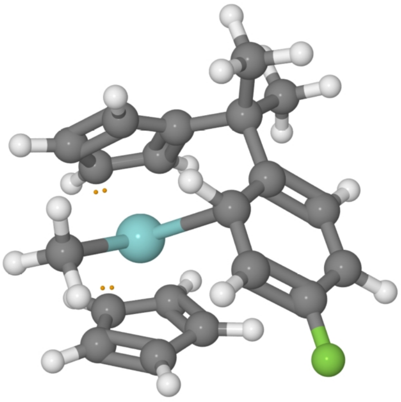

5

Wgt=6.18%;
rhoNL=5.89144;
D(0)=0.10167

Natural Localised Molecular Orbitals (NLMO):

Only contributions over 1% are reported.

NLMO / Occupancy / Percent from Parent NBO / Atomic Hybrid Contributions

Resonance structure **1**:
C-H interaction:

```
 61. (2.00000)  97.2357% BD ( 1) C  7- H 24
                  1.277% Zr  6 s( 29.36%)p 0.02(  0.71%)d 2.38( 69.92%)
                 61.490%  C  7 s( 26.34%)p 2.79( 73.58%)d 0.00(  0.08%)
                 35.778%  H 24 s( 99.97%)p 0.00(  0.03%)
```

C-C interaction:

```
 58. (2.00000)  98.3233% BD ( 1) C  7- C  8
                 49.819%  C  7 s( 32.76%)p 2.05( 67.19%)d 0.00(  0.05%)
                 48.651%  C  8 s( 29.17%)p 2.43( 70.79%)d 0.00(  0.04%)
 59. (2.00000)  82.8627% BD ( 2) C  7- C  8
                  3.553% Zr  6 s( 18.51%)p 0.04(  0.83%)d 4.36( 80.66%)
                 46.653%  C  7 s(  0.75%)p99.99( 99.22%)d 0.05(  0.04%)
                 36.228%  C  8 s(  0.09%)p99.99( 99.86%)d 0.52(  0.05%)
                  4.910%  C  9 s(  0.01%)p 1.00( 99.67%)d 0.00(  0.32%)
                  1.274%  C 10 s(  0.12%)p99.99( 99.53%)d 2.89(  0.35%)
                  1.520%  C 11 s(  0.26%)p99.99( 99.49%)d 0.96(  0.25%)
                  4.642%  C 12 s(  0.03%)p99.99( 99.65%)d11.52(  0.32%)
```

Zr-Me interaction:

```
 57. (2.00000)  97.1423% BD ( 1)Zr  6- C 21
                 20.303% Zr  6 s( 11.96%)p 0.00(  0.05%)d 7.36( 88.00%)
                 76.851%  C 21 s( 26.72%)p 2.74( 73.28%)d 0.00(  0.01%)
```

Resonance structure **2**:
C-H interaction:

```
 61. (2.00000)  97.2434% BD ( 1) C  7- H 24
                  1.313% Zr  6 s( 29.83%)p 0.02(  0.70%)d 2.33( 69.47%)
                 61.518%  C  7 s( 26.42%)p 2.78( 73.49%)d 0.00(  0.08%)
                 35.751%  H 24 s( 99.97%)p 0.00(  0.03%)
```

C-C interaction:

```
 59. (2.00000)  67.4098% BD ( 1) C  7- C 10
                  3.151% Zr  6 s( 18.00%)p 0.06(  1.08%)d 4.49( 80.91%)
                 41.072%  C  7 s(  0.67%)p99.99( 99.31%)d 0.02(  0.01%)
                  7.223%  C  8 s(  0.14%)p99.99( 99.58%)d 1.91(  0.28%)
                  7.459%  C  9 s(  0.04%)p99.99( 99.79%)d 4.49(  0.17%)
                 26.392%  C 10 s(  0.04%)p99.99( 99.95%)d 0.51(  0.02%)
                  6.717%  C 11 s(  0.12%)p99.99( 99.61%)d 2.14(  0.27%)
                  7.088%  C 12 s(  0.03%)p99.99( 99.75%)d 8.24(  0.22%)
```

Zr-Me interaction:

```
  57. (2.00000)  97.1426% BD ( 1)Zr  6- C 21
                 20.299% Zr  6 s( 11.96%)p 0.00(  0.05%)d 7.36( 88.00%)
                 76.856%  C 21 s( 26.72%)p 2.74( 73.28%)d 0.00(  0.01%)
```

Resonance structure **3**:

C-H interaction:

```
  61. (2.00000)  97.2355% BD ( 1) C  7- H 24
                  1.273% Zr  6 s( 29.41%)p 0.02(  0.72%)d 2.38( 69.87%)
                 61.486%  C  7 s( 26.34%)p 2.79( 73.58%)d 0.00(  0.08%)
                 35.779%  H 24 s( 99.97%)p 0.00(  0.03%)
```

C-C interaction:

```
  59. (2.00000)  98.4683% BD ( 1) C  7- C 12
                 49.852%  C  7 s( 31.67%)p 2.16( 68.28%)d 0.00(  0.05%)
                 48.747%  C 12 s( 33.24%)p 2.01( 66.71%)d 0.00(  0.04%)
  60. (2.00000)  83.9291% BD ( 2) C  7- C 12
                  2.815% Zr  6 s( 22.39%)p 0.04(  0.84%)d 3.43( 76.77%)
                 47.709%  C  7 s(  0.68%)p99.99( 99.29%)d 0.04(  0.03%)
                  4.692%  C  8 s(  0.16%)p99.99( 99.48%)d 2.21(  0.36%)
                  1.628%  C  9 s(  0.04%)p99.99( 99.79%)d 4.51(  0.18%)
                  1.094%  C 10 s(  0.10%)p99.99( 99.59%)d 2.95(  0.31%)
                  5.136%  C 11 s(  0.02%)p99.99( 99.60%)d25.41(  0.38%)
                 36.253%  C 12 s(  0.03%)p99.99( 99.93%)d 1.38(  0.04%)
```

Zr-Me interaction:

```
  57. (2.00000)  97.1421% BD ( 1)Zr  6- C 21
                 20.304% Zr  6 s( 11.95%)p 0.00(  0.05%)d 7.36( 88.00%)
                 76.851%  C 21 s( 26.72%)p 2.74( 73.28%)d 0.00(  0.01%)
```

Resonance structure **4**:

C-H interaction:

```
  61. (2.00000)  97.2346% BD ( 1) C  7- H 24
                  1.271% Zr  6 s( 29.47%)p 0.02(  0.72%)d 2.37( 69.82%)
                 61.485%  C  7 s( 26.34%)p 2.79( 73.57%)d 0.00(  0.08%)
                 35.779%  H 24 s( 99.97%)p 0.00(  0.03%)
```

C-C interaction:

```
  59. (2.00000)  98.4682% BD ( 1) C  7- C 12
                 49.850%  C  7 s( 31.69%)p 2.15( 68.27%)d 0.00(  0.05%)
                 48.748%  C 12 s( 33.24%)p 2.01( 66.72%)d 0.00(  0.04%)
  60. (2.00000)  84.5013% BD ( 2) C  7- C 12
                  2.722% Zr  6 s( 22.52%)p 0.04(  0.83%)d 3.40( 76.66%)
                 46.643%  C  7 s(  0.68%)p99.99( 99.29%)d 0.04(  0.03%)
                  3.935%  C  8 s(  0.17%)p99.99( 99.42%)d 2.45(  0.41%)
                  1.662%  C  9 s(  0.04%)p99.99( 99.81%)d 3.51(  0.15%)
                  0.778%  C 10 s(  0.15%)p99.99( 99.34%)d 3.27(  0.50%)
                  5.237%  C 11 s(  0.01%)p99.99( 99.53%)d43.86(  0.46%)
                 37.877%  C 12 s(  0.03%)p99.99( 99.92%)d 1.30(  0.04%)
```

Zr-Me interaction:

```
 57. (2.00000)  97.1418% BD ( 1)Zr  6- C 21
                 20.304% Zr  6 s( 11.95%)p 0.00(  0.05%)d 7.37( 88.01%)
                 76.850%  C 21 s( 26.72%)p 2.74( 73.28%)d 0.00(  0.01%)
```

Resonance structure **5**:

C-H interaction:

```
 61. (2.00000)  97.2504% BD ( 1) C  7- H 24
                  1.301% Zr  6 s( 29.66%)p 0.02(  0.70%)d 2.35( 69.64%)
                 61.511%  C  7 s( 26.41%)p 2.78( 73.51%)d 0.00(  0.08%)
                 35.763%  H 24 s( 99.97%)p 0.00(  0.03%)
```

C-C interaction:

```
 59. (2.00000)  98.3088% BD ( 1) C  7- C  8
                 49.819%  C  7 s( 32.83%)p 2.04( 67.12%)d 0.00(  0.05%)
                 48.641%  C  8 s( 29.23%)p 2.42( 70.73%)d 0.00(  0.04%)
 60. (2.00000)  98.4623% BD ( 1) C  7- C 12
                 49.856%  C  7 s( 31.69%)p 2.15( 68.26%)d 0.00(  0.05%)
                 48.739%  C 12 s( 33.31%)p 2.00( 66.65%)d 0.00(  0.04%)
```

Zr-Me interaction:

```
 58. (2.00000)  97.1432% BD ( 1)Zr  6- C 21
                 20.295% Zr  6 s( 11.96%)p 0.00(  0.05%)d 7.36( 87.99%)
                 76.861%  C 21 s( 26.71%)p 2.74( 73.29%)d 0.00(  0.01%)
```

Non-Covalent Interactions (NCI)

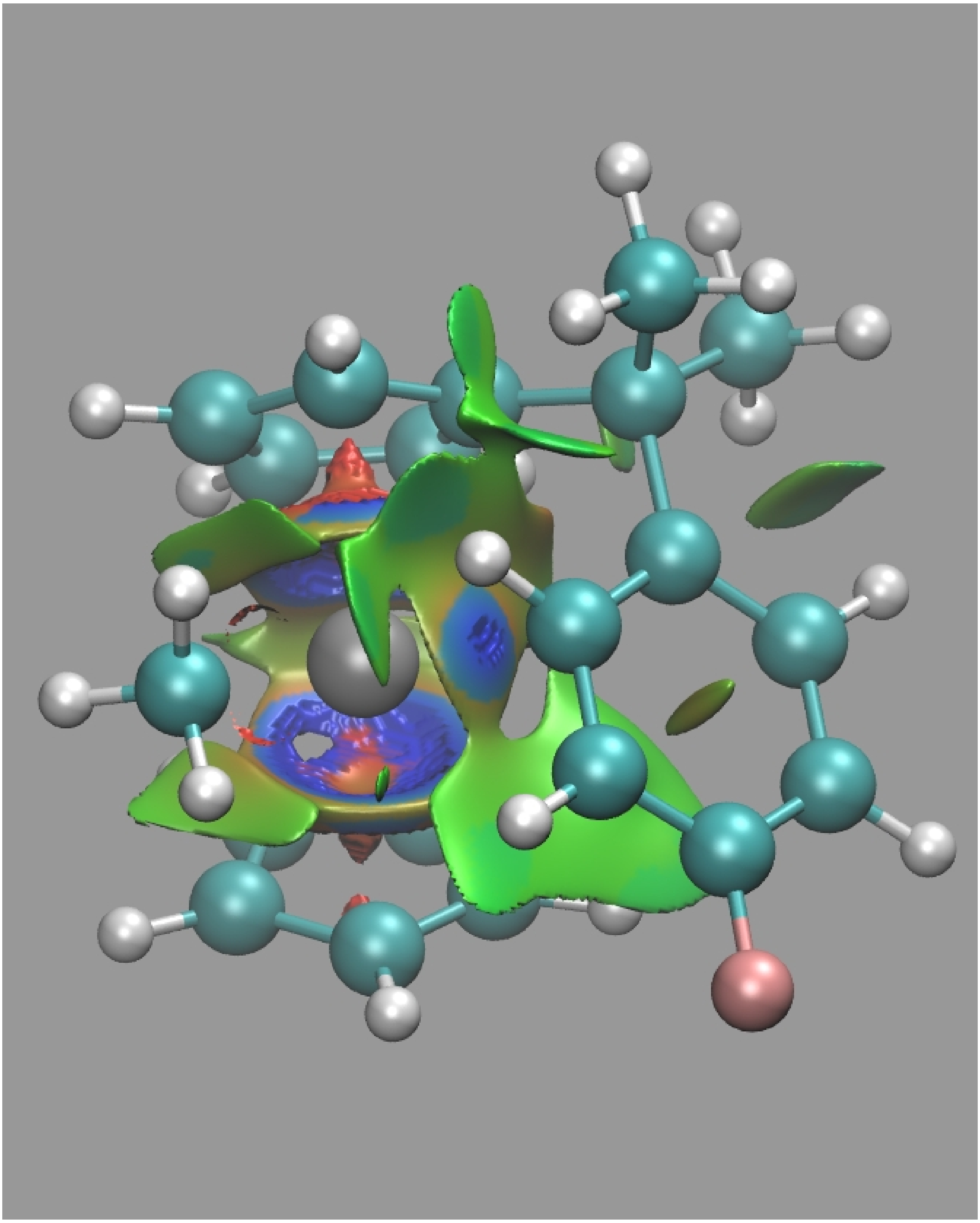

5B-PBE-D3

Bader:

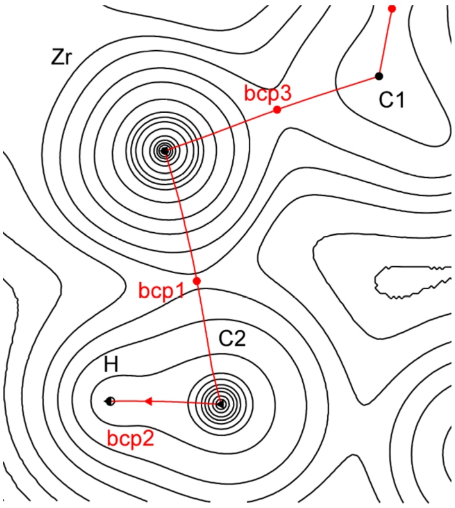

Electrondensity

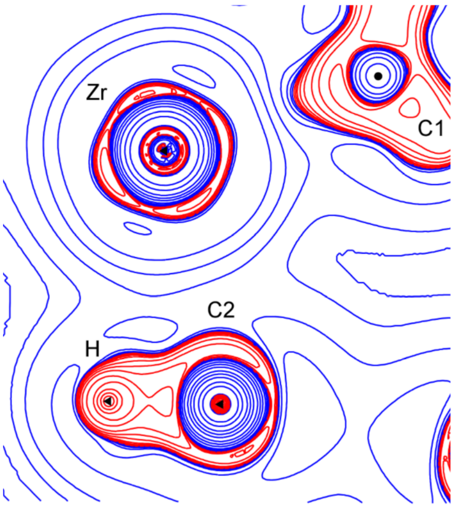

Laplacian

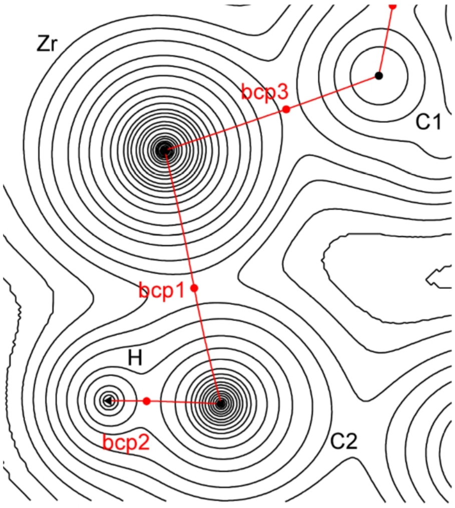

Virial

	$\varrho(\mathbf{r})$	$\nabla^2\varrho(\mathbf{r})$
bcp1	0.03817	-0.02359
bcp2	0.26054	0.20366
bcp3	0.09531	-0.01180

NBO:

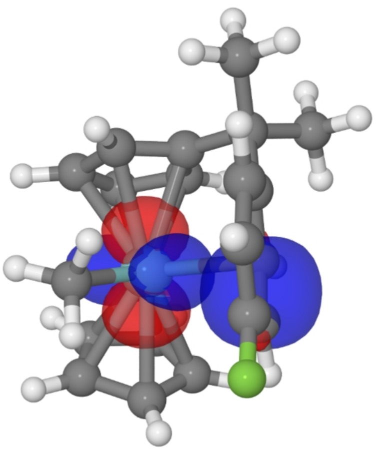

1

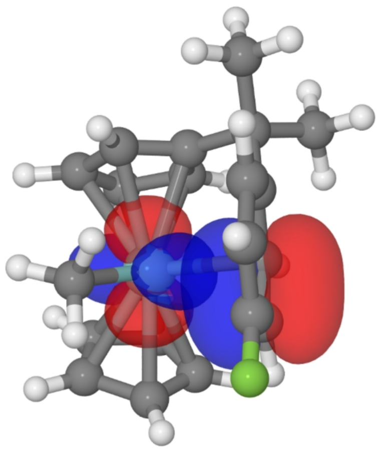

2

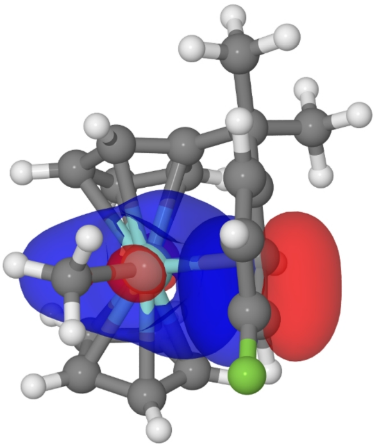

3

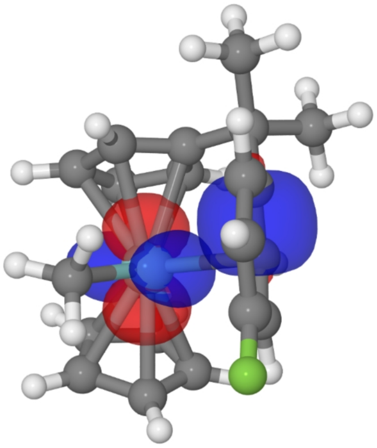

4

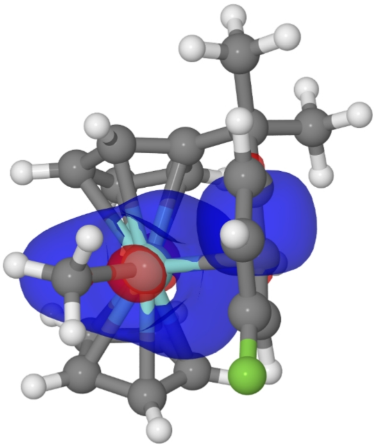

5

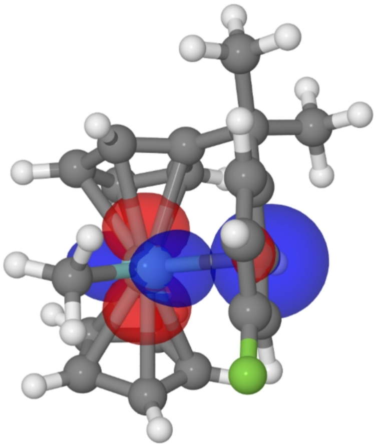

6

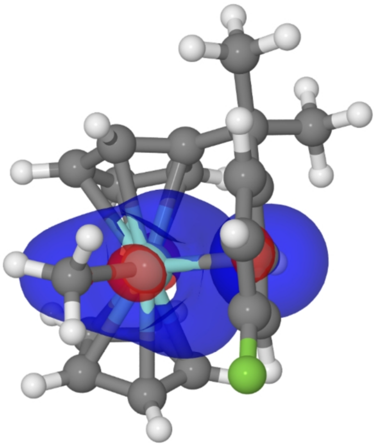

7

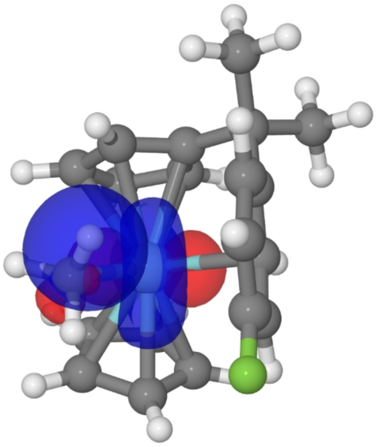

8

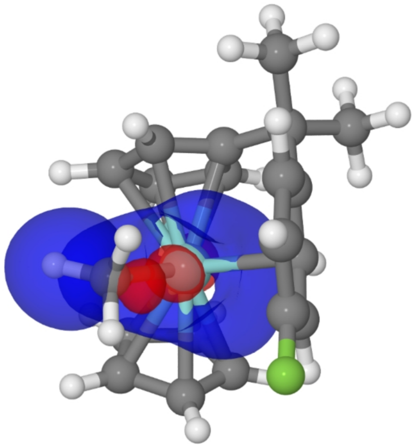

9

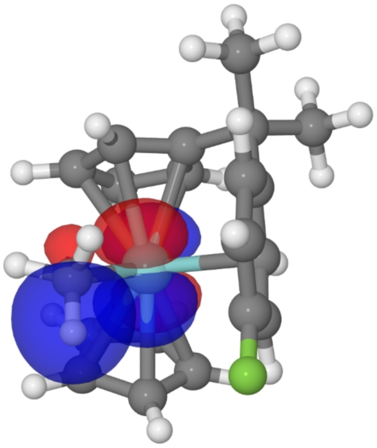

10

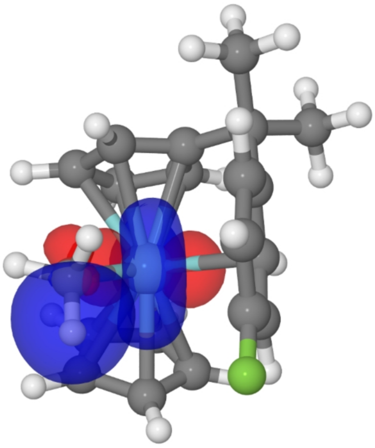

11

	Orbitals	E(2P)
1	σ_{CC} = 0.711$(sp^{1.84})_{C7}$ – 0.702$(sp^{1.73})_{C8}$ → LV_{Zr} = $sd^{90.66}$	3.74
2	π_{CC} = 0.752$(p)_{C7}$ – 0.659$(p)_{C8}$ → LV_{Zr} = $sd^{90.66}$	8.60
3	π_{CC} = 0.752$(p)_{C7}$ – 0.659$(p)_{C8}$ → LV_{Zr} = $sd^{0.39}$	5.17
4	σ_{CC} = 0.713$(sp^{1.71})_{C7}$ – 0.701$(sp^{2.07})_{C12}$ → LV_{Zr} = $sd^{90.66}$	2.73
5	σ_{CC} = 0.713$(sp^{1.71})_{C7}$ – 0.701$(sp^{2.07})_{C12}$ → LV_{Zr} = $sd^{0.39}$	3.32
6	σ_{CH} = 0.790$(sp^{2.65})_{C7}$ – 0.614$(s)_{H26}$ → LV_{Zr} = $sd^{90.66}$	3.34
7	σ_{CH} = 0.790$(sp^{2.65})_{C7}$ – 0.614$(s)_{H26}$ → LV_{Zr} = $sd^{0.39}$	8.44
8	σ_{CH} = 0.780$(sp^{3.03})_{C21}$ – 0.626$(s)_{H37}$ → LV_{Zr} = $sd^{6.49}$	4.26
9	σ_{CH} = 0.787$(sp^{3.18})_{C21}$ – 0.618$(s)_{H38}$ → LV_{Zr} = $sd^{0.39}$	2.28
10	σ_{CH} = 0.781$(sp^{2.96})_{C21}$ – 0.624$(s)_{H39}$ → LV_{Zr} = $sp^{0.24}d^{99.99}$	2.38
11	σ_{CH} = 0.781$(sp^{2.96})_{C21}$ – 0.624$(s)_{H39}$ → LV_{Zr} = $sd^{6.49}$	2.57

Natural Resonance Theory:

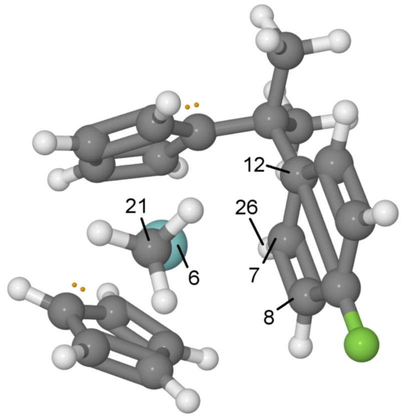

1

Wgt=13.08%;
rhoNL=5.78668;
D(0)=0.10077

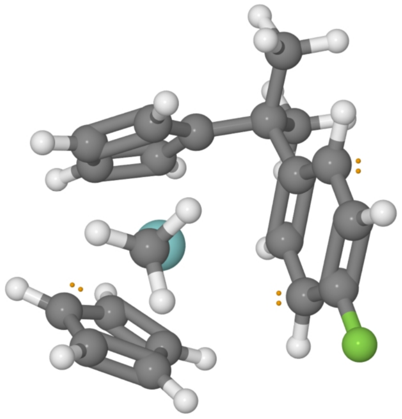

2

Wgt=12.32%;
rhoNL=6.23562;
D(0)=0.10460

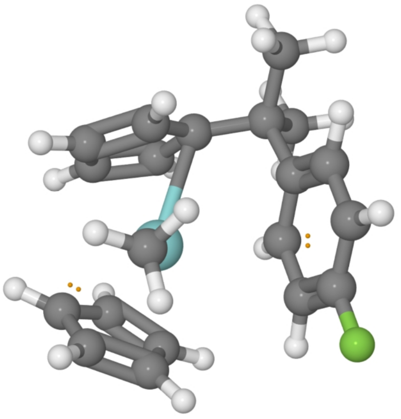

3

Wgt=9.62%;
rhoNL=5.81551;
D(0)=0.10102

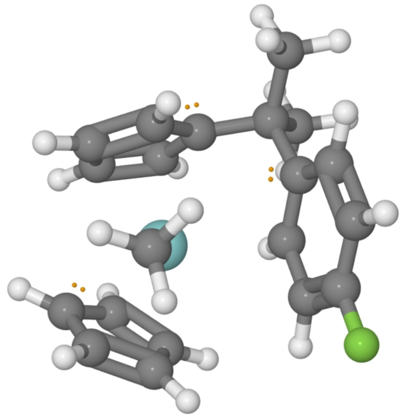

4

Wgt=8.58%;
rhoNL=6.17259;
D(0)=0.10407

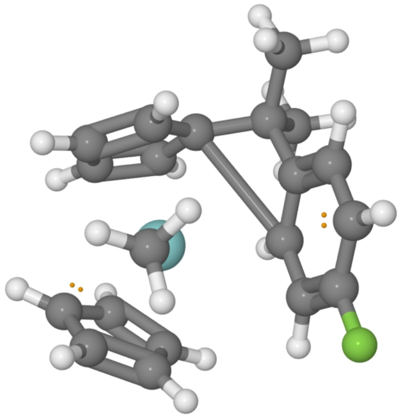

5

Wgt=5.87%;
rhoNL=6.09265;
D(0)=0.10340

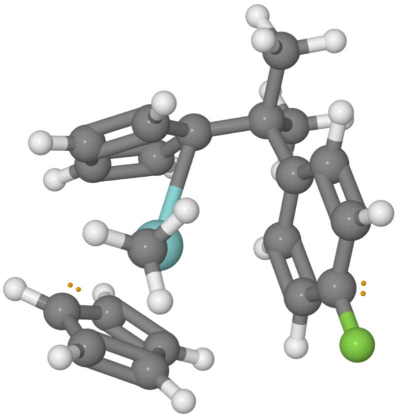

6

Wgt=5.16%;
rhoNL=5.89154;
D(0)=0.10167

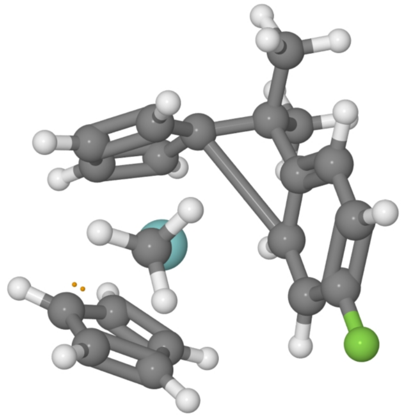

7

Wgt=5.08%; rhoNL=6.72636; D(0)=0.10864

Natural Localised Molecular Orbitals (NLMO):

Only contributions over 1% are reported.

NLMO / Occupancy / Percent from Parent NBO / Atomic Hybrid Contributions

Resonance structure **1**:
C-H interaction:

```
 61. (2.00000)  96.6353% BD ( 1) C  7- H 26
                  1.977% Zr  6 s( 26.45%)p 0.02(  0.51%)d 2.76( 73.04%)
                 60.348%  C  7 s( 25.97%)p 2.85( 73.95%)d 0.00(  0.09%)
                 36.339%  H 26 s( 99.97%)p 0.00(  0.03%)
```

C-C interaction:

```
 58. (2.00000)  98.4924% BD ( 1) C  7- C  8
                 49.931%  C  7 s( 31.86%)p 2.14( 68.09%)d 0.00(  0.05%)
                 48.689%  C  8 s( 33.18%)p 2.01( 66.78%)d 0.00(  0.04%)
 59. (2.00000)  84.8744% BD ( 2) C  7- C  8
                  2.805% Zr  6 s( 24.10%)p 0.04(  0.85%)d 3.11( 75.05%)
                 49.111%  C  7 s(  0.77%)p99.99( 99.20%)d 0.03(  0.02%)
                 35.786%  C  8 s(  0.03%)p99.99( 99.93%)d 1.24(  0.04%)
                  4.498%  C  9 s(  0.01%)p99.99( 99.57%)d31.31(  0.41%)
                  1.075%  C 10 s(  0.07%)p99.99( 99.64%)d 3.79(  0.28%)
                  1.443%  C 11 s(  0.15%)p99.99( 99.63%)d 1.44(  0.22%)
                  4.523%  C 12 s(  0.23%)p99.99( 99.43%)d 1.45(  0.33%)
```

Zr-Me interaction:

```
 57. (2.00000)  97.2039% BD ( 1)Zr  6- C 21
                 21.217% Zr  6 s( 11.54%)p 0.00(  0.03%)d 7.67( 88.44%)
                 76.002%  C 21 s( 26.52%)p 2.77( 73.47%)d 0.00(  0.01%)
```

Resonance structure **2**:
C-H interaction:

```
 62. (2.00000)  96.6275% BD ( 1) C  7- H 26
                  2.035% Zr  6 s( 26.39%)p 0.02(  0.49%)d 2.77( 73.11%)
                 60.392%  C  7 s( 26.03%)p 2.84( 73.89%)d 0.00(  0.09%)
                 36.280%  H 26 s( 99.97%)p 0.00(  0.03%)
```

C-C interaction:

```
  60. (2.00000)  98.4330% BD ( 1) C  7- C 12
                  50.111%  C  7 s( 33.09%)p 2.02( 66.87%)d 0.00(  0.04%)
                  48.471%  C 12 s( 29.10%)p 2.43( 70.86%)d 0.00(  0.04%)
  61. (2.00000)  82.3724% BD ( 2) C  7- C 12
                   3.297% Zr  6 s( 18.53%)p 0.04(  0.66%)d 4.36( 80.81%)
                  50.222%  C  7 s(  0.73%)p99.99( 99.25%)d 0.02(  0.02%)
                   5.011%  C  8 s(  0.01%)p99.99( 99.65%)d26.43(  0.33%)
                   2.029%  C  9 s(  0.14%)p99.99( 99.69%)d 1.27(  0.17%)
                   1.996%  C 10 s(  0.07%)p99.99( 99.74%)d 2.98(  0.20%)
                   3.901%  C 11 s(  0.00%)p 1.00( 99.59%)d 0.00(  0.41%)
                  32.233%  C 12 s(  0.12%)p99.99( 99.83%)d 0.39(  0.05%)
```

Zr-Me interaction:

```
  58. (2.00000)  97.0476% BD ( 1)Zr  6- C 21
                  21.231% Zr  6 s( 11.57%)p 0.00(  0.03%)d 7.64( 88.41%)
                  75.850%  C 21 s( 26.54%)p 2.77( 73.45%)d 0.00(  0.01%)
```

Resonance structure **3**:

C-H interaction:

```
  61. (2.00000)  96.6561% BD ( 1) C  7- H 26
                   2.009% Zr  6 s( 27.33%)p 0.02(  0.50%)d 2.64( 72.17%)
                  60.360%  C  7 s( 26.05%)p 2.84( 73.86%)d 0.00(  0.09%)
                  36.315%  H 26 s( 99.97%)p 0.00(  0.03%)
```

C-C interaction:

```
  59. (2.00000)  98.4841% BD ( 1) C  7- C  8
                  49.937%  C  7 s( 31.84%)p 2.14( 68.12%)d 0.00(  0.05%)
                  48.684%  C  8 s( 33.13%)p 2.02( 66.83%)d 0.00(  0.04%)
  60. (2.00000)  98.4209% BD ( 1) C  7- C 12
                  50.119%  C  7 s( 33.16%)p 2.01( 66.79%)d 0.00(  0.04%)
                   0.274%  C  8 s(  2.00%)p48.31( 96.66%)d 0.67(  1.34%)
                   0.012%  C 10 s( 26.85%)p 2.58( 69.17%)d 0.15(  3.99%)
                   0.208%  C 11 s(  1.13%)p85.53( 96.83%)d 1.80(  2.03%)
                  48.456%  C 12 s( 29.09%)p 2.44( 70.87%)d 0.00(  0.04%)
```

Zr-Me interaction:

```
  58. (2.00000)  97.2028% BD ( 1)Zr  6- C 21
                  21.196% Zr  6 s( 11.51%)p 0.00(  0.03%)d 7.69( 88.47%)
                  76.022%  C 21 s( 26.49%)p 2.78( 73.51%)d 0.00(  0.01%)
```

Resonance structure **4**:

NLMO algorithm failed to converge

Resonance structure **5**:

C-H interaction:

```
  61. (2.00000)  96.6542% BD ( 1) C  7- H 26
                   2.042% Zr  6 s( 26.78%)p 0.02(  0.50%)d 2.71( 72.71%)
                  60.406%  C  7 s( 26.10%)p 2.83( 73.82%)d 0.00(  0.09%)
                  36.276%  H 26 s( 99.97%)p 0.00(  0.03%)
```

C-C interaction:
```
 52. (2.00000)  53.6188% BD ( 1) C  3- C  7
                  1.257%  C  1 s(  0.37%)p99.99( 99.43%)d 0.52(  0.19%)
                  4.530%  C  2 s(  0.72%)p99.99( 99.03%)d 0.35(  0.25%)
                 22.725%  C  3 s(  0.41%)p99.99( 99.55%)d 0.08(  0.03%)
                  4.649%  C  4 s(  0.49%)p99.99( 99.24%)d 0.55(  0.27%)
                  1.097%  C  5 s(  0.27%)p99.99( 99.49%)d 0.93(  0.25%)
                  2.752% Zr  6 s( 21.15%)p 0.06(  1.26%)d 3.67( 77.60%)
                 33.110%  C  7 s(  0.51%)p99.99( 99.48%)d 0.02(  0.01%)
                  5.867%  C  8 s(  0.27%)p99.99( 99.59%)d 0.50(  0.14%)
                  1.498%  C  9 s(  0.04%)p99.99( 99.43%)d13.78(  0.53%)
                  8.342%  C 10 s(  0.00%)p 1.00( 99.99%)d 0.00(  0.01%)
                  1.126%  C 11 s(  0.71%)p99.99( 98.58%)d 1.01(  0.71%)
                 12.241%  C 12 s(  0.62%)p99.99( 99.26%)d 0.20(  0.13%)
```

Zr-Me interaction:
```
 58. (2.00000)  97.1872% BD ( 1)Zr  6- C 21
                 21.192% Zr  6 s( 11.55%)p 0.00(  0.03%)d 7.66( 88.42%)
                 76.013%  C 21 s( 26.52%)p 2.77( 73.47%)d 0.00(  0.01%)
```

Resonance structure **6**:
C-H interaction:
```
 62. (2.00000)  96.6292% BD ( 1) C  7- H 26
                  1.959% Zr  6 s( 27.11%)p 0.02(  0.50%)d 2.67( 72.38%)
                 60.306%  C  7 s( 25.91%)p 2.86( 74.00%)d 0.00(  0.09%)
                 36.355%  H 26 s( 99.97%)p 0.00(  0.03%)
```

C-C interaction:
```
 59. (2.00000)  98.4920% BD ( 1) C  7- C  8
                 49.935%  C  7 s( 31.83%)p 2.14( 68.13%)d 0.00(  0.05%)
                 48.688%  C  8 s( 33.12%)p 2.02( 66.84%)d 0.00(  0.04%)
 60. (2.00000)  85.1178% BD ( 2) C  7- C  8
                  2.917% Zr  6 s( 25.34%)p 0.03(  0.81%)d 2.92( 73.85%)
                 49.687%  C  7 s(  0.86%)p99.99( 99.12%)d 0.02(  0.02%)
                 35.482%  C  8 s(  0.03%)p99.99( 99.92%)d 1.21(  0.04%)
                  4.218%  C  9 s(  0.01%)p99.99( 99.54%)d43.43(  0.45%)
                  1.137%  C 11 s(  0.20%)p99.99( 99.51%)d 1.49(  0.29%)
                  4.928%  C 12 s(  0.20%)p99.99( 99.49%)d 1.48(  0.30%)
```

Zr-Me interaction:
```
 58. (2.00000)  97.2028% BD ( 1)Zr  6- C 21
                 21.199% Zr  6 s( 11.51%)p 0.00(  0.03%)d 7.69( 88.46%)
                 76.018%  C 21 s( 26.49%)p 2.78( 73.51%)d 0.00(  0.01%)
```

Resonance structure **7**:
NLMO algorithm failed to converge

Non-Covalent Interactions (NCI)

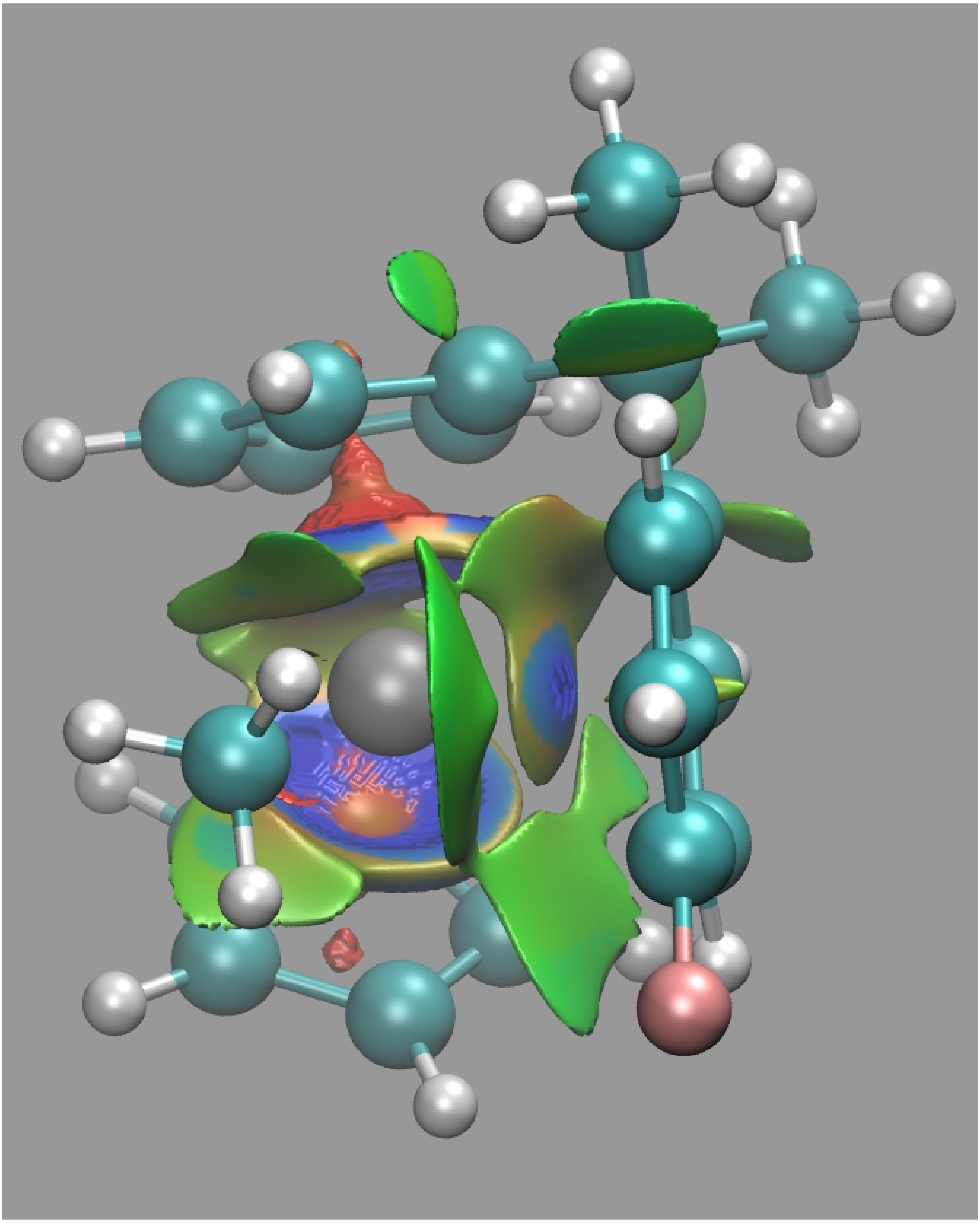

6A-PBE-D3

Bader:

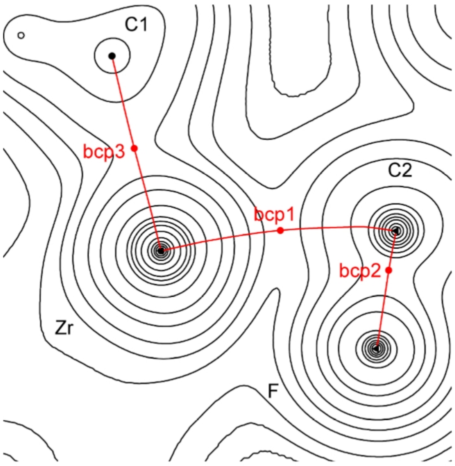

Electrondensity

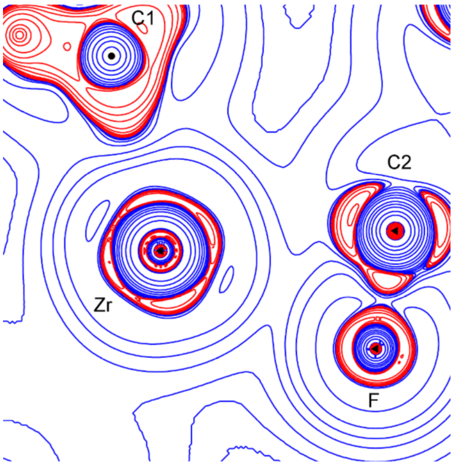

Laplacian

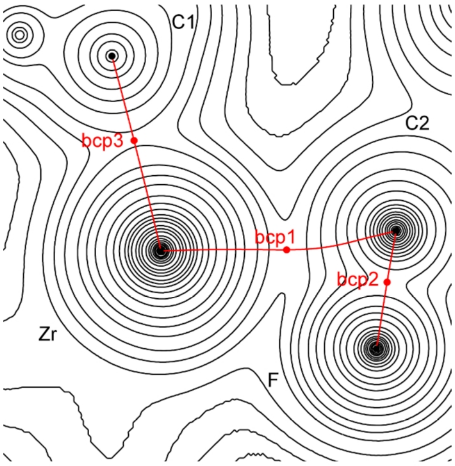

Virial

	ϱ(**r**)	∇^2ϱ(**r**)
bcp1	0.02865	-0.02061
bcp2	0.25939	-0.04959
bcp3	0.09436	-0.01096

NBO:

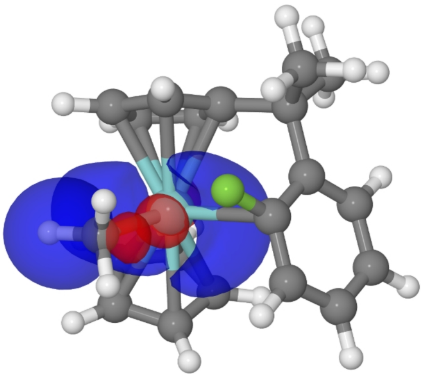

1

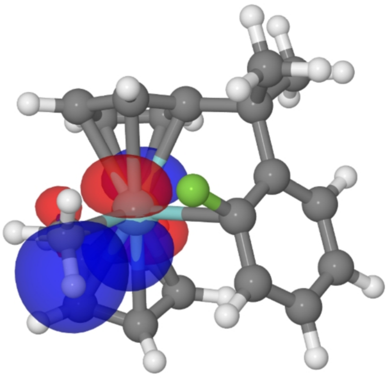

2

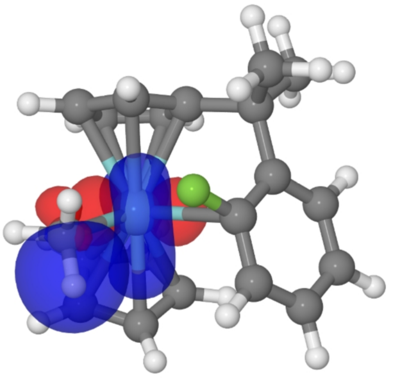

3

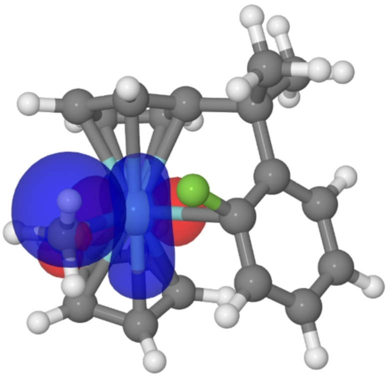

4

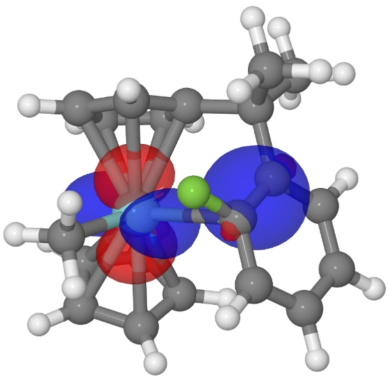

5

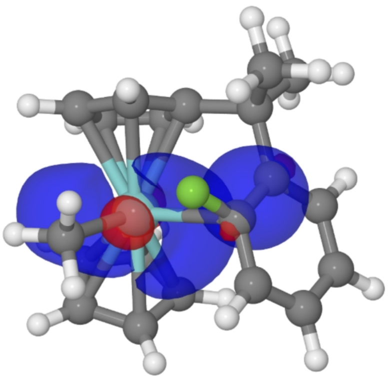

6

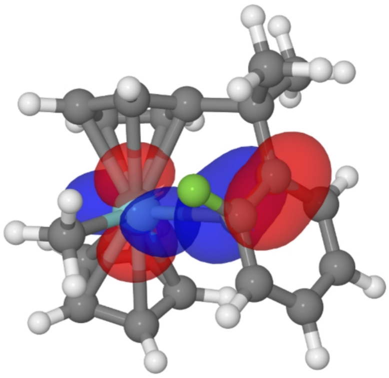

7

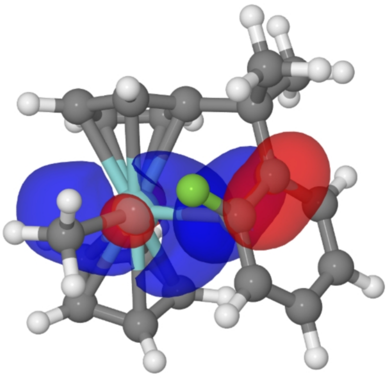

8

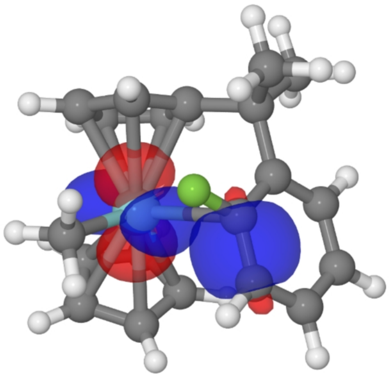

9

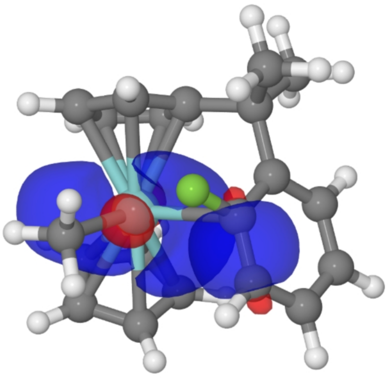

10

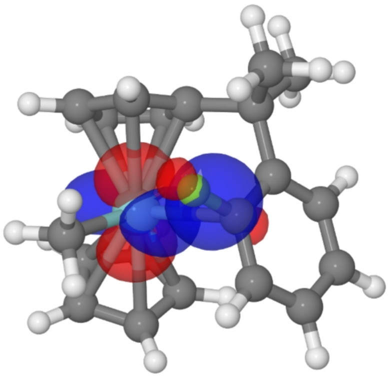

11

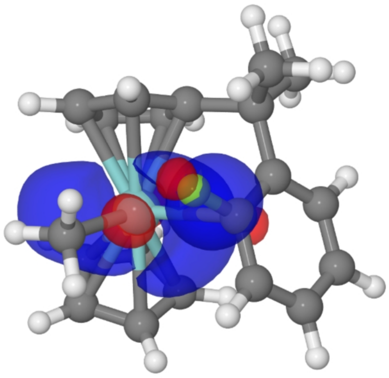

12

	Orbitals	E(2P)
1	σ_{CH} = 0.784$(sp^{3.27})_{C12}$ – 0.620$(s)_{H13}$ → LV_{Zr} = $sd^{0.49}$	2.65
2	σ_{CH} = 0.780$(sp^{2.97})_{C12}$ – 0.626$(s)_{H14}$ → LV_{Zr} = $p^{2.44}d^{99.9}$	2.18
3	σ_{CH} = 0.780$(sp^{2.97})_{C12}$ – 0.626$(s)_{H14}$ → LV_{Zr} = $sd^{4.33}$	3.39
4	σ_{CH} = 0.782$(sp^{2.91})_{C12}$ – 0.624$(s)_{H15}$ → LV_{Zr} = $sd^{4.33}$	2.82
5	σ_{CC} = 0.705$(sp^{2.16})_{C30}$ – 0.710$(sp^{1.52})_{C31}$ → LV_{Zr} = $sp^{0.12}d^{99.99}$	2.86
6	σ_{CC} = 0.705$(sp^{2.16})_{C30}$ – 0.710$(sp^{1.52})_{C31}$ → LV_{Zr} = $sd^{0.49}$	5.36
7	π_{CH} = 0.674$(p)_{C30}$ – 0.739$(p)_{C31}$ → LV_{Zr} = $sp^{0.12}d^{99.99}$	5.05
8	π_{CH} = 0.674$(p)_{C30}$ – 0.739$(p)_{C31}$ → LV_{Zr} = $sd^{0.49}$	5.10
9	σ_{CH} = 0.713$(sp^{1.59})_{C31}$ – 0.701$(sp^{1.91})_{C23}$ → LV_{Zr} = $sp^{0.12}d^{99.99}$	3.34
10	σ_{CH} = 0.713$(sp^{1.59})_{C31}$ – 0.701$(sp^{1.91})_{C23}$ → LV_{Zr} = $sd^{0.49}$	2.69
11	σ_{CH} = 0.526$(sp^{3.69})_{C31}$ – 0.851$(sp^{2.22})_{F}$ → LV_{Zr} = $sp^{0.12}d^{99.99}$	2.68
12	σ_{CH} = 0.526$(sp^{3.69})_{C31}$ – 0.851$(sp^{2.22})_{F}$ → LV_{Zr} = $sd^{0.49}$	2.53

Natural Resonance Theory:

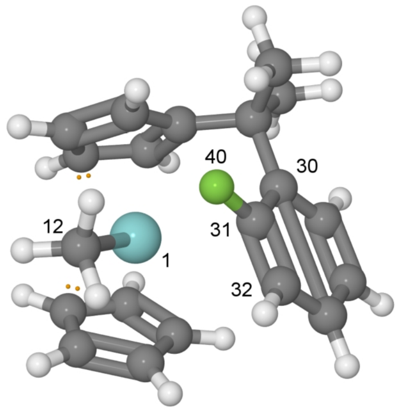 **1**	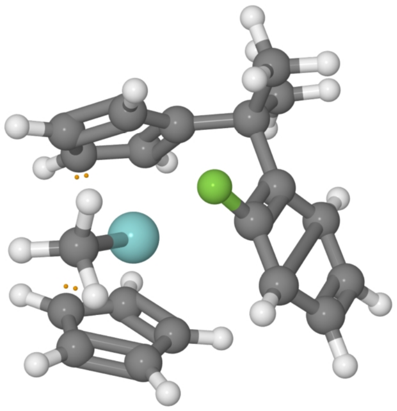 **2**	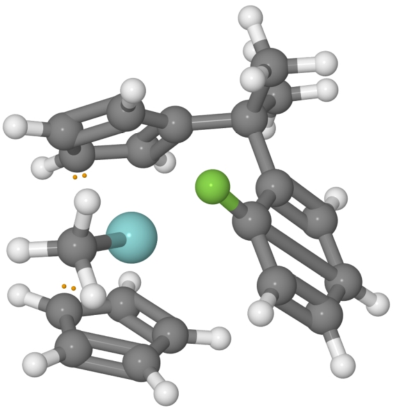 **3**
Wgt=19.64%; rhoNL=5.74281; D(0)=0.10038	Wgt=19.28%; rhoNL=5.75225; D(0)=0.10047	Wgt=14.59%; rhoNL=5.70233; D(0)=0.10003
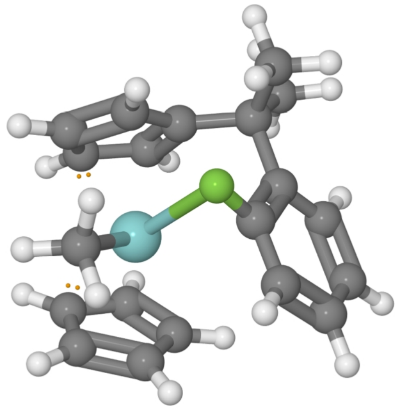 **4**		
Wgt=6.76%; rhoNL=5.37103; D(0)=0.09708		

Natural Localised Molecular Orbitals (NLMO):

Only contributions over 1% are reported.

NLMO / Occupancy / Percent from Parent NBO / Atomic Hybrid Contributions

Resonance structure **1**:
C-F interaction:
```
 84. (2.00000)  99.5807% BD ( 1) C 31- F 40
                27.470%  C 31 s( 22.24%)p 3.48( 77.42%)d 0.02(  0.34%)
                72.123%  F 40 s( 30.69%)p 2.26( 69.26%)d 0.00(  0.05%)
```

C-C interaction:
```
 82. (2.00000)  98.7304% BD ( 1) C 31- C 32
                50.228%  C 31 s( 36.23%)p 1.76( 63.74%)d 0.00(  0.02%)
                48.602%  C 32 s( 31.77%)p 2.15( 68.16%)d 0.00(  0.06%)
 83. (2.00000)  82.8105% BD ( 2) C 31- C 32
                 2.379% Zr  1 s( 20.26%)p 0.08(  1.53%)d 3.86( 78.21%)
                 5.240%  C 30 s(  0.33%)p99.99( 99.30%)d 1.12(  0.37%)
                45.265%  C 31 s(  0.78%)p99.99( 99.19%)d 0.03(  0.03%)
                37.561%  C 32 s(  0.03%)p99.99( 99.92%)d 1.50(  0.05%)
                 5.863%  C 33 s(  0.07%)p99.99( 99.64%)d 4.17(  0.29%)
                 1.256%  C 34 s(  0.09%)p99.99( 99.59%)d 3.52(  0.32%)
                 1.846%  C 35 s(  0.04%)p99.99( 99.79%)d 3.65(  0.16%)
```

Zr-Me interaction:
```
 45. (2.00000)  97.3783% BD ( 1)Zr  1- C 12
                20.616% Zr  1 s( 10.95%)p 0.00(  0.05%)d 8.13( 89.00%)
                76.775%  C 12 s( 26.35%)p 2.80( 73.65%)d 0.00(  0.01%)
```

Resonance structure **2**:
C-F interaction:
```
 83. (2.00000)  99.5812% BD ( 1) C 31- F 40
                27.472%  C 31 s( 22.23%)p 3.48( 77.43%)d 0.02(  0.34%)
                72.121%  F 40 s( 30.69%)p 2.26( 69.26%)d 0.00(  0.05%)
```

C-C interaction:
```
 79. (2.00000)  98.3074% BD ( 1) C 30- C 31
                48.869%  C 30 s( 28.60%)p 2.49( 71.33%)d 0.00(  0.07%)
                49.551%  C 31 s( 37.07%)p 1.70( 62.90%)d 0.00(  0.03%)
 80. (2.00000)  82.6531% BD ( 2) C 30- C 31
                 3.382% Zr  1 s( 17.01%)p 0.07(  1.26%)d 4.80( 81.72%)
                37.932%  C 30 s(  0.12%)p99.99( 99.82%)d 0.47(  0.06%)
                44.735%  C 31 s(  0.84%)p99.99( 99.12%)d 0.04(  0.04%)
                 4.881%  C 32 s(  0.10%)p99.99( 99.55%)d 3.54(  0.35%)
                 1.542%  C 33 s(  0.15%)p99.99( 99.61%)d 1.54(  0.24%)
                 1.293%  C 34 s(  0.09%)p99.99( 99.59%)d 3.47(  0.32%)
                 5.002%  C 35 s(  0.02%)p99.99( 99.66%)d14.19(  0.31%)
```

Zr-Me interaction:
45. (2.00000) 97.3774% BD (1)Zr 1- C 12
20.616% Zr 1 s(10.94%)p 0.00(0.05%)d 8.14(89.01%)
76.774% C 12 s(26.35%)p 2.79(73.64%)d 0.00(0.01%)

Resonance structure **3**:
C-F interaction:
84. (2.00000) 99.5959% BD (1) C 31- F 40
27.509% C 31 s(22.32%)p 3.46(77.34%)d 0.02(0.34%)
72.090% F 40 s(30.66%)p 2.26(69.29%)d 0.00(0.05%)

C-C interaction:
83. (2.00000) 66.2262% BD (1) C 31- C 34
2.897% Zr 1 s(16.51%)p 0.10(1.60%)d 4.96(81.88%)
7.885% C 30 s(0.25%)p99.99(99.45%)d 1.17(0.30%)
39.914% C 31 s(0.83%)p99.99(99.16%)d 0.02(0.01%)
7.554% C 32 s(0.05%)p99.99(99.70%)d 4.70(0.24%)
6.766% C 33 s(0.13%)p99.99(99.67%)d 1.49(0.20%)
26.497% C 34 s(0.03%)p99.99(99.96%)d 0.36(0.01%)
7.645% C 35 s(0.06%)p99.99(99.78%)d 2.70(0.16%)

Zr-Me interaction:
45. (2.00000) 97.3778% BD (1)Zr 1- C 12
20.612% Zr 1 s(10.94%)p 0.00(0.05%)d 8.14(89.01%)
76.778% C 12 s(26.35%)p 2.79(73.64%)d 0.00(0.01%)

Resonance structure **4**:
C-F interaction:
83. (2.00000) 99.5808% BD (1) C 31- F 40
27.466% C 31 s(22.39%)p 3.45(77.26%)d 0.02(0.34%)
72.126% F 40 s(28.96%)p 2.45(70.99%)d 0.00(0.05%)

C-C interaction:
79. (2.00000) 98.3072% BD (1) C 30- C 31
48.862% C 30 s(28.59%)p 2.49(71.34%)d 0.00(0.07%)
49.553% C 31 s(37.17%)p 1.69(62.80%)d 0.00(0.03%)
80. (2.00000) 82.6211% BD (2) C 30- C 31
3.422% Zr 1 s(16.39%)p 0.07(1.22%)d 5.03(82.38%)
38.048% C 30 s(0.12%)p99.99(99.82%)d 0.47(0.06%)
44.588% C 31 s(0.81%)p99.99(99.15%)d 0.05(0.04%)
4.781% C 32 s(0.11%)p99.99(99.53%)d 3.27(0.36%)
1.599% C 33 s(0.18%)p99.99(99.59%)d 1.27(0.23%)
1.324% C 34 s(0.09%)p99.99(99.59%)d 3.41(0.32%)
5.005% C 35 s(0.02%)p99.99(99.67%)d17.79(0.32%)

Zr-Me interaction:
44. (2.00000) 97.3758% BD (1)Zr 1- C 12
20.619% Zr 1 s(10.89%)p 0.00(0.05%)d 8.18(89.06%)
76.768% C 12 s(26.33%)p 2.80(73.67%)d 0.00(0.01%)

Zr-F interaction:

```
  45. (2.00000)  99.2828% BD ( 1)Zr  1- F 40
                          0.678% Zr  1 s( 34.75%)p 0.00(  0.08%)d 1.88( 65.17%)
                         98.612%  F 40 s( 70.04%)p 0.43( 29.96%)d 0.00(  0.00%)
```

Non-Covalent Interactions (NCI)

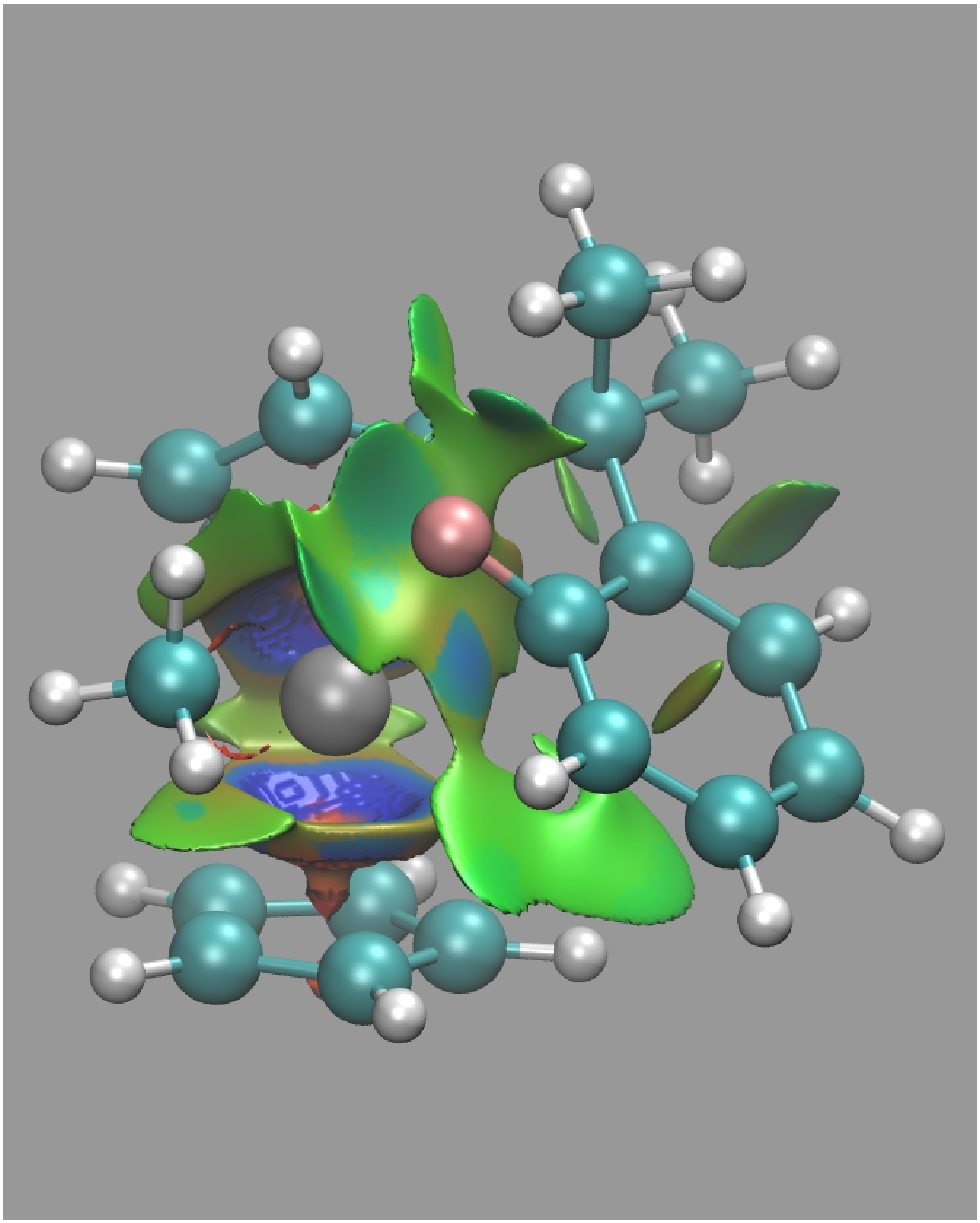

6B-PBE-D3

Bader:

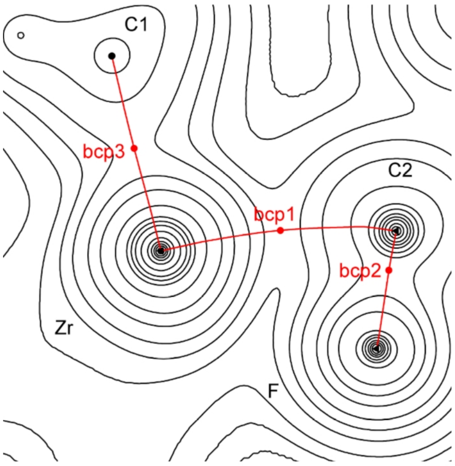

Electrondensity

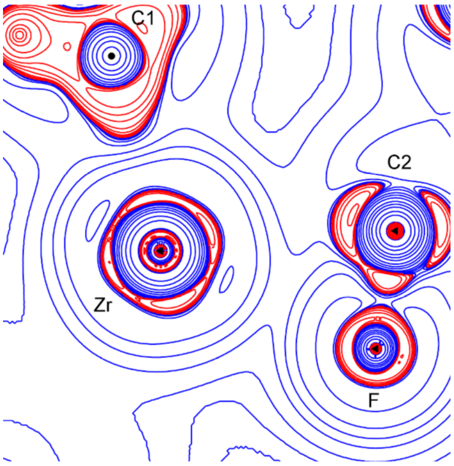

Laplacian

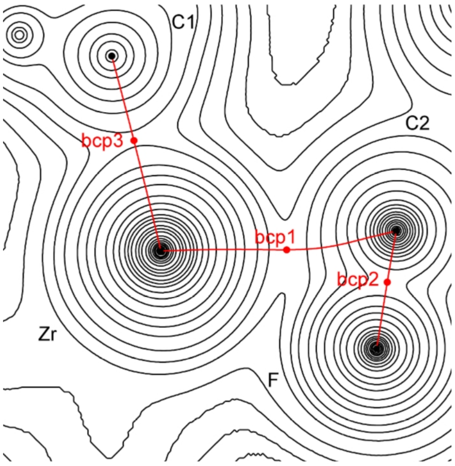

Virial

	ϱ(**r**)	∇^2ϱ(**r**)
bcp1	0.02926	-0.02136
bcp2	0.25621	-0.06718
bcp3	0.09550	-0.01136

NBO:

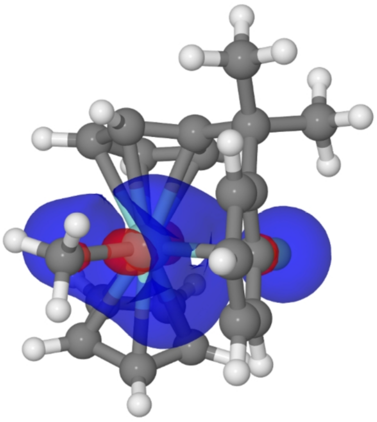

1

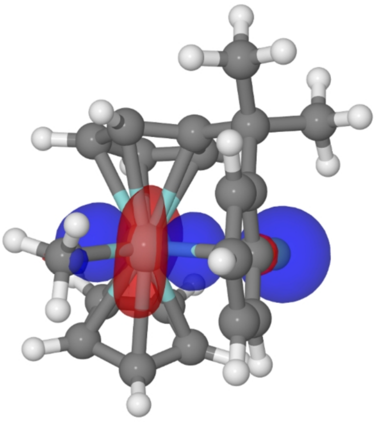

2

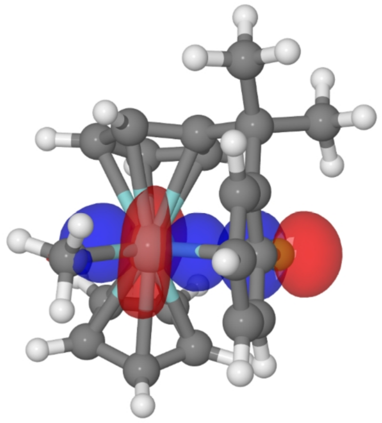

3

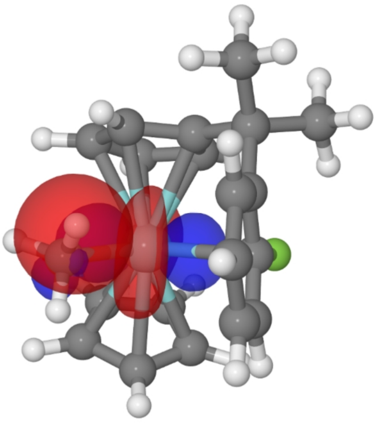

4

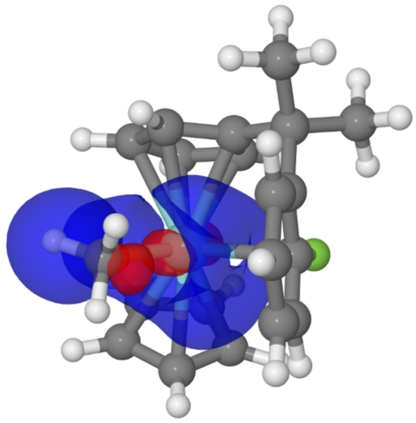

5

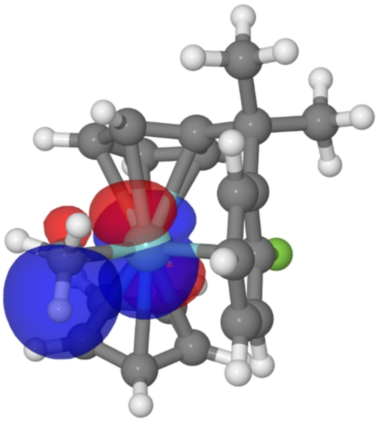

6

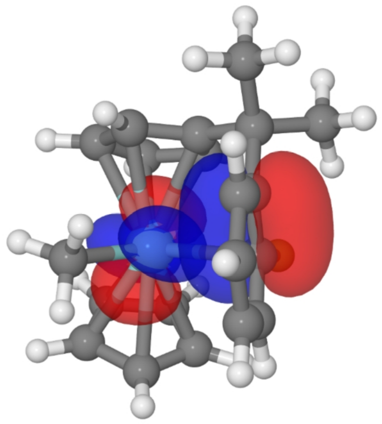

7

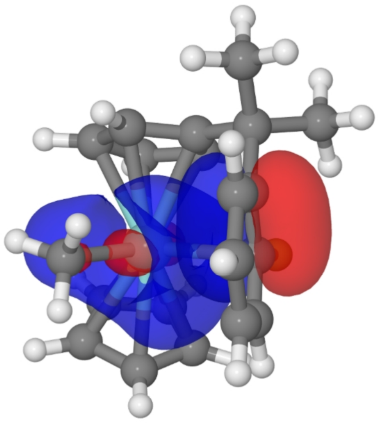

8

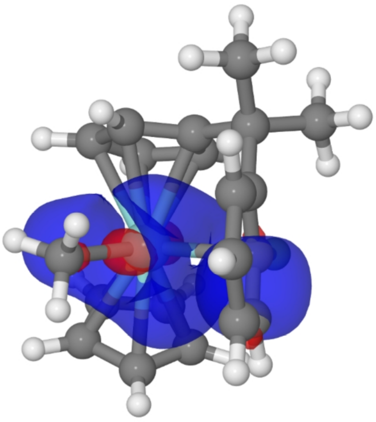

9

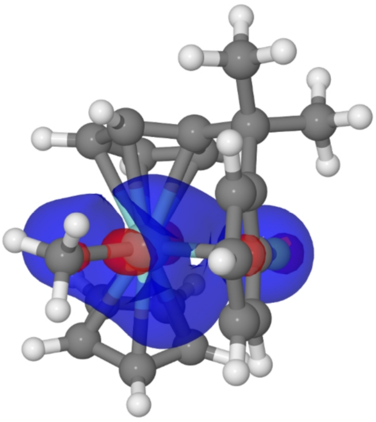

10

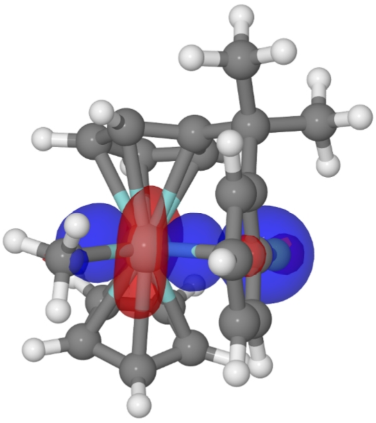

11

	Orbitals	E(2P)
1	$LP_F = sp^{0.50} \rightarrow$ $LV_{Zr} = sd^{0.18}$	4.94
2	$LP_F = sp^{0.50} \rightarrow$ $LV_{Zr} = sd^{45.37}$	3.27
3	$LP_F = sp^{78.26} \rightarrow$ $LV_{Zr} = sd^{45.37}$	2.42
4	$\sigma_{CH} = 0.781(sp^{3.02})_{C12} - 0.625(s)_{H13} \rightarrow$ $LV_{Zr} = sd^{45.37}$	2.37
5	$\sigma_{CH} = 0.786(sp^{3.14})_{C12} - 0.619(s)_{H14} \rightarrow$ $LV_{Zr} = sd^{0.18}$	2.13
6	$\sigma_{CH} = 0.781(sp^{2.95})_{C12} - 0.624(s)_{H15} \rightarrow$ $LV_{Zr} = sp^{0.14}d^{99.99}$	2.10
7	$\pi_{CC} = 0.662(p)_{C28} - 0.750(p)_{C29} \rightarrow$ $LV_{Zr} = sp^{0.16}d^{99.99}$	5.26
8	$\pi_{CC} = 0.662(p)_{C28} - 0.750(p)_{C29} \rightarrow$ $LV_{Zr} = sd^{0.18}$	2.85
9	$\sigma_{CH} = 0.713(sp^{1.59})_{C29} - 0.701(sp^{1.91})_{C30} \rightarrow$ $LV_{Zr} = sd^{0.18}$	2.82
10	$\sigma_{CF} = 0.521(sp^{3.80})_{C29} - 0.854(sp^{2.13})_{F} \rightarrow$ $LV_{Zr} = sd^{0.18}$	3.80
11	$\sigma_{CF} = 0.521(sp^{3.80})_{C29} - 0.854(sp^{2.13})_{F} \rightarrow$ $LV_{Zr} = sd^{45.37}$	2.56

Natural Resonance Theory:

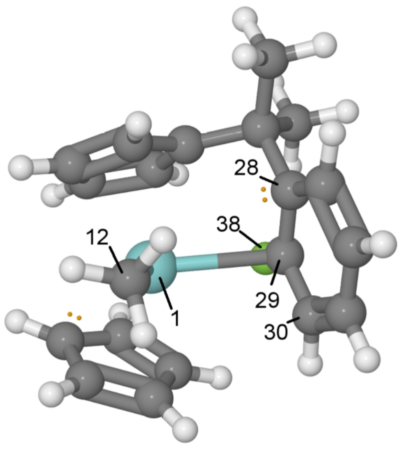

1

Wgt=17.63%;
rhoNL=5.94969;
D(0)=0.10218

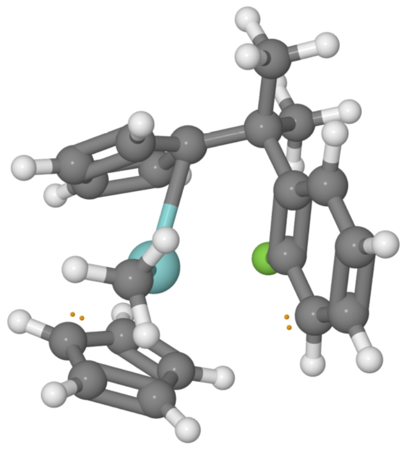

2

Wgt=15.49%;
rhoNL=5.88611;
D(0)=0.10163

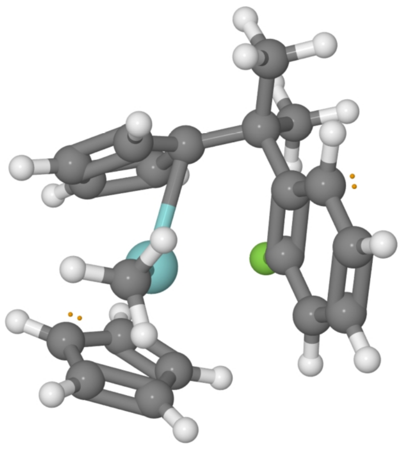

3

Wgt=13.36%;
rhoNL=5.90573;
D(0)=0.10180

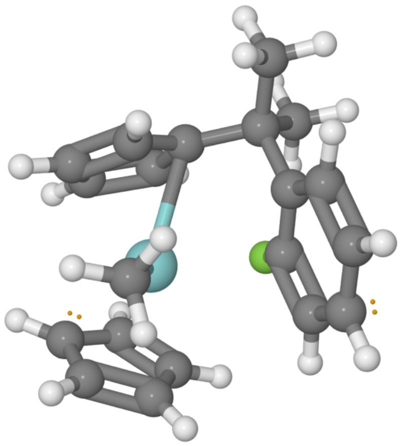

4

Wgt=10.18%;
rhoNL=5.88472;
D(0)=0.10162

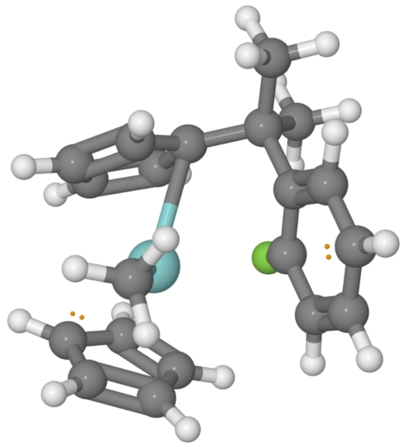

5

Wgt=10.16%;
rhoNL=5.92548;
D(0)=0.10197

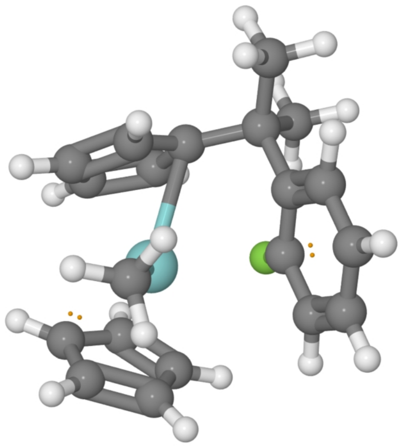

6

Wgt=9.64%;
rhoNL=5.72564;
D(0)=0.10023

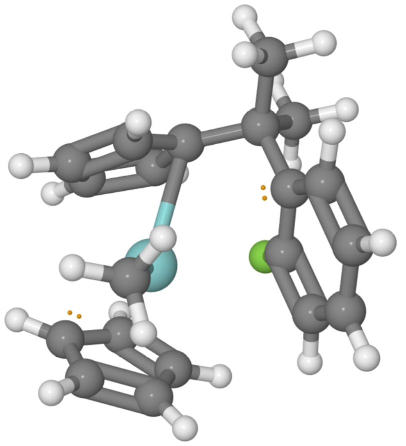	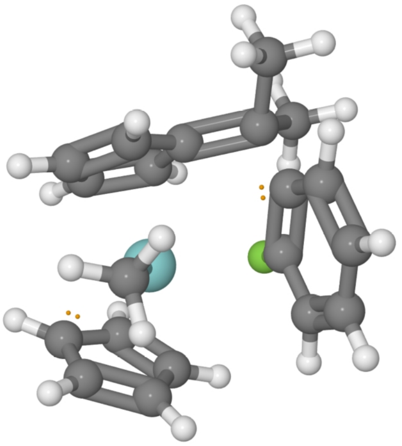
**7**	**8**
Wgt=6.84%;<br>rhoNL=5.86347;<br>D(0)=0.10143	Wgt=6.05%;<br>rhoNL=6.25953;<br>D(0)=0.10480

Natural Localised Molecular Orbitals (NLMO):

Only contributions over 1% are reported.

NLMO / Occupancy / Percent from Parent NBO / Atomic Hybrid Contributions

Resonance structure **1**:
NLMO algorithm failed to converge

Resonance structure **2**:
C-F interaction:

```
  84. (2.00000)  99.5496% BD ( 1) C 29- F 38
                 26.965%  C 29 s( 21.88%)p 3.55( 77.76%)d 0.02(  0.36%)
                 72.598%  F 38 s( 31.61%)p 2.16( 68.34%)d 0.00(  0.05%)
```

C-C interaction:

```
  80. (2.00000)  98.3347% BD ( 1) C 28- C 29
                 48.717%  C 28 s( 28.45%)p 2.51( 71.48%)d 0.00(  0.07%)
                 49.736%  C 29 s( 37.35%)p 1.68( 62.63%)d 0.00(  0.02%)
  81. (2.00000)  82.7946% BD ( 2) C 28- C 29
                  2.464% Zr  1 s( 16.17%)p 0.04(  0.71%)d 5.14( 83.12%)
                 36.923%  C 28 s(  0.16%)p99.99( 99.79%)d 0.34(  0.05%)
                 45.893%  C 29 s(  1.06%)p93.32( 98.91%)d 0.02(  0.03%)
                  5.345%  C 30 s(  0.09%)p99.99( 99.57%)d 3.53(  0.33%)
                  1.252%  C 31 s(  0.14%)p99.99( 99.58%)d 2.11(  0.29%)
```

Zr-Me interaction:

```
  45. (2.00000)  97.6029% BD ( 1)Zr  1- C 12
                 20.170% Zr  1 s( 10.66%)p 0.00(  0.05%)d 8.38( 89.29%)
                 77.441%  C 12 s( 26.04%)p 2.84( 73.96%)d 0.00(  0.01%)
```

Resonance structure **3**:
C-F interaction:

```
  84. (2.00000)  99.5495% BD ( 1) C 29- F 38
                 26.966%  C 29 s( 21.88%)p 3.55( 77.76%)d 0.02(  0.36%)
                 72.598%  F 38 s( 31.61%)p 2.16( 68.34%)d 0.00(  0.05%)
```

C-C interaction:

```
  80. (2.00000)  98.3352% BD ( 1) C 28- C 29
                 48.716%  C 28 s( 28.45%)p 2.51( 71.48%)d 0.00(  0.07%)
                 49.735%  C 29 s( 37.34%)p 1.68( 62.64%)d 0.00(  0.02%)
  81. (2.00000)  82.7546% BD ( 2) C 28- C 29
                  2.538% Zr  1 s( 16.32%)p 0.04(  0.72%)d 5.08( 82.96%)
                 35.514%  C 28 s(  0.16%)p99.99( 99.78%)d 0.35(  0.06%)
                 47.267%  C 29 s(  1.06%)p93.10( 98.91%)d 0.02(  0.02%)
                  6.248%  C 30 s(  0.09%)p99.99( 99.62%)d 3.37(  0.29%)
                  1.113%  C 31 s(  0.15%)p99.99( 99.49%)d 2.48(  0.36%)
                  5.076%  C 33 s(  0.03%)p99.99( 99.67%)d11.64(  0.30%)
```

Zr-Me interaction:
45. (2.00000) 97.6023% BD (1)Zr 1- C 12
20.173% Zr 1 s(10.66%)p 0.00(0.05%)d 8.38(89.30%)
77.438% C 12 s(26.04%)p 2.84(73.96%)d 0.00(0.01%)

Resonance structure **4**:
C-F interaction:
84. (2.00000) 99.5464% BD (1) C 29- F 38
26.962% C 29 s(21.86%)p 3.56(77.78%)d 0.02(0.36%)
72.600% F 38 s(31.61%)p 2.16(68.34%)d 0.00(0.05%)

C-C interaction:
82. (2.00000) 98.7316% BD (1) C 29- C 30
50.272% C 29 s(36.29%)p 1.76(63.69%)d 0.00(0.03%)
48.563% C 30 s(31.75%)p 2.15(68.19%)d 0.00(0.06%)
83. (2.00000) 83.7032% BD (2) C 29- C 30
2.518% Zr 1 s(19.05%)p 0.05(0.87%)d 4.20(80.08%)
5.738% C 28 s(0.38%)p99.99(99.30%)d 0.83(0.32%)
47.680% C 29 s(1.06%)p93.45(98.92%)d 0.02(0.02%)
36.052% C 30 s(0.03%)p99.99(99.92%)d 1.46(0.05%)
4.440% C 31 s(0.05%)p99.99(99.61%)d 7.03(0.34%)
1.400% C 32 s(0.08%)p99.99(99.70%)d 2.94(0.23%)
1.427% C 33 s(0.11%)p99.99(99.65%)d 2.23(0.24%)

Zr-Me interaction:
45. (2.00000) 97.6037% BD (1)Zr 1- C 12
20.170% Zr 1 s(10.67%)p 0.00(0.05%)d 8.37(89.29%)
77.441% C 12 s(26.04%)p 2.84(73.96%)d 0.00(0.01%)

Resonance structure **5**:
C-F interaction:
84. (2.00000) 99.5672% BD (1) C 29- F 38
27.018% C 29 s(22.00%)p 3.53(77.64%)d 0.02(0.35%)
72.554% F 38 s(31.58%)p 2.16(68.37%)d 0.00(0.05%)

C-C interaction:
80. (2.00000) 98.3223% BD (1) C 28- C 29
48.702% C 28 s(28.43%)p 2.52(71.50%)d 0.00(0.07%)
49.743% C 29 s(37.45%)p 1.67(62.53%)d 0.00(0.02%)
83. (2.00000) 98.7237% BD (1) C 29- C 30
50.278% C 29 s(36.36%)p 1.75(63.62%)d 0.00(0.03%)
48.553% C 30 s(31.75%)p 2.15(68.19%)d 0.00(0.06%)

Zr-Me interaction:
45. (2.00000) 97.6015% BD (1)Zr 1- C 12
20.174% Zr 1 s(10.64%)p 0.00(0.05%)d 8.39(89.31%)
77.435% C 12 s(26.04%)p 2.84(73.95%)d 0.00(0.01%)

Resonance structure **6**:
C-F interaction:
84. (2.00000) 99.5671% BD (1) C 29- F 38
27.017% C 29 s(22.00%)p 3.53(77.64%)d 0.02(0.36%)
72.554% F 38 s(31.59%)p 2.16(68.36%)d 0.00(0.05%)

C-C interaction:
80. (2.00000) 98.3229% BD (1) C 28- C 29
48.703% C 28 s(28.43%)p 2.51(71.50%)d 0.00(0.07%)
49.742% C 29 s(37.43%)p 1.67(62.54%)d 0.00(0.02%)
83. (2.00000) 98.7238% BD (1) C 29- C 30
50.276% C 29 s(36.35%)p 1.75(63.62%)d 0.00(0.03%)
48.556% C 30 s(31.75%)p 2.15(68.19%)d 0.00(0.06%)

Zr-Me interaction:

45. (2.00000) 97.6037% BD (1)Zr 1- C 12
20.167% Zr 1 s(10.67%)p 0.00(0.05%)d 8.37(89.29%)
77.445% C 12 s(26.04%)p 2.84(73.96%)d 0.00(0.01%)

Resonance structure **7**:
C-F interaction:
84. (2.00000) 99.5464% BD (1) C 29- F 38
26.961% C 29 s(21.88%)p 3.55(77.76%)d 0.02(0.36%)
72.601% F 38 s(31.61%)p 2.16(68.34%)d 0.00(0.05%)

C-C interaction:
82. (2.00000) 98.7317% BD (1) C 29- C 30
50.272% C 29 s(36.28%)p 1.76(63.69%)d 0.00(0.03%)
48.563% C 30 s(31.75%)p 2.15(68.19%)d 0.00(0.06%)
83. (2.00000) 80.5490% BD (2) C 29- C 30
1.977% Zr 1 s(19.61%)p 0.05(0.94%)d 4.05(79.45%)
37.472% C 29 s(1.03%)p95.77(98.93%)d 0.04(0.04%)
43.864% C 30 s(0.03%)p99.99(99.94%)d 1.20(0.03%)
10.104% C 31 s(0.05%)p99.99(99.78%)d 3.16(0.17%)
4.458% C 33 s(0.06%)p99.99(99.92%)d 0.30(0.02%)

Zr-Me interaction:
45. (2.00000) 97.6024% BD (1)Zr 1- C 12
20.172% Zr 1 s(10.66%)p 0.00(0.05%)d 8.38(89.29%)
77.439% C 12 s(26.04%)p 2.84(73.96%)d 0.00(0.01%)

Resonance structure **8**:
C-F interaction:
83. (2.00000) 99.5494% BD (1) C 29- F 38
26.969% C 29 s(21.88%)p 3.55(77.76%)d 0.02(0.36%)
72.595% F 38 s(31.61%)p 2.16(68.34%)d 0.00(0.05%)

C-C interaction:

```
  79. (2.00000)  98.3903% BD ( 1) C 28- C 29
                 48.818%  C 28 s( 27.12%)p 2.69( 72.82%)d 0.00(  0.07%)
                 49.654%  C 29 s( 37.45%)p 1.67( 62.53%)d 0.00(  0.02%)
  80. (2.00000)  82.8973% BD ( 2) C 28- C 29
                  2.498% Zr  1 s( 16.17%)p 0.05(  0.75%)d 5.14( 83.08%)
                 36.484%  C 28 s(  0.17%)p99.99( 99.77%)d 0.31(  0.05%)
                 46.441%  C 29 s(  1.04%)p95.34( 98.94%)d 0.02(  0.03%)
                  5.061%  C 30 s(  0.12%)p99.99( 99.52%)d 3.08(  0.36%)
                  1.772%  C 31 s(  0.13%)p99.99( 99.67%)d 1.50(  0.20%)
                  1.314%  C 32 s(  0.10%)p99.99( 99.58%)d 3.24(  0.32%)
                  5.135%  C 33 s(  0.02%)p99.99( 99.67%)d18.15(  0.31%)
```

Zr-Me interaction:

```
  45. (2.00000)  97.6039% BD ( 1)Zr  1- C 12
                 20.191% Zr  1 s( 10.68%)p 0.00(  0.05%)d 8.36( 89.27%)
                 77.421%  C 12 s( 26.04%)p 2.84( 73.95%)d 0.00(  0.01%)
```

Non-Covalent Interactions (NCI)

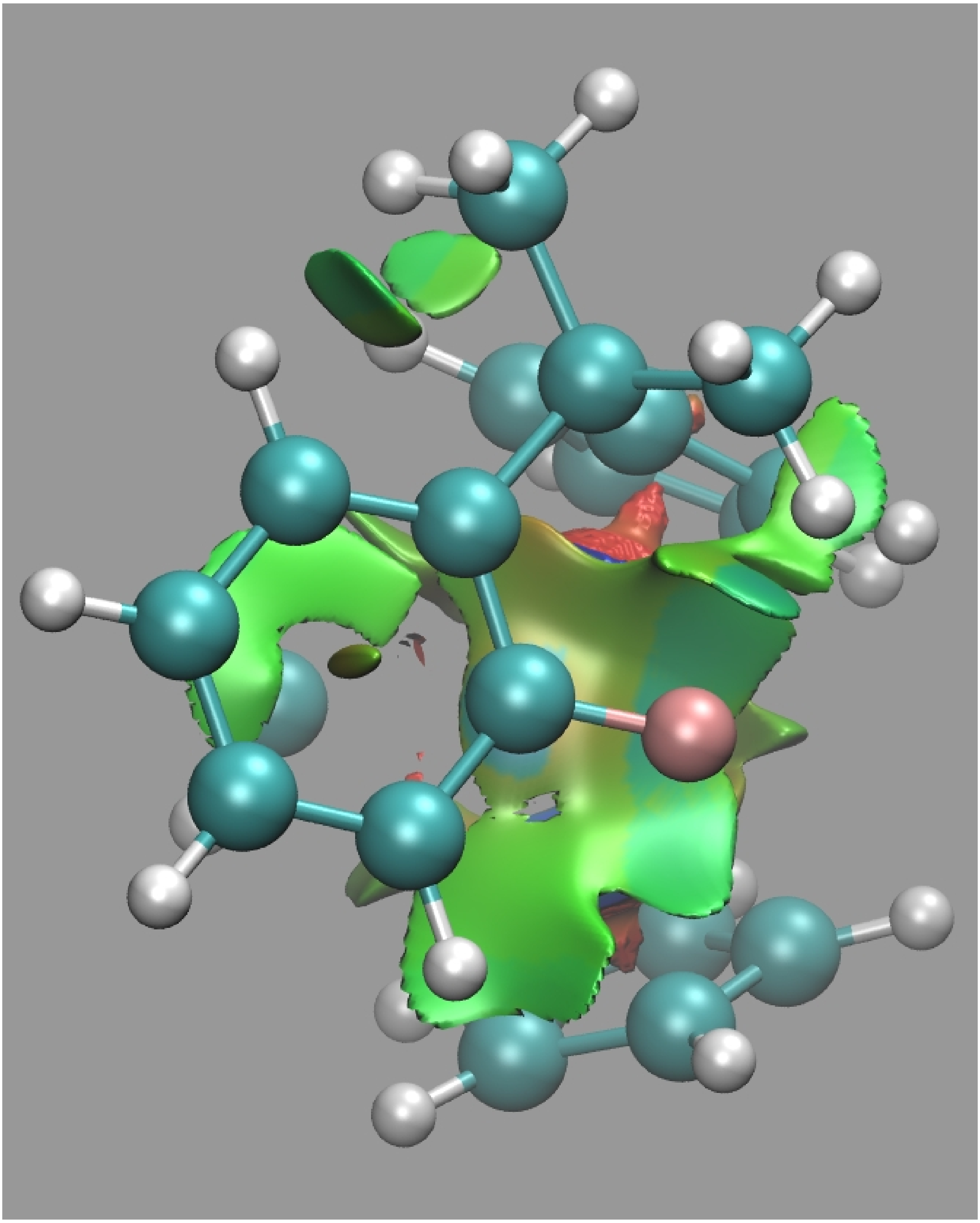

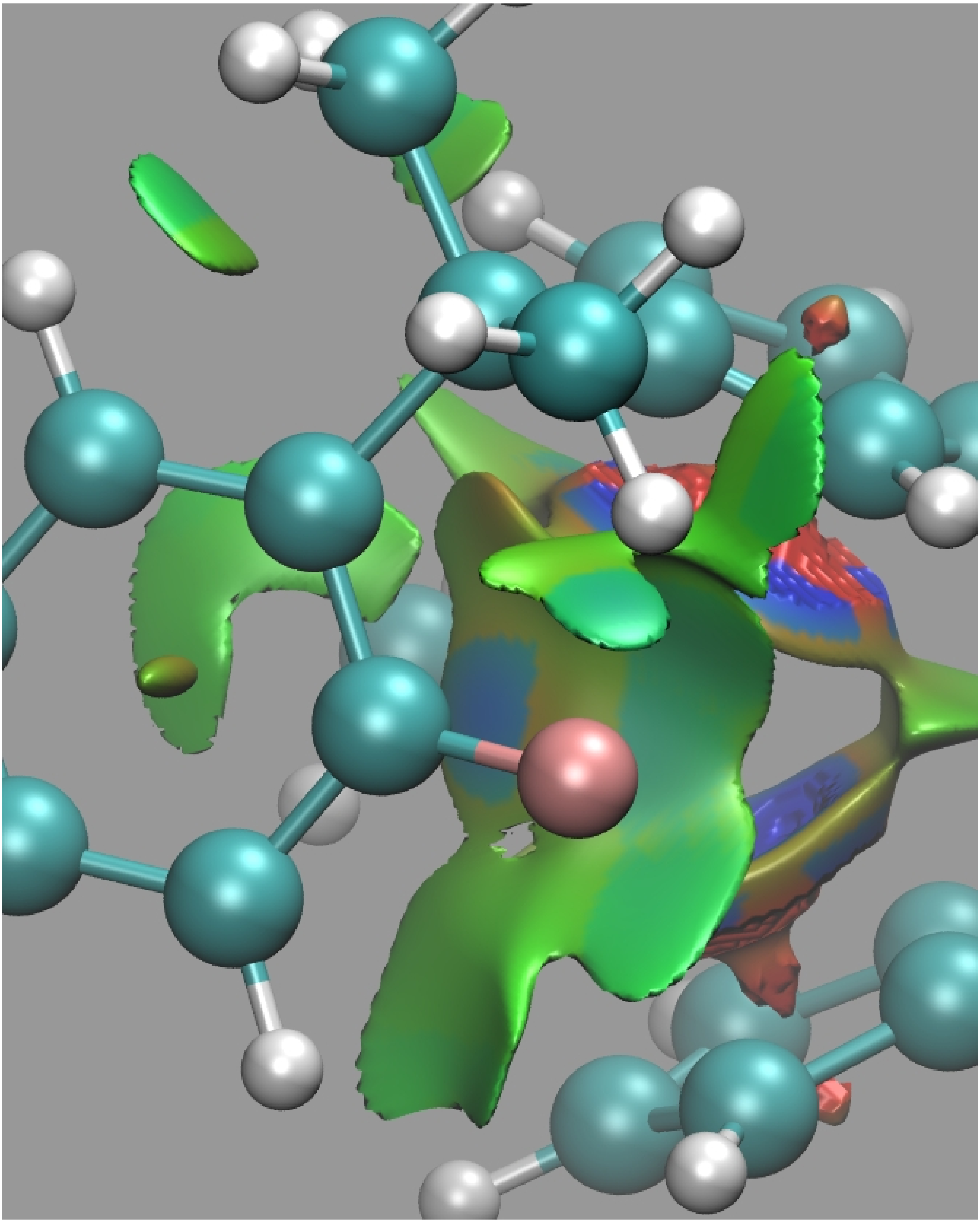

Miscellaneous

Correlation graphs of selected compounds and properties

Graph S1: Bader and NBO charges vs. selected compounds

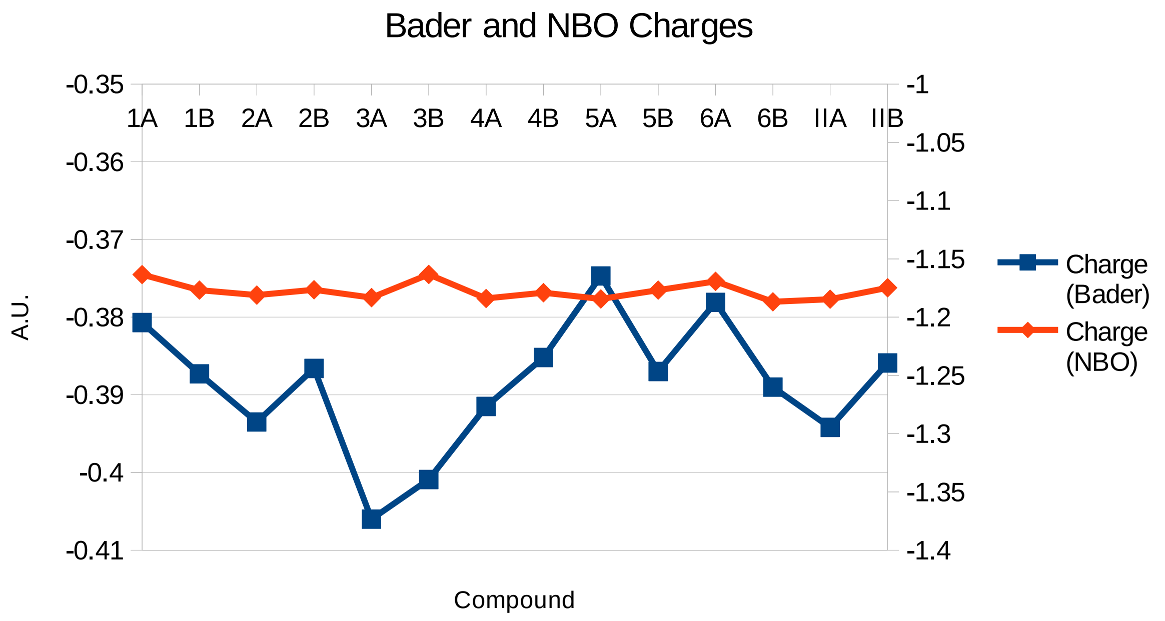

Graph S2: Bader charges and Zr-Me bond distances vs. selected compounds

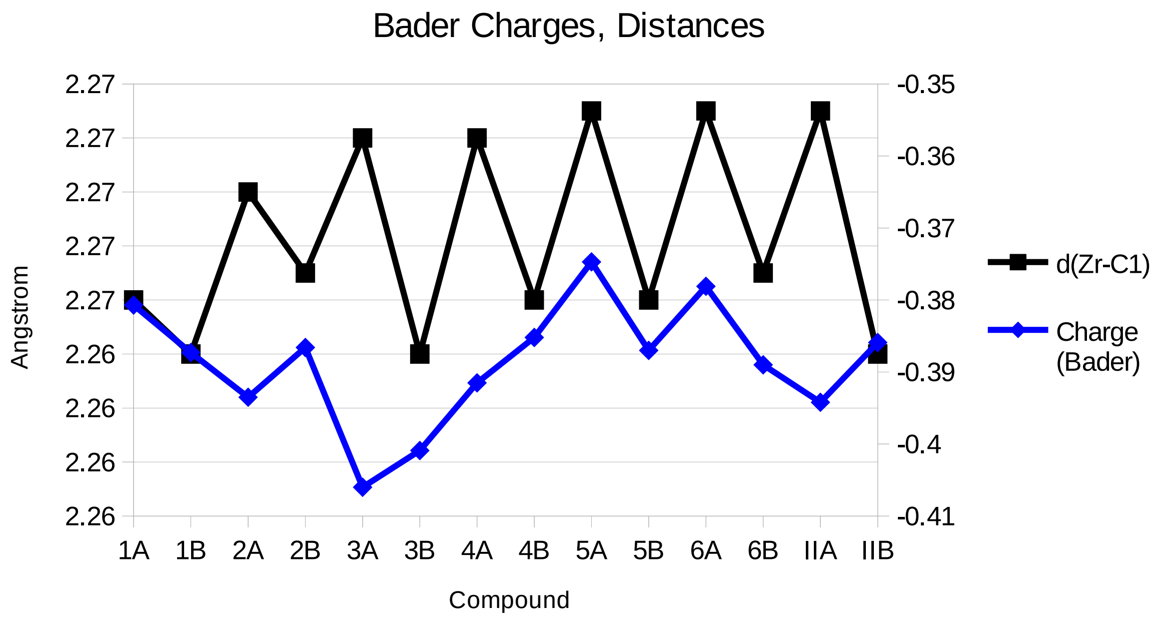

Graph S3: Wiberg Bond Index and Zr-Me bond distances vs. selected compounds

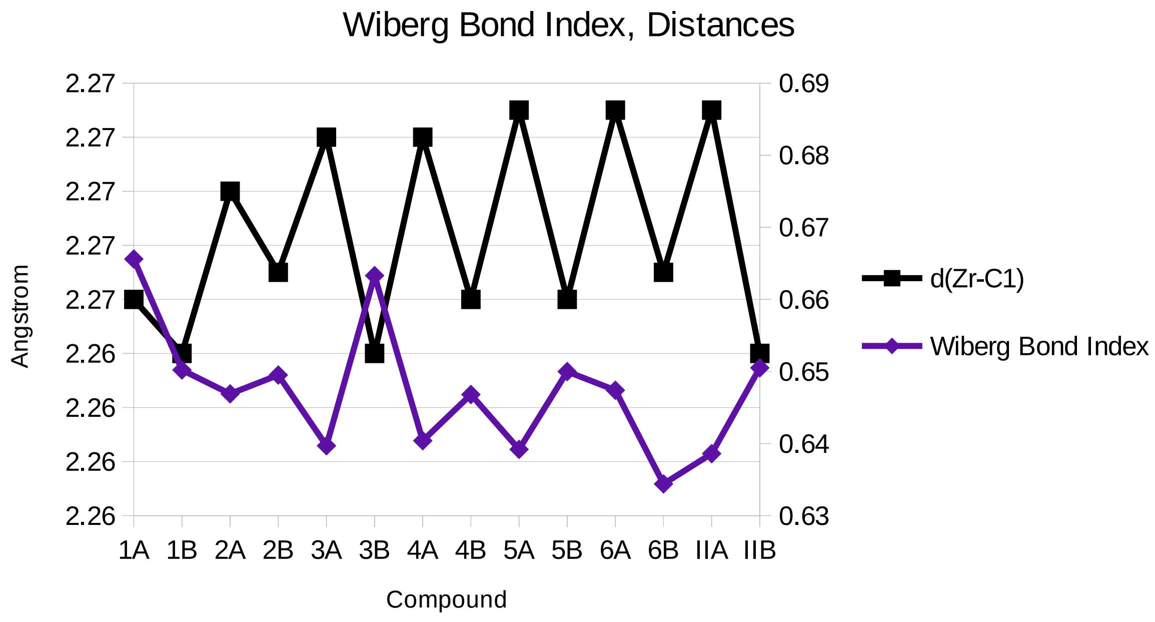

Graph S4: Natural Binding Index and Zr-Me bond distances vs. selected compounds

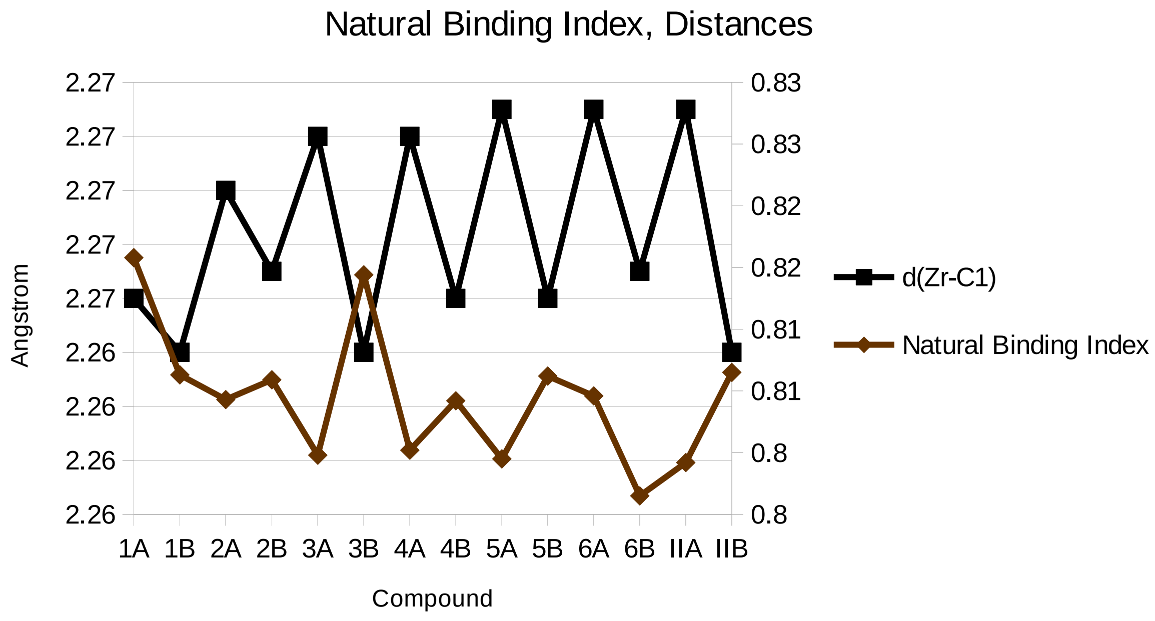

Graph S5: Bond Critical Point and Zr-Me bond distances vs. selected compounds

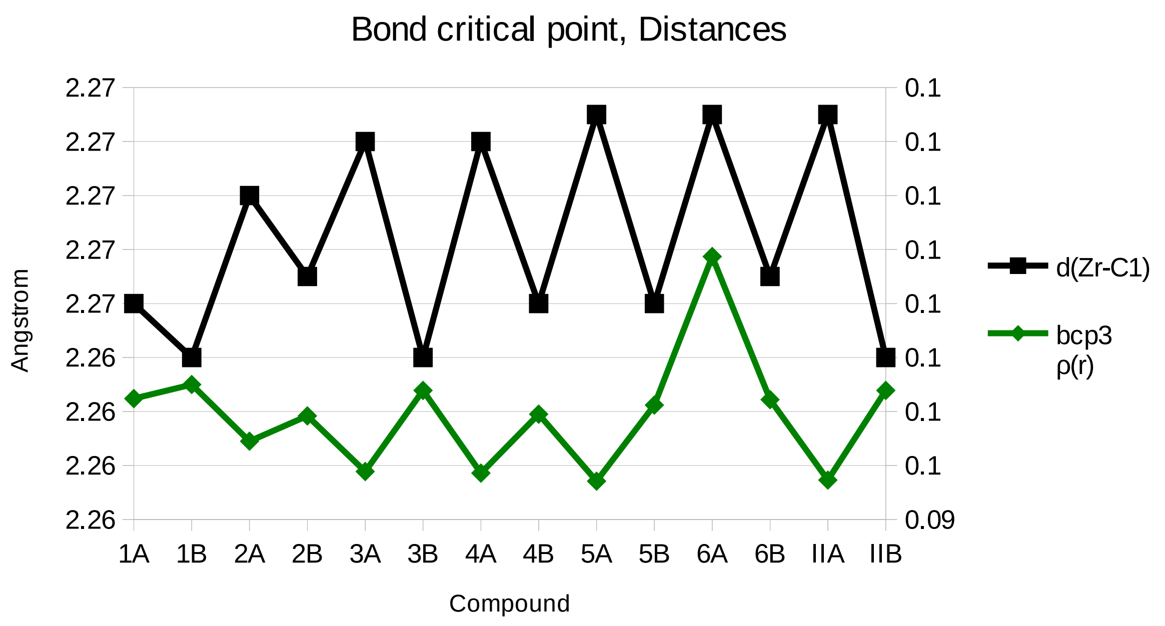

Graph S6: Calculated ^{13}C chemical shifts and Zr-Me bond distances vs. selected compounds

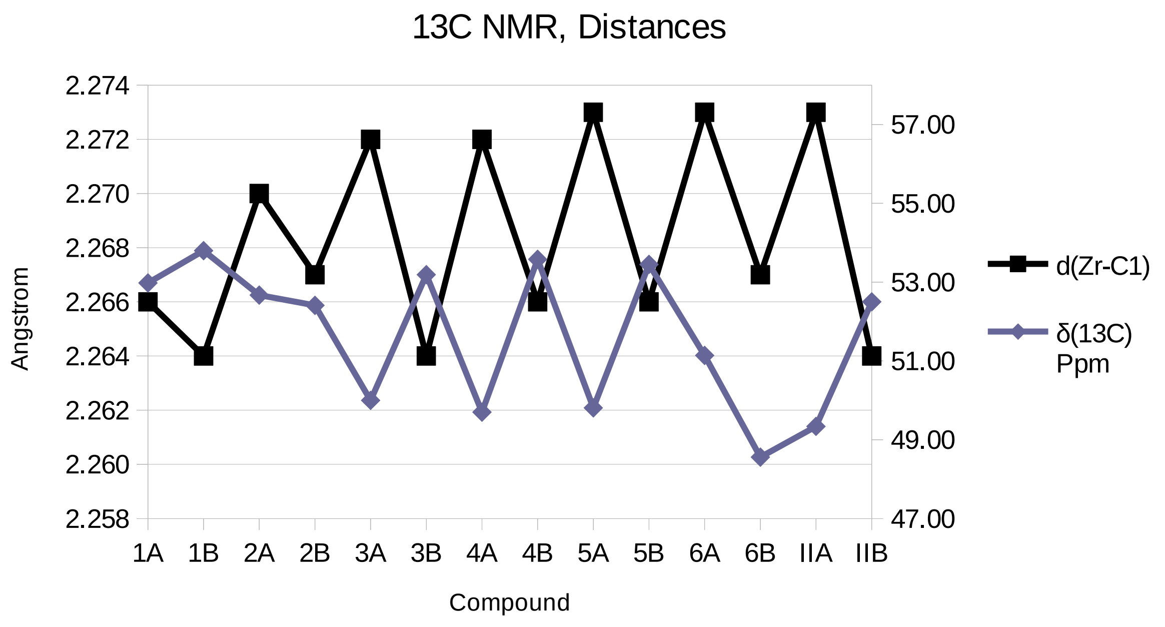

Supplement: Supplementary file 1 — ao2c04053_si_001.pdf [file ao2c04053_si_001.pdf]
